# Influence of a Non-Hospital Medical Care Facility on Antimicrobial Resistance in Wastewater

**DOI:** 10.1371/journal.pone.0122635

**Published:** 2015-03-30

**Authors:** Mathias Bäumlisberger, Loubna Youssar, Markus B. Schilhabel, Daniel Jonas

**Affiliations:** 1 Institute for Environmental Health Sciences and Hospital Infection Control, Medical Center—University of Freiburg, Freiburg, Germany; 2 Institute of Clinical Molecular Biology, Christian-Albrechts-University Kiel, University Hospital Schleswig Holstein, Campus Kiel, Kiel, Germany; University Medical Center Utrecht, NETHERLANDS

## Abstract

The global widespread use of antimicrobials and accompanying increase in resistant bacterial strains is of major public health concern. Wastewater systems and wastewater treatment plants are considered a niche for antibiotic resistance genes (ARGs), with diverse microbial communities facilitating ARG transfer via mobile genetic element (MGE). In contrast to hospital sewage, wastewater from other health care facilities is still poorly investigated. At the instance of a nursing home located in south-west Germany, in the present study, shotgun metagenomics was used to investigate the impact on wastewater of samples collected up- and down-stream in different seasons. Microbial composition, ARGs and MGEs were analyzed using different annotation approaches with various databases, including Antibiotic Resistance Ontologies (ARO), integrons and plasmids. Our analysis identified seasonal differences in microbial communities and abundance of ARG and MGE between samples from different seasons. However, no obvious differences were detected between up- and downstream samples. The results suggest that, in contrast to hospitals, sewage from the nursing home does not have a major impact on ARG or MGE in wastewater, presumably due to much less intense antimicrobial usage. Possible limitations of metagenomic studies using high-throughput sequencing for detection of genes that seemingly confer antibiotic resistance are discussed.

## Introduction

Antimicrobial consumption is constantly increasing, both in human and veterinary medicine, leading to a concomitant rise in antibiotic resistant bacteria [1]. It has been reported that antimicrobial resistance evolves in so called “genetic reactors” [2], e.g. farms, hospitals and long-term healthcare facilities (LTCF), which then act as a niche for bacterial exchange between individuals. The large quantities of antibiotics used in healthcare facilities exert a selection pressure on the microbial community. Furthermore, health care facilities emit the resistant bacteria that have evolved into the sewage system, where another potential recruitment pool for antibiotic resistant human pathogens can emerge [3,4]. Recent studies have reported on analyses of antibiotic resistant bacteria and resistance genes in sewage from hospitals [5–7], slaughterhouses and farms [8,9]. Despite the fact that resistant bacteria like methicillin-resistant *Staphylococcus aureus*, vancomycin-resistant enterococci and other multidrug-resistant enterics are endemic in the residents of LTCF [10–13], little is known about the release of resistant bacteria into the water cycle in sewage from LTCF. Indeed, in some countries the ageing population has prompted a considerable increase in the number of nursing homes. Thus, an investigation of corresponding wastewater systems is of interest.

In this study, a metagenomic approach was used to survey the taxonomic composition and content of resistance determinants in wastewater up- and downstream from a nursing home in south-west Germany. We identified differences in microbial communities in samples from different seasons but no obvious differences between the up- and downstream samples. Our results may suggest that the nursing home investigated has no marked influence on the wastewater system.

## Materials and Methods

### Ethics statement

The nursing home operator and the municipality responsible for the combined sewer system granted permission to analyze wastewater.

### Water sampling

Sewage samples were collected from the combined sewer of a rural village (800 inhabitants) without any industry in south-west Germany (N: 48.40, E: 8.01). The water-samples were collected from two interconnected sites, one upstream (sample names: C1754, C1755), i.e. municipal sewage from the village, and the other 75 meters downstream, i.e. after wastewater discharge from a nursing home (280 residents) to the sewer system (sample names: C1756, C1757). According to the German Association for Water, Wastewater and Waste [14], the volume of wastewater calculated per day downstream of the nursing home is approximately 204 m^3^, of which roughly 25% originates from the nursing home.

The samples, each of which was taken every two hours over a 24-h period, were combined as “24-h composite samples”. To control for seasonal variations, samples were collected twice during the course of this study in November 2012 (November samples) and March 2013 (March samples).

### DNA extraction and sequencing

The samples were stored and transported at + 4°C and processed immediately. Nine hundred ml were prepared for DNA extraction according to Lemarchand et al. [15]. Total DNA was extracted by use of the NucleoSpin-Soil Kit (Macherey-Nagel) and the concentrations were measured using a Qubit 2.0 Fluorometer and the Qubit dsDNA BR assay kit (Invitrogen). Additionally, gel electrophoresis was run to confirm the DNA integrity. The purified DNA was shipped to the ICMB (Institute of Clinical Molecular Biology, University of Kiel; Germany) for High-Throughput sequencing employing an Illumina-HighSeq sequencer (paired end sequencing; 101-bp reads; 6-bp Index sequence). The Illumina reads were preprocessed by means of the CASAVA-1.8.2. pipeline. Raw reads data have been deposited at the sequence read archive (SRA) of NCBI under Accession number SAMN02725022, SAMN02725023, SAMN02725024 and SAMN02725025.

### Bioinformatics

For automated taxonomic and functional profiling, the sequences were submitted to MG-RAST (Meta Genome Rapid Annotation using Subsystem Technology, v3.2.2; website http://metagenomics.anl.gov; last access 16.06.2014) [16]. Subsequently, replicate sequences and reads with five or more ambiguous bases were eliminated.

### Taxonomic analysis

The reads, clustered and identified as SSU-rDNA, were annotated against the M5RNA database in MG-RAST [17]. This database combined SSU-rDNA sequence databases RDP [18], Greengenes [19] and SILVA [20]. Taking a previous study [21], as reference, parameters in BLAT were adjusted to e-Value ≤ 10^–5^, identity ≥ 80% and alignment length of ≥ 50 bp.

### Functional annotation

All reads were annotated against the SEED subsystem by means of BLAT [22] with the following parameters: e-Value ≤10^–5^, identity ≥ 60% and alignment length of ≥ 15 amino-acids (aa). The subsystems, i.e. clusters of functional roles, were created by curators to analyze annotations on differently detailed levels. In this study, we scrutinized at level 1 the subsystem “Virulence, Defense and Diseases” (“VDD”), which includes especially the level 2 subsystem “Resistance to antibiotics and toxic compounds” (RATC).

### Assembly

CLC Genomic Workbench 6.5.1 was used to assemble the reads after removal of reads with more than 2 unknown bases. Assembly parameters were adjusted to a length fraction of ≥ 0.8, similarity to ≥ 0.95 and a minimum contig length of ≥ 250 bp [23].

### Antibiotic resistance genes and mobile genetic elements

For subsequent analyses, reads were re-filtered using FASTX–Toolkit available at the Galaxy platform [24]. Reads with unknown nucleotides or having a score lower than minimum Illumina quality score (75% of all bases higher than 30) were removed [25]. The obtained reads and assembled contigs were blasted against antibiotic resistance genes and mobile genetic elements databases.

To generate a local BLAST database with antibiotic resistance genes and a controlled vocabulary of antibiotic resistance ontologies (ARO), the corresponding amino acid sequences, tagged specifically for antibiotic resistance, were downloaded from the Comprehensive Antibiotic Resistance Database (CARD) [26] (1,845 sequences—04.12.2013). This database was blasted using a BLASTx [27,28], implemented on the Galaxy-based framework Orion [29], against the unassembled reads and contigs. For extraction of the best hits the parameters were set according to Kristiansson *et al*. [3], e-Value ≤ 10^–5^, identity ≥ 90% and alignment length ≥ 25 aa. To check for signatures of mobile genetic elements, two additional databases were created. The first one was developed with 1,511 integron integrase gene sequences from the INTEGRALL database (kindly provided by Alexandra Moura) [30]. This was locally blasted using BLASTn [26, 27] against the unassembled and assembled reads (e-Value ≤10^–5^, identity ≥ 90% and alignment length of ≥ 50 bp) and the best hit in each alignment was selected [3]. For detection of reads and contigs associated with plasmids, the plasmid database available in NCBI (4,402 sequences—01.04.2014) was blasted against reads and contigs using BLASTn (cutoff e-Value ≤10^–5^; minimum similarity ≥ 95; alignment length above ≥ 90) and the best hit in each case was selected [31]. All local blast analyses were run on the Galaxy-based framework Orion.

## Results

### Sequencing and taxonomy

**Fig 1:**
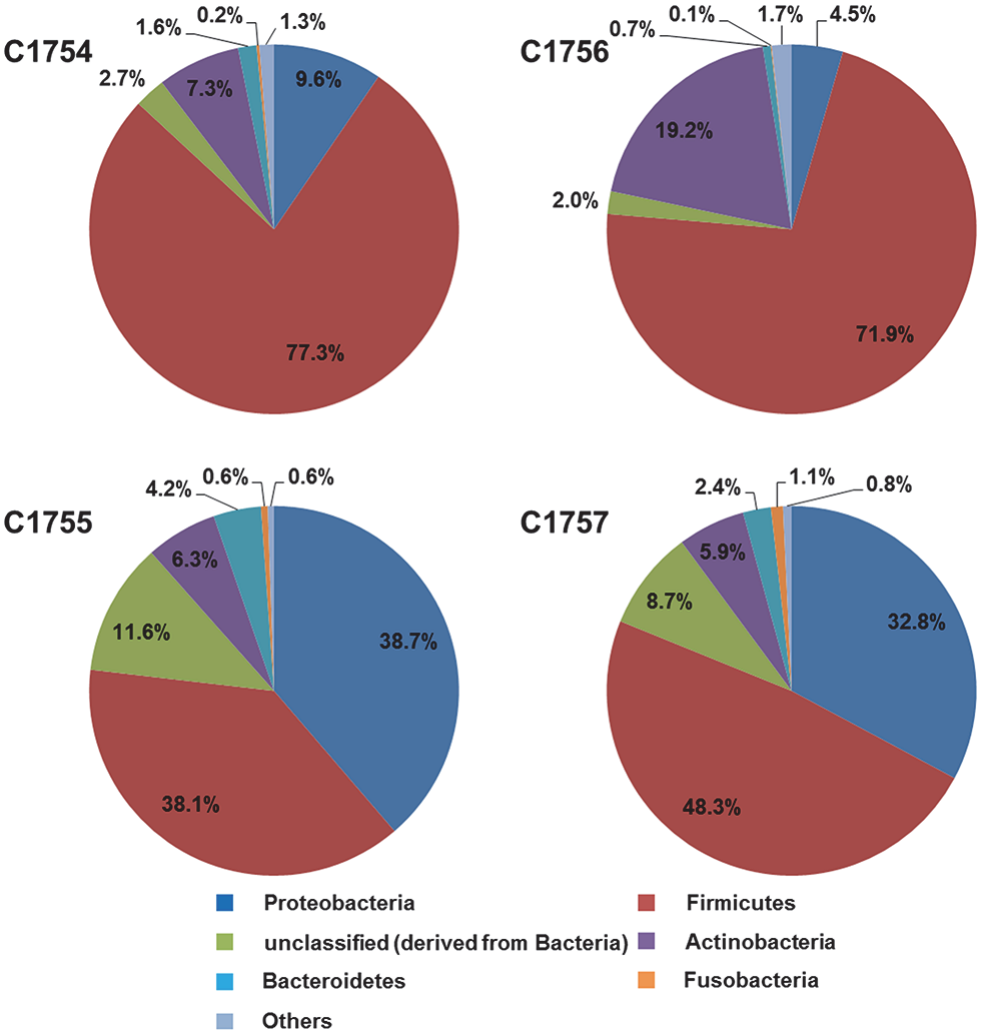
Phylogenetic compositions of the bacteria domain in the samples.

Illumina sequencing of the wastewater samples generated a total of 5.4 x 10^7^ reads. The read number varied largely between the samples. Further details on QC-results and assembling are depicted in S1 Table. Two percent (± 0.3%) of the quality filtered reads from samples C1754, C1756, C1755 and C1757 contained SSU ribosomal RNA genes. They were assigned in MG-RAST to the M5RNA database in order to compare their domains. In all samples, 94.39% (± 1.78%) of the reads were assigned to the bacteria domain. The remaining reads were mapped to eukaryotes (2.37% ± 0.86%), archaea (0.18% ± 0.14%) and the virus domains (0.14% ± 0.08%) (Table 1). A high percentage of the reads from both November samples was assigned to the phylum *Firmicutes* (C1754: 77.43%; C1756: 71.85%) followed to a much lower extent by the phyla *Actinobacteria* (7.2 7%; 19.19%), *Proteobacteria* (9.55%; 4.48%), *Bacteroidetes* (1.60%; 0.69%) and *Fusobacteria* (0.24%; 0.08%) (Fig 1). In contrast, in both March samples (C1755, C1757), a considerably lower percentage of SSU rRNA-reads were assigned to *Firmicutes* (38.12%; 48.32%) and to a comparatively larger extent to *Proteobacteria* (38.71%; 32.83%). Remaining reads were classified to *Actinobacteria* (7.27%; 5.89%), *Bacteroidetes* (4.15%; 2.44%), and *Fusobacteria* (0.59%; 1.05%) (Fig 1). rRNA sequences, derived by unclassified bacteria and by SSU ribosomal RNA from other phyla together contained 12.16% and 9.47%, respectively, of the remaining rDNA reads. In contrast to the samples taken from both interconnected sampling sites on the same date, the taxonomic assignment showed high differences in phyla arrangement between the November and March samples.

**Table 1:** Alphabetically ordered functional gene categories in the wastewater samples (MG-RAST SEED Level 1 Distribution).

Functional gene category	Percentage of assigned sequences			
	C1754	C1756	C1755	C1757
**Amino Acids and Derivatives**	8.35%	8.14%	8.94%	8.87%
**Carbohydrates**	11.74%	12.21%	10.01%	10.37%
**Cell Division and Cell Cycle**	1.73%	1.78%	1.51%	1.55%
**Cell Wall and Capsule**	3.54%	3.54%	3.43%	3.51%
**Clustering-based subsystems**	15.81%	16.19%	14.66%	14.88%
**Cofactors, Vitamins, Prosthetic Groups, Pigments**	5.90%	5.82%	6.70%	6.49%
**DNA Metabolism**	5.26%	5.37%	4.68%	4.79%
**Dormancy and Sporulation**	0.46%	0.43%	0.23%	0.28%
**Fatty Acids, Lipids, and Isoprenoids**	2.42%	2.39%	3.08%	2.92%
**Iron acquisition and metabolism**	0.66%	0.60%	0.65%	0.66%
**Membrane Transport**	3.09%	3.25%	3.36%	3.35%
**Metabolism of Aromatic Compounds**	0.97%	0.89%	1.95%	1.76%
**Miscellaneous**	7.78%	8.11%	7.86%	7.82%
**Motility and Chemotaxis**	0.64%	0.59%	1.04%	0.94%
**Nitrogen Metabolism**	1.22%	1.14%	1.85%	1.73%
**Nucleosides and Nucleotides**	3.44%	3.59%	3.05%	3.16%
**Phages, Prophages, Transposable elements, Plasmids**	2.29%	1.86%	1.79%	1.73%
**Phosphorus Metabolism**	0.72%	0.76%	0.78%	0.79%
**Photosynthesis**	0.05%	0.04%	0.08%	0.08%
**Potassium metabolism**	0.34%	0.33%	0.41%	0.41%
**Protein Metabolism**	9.04%	8.99%	7.93%	8.22%
**Regulation and Cell signaling**	1.30%	1.32%	1.52%	1.51%
**Respiration**	2.36%	2.12%	3.04%	2.89%
**RNA Metabolism**	4.59%	4.46%	4.14%	4.24%
**Secondary Metabolism**	0.32%	0.36%	0.31%	0.32%
**Stress Response**	2.10%	2.03%	2.62%	2.55%
**Sulfur Metabolism**	1.14%	1.13%	1.00%	0.99%
**Virulence, Disease and Defense**	2.73%	2.56%	3.38%	3.20%

The samples were annotated using M5RNA database: C1754 and C1756: Upstream and downstream samples from November; C1755 and C1757: Upstream and downstream samples from March.

### Functional analyses of wastewater sequences

After quality-filtering the reads by the MG-RAST pipeline, 30% to 49% of the remaining reads were annotated to level 1 subsystems (S1 Table). Based on SEED, the 12 most abundant subsystems were distributed similarly among all samples. Making up 41% to 45% of all aligned reads, the most frequent subsystems were “Clustering-based subsystems”, “Carbohydrates”, “Protein metabolism” and “Amino acids and derivatives” (Table 1). Between 2.5% and 3.3% of all level 1 reads were annotated to the subsystem “Virulence, disease and defense” (VDD) and scrutinized in depth. VDD consisted of seven level 2 clusters with the predominance of “Resistance to antibiotics and toxic compounds” (RATC). In all samples, around 68% (± 4%) of the VDD reads were assigned to the RATC subsystem (Table 2). This level 2 cluster contained genes assigned to resistance and adaption to antibiotics, metals and other environmental influences. A high percentage of assigned sequences belonged to five functional groups. The first group was attributed to “Resistance to fluoroquinolones” including DNA gyrase subunit A (*gyrA)*, B (*gyrB)*, Topoisomerase IV subunit A (*parC*) and B (*parE)* (C1754: 22.61%; C1756: 24.78%; C1755:13.48%; C1757:14.97%). The second was “Multidrug resistance efflux pumps”, such as members of the multi antimicrobial extrusion protein family and resistance-nodulation-cell division superfamily (14.67%; 12.00%; 13.18%; 13.70%). The third was “Copper homeostasis” (14.24%; 14.14%; 18.11%; 16.90%). The fourth was “BlaR1 family regulatory sensor-transducer disambiguation” mainly represented by Cu^2+^-exporting ATPase and rarely by regulatory sensor-transducer of the *blaR1*/*mecR1 family* (12.00%; 12.81%; 12.26%; 11.67%) and the fifth was “cobalt-zinc-cadmium resistance” (10.07%; 7.85%; 18.29%; 16.51%) (Table 2).

**Table 2:** Relative abundance of VDD and RATC genes (≥1%) in the samples.

Subsystems	C1754	C1756	C1755	C1757
**Level 1 VDD-hits (%)**	2.73	2.56	3.38	3.20
**Level 2 RATC within VDD (%)**	68.22	64.98	71.29	70.33
**Level 3 Categories within RATC (%)** ^**a)**^				
**Resistance to fluoroquinolones**	22.61	24.78	13.48	14.97
**Multidrug Resistance Efflux Pumps**	14.67	12.00	13.18	13.70
**Copper homeostasis**	14.24	14.14	18.11	16.90
**BlaR1 Family Regulatory Sensor-transducer Disambiguation**	12.00	12.81	12.26	11.67
**Cobalt-zinc-cadmium resistance**	10.07	7.85	18.29	16.51
**Methicillin resistance in Staphylococci**	5.71	6.55	3.68	4.04
**Arsenic resistance**	3.56	3.29	2.89	3.17
**Multidrug efflux pump in Campylobacter jejuni (CmeABC operon)**	2.78	1.93	4.67	4.89
**Resistance to Vancomycin**	1.95	1.96	0.19	0.41
**Erythromycin resistance**	1.81	2.05	1.14	1.14
**Copper homeostasis: copper tolerance**	1.77	1.72	2.06	2.10
**Beta-lactamase**	1.46	1.76	1.91	1.98
**Cadmium resistance**	1.39	1.42	0.52	0.74
**Bile hydrolysis**	1.32	1.77	0.43	0.58
**The mdtABCD multidrug resistance cluster**	1.07	0.88	2.47	2.46
**Streptococcus pneumoniae Vancomycin Tolerance Locus**	0.82	1.42	0.19	0.20
**Mercury resistance operon**	0.97	1.27	1.89	1.73
**Mercuric reductase**	0.78	1.13	1.40	1.30

^a)^Legend: (RATC ≥1%).

### Abundance and ARG diversity

**Fig 2:**
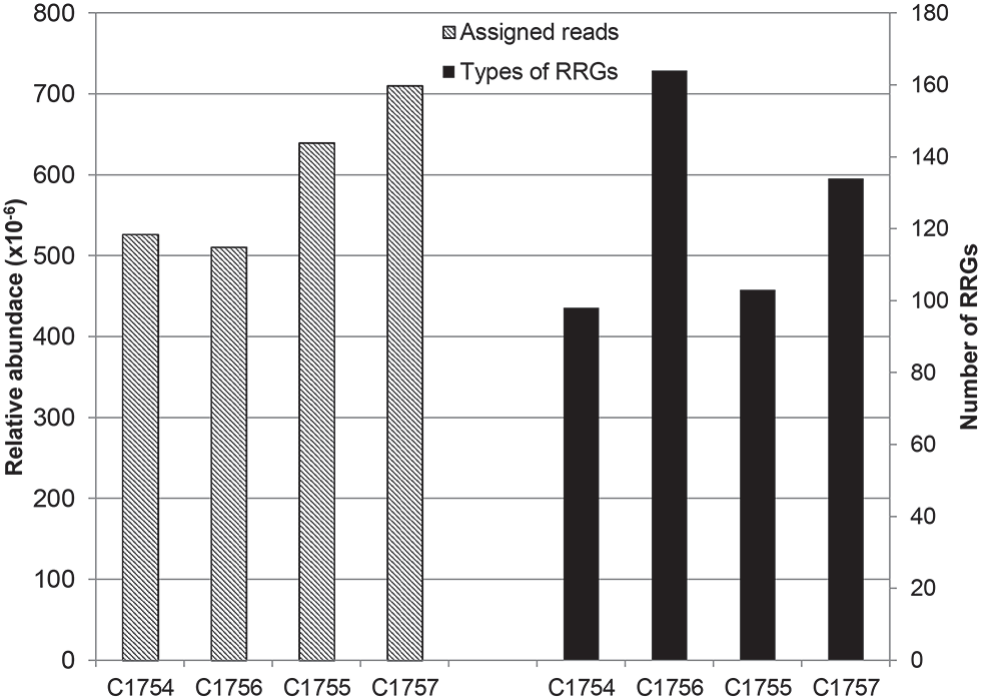
Abundance and diversity of annotated reads. Abundance (left) and diversity (right) of reads annotated against CARD are depicted. The hits were normalized against the total number of reads in each sample after QC-filtering.

After blasting by means of “BLASTX” against CARD, the best hits were normalized to the total number of quality filtered reads and later referred to as relative abundance. This abundance of reads was subsequently expressed in parts per million (ppm) according to Ma *et al*. [32]. Analyses of the four samples revealed reads of 526 ppm in sample C1754, 511 ppm in C1756, 639 ppm in C1755 and 710 ppm in C1757. Moreover, the genes were subsequently classified into resistance-related gene (RRG) clusters depending on their ARO numbers (Fig 2). In up- and downstream samples, we found 98, respectively 164 different RRGs corresponding to November samples, and 103, respectively 134 corresponding to March samples. Apparently, the inflows from nursing home sewage had no principal effect on RRG abundance in municipal wastewater. However, their diversity increased slightly and their composition changed. We classified the RRGs into groups with similar resistance spectrum (resistance against antimicrobial compounds from the same class). In all samples, *rpoB* genes prevailed, which, provided particular mutations have occurred, can confer resistance against antibiotics of the rifamycine group (Table 3).

**Table 3:** Distribution of RRG-groups in the samples.

RRG groups	C1754 [ppm]	C1756 [ppm]	C1755 [ppm]	C1757 [ppm]
**Rifamycine**	203.2	155.9	243.4	245.1
**Multidrug**	70.3	86.1	87.0	136.3
**Quinolone**	58.8	45.6	102.5	102.7
**Tetracycline**	94.2	123.5	37.4	58
**Macrolides**	43.7	42.6	64.8	56.7
**Aminocoumarins**	27.7	25.3	50.6	45.1
**Beta-Lactam**	11.6	14.4	24.7	29.4
**Aminoglycosides**	7.6	8.7	17.6	19.3
**Sulfonamides**	1.5	3.3	2.1	6.8
**Chloramphenicol**	2.0	1.3	4.2	4.1
**Vancomycin**	5.7	3.3	1.3	2.7
**Others**	0.6	0.7	4.2	3.8

The number of hits against the CARD in each RRG-group is presented as hits per million reads (ppm).

For instance, in the November samples, the relative abundance of reads related to the rifamycine group decreased after the inflow. Moreover, March samples showed a smaller variation of reads tagged to the RRG group of multidrug resistance.

Notably, tetracycline related reads such as *tetA*, *tetB(P)*, *tetC*, *tetD*, *tetG*. *tetL*, *tetK*, *tetM*, *tetO*, *tetQ*, *tetS*, *tetT*, *tetW*, *tetX*, *tet32*, *tet39*, *tet43* and *tet45* showed an increment of over 30% in both downstream samples. The other RRG-groups did not exhibit any remarkable changes.

**Fig 3:**
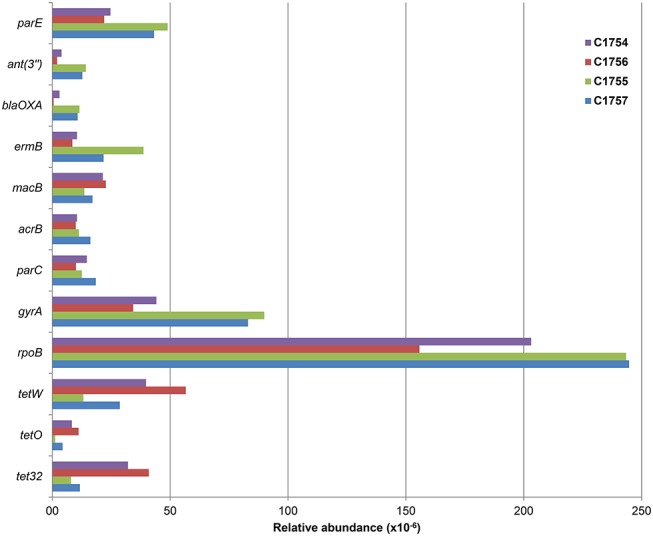
Abundance of the 12 most frequent ARGs types with the highest blast hits number (Relative abundance in any sample ≥10 ppm).

The resistance genes with the highest frequency in the samples are presented in Fig 3. The RNA polymerase encoding *rpoB* gene, which can confer resistance against rifamycin in the presence of few particular mutations, was detected in all samples with an abundance of over 150 ppm (S2 Table). The resistance genes *tetW* and *gyrA* had a relative abundance higher than 50 ppm (S2 Table) in at least one sample from November and March. The following relevant RRGs [33] had a relative abundance higher than 10 ppm: aminoglycoside adenyltransferases, macrolide resistance genes *ermB* and *mac*B, the multidrug gene *acr*B from the resistance-nodulation-division (RND) family. Furthermore, the quinolone resistance genes *par*C and both tetracycline genes *tet*O and *tet*32 were also detected with the same relative abundance. In the November samples, OXA gene family reads were present with a relative abundance lower than 10 ppm, in contrast to the March samples with a relative abundance higher than 10 ppm. This gene family belongs to β-lactam resistance gene ontologies among other detected determinants coding for β-lactamases (CTX-M, GES, AER CARB, PSE, R1, OMP, CphA, PC1, SHV, FEZ, TEM, AmpC and mecA) and was the one with the highest abundance in this group. Overall, sulfonamide resistance genes such as *fol*P, *sul*1, *sul*2 and vanocmycin resistance related genes such as *van*C, *van*R, *van*S, *van*T, *van*W *van*XYC, *van*Y and *van*Z were present below 5 ppm or at the detection limit (S2 Table).

Reads assembled into contigs from C1754, C1756, C1755 and C1757 were blasted against CARD database with a total of 13, 193, 29 and 80 aligned contigs, respectively, and resulted in different types and numbers of RRG (8, 71, 19 and 80) (S2 Table).

### Abundance of mobile genetic elements

**Fig 4:**
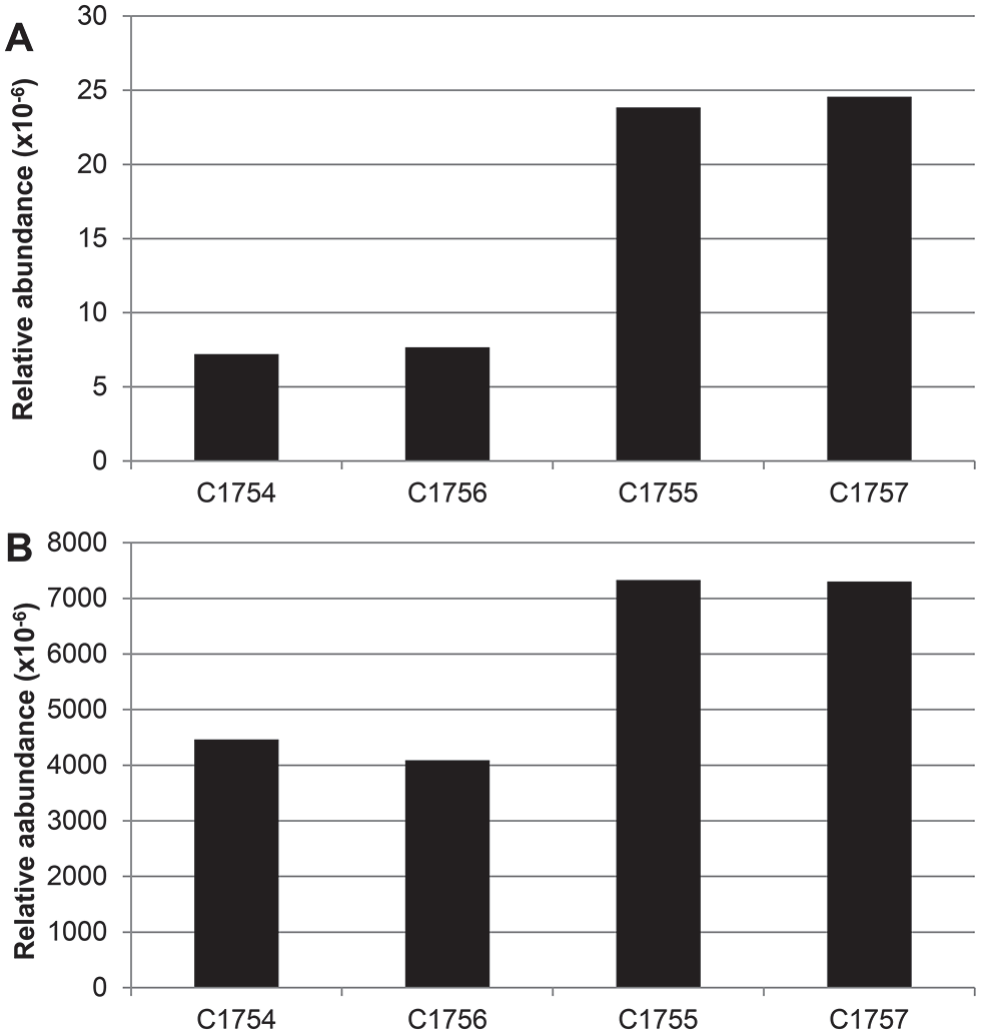
Relative abundance of integronase genes (A) and plasmids (B).

Reads of seven to approximately 25 ppm could be assigned to integrase coding sequences of the INTEGRALL database (Fig 4). The November samples (C1754 and C1756) and the March samples (C1755 and C1757) were mapped against known integrase encoding genes with reads of 7, 8, 24 and 25 ppm, respectively. About 74% to 85% of the reads were assigned to class 1 integrase gene *intI1*. The remaining reads were associated to *intI1delta*, *intI2*, *intI3*, *intIPac*, *groEL/intI1*, *intIA* and *intI (* S3 Table). Furthermore, assembled reads of all samples were simultaneously blasted against the integrall database. None of the 43,808 contigs from C1754 could be identified as encoding an integrase gene. Only 4 of 234,564 contigs (C1756), 4 of 23,420 contigs (C1755) and 7 of 74,589 (C1757) matched known integrase (S3 Table).

The hits were normalized against the total number of reads in each sample after QC-filtering.

Moreover, reads of 4460 and 4087 ppm from C1754 and C1756 respectively matched with plasmid sequences available in the NCBI RefSeq database. Of these, 31% were assigned to an unnamed plasmid of *Eubacterium eligens* ATCC 27750 (NC_012780.1). Interestingly, a higher number of reads from C1755 (7329 ppm) and C1757 (7303 ppm) were annotated (Fig 4), where 8.5% were assigned to the plasmid pKV29 of β-Proteobacterium *Delftia* sp. Strain KV29 (NC_019312.1). Notably, we did not find any dominating plasmids in either March samples (S4 Table).

Finally, after blasting of contigs against the plasmid database, we founded 210 contigs (C1754), 1440 contigs (C1756), 292 contigs (C1755) and 575 contigs (C1757) matching with 86, 366, 134 and 264 plasmids, respectively (S4 Table).

## Discussion

In this study on the potential introduction of ARG into the water cycle by medical facilities other than hospitals, we investigated differences between the microbial community in up- and downstream samples of wastewater from a nursing home collected in two different seasons. We used the M5RNA database available on MG-RAST, which allows examination of microbial composition in metagenomic wastewater samples. In accordance with a recent study, which assumed 100 bp sequences as too short for accurate identification at sub-phyla levels [34], we only analyzed the community at the phylum level, and not in greater detail at the level of order or even genus. Our results did not show any differences at domain level when comparing samples from both sampling sites in the same season. However, comparison of the bacteria phyla distribution showed a seasonal difference between samples. The phylum *Firmicutes* dominated in the November samples. Firmicutes has been reported as the dominant phylum in wastewater samples with a high level of pollution and extreme conditions [35]. *Firmicutes* are frequently found alongside the phyla *Bacteroidetes* in human and animal feces [36–39]. Some studies describe them as being one of the dominating phyla in raw wastewater, while others describe low abundance [35,39–42]. Beside these phyla, it has been shown that *Proteobacteria*, belong to the microbial community of the sewer system and, dominate in sewage samples [35,42,43], although only found to represent *c*. 0.1% of the species in the human colon [37,42]. The low abundance of *Proteobacteria* and high number of feces-associated bacterial groups found in the November wastewater samples may indicate a domination of bacteria of fecal origin over other environmental bacteria. In contrast, the March sewage samples showed a different phyla distribution, with a similar number of reads linked to *Firmicutes* and to *Proteobacteria* in C1755 and an approximately comparable distribution in C1757. Our results indicate the presence of fecal bacteria independent of sampling upstream or downstream from the nursing home, yet to a different extent on both sampling dates. No extreme environmental conditions in the municipal sewage system were reported or investigated in further tests (data not shown). Due to the limited number of samples and without the possibility of statistical power tests our taxonomic analyses did not reveal an apparent alteration of the municipal sewage system community by the medical facility.

MG-RAST was used for functional gene annotations, especially those related to resistance against antibiotics and other compounds. Around 30% to 49% of the reads, which passed the MG-RAST quality control filter, were assigned to genes from the SEED level 1. These values were markedly higher than those reported or calculated on Illumina reads from other studies [25,34]. Our results were in accordance with a previous study by Wang et al. [25], where the number of annotated reads was closely related depending on the sample origin and the bacterial composition.

Regarding level 1 subsystem distribution, a large number of reads was assigned to the subsystem with basic prokaryotic processes such as carbohydrate metabolism. However, the samples did not show obvious differences at this level. Neither did the subsystem “Virulence, Disease and Defense” (VDD), which includes the level 2 system “Resistance to antibiotics and toxic compounds” (RATC), show any differences between the up- and downstream samples. Furthermore, in all four samples, the major number of reads in the subsystem VDD was assigned to the RATC system. For instance, resistance to fluoroquinolones, which proved to be one of the highest portions of RATC-assigned reads, has already been reported as dominating in the sludge of wastewater treatment plant [25]. The others categories were multidrug resistance efflux pumps, *bla*R1 family regulatory sensor-transducer disambiguation, methicillin resistance in staphylococci, multidrug efflux pump in *Campylobacter jejuni* (CmeABC operon), resistance to vancomycin, erythromycin, beta-lactamase and the mdtABCD multidrug resistance cluster. Overall, the samples were classified in the same range without any noteworthy differences in the number of reads. In addition to ARG, the RATC system includes heavy metal resistances against cobalt, zinc, copper and others. Different authors have already reported the traits resistance to antibiotics and heavy metals to be linked, due to their close association to mobile genetic elements and a possible co-selection with ARGs [44–46]. Based on this, heavy metal resistance genes could be added to the ARG index for better description of the antibiotics resistance potential [47]. Therefore, we included the reads associated with metal resistance in our analyses. At least 1% of reads in the RATC subsystem were assigned to copper [48,49], cobalt [44], zinc [50], cadmium [51] and arsenic resistance [52]. Like ARGs no apparent differences were detected between samples collected at the same time.

We used the strict and unambiguous resistance ontology of the CARD database to annotate genes possibly involved in contamination of wastewater with ARG. Up- and downstream samples contained ARG without salient differences between one another. These data may indicate that the ARG load from the nursing home did not have a detectable influence on the municipal wastewater. Notably, this result is not comparable to a hospital analysis, where wastewater can carry 27 times the number of antibiotic resistant bacteria than urban wastewater [5]. A conceivable reason could be the high use of antimicrobials in hospitals, whereas in Germany the antibiotic use point prevalence (defined daily doses per 1,000 residents per day) is relatively low in nursing homes [53]. Although the increase in ARG found between the up- and downstream samples was not considerable, slight differences were by all means detected between both sampling sites. Moreover, a large number of reads were assigned to genes involved in rifamycine resistance without any notable differences between samples. Rifamycine resistance genes, i.e. RNA polymerase subunit B encoding genes, are ubiquitous in many environments, and are often detected at the highest level [32]. Furthermore, no obvious differences were detected in genes causing resistance against antibiotics such as β-lactams, macrolides or sulfonamides found in samples taken up- and downstream. However, on both sampling dates there was an increase of tetracycline resistance genes and sequences encoding multidrug resistance systems downstream from the nursing home [54]. Both types of resistance are of medical importance, however the increase detected in the nursing home’s inflow was considered too small to allow to draw a reliable conclusion on the base of the limited sample size.

There are many differences in the output of metagenome annotation when using the MG-RAST platform or blast against CARD. Using CARD, the relative abundance of genes associated with resistance against tetracycline showed a similar abundance as genes associated with the quinolone RGG. In contrast, the subcategory “Resistance to fluoroquinolones” showed the highest relative abundance of all antibiotic resistance subcategories in MG-RAST; however, no resistance to tetracycline was reported in this RATC subcategory. These differences are primarily due to the organization of the databases. The SEED database focuses on the annotation of core metabolism rather than on categories such as resistance groups. Furthermore, not all related genes are summarized in one comprehensive category [16]. For instance, tetracycline resistance genes like *tet*O, are neither included in RATC-Category nor in the Level1 subsystem “VDD” as expected; rather they are located in sub-categories of the level 1 subsystem “Protein Metabolism”. Basically, the differences between the results are a consequence of the differences in the structure and design of the databases. Again, the dissimilarities in the used cutoffs and possible gene coverages render the results incomparable. MG-RAST provides a good overview of all the genes present in the metagenome. Special databases like CARD are nonetheless required especially for deep ARG analysis.

Mobile genetic elements (MGE) like integrons or plasmids play an important role in the transfer and spread of genes between bacteria, especially those related to antibiotic resistance [55]. In this study, the abundance of MGE was investigated to describe the potential of gene mobility in the metagenome samples, and to control for a possible spread of these elements by nursing home residents into the sewage [3,56]. The integrase genes, which were selected as unambiguous markers for integrons, showed nearly the same abundance at the interconnected sampling sites, albeit marked differences on both sampling dates, which might be in line with the different microbial composition in both sampling seasons. Additionally, *IntI*1 was the dominating integrase gene in all samples, confirming its higher prevalence in wastewater-associated environments [25,31].

Like integrase genes, plasmids were present to a similar extent at the interconnected sample sites, although the extent to which they were present differed in the two sampling seasons and the nursing home inflow showed no influence on the abundance of plasmids. The annotated plasmids were classified according to their host. The resulting taxonomy results revealed a notable relation between dominating phyla, based on the analysis of SSU reads and plasmids with high frequency. Comparison of the outcome of this plasmid BLAST with the taxonomy results revealed a relation between dominating phylum and the most frequent plasmid host. An unnamed plasmid of *Eubacterium eligens* [57], a species belonging to the phylum *Firmicutes* [58], was found in more than 30% of the total BLAST hits in the November samples. This result is in accordance with the high prevalence of *Firmicutes* SSU-reads in these samples. Yet, we did not detect similarly prevalent phyla in the March samples.

Besides providing new insights and opportunities, microbial metagenome studies are challenging and confined in their reproducibility [59]. However, the similar distribution of the various genetic traits found at both interconnected sampling sites argues for the reproducibility of the approach used in this work. Indeed, wastewater changes continuously, for which reason we collected “24-hour composite samples” in different seasons to avoid a bias due to differences in the microbial load of the wastewater at different times of day or seasonal variations. Despite these precautions, neither comparison of up- or downstream samples, nor samples taken from different seasons revealed major differences in ARG. Another limitation here is the read length of 100 bases, which, as mentioned above, might not be sufficient to provide a reliable annotation beyond taxon phylum [23]. Furthermore, housekeeping genes like the RNA polymerase subunit B encoding gene (*rpoB*) [60] confer resistance against antibiotics of the rifamycine group only, if mutations in a few particular codons are present [61,62]. Consequently, reads were assigned to resistance genes irrespective of whether the sequence contained a corresponding mutation or not. Similar restrictions are encountered with genes assigned to resistance against aminocumarines [63] and quinolones [64], which differ in only a few mutations from their susceptible types. Also, multidrug efflux systems, like the Resistance-Nodulation-Division Family, are usually present in the bacterial genome, and only overexpression of this pump, which cannot be detected without expression experiments, confers resistance against different antibiotics [65].

In an attempt to improve the sequence length and results, reads were assembled in contigs. Only about 23% to 51% of the reads were assembled in contigs larger than 250 bp. This leads to a loss of more than 49% of the sequence data. Previous studies reported similar drawbacks in assembling environmental sequences and explained it with the high bacterial diversity in environmental samples [43,66] (S1 Table). Additionally, the annotations of unassembled reads and contigs were not comparable because the information about the frequencies of reads was lost after assembly [16].

This study was conducted at the instance of a 280-resident nursing home to investigate the impact of ARG load in wastewater from a long-term health care facility on municipal sewage of an 800-inhabitant village. The results demonstrate that the microbial composition of the wastewater did not differ before or after the inflow from the nursing home. However, the study revealed seasonal changes. Different approaches in annotation to ARG and MGE also revealed mainly seasonal differences and only minute sampling site differences. In contrast to sewage from hospitals, which is well known to introduce ARG into the water cycle, similar pollution by a long term health care facility was not detected. Further studies with a higher number of interconnected samples should be performed for statistical proof.

## Supporting Information

S1 Table Sequence quality control (QC) steps and the de novo assembling. Details about the sequence quality control (QC) steps and the *de novo* assembling by use of the CLC Genomic Workbench 6.5.1 (Number of reads).
(DOCX)

S2 Table Abundance and Identity of detected antibiotic resistance related genes. Matched high-throughput sequencing reads/contigs of the samples C1754, C1755, C1756 and C1757 against CARD.
(DOCX)

S3 Table Abundance and Identity of detected integrase sequences. Matched high-throughput sequencing reads/contigs of the samples C1754, C1755, C1756 and C1757 against the INTEGRALL database.
(DOCX)

S4 Table Abundance and Identity of detected plasmid sequences. Assigned high-throughput sequencing reads/contigs of the samples C1754, C1755, C1756 and C1757 against the RefSeq plasmid.
(DOCX)
