# Supplementary material for: Influence of a Non-Hospital Medical Care Facility on Antimicrobial Resistance in Wastewater
Source: PLoS One. 2015 Mar 30;10(3):e0122635. doi: 10.1371/journal.pone.0122635 (PMC4379178; doi:10.1371/journal.pone.0122635)
Supplement: S1 Table — Details about the sequence quality control (QC) steps and the de novo assembling by use of the CLC Genomic Workbench 6.5.1 (Number of reads). (DOCX) [file pone.0122635.s001.docx]

**Table S1.** Details about the sequence quality control (QC) steps and the *de novo* assembling by use of the CLC Genomic Workbench 6.5.1 (Number of reads).

|  | **Samples** | | | |
| --- | --- | --- | --- | --- |
|  | **C1754** | **C1756** | **C1755** | **C1757** |
|  |  |  |  |  |
| Sequencing (raw reads) | 5170002 | 31723714 | 5520370 | 11738166 |
|  |  |  |  |  |
| **MG-RAST** |  |  |  |  |
| After MG-QC pipeline [reads] | 4023711 | 26454707 | 4958391 | 10477959 |
| Ribosomal RNA [reads] | 82440 | 719321 | 89910 | 227586 |
| SEED Level 1 [reads] | 1237566 | 8924699 | 2432056 | 4664892 |
|  |  |  |  |  |
| **Blast quality control** |  |  |  |  |
| After FASTX-QC [reads] | 4577752 | 28139075 | 4782085 | 10258085 |
|  |  |  |  |  |
| **Assembling** |  |  |  |  |
| After QC [reads] | 5020675 | 31074051 | 5372132 | 11445801 |
| Matched [reads] | 1231502 | 15857899 | 1552371 | 3930866 |
| Not matched [reads] | 3789173 | 15216152 | 3819761 | 7514935 |
| Contigs | 43808 | 234564 | 23420 | 74589 |
| Average contig length (bp) | 555 | 722 | 877 | 737 |
