# Supplementary material for: Influence of a Non-Hospital Medical Care Facility on Antimicrobial Resistance in Wastewater
Source: PLoS One. 2015 Mar 30;10(3):e0122635. doi: 10.1371/journal.pone.0122635 (PMC4379178; doi:10.1371/journal.pone.0122635)
Supplement: S2 Table — Matched high-throughput sequencing reads/contigs of the samples C1754, C1755, C1756 and C1757 against CARD. (DOCX) [file pone.0122635.s002.docx]

**Table S2: Matched high-throughput sequencing reads/contigs of the samples C1754, C1755, C1756 and C1757 against CARD**

**Matched high-throughput sequencing reads of sample C1754 against CARD**

(Sorted by ARO-number)

| **ARO** | **Organism** | **Accession number** | **Gene** | **E value ≤** | **Identity (%) ≥** | **Hit length (aa) ≥** | **Number of reads** |
| --- | --- | --- | --- | --- | --- | --- | --- |
| 3000017 | Pseudomonas aeruginosa | AF350424.1.gene1.p01 | blaOXA-34 | 9.0E-13 | 96.97 | 33 | 1 |
| 3000017 | Pseudomonas aeruginosa | AJ854182.1.gene1.p01 | blaOXA-74 | 7.0E-12 | 100 | 33 | 1 |
| 3000017 | Salmonella enterica subsp. enterica serovar Bredeney | AM932669.1.gene2.p01 | blaOXA-129 | 3.0E-12 | 100 | 33 | 3 |
| 3000017 | Acinetobacter lwoffii | HQ122933.1.gene1.p01 | blaOXA-134a | 2.0E-10 | 90.91 | 33 | 5 |
| 3000017 | Riemerella anatipestifer | JF268688.1.gene5.p01 | blaOXA-209 | 3.0E-12 | 100 | 33 | 1 |
| 3000017 | Acinetobacter baumannii | JN215211.1.gene1.p01 | blaOXA-219 | 3.0E-11 | 93.94 | 33 | 1 |
| 3000017 | Pseudomonas aeruginosa | U37105.2.gene2.p01 | blaOXA-10 | 3.0E-10 | 93.75 | 32 | 1 |
| 3000017 | Pseudomonas aeruginosa | U59183.1.gene3.p01 | blaOXA-13 | 7.0E-13 | 93.94 | 33 | 1 |
| 3000019 | Shigella dysenteriae Sd197 | CP000034.1.gene4394.p01 | ampC | 6.0E-12 | 100 | 33 | 1 |
| 3000019 | Escherichia coli O157:H7 str. Sakai | NC_002695.1.914045.p01 | ampC | 5.0E-12 | 100 | 33 | 4 |
| 3000027 | Salmonella enterica subsp. enterica serovar Agona str. SL483 | CP001138.1.gene2905.p01 | emrA | 4.0E-11 | 96.97 | 33 | 1 |
| 3000027 | Escherichia coli O157:H7 str. Sakai | NC_002695.1.914737.p01 | ECs3547 | 3.0E-11 | 100 | 33 | 5 |
| 3000054 | Escherichia coli O157:H7 str. Sakai | NC_002695.1.914620.p01 | ECs0516 | 2.0E-10 | 100 | 32 | 1 |
| 3000071 | Enterococcus faecium | AF130997.1.orf1.gene.p01 | vanSD | 6.0E-10 | 96.88 | 32 | 1 |
| 3000071 | Clostridium difficile 630 | AM180355.1.gene1831.p01 | vanS | 1.0E-07 | 90 | 30 | 3 |
| 3000074 | Enterococcus faecalis V583 | AE016830.1.gene1357.p01 | EF_1370 | 1.0E-11 | 100 | 33 | 1 |
| 3000074 | Enterococcus faecalis V583 | AE016830.1.gene424.p01 | EF_0420 | 1.0E-09 | 90.91 | 31 | 2 |
| 3000074 | Klebsiella pneumoniae subsp. pneumoniae MGH 78578 | CP000647.1.gene3015.p01 | emrB | 6.0E-07 | 100 | 33 | 2 |
| 3000074 | Enterobacter cloacae subsp. cloacae ATCC 13047 | CP001918.1.gene4071.p01 | ECL_04023 | 2.0E-12 | 100 | 33 | 3 |
| 3000074 | Escherichia coli O157:H7 str. Sakai | NC_002695.1.914736.p01 | ECs3548 | 2.0E-10 | 100 | 33 | 5 |
| 3000074 | Acinetobacter baumannii AYE | NC_010410.6003262.p01 | emrB | 5.0E-06 | 90.91 | 27 | 8 |
| 3000077 | Enterococcus faecium | AF310956.2.orf2.gene.p01 | vanYB2 | 1.0E-13 | 100 | 33 | 1 |
| 3000077 | Enterococcus faecalis | U35369.1.gene3.p01 | vanYB | 3.0E-14 | 100 | 33 | 1 |
| 3000092 | Pseudomonas aeruginosa | Z22590.1.gene1.p01 | beta-lactamase | 2.0E-11 | 100 | 33 | 3 |
| 3000122 | Acinetobacter baumannii AB0057 | NC_011586.7045146.p01 | AB57_3104 | 3.0E-13 | 96.97 | 33 | 1 |
| 3000122 | Acinetobacter baumannii AB307-0294] | NC_011595.7057349.p01 | ABBFA_000784 | 1.0E-12 | 90.91 | 33 | 2 |
| 3000122 | Acinetobacter baumannii AB307-0294] | NC_011595.7057747.p01 | ABBFA_002299 | 3.0E-11 | 90.91 | 32 | 4 |
| 3000126 | Enterococcus faecalis | V01547.1.orf0.gene.p01 | V01547 | 4.0E-12 | 100 | 33 | 9 |
| 3000165 | Shigella sonnei | AF534183.gene.p01 | tetA | 7.0E-11 | 100 | 33 | 1 |
| 3000165 | Acinetobacter baumannii AYE | NC_010410.6002612.p01 | tetA | 5.0E-09 | 100 | 29 | 3 |
| 3000165 | Pseudomonas aeruginosa | X75761.gene.p01 | tetA | 2.0E-11 | 100 | 33 | 1 |
| 3000167 | Escherichia coli | Y19114.gene.p01 | tetC | 5.0E-09 | 93.94 | 32 | 2 |
| 3000168 | Salmonella enterica subsp. enterica serovar Agona str. SL483 | CP001138.1.gene4488.p01 | SeAg_B4524 | 3.0E-11 | 100 | 31 | 1 |
| 3000174 | Pseudomonas sp. | AF133139.gene.p01 | tetG | 2.0E-11 | 100 | 33 | 1 |
| 3000178 | Plasmid pNS1 | M16217.gene.p01 | tetK_2* | 4.0E-11 | 100 | 33 | 1 |
| 3000186 | Enterococcus faecalis | M85225.gene.p01 | tetM | 4.0E-12 | 100 | 33 | 1 |
| 3000186 | Staphylococcus aureus subsp. aureus Mu3 | NC_009782.5559075.p01 | tetM | 1.0E-11 | 96.97 | 33 | 1 |
| 3000186 | Enterococcus faecalis | X04388.gene.p01 | tetM | 4.0E-10 | 90.91 | 27 | 19 |
| 3000190 | Megasphaera elsdenii | AY485126.gene.p01 | tetOW | 3.0E-09 | 93.94 | 26 | 38 |
| 3000191 | Bacteroides fragilis | Z21523.gene.p01 | tetQ | 3.0E-10 | 93.94 | 33 | 12 |
| 3000192 | Listeria monocytogenes | L09756.gene.p01 | tetS* | 1.0E-06 | 96 | 25 | 1 |
| 3000194 | Butyrivibrio fibrisolvens | AJ222769.gene.p01 | tetW* | 1.0E-06 | 96.97 | 25 | 70 |
| 3000194 | Bifidobacterium longum subsp. longum F8 | DQ294299.gene.p01 | tetW_2 | 1.0E-06 | 90.91 | 25 | 112 |
| 3000195 | Clostridium perfringens | L20800.gene.p01 | tetBP | 3.0E-09 | 92.59 | 27 | 2 |
| 3000196 | Clostridiaceae bacterium K10 | AJ295238.gene.p01 | tet32 | 2.0E-07 | 90.62 | 25 | 147 |
| 3000205 | Bacteroides fragilis | M37699.gene1.p01 | tetX | 3.0E-11 | 100 | 33 | 1 |
| 3000207 | Escherichia coli O157:H7 str. Sakai] | NC_002695.1.912781.p01 | ECs1863 | 3.0E-11 | 100 | 33 | 1 |
| 3000210 | Mycobacterium tuberculosis CDC1551 | AE000516.2.gene708.p01 | rpoB | 6.0E-08 | 90 | 25 | 34 |
| 3000210 | Enterococcus faecalis V583 | AE016830.1.gene3155.p01 | rpoB | 9.0E-08 | 90 | 27 | 20 |
| 3000210 | Clostridium difficile 630 | AM180355.1.gene120.p01 | rpoB | 5.0E-06 | 90 | 25 | 94 |
| 3000210 | Shigella dysenteriae Sd197 | CP000034.1.gene3741.p01 | rpoB | 6.0E-11 | 100 | 33 | 2 |
| 3000210 | Klebsiella pneumoniae subsp. pneumoniae MGH 78578 | CP000647.1.gene4402.p01 | rpoB | 3.0E-09 | 100 | 31 | 6 |
| 3000210 | Legionella pneumophila str. Corby | CP000675.2.gene392.p01 | rpoB | 5.0E-09 | 90.32 | 31 | 18 |
| 3000210 | Clostridium botulinum A2 str. Kyoto | CP001581.1.gene3846.p01 | rpoB | 6.0E-06 | 90 | 25 | 208 |
| 3000210 | Enterobacter cloacae subsp. cloacae ATCC 13047 | CP001918.1.gene250.p01 | rpoB | 1.0E-11 | 100 | 33 | 2 |
| 3000210 | Bordetella pertussis CS | CP002695.1.gene18.p01 | rpoB | 4.0E-07 | 90.62 | 26 | 73 |
| 3000210 | Enterococcus faecium DO | CP003583.1.gene2745.p01 | rpoB | 2.0E-07 | 90 | 26 | 53 |
| 3000210 | Proteus mirabilis BB2000 | CP004022.1.gene2794.p01 | rpoB | 2.0E-10 | 90.91 | 33 | 3 |
| 3000210 | Pseudomonas aeruginosa PAO1 | NC_002516.2.881699.p01 | rpoB | 4.0E-08 | 90.91 | 27 | 10 |
| 3000210 | Escherichia coli O157:H7 str. Sakai | NC_002695.1.914942.p01 | rpoB | 9.0E-07 | 90 | 26 | 29 |
| 3000210 | Staphylococcus aureus subsp. aureus Mu50 | NC_002758.1120515.p01 | rpoB | 5.0E-08 | 96.43 | 28 | 1 |
| 3000210 | Staphylococcus aureus subsp. aureus COL | NC_002951.3236234.p01 | rpoB | 1.0E-08 | 93.55 | 31 | 1 |
| 3000210 | Staphylococcus aureus subsp. aureus MRSA252 | NC_002952.2860169.p01 | rpoB | 1.0E-13 | 90.91 | 33 | 1 |
| 3000210 | Azoarcus sp. BH72 | NC_008702.1.4609796.p01 | rpoB | 6.0E-08 | 90.32 | 25 | 33 |
| 3000210 | Acinetobacter baumannii ATCC 17978 | NC_009085.4918494.p01 | rpoB | 6.0E-08 | 90.62 | 28 | 14 |
| 3000210 | Staphylococcus aureus subsp. aureus JH9 | NC_009487.5169226.p01 | rpoB | 6.0E-10 | 93.94 | 33 | 2 |
| 3000210 | Staphylococcus aureus subsp. aureus USA300_TCH1516 | NC_010079.5776819.p01 | rpoB | 9.0E-11 | 90.62 | 32 | 2 |
| 3000210 | Acinetobacter baumannii SDF | NC_010400.5987325.p01 | rpoB | 9.0E-10 | 90.91 | 32 | 3 |
| 3000210 | Neisseria gonorrhoeae NCCP11945 | NC_011035.1.6448762.p01 | rpoB | 2.0E-06 | 90.32 | 25 | 42 |
| 3000210 | Acinetobacter baumannii AB307-0294 | NC_011595.7060572.p01 | rpoB | 4.0E-07 | 90 | 26 | 129 |
| 3000210 | Streptococcus pneumoniae Taiwan19F-14 | NC_012469.1.7686402.p01 | rpoB | 9.0E-08 | 90.32 | 26 | 49 |
| 3000210 | Staphylococcus aureus subsp. aureus ED98 | NC_013450.8613267.p01 | rpoB | 8.0E-07 | 90.32 | 25 | 101 |
| 3000216 | Shigella dysenteriae Sd197 | CP000034.1.gene457.p01 | acrB | 2.0E-10 | 100 | 33 | 2 |
| 3000216 | Klebsiella pneumoniae subsp. pneumoniae MGH 78578 | CP000647.1.gene443.p01 | acrB | 2.0E-07 | 96.15 | 26 | 1 |
| 3000216 | Enterobacter cloacae subsp. cloacae ATCC 13047 | CP001918.1.gene1257.p01 | acrB | 2.0E-11 | 100 | 33 | 1 |
| 3000216 | Bordetella pertussis CS | CP002695.1.gene992.p01 | acrB | 9.0E-09 | 90.91 | 33 | 1 |
| 3000216 | Escherichia coli O157:H7 str. Sakai] | NC_002695.1.912777.p01 | ECs1864 | 2.0E-06 | 92.86 | 25 | 5 |
| 3000216 | Escherichia coli O157:H7 str. Sakai | NC_002695.1.914619.p01 | ECs0515 | 1.0E-08 | 90.91 | 30 | 6 |
| 3000216 | Acinetobacter baumannii AB307-0294] | NC_011595.7058581.p01 | ABBFA_000732 | 6.0E-07 | 90.62 | 26 | 32 |
| 3000225 | Campylobacter fetus subsp. fetus | FN594949.1.orf3.gene.p01 | ant(6)-Ib | 3.0E-12 | 100 | 33 | 2 |
| 3000225 | Staphylococcus epidermidis RP62A | NC_006663.1.orf0.gene.p01 | aadE | 2.0E-13 | 100 | 33 | 1 |
| 3000226 | Escherichia coli O157:H7 str. Sakai | NC_002695.1.916103.p01 | folP | 2.0E-07 | 92.59 | 27 | 1 |
| 3000226 | Acinetobacter baumannii AB0057 | NC_011586.7045179.p01 | folP | 4.0E-08 | 100 | 25 | 1 |
| 3000232 | Plasmid NR79 | AF047479.2.orf1.gene.p01 | aadA3 | 6.0E-11 | 90.91 | 33 | 1 |
| 3000232 | Salmonella enterica | AJ628353.gene.p01 | aadA1b | 4.0E-13 | 93.94 | 33 | 2 |
| 3000232 | uncultured bacterium | AY139598.1.gene2.p01 | aadA5 | 7.0E-13 | 100 | 33 | 1 |
| 3000232 | Salmonella enterica subsp. enterica serovar Stanley | EU118119.1.orf1.gene.p01 | aadA2 | 1.0E-09 | 90.32 | 31 | 2 |
| 3000232 | Acinetobacter baumannii AYE | NC_010410.6003170.p01 | aadA1 | 4.0E-12 | 100 | 33 | 4 |
| 3000232 | Escherichia coli 1520 | NC_010558.1.6275994.p01 | aadA4 | 2.0E-11 | 100 | 33 | 2 |
| 3000232 | Pseudomonas aeruginosa | U37105.2.gene4.p01 | aadA10 | 1.0E-12 | 100 | 33 | 2 |
| 3000232 | Pseudomonas aeruginosa | Y18050.2.gene6.p01 | aadA1 | 5.0E-09 | 100 | 30 | 4 |
| 3000237 | Shigella dysenteriae Sd197 | CP000034.1.gene3205.p01 | tolC | 1.0E-11 | 100 | 33 | 1 |
| 3000237 | Klebsiella pneumoniae subsp. pneumoniae MGH 78578 | CP000647.1.gene3449.p01 | tolC | 2.0E-11 | 100 | 33 | 2 |
| 3000237 | Escherichia coli O157:H7 str. Sakai | NC_002695.1.916248.p01 | tolC | 2.0E-08 | 96.97 | 26 | 11 |
| 3000252 | Enterobacter aerogenes | AF336096.1.gene1.p01 | omp36 | 1.0E-10 | 100 | 30 | 2 |
| 3000252 | Enterobacter aerogenes | AF336097.1.gene1.p01 | omp36 | 9.0E-11 | 90.91 | 33 | 1 |
| 3000275 | Enterococcus faecium | AF110130.1.orf0.gene.p01 | linB | 2.0E-12 | 100 | 33 | 1 |
| 3000309 | Klebsiella pneumoniae subsp. pneumoniae MGH 78578 | CP000647.1.gene4115.p01 | emrD | 5.0E-07 | 90.62 | 32 | 2 |
| 3000309 | Escherichia coli O157:H7 str. Sakai | NC_002695.1.915420.p01 | emrD | 5.0E-08 | 96.15 | 26 | 2 |
| 3000319 | Staphylococcus aureus | AF167161.gene.p01 | mphC | 2.0E-13 | 100 | 33 | 1 |
| 3000368 | Enterococcus casseliflavus | L29638.gene.p01 | vanC2 | 4.0E-13 | 96.97 | 33 | 1 |
| 3000373 | Escherichia coli O157:H7 str. Sakai | NC_002695.1.915653.p01 | ECs3247 | 4.0E-11 | 100 | 33 | 4 |
| 3000375 | Clostridium difficile 630 | AM180355.1.gene2259.p01 | ermB | 3.0E-08 | 96.97 | 27 | 16 |
| 3000375 | Streptococcus pneumoniae | AM903082.gene3.p01 | ermB | 1.0E-06 | 93.94 | 25 | 32 |
| 3000378 | Pseudomonas aeruginosa PAO1 | NC_002516.2.877852.p01 | mexB | 7.0E-08 | 90.32 | 31 | 1 |
| 3000378 | Acinetobacter baumannii AB307-0294] | NC_011595.7057907.p01 | ABBFA_000816 | 4.0E-06 | 90.91 | 26 | 11 |
| 3000379 | Acinetobacter baumannii AB307-0294] | NC_011595.7058890.p01 | ABBFA_003020 | 5.0E-08 | 90.91 | 33 | 1 |
| 3000412 | Escherichia coli | DQ464881.1.gene2.p01 | sul2 | 2.0E-11 | 100 | 33 | 5 |
| 3000457 | Enterococcus faecalis V583 | AE016830.1.gene1599.p01 | parE | 2.0E-09 | 90 | 29 | 3 |
| 3000457 | Shigella dysenteriae Sd197 | CP000034.1.gene3210.p01 | parE | 3.0E-11 | 96.97 | 33 | 4 |
| 3000457 | Klebsiella pneumoniae subsp. pneumoniae MGH 78578 | CP000647.1.gene3444.p01 | parE | 5.0E-12 | 100 | 33 | 1 |
| 3000457 | Legionella pneumophila str. Corby | CP000675.2.gene802.p01 | parE | 3.0E-08 | 90 | 30 | 1 |
| 3000457 | Salmonella enterica subsp. enterica serovar Agona str. SL483 | CP001138.1.gene3336.p01 | parE | 6.0E-12 | 96.97 | 33 | 6 |
| 3000457 | Bordetella pertussis CS | CP002695.1.gene1273.p01 | parE | 7.0E-10 | 90.91 | 33 | 7 |
| 3000457 | Enterococcus faecium DO | CP003583.1.gene1173.p01 | parE | 2.0E-09 | 90 | 29 | 25 |
| 3000457 | Staphylococcus aureus subsp. aureus MRSA252 | NC_002952.2859942.p01 | grlB | 4.0E-11 | 90.91 | 33 | 1 |
| 3000457 | Staphylococcus aureus RF122 | NC_007622.3794232.p01 | grlB | 9.0E-09 | 90 | 30 | 5 |
| 3000457 | Azoarcus sp. BH72 | NC_008702.1.4609137.p01 | parE | 1.0E-10 | 93.94 | 33 | 1 |
| 3000457 | Acinetobacter baumannii SDF | NC_010400.5986295.p01 | parE | 6.0E-11 | 90.91 | 33 | 1 |
| 3000457 | Acinetobacter baumannii ACICU | NC_010611.6235284.p01 | parE | 7.0E-11 | 90.91 | 33 | 1 |
| 3000457 | Acinetobacter baumannii AB307-0294 | NC_011595.7060308.p01 | parE | 2.0E-07 | 90.91 | 25 | 36 |
| 3000457 | Streptococcus pneumoniae Taiwan19F-14 | NC_012469.1.7686068.p01 | parE | 4.0E-08 | 90.91 | 27 | 8 |
| 3000457 | Staphylococcus aureus subsp. aureus ED98 | NC_013450.8614085.p01 | parE | 6.0E-09 | 90.32 | 31 | 13 |
| 3000479 | Streptomyces caeruleus | AF205854.1.orf0.gene.p01 | gyrB-Rn | 1.0E-09 | 90.91 | 33 | 3 |
| 3000479 | Streptomyces roseochromogenes subsp. oscitans | AY136281.1.orf1.gene.p01 | gyrBR | 3.0E-09 | 90.62 | 30 | 3 |
| 3000480 | Streptomyces rishiriensis | AF205853.1.orf2.gene.p01 | parYR | 5.0E-09 | 90.91 | 33 | 2 |
| 3000480 | Streptomyces roseochromogenes subsp. oscitans | AY136281.1.orf0.gene.p01 | parYR | 5.0E-11 | 90.91 | 32 | 6 |
| 3000489 | Staphylococcus aureus subsp. aureus Mu50 | NC_002758.1121879.p01 | SAV1866 | 6.0E-10 | 90.62 | 32 | 1 |
| 3000491 | Salmonella enterica subsp. enterica serovar Agona str. SL483 | CP001138.1.gene2601.p01 | acrD | 1.0E-10 | 90.91 | 33 | 1 |
| 3000491 | Escherichia coli O157:H7 str. Sakai | NC_002695.1.915267.p01 | ECs3332 | 7.0E-10 | 96.97 | 28 | 9 |
| 3000493 | Shigella dysenteriae Sd197 | CP000034.1.gene2198.p01 | asmA | 3.0E-11 | 100 | 33 | 7 |
| 3000493 | Shigella dysenteriae Sd197 | CP000034.1.gene3671.p01 | ompR | 1.0E-09 | 90.32 | 27 | 7 |
| 3000493 | Shigella dysenteriae Sd197 | CP000034.1.gene3672.p01 | envZ | 7.0E-07 | 100 | 26 | 3 |
| 3000493 | Escherichia coli | GQ465831.1.gene2.p01 | ompF | 4.0E-11 | 100 | 33 | 4 |
| 3000498 | Bacteroides fragilis | M14730.gene.p01 | ermF* | 1.0E-12 | 100 | 33 | 1 |
| 3000499 | Escherichia coli O157:H7 str. Sakai | NC_002695.1.916015.p01 | ECs4137 | 2.0E-12 | 100 | 33 | 2 |
| 3000502 | Klebsiella pneumoniae subsp. pneumoniae MGH 78578 | CP000647.1.gene3710.p01 | acrF | 7.0E-06 | 90.62 | 25 | 7 |
| 3000508 | Escherichia coli O157:H7 str. Sakai | NC_002695.1.915747.p01 | ECs4396 | 4.0E-12 | 96.97 | 33 | 2 |
| 3000516 | Shigella dysenteriae Sd197 | CP000034.1.gene2879.p01 | emrR | 3.0E-07 | 96.97 | 25 | 8 |
| 3000516 | Klebsiella pneumoniae subsp. pneumoniae MGH 78578 | CP000647.1.gene3013.p01 | emrR | 6.0E-13 | 100 | 33 | 1 |
| 3000516 | Acinetobacter baumannii AB307-0294] | NC_011595.7058564.p01 | ABBFA_001502 | 2.0E-10 | 90.91 | 33 | 1 |
| 3000518 | Shigella dysenteriae Sd197 | CP000034.1.gene3519.p01 | crp | 2.0E-10 | 100 | 33 | 1 |
| 3000518 | Klebsiella pneumoniae subsp. pneumoniae MGH 78578 | CP000647.1.gene3780.p01 | crp | 7.0E-13 | 100 | 33 | 2 |
| 3000518 | Proteus mirabilis BB2000 | CP004022.1.gene2827.p01 | crp | 7.0E-08 | 100 | 27 | 2 |
| 3000522 | Lysinibacillus sphaericus | M15332.gene.p01 | erm(G)**_ermG* | 3.0E-10 | 96.97 | 33 | 3 |
| 3000533 | Escherichia coli O157:H7 str. Sakai | NC_002695.1.917702.p01 | ECs0964 | 3.0E-08 | 96.97 | 25 | 6 |
| 3000533 | Acinetobacter baumannii AB307-0294] | NC_011595.7058613.p01 | ABBFA_003018 | 7.0E-09 | 90 | 29 | 19 |
| 3000535 | Klebsiella pneumoniae subsp. pneumoniae MGH 78578 | CP000647.1.gene912.p01 | macB | 1.0E-08 | 90.32 | 31 | 1 |
| 3000535 | Clostridium botulinum A2 str. Kyoto | CP001581.1.gene598.p01 | CLM_0622 | 3.0E-07 | 90 | 26 | 20 |
| 3000535 | Clostridium botulinum A2 str. Kyoto | CP001581.1.gene798.p01 | CLM_0827 | 2.0E-06 | 90.32 | 26 | 25 |
| 3000535 | Proteus mirabilis BB2000 | CP004022.1.gene758.p01 | macB | 8.0E-11 | 90.91 | 33 | 1 |
| 3000535 | Listeria monocytogenes | HE999704.1.gene196.p01 | BN418_0205 | 6.0E-06 | 90 | 25 | 6 |
| 3000535 | Azoarcus sp. BH72 | NC_008702.1.4606598.p01 | azo0834 | 7.0E-10 | 90.91 | 33 | 1 |
| 3000535 | Azoarcus sp. BH72 | NC_008702.1.4609454.p01 | macB | 8.0E-06 | 92 | 25 | 3 |
| 3000535 | Acinetobacter baumannii SDF | NC_010400.5985985.p01 | macB | 8.0E-11 | 90.91 | 33 | 2 |
| 3000535 | Acinetobacter baumannii AB307-0294] | NC_011595.7060505.p01 | rpoB | 2.0E-06 | 90.62 | 26 | 20 |
| 3000535 | Streptococcus pneumoniae Taiwan19F-14 | NC_012469.1.7685735.p01 | SPT_1593 | 4.0E-09 | 90.32 | 30 | 18 |
| 3000535 | Streptococcus pneumoniae Taiwan19F-14 | NC_012469.1.7686878.p01 | SPT_1414 | 2.0E-09 | 90.62 | 32 | 1 |
| 3000556 | Campylobacter fetus subsp. fetus | FN594949.1.gene24.p01 | tet44 | 2.0E-09 | 90.62 | 32 | 8 |
| 3000559 | Shigella dysenteriae Sd197 | CP000034.1.gene1340.p01 | btuR | 2.0E-13 | 100 | 33 | 1 |
| 3000566 | Acinetobacter sp. LUH5605 | AY743590.gene.p01 | tet39 | 8.0E-09 | 96.97 | 29 | 9 |
| 3000574 | Enterococcus faecium | AF130997.1.orf0.gene.p01 | vanRD | 4.0E-10 | 90.91 | 33 | 2 |
| 3000574 | Enterococcus gallinarum | AF162694.1.orf4.gene.p01 | vanRc | 5.0E-10 | 90.62 | 32 | 2 |
| 3000574 | Clostridium difficile 630 | AM180355.1.gene1830.p01 | vanR | 4.0E-10 | 90.62 | 32 | 11 |
| 3000574 | Enterococcus faecalis | DQ212986.1.gene4.p01 | vanRG | 4.0E-09 | 90.32 | 31 | 3 |
| 3000574 | Enterococcus faecalis | NC_014475.1.orf0.gene.p01 | vanR | 1.0E-11 | 93.94 | 33 | 1 |
| 3000616 | Streptococcus pneumoniae Taiwan19F-14 | NC_012469.1.7685970.p01 | SPT_1925 | 3.0E-07 | 90.91 | 29 | 14 |
| 3000618 | Staphylococcus epidermidis ATCC 12228 | AE015929.1.gene5.p01 | SE_0005 | 1.0E-06 | 90.32 | 26 | 9 |
| 3000618 | Enterococcus faecalis V583 | AE016830.1.gene6.p01 | gyrA | 7.0E-08 | 90 | 26 | 21 |
| 3000618 | Clostridium difficile 630 | AM180355.1.gene6.p01 | gyrA | 9.0E-08 | 90.62 | 28 | 13 |
| 3000618 | Yersinia pestis Antiqua | CP000308.1.gene965.p01 | YPA_0930 | 6.0E-06 | 90.32 | 25 | 9 |
| 3000618 | Klebsiella pneumoniae subsp. pneumoniae MGH 78578 | CP000647.1.gene2640.p01 | gyrA | 2.0E-09 | 90 | 30 | 2 |
| 3000618 | Legionella pneumophila str. Corby | CP000675.2.gene1514.p01 | gyrA | 2.0E-09 | 90.32 | 28 | 7 |
| 3000618 | Vibrio cholerae MJ-1236 | CP001485.1.gene2164.p01 | VCD_003093 | 4.0E-12 | 96.97 | 33 | 1 |
| 3000618 | Clostridium botulinum A2 str. Kyoto | CP001581.1.gene7.p01 | gyrA | 9.0E-07 | 90.32 | 26 | 19 |
| 3000618 | Enterobacter cloacae subsp. cloacae ATCC 13047 | CP001918.1.gene3562.p01 | ECL_03523 | 1.0E-12 | 100 | 33 | 1 |
| 3000618 | Helicobacter pylori Gambia94/24 | CP002332.1.gene704.p01 | HPGAM_03615 | 9.0E-08 | 96 | 25 | 1 |
| 3000618 | Bordetella pertussis CS | CP002695.1.gene952.p01 | gyrA | 8.0E-07 | 90.32 | 27 | 17 |
| 3000618 | Proteus mirabilis BB2000 | CP004022.1.gene1837.p01 | gyrA | 2.0E-09 | 90.62 | 32 | 3 |
| 3000618 | Haemophilus influenzae 10810 | FQ312006.1.gene1417.p01 | HIB_14190 | 2.0E-06 | 90.91 | 25 | 2 |
| 3000618 | Listeria monocytogenes | HE999704.1.gene7.p01 | BN418_0007 | 3.0E-08 | 90 | 28 | 6 |
| 3000618 | Pseudomonas aeruginosa PAO1 | NC_002516.2.882800.p01 | gyrA | 2.0E-08 | 90.91 | 29 | 3 |
| 3000618 | Escherichia coli O157:H7 str. Sakai | NC_002695.1.916822.p01 | ECs3114 | 2.0E-06 | 92.31 | 25 | 10 |
| 3000618 | Staphylococcus aureus RF122 | NC_007622.3794239.p01 | gyrA | 5.0E-10 | 93.55 | 31 | 1 |
| 3000618 | Azoarcus sp. BH72 | NC_008702.1.4606680.p01 | gyrA | 8.0E-07 | 93.94 | 25 | 13 |
| 3000618 | Acinetobacter baumannii SDF | NC_010400.5986734.p01 | gyrA | 7.0E-10 | 90.91 | 32 | 6 |
| 3000618 | Acinetobacter baumannii AYE | NC_010410.6003186.p01 | gyrA | 1.0E-10 | 96.97 | 33 | 1 |
| 3000618 | Neisseria gonorrhoeae NCCP11945 | NC_011035.1.6447337.p01 | NGK_1285 | 3.0E-10 | 90.62 | 32 | 4 |
| 3000618 | Acinetobacter baumannii AB307-0294] | NC_011595.7058445.p01 | gyrA | 2.0E-07 | 90.32 | 29 | 41 |
| 3000618 | Streptococcus pneumoniae Taiwan19F-14 | NC_012469.1.7686721.p01 | gyrA | 3.0E-10 | 90.62 | 31 | 11 |
| 3000618 | Staphylococcus aureus subsp. aureus ED98 | NC_013450.8612775.p01 | gyrA | 1.0E-10 | 90 | 30 | 1 |
| 3000619 | Enterococcus faecalis V583 | AE016830.1.gene1598.p01 | parC | 6.0E-12 | 90.91 | 33 | 2 |
| 3000619 | Shigella dysenteriae Sd197 | CP000034.1.gene3218.p01 | parC | 9.0E-12 | 90.91 | 33 | 2 |
| 3000619 | Klebsiella pneumoniae subsp. pneumoniae MGH 78578 | CP000647.1.gene3437.p01 | parC | 2.0E-11 | 96.97 | 33 | 2 |
| 3000619 | Legionella pneumophila str. Corby | CP000675.2.gene3231.p01 | parC | 3.0E-07 | 92 | 25 | 1 |
| 3000619 | Clostridium botulinum A2 str. Kyoto | CP001581.1.gene3048.p01 | CLM_3137 | 1.0E-10 | 90.62 | 32 | 1 |
| 3000619 | Bordetella pertussis CS | CP002695.1.gene1275.p01 | parC | 6.0E-11 | 90.91 | 33 | 1 |
| 3000619 | Enterococcus faecium DO | CP003583.1.gene1174.p01 | parC | 8.0E-12 | 96.97 | 33 | 1 |
| 3000619 | Escherichia coli str. K-12 substr. W3110 | M58408.gene.p01 | parC | 2.0E-10 | 90.91 | 31 | 16 |
| 3000619 | Pseudomonas aeruginosa PAO1 | NC_002516.2.879741.p01 | parC | 6.0E-10 | 90.32 | 31 | 2 |
| 3000619 | Acinetobacter baumannii ACICU | NC_010611.6237080.p01 | parC | 3.0E-11 | 100 | 31 | 1 |
| 3000619 | Acinetobacter baumannii AB0057 | NC_011586.7046300.p01 | parC | 7.0E-10 | 90.91 | 31 | 4 |
| 3000619 | Acinetobacter baumannii AB307-0294 | NC_011595.7059884.p01 | parC | 2.0E-06 | 90.62 | 25 | 30 |
| 3000619 | Streptococcus pneumoniae Taiwan19F-14 | NC_012469.1.7685406.p01 | parC | 6.0E-07 | 90.91 | 28 | 4 |
| 3000621 | Azoarcus sp. BH72 | NC_008702.1.4608898.p01 | azo0443 | 2.0E-08 | 92.59 | 27 | 1 |
| 3000621 | Staphylococcus aureus subsp. aureus USA300_TCH1516 | NC_010079.5776919.p01 | USA300HOU_0022 | 2.0E-12 | 96.97 | 33 | 1 |
| 3000621 | Acinetobacter baumannii AB0057 | NC_011586.7045516.p01 | AB57_0437 | 5.0E-10 | 93.94 | 31 | 3 |
| 3000621 | Acinetobacter baumannii AB0057 | NC_011586.7045804.p01 | AB57_2380 | 4.0E-11 | 96.97 | 28 | 2 |
| 3000656 | Escherichia coli O157:H7 str. Sakai | NC_002695.1.916016.p01 | ECs4136 | 1.0E-08 | 96.97 | 27 | 9 |
| 3000662 | Escherichia coli O157:H7 str. Sakai | NC_002695.1.912474.p01 | ECs1443 | 3.0E-12 | 96.97 | 33 | 2 |
| 3000676 | Salmonella enterica subsp. enterica serovar Agona str. SL483 | CP001138.1.gene1383.p01 | hns | 1.0E-10 | 90.91 | 33 | 1 |
| 3000676 | Acinetobacter baumannii AB0057 | NC_011586.7046013.p01 | AB57_0355 | 1.0E-08 | 90.91 | 33 | 2 |
| 3000702 | Shigella dysenteriae Sd197 | CP000034.1.gene455.p01 | acrR | 2.0E-12 | 100 | 33 | 1 |
| 3000718 | Shigella dysenteriae Sd197 | CP000034.1.gene1597.p01 | marR | 7.0E-12 | 100 | 33 | 2 |
| 3000718 | Acinetobacter baumannii ATCC 19606 | NC_006877.3293011.p01 | marR | 2.0E-12 | 96.97 | 33 | 2 |
| 3000753 | Acinetobacter baumannii AB0057 | NC_011586.7045550.p01 | abeM | 1.0E-09 | 90.91 | 30 | 5 |
| 3000775 | Acinetobacter baumannii ATCC 17978 | NC_009085.4919117.p01 | A1S_1750 | 5.0E-08 | 90 | 30 | 1 |
| 3000776 | Acinetobacter baumannii SDF | NC_010400.5984384.p01 | ABSDF0736 | 8.0E-13 | 90.91 | 33 | 1 |
| 3000777 | Acinetobacter baumannii SDF | NC_010400.5984910.p01 | ABSDF1463 | 8.0E-12 | 96.97 | 33 | 1 |
| 3000778 | Acinetobacter baumannii SDF | NC_010400.5984909.p01 | ABSDF1462 | 4.0E-09 | 90.62 | 30 | 2 |
| 3000780 | Acinetobacter baumannii ATCC 17978 | NC_009085.4918693.p01 | A1S_2735 | 6.0E-11 | 93.94 | 33 | 2 |
| 3000780 | Acinetobacter baumannii AB0057 | NC_011586.7045443.p01 | adeI | 8.0E-07 | 90.91 | 26 | 4 |
| 3000782 | Acinetobacter baumannii ATCC 17978 | NC_009085.4918695.p01 | A1S_2737 | 2.0E-11 | 96.88 | 32 | 1 |
| 3000792 | Enterobacter cloacae subsp. cloacae ATCC 13047 | CP001918.1.gene3439.p01 | ECL_03401 | 5.0E-11 | 100 | 33 | 1 |
| 3000792 | Escherichia coli O157:H7 str. Sakai | NC_002695.1.916584.p01 | ECs2882 | 4.0E-06 | 100 | 25 | 6 |
| 3000793 | Salmonella enterica subsp. enterica serovar Agona str. SL483 | CP001138.1.gene2235.p01 | SeAg_B2257 | 8.0E-07 | 100 | 25 | 1 |
| 3000793 | Escherichia coli O157:H7 str. Sakai | NC_002695.1.916585.p01 | ECs2883 | 3.0E-09 | 90.91 | 31 | 10 |
| 3000794 | Salmonella enterica subsp. enterica serovar Agona str. SL483 | CP001138.1.gene2236.p01 | mdtC | 9.0E-08 | 90.32 | 31 | 2 |
| 3000794 | Enterobacter cloacae subsp. cloacae ATCC 13047 | CP001918.1.gene3441.p01 | ECL_03403 | 1.0E-06 | 90.91 | 25 | 5 |
| 3000794 | Proteus mirabilis BB2000 | CP004022.1.gene1674.p01 | mdtC | 3.0E-09 | 90.91 | 33 | 1 |
| 3000794 | Escherichia coli O157:H7 str. Sakai | NC_002695.1.916586.p01 | ECs2884 | 5.0E-07 | 93.94 | 25 | 7 |
| 3000795 | Escherichia coli O157:H7 str. Sakai | NC_002695.1.915750.p01 | ECs4393 | 8.0E-11 | 96.97 | 33 | 7 |
| 3000795 | Escherichia coli O157:H7 str. Sakai | NC_002695.1.916587.p01 | ECs2885 | 9.0E-09 | 96.97 | 28 | 9 |
| 3000800 | Pseudomonas aeruginosa PAO1 | NC_002516.2.881078.p01 | mexC | 4.0E-09 | 96.77 | 31 | 1 |
| 3000801 | Pseudomonas aeruginosa | U57969.gene.p01 | mexD | 3.0E-09 | 90.91 | 33 | 1 |
| 3000804 | Pseudomonas aeruginosa PAO1 | NC_002516.2.882884.p01 | mexF | 5.0E-10 | 90.91 | 33 | 1 |
| 3000814 | Acinetobacter baumannii AB307-0294] | NC_011595.7059912.p01 | ABBFA_002603 | 2.0E-07 | 90.91 | 26 | 20 |
| 3000826 | Klebsiella pneumoniae subsp. pneumoniae MGH 78578 | CP000647.1.gene2414.p01 | sdiA | 2.0E-14 | 100 | 33 | 1 |
| 3000828 | Acinetobacter baumannii SDF | NC_010400.5986590.p01 | baeR | 2.0E-11 | 93.94 | 33 | 1 |
| 3000828 | Acinetobacter baumannii AB307-0294] | NC_011595.7057856.p01 | ABBFA_000579 | 3.0E-10 | 93.94 | 29 | 3 |
| 3000829 | Klebsiella pneumoniae subsp. pneumoniae MGH 78578 | CP000647.1.gene2531.p01 | baeR | 1.0E-07 | 92.31 | 26 | 2 |
| 3000829 | Enterobacter cloacae subsp. cloacae ATCC 13047 | CP001918.1.gene3443.p01 | ECL_03405 | 9.0E-14 | 96.97 | 33 | 1 |
| 3000829 | Escherichia coli O157:H7 str. Sakai | NC_002695.1.916588.p01 | ECs2886 | 2.0E-11 | 96.97 | 33 | 2 |
| 3000829 | Acinetobacter baumannii SDF | NC_010400.5984250.p01 | baeS | 5.0E-07 | 92 | 25 | 1 |
| 3000829 | Acinetobacter baumannii AB307-0294] | NC_011595.7057524.p01 | ABBFA_000578 | 2.0E-07 | 92.31 | 26 | 2 |
| 3000830 | Klebsiella pneumoniae subsp. pneumoniae MGH 78578 | CP000647.1.gene4256.p01 | cpxA | 1.0E-11 | 93.94 | 33 | 2 |
| 3000830 | Escherichia coli O157:H7 str. Sakai | NC_002695.1.914983.p01 | cpxA | 1.0E-09 | 93.94 | 30 | 5 |
| 3000833 | Shigella dysenteriae Sd197 | CP000034.1.gene4478.p01 | evgS | 2.0E-11 | 96.97 | 33 | 3 |
| 3000833 | Escherichia coli O157:H7 str. Sakai | NC_002695.1.915650.p01 | ECs3249 | 1.0E-09 | 90.91 | 33 | 17 |
| 3000833 | Escherichia coli O157:H7 str. Sakai | NC_002695.1.915651.p01 | ECs3248 | 6.0E-07 | 100 | 25 | 2 |
| 3000834 | Shigella dysenteriae Sd197 | CP000034.1.gene2022.p01 | phoP | 2.0E-12 | 100 | 33 | 1 |
| 3000834 | Klebsiella pneumoniae subsp. pneumoniae MGH 78578 | CP000647.1.gene2625.p01 | yejM | 7.0E-10 | 100 | 28 | 2 |
| 3000835 | Escherichia coli O157:H7 str. Sakai | NC_002695.1.913289.p01 | ECs1602 | 1.0E-11 | 100 | 33 | 3 |
| 3000835 | Escherichia coli O157:H7 str. Sakai | NC_002695.1.913290.p01 | ECs1601 | 2.0E-08 | 96.97 | 29 | 4 |
| 3000836 | Salmonella enterica subsp. enterica serovar Agona str. SL483 | CP001138.1.gene4489.p01 | soxR | 2.0E-10 | 90.62 | 32 | 1 |
| 3000836 | Escherichia coli O157:H7 str. Sakai | NC_002695.1.914292.p01 | ECs5045 | 2.0E-11 | 100 | 33 | 2 |
| 3000837 | Shigella dysenteriae Sd197 | CP000034.1.gene4505.p01 | soxS | 7.0E-12 | 96.97 | 33 | 1 |
| 3000837 | Klebsiella pneumoniae subsp. pneumoniae MGH 78578 | CP000647.1.gene4499.p01 | soxS | 2.0E-11 | 100 | 31 | 2 |
| 3000837 | Escherichia coli O157:H7 str. Sakai | NC_002695.1.914293.p01 | ECs5044 | 1.0E-11 | 100 | 33 | 1 |
| 3000837 | Escherichia coli O157:H7 str. Sakai | NC_002695.1.917670.p01 | ECs0930 | 2.0E-09 | 93.94 | 33 | 2 |
| 3001205 | Campylobacter fetus subsp. fetus | FN594949.1.gene22.p01 | blmA | 8.0E-12 | 100 | 32 | 2 |
| 3001211 | Pseudomonas aeruginosa | AF024602.1.gene5.p01 | strA | 2.0E-11 | 100 | 27 | 1 |
| 3001212 | Escherichia coli | DQ464881.1.gene5.p01 | strB | 1.0E-08 | 100 | 28 | 2 |
| 3001212 | Acinetobacter baumannii AYE | NC_010410.6003392.p01 | strB | 4.0E-11 | 100 | 33 | 2 |
| 3001213 | Acinetobacter baumannii AYE | NC_010410.6003949.p01 | ABAYE3640 | 4.0E-09 | 93.55 | 31 | 2 |
| 3001214 | Salmonella enterica subsp. enterica serovar Agona str. SL483 | CP001138.1.gene4809.p01 | SeAg_B4848 | 1.0E-09 | 90.91 | 30 | 2 |
| 3001214 | Salmonella enterica subsp. enterica serovar Agona str. SL483 | CP001138.1.gene894.p01 | SeAg_B0902 | 4.0E-09 | 90.91 | 29 | 5 |
| 3001215 | Escherichia coli O157:H7 str. Sakai | NC_002695.1.915390.p01 | ECs4647 | 1.0E-09 | 93.94 | 33 | 3 |
| 3001215 | Acinetobacter baumannii AB307-0294] | NC_011595.7060598.p01 | ABBFA_001268 | 1.0E-08 | 93.94 | 30 | 3 |

**Matched contigs of sample C1754 against CARD**

(Sorted by ARO-number)

| **ARO** | **Organism** | **Accession number** | **Gene** | **E value ≤** | **Identity (%) ≥** | **Hit length (aa) ≥** | **Number of contigs** |
| --- | --- | --- | --- | --- | --- | --- | --- |
| 3000194 | Butyrivibrio fibrisolvens | AJ222769.gene.p01 | tetW* | < 1.0E-150 | 100 | 639 | 1 |
| 3000196 | Clostridiaceae bacterium K10 | AJ295238.gene.p01 | tet32 | 6E-53 | 100 | 101 | 1 |
| 3000210 | Acinetobacter baumannii ATCC 17978 | NC_009085.4918494.p01 | rpoB | 5E-74 | 95.35 | 119 | 2 |
| 3000210 | Neisseria gonorrhoeae NCCP11945 | NC_011035.1.6448762.p01 | rpoB | 2E-52 | 92 | 100 | 1 |
| 3000210 | Acinetobacter baumannii AB307-0294 | NC_011595.7060572.p01 | rpoB | 2E-14 | 95.18 | 31 | 2 |
| 3000375 | Streptococcus pneumoniae | AM903082.gene3.p01 | ermB | < 1.0E-150 | 98.78 | 245 | 1 |
| 3000533 | Acinetobacter baumannii AB307-0294 | NC_011595.7058613.p01 | ABBFA_00301 | 3.00E-61 | 90.83 | 120 | 1 |
| 3000556 | Campylobacter fetus subsp. fetus | FN594949.1.gene24.p01 | tet44 | 8.00E-72 | 90.55 | 127 | 1 |
| 3000566 | Acinetobacter sp. LUH5605 | AY743590.gene.p01 | tet39 | 1.00E-15 | 97.30 | 37 | 1 |
| 3000618 | Acinetobacter baumannii AB0057 | NC_011586.7046500.p01 | gyrA | 1.00E-30 | 98.25 | 57 | 1 |
| 3000618 | Azoarcus sp. BH72 | NC_008702.1.4606680.p01 | gyrA | 2.00E-42 | 94.74 | 76 | 1 |

**Matched high-throughput sequencing reads of sample C1755 against CARD**

(Sorted by ARO-number)

| **ARO** | **Organism** | **Accession number** | **Gene** | **E value ≤** | **Identity (%) ≥** | **Hit length (aa) ≥** | **Number of reads** |
| --- | --- | --- | --- | --- | --- | --- | --- |
| 3000016 | Citrobacter freundii | Y10278.1.gene1.p01 | blaCTX-M 3 | 3.0E-10 | 90.62 | 32 | 1 |
| 3000017 | Pseudomonas aeruginosa | AF024602.1.gene3.p01 | blaOXA-20 | 1.0E-12 | 100 | 33 | 1 |
| 3000017 | Pseudomonas aeruginosa | AF300985.1.gene1.p01 | blaOXA-2-related | 6.0E-11 | 100 | 30 | 3 |
| 3000017 | Pseudomonas aeruginosa | U59183.1.gene3.p01 | blaOXA-13 | 2.0E-10 | 93.94 | 30 | 11 |
| 3000017 | Pseudomonas aeruginosa | X58272.1.gene1.p01 | blaOXA-5 | 7.0E-12 | 100 | 33 | 1 |
| 3000017 | Brachyspira pilosicoli | JF273470.1.gene1.p01 | blaOXA-192 | 2.0E-11 | 90.91 | 33 | 1 |
| 3000017 | Burkholderia cepacia | AF371964.1.gene1.p1 | AAK55330 | 4.0E-10 | 93.94 | 29 | 7 |
| 3000017 | Pseudomonas aeruginosa | AF317511.1.gene5.p01 | blaOXA | 6.0E-10 | 90.91 | 33 | 3 |
| 3000017 | Pseudomonas aeruginosa | AJ854182.1.gene1.p01 | blaOXA-74 | 3.0E-09 | 90.91 | 33 | 1 |
| 3000017 | Pseudomonas aeruginosa | EF552405.1.gene1.p01 | blaOXA-141 | 3.0E-08 | 100 | 26 | 1 |
| 3000017 | Pseudomonas aeruginosa | U37105.2.gene2.p01 | blaOXA10 | 2.0E-11 | 90.91 | 33 | 7 |
| 3000017 | Pseudomonas aeruginosa | U63835.1.gene1.p01 | blaOXA-15 | 4.0E-07 | 100 | 25 | 6 |
| 3000017 | Salmonella enterica subsp. enterica serovar Bredeney | AM932669.1.gene2.p01 | blaOXA-129 | 9.0E-12 | 96.97 | 33 | 7 |
| 3000017 | Acinetobacter lwoffii | HQ122933.1.gene1.p01 | blaOXA-134a | 3.0E-10 | 90.91 | 33 | 2 |
| 3000017 | Acinetobacter baumannii | JN638887.1.gene1.p01 | blaOXA-225 | 3.0E-11 | 90.91 | 33 | 1 |
| 3000017 | Acinetobacter baumannii | AY570763.1.gene3.p01 | blaOXA-58 | 5.0E-12 | 100 | 33 | 1 |
| 3000017 | Klebsiella pneumoniae | AY237830.1.gene1.p01 | blaOXA-47 | 4.0E-13 | 100 | 33 | 1 |
| 3000017 | Pseudomonas aeruginosa | JF800667.1.gene2.p01 | blaOXA-205 | 5.0E-13 | 100 | 33 | 1 |
| 3000019 | Escherichia coli O157:H7 str. Sakai | NC_002695.1.914045.p01 | ampC | 5.0E-11 | 96.97 | 33 | 4 |
| 3000027 | Acinetobacter baumannii AYE | NC_010410.6003177.p01 | emrA | 7.0E-09 | 90.62 | 31 | 7 |
| 3000027 | Escherichia coli O157:H7 str. Sakai | NC_002695.1.914737.p01 | ECs3547 | 1.0E-10 | 90.62 | 32 | 4 |
| 3000027 | Klebsiella pneumoniae subsp. pneumoniae MGH 78578 | CP000647.1.gene3014.p01 | emrA | 1.0E-08 | 90 | 30 | 1 |
| 3000054 | Escherichia coli O157:H7 str. Sakai | NC_002695.1.914620.p01 | ECs0516 | 9.0E-10 | 100 | 29 | 3 |
| 3000066 | Acinetobacter baumannii | FN554543.1.gene1.p01 | blaGES-12 | 3.0E-11 | 100 | 33 | 1 |
| 3000066 | Pseudomonas aeruginosa | AF326355.1.gene1.p01 | blaGES-2 | 6.0E-08 | 96.97 | 25 | 8 |
| 3000071 | Enterococcus faecium | AF130997.1.orf1.gene.p01 | vanSD | 2.0E-10 | 96.97 | 33 | 1 |
| 3000074 | Acinetobacter baumannii AYE | NC_010410.6003262.p01 | emrB | 3.0E-09 | 93.33 | 30 | 3 |
| 3000074 | Enterococcus faecalis V583 | AE016830.1.gene768.p01 | EF_0785 | 1.0E-11 | 100 | 33 | 1 |
| 3000074 | Escherichia coli O157:H7 str. Sakai | NC_002695.1.914736.p01 | ECs3548 | 1.0E-10 | 96.97 | 33 | 2 |
| 3000074 | Salmonella enterica subsp. enterica serovar Agona str. SL483 | CP001138.1.gene2906.p01 | emrB | 1.0E-09 | 100 | 31 | 4 |
| 3000091 | Acinetobacter calcoaceticus subsp. anitratus | AF135373.1.gene1.p01 | blaCARB-5 | 4.0E-11 | 96.97 | 33 | 2 |
| 3000092 | Pseudomonas aeruginosa | Z22590.1.gene1.p01 | blaZ | 8.0E-11 | 96.97 | 33 | 5 |
| 3000122 | Acinetobacter baumannii AB307-0294 | NC_011595.7057747.p01 | ABBFA_002299 | 2.0E-10 | 90 | 30 | 6 |
| 3000122 | Acinetobacter baumannii AYE | NC_010410.6000796.p01 | cat | 1.0E-12 | 93.75 | 32 | 4 |
| 3000122 | Staphylococcus aureus | NC_010426.6155849.p01 | pKH13_02 | 5.0E-12 | 96.97 | 33 | 1 |
| 3000126 | Enterococcus faecalis | V01547.1.orf0.gene.p01 | V01547 | 7.0E-12 | 100 | 33 | 2 |
| 3000165 | Escherichia coli 1520 | NC_010558.1.6275971.p01 | tetA(B) | 1.0E-11 | 100 | 33 | 1 |
| 3000165 | Pseudomonas aeruginosa | X75761.gene.p01 | tetA | 6.0E-12 | 100 | 33 | 1 |
| 3000165 | Shigella sonnei | AF534183.gene.p01 | tetA | 3.0E-06 | 100 | 25 | 1 |
| 3000167 | Escherichia coli | Y19114.gene.p01 | tetC | 2.0E-11 | 100 | 33 | 2 |
| 3000174 | Pseudomonas sp. | AF133139.gene.p01 | tetG | 2.0E-11 | 96.88 | 32 | 2 |
| 3000174 | Pseudomonas sp. | AF133140.gene.p01 | tetG | 1.0E-06 | 96.15 | 26 | 2 |
| 3000186 | Enterococcus faecalis | M85225.gene.p01 | tetM | 2.0E-08 | 96.97 | 26 | 9 |
| 3000186 | Enterococcus faecalis | X04388.gene.p01 | tetM | 3.0E-10 | 90.91 | 33 | 12 |
| 3000186 | Staphylococcus aureus subsp. aureus Mu50 | NC_002758.1120355.p01 | tetM | 1.0E-12 | 100 | 33 | 1 |
| 3000190 | Megasphaera elsdenii | AY485126.gene.p01 | tetOW | 5.0E-08 | 90.91 | 27 | 6 |
| 3000191 | Bacteroides fragilis | Z21523.gene.p01 | tetQ | 9.0E-07 | 93.94 | 25 | 10 |
| 3000193 | Streptococcus pyogenes | L42544.gene.p01 | tetT* | 5.0E-12 | 100 | 33 | 2 |
| 3000194 | Bifidobacterium longum subsp. longum F8 | DQ294299.gene.p01 | tetW_2 | 5.0E-09 | 100 | 26 | 18 |
| 3000194 | Butyrivibrio fibrisolvens | AJ222769.gene.p01 | tetW* | 2.0E-07 | 93.94 | 25 | 45 |
| 3000196 | Clostridiaceae bacterium K10 | AJ295238.gene.p01 | tet32 | 6.0E-09 | 90.91 | 27 | 38 |
| 3000207 | Escherichia coli O157:H7 str. Sakai | NC_002695.1.912781.p01 | ECs1863 | 5.0E-07 | 90.91 | 26 | 5 |
| 3000210 | Acinetobacter baumannii ATCC 17978 | NC_009085.4918494.p01 | rpoB | 5.0E-06 | 90.32 | 25 | 123 |
| 3000210 | Acinetobacter baumannii AYE | NC_010410.6003841.p01 | rpoB | 6.0E-11 | 93.94 | 33 | 1 |
| 3000210 | Acinetobacter baumannii SDF | NC_010400.5987325.p01 | rpoB | 6.0E-09 | 93.75 | 28 | 13 |
| 3000210 | Azoarcus sp. BH72 | NC_008702.1.4609796.p01 | rpoB | 3.0E-06 | 90 | 25 | 175 |
| 3000210 | Bordetella pertussis CS | CP002695.1.gene18.p01 | rpoB | 6.0E-07 | 90 | 25 | 217 |
| 3000210 | Clostridium botulinum A2 str. Kyoto | CP001581.1.gene3846.p01 | rpoB | 4.0E-06 | 90.32 | 25 | 46 |
| 3000210 | Clostridium difficile 630 | AM180355.1.gene120.p01 | rpoB | 8.0E-07 | 90.32 | 25 | 53 |
| 3000210 | Enterococcus faecalis V583 | AE016830.1.gene3155.p01 | rpoB | 8.0E-07 | 90.62 | 25 | 99 |
| 3000210 | Enterococcus faecium DO | CP003583.1.gene2745.p01 | rpoB | 8.0E-07 | 90.32 | 25 | 24 |
| 3000210 | Escherichia coli O157:H7 str. Sakai | NC_002695.1.914942.p01 | rpoB | 2.0E-10 | 96.97 | 30 | 14 |
| 3000210 | Klebsiella pneumoniae subsp. pneumoniae MGH 78578 | CP000647.1.gene4402.p01 | rpoB | 1.0E-09 | 93.94 | 29 | 4 |
| 3000210 | Legionella pneumophila str. Corby | CP000675.2.gene392.p01 | rpoB | 2.0E-06 | 90.91 | 26 | 36 |
| 3000210 | Mycobacterium tuberculosis CDC1551 | AE000516.2.gene708.p01 | rpoB | 4.0E-07 | 90.32 | 25 | 31 |
| 3000210 | Neisseria gonorrhoeae NCCP11945 | NC_011035.1.6448762.p01 | rpoB | 3.0E-06 | 90 | 25 | 128 |
| 3000210 | Proteus mirabilis BB2000 | CP004022.1.gene2794.p01 | rpoB | 3.0E-08 | 90.91 | 30 | 5 |
| 3000210 | Pseudomonas aeruginosa PAO1 | NC_002516.2.881699.p01 | rpoB | 2.0E-06 | 90.32 | 26 | 68 |
| 3000210 | Salmonella enterica subsp. enterica serovar Agona str. SL483 | CP001138.1.gene4362.p01 | rpoB | 3.0E-08 | 90.62 | 25 | 36 |
| 3000210 | Shigella dysenteriae Sd197 | CP000034.1.gene3741.p01 | rpoB | 5.0E-12 | 100 | 33 | 2 |
| 3000210 | Staphylococcus aureus subsp. aureus COL | NC_002951.3236234.p01 | rpoB | 3.0E-14 | 100 | 33 | 7 |
| 3000210 | Staphylococcus aureus subsp. aureus JH9 | NC_009487.5169226.p01 | rpoB | 4.0E-09 | 93.1 | 29 | 1 |
| 3000210 | Staphylococcus aureus subsp. aureus Mu50 | NC_002758.1120515.p01 | rpoB | 5.0E-07 | 92.59 | 27 | 2 |
| 3000210 | Staphylococcus aureus subsp. aureus N315 | NC_002745.1123305.p01 | rpoB | 6.0E-07 | 90.62 | 26 | 45 |
| 3000210 | Streptococcus pneumoniae Taiwan19F-14 | NC_012469.1.7686402.p01 | rpoB | 2.0E-08 | 90.62 | 29 | 34 |
| 3000216 | Shigella dysenteriae Sd197 | CP000034.1.gene457.p01 | acrB | 2.0E-07 | 90.62 | 32 | 5 |
| 3000216 | Klebsiella pneumoniae subsp. pneumoniae MGH 78578 | CP000647.1.gene443.p01 | acrB | 4.0E-09 | 90.91 | 33 | 3 |
| 3000216 | Escherichia coli O157:H7 str. Sakai | NC_002695.1.914619.p01 | ECs0515 | 2.0E-08 | 90 | 27 | 15 |
| 3000216 | Enterobacter cloacae subsp. cloacae ATCC 13047 | CP001918.1.gene1257.p01 | acrB | 1.0E-09 | 90.91 | 31 | 4 |
| 3000216 | Escherichia coli O157:H7 str. Sakai | NC_002695.1.912777.p01 | ECs1864 | 9.0E-07 | 90.91 | 26 | 16 |
| 3000216 | Bordetella pertussis CS | CP002695.1.gene992.p01 | acrB | 1.0E-06 | 90 | 27 | 11 |
| 3000226 | Shigella dysenteriae Sd197 | CP000034.1.gene3358.p01 | folP | 4.0E-11 | 96.97 | 33 | 2 |
| 3000232 | Acinetobacter baumannii AYE | NC_010410.6002671.p01 | ABAYE3739 | 2.0E-12 | 90.91 | 33 | 1 |
| 3000232 | Acinetobacter baumannii AYE | NC_010410.6003170.p01 | aadA1 | 6.0E-13 | 100 | 33 | 1 |
| 3000232 | Escherichia coli 1520 | NC_010558.1.6275994.p01 | aadA4 | 5.0E-07 | 96 | 25 | 27 |
| 3000232 | Pseudomonas aeruginosa | AF294653.1.gene3.p01 | aadA2a | 2.0E-11 | 96.97 | 33 | 2 |
| 3000232 | Pseudomonas aeruginosa | U37105.2.gene4.p01 | aadA10 | 1.0E-09 | 90.91 | 32 | 28 |
| 3000232 | Salmonella enterica subsp. enterica serovar Bredeney | AM932669.1.gene3.p01 | aadA1 | 9.0E-11 | 100 | 33 | 1 |
| 3000232 | Salmonella enterica subsp. enterica serovar Stanley | EU118119.1.orf1.gene.p01 | aadA2 | 1.0E-09 | 90.32 | 31 | 1 |
| 3000232 | Salmonella enterica subsp. enterica serovar Typhi | AY123251.gene3.p01 | aadA1 | 1.0E-10 | 93.94 | 33 | 6 |
| 3000232 | uncultured bacterium | AY139598.1.gene2.p01 | aadA5 | 5.0E-11 | 100 | 33 | 1 |
| 3000237 | Escherichia coli O157:H7 str. Sakai | NC_002695.1.916248.p01 | tolC | 8.0E-12 | 96.97 | 33 | 2 |
| 3000237 | Shigella dysenteriae Sd197 | CP000034.1.gene3205.p01 | tolC | 5.0E-11 | 96.97 | 33 | 2 |
| 3000263 | Salmonella enterica subsp. enterica serovar Agona str. SL483 | CP001138.1.gene1637.p01 | SeAg_B1651 | 2.0E-12 | 100 | 33 | 1 |
| 3000275 | Enterococcus faecium | AF110130.1.orf0.gene.p01 | linB | 2.0E-08 | 93.1 | 28 | 20 |
| 3000309 | Escherichia coli O157:H7 str. Sakai | NC_002695.1.915420.p01 | emrD | 4.0E-06 | 100 | 25 | 3 |
| 3000309 | Salmonella enterica subsp. enterica serovar Agona str. SL483 | CP001138.1.gene3995.p01 | emrD | 9.0E-13 | 100 | 33 | 1 |
| 3000322 | Acinetobacter baumannii AYE | NC_010410.6002585.p01 | aacC1 | 5.0E-12 | 100 | 33 | 1 |
| 3000345 | Salmonella enterica subsp. enterica serovar Typhi | AY123251.gene5.p01 | aac6-II | 7.0E-12 | 93.94 | 33 | 1 |
| 3000373 | Escherichia coli O157:H7 str. Sakai | NC_002695.1.915653.p01 | ECs3247 | 9.0E-12 | 100 | 33 | 2 |
| 3000373 | Shigella dysenteriae Sd197 | CP000034.1.gene2563.p01 | emrK | 1.0E-10 | 96.97 | 33 | 1 |
| 3000375 | Clostridium difficile 630 | AM180355.1.gene2254.p01 | ermB | 6.0E-08 | 93.75 | 28 | 26 |
| 3000375 | Streptococcus pneumoniae | AM410044.gene14.p01 | ermB | 1.0E-06 | 93.94 | 26 | 159 |
| 3000378 | Acinetobacter baumannii AB307-0294 | NC_011595.7057907.p01 | ABBFA_000816 | 2.0E-06 | 90.91 | 27 | 6 |
| 3000378 | Pseudomonas aeruginosa PAO1 | NC_002516.2.877852.p01 | mexB | 9.0E-08 | 90 | 30 | 14 |
| 3000379 | Acinetobacter baumannii AB307-0294 | NC_011595.7058890.p01 | ABBFA_003020 | 8.0E-11 | 90.91 | 33 | 1 |
| 3000410 | Pseudomonas aeruginosa | AF191564.1.gene5.p01 | sul1 | 6.0E-12 | 100 | 33 | 2 |
| 3000410 | Pseudomonas aeruginosa | U37105.2.gene6.p01 | sul1 | 2.0E-07 | 100 | 26 | 5 |
| 3000412 | Escherichia coli | DQ464881.1.gene2.p01 | sul2 | 1.0E-08 | 93.55 | 31 | 1 |
| 3000457 | Acinetobacter baumannii AYE | NC_010410.6000336.p01 | parE | 4.0E-09 | 90.62 | 30 | 8 |
| 3000457 | Acinetobacter baumannii SDF | NC_010400.5986295.p01 | parE | 8.0E-08 | 90.32 | 26 | 39 |
| 3000457 | Azoarcus sp. BH72 | NC_008702.1.4609137.p01 | parE | 9.0E-09 | 90 | 30 | 18 |
| 3000457 | Bordetella pertussis CS | CP002695.1.gene1273.p01 | parE | 5.0E-07 | 90 | 25 | 109 |
| 3000457 | Enterococcus faecalis V583 | AE016830.1.gene1599.p01 | parE | 4.0E-06 | 90.91 | 26 | 16 |
| 3000457 | Enterococcus faecium DO | CP003583.1.gene1173.p01 | parE | 4.0E-09 | 90 | 30 | 6 |
| 3000457 | Klebsiella pneumoniae subsp. pneumoniae MGH 78578 | CP000647.1.gene3444.p01 | parE | 6.0E-12 | 100 | 33 | 1 |
| 3000457 | Pseudomonas aeruginosa PAO1 | NC_002516.2.879897.p01 | parE | 3.0E-07 | 90.91 | 26 | 14 |
| 3000457 | Salmonella enterica subsp. enterica serovar Agona str. SL483 | CP001138.1.gene3336.p01 | parE | 1.0E-07 | 100 | 25 | 3 |
| 3000457 | Shigella dysenteriae Sd197 | CP000034.1.gene3210.p01 | parE | 5.0E-08 | 96.55 | 29 | 5 |
| 3000457 | Staphylococcus aureus subsp. aureus N315 | NC_002745.1124025.p01 | parE | 3.0E-10 | 90.62 | 32 | 5 |
| 3000457 | Streptococcus pneumoniae Taiwan19F-14 | NC_012469.1.7686068.p01 | parE | 3.0E-08 | 90.32 | 27 | 10 |
| 3000461 | Clostridium botulinum A2 str. Kyoto | CP001581.1.gene3143.p01 | CLM_3236 | 4.0E-09 | 93.94 | 28 | 5 |
| 3000479 | Streptomyces caeruleus | AF205854.1.orf0.gene.p01 | gyrB-Rn | 6.0E-09 | 90.62 | 32 | 2 |
| 3000479 | Streptomyces roseochromogenes subsp. oscitans | AY136281.1.orf1.gene.p01 | gyrBR | 3.0E-11 | 90.91 | 33 | 1 |
| 3000480 | Streptomyces rishiriensis | AF205853.1.orf1.gene.p01 | parYR | 2.0E-07 | 96.3 | 27 | 2 |
| 3000480 | Streptomyces roseochromogenes subsp. oscitans | AY136281.1.orf0.gene.p01 | parYR | 3.0E-10 | 90.62 | 32 | 3 |
| 3000489 | Staphylococcus aureus subsp. aureus Mu50 | NC_002758.1121879.p01 | SAV1866 | 7.0E-10 | 90.62 | 32 | 1 |
| 3000491 | Escherichia coli O157:H7 str. Sakai | NC_002695.1.915267.p01 | ECs3332 | 1.0E-08 | 90.91 | 26 | 8 |
| 3000491 | Klebsiella pneumoniae subsp. pneumoniae MGH 78578 | CP000647.1.gene2803.p01 | acrD | 3.0E-09 | 90.91 | 31 | 5 |
| 3000491 | Salmonella enterica subsp. enterica serovar Agona str. SL483 | CP001138.1.gene2601.p01 | acrD | 4.0E-08 | 90.32 | 29 | 9 |
| 3000493 | Azoarcus sp. BH72 | NC_008702.1.4607594.p01 | ompR2 | 5.0E-09 | 90.91 | 33 | 1 |
| 3000493 | Escherichia coli | GQ465831.1.gene2.p01 | ompF | 1.0E-11 | 100 | 33 | 5 |
| 3000493 | Shigella dysenteriae Sd197 | CP000034.1.gene2198.p01 | asmA | 1.0E-11 | 96.97 | 33 | 3 |
| 3000493 | Shigella dysenteriae Sd197 | CP000034.1.gene3671.p01 | ompR | 1.0E-10 | 90.91 | 33 | 6 |
| 3000493 | Shigella dysenteriae Sd197 | CP000034.1.gene3672.p01 | envZ | 3.0E-10 | 96.97 | 33 | 8 |
| 3000498 | Bacteroides fragilis | M14730.gene.p01 | ermF* | 2.0E-09 | 100 | 29 | 2 |
| 3000499 | Escherichia coli O157:H7 str. Sakai | NC_002695.1.916015.p01 | ECs4137 | 2.0E-11 | 100 | 32 | 2 |
| 3000502 | Klebsiella pneumoniae subsp. pneumoniae MGH 78578 | CP000647.1.gene3710.p01 | acrF | 9.0E-10 | 90.91 | 33 | 3 |
| 3000506 | Azoarcus sp. BH72 | NC_008702.1.4610136.p01 | mexR | 6.0E-08 | 92.59 | 27 | 2 |
| 3000508 | Escherichia coli O157:H7 str. Sakai | NC_002695.1.915747.p01 | ECs4396 | 3.0E-10 | 90.91 | 33 | 3 |
| 3000516 | Acinetobacter baumannii AB307-0294 | NC_011595.7058564.p01 | ABBFA_001502 | 4.0E-08 | 93.1 | 29 | 2 |
| 3000516 | Shigella dysenteriae Sd197 | CP000034.1.gene2879.p01 | emrR | 2.0E-11 | 96.97 | 33 | 1 |
| 3000518 | Proteus mirabilis BB2000 | CP004022.1.gene2827.p01 | crp | 1.0E-12 | 100 | 33 | 1 |
| 3000518 | Shigella dysenteriae Sd197 | CP000034.1.gene3519.p01 | crp | 2.0E-08 | 100 | 28 | 3 |
| 3000522 | Lysinibacillus sphaericus | M15332.gene.p01 | erm(G)**_ermG* | 2.0E-08 | 93.94 | 27 | 2 |
| 3000533 | Acinetobacter baumannii AB307-0294 | NC_011595.7058613.p01 | ABBFA_003018 | 2.0E-10 | 93.94 | 33 | 3 |
| 3000533 | Azoarcus sp. BH72 | NC_008702.1.4609191.p01 | macA | 4.0E-10 | 90.62 | 32 | 2 |
| 3000533 | Escherichia coli O157:H7 str. Sakai | NC_002695.1.917702.p01 | ECs0964 | 5.0E-11 | 100 | 33 | 3 |
| 3000535 | Acinetobacter baumannii AB307-0294 | NC_011595.7060505.p01 | ABBFA_003019 | 4.0E-06 | 90.91 | 26 | 20 |
| 3000535 | Acinetobacter baumannii SDF | NC_010400.5985985.p01 | macB | 7.0E-10 | 90.91 | 33 | 2 |
| 3000535 | Azoarcus sp. BH72 | NC_008702.1.4606597.p01 | azo0833 | 7.0E-08 | 90.91 | 33 | 3 |
| 3000535 | Azoarcus sp. BH72 | NC_008702.1.4606598.p01 | azo0834 | 7.0E-10 | 90.91 | 33 | 2 |
| 3000535 | Azoarcus sp. BH72 | NC_008702.1.4609454.p01 | macB | 4.0E-09 | 90.91 | 33 | 8 |
| 3000535 | Clostridium botulinum A2 str. Kyoto | CP001581.1.gene598.p01 | CLM_0622 | 3.0E-06 | 90 | 25 | 6 |
| 3000535 | Clostridium botulinum A2 str. Kyoto | CP001581.1.gene798.p01 | CLM_0827 | 3.0E-08 | 90.62 | 28 | 13 |
| 3000535 | Klebsiella pneumoniae subsp. pneumoniae MGH 78578 | CP000647.1.gene912.p01 | macB | 3.0E-11 | 90.91 | 33 | 1 |
| 3000535 | Listeria monocytogenes | HE999704.1.gene196.p01 | BN418_0205 | 1.0E-07 | 92.86 | 28 | 2 |
| 3000535 | Proteus mirabilis BB2000 | CP004022.1.gene758.p01 | macB | 8.0E-07 | 90.91 | 25 | 2 |
| 3000535 | Salmonella enterica subsp. enterica serovar Agona str. SL483 | CP001138.1.gene935.p01 | SeAg_B0943 | 8.0E-10 | 90.91 | 33 | 2 |
| 3000535 | Streptococcus pneumoniae Taiwan19F-14 | NC_012469.1.7685735.p01 | SPT_1593 | 2.0E-10 | 90.32 | 31 | 4 |
| 3000556 | Campylobacter fetus subsp. fetus | FN594949.1.gene24.p01 | tet44 | 4.0E-10 | 96.97 | 33 | 7 |
| 3000559 | Shigella dysenteriae Sd197 | CP000034.1.gene1340.p01 | btuR | 7.0E-12 | 93.94 | 33 | 3 |
| 3000566 | Acinetobacter sp. LUH5605 | AY743590.gene.p01 | tet39 | 2.0E-07 | 96.97 | 28 | 22 |
| 3000574 | Acinetobacter baumannii AB307-0294 | NC_011595.7059276.p01 | ABBFA_002430 | 1.0E-09 | 90.62 | 32 | 1 |
| 3000574 | Clostridium difficile 630 | AM180355.1.gene1830.p01 | vanR | 7.0E-11 | 93.94 | 33 | 1 |
| 3000574 | Streptomyces toyocaensis | U82965.2.orf14.gene.p01 | U82965 | 2.0E-08 | 92.86 | 28 | 3 |
| 3000581 | Bordetella pertussis CS | CP002695.1.gene1737.p01 | cphA | 8.0E-08 | 92.59 | 27 | 1 |
| 3000581 | Bordetella pertussis CS | CP002695.1.gene1738.p01 | cphA | 5.0E-07 | 90.32 | 25 | 2 |
| 3000616 | Streptococcus pneumoniae Taiwan19F-14 | NC_012469.1.7685970.p01 | SPT_1925 | 5.0E-07 | 90.62 | 26 | 40 |
| 3000618 | Acinetobacter baumannii AB307-0294 | NC_011595.7058445.p01 | gyrA | 9.0E-08 | 90.62 | 28 | 49 |
| 3000618 | Acinetobacter baumannii AYE | NC_010410.6003186.p01 | gyrA | 8.0E-13 | 93.94 | 33 | 1 |
| 3000618 | Acinetobacter baumannii SDF | NC_010400.5986734.p01 | gyrA | 5.0E-10 | 90.91 | 33 | 3 |
| 3000618 | Azoarcus sp. BH72 | NC_008702.1.4606680.p01 | gyrA | 2.0E-06 | 90 | 25 | 109 |
| 3000618 | Bordetella pertussis CS | CP002695.1.gene952.p01 | gyrA | 2.0E-06 | 90 | 25 | 154 |
| 3000618 | Clostridium botulinum A2 str. Kyoto | CP001581.1.gene7.p01 | gyrA | 9.0E-09 | 90.32 | 31 | 2 |
| 3000618 | Clostridium difficile 630 | AM180355.1.gene6.p01 | gyrA | 2.0E-09 | 90.91 | 33 | 3 |
| 3000618 | Enterobacter cloacae subsp. cloacae ATCC 13047 | CP001918.1.gene3562.p01 | ECL_03523 | 5.0E-08 | 96.3 | 27 | 1 |
| 3000618 | Enterococcus faecalis V583 | AE016830.1.gene6.p01 | gyrA | 2.0E-06 | 90.91 | 26 | 26 |
| 3000618 | Escherichia coli O157:H7 str. Sakai | NC_002695.1.916822.p01 | ECs3114 | 3.0E-07 | 90 | 25 | 8 |
| 3000618 | Haemophilus influenzae 10810 | FQ312006.1.gene1417.p01 | HIB_14190 | 4.0E-12 | 96.97 | 33 | 3 |
| 3000618 | Helicobacter pylori Gambia94/24 | CP002332.1.gene704.p01 | HPGAM_03615 | 9.0E-08 | 90.62 | 25 | 9 |
| 3000618 | Legionella pneumophila str. Corby | CP000675.2.gene1514.p01 | gyrA | 6.0E-08 | 90.62 | 27 | 3 |
| 3000618 | Listeria monocytogenes | HE999704.1.gene7.p01 | BN418_0007 | 3.0E-08 | 90.62 | 28 | 3 |
| 3000618 | Mycobacterium tuberculosis CDC1551 | AE000516.2.gene6.p01 | gyrA | 4.0E-10 | 90.32 | 31 | 4 |
| 3000618 | Neisseria gonorrhoeae NCCP11945 | NC_011035.1.6447337.p01 | NGK_1285 | 1.0E-09 | 90.91 | 33 | 2 |
| 3000618 | Proteus mirabilis BB2000 | CP004022.1.gene1837.p01 | gyrA | 1.0E-06 | 92.59 | 27 | 2 |
| 3000618 | Pseudomonas aeruginosa PAO1 | NC_002516.2.882800.p01 | gyrA | 1.0E-10 | 90.62 | 32 | 10 |
| 3000618 | Salmonella enterica subsp. enterica serovar Agona str. SL483 | CP001138.1.gene2385.p01 | gyrA | 7.0E-08 | 90.32 | 25 | 8 |
| 3000618 | Staphylococcus aureus subsp. aureus COL | NC_002951.3236187.p01 | gyrA | 1.0E-13 | 90.91 | 33 | 2 |
| 3000618 | Staphylococcus aureus subsp. aureus MSSA476 | NC_002953.2863526.p01 | SAS0006 | 7.0E-11 | 90.91 | 33 | 1 |
| 3000618 | Staphylococcus aureus subsp. aureus Mu50 | NC_002758.1119966.p01 | gyrA | 3.0E-11 | 90.91 | 33 | 1 |
| 3000618 | Staphylococcus aureus subsp. aureus N315 | NC_002745.1122777.p01 | gyrA | 4.0E-11 | 90.91 | 33 | 2 |
| 3000618 | Staphylococcus epidermidis ATCC 12228 | AE015929.1.gene5.p01 | SE_0005 | 1.0E-12 | 93.94 | 33 | 1 |
| 3000618 | Streptococcus pneumoniae Taiwan19F-14 | NC_012469.1.7686721.p01 | gyrA | 2.0E-07 | 90 | 29 | 6 |
| 3000618 | Vibrio cholerae MJ-1236 | CP001485.1.gene2164.p01 | VCD_003093 | 3.0E-08 | 90.32 | 31 | 4 |
| 3000618 | Yersinia pestis Antiqua | CP000308.1.gene965.p01 | YPA_0930 | 2.0E-06 | 90.32 | 25 | 13 |
| 3000619 | Acinetobacter baumannii ACICU | NC_010611.6237080.p01 | parC | 6.0E-11 | 93.94 | 32 | 2 |
| 3000619 | Acinetobacter baumannii AYE | NC_010410.6003198.p01 | parC | 1.0E-13 | 90.91 | 33 | 1 |
| 3000619 | Acinetobacter baumannii SDF | NC_010400.5984045.p01 | parC | 2.0E-08 | 90.91 | 28 | 21 |
| 3000619 | Bordetella pertussis CS | CP002695.1.gene1275.p01 | parC | 4.0E-06 | 90 | 25 | 15 |
| 3000619 | Enterococcus faecalis V583 | AE016830.1.gene1598.p01 | parC | 5.0E-11 | 90.91 | 32 | 5 |
| 3000619 | Escherichia coli str. K-12 substr. W3110 | M58408.gene.p01 | parC | 2.0E-07 | 100 | 25 | 4 |
| 3000619 | Klebsiella pneumoniae subsp. pneumoniae MGH 78578 | CP000647.1.gene3437.p01 | parC | 6.0E-12 | 93.94 | 33 | 1 |
| 3000619 | Legionella pneumophila str. Corby | CP000675.2.gene3231.p01 | parC | 1.0E-09 | 90.91 | 33 | 3 |
| 3000619 | Pseudomonas aeruginosa PAO1 | NC_002516.2.879741.p01 | parC | 2.0E-06 | 90.91 | 25 | 5 |
| 3000619 | Salmonella enterica subsp. enterica serovar Agona str. SL483 | CP001138.1.gene3329.p01 | parC | 4.0E-12 | 100 | 33 | 1 |
| 3000619 | Staphylococcus aureus subsp. aureus N315 | NC_002745.1124026.p01 | parC | 9.0E-10 | 93.33 | 30 | 1 |
| 3000619 | Streptococcus pneumoniae Taiwan19F-14 | NC_012469.1.7685406.p01 | parC | 4.0E-11 | 90.32 | 31 | 1 |
| 3000621 | Acinetobacter baumannii AB0057 | NC_011586.7045516.p01 | AB57_0437 | 3.0E-08 | 90.32 | 30 | 5 |
| 3000621 | Azoarcus sp. BH72 | NC_008702.1.4608898.p01 | azo0443 | 2.0E-08 | 90 | 27 | 8 |
| 3000662 | Escherichia coli O157:H7 str. Sakai | NC_002695.1.912474.p01 | ECs1443 | 5.0E-11 | 96.97 | 32 | 2 |
| 3000676 | Acinetobacter baumannii AB0057 | NC_011586.7046013.p01 | AB57_0355 | 8.0E-10 | 96.97 | 33 | 1 |
| 3000676 | Escherichia coli O157:H7 str. Sakai | NC_002695.1.913113.p01 | ECs1739 | 3.0E-12 | 100 | 32 | 1 |
| 3000702 | Shigella dysenteriae Sd197 | CP000034.1.gene455.p01 | acrR | 4.0E-08 | 100 | 26 | 2 |
| 3000718 | Acinetobacter baumannii ATCC 19606 | NC_006877.3293011.p01 | marR | 6.0E-11 | 96.97 | 33 | 2 |
| 3000753 | Acinetobacter baumannii AB0057 | NC_011586.7045550.p01 | abeM | 1.0E-06 | 90.91 | 26 | 4 |
| 3000774 | Acinetobacter baumannii SDF | NC_010400.5984386.p01 | ABSDF0738 | 1.0E-09 | 96.77 | 31 | 1 |
| 3000776 | Enterococcus faecium DO | CP003583.1.gene2512.p01 | adeC | 5.0E-12 | 100 | 33 | 1 |
| 3000778 | Acinetobacter baumannii SDF | NC_010400.5984909.p01 | ABSDF1462 | 6.0E-06 | 90.91 | 26 | 9 |
| 3000780 | Acinetobacter baumannii ATCC 17978 | NC_009085.4918693.p01 | A1S_2735 | 7.0E-08 | 90.32 | 28 | 6 |
| 3000781 | Acinetobacter baumannii AB0057 | NC_011586.7045444.p01 | adeJ | 2.0E-06 | 90.62 | 26 | 31 |
| 3000782 | Acinetobacter baumannii AB0057 | NC_011586.7045445.p01 | adeK | 6.0E-09 | 90.32 | 29 | 5 |
| 3000782 | Acinetobacter baumannii ATCC 17978 | NC_009085.4918695.p01 | A1S_2737 | 3.0E-10 | 90.62 | 32 | 5 |
| 3000792 | Escherichia coli O157:H7 str. Sakai | NC_002695.1.916584.p01 | ECs2882 | 6.0E-12 | 96.97 | 33 | 1 |
| 3000793 | Escherichia coli O157:H7 str. Sakai | NC_002695.1.916585.p01 | ECs2883 | 3.0E-07 | 90.62 | 26 | 21 |
| 3000793 | Proteus mirabilis BB2000 | CP004022.1.gene1673.p01 | mdtB | 7.0E-09 | 90.62 | 32 | 2 |
| 3000793 | Salmonella enterica subsp. enterica serovar Agona str. SL483 | CP001138.1.gene2235.p01 | SeAg_B2257 | 2.0E-06 | 90 | 25 | 8 |
| 3000794 | Enterobacter cloacae subsp. cloacae ATCC 13047 | CP001918.1.gene3441.p01 | ECL_03403 | 1.0E-08 | 90.32 | 31 | 6 |
| 3000794 | Escherichia coli O157:H7 str. Sakai | NC_002695.1.916586.p01 | ECs2884 | 3.0E-08 | 93.94 | 28 | 7 |
| 3000794 | Proteus mirabilis BB2000 | CP004022.1.gene1674.p01 | mdtC | 7.0E-09 | 90.91 | 31 | 20 |
| 3000794 | Salmonella enterica subsp. enterica serovar Agona str. SL483 | CP001138.1.gene2236.p01 | mdtC | 4.0E-07 | 90.32 | 28 | 4 |
| 3000795 | Escherichia coli O157:H7 str. Sakai | NC_002695.1.915750.p01 | ECs4393 | 3.0E-11 | 100 | 32 | 2 |
| 3000795 | Escherichia coli O157:H7 str. Sakai | NC_002695.1.916587.p01 | ECs2885 | 2.0E-07 | 96.43 | 27 | 3 |
| 3000800 | Pseudomonas aeruginosa PAO1 | NC_002516.2.881078.p01 | mexC | 3.0E-08 | 90.91 | 32 | 7 |
| 3000801 | Pseudomonas aeruginosa PAO1 | NC_002516.2.881071.p01 | mexD | 7.0E-09 | 90.91 | 30 | 4 |
| 3000801 | Pseudomonas aeruginosa | U57969.gene.p01 | mexD | 5.0E-08 | 90 | 30 | 5 |
| 3000804 | Pseudomonas aeruginosa PAO1 | NC_002516.2.882884.p01 | mexF | 3.0E-06 | 90.91 | 25 | 6 |
| 3000814 | Acinetobacter baumannii AB307-0294 | NC_011595.7059912.p01 | ABBFA_002603 | 8.0E-07 | 90.91 | 25 | 28 |
| 3000814 | Pseudomonas aeruginosa PAO1 | NC_002516.2.880417.p01 | mexT | 7.0E-11 | 90.62 | 32 | 1 |
| 3000816 | Mycobacterium tuberculosis CDC1551 | AE000516.2.gene3505.p01 | mtrA | 8.0E-13 | 100 | 33 | 1 |
| 3000819 | Pseudomonas aeruginosa PAO1 | NC_002516.2.880183.p01 | nalD | 2.0E-11 | 90.91 | 33 | 1 |
| 3000828 | Acinetobacter baumannii AB307-0294 | NC_011595.7057856.p01 | ABBFA_000579 | 1.0E-07 | 90.91 | 25 | 6 |
| 3000828 | Acinetobacter baumannii SDF | NC_010400.5986590.p01 | baeR | 1.0E-08 | 90.62 | 25 | 8 |
| 3000828 | Escherichia coli O157:H7 str. Sakai | NC_002695.1.916589.p01 | ECs2887 | 1.0E-12 | 100 | 33 | 1 |
| 3000829 | Shigella dysenteriae Sd197 | CP000034.1.gene2187.p01 | baeS | 1.0E-12 | 96.97 | 33 | 1 |
| 3000829 | Acinetobacter baumannii AB307-0294 | NC_011595.7057524.p01 | ABBFA_000578 | 1.0E-09 | 90.91 | 33 | 5 |
| 3000829 | Acinetobacter baumannii SDF | NC_010400.5984250.p01 | baeS | 3.0E-10 | 93.33 | 30 | 2 |
| 3000829 | Escherichia coli O157:H7 str. Sakai | NC_002695.1.916588.p01 | ECs2886 | 2.0E-11 | 100 | 33 | 2 |
| 3000829 | Salmonella enterica subsp. enterica serovar Agona str. SL483 | CP001138.1.gene2238.p01 | SeAg_B2260 | 4.0E-12 | 100 | 33 | 1 |
| 3000830 | Escherichia coli O157:H7 str. Sakai | NC_002695.1.914983.p01 | cpxA | 2.0E-10 | 100 | 32 | 6 |
| 3000831 | Enterobacter cloacae subsp. cloacae ATCC 13047 | CP001918.1.gene5135.p01 | ECL_05064 | 8.0E-12 | 100 | 33 | 1 |
| 3000831 | Escherichia coli O157:H7 str. Sakai | NC_002695.1.915041.p01 | ECs4838 | 1.0E-09 | 100 | 27 | 3 |
| 3000833 | Shigella dysenteriae Sd197 | CP000034.1.gene4478.p01 | evgS | 1.0E-06 | 96 | 25 | 4 |
| 3000833 | Escherichia coli O157:H7 str. Sakai | NC_002695.1.915650.p01 | ECs3249 | 4.0E-11 | 96.97 | 33 | 3 |
| 3000834 | Klebsiella pneumoniae subsp. pneumoniae MGH 78578 | CP000647.1.gene2625.p01 | yejM | 2.0E-12 | 100 | 33 | 1 |
| 3000834 | Listeria monocytogenes | HE999704.1.gene2815.p01 | BN418_2960 | 4.0E-09 | 90 | 30 | 2 |
| 3000834 | Staphylococcus aureus subsp. aureus N315 | NC_002745.1124361.p01 | phoP | 2.0E-06 | 92 | 25 | 2 |
| 3000834 | Streptococcus pneumoniae Taiwan19F-14 | NC_012469.1.7685629.p01 | SPT_1001 | 2.0E-07 | 96 | 25 | 1 |
| 3000835 | Escherichia coli O157:H7 str. Sakai | NC_002695.1.913289.p01 | ECs1602 | 3.0E-12 | 100 | 33 | 2 |
| 3000836 | Escherichia coli O157:H7 str. Sakai | NC_002695.1.914292.p01 | ECs5045 | 2.0E-11 | 100 | 33 | 4 |
| 3000837 | Escherichia coli O157:H7 str. Sakai | NC_002695.1.917670.p01 | ECs0930 | 5.0E-08 | 93.33 | 30 | 1 |
| 3000838 | Listeria monocytogenes | HE999704.1.gene1528.p01 | BN418_1620 | 2.0E-09 | 90.91 | 31 | 3 |
| 3000942 | Escherichia coli | AF190694.1.gene1.p01 | blaTEM-76 | 4.0E-13 | 100 | 33 | 1 |
| 3000944 | Escherichia coli | AF190693.1.gene1.p01 | blaTEM-78 | 2.0E-12 | 100 | 33 | 1 |
| 3001168 | Klebsiella pneumoniae | GQ428198.1.gene1.p01 | blaSHV-121 | 3.0E-12 | 100 | 33 | 1 |
| 3001211 | Escherichia coli | DQ464881.1.gene4.p01 | strA | 8.0E-08 | 100 | 27 | 3 |
| 3001211 | Pseudomonas aeruginosa | AF024602.1.gene5.p01 | strA | 2.0E-11 | 96.97 | 32 | 6 |
| 3001212 | Acinetobacter baumannii AYE | NC_010410.6003392.p01 | strB | 2.0E-11 | 100 | 33 | 1 |
| 3001212 | Escherichia coli | DQ464881.1.gene5.p01 | strB | 2.0E-11 | 100 | 31 | 4 |
| 3001213 | Acinetobacter baumannii AYE | NC_010410.6003949.p01 | ABAYE3640 | 5.0E-10 | 93.94 | 33 | 4 |
| 3001214 | Salmonella enterica subsp. enterica serovar Agona str. SL483 | CP001138.1.gene4809.p01 | SeAg_B4848 | 4.0E-10 | 93.94 | 33 | 1 |
| 3001214 | Salmonella enterica subsp. enterica serovar Agona str. SL483 | CP001138.1.gene894.p01 | SeAg_B0902 | 1.0E-10 | 93.94 | 33 | 1 |
| 3001215 | Escherichia coli O157:H7 str. Sakai | NC_002695.1.915390.p01 | ECs4647 | 7.0E-10 | 93.94 | 33 | 3 |
| 3001215 | Salmonella enterica subsp. enterica serovar Agona str. SL483 | CP001138.1.gene4042.p01 | SeAg_B4071 | 1.0E-07 | 92.86 | 28 | 1 |

**Matched contigs of sample C1755 against CARD**

(Sorted by ARO-number)

| **ARO** | **Organism** | **Accession number** | **Gene** | **E value ≤** | **Identity (%) ≥** | **Hit length (aa) ≥** | **Number of contigs** |
| --- | --- | --- | --- | --- | --- | --- | --- |
| 3000017 | uncultured bacterium | AY139598.1.gene3.p01 | blaZ | 8.0E-131 | 96.26 | 187 | 1 |
| 3000017 | Pseudomonas aeruginosa | U59183.1.gene3.p01 | blaOXA-13 | 3.0E-95 | 100 | 136 | 1 |
| 3000017 | Pseudomonas aeruginosa | AF231133.1.gene3.p01 | blaOXA-28 | 1.0E-77 | 99.23 | 130 | 1 |
| 3000194 | Bifidobacterium longum subsp. longum F8 | DQ294299.gene.p01 | tetW_2 | 3.0E-113 | 100 | 169 | 1 |
| 3000194 | Butyrivibrio fibrisolvens | AJ222769.gene.p01 | tetW* | 3.0E-37 | 100 | 67 | 1 |
| 3000196 | Clostridiaceae bacterium K10 | AJ295238.gene.p01 | tet32 | 2.0E-15 | 97.37 | 38 | 3 |
| 3000210 | Acinetobacter baumannii ATCC 17978 | NC_009085.4918494.p01 | rpoB | 4.0E-54 | 93.98 | 122 | 2 |
| 3000210 | Enterococcus faecium DO | CP003583.1.gene2745.p01 | rpoB | 5.0E-41 | 95.45 | 88 | 1 |
| 3000232 | Escherichia coli 1520 | NC_010558.1.6275994.p01 | AadA4 | < 1.0E-150 | 100 | 242 | 1 |
| 3000275 | Enterococcus faecium | AF110130.1.orf0.gene.p01 | linB | 2.0E-42 | 97.26 | 73 | 2 |
| 3000375 | Streptococcus pneumoniae | AM410044.gene14.p01 | ermB | 7.0E-161 | 99.18 | 245 | 1 |
| 3000410 | Pseudomonas aeruginosa | AF191564.1.gene5.p01 | sul1 | 5.0E-43 | 100 | 73 | 1 |
| 3000457 | Acinetobacter baumannii SDF | NC_010400.5986295.p01 | parE | 3.0E-80 | 96.03 | 126 | 1 |
| 3000518 | Proteus mirabilis BB2000 | CP004022.1.gene2827.p01 | crp | 4.0E-44 | 100 | 69 | 1 |
| 3000533 | Azoarcus sp. BH72 | NC_008702.1.4609191.p01 | macA | 6.0E-15 | 91.67 | 36 | 1 |
| 3000566 | Acinetobacter sp. LUH5605 | AY743590.gene.p01 | tet39 | 5.0E-84 | 99.24 | 132 | 2 |
| 3000616 | Streptococcus pneumoniae Taiwan19F-14 | NC_012469.1.7685970.p01 | SPT_1925 | 2.0E-97 | 91.81 | 171 | 1 |
| 3000618 | Azoarcus sp. BH72 | NC_008702.1.4606680.p01 | gyrA | < 1.0E-150 | 97.53 | 81 | 1 |
| 3000618 | Enterococcus faecalis V583 | AE016830.1.gene6.p01 | gyrA | 6.0E-67 | 91.3 | 115 | 1 |
| 3000619 | Acinetobacter baumannii SDF | NC_010400.5984045.p01 | parC | 3.0E-50 | 98.7 | 77 | 1 |
| 3000621 | Salmonella enterica subsp. enterica serovar Typhimurium | X07260.1.gene1.p01 | blaZ | 2.0E-124 | 100 | 173 | 1 |
| 3000814 | Acinetobacter baumannii AB307-0294 | NC_011595.7059912.p01 | mexT | 5.0E-83 | 93.5 | 123 | 1 |
| 3000829 | Acinetobacter baumannii AYE | NC_010410.6000672.p01 | baeS | 8.0E-22 | 95.12 | 41 | 1 |
| 3001211 | Escherichia coli | DQ464881.1.gene4.p01 | strA | 5.0E-41 | 98.53 | 68 | 1 |

**Matched high-throughput sequencing reads of sample C1756 against CARD**

(Sorted by ARO-number)

| **ARO** | **Organism** | **Accession number** | **Gene** | **E value ≤** | **Identity (%) ≥** | **Hit length (aa) ≥** | **Number of reads** |
| --- | --- | --- | --- | --- | --- | --- | --- |
| 3000002 | *Enterococcus faecalis* | AF253562.2.orf3.gene.p01 | vanWG | 6.0E-11 | 90.91 | 33 | 3 |
| 3000014 | *Escherichia coli* | AF397067.1.gene1.p1 | AAK85244.1 | 1.0E-13 | 100 | 33 | 1 |
| 3000014 | *Escherichia coli* | X57972.1.gene1.p1 | CAA41038.1 | 2.0E-13 | 100 | 33 | 1 |
| 3000017 | *Pseudomonas aeruginosa* | U59183.1.gene3.p01 | bla oxa-13 | 2.0E-13 | 100 | 33 | 2 |
| 3000017 | *Acinetobacter baumannii* | AY750910.1.gene1.p01 | bla-oxa-68 | 3.0E-12 | 93.75 | 32 | 1 |
| 3000017 | *Acinetobacter baumannii* | DQ519090.1.gene1.p01 | blaOXA-96 | 5.0E-13 | 100 | 31 | 1 |
| 3000017 | *Acinetobacter baumannii* | Y10693.2.gene1.p2 | CAA71699.2 | 3.0E-15 | 100 | 33 | 1 |
| 3000017 | *Burkholderia cepacia* | AF371964.1.gene1.p1 | AAK55330.1 | 5.0E-10 | 100 | 27 | 1 |
| 3000017 | *Klebsiella pneumoniae* | M55547.gene.p01 | blaOXA-9 | 8.0E-15 | 100 | 33 | 1 |
| 3000017 | *Pseudomonas aeruginosa* | AF350424.1.gene1.p01 | bla oxa-34 | 3.0E-15 | 100 | 33 | 1 |
| 3000017 | *Pseudomonas aeruginosa* | AJ854182.1.gene1.p01 | blaOXA-74 | 9.0E-13 | 100 | 33 | 1 |
| 3000017 | *Pseudomonas aeruginosa* | U37105.2.gene2.p01 | oxa10 | 9.0E-13 | 100 | 33 | 5 |
| 3000017 | *Pseudomonas aeruginosa* | U63835.1.gene1.p01 | blaOXA-15 | 2.0E-13 | 100 | 33 | 1 |
| 3000017 | *Pseudomonas aeruginosa* | JF800667.1.gene2.p01 | blaOXA-205 | 4.0E-15 | 100 | 33 | 1 |
| 3000019 | *Escherichia coli O157:H7 str. Sakai* | NC_002695.1.914045.p01 | ampC | 3.0E-08 | 90.91 | 25 | 28 |
| 3000019 | *Enterobacter cloacae subsp. cloacae ATCC 13047* | CP001918.1.gene3289.p01 | ampC | 2.0E-12 | 90.91 | 33 | 1 |
| 3000019 | *Enterococcus faecium DO* | CP003583.1.gene2367.p01 | ampC | 3.0E-09 | 96.97 | 27 | 10 |
| 3000019 | *Shigella dysenteriae Sd197* | CP000034.1.gene4394.p01 | ampC | 1.0E-08 | 100 | 25 | 3 |
| 3000027 | *Acinetobacter baumannii AYE* | NC_010410.6003177.p01 | emrA | 1.0E-08 | 90.32 | 28 | 4 |
| 3000027 | *Escherichia coli O157:H7 str. Sakai* | NC_002695.1.914737.p01 | ECs3547 | 4.0E-06 | 100 | 26 | 28 |
| 3000027 | *Klebsiella pneumoniae subsp. pneumoniae MGH 78578* | CP000647.1.gene3014.p01 | emrA | 2.0E-11 | 96.97 | 31 | 11 |
| 3000027 | *Salmonella enterica subsp. enterica serovar Agona str. SL483* | CP001138.1.gene2905.p01 | emrA | 5.0E-12 | 96.97 | 33 | 3 |
| 3000027 | *Shigella dysenteriae Sd197* | CP000034.1.gene2880.p01 | emrA | 4.0E-06 | 96.97 | 25 | 13 |
| 3000054 | *Klebsiella pneumoniae subsp. pneumoniae MGH 78578* | CP000647.1.gene444.p01 | acrA | 1.0E-07 | 93.94 | 25 | 28 |
| 3000054 | *Escherichia coli O157:H7 str. Sakai* | NC_002695.1.914620.p01 | ECs0516 | 1.0E-07 | 96.77 | 26 | 33 |
| 3000066 | *Klebsiella pneumoniae* | AY494718.1.gene1.p01 | blaGES-4 | 4.0E-13 | 100 | 33 | 1 |
| 3000066 | *Pseudomonas aeruginosa* | AF326355.1.gene1.p01 | blaGES-2 | 2.0E-14 | 100 | 33 | 1 |
| 3000066 | *Pseudomonas aeruginosa* | GU208678.1.gene1.p01 | blaGES-15 | 2.0E-12 | 96.97 | 33 | 2 |
| 3000071 | *Clostridium difficile 630* | AM180355.1.gene1831.p01 | vanS | 1.0E-08 | 90.32 | 31 | 9 |
| 3000071 | *Enterococcus faecalis* | DQ212986.1.gene5.p01 | vanSG | 3.0E-08 | 90.62 | 27 | 2 |
| 3000071 | *Enterococcus faecium* | AF130997.1.orf1.gene.p01 | vanSD | 9.0E-08 | 90.91 | 26 | 2 |
| 3000071 | *Enterococcus faecium* | AF310956.2.orf1.gene.p01 | vanSB2 | 2.0E-11 | 100 | 30 | 1 |
| 3000071 | *Enterococcus gallinarum* | AF162694.1.orf5.gene.p01 | vanSc | 2.0E-12 | 96.97 | 33 | 2 |
| 3000072 | *Enterobacter cloacae subsp. cloacae ATCC 13047* | CP001918.1.gene4505.p01 | cdiA | 3.0E-08 | 90.91 | 33 | 2 |
| 3000073 | *Hafnia alvei* | AF180958.1.gene1.p01 | acc-3 | 3.0E-13 | 100 | 33 | 1 |
| 3000074 | *Acinetobacter baumannii AYE* | NC_010410.6003262.p01 | emrB | 1.0E-10 | 90.91 | 33 | 2 |
| 3000074 | *Enterobacter cloacae subsp. cloacae ATCC 13047* | CP001918.1.gene4071.p01 | ECL_04023 | 4.0E-10 | 93.94 | 33 | 3 |
| 3000074 | *Enterococcus faecalis V583* | AE016830.1.gene1357.p01 | EF_1370 | 2.0E-11 | 93.94 | 33 | 2 |
| 3000074 | *Enterococcus faecalis V583* | AE016830.1.gene424.p01 | EF_0420 | 1.0E-08 | 90.91 | 28 | 5 |
| 3000074 | *Enterococcus faecalis V583* | AE016830.1.gene768.p01 | EF_0785 | 8.0E-11 | 90.91 | 33 | 2 |
| 3000074 | *Enterococcus faecium DO* | CP003583.1.gene510.p01 | yniG | 5.0E-11 | 96.97 | 33 | 4 |
| 3000074 | *Escherichia coli O157:H7 str. Sakai* | NC_002695.1.914736.p01 | ECs3548 | 2.0E-11 | 96.97 | 33 | 32 |
| 3000074 | *Klebsiella pneumoniae subsp. pneumoniae MGH 78578* | CP000647.1.gene3015.p01 | emrB | 2.0E-07 | 96.97 | 25 | 13 |
| 3000074 | *Proteus mirabilis BB2000* | CP004022.1.gene546.p01 | emrB | 3.0E-09 | 90.32 | 31 | 1 |
| 3000074 | *Salmonella enterica subsp. enterica serovar Agona str. SL483* | CP001138.1.gene2906.p01 | emrB | 2.0E-06 | 90.91 | 25 | 34 |
| 3000074 | *Staphylococcus aureus subsp. aureus COL* | NC_002951.3237419.p01 | SACOL2157 | 5.0E-10 | 96.55 | 29 | 1 |
| 3000074 | *Staphylococcus aureus subsp. aureus MRSA252* | NC_002952.2859362.p01 | SAR2437 | 3.0E-11 | 90.91 | 33 | 1 |
| 3000077 | *Enterococcus faecalis* | AF253562.2.orf2.gene.p01 | vanYG1 | 6.0E-13 | 93.94 | 33 | 1 |
| 3000077 | *Enterococcus faecalis* | DQ212986.1.gene6.p01 | vanYG | 1.0E-13 | 90.91 | 33 | 1 |
| 3000083 | *Aeromonas caviae* | GQ152600.1.gene1.p01 | blaMOX-5 | 4.0E-08 | 92.59 | 27 | 1 |
| 3000091 | *Acinetobacter calcoaceticus subsp. anitratus* | AF135373.1.gene1.p01 | blaCARB-5 | 4.0E-10 | 100 | 29 | 2 |
| 3000092 | *Pseudomonas aeruginosa* | Z22590.1.gene1.p01 | blaZ | 2.0E-15 | 100 | 33 | 1 |
| 3000116 | *Enterococcus faecium DO* | CP003583.1.gene1365.p01 | HMPREF0351_11334 | 1.0E-11 | 100 | 32 | 4 |
| 3000116 | *Enterococcus faecium DO* | CP003583.1.gene22.p01 | vanZ | 4.0E-12 | 96.97 | 33 | 3 |
| 3000122 | *Acinetobacter baumannii AB307-0294* | NC_011595.7057747.p01 | ABBFA_002299 | 8.0E-09 | 90.62 | 25 | 8 |
| 3000122 | *Acinetobacter baumannii AYE* | NC_010410.6000796.p01 | cat | 2.0E-15 | 90.91 | 33 | 5 |
| 3000122 | *Escherichia coli 1520* | NC_010558.1.6276004.p01 | CatA1 | 2.0E-13 | 96.97 | 33 | 13 |
| 3000122 | *Klebsiella pneumoniae subsp. pneumoniae MGH 78578* | CP000647.1.gene2018.p01 | KPN_02018 | 5.0E-14 | 100 | 33 | 8 |
| 3000126 | *Enterococcus faecalis* | V01547.1.orf0.gene.p01 | V01547.1.orf0 | 7.0E-08 | 100 | 25 | 44 |
| 3000126 | *Escherichia coli* | V00359.1.orf1.gene.p01 | V00359.1.orf1 | 2.0E-09 | 100 | 25 | 14 |
| 3000151 | *Streptomyces glaucescens* | X05648.1.orf1.gene.p01 | X05648.1.orf1 | 2.0E-13 | 90.91 | 33 | 1 |
| 3000165 | *Acinetobacter baumannii AB0057* | NC_011586.7045189.p01 | tetA | 4.0E-11 | 96.97 | 33 | 2 |
| 3000165 | *Pseudomonas aeruginosa* | X75761.gene.p01 | tetA | 8.0E-10 | 100 | 29 | 3 |
| 3000165 | *Shigella sonnei* | AF534183.gene.p01 | tetA | 2.0E-12 | 100 | 33 | 2 |
| 3000167 | *Escherichia coli* | Y19114.gene.p01 | tetC | 1.0E-06 | 100 | 28 | 2 |
| 3000168 | *Plasmid pRA1* | L06798.gene.p01 | tetD | 6.0E-07 | 100 | 25 | 4 |
| 3000168 | *Salmonella enterica subsp. enterica serovar Agona str. SL483* | CP001138.1.gene4488.p01 | SeAg_B4524 | 2.0E-15 | 100 | 33 | 1 |
| 3000168 | *Salmonella enterica subsp. enterica serovar Agona str. SL483* | CP001138.1.gene612.p01 | SeAg_B0620 | 9.0E-09 | 93.94 | 26 | 4 |
| 3000174 | *Pseudomonas sp.* | AF133139.gene.p01 | tetG | 5.0E-07 | 90.91 | 26 | 14 |
| 3000174 | *Pseudomonas sp.* | AF133140.gene.p01 | tetG | 1.0E-11 | 93.94 | 33 | 3 |
| 3000179 | *Geobacillus stearothermophilus* | M11036.gene.p01 | tetL* | 3.0E-10 | 96.97 | 29 | 5 |
| 3000186 | *Clostridium difficile 630* | AM180355.1.gene636.p01 | tetM | 1.0E-06 | 90.91 | 26 | 10 |
| 3000186 | *Enterococcus faecalis* | M85225.gene.p01 | tetM | 3.0E-07 | 90.62 | 26 | 96 |
| 3000186 | *Enterococcus faecalis* | X04388.gene.p01 | tetM | 4.0E-09 | 90.91 | 27 | 92 |
| 3000186 | *Staphylococcus aureus subsp. aureus Mu50* | NC_002758.1120355.p01 | tetM | 8.0E-11 | 96.97 | 32 | 12 |
| 3000190 | *Megasphaera elsdenii* | AY485126.gene.p01 | tetOW | 3.0E-06 | 90.62 | 25 | 314 |
| 3000191 | *Bacteroides fragilis* | Z21523.gene.p01 | tetQ | 1.0E-06 | 90.91 | 25 | 59 |
| 3000192 | *Listeria monocytogenes* | L09756.gene.p01 | tetS* | 4.0E-11 | 90.32 | 31 | 1 |
| 3000193 | *Streptococcus pyogenes* | L42544.gene.p01 | tetT* | 4.0E-10 | 93.94 | 33 | 6 |
| 3000194 | *Bifidobacterium longum subsp. longum F8* | DQ294299.gene.p01 | tetW_2 | 8.0E-07 | 90.91 | 25 | 348 |
| 3000194 | *Butyrivibrio fibrisolvens* | AJ222769.gene.p01 | tetW* | 4.0E-06 | 90 | 25 | 1245 |
| 3000195 | *Clostridium perfringens* | L20800.gene.p01 | tetBP | 2.0E-09 | 90.91 | 30 | 17 |
| 3000196 | *Clostridiaceae bacterium K10* | AJ295238.gene.p01 | tet32 | 2.0E-06 | 90.32 | 25 | 1153 |
| 3000197 | *Bacteroides coprosuis DSM 18011* | AJ514254.gene.p01 | tet36 | 9.0E-12 | 96.97 | 33 | 1 |
| 3000205 | *Bacteroides fragilis* | M37699.gene1.p01 | tetX | 1.0E-12 | 100 | 33 | 2 |
| 3000207 | *Enterobacter cloacae subsp. cloacae ATCC 13047* | CP001918.1.gene1258.p01 | acrA | 1.0E-08 | 96.97 | 27 | 3 |
| 3000207 | *Escherichia coli O157:H7 str. Sakai* | NC_002695.1.912781.p01 | ECs1863 | 2.0E-06 | 90.91 | 26 | 7 |
| 3000210 | *Acinetobacter baumannii ATCC 17978* | NC_009085.4918494.p01 | rpoB | 4.0E-06 | 90.62 | 25 | 119 |
| 3000210 | *Acinetobacter baumannii AYE* | NC_010410.6003841.p01 | rpoB | 6.0E-11 | 100 | 33 | 1 |
| 3000210 | *Acinetobacter baumannii SDF* | NC_010400.5987325.p01 | rpoB | 9.0E-08 | 93.94 | 25 | 13 |
| 3000210 | *Azoarcus sp. BH72* | NC_008702.1.4609796.p01 | rpoB | 2.0E-06 | 90.32 | 25 | 87 |
| 3000210 | *Bordetella pertussis CS* | CP002695.1.gene18.p01 | rpoB | 1.0E-06 | 90 | 25 | 236 |
| 3000210 | *Clostridium botulinum A2 str. Kyoto* | CP001581.1.gene3846.p01 | rpoB | 3.0E-06 | 90 | 26 | 772 |
| 3000210 | *Clostridium difficile 630* | AM180355.1.gene120.p01 | rpoB | 9.0E-06 | 90 | 25 | 645 |
| 3000210 | *Enterobacter cloacae subsp. cloacae ATCC 13047* | CP001918.1.gene250.p01 | rpoB | 5.0E-11 | 93.94 | 33 | 14 |
| 3000210 | *Enterococcus faecalis V583* | AE016830.1.gene3155.p01 | rpoB | 4.0E-06 | 90 | 25 | 458 |
| 3000210 | *Enterococcus faecium DO* | CP003583.1.gene2745.p01 | rpoB | 1.0E-06 | 90 | 25 | 116 |
| 3000210 | *Escherichia coli O157:H7 str. Sakai* | NC_002695.1.914942.p01 | rpoB | 1.0E-07 | 90.91 | 28 | 76 |
| 3000210 | *Klebsiella pneumoniae subsp. pneumoniae MGH 78578* | CP000647.1.gene4402.p01 | rpoB | 5.0E-08 | 93.94 | 29 | 28 |
| 3000210 | *Legionella pneumophila str. Corby* | CP000675.2.gene392.p01 | rpoB | 1.0E-06 | 90 | 26 | 88 |
| 3000210 | *Mycobacterium tuberculosis CDC1551* | AE000516.2.gene708.p01 | rpoB | 4.0E-06 | 90 | 25 | 410 |
| 3000210 | *Neisseria gonorrhoeae NCCP11945* | NC_011035.1.6448762.p01 | rpoB | 1.0E-06 | 90 | 25 | 94 |
| 3000210 | *Proteus mirabilis BB2000* | CP004022.1.gene2794.p01 | rpoB | 2.0E-07 | 90.91 | 26 | 18 |
| 3000210 | *Pseudomonas aeruginosa PAO1* | NC_002516.2.881699.p01 | rpoB | 3.0E-06 | 90.62 | 25 | 60 |
| 3000210 | *Salmonella enterica subsp. enterica serovar Agona str. SL483* | CP001138.1.gene4362.p01 | rpoB | 3.0E-06 | 90 | 25 | 168 |
| 3000210 | *Shigella dysenteriae Sd197* | CP000034.1.gene3741.p01 | rpoB | 9.0E-11 | 100 | 33 | 4 |
| 3000210 | *Staphylococcus aureus subsp. aureus COL* | NC_002951.3236234.p01 | rpoB | 1.0E-07 | 90.32 | 31 | 15 |
| 3000210 | *Staphylococcus aureus subsp. aureus ED98* | NC_013450.8613267.p01 | rpoB | 7.0E-11 | 90.91 | 33 | 1 |
| 3000210 | *Staphylococcus aureus subsp. aureus JH9* | NC_009487.5169226.p01 | rpoB | 3.0E-08 | 90.32 | 31 | 7 |
| 3000210 | *Staphylococcus aureus subsp. aureus MRSA252* | NC_002952.2860169.p01 | rpoB | 2.0E-09 | 90.62 | 32 | 6 |
| 3000210 | *Staphylococcus aureus subsp. aureus Mu50* | NC_002758.1120515.p01 | rpoB | 2.0E-06 | 90.62 | 26 | 27 |
| 3000210 | *Staphylococcus aureus subsp. aureus N315* | NC_002745.1123305.p01 | rpoB | 4.0E-06 | 90.32 | 26 | 511 |
| 3000210 | *Streptococcus pneumoniae Taiwan19F-14* | NC_012469.1.7686402.p01 | rpoB | 4.0E-06 | 90 | 25 | 409 |
| 3000216 | *Shigella dysenteriae Sd197* | CP000034.1.gene457.p01 | acrB | 4.0E-07 | 90.62 | 28 | 29 |
| 3000216 | *Klebsiella pneumoniae subsp. pneumoniae MGH 78578* | CP000647.1.gene443.p01 | acrB | 3.0E-06 | 90.62 | 25 | 52 |
| 3000216 | *Escherichia coli O157:H7 str. Saka* | NC_002695.1.914619.p01 | ECs0515 | 6.0E-06 | 90.91 | 25 | 113 |
| 3000216 | *Enterobacter cloacae subsp. cloacae ATCC 13047* | CP001918.1.gene1257.p01 | acrB | 3.0E-06 | 90.62 | 28 | 18 |
| 3000216 | *Escherichia coli O157:H7 str. Sakai* | NC_002695.1.912777.p01 | ECs1864 | 2.0E-06 | 90.91 | 25 | 63 |
| 3000216 | *Bordetella pertussis CS* | CP002695.1.gene992.p01 | acrB | 8.0E-07 | 92.86 | 28 | 5 |
| 3000217 | *Staphylococcus aureus subsp. aureus JH1* | NC_009619.5314975.p01 | SaurJH1_2825 | 1.0E-12 | 100 | 33 | 3 |
| 3000225 | *Campylobacter fetus subsp. fetus* | FN594949.1.gene25.p01 | ant(6)-Ib | 1.0E-07 | 90.62 | 25 | 27 |
| 3000225 | *Campylobacter jejuni* | AJ489618.1.orf2.gene.p01 | aadE | 5.0E-10 | 90.62 | 25 | 2 |
| 3000225 | *Staphylococcus epidermidis RP62A* | NC_006663.1.orf0.gene.p01 | aadE | 5.0E-12 | 96.77 | 31 | 5 |
| 3000225 | *Streptococcus pneumoniae* | AM410044.gene9.p01 | aadE | 5.0E-14 | 100 | 32 | 3 |
| 3000226 | *Acinetobacter baumannii AB0057* | NC_011586.7045179.p01 | folP | 1.0E-07 | 96.88 | 26 | 4 |
| 3000226 | *Escherichia coli O157:H7 str. Sakai* | NC_002695.1.916103.p01 | folP | 8.0E-08 | 92.31 | 25 | 32 |
| 3000226 | *Klebsiella pneumoniae subsp. pneumoniae MGH 78578* | CP000647.1.gene3624.p01 | folP | 1.0E-11 | 96.97 | 30 | 16 |
| 3000226 | *Salmonella enterica subsp. enterica serovar Agona str. SL483* | CP001138.1.gene3456.p01 | folP | 1.0E-06 | 90.91 | 25 | 3 |
| 3000226 | *Shigella dysenteriae Sd197* | CP000034.1.gene3358.p01 | folP | 3.0E-06 | 92.31 | 25 | 8 |
| 3000230 | *Pseudomonas aeruginosa* | U37105.2.gene3.p01 | aadB | 3.0E-13 | 96.97 | 33 | 1 |
| 3000232 | *Acinetobacter baumannii AYE* | NC_010410.6002671.p01 | ABAYE3739 | 5.0E-15 | 100 | 33 | 1 |
| 3000232 | *Acinetobacter baumannii AYE* | NC_010410.6003170.p01 | aadA1 | 1.0E-07 | 96.43 | 28 | 1 |
| 3000232 | *Acinetobacter baumannii SDF* | NC_010400.5986843.p01 | ABSDF0145 | 2.0E-14 | 100 | 33 | 2 |
| 3000232 | *Escherichia coli 1520* | NC_010558.1.6275994.p01 | AadA4 | 6.0E-07 | 100 | 25 | 8 |
| 3000232 | *Klebsiella pneumoniae* | AJ704863.gene12.p01 | aadA1 | 1.0E-11 | 100 | 30 | 6 |
| 3000232 | *Plasmid NR79* | AF047479.2.orf1.gene.p01 | aadA3 | 6.0E-14 | 100 | 33 | 1 |
| 3000232 | *Pseudomonas aeruginosa* | AF294653.1.gene3.p01 | AadA2a | 1.0E-11 | 90.91 | 33 | 2 |
| 3000232 | *Pseudomonas aeruginosa* | AJ584652.2.gene7.p01 | aadA1 | 1.0E-11 | 93.55 | 31 | 1 |
| 3000232 | *Pseudomonas aeruginosa* | U37105.2.gene4.p01 | aadA10 | 9.0E-11 | 90.91 | 32 | 15 |
| 3000232 | *Salmonella enterica subsp. enterica serovar Typhi* | AY123251.gene3.p01 | aadA1 | 1.0E-12 | 96.97 | 33 | 7 |
| 3000232 | *Salmonella enterica* | AJ628353.gene.p01 | aadA1b | 3.0E-12 | 93.94 | 33 | 11 |
| 3000232 | *uncultured bacterium* | AY139598.1.gene2.p01 | aadA5 | 3.0E-14 | 100 | 33 | 2 |
| 3000235 | *Mycobacterium tuberculosis CDC1551* | AE000516.2.gene4101.p01 | embB | 5.0E-08 | 90.32 | 31 | 2 |
| 3000237 | *Acinetobacter baumannii AB307-0294* | NC_011595.7058144.p01 | ABBFA_003277 | 1.0E-09 | 90.91 | 33 | 1 |
| 3000237 | *Escherichia coli O157:H7 str. Sakai* | NC_002695.1.916248.p01 | tolC | 2.0E-06 | 93.75 | 25 | 37 |
| 3000237 | *Klebsiella pneumoniae subsp. pneumoniae MGH 78578* | CP000647.1.gene3449.p01 | tolC | 1.0E-10 | 96.97 | 31 | 11 |
| 3000237 | *Salmonella enterica subsp. enterica serovar Agona str. SL483* | CP001138.1.gene3341.p01 | SeAg_B3367 | 2.0E-08 | 93.94 | 27 | 10 |
| 3000237 | *Shigella dysenteriae Sd197* | CP000034.1.gene3205.p01 | tolC | 3.0E-12 | 100 | 33 | 6 |
| 3000245 | *Mycobacterium smegmatis str. MC2 155* | HQ203032.1.gene1.p01 | rbpA | 5.0E-09 | 92.59 | 27 | 1 |
| 3000252 | *Enterobacter aerogenes* | AF335467.1.gene1.p01 | omp36 | 6.0E-12 | 90.91 | 32 | 5 |
| 3000252 | *Enterobacter aerogenes* | AF336095.1.gene1.p01 | omp36 | 5.0E-06 | 90.62 | 25 | 15 |
| 3000252 | *Enterobacter aerogenes* | AF336096.1.gene1.p01 | omp36 | 2.0E-09 | 93.94 | 33 | 3 |
| 3000252 | *Enterobacter aerogenes* | AF336097.1.gene1.p01 | omp36 | 5.0E-11 | 90.91 | 33 | 1 |
| 3000258 | *Enterococcus faecium DO* | CP003583.1.gene212.p01 | ermC | 6.0E-12 | 93.94 | 32 | 12 |
| 3000263 | *Escherichia coli O157:H7 str. Sakai* | NC_002695.1.917339.p01 | ECs2138 | 8.0E-12 | 100 | 28 | 6 |
| 3000263 | *Klebsiella pneumoniae subsp. pneumoniae MGH 78578* | CP000647.1.gene1624.p01 | marA | 6.0E-14 | 100 | 33 | 3 |
| 3000263 | *Salmonella enterica subsp. enterica serovar Agona str. SL483* | CP001138.1.gene1637.p01 | SeAg_B1651 | 2.0E-09 | 100 | 25 | 4 |
| 3000263 | *Shigella dysenteriae Sd197* | CP000034.1.gene1596.p01 | marA | 6.0E-14 | 100 | 32 | 1 |
| 3000264 | *Escherichia coli O157:H7 str. Sakai* | NC_002695.1.913273.p01 | emrE | 2.0E-07 | 92 | 25 | 12 |
| 3000272 | *Staphylococcus aureus* | AY541446.1.orf1.gene.p01 | linA | 1.0E-12 | 90.91 | 33 | 1 |
| 3000275 | *Enterococcus faecium* | AF110130.1.orf0.gene.p01 | linB | 1.0E-11 | 96.97 | 32 | 4 |
| 3000300 | *Staphylococcus sciuri* | AJ579365.gene.p01 | lsaB_orf3 | 9.0E-10 | 90.91 | 33 | 1 |
| 3000309 | *Escherichia coli O157:H7 str. Sakai* | NC_002695.1.915420.p01 | emrD | 2.0E-08 | 96.97 | 26 | 22 |
| 3000309 | *Klebsiella pneumoniae subsp. pneumoniae MGH 78578* | CP000647.1.gene4115.p01 | emrD | 8.0E-06 | 93.94 | 25 | 9 |
| 3000309 | *Salmonella enterica subsp. enterica serovar Agona str. SL483* | CP001138.1.gene3995.p01 | emrD | 3.0E-12 | 100 | 30 | 3 |
| 3000316 | *Escherichia coli* | D16251.1.orf0.gene.p01 | mphA | 7.0E-09 | 100 | 27 | 5 |
| 3000322 | *Plasmid pWP14a* | X13542.gene.p01 | aac(3)-III | 2.0E-11 | 93.94 | 33 | 9 |
| 3000345 | *Streptococcus pneumoniae Taiwan19F-14* | NC_012469.1.7685616.p01 | SPT_1105 | 5.0E-16 | 100 | 33 | 1 |
| 3000361 | *Escherichia coli* | AY183453.gene.p01 | ereA3 | 2.0E-10 | 90.91 | 33 | 1 |
| 3000368 | *Enterococcus gallinarum* | AF162694.gene.p01 | vanC* | 1.0E-12 | 100 | 33 | 1 |
| 3000372 | *Enterococcus faecalis* | DQ212986.1.gene11.p01 | vanTG | 1.0E-08 | 100 | 26 | 1 |
| 3000373 | *Escherichia coli O157:H7 str. Sakai* | NC_002695.1.915653.p01 | ECs3247 | 2.0E-09 | 90.62 | 28 | 37 |
| 3000373 | *Shigella dysenteriae Sd197* | CP000034.1.gene2563.p01 | emrK | 6.0E-14 | 100 | 33 | 3 |
| 3000375 | *Clostridium difficile 630* | AM180355.1.gene2254.p01 | ermB | 8.0E-07 | 93.94 | 25 | 63 |
| 3000375 | *Streptococcus pneumoniae* | AM410044.gene14.p01 | ermB | 2.0E-06 | 90.91 | 25 | 177 |
| 3000378 | *Acinetobacter baumannii AB307-0294* | NC_011595.7057907.p01 | ABBFA_000816 | 7.0E-07 | 90.91 | 26 | 14 |
| 3000378 | *Pseudomonas aeruginosa PAO1* | NC_002516.2.877852.p01 | mexB | 7.0E-06 | 90.62 | 27 | 15 |
| 3000379 | *Acinetobacter baumannii AB307-0294* | NC_011595.7058890.p01 | ABBFA_003020 | 8.0E-10 | 90.91 | 30 | 5 |
| 3000390 | *Streptomyces coelicolor A3(2)* | AL939114.1.orf1.gene.p01 | SCO2860 | 1.0E-12 | 90.91 | 33 | 1 |
| 3000391 | *Staphylococcus aureus* | D90119.gene.p01 | NorA | 3.0E-13 | 100 | 33 | 1 |
| 3000410 | *Citrobacter freundii* | AY162283.2.gene7.p01 | sul1 | 5.0E-13 | 100 | 33 | 2 |
| 3000410 | *Klebsiella pneumoniae* | AF322577.2.gene6.p01 | sul1 | 1.0E-13 | 100 | 33 | 1 |
| 3000410 | *Klebsiella pneumoniae* | AJ704863.gene21.p01 | partial sul1 | 3.0E-12 | 100 | 28 | 2 |
| 3000410 | *Pseudomonas aeruginosa* | U37105.2.gene6.p01 | sul1 | 5.0E-07 | 100 | 25 | 7 |
| 3000412 | *Escherichia coli* | DQ464881.1.gene2.p01 | sul2 | 3.0E-08 | 93.33 | 27 | 18 |
| 3000419 | *Klebsiella pneumoniae* | DQ351241.gene.p01 | qnrB | 4.0E-12 | 90.62 | 32 | 2 |
| 3000445 | *Rhodococcus equi* | U56415.1.orf0.gene.p01 | iri | 8.0E-12 | 90.91 | 33 | 3 |
| 3000457 | *Acinetobacter baumannii ACICU* | NC_010611.6235284.p01 | parE | 6.0E-13 | 96.97 | 33 | 1 |
| 3000457 | *Acinetobacter baumannii AYE* | NC_010410.6000336.p01 | parE | 5.0E-09 | 90.91 | 33 | 9 |
| 3000457 | *Acinetobacter baumannii SDF* | NC_010400.5986295.p01 | parE | 4.0E-07 | 90.91 | 25 | 33 |
| 3000457 | *Azoarcus sp. BH72* | NC_008702.1.4609137.p01 | parE | 1.0E-09 | 90.91 | 29 | 8 |
| 3000457 | *Bordetella pertussis CS* | CP002695.1.gene1273.p01 | parE | 8.0E-09 | 90 | 30 | 37 |
| 3000457 | *Clostridium botulinum A2 str. Kyoto* | CP001581.1.gene3049.p01 | CLM_3138 | 6.0E-06 | 90.32 | 25 | 9 |
| 3000457 | *Enterococcus faecalis V583* | AE016830.1.gene1599.p01 | parE | 2.0E-07 | 90 | 25 | 142 |
| 3000457 | *Enterococcus faecium DO* | CP003583.1.gene1173.p01 | parE | 9.0E-07 | 90 | 25 | 65 |
| 3000457 | *Klebsiella pneumoniae subsp. pneumoniae MGH 78578* | CP000647.1.gene3444.p01 | parE | 4.0E-10 | 96.97 | 28 | 23 |
| 3000457 | *Legionella pneumophila str. Corby* | CP000675.2.gene802.p01 | parE | 8.0E-09 | 90 | 30 | 4 |
| 3000457 | *Proteus mirabilis BB2000* | CP004022.1.gene2481.p01 | parE | 6.0E-12 | 90.91 | 33 | 1 |
| 3000457 | *Pseudomonas aeruginosa PAO1* | NC_002516.2.879897.p01 | parE | 5.0E-10 | 90.91 | 30 | 6 |
| 3000457 | *Salmonella enterica subsp. enterica serovar Agona str. SL483* | CP001138.1.gene3336.p01 | parE | 9.0E-09 | 96.97 | 27 | 35 |
| 3000457 | *Shigella dysenteriae Sd197* | CP000034.1.gene3210.p01 | parE | 3.0E-07 | 96.97 | 26 | 37 |
| 3000457 | *Staphylococcus aureus RF122* | NC_007622.3794232.p01 | grlB | 9.0E-08 | 90.62 | 32 | 5 |
| 3000457 | *Staphylococcus aureus subsp. aureus COL* | NC_002951.3236245.p01 | parE | 3.0E-07 | 90.62 | 26 | 14 |
| 3000457 | *Staphylococcus aureus subsp. aureus MRSA252* | NC_002952.2859942.p01 | grlB | 9.0E-07 | 90.32 | 26 | 26 |
| 3000457 | *Staphylococcus aureus subsp. aureus N315* | NC_002745.1124025.p01 | parE | 1.0E-07 | 90 | 25 | 82 |
| 3000457 | *Staphylococcus aureus subsp. aureus str. Newman* | NC_009641.5330756.p01 | parE | 7.0E-12 | 93.94 | 33 | 1 |
| 3000457 | *Streptococcus pneumoniae Taiwan19F-14* | NC_012469.1.7686068.p01 | parE | 9.0E-06 | 90 | 25 | 81 |
| 3000479 | *Streptomyces caeruleus* | AF205854.1.orf0.gene.p01 | gyrB-Rn | 2.0E-08 | 90.32 | 31 | 8 |
| 3000479 | *Streptomyces roseochromogenes subsp. oscitans* | AF329398.1.orf36.gene.p01 | gyrBR | 8.0E-10 | 90.62 | 30 | 10 |
| 3000479 | *Streptomyces roseochromogenes subsp. oscitans* | AY136281.1.orf1.gene.p01 | gyrBR | 1.0E-09 | 90.32 | 31 | 4 |
| 3000480 | *Streptomyces rishiriensis* | AF205853.1.orf1.gene.p01 | parYR | 6.0E-06 | 90.62 | 25 | 27 |
| 3000480 | *Streptomyces roseochromogenes subsp. oscitans* | AY136281.1.orf0.gene.p01 | parYR | 9.0E-08 | 90.62 | 25 | 43 |
| 3000489 | *Staphylococcus aureus subsp. aureus Mu50* | NC_002758.1121879.p01 | SAV1866 | 4.0E-08 | 90 | 30 | 17 |
| 3000491 | *Escherichia coli O157:H7 str. Sakai* | NC_002695.1.915267.p01 | ECs3332 | 6.0E-07 | 90 | 25 | 80 |
| 3000491 | *Klebsiella pneumoniae subsp. pneumoniae MGH 78578* | CP000647.1.gene2803.p01 | acrD | 8.0E-09 | 90.91 | 30 | 36 |
| 3000491 | *Shigella dysenteriae Sd197* | CP000034.1.gene2654.p01 | acrD | 1.0E-10 | 100 | 33 | 30 |
| 3000491 | *Salmonella enterica subsp. enterica serovar Agona str. SL483* | CP001138.1.gene2601.p01 | acrD | 8.0E-06 | 90.91 | 26 | 37 |
| 3000493 | *Azoarcus sp. BH72* | NC_008702.1.4607594.p01 | ompR2 | 3.0E-12 | 90.91 | 33 | 1 |
| 3000493 | *Escherichia coli* | GQ465831.1.gene2.p01 | ompF | 2.0E-07 | 90.91 | 26 | 29 |
| 3000493 | *Klebsiella pneumoniae subsp. pneumoniae MGH 78578* | CP000647.1.gene2517.p01 | asmA | 2.0E-09 | 90.91 | 29 | 22 |
| 3000493 | *Klebsiella pneumoniae subsp. pneumoniae MGH 78578* | CP000647.1.gene956.p01 | ompF | 8.0E-08 | 96.15 | 26 | 7 |
| 3000493 | *Shigella dysenteriae Sd197* | CP000034.1.gene2198.p01 | asmA | 1.0E-06 | 90.91 | 27 | 61 |
| 3000493 | *Shigella dysenteriae Sd197* | CP000034.1.gene2328.p01 | ompF | 9.0E-14 | 100 | 33 | 1 |
| 3000493 | *Shigella dysenteriae Sd197* | CP000034.1.gene3671.p01 | ompR | 3.0E-11 | 93.94 | 29 | 26 |
| 3000493 | *Shigella dysenteriae Sd197* | CP000034.1.gene3672.p01 | envZ | 9.0E-07 | 90.62 | 25 | 69 |
| 3000496 | *Enterococcus faecalis* | DQ212986.1.gene9.p01 | vanXYG | 1.0E-12 | 90.91 | 33 | 1 |
| 3000496 | *Enterococcus gallinarum* | AF162694.1.orf2.gene.p01 | vanXYc | 2.0E-14 | 100 | 33 | 2 |
| 3000498 | *Bacteroides fragilis* | M14730.gene.p01 | ermF* | 2.0E-13 | 100 | 33 | 4 |
| 3000499 | *Enterococcus faecium DO* | CP003583.1.gene862.p01 | terL | 6.0E-13 | 100 | 33 | 2 |
| 3000499 | *Escherichia coli O157:H7 str. Sakai* | NC_002695.1.916015.p01 | ECs4137 | 1.0E-06 | 93.94 | 25 | 30 |
| 3000499 | *Klebsiella pneumoniae subsp. pneumoniae MGH 78578* | CP000647.1.gene3709.p01 | acrE | 2.0E-07 | 90.32 | 25 | 16 |
| 3000502 | *Klebsiella pneumoniae subsp. pneumoniae MGH 78578* | CP000647.1.gene3710.p01 | acrF | 8.0E-07 | 90.32 | 27 | 67 |
| 3000506 | *Azoarcus sp. BH72* | NC_008702.1.4610136.p01 | mexR | 3.0E-13 | 93.94 | 33 | 1 |
| 3000508 | *Escherichia coli O157:H7 str. Sakai* | NC_002695.1.915747.p01 | ECs4396 | 3.0E-06 | 93.94 | 25 | 25 |
| 3000516 | *Acinetobacter baumannii AB307-0294* | NC_011595.7058564.p01 | ABBFA_001502 | 6.0E-11 | 90.91 | 29 | 2 |
| 3000516 | *Klebsiella pneumoniae subsp. pneumoniae MGH 78578* | CP000647.1.gene3013.p01 | emrR | 1.0E-13 | 100 | 33 | 5 |
| 3000516 | *Shigella dysenteriae Sd197* | CP000034.1.gene2879.p01 | emrR | 1.0E-09 | 90.91 | 28 | 12 |
| 3000518 | *Klebsiella pneumoniae subsp. pneumoniae MGH 78578* | CP000647.1.gene3780.p01 | crp | 4.0E-12 | 100 | 33 | 7 |
| 3000518 | *Proteus mirabilis BB2000* | CP004022.1.gene2827.p01 | crp | 3.0E-09 | 100 | 25 | 12 |
| 3000518 | *Shigella dysenteriae Sd197* | CP000034.1.gene3519.p01 | crp | 2.0E-10 | 100 | 27 | 19 |
| 3000522 | *Lysinibacillus sphaericus* | M15332.gene.p01 | erm(G)**_ermG* | 3.0E-09 | 93.94 | 25 | 10 |
| 3000533 | *Acinetobacter baumannii AB307-0294* | NC_011595.7058613.p01 | ABBFA_003018 | 7.0E-10 | 90.91 | 30 | 5 |
| 3000533 | *Enterobacter cloacae subsp. cloacae ATCC 13047* | CP001918.1.gene2796.p01 | ECL_02771 | 1.0E-09 | 90.91 | 33 | 4 |
| 3000533 | *Escherichia coli O157:H7 str. Sakai* | NC_002695.1.917702.p01 | ECs0964 | 2.0E-06 | 93.94 | 25 | 32 |
| 3000533 | *Salmonella enterica subsp. enterica serovar Agona str. SL483* | CP001138.1.gene934.p01 | SeAg_B0942 | 2.0E-11 | 100 | 33 | 1 |
| 3000535 | *Acinetobacter baumannii AB307-0294* | NC_011595.7060505.p01 | ABBFA_003019 | 1.0E-06 | 90.91 | 25 | 22 |
| 3000535 | *Azoarcus sp. BH72* | NC_008702.1.4609454.p01 | macB | 3.0E-06 | 90 | 28 | 31 |
| 3000535 | *Clostridium botulinum A2 str. Kyoto* | CP001581.1.gene598.p01 | CLM_0622 | 5.0E-06 | 90 | 25 | 142 |
| 3000535 | *Clostridium botulinum A2 str. Kyoto* | CP001581.1.gene798.p01 | CLM_0827 | 4.0E-06 | 90.32 | 25 | 190 |
| 3000535 | *Klebsiella pneumoniae subsp. pneumoniae MGH 78578* | CP000647.1.gene911.p01 | macA | 1.0E-08 | 96.97 | 28 | 12 |
| 3000535 | *Klebsiella pneumoniae subsp. pneumoniae MGH 78578* | CP000647.1.gene912.p01 | macB | 4.0E-06 | 90 | 25 | 32 |
| 3000535 | *Listeria monocytogenes* | HE999704.1.gene196.p01 | BN418_0205 | 4.0E-06 | 90 | 25 | 19 |
| 3000535 | *Proteus mirabilis BB2000* | CP004022.1.gene758.p01 | macB | 5.0E-08 | 90.32 | 31 | 3 |
| 3000535 | *Salmonella enterica subsp. enterica serovar Agona str. SL483* | CP001138.1.gene935.p01 | SeAg_B0943 | 2.0E-06 | 90 | 25 | 18 |
| 3000535 | *Streptococcus pneumoniae Taiwan19F-14* | NC_012469.1.7685735.p01 | SPT_1593 | 4.0E-09 | 90 | 27 | 164 |
| 3000535 | *Streptococcus pneumoniae Taiwan19F-14* | NC_012469.1.7686878.p01 | SPT_1414 | 3.0E-08 | 90.62 | 26 | 7 |
| 3000553 | *Acinetobacter baumannii ATCC 17978* | NC_009085.4919120.p01 | A1S_1753 | 1.0E-13 | 96.97 | 33 | 1 |
| 3000556 | *Campylobacter fetus subsp. fetus* | FN594949.1.gene24.p01 | tet(44) | 7.0E-07 | 90 | 26 | 53 |
| 3000559 | *Shigella dysenteriae Sd197* | CP000034.1.gene1340.p01 | btuR | 5.0E-09 | 90.91 | 28 | 16 |
| 3000566 | *Acinetobacter sp. LUH5605* | AY743590.gene.p01 | tet39 | 6.0E-07 | 100 | 27 | 23 |
| 3000573 | *uncultured bacterium AOTet43* | GQ244501.1.gene1.p01 | tet43 | 3.0E-11 | 93.94 | 33 | 2 |
| 3000574 | *Amycolatopsis balhimycina* | Y16952.3.orf35.gene.p01 | vanR | 2.0E-09 | 90.62 | 28 | 8 |
| 3000574 | *Clostridium difficile 630* | AM180355.1.gene1830.p01 | vanR | 4.0E-09 | 90.32 | 26 | 23 |
| 3000574 | *Enterococcus faecalis* | AF253562.2.orf0.gene.p01 | vanRG | 5.0E-13 | 90.91 | 33 | 2 |
| 3000574 | *Enterococcus faecalis* | DQ212986.1.gene4.p01 | vanRG | 5.0E-10 | 90.62 | 32 | 5 |
| 3000574 | *Enterococcus faecalis* | NC_014475.1.orf0.gene.p01 | vanR | 2.0E-12 | 90.62 | 32 | 1 |
| 3000574 | *Enterococcus faecium* | AF130997.1.orf0.gene.p01 | vanRD | 1.0E-14 | 90.91 | 33 | 1 |
| 3000574 | *Enterococcus gallinarum* | AF162694.1.orf4.gene.p01 | vanRc | 1.0E-10 | 90.32 | 30 | 9 |
| 3000574 | *Streptomyces toyocaensis* | U82965.2.orf14.gene.p01 | U82965.2.orf14 | 1.0E-07 | 90 | 27 | 11 |
| 3000581 | *Bordetella pertussis CS* | CP002695.1.gene1737.p01 | cphA | 2.0E-09 | 90.91 | 33 | 1 |
| 3000593 | *Clostridium perfringens* | L22689.gene.p01 | ermQ* | 3.0E-13 | 100 | 33 | 5 |
| 3000595 | *Plasmid pGT633* | M64090.gene.p01 | ermT* | 7.0E-13 | 96.97 | 33 | 2 |
| 3000596 | *Plasmid pNG2* | M36726.gene.p01 | ermX* | 4.0E-13 | 100 | 33 | 1 |
| 3000602 | *Mycobacterium fortuitum* | AY487229.1.gene4.p01 | Erm(39) | 4.0E-12 | 93.94 | 33 | 1 |
| 3000606 | *Fluoribacter gormanii* | Y17896.1.gene1.p01 | blaFEZ-1 | 3.0E-12 | 93.94 | 33 | 1 |
| 3000616 | *Streptococcus pneumoniae Taiwan19F-14* | NC_012469.1.7685970.p01 | SPT_1925 | 3.0E-06 | 90.32 | 25 | 69 |
| 3000617 | *Enterococcus faecalis V583* | AE016830.1.gene2617.p01 | EF_2677 | 3.0E-11 | 100 | 30 | 2 |
| 3000617 | *Enterococcus faecium DO* | CP003583.1.gene2474.p01 | mecA | 1.0E-09 | 100 | 27 | 4 |
| 3000618 | *Acinetobacter baumannii AB307-0294* | NC_011595.7058445.p01 | gyrA | 3.0E-08 | 90 | 26 | 58 |
| 3000618 | *Acinetobacter baumannii SDF* | NC_010400.5986734.p01 | gyrA | 2.0E-09 | 90 | 30 | 4 |
| 3000618 | *Azoarcus sp. BH72* | NC_008702.1.4606680.p01 | gyrA | 3.0E-07 | 90.32 | 27 | 32 |
| 3000618 | *Bordetella pertussis CS* | CP002695.1.gene952.p01 | gyrA | 2.0E-06 | 90.32 | 25 | 53 |
| 3000618 | *Clostridium botulinum A2 str. Kyoto* | CP001581.1.gene7.p01 | gyrA | 3.0E-06 | 90 | 25 | 97 |
| 3000618 | *Clostridium difficile 630* | AM180355.1.gene6.p01 | gyrA | 8.0E-06 | 90 | 25 | 63 |
| 3000618 | *Enterobacter cloacae subsp. cloacae ATCC 13047* | CP001918.1.gene3562.p01 | ECL_03523 | 2.0E-06 | 92 | 25 | 8 |
| 3000618 | *Enterococcus faecalis V583* | AE016830.1.gene6.p01 | gyrA | 4.0E-06 | 90 | 25 | 128 |
| 3000618 | *Escherichia coli O157:H7 str. Sakai* | NC_002695.1.916822.p01 | ECs3114 | 2.0E-06 | 90 | 25 | 98 |
| 3000618 | *Haemophilus influenzae 10810* | FQ312006.1.gene1417.p01 | HIB_14190 | 2.0E-10 | 90.62 | 31 | 7 |
| 3000618 | *Helicobacter pylori Gambia94/24* | CP002332.1.gene704.p01 | HPGAM_03615 | 1.0E-06 | 90.32 | 26 | 2 |
| 3000618 | *Klebsiella pneumoniae subsp. pneumoniae MGH 78578* | CP000647.1.gene2640.p01 | gyrA | 4.0E-06 | 90.62 | 25 | 22 |
| 3000618 | *Legionella pneumophila str. Corby* | CP000675.2.gene1514.p01 | gyrA | 7.0E-08 | 90 | 27 | 19 |
| 3000618 | *Listeria monocytogenes* | HE999704.1.gene7.p01 | BN418_0007 | 7.0E-06 | 90 | 25 | 49 |
| 3000618 | *Mycobacterium tuberculosis CDC1551* | AE000516.2.gene6.p01 | gyrA | 2.0E-06 | 90.32 | 26 | 48 |
| 3000618 | *Neisseria gonorrhoeae NCCP11945* | NC_011035.1.6447337.p01 | NGK_1285 | 1.0E-07 | 90.62 | 28 | 18 |
| 3000618 | *Proteus mirabilis BB2000* | CP004022.1.gene1837.p01 | gyrA | 5.0E-06 | 90.32 | 26 | 11 |
| 3000618 | *Pseudomonas aeruginosa PAO1* | NC_002516.2.882800.p01 | gyrA | 5.0E-10 | 90.91 | 30 | 4 |
| 3000618 | *Salmonella enterica subsp. enterica serovar Agona str. SL483* | CP001138.1.gene2385.p01 | gyrA | 5.0E-06 | 90.32 | 25 | 34 |
| 3000618 | *Shigella dysenteriae Sd197* | CP000034.1.gene2423.p01 | gyrA | 9.0E-13 | 100 | 33 | 2 |
| 3000618 | *Staphylococcus aureus subsp. aureus COL* | NC_002951.3236187.p01 | gyrA | 4.0E-11 | 90.91 | 32 | 3 |
| 3000618 | *Staphylococcus aureus subsp. aureus Mu50* | NC_002758.1119966.p01 | gyrA | 9.0E-13 | 100 | 33 | 1 |
| 3000618 | *Staphylococcus aureus subsp. aureus N315* | NC_002745.1122777.p01 | gyrA | 3.0E-07 | 90 | 29 | 22 |
| 3000618 | *Staphylococcus aureus subsp. aureus str. Newman* | NC_009641.5331984.p01 | gyrA | 9.0E-10 | 93.94 | 30 | 2 |
| 3000618 | *Staphylococcus aureus subsp. aureus USA300_FPR3757* | NC_007793.3912939.p01 | gyrA | 6.0E-11 | 90.32 | 31 | 1 |
| 3000618 | *Staphylococcus epidermidis ATCC 12228* | AE015929.1.gene5.p01 | SE_0005 | 1.0E-07 | 90 | 25 | 24 |
| 3000618 | *Streptococcus pneumoniae Taiwan19F-14* | NC_012469.1.7686721.p01 | gyrA | 4.0E-06 | 90 | 25 | 132 |
| 3000618 | *Vibrio cholerae MJ-1236* | CP001485.1.gene2164.p01 | VCD_003093 | 2.0E-13 | 100 | 33 | 2 |
| 3000618 | *Yersinia pestis Antiqua* | CP000308.1.gene965.p01 | YPA_0930 | 2.0E-08 | 90.32 | 30 | 22 |
| 3000619 | *Acinetobacter baumannii ACICU* | NC_010611.6237080.p01 | parC | 2.0E-08 | 93.1 | 29 | 1 |
| 3000619 | *Acinetobacter baumannii AYE* | NC_010410.6003198.p01 | parC | 3.0E-11 | 90.91 | 32 | 2 |
| 3000619 | *Acinetobacter baumannii SDF* | NC_010400.5984045.p01 | parC | 2.0E-06 | 90.91 | 25 | 39 |
| 3000619 | *Bordetella pertussis CS* | CP002695.1.gene1275.p01 | parC | 1.0E-09 | 90 | 30 | 9 |
| 3000619 | *Clostridium botulinum A2 str. Kyoto* | CP001581.1.gene3048.p01 | CLM_3137 | 3.0E-14 | 90.91 | 33 | 1 |
| 3000619 | *Enterococcus faecalis V583* | AE016830.1.gene1598.p01 | parC | 2.0E-11 | 90.91 | 32 | 11 |
| 3000619 | *Enterococcus faecium DO* | CP003583.1.gene1174.p01 | parC | 6.0E-08 | 90.91 | 27 | 16 |
| 3000619 | *Escherichia coli str. K-12 substr. W3110* | M58408.gene.p01 | parC | 1.0E-06 | 90.91 | 25 | 58 |
| 3000619 | *Klebsiella pneumoniae subsp. pneumoniae MGH 78578* | CP000647.1.gene3437.p01 | parC | 2.0E-06 | 90.91 | 25 | 46 |
| 3000619 | *Legionella pneumophila str. Corby* | CP000675.2.gene3231.p01 | parC | 2.0E-10 | 90.91 | 32 | 3 |
| 3000619 | *Pseudomonas aeruginosa PAO1* | NC_002516.2.879741.p01 | parC | 5.0E-10 | 90.91 | 33 | 3 |
| 3000619 | *Salmonella enterica subsp. enterica serovar Agona str. SL483* | CP001138.1.gene3329.p01 | parC | 2.0E-09 | 93.94 | 29 | 18 |
| 3000619 | *Shigella dysenteriae Sd197* | CP000034.1.gene3218.p01 | parC | 5.0E-08 | 100 | 25 | 9 |
| 3000619 | *Staphylococcus aureus subsp. aureus MW2* | NC_003923.1003354.p01 | parC | 2.0E-09 | 93.94 | 29 | 2 |
| 3000619 | *Staphylococcus aureus subsp. aureus N315* | NC_002745.1124026.p01 | parC | 1.0E-09 | 90.91 | 29 | 7 |
| 3000619 | *Staphylococcus aureus subsp. aureus USA300_FPR3757* | NC_007793.3913356.p01 | parC | 6.0E-07 | 92 | 25 | 1 |
| 3000619 | *Streptococcus pneumoniae Taiwan19F-14* | NC_012469.1.7685406.p01 | parC | 3.0E-07 | 90 | 25 | 57 |
| 3000621 | *Acinetobacter baumannii AB0057* | NC_011586.7045516.p01 | AB57_0437 | 1.0E-11 | 90.91 | 32 | 10 |
| 3000621 | *Azoarcus sp. BH72* | NC_008702.1.4608898.p01 | azo0443 | 9.0E-13 | 90.91 | 33 | 1 |
| 3000621 | *Klebsiella pneumoniae* | AY034848.1.gene1.p01 | blaFOX-6 | 5.0E-13 | 96.97 | 33 | 1 |
| 3000621 | *Serratia marcescens* | X97254.1.gene1.p01 | blazZ | 5.0E-12 | 100 | 27 | 4 |
| 3000621 | *Staphylococcus aureus subsp. aureus USA300_TCH1516* | NC_010079.5775899.p01 | USA300HOU_1207 | 7.0E-11 | 90.91 | 32 | 6 |
| 3000656 | *Escherichia coli O157:H7 str. Sakai* | NC_002695.1.916016.p01 | ECs4136 | 2.0E-07 | 92.59 | 25 | 24 |
| 3000656 | *Klebsiella pneumoniae subsp. pneumoniae MGH 78578* | CP000647.1.gene3708.p01 | envR | 2.0E-14 | 100 | 33 | 3 |
| 3000662 | *Escherichia coli O157:H7 str. Sakai* | NC_002695.1.912474.p01 | ECs1443 | 7.0E-08 | 96.43 | 26 | 32 |
| 3000676 | *Acinetobacter baumannii AB0057* | NC_011586.7046013.p01 | AB57_0355 | 7.0E-07 | 90.91 | 27 | 5 |
| 3000676 | *Escherichia coli O157:H7 str. Sakai* | NC_002695.1.913113.p01 | ECs1739 | 2.0E-12 | 100 | 29 | 18 |
| 3000676 | *Salmonella enterica subsp. enterica serovar Agona str. SL483* | CP001138.1.gene1383.p01 | hns | 2.0E-07 | 90 | 25 | 2 |
| 3000702 | *Enterobacter cloacae subsp. cloacae ATCC 13047* | CP001918.1.gene1259.p01 | acrR | 1.0E-10 | 90.91 | 33 | 1 |
| 3000702 | *Klebsiella pneumoniae subsp. pneumoniae MGH 78578* | CP000647.1.gene445.p01 | acrR | 7.0E-11 | 96.97 | 30 | 13 |
| 3000702 | *Shigella dysenteriae Sd197* | CP000034.1.gene455.p01 | acrR | 2.0E-07 | 100 | 26 | 33 |
| 3000718 | *Acinetobacter baumannii ATCC 19606* | NC_006877.3293011.p01 | marR | 2.0E-12 | 96.97 | 33 | 5 |
| 3000718 | *Klebsiella pneumoniae subsp. pneumoniae MGH 78578* | CP000647.1.gene1252.p01 | KPN_01252 | 4.0E-11 | 96.55 | 29 | 4 |
| 3000718 | *Klebsiella pneumoniae subsp. pneumoniae MGH 78578* | CP000647.1.gene1489.p01 | KPN_01489 | 1.0E-11 | 90.91 | 33 | 1 |
| 3000718 | *Klebsiella pneumoniae subsp. pneumoniae MGH 78578* | CP000647.1.gene1625.p01 | marR | 1.0E-07 | 100 | 25 | 1 |
| 3000718 | *Klebsiella pneumoniae subsp. pneumoniae MGH 78578* | CP000647.1.gene1851.p01 | KPN_01851 | 6.0E-12 | 90.91 | 33 | 14 |
| 3000718 | *Klebsiella pneumoniae subsp. pneumoniae MGH 78578* | CP000647.1.gene3283.p01 | KPN_03283 | 2.0E-13 | 100 | 33 | 3 |
| 3000718 | *Klebsiella pneumoniae subsp. pneumoniae MGH 78578* | CP000647.1.gene4798.p01 | KPN_04761 | 3.0E-11 | 90.91 | 30 | 8 |
| 3000718 | *Shigella dysenteriae Sd197* | CP000034.1.gene1597.p01 | marR | 4.0E-09 | 96.97 | 27 | 19 |
| 3000718 | *Staphylococcus aureus subsp. aureus JH1* | NC_009632.5316408.p01 | SaurJH1_2332 | 2.0E-13 | 100 | 33 | 2 |
| 3000718 | *Staphylococcus aureus subsp. aureus JH1* | NC_009632.5317857.p01 | SaurJH1_2708 | 4.0E-15 | 100 | 33 | 1 |
| 3000718 | *Staphylococcus aureus subsp. aureus JH9* | NC_009487.5168369.p01 | SaurJH9_2543 | 9.0E-14 | 100 | 33 | 1 |
| 3000753 | *Acinetobacter baumannii AB0057* | NC_011586.7045550.p01 | abeM | 3.0E-09 | 90.91 | 32 | 9 |
| 3000774 | *Acinetobacter baumannii AYE* | NC_010410.6002906.p01 | adeA. | 2.0E-12 | 93.75 | 32 | 1 |
| 3000775 | *Acinetobacter baumannii ATCC 17978* | NC_009085.4919117.p01 | A1S_1750 | 3.0E-10 | 90.91 | 33 | 1 |
| 3000776 | *Acinetobacter baumannii AYE* | NC_010410.6002904.p01 | adeC | 1.0E-11 | 90.91 | 33 | 1 |
| 3000776 | *Enterococcus faecium DO* | CP003583.1.gene2512.p01 | adeC | 2.0E-11 | 93.94 | 33 | 12 |
| 3000777 | *Acinetobacter baumannii SDF* | NC_010400.5984910.p01 | ABSDF1463 | 4.0E-12 | 100 | 33 | 1 |
| 3000778 | *Acinetobacter baumannii SDF* | NC_010400.5984909.p01 | ABSDF1462 | 1.0E-10 | 93.94 | 33 | 2 |
| 3000780 | *Acinetobacter baumannii ATCC 17978* | NC_009085.4918693.p01 | A1S_2735 | 3.0E-11 | 90.91 | 33 | 2 |
| 3000781 | *Acinetobacter baumannii AB0057* | NC_011586.7045444.p01 | adeJ | 5.0E-06 | 90 | 25 | 36 |
| 3000782 | *Acinetobacter baumannii AB0057* | NC_011586.7045445.p01 | adeK | 2.0E-09 | 90.32 | 31 | 3 |
| 3000782 | *Acinetobacter baumannii ATCC 17978* | NC_009085.4918695.p01 | A1S_2737 | 9.0E-11 | 90.91 | 33 | 3 |
| 3000792 | *Enterobacter cloacae subsp. cloacae ATCC 13047* | CP001918.1.gene3439.p01 | ECL_03401 | 3.0E-11 | 96.97 | 33 | 6 |
| 3000792 | *Escherichia coli O157:H7 str. Sakai* | NC_002695.1.916584.p01 | ECs2882 | 3.0E-08 | 90.91 | 28 | 34 |
| 3000792 | *Lactococcus lactis subsp. lactis K214* | X92946.gene.p01 | mdtA | 2.0E-11 | 96.77 | 31 | 5 |
| 3000792 | *Salmonella enterica subsp. enterica serovar Agona str. SL483* | CP001138.1.gene2234.p01 | SeAg_B2256 | 5.0E-11 | 96.97 | 33 | 1 |
| 3000793 | *Enterobacter cloacae subsp. cloacae ATCC 13047* | CP001918.1.gene3440.p01 | ECL_03402 | 9.0E-09 | 90.62 | 31 | 18 |
| 3000793 | *Escherichia coli O157:H7 str. Sakai* | NC_002695.1.916585.p01 | ECs2883 | 8.0E-06 | 90 | 25 | 98 |
| 3000793 | *Proteus mirabilis BB2000* | CP004022.1.gene1673.p01 | mdtB | 9.0E-10 | 96.97 | 33 | 4 |
| 3000793 | *Salmonella enterica subsp. enterica serovar Agona str. SL483* | CP001138.1.gene2235.p01 | SeAg_B2257 | 1.0E-07 | 90 | 30 | 23 |
| 3000794 | *Enterobacter cloacae subsp. cloacae ATCC 13047* | CP001918.1.gene3441.p01 | ECL_03403 | 1.0E-06 | 90 | 28 | 15 |
| 3000794 | *Escherichia coli O157:H7 str. Sakai* | NC_002695.1.916586.p01 | ECs2884 | 2.0E-06 | 93.94 | 25 | 102 |
| 3000794 | *Proteus mirabilis BB2000* | CP004022.1.gene1674.p01 | mdtC | 8.0E-06 | 90.91 | 25 | 5 |
| 3000794 | *Salmonella enterica subsp. enterica serovar Agona str. SL483* | CP001138.1.gene2236.p01 | mdtC | 5.0E-06 | 90.62 | 25 | 34 |
| 3000795 | *Salmonella enterica subsp. enterica serovar Agona str. SL483* | NC_002695.1.915750.p01 | ECs4393 | 3.0E-07 | 92.59 | 26 | 41 |
| 3000795 | *Enterobacter cloacae subsp. cloacae ATCC 13047* | CP001918.1.gene3442.p01 | ECL_03404 | 1.0E-09 | 90.32 | 31 | 4 |
| 3000795 | *Escherichia coli O157:H7 str. Sakai* | NC_002695.1.916587.p01 | ECs2885 | 1.0E-06 | 90.62 | 25 | 52 |
| 3000801 | *Pseudomonas aeruginosa* | U57969.gene.p01 | mexD | 5.0E-08 | 90 | 30 | 3 |
| 3000804 | *Pseudomonas aeruginosa PAO1* | NC_002516.2.882884.p01 | mexF | 1.0E-09 | 93.75 | 32 | 4 |
| 3000814 | *Acinetobacter baumannii AB307-0294* | NC_011595.7059912.p01 | ABBFA_002603 | 8.0E-12 | 90.91 | 32 | 27 |
| 3000816 | *Mycobacterium tuberculosis CDC1551* | AE000516.2.gene3505.p01 | mtrA | 1.0E-09 | 90.91 | 30 | 7 |
| 3000822 | *Enterococcus faecium DO* | CP003583.1.gene1005.p01 | pmrA | 1.0E-11 | 93.94 | 33 | 6 |
| 3000822 | *Staphylococcus aureus subsp. aureus MRSA252* | NC_002952.2860819.p01 | SAR0122 | 1.0E-12 | 100 | 33 | 2 |
| 3000826 | *Escherichia coli O157:H7 str. Sakai* | NC_002695.1.912965.p01 | ECs2654 | 2.0E-08 | 92.59 | 27 | 26 |
| 3000826 | *Klebsiella pneumoniae subsp. pneumoniae MGH 78578* | CP000647.1.gene2414.p01 | sdiA | 7.0E-09 | 90.91 | 25 | 8 |
| 3000828 | *Acinetobacter baumannii AB307-0294* | NC_011595.7057856.p01 | ABBFA_000579 | 3.0E-10 | 93.94 | 30 | 13 |
| 3000828 | *Acinetobacter baumannii SDF* | NC_010400.5986590.p01 | baeR | 2.0E-13 | 96.97 | 33 | 3 |
| 3000828 | *Enterobacter cloacae subsp. cloacae ATCC 13047* | CP001918.1.gene3444.p01 | ECL_03406 | 2.0E-11 | 100 | 29 | 1 |
| 3000828 | *Escherichia coli O157:H7 str. Sakai* | NC_002695.1.916589.p01 | ECs2887 | 2.0E-08 | 100 | 26 | 8 |
| 3000828 | *Salmonella enterica subsp. enterica serovar Agona str. SL483* | CP001138.1.gene2239.p01 | baeR | 3.0E-10 | 100 | 29 | 2 |
| 3000829 | *Shigella dysenteriae Sd197* | CP000034.1.gene2186.p01 | baeR | 9.0E-11 | 100 | 29 | 2 |
| 3000829 | *Shigella dysenteriae Sd197* | CP000034.1.gene2187.p01 | baeS | 1.0E-12 | 100 | 33 | 4 |
| 3000829 | *Klebsiella pneumoniae subsp. pneumoniae MGH 78578* | CP000647.1.gene2530.p01 | baeS | 5.0E-09 | 96.97 | 26 | 16 |
| 3000829 | *Klebsiella pneumoniae subsp. pneumoniae MGH 78578* | CP000647.1.gene2531.p01 | baeR | 2.0E-07 | 93.94 | 25 | 8 |
| 3000829 | *Acinetobacter baumannii AB307-0294* | NC_011595.7057524.p01 | ABBFA_000578 | 1.0E-10 | 90.62 | 30 | 6 |
| 3000829 | *Acinetobacter baumannii SDF* | NC_010400.5984250.p01 | baeS | 5.0E-08 | 92.31 | 26 | 2 |
| 3000829 | *Enterobacter cloacae subsp. cloacae ATCC 13047* | CP001918.1.gene3443.p01 | ECL_03405 | 9.0E-07 | 92.31 | 26 | 5 |
| 3000829 | *Escherichia coli O157:H7 str. Sakai* | NC_002695.1.916588.p01 | ECs2886 | 1.0E-06 | 93.94 | 25 | 30 |
| 3000830 | *Escherichia coli O157:H7 str. Sakai* | NC_002695.1.914983.p01 | cpxA | 4.0E-07 | 96 | 25 | 46 |
| 3000830 | *Klebsiella pneumoniae subsp. pneumoniae MGH 78578* | CP000647.1.gene4256.p01 | cpxA | 4.0E-09 | 93.94 | 27 | 15 |
| 3000831 | *Enterobacter cloacae subsp. cloacae ATCC 13047* | CP001918.1.gene5135.p01 | ECL_05064 | 3.0E-11 | 96.77 | 31 | 2 |
| 3000831 | *Escherichia coli O157:H7 str. Sakai* | NC_002695.1.915041.p01 | ECs4838 | 3.0E-09 | 96.67 | 25 | 19 |
| 3000831 | *Klebsiella pneumoniae subsp. pneumoniae MGH 78578* | CP000647.1.gene4257.p01 | cpxR | 4.0E-14 | 100 | 33 | 2 |
| 3000831 | *Proteus mirabilis BB2000* | CP004022.1.gene3215.p01 | cpxR | 2.0E-11 | 90.91 | 33 | 6 |
| 3000831 | *Salmonella enterica subsp. enterica serovar Agona str. SL483* | CP001138.1.gene4273.p01 | cpxR | 4.0E-09 | 93.55 | 28 | 5 |
| 3000831 | *Vibrio cholerae MJ-1236* | CP001485.1.gene721.p01 | VCD_001676 | 1.0E-11 | 90.62 | 32 | 1 |
| 3000832 | *Klebsiella pneumoniae subsp. pneumoniae MGH 78578* | CP000647.1.gene3517.p01 | evgA | 1.0E-11 | 100 | 27 | 8 |
| 3000833 | *Shigella dysenteriae Sd197* | CP000034.1.gene4478.p01 | evgS | 3.0E-06 | 93.94 | 25 | 20 |
| 3000833 | *Escherichia coli O157:H7 str. Sakai* | NC_002695.1.915650.p01 | ECs3249 | 5.0E-07 | 90.91 | 25 | 86 |
| 3000833 | *Escherichia coli O157:H7 str. Sakai* | NC_002695.1.915651.p01 | ECs3248 | 7.0E-08 | 100 | 25 | 7 |
| 3000833 | *Klebsiella pneumoniae subsp. pneumoniae MGH 78578* | CP000647.1.gene3518.p01 | evgS | 6.0E-07 | 93.94 | 25 | 66 |
| 3000834 | *Enterobacter cloacae subsp. cloacae ATCC 13047* | CP001918.1.gene2526.p01 | ECL_02504 | 1.0E-14 | 100 | 33 | 1 |
| 3000834 | *Enterococcus faecalis V583* | AE016830.1.gene1681.p01 | phoP | 1.0E-07 | 92 | 25 | 1 |
| 3000834 | *Klebsiella pneumoniae subsp. pneumoniae MGH 78578* | CP000647.1.gene2625.p01 | yejM | 1.0E-07 | 90.62 | 26 | 53 |
| 3000834 | *Listeria monocytogenes* | HE999704.1.gene2815.p01 | BN418_2960 | 4.0E-12 | 90.91 | 33 | 1 |
| 3000834 | *Salmonella enterica subsp. enterica serovar Agona str. SL483* | CP001138.1.gene1939.p01 | SeAg_B1953 | 5.0E-14 | 100 | 33 | 1 |
| 3000834 | *Shigella dysenteriae Sd197* | CP000034.1.gene2022.p01 | phoP | 1.0E-06 | 96.88 | 28 | 10 |
| 3000834 | *Staphylococcus aureus subsp. aureus N315* | NC_002745.1124361.p01 | phoP | 9.0E-11 | 90.32 | 31 | 1 |
| 3000834 | *Streptococcus pneumoniae Taiwan19F-14* | NC_012469.1.7685629.p01 | SPT_1001 | 2.0E-07 | 90.32 | 25 | 7 |
| 3000835 | *Klebsiella pneumoniae subsp. pneumoniae MGH 78578* | CP000647.1.gene1136.p01 | phoP | 6.0E-06 | 96.97 | 31 | 8 |
| 3000835 | *Escherichia coli O157:H7 str. Sakai* | NC_002695.1.913289.p01 | ECs1602 | 7.0E-06 | 96.97 | 25 | 17 |
| 3000835 | *Escherichia coli O157:H7 str. Sakai* | NC_002695.1.913290.p01 | ECs1601 | 4.0E-07 | 96.67 | 26 | 32 |
| 3000835 | *Klebsiella pneumoniae subsp. pneumoniae MGH 78578* | CP000647.1.gene1135.p01 | phoQ | 3.0E-08 | 90.62 | 31 | 22 |
| 3000835 | *Salmonella enterica subsp. enterica serovar Agona str. SL483* | CP001138.1.gene1940.p01 | SeAg_B1954 | 6.0E-14 | 100 | 32 | 3 |
| 3000835 | *Shigella dysenteriae Sd197* | CP000034.1.gene2023.p01 | phoQ | 9.0E-13 | 100 | 33 | 10 |
| 3000836 | *Klebsiella pneumoniae subsp. pneumoniae MGH 78578* | CP000647.1.gene4500.p01 | soxR | 8.0E-13 | 93.75 | 32 | 6 |
| 3000836 | *Escherichia coli O157:H7 str. Sakai* | NC_002695.1.914292.p01 | ECs5045 | 5.0E-09 | 96.3 | 26 | 16 |
| 3000836 | *Salmonella enterica subsp. enterica serovar Agona str. SL483* | CP001138.1.gene4489.p01 | soxR | 1.0E-08 | 92.31 | 26 | 7 |
| 3000837 | *Escherichia coli O157:H7 str. Sakai* | NC_002695.1.914293.p01 | ECs5044 | 1.0E-08 | 90.62 | 25 | 10 |
| 3000837 | *Escherichia coli O157:H7 str. Sakai* | NC_002695.1.917670.p01 | ECs0930 | 4.0E-12 | 93.94 | 30 | 7 |
| 3000837 | *Klebsiella pneumoniae subsp. pneumoniae MGH 78578* | CP000647.1.gene4499.p01 | soxS | 2.0E-15 | 96.97 | 33 | 2 |
| 3000837 | *Shigella dysenteriae Sd197* | CP000034.1.gene4505.p01 | soxS | 5.0E-12 | 100 | 29 | 1 |
| 3000838 | *Listeria monocytogenes* | HE999704.1.gene1528.p01 | BN418_1620 | 3.0E-09 | 90.91 | 27 | 14 |
| 3000839 | *Staphylococcus aureus subsp. aureus N315* | NC_002745.1124085.p01 | arlS | 3.0E-11 | 100 | 33 | 1 |
| 3000863 | *Staphylococcus aureus subsp. aureus N315* | NC_002745.1124031.p01 | fmtC | 4.0E-10 | 96.97 | 33 | 2 |
| 3000873 | *Escherichia coli 1520* | NC_010558.1.6276043.p01 | blaTEM-1 | 3.0E-11 | 100 | 26 | 8 |
| 3000873 | *Klebsiella pneumoniae* | JF949915.1.gene1.p01 | blaTEM-1 | 1.0E-13 | 100 | 33 | 2 |
| 3000893 | *Klebsiella pneumoniae* | Y17583.1.gene1.p01 | blaTEM-22 | 1.0E-11 | 100 | 29 | 1 |
| 3000899 | *Escherichia coli* | Y17584.1.gene1.p01 | blaTEM-29 | 2.0E-13 | 100 | 33 | 1 |
| 3000903 | *Escherichia coli* | GU371926.1.gene95.p01 | blaTEM-33 | 2.0E-06 | 90 | 26 | 12 |
| 3000916 | *Klebsiella pneumoniae* | Y10279.1.gene1.p01 | blaTEM-47 | 2.0E-14 | 100 | 33 | 1 |
| 3000917 | *Klebsiella pneumoniae* | Y10280.1.gene1.p01 | blaTEM-48 | 4.0E-13 | 100 | 33 | 4 |
| 3000924 | *Escherichia coli* | DQ286729.1.gene1.p1 | ABB97007.1 | 2.0E-13 | 100 | 33 | 1 |
| 3000931 | *Escherichia coli* | AF332513.1.gene1.p01 | blaTEM-63 | 8.0E-15 | 100 | 33 | 1 |
| 3000941 | *Klebsiella pneumoniae* | AY130284.1.gene1.p1 | AAN05028.1 | 1.0E-09 | 100 | 28 | 9 |
| 3000950 | *Escherichia coli* | AF427129.1.gene1.p01 | blaTEM-83 | 3.0E-14 | 100 | 33 | 1 |
| 3000954 | *Proteus mirabilis* | AF250872.1.gene1.p1 | AAG44570.1 | 2.0E-13 | 100 | 33 | 3 |
| 3000958 | *Escherichia coli* | AB049569.1.gene1.p01 | blaTEM-91 | 5.0E-14 | 100 | 33 | 3 |
| 3000980 | *Escherichia coli* | AY130282.1.gene1.p1 | AAN05026.1 | 5.0E-09 | 100 | 25 | 8 |
| 3000981 | *Klebsiella oxytoca* | AY130285.1.gene1.p1 | AAN05029.1 | 3.0E-14 | 100 | 33 | 1 |
| 3001002 | *Salmonella enterica* | AY853593.1.gene1.p1 | AAW47922.1 | 8.0E-16 | 100 | 33 | 1 |
| 3001065 | *Klebsiella pneumoniae* | Y11069.1.gene1.p01 | blaSHV-6 | 4.0E-09 | 100 | 27 | 3 |
| 3001110 | *Klebsiella pneumoniae* | AY590467.1.gene1.p1 | AAT01223.1 | 7.0E-11 | 100 | 28 | 1 |
| 3001205 | *Campylobacter fetus subsp. fetus* | FN594949.1.gene22.p01 | blmA | 2.0E-12 | 96.97 | 33 | 1 |
| 3001205 | *Klebsiella pneumoniae subsp. pneumoniae MGH 78578* | CP000647.1.gene1649.p01 | KPN_01649 | 8.0E-16 | 100 | 33 | 1 |
| 3001205 | *Klebsiella pneumoniae subsp. pneumoniae MGH 78578* | CP000647.1.gene3367.p01 | KPN_03367 | 2.0E-08 | 96.97 | 25 | 9 |
| 3001211 | *Escherichia coli* | DQ464881.1.gene4.p01 | strA | 1.0E-10 | 100 | 28 | 14 |
| 3001211 | *Pseudomonas aeruginosa* | AF024602.1.gene5.p01 | strA | 8.0E-10 | 96.15 | 26 | 16 |
| 3001212 | *Acinetobacter baumannii AYE* | NC_010410.6003392.p01 | strB | 8.0E-13 | 100 | 33 | 8 |
| 3001212 | *Escherichia coli* | AJ313522.gene.p01 | strB | 6.0E-13 | 100 | 32 | 5 |
| 3001212 | *Escherichia coli* | DQ464881.1.gene5.p01 | strB | 5.0E-09 | 96.55 | 26 | 26 |
| 3001212 | *Pseudomonas aeruginosa* | AF024602.1.gene6.p01 | strB | 1.0E-12 | 100 | 30 | 6 |
| 3001213 | *Acinetobacter baumannii AYE* | NC_010410.6003949.p01 | ABAYE3640 | 6.0E-10 | 90.91 | 33 | 3 |
| 3001214 | *Salmonella enterica subsp. enterica serovar Agona str. SL483* | CP001138.1.gene4809.p01 | SeAg_B4848 | 7.0E-07 | 90 | 26 | 9 |
| 3001214 | *Salmonella enterica subsp. enterica serovar Agona str. SL483* | CP001138.1.gene894.p01 | SeAg_B0902 | 2.0E-08 | 90.62 | 29 | 43 |
| 3001215 | *Escherichia coli O157:H7 str. Sakai* | NC_002695.1.915390.p01 | ECs4647 | 1.0E-06 | 93.1 | 25 | 39 |
| 3001215 | *Salmonella enterica subsp. enterica serovar Agona str. SL483* | CP001138.1.gene4042.p01 | SeAg_B4071 | 2.0E-11 | 100 | 33 | 5 |
| 3001216 | *Enterobacter cloacae subsp. cloacae ATCC 13047* | CP001918.1.gene2596.p01 | ECL_02574 | 5.0E-09 | 100 | 28 | 2 |
| 3001216 | *Salmonella enterica subsp. enterica serovar Agona str. SL483* | CP001138.1.gene2007.p01 | SeAg_B2022 | 2.0E-07 | 100 | 26 | 7 |

**Matched contigs of sample C1756 against CARD**

(Sorted by ARO-number)

| **ARO** | **Organism** | **Accession number** | **Gene** | **E value ≤** | **Identity (%) ≥** | **Hit length (aa) ≥** | **Number of contigs** |
| --- | --- | --- | --- | --- | --- | --- | --- |
| 3000002 | Enterococcus faecalis | AF253562.2.orf3.gene.p01 | vanWG | 2.0E-18 | 92.86 | 42 | 1 |
| 3000019 | Escherichia coli O157:H7 str. Sakai | NC_002695.1.914045.p01 | ampC | 3.0E-63 | 98.06 | 103 | 2 |
| 3000054 | Klebsiella pneumoniae subsp. pneumoniae MGH 78578 | CP000647.1.gene444.p01 | acrA | 1.0E-70 | 99.29 | 111 | 3 |
| 3000054 | Escherichia coli O157:H7 str. Sakai | NC_002695.1.914620.p01 | ECs0516 | < 1.0E-150 | 100 | 293 | 1 |
| 3000074 | Escherichia coli O157:H7 str. Sakai | NC_002695.1.914736.p01 | ECs3548 | 3.0E-76 | 99.22 | 114 | 3 |
| 3000122 | Klebsiella pneumoniae subsp. pneumoniae MGH 78578 | CP000647.1.gene2018.p01 | CatA1 | 5.0E-142 | 99.49 | 195 | 1 |
| 3000122 | Escherichia coli 1520 | NC_010558.1.6276004.p01 | KPN_02018 | 1.0E-126 | 100 | 171 | 1 |
| 3000126 | Escherichia coli | V00359.1.orf1.gene.p01 | V01547.1.orf0 | 4.0E-110 | 100 | 154 | 1 |
| 3000126 | Enterococcus faecalis | V01547.1.orf0.gene.p01 | V00359.1.orf1 | < 1.0E-150 | 100 | 264 | 1 |
| 3000168 | Plasmid pRA1 | L06798.gene.p01 | tetD | 2.0E-29 | 100 | 56 | 2 |
| 3000186 | Enterococcus faecalis | M85225.gene.p01 | tetM | 1.0E-107 | 100 | 155 | 2 |
| 3000190 | Megasphaera elsdenii | AY485126.gene.p01 | tetOW | 1.0E-22 | 91.49 | 47 | 1 |
| 3000191 | Bacteroides fragilis | Z21523.gene.p01 | tetQ | 2.0E-51 | 95.93 | 86 | 3 |
| 3000194 | Butyrivibrio fibrisolvens | AJ222769.gene.p01 | tetW_2 | 2.0E-125 | 100 | 183 | 3 |
| 3000194 | Bifidobacterium longum subsp. longum F8 | DQ294299.gene.p01 | tetW* | < 1.0E-150 | 99.65 | 283 | 1 |
| 3000196 | Clostridiaceae bacterium K10 | AJ295238.gene.p01 | tet32 | 3.0E-14 | 93.75 | 32 | 3 |
| 3000210 | Enterococcus faecalis V583 | AE016830.1.gene3155.p01 | rpoB | 8.0E-13 | 90 | 28 | 3 |
| 3000210 | Clostridium difficile 630 | AM180355.1.gene120.p01 | rpoB | 3.0E-10 | 90.62 | 32 | 2 |
| 3000210 | Escherichia coli O157:H7 str. Sakai | NC_002695.1.914942.p01 | rpoB | < 1.0E-150 | 99.81 | 513 | 2 |
| 3000210 | Staphylococcus aureus subsp. aureus N315 | NC_002745.1123305.p01 | rpoB | 6.0E-49 | 90 | 90 | 1 |
| 3000210 | Acinetobacter baumannii ATCC 17978 | NC_009085.4918494.p01 | rpoB | 3.0E-47 | 94.2 | 88 | 2 |
| 3000210 | Acinetobacter baumannii SDF | NC_010400.5987325.p01 | rpoB | 1.0E-16 | 94.87 | 39 | 1 |
| 3000210 | Neisseria gonorrhoeae NCCP11945 | NC_011035.1.6448762.p01 | rpoB | 9.0E-34 | 91.18 | 68 | 1 |
| 3000210 | Streptococcus pneumoniae Taiwan19F-14 | NC_012469.1.7686402.p01 | rpoB | 2.0E-64 | 92.09 | 107 | 3 |
| 3000216 | Klebsiella pneumoniae subsp. pneumoniae MGH 78578 | CP000647.1.gene443.p01 | acrB | 7.0E-37 | 90.91 | 77 | 1 |
| 3000216 | Escherichia coli O157:H7 str. Sakai | NC_002695.1.912777.p01 | ECs0515 | 2.0E-52 | 98.15 | 108 | 1 |
| 3000216 | Escherichia coli O157:H7 str. Sakai | NC_002695.1.914619.p01 | ECs1864 | 6.0E-91 | 99.29 | 140 | 1 |
| 3000225 | Streptococcus pneumoniae | AM410044.gene9.p01 | ant(6)-Ib | 3.0E-14 | 100 | 32 | 1 |
| 3000225 | Campylobacter fetus subsp. fetus | FN594949.1.gene25.p01 | aadE | 6.0E-89 | 100 | 128 | 1 |
| 3000226 | Klebsiella pneumoniae subsp. pneumoniae MGH 78578 | CP000647.1.gene3624.p01 | folP | 6.0E-85 | 100 | 121 | 1 |
| 3000226 | Escherichia coli O157:H7 str. Sakai | NC_002695.1.916103.p01 | folP | 3.0E-25 | 100 | 52 | 3 |
| 3000232 | Salmonella enterica subsp. enterica serovar Typhi | AY123251.gene3.p01 | aadA1 | 2.0E-64 | 100 | 97 | 1 |
| 3000237 | Escherichia coli O157:H7 str. Sakai | NC_002695.1.916248.p01 | tolC | 5.0E-87 | 100 | 151 | 2 |
| 3000252 | Enterobacter aerogenes | AF336095.1.gene1.p01 | omp36 | 3.0E-26 | 92.59 | 54 | 1 |
| 3000263 | Salmonella enterica subsp. enterica serovar Agona str. SL483 | CP001138.1.gene1637.p01 | SeAg_B1651 | 2.0E-24 | 100 | 45 | 1 |
| 3000264 | Escherichia coli O157:H7 str. Sakai | NC_002695.1.913273.p01 | emrE | 4.0E-66 | 98.18 | 110 | 1 |
| 3000309 | Escherichia coli O157:H7 str. Sakai | NC_002695.1.915420.p01 | emrD | 1.0E-73 | 98.15 | 162 | 2 |
| 3000322 | Plasmid pWP14a | X13542.gene.p01 | aac(3)-III | 1.0E-17 | 95.35 | 43 | 1 |
| 3000373 | Escherichia coli O157:H7 str. Sakai | NC_002695.1.915653.p01 | ECs3247 | < 1.0E-150 | 99.22 | 387 | 1 |
| 3000375 | Streptococcus pneumoniae | AM410044.gene14.p01 | ermB | 4.0E-08 | 96.3 | 27 | 3 |
| 3000410 | Klebsiella pneumoniae | AF322577.2.gene6.p01 | sul1 | 1.0E-74 | 100 | 114 | 1 |
| 3000410 | Pseudomonas aeruginosa | U37105.2.gene6.p01 | sul1 | 2.0E-65 | 100 | 105 | 1 |
| 3000412 | Escherichia coli | DQ464881.1.gene2.p01 | sul2 | 1.0E-38 | 98.34 | 70 | 2 |
| 3000457 | Shigella dysenteriae Sd197 | CP000034.1.gene3210.p01 | parE | < 1.0E-150 | 99.65 | 242 | 2 |
| 3000457 | Klebsiella pneumoniae subsp. pneumoniae MGH 78578 | CP000647.1.gene3444.p01 | parE | 3.0E-13 | 100 | 33 | 2 |
| 3000457 | Acinetobacter baumannii SDF | NC_010400.5986295.p01 | parE | 1.0E-83 | 95.49 | 133 | 1 |
| 3000457 | Acinetobacter baumannii ACICU | NC_010611.6235284.p01 | parE | 8.0E-75 | 91.87 | 123 | 1 |
| 3000491 | Shigella dysenteriae Sd197 | CP000034.1.gene2654.p01 | ECs3332 | < 1.0E-150 | 100 | 400 | 1 |
| 3000491 | Escherichia coli O157:H7 str. Sakai | NC_002695.1.915267.p01 | acrD | 2.0E-43 | 99.08 | 75 | 2 |
| 3000493 | Shigella dysenteriae Sd197 | CP000034.1.gene2198.p01 | ompF | 2.0E-79 | 96.83 | 126 | 3 |
| 3000493 | Shigella dysenteriae Sd197 | CP000034.1.gene2328.p01 | asmA | 8.0E-65 | 96.36 | 110 | 1 |
| 3000493 | Shigella dysenteriae Sd197 | CP000034.1.gene3671.p01 | ompF | 2.0E-42 | 100 | 72 | 2 |
| 3000493 | Shigella dysenteriae Sd197 | CP000034.1.gene3672.p01 | asmA | 5.0E-107 | 90.99 | 157 | 2 |
| 3000493 | Klebsiella pneumoniae subsp. pneumoniae MGH 78578 | CP000647.1.gene2517.p01 | ompF | 5.0E-39 | 98.61 | 72 | 1 |
| 3000493 | Klebsiella pneumoniae subsp. pneumoniae MGH 78578 | CP000647.1.gene956.p01 | ompR | 4.0E-10 | 96.3 | 27 | 1 |
| 3000493 | Escherichia coli | GQ465831.1.gene2.p01 | envZ | 2.0E-77 | 93.98 | 133 | 1 |
| 3000499 | Klebsiella pneumoniae subsp. pneumoniae MGH 78578 | CP000647.1.gene3709.p01 | ECs4137 | 5.0E-63 | 98.04 | 102 | 1 |
| 3000499 | Escherichia coli O157:H7 str. Sakai | NC_002695.1.916015.p01 | acrE | 6.0E-64 | 98.47 | 83 | 3 |
| 3000502 | Klebsiella pneumoniae subsp. pneumoniae MGH 78578 | CP000647.1.gene3710.p01 | acrF | 8.0E-42 | 95.06 | 81 | 1 |
| 3000508 | Escherichia coli O157:H7 str. Sakai | NC_002695.1.915747.p01 | ECs4396 | 3.0E-38 | 91.96 | 64 | 3 |
| 3000516 | Shigella dysenteriae Sd197 | CP000034.1.gene2879.p01 | emrR | 1.0E-53 | 98.95 | 84 | 2 |
| 3000516 | Klebsiella pneumoniae subsp. pneumoniae MGH 78578 | CP000647.1.gene3013.p01 | emrR | 3.0E-60 | 100 | 91 | 1 |
| 3000518 | Shigella dysenteriae Sd197 | CP000034.1.gene3519.p01 | crp | 5.0E-149 | 100 | 210 | 1 |
| 3000522 | Lysinibacillus sphaericus | M15332.gene.p01 | erm(G)**_ermG* | 4.0E-87 | 100 | 127 | 1 |
| 3000533 | Escherichia coli O157:H7 str. Sakai | NC_002695.1.917702.p01 | ECs0964 | 1.0E-78 | 99.15 | 116 | 2 |
| 3000535 | Klebsiella pneumoniae subsp. pneumoniae MGH 78578 | CP000647.1.gene911.p01 | macA | 6.0E-35 | 100 | 63 | 2 |
| 3000535 | Klebsiella pneumoniae subsp. pneumoniae MGH 78578 | CP000647.1.gene912.p01 | macB | 5.0E-26 | 91.38 | 58 | 1 |
| 3000535 | Salmonella enterica subsp. enterica serovar Agona str. SL483 | CP001138.1.gene935.p01 | SeAg_B0943 | 2.0E-59 | 92.59 | 108 | 1 |
| 3000556 | Campylobacter fetus subsp. fetus | FN594949.1.gene24.p01 | tet44 | 7.0E-109 | 100 | 159 | 1 |
| 3000559 | Shigella dysenteriae Sd197 | CP000034.1.gene1340.p01 | btuR | 1.0E-41 | 97.6 | 94 | 2 |
| 3000566 | Acinetobacter sp. LUH5605 | AY743590.gene.p01 | tet39 | < 1.0E-150 | 100 | 283 | 1 |
| 3000574 | Streptomyces toyocaensis | U82965.2.orf14.gene.p01 | U82965.2.orf14 | 2.0E-51 | 90.11 | 91 | 1 |
| 3000616 | Streptococcus pneumoniae Taiwan19F-14 | NC_012469.1.7685970.p01 | SPT_1925 | 0.0E+00 | 95.05 | 485 | 1 |
| 3000618 | Clostridium difficile 630 | AM180355.1.gene6.p01 | gyrA | 9.0E-74 | 90.48 | 126 | 1 |
| 3000618 | Shigella dysenteriae Sd197 | CP000034.1.gene2423.p01 | gyrA | 1.0E-139 | 99.04 | 208 | 1 |
| 3000618 | Klebsiella pneumoniae subsp. pneumoniae MGH 78578 | CP000647.1.gene2640.p01 | gyrA | 5.0E-09 | 100 | 29 | 1 |
| 3000618 | Escherichia coli O157:H7 str. Sakai | NC_002695.1.916822.p01 | ECs3114 | 9.0E-28 | 99.18 | 72 | 5 |
| 3000618 | Azoarcus sp. BH72 | NC_008702.1.4606680.p01 | gyrA | 2.0E-18 | 97.67 | 43 | 1 |
| 3000618 | Acinetobacter baumannii SDF | NC_010400.5986734.p01 | gyrA | 2.0E-80 | 91.37 | 139 | 1 |
| 3000618 | Streptococcus pneumoniae Taiwan19F-14 | NC_012469.1.7686721.p01 | gyrA | 7.0E-101 | 93.98 | 83 | 1 |
| 3000619 | Escherichia coli str. K-12 substr. W3110 | M58408.gene.p01 | parC | 2.0E-55 | 99.75 | 102 | 3 |
| 3000619 | Acinetobacter baumannii SDF | NC_010400.5984045.p01 | parC | 3.0E-79 | 93.13 | 131 | 1 |
| 3000619 | Acinetobacter baumannii ACICU | NC_010611.6237080.p01 | parC | 1.0E-23 | 91.77 | 49 | 2 |
| 3000656 | Escherichia coli O157:H7 str. Sakai | NC_002695.1.916016.p01 | ECs4136 | 3.0E-137 | 99.48 | 192 | 1 |
| 3000662 | [Escherichia coli O157:H7 str. Sakai | NC_002695.1.912474.p01 | ECs1443 | 7.0E-88 | 99.51 | 133 | 2 |
| 3000676 | Escherichia coli O157:H7 str. Sakai | NC_002695.1.913113.p01 | ECs1739 | 2.0E-57 | 100 | 137 | 1 |
| 3000702 | Shigella dysenteriae Sd197 | CP000034.1.gene455.p01 | acrR | < 1.0E-150 | 100 | 215 | 1 |
| 3000702 | Klebsiella pneumoniae subsp. pneumoniae MGH 78578 | CP000647.1.gene445.p01 | acrR | 5.0E-105 | 100 | 146 | 1 |
| 3000718 | Klebsiella pneumoniae subsp. pneumoniae MGH 78578 | CP000647.1.gene1851.p01 | KPN_01851 | 9.0E-50 | 98.72 | 78 | 1 |
| 3000718 | Klebsiella pneumoniae subsp. pneumoniae MGH 78578 | CP000647.1.gene4798.p01 | KPN_04761 | 7.0E-53 | 98.81 | 84 | 1 |
| 3000792 | Escherichia coli O157:H7 str. Sakai | NC_002695.1.916584.p01 | ECs2882 | 1.0E-18 | 98.17 | 42 | 2 |
| 3000793 | Escherichia coli O157:H7 str. Sakai | NC_002695.1.916585.p01 | ECs2883 | 1.0E-42 | 98.95 | 97 | 5 |
| 3000794 | Escherichia coli O157:H7 str. Sakai | NC_002695.1.916586.p01 | ECs2884 | 5.0E-88 | 97.06 | 170 | 2 |
| 3000795 | Escherichia coli O157:H7 str. Sakai | NC_002695.1.915750.p01 | ECs4393 | 1.0E-77 | 99.22 | 128 | 2 |
| 3000795 | Escherichia coli O157:H7 str. Sakai | NC_002695.1.916587.p01 | ECs2885 | 2.0E-43 | 98.9 | 91 | 3 |
| 3000814 | Acinetobacter baumannii AB307-0294 | NC_011595.7059912.p01 | ABBFA_002603 | 2.0E-83 | 98.33 | 120 | 1 |
| 3000826 | Escherichia coli O157:H7 str. Sakai | NC_002695.1.912965.p01 | ECs2654 | < 1.0E-150 | 98.33 | 240 | 1 |
| 3000828 | Escherichia coli O157:H7 str. Sakai | NC_002695.1.916589.p01 | ABBFA_000579 | 5.0E-71 | 100 | 104 | 1 |
| 3000828 | Acinetobacter baumannii AB307-0294 | NC_011595.7057856.p01 | ECs2887 | 5.0E-69 | 94.17 | 109 | 2 |
| 3000829 | Shigella dysenteriae Sd197 | CP000034.1.gene2186.p01 | baeR | 1.0E-57 | 100 | 110 | 1 |
| 3000829 | Shigella dysenteriae Sd197 | CP000034.1.gene2187.p01 | baeS | 4.0E-61 | 100 | 119 | 1 |
| 3000829 | Escherichia coli O157:H7 str. Sakai | NC_002695.1.916588.p01 | ECs2886 | 1.0E-65 | 100 | 97 | 2 |
| 3000830 | Klebsiella pneumoniae subsp. pneumoniae MGH 78578 | CP000647.1.gene4256.p01 | cpxA | 4.0E-21 | 100 | 42 | 1 |
| 3000830 | Escherichia coli O157:H7 str. Sakai | NC_002695.1.914983.p01 | cpxA | 2.0E-42 | 100 | 77 | 3 |
| 3000832 | Klebsiella pneumoniae subsp. pneumoniae MGH 78578 | CP000647.1.gene3517.p01 | evgA | 2.0E-54 | 100 | 84 | 1 |
| 3000833 | Shigella dysenteriae Sd197 | CP000034.1.gene4478.p01 | evgS | < 1.0E-150 | 98.6 | 356 | 1 |
| 3000833 | Klebsiella pneumoniae subsp. pneumoniae MGH 78578 | CP000647.1.gene3518.p01 | ECs3249 | 7.0E-58 | 99.24 | 112 | 4 |
| 3000833 | Escherichia coli O157:H7 str. Sakai | NC_002695.1.915650.p01 | ECs3248 | 3.0E-69 | 97.19 | 112 | 3 |
| 3000833 | Escherichia coli O157:H7 str. Sakai | NC_002695.1.915651.p01 | evgS | 9.0E-120 | 100 | 204 | 1 |
| 3000834 | Shigella dysenteriae Sd197 | CP000034.1.gene2022.p01 | yejM | 5.0E-83 | 100 | 135 | 1 |
| 3000834 | Klebsiella pneumoniae subsp. pneumoniae MGH 78578 | CP000647.1.gene2625.p01 | phoP | 2.0E-67 | 100 | 109 | 2 |
| 3000835 | Shigella dysenteriae Sd197 | CP000034.1.gene2023.p01 | ECs1602 | 3.0E-43 | 98.67 | 75 | 1 |
| 3000835 | Klebsiella pneumoniae subsp. pneumoniae MGH 78578 | CP000647.1.gene1135.p01 | phoQ | 1.0E-99 | 100 | 146 | 1 |
| 3000835 | Escherichia coli O157:H7 str. Sakai | NC_002695.1.913289.p01 | phoQ | 1.0E-69 | 100 | 111 | 1 |
| 3000836 | Escherichia coli O157:H7 str. Sakai | NC_002695.1.914292.p01 | ECs5045 | 2.0E-70 | 100 | 114 | 1 |
| 3000837 | Escherichia coli O157:H7 str. Sakai | NC_002695.1.917670.p01 | ECs0930 | 2.0E-45 | 100 | 95 | 1 |
| 3000873 | Escherichia coli 1520 | NC_010558.1.6276043.p01 | blaTEM-1 | < 1.0E-150 | 100 | 286 | 1 |
| 3001121 | Klebsiella pneumoniae | DQ174306.1.gene1.p01 | blaSHV-66 | 9.0E-23 | 100 | 46 | 1 |
| 3001205 | Klebsiella pneumoniae subsp. pneumoniae MGH 78578 | CP000647.1.gene3367.p01 | blmA | 3.0E-14 | 91.43 | 35 | 2 |
| 3001205 | Campylobacter fetus subsp. fetus | FN594949.1.gene22.p01 | KPN_03367 | 2.0E-23 | 97.87 | 47 | 1 |
| 3001214 | Salmonella enterica subsp. enterica serovar Agona str. SL483 | CP001138.1.gene894.p01 | SeAg_B0902 | 5.0E-136 | 92.54 | 228 | 1 |
| 3001215 | Escherichia coli O157:H7 str. Sakai | NC_002695.1.915390.p01 | ECs4647 | < 1.0E-150 | 98.46 | 390 | 1 |
| 3001216 | Salmonella enterica subsp. enterica serovar Agona str. SL483 | CP001138.1.gene2007.p01 | SeAg_B2022 | 1.0E-33 | 100 | 98 | 1 |

**Matched high-throughput sequencing reads of sample C1757 against CARD**

(Sorted by ARO-number)

| **ARO** | **Organism** | **Accession number** | **Gene** | **E value ≤** | **Identity (%) ≥** | **Hit length (aa) ≥** | **Number of reads** |
| --- | --- | --- | --- | --- | --- | --- | --- |
| 3000014 | Proteus mirabilis | HM246246.1.gene1.p1 | ADM61585.1 | 8.0E-15 | 100 | 33 | 1 |
| 3000017 | Pseudomonas aeruginosa | AF024602.1.gene3.p01 | blaOXA-20 | 7.0E-15 | 96.97 | 33 | 2 |
| 3000017 | Pseudomonas aeruginosa | AF300985.1.gene1.p01 | blaOXA-2-related | 3.0E-14 | 96.97 | 33 | 8 |
| 3000017 | uncultured bacterium | AY139598.1.gene3.p01 | blaOXA | 5.0E-09 | 100 | 26 | 3 |
| 3000017 | Pseudomonas aeruginosa | U59183.1.gene3.p01 | blaOXA-13 | 4.0E-09 | 96.97 | 26 | 8 |
| 3000017 | Pseudomonas aeruginosa | X58272.1.gene1.p01 | blaOXA-5 | 1.0E-08 | 96.15 | 25 | 9 |
| 3000017 | Acinetobacter baumannii | HM488987.1.gene1.p1 | blaOXA-166 | 6.0E-14 | 90.91 | 33 | 1 |
| 3000017 | Burkholderia cepacia | AF371964.1.gene1.p1 | AAK55330.1 | 1.0E-12 | 93.94 | 33 | 6 |
| 3000017 | Klebsiella oxytoca | AY303807.gene.p01 | blaOXA-2b | 2.0E-11 | 90.91 | 33 | 2 |
| 3000017 | Klebsiella pneumoniae | M55547.gene.p01 | blaOXA-9 | 7.0E-14 | 100 | 33 | 2 |
| 3000017 | Pseudomonas aeruginosa | AF317511.1.gene5.p01 | blaOXA | 3.0E-09 | 90.91 | 26 | 2 |
| 3000017 | Pseudomonas aeruginosa | AJ854182.1.gene1.p01 | blaOXA-74 | 2.0E-14 | 100 | 33 | 1 |
| 3000017 | Pseudomonas aeruginosa | AY008291.1.gene1.p1 | blaOXA-1-like | 3.0E-15 | 100 | 33 | 1 |
| 3000017 | Pseudomonas aeruginosa | U37105.2.gene2.p01 | blaOXA-10 | 2.0E-07 | 90.91 | 25 | 14 |
| 3000017 | Pseudomonas aeruginosa | U63835.1.gene1.p01 | blaOXA-15 | 1.0E-08 | 100 | 27 | 3 |
| 3000017 | Salmonella enterica subsp. enterica serovar Bredeney | AM932669.1.gene2.p01 | blaOXA-129 | 9.0E-08 | 90.91 | 26 | 26 |
| 3000017 | Acinetobacter baumannii | EU220745.1.gene1.p01 | bla-OXA-117 | 6.0E-13 | 93.75 | 32 | 1 |
| 3000017 | Acinetobacter lwoffii | HQ122933.1.gene1.p01 | blaOXA-134a | 2.0E-12 | 93.94 | 31 | 4 |
| 3000017 | Klebsiella pneumoniae | AY237830.1.gene1.p01 | blaOXA-47 | 1.0E-14 | 100 | 33 | 1 |
| 3000017 | Pseudomonas aeruginosa | AF231133.1.gene3.p01 | blaOXA-28 | 7.0E-11 | 93.94 | 27 | 6 |
| 3000017 | Pseudomonas aeruginosa | JF800667.1.gene2.p01 | blaOXA-205 | 2.0E-12 | 93.94 | 33 | 10 |
| 3000019 | Pantoea agglomerans | HQ693810.1.gene2.p01 | blaACT-9 | 3.0E-08 | 90.32 | 31 | 1 |
| 3000019 | Escherichia coli O157:H7 str. Sakai | NC_002695.1.914045.p01 | ampC | 1.0E-12 | 93.94 | 31 | 11 |
| 3000019 | Acinetobacter baumannii ATCC 17978 | NC_009085.4920334.p01 | ampC | 6.0E-15 | 96.97 | 33 | 1 |
| 3000019 | Enterobacter cloacae subsp. cloacae ATCC 13047 | CP001918.1.gene3289.p01 | ampC | 9.0E-12 | 90.91 | 33 | 3 |
| 3000027 | Acinetobacter baumannii AYE | NC_010410.6003177.p01 | emrA | 6.0E-12 | 90.62 | 31 | 5 |
| 3000027 | Escherichia coli O157:H7 str. Sakai | NC_002695.1.914737.p01 | ECs3547 | 4.0E-11 | 90.91 | 31 | 9 |
| 3000027 | Klebsiella pneumoniae subsp. pneumoniae MGH 78578 | CP000647.1.gene3014.p01 | emrA | 3.0E-07 | 100 | 25 | 5 |
| 3000027 | Salmonella enterica subsp. enterica serovar Agona str. SL483 | CP001138.1.gene2905.p01 | emrA | 4.0E-12 | 93.94 | 33 | 2 |
| 3000054 | Klebsiella pneumoniae subsp. pneumoniae MGH 78578 | CP000647.1.gene444.p01 | acrA | 2.0E-09 | 96.43 | 28 | 9 |
| 3000054 | Escherichia coli O157:H7 str. Sakai | NC_002695.1.914620.p01 | ECs0516 | 4.0E-10 | 100 | 27 | 15 |
| 3000058 | Enterobacter cloacae | AY227752.1.gene1.p01 | blaMIR-2 | 4.0E-14 | 100 | 33 | 2 |
| 3000058 | Enterobacter cloacae | AY743435.1.gene1.p01 | blaMIR-3 | 3.0E-14 | 100 | 33 | 1 |
| 3000066 | Klebsiella pneumoniae | AY494718.1.gene1.p01 | blaGES-4 | 3.0E-14 | 100 | 33 | 2 |
| 3000066 | Pseudomonas aeruginosa | AF326355.1.gene1.p01 | blaGES-2 | 3.0E-07 | 100 | 25 | 6 |
| 3000066 | Pseudomonas aeruginosa | GU208678.1.gene1.p01 | blaGES-15 | 9.0E-11 | 100 | 28 | 4 |
| 3000071 | Actinoplanes teichomyceticus | AJ632270.1.orf8.gene.p01 | AJ632270.1.orf8 | 1.0E-06 | 93.75 | 32 | 1 |
| 3000071 | Clostridium difficile 630 | AM180355.1.gene1831.p01 | vanS | 4.0E-11 | 93.94 | 33 | 1 |
| 3000071 | Enterococcus faecium | AF130997.1.orf1.gene.p01 | vanSD | 1.0E-11 | 96.97 | 33 | 1 |
| 3000074 | Acinetobacter baumannii AYE | NC_010410.6003262.p01 | emrB | 1.0E-08 | 90.91 | 27 | 16 |
| 3000074 | Enterobacter cloacae subsp. cloacae ATCC 13047 | CP001918.1.gene2931.p01 | ECL_02910 | 3.0E-12 | 96.97 | 33 | 2 |
| 3000074 | Enterobacter cloacae subsp. cloacae ATCC 13047 | CP001918.1.gene4071.p01 | ECL_04023 | 3.0E-09 | 93.94 | 33 | 5 |
| 3000074 | Enterococcus faecalis V583 | AE016830.1.gene768.p01 | EF_0785 | 1.0E-11 | 90.91 | 33 | 3 |
| 3000074 | Escherichia coli O157:H7 str. Sakai | NC_002695.1.914736.p01 | ECs3548 | 2.0E-07 | 92 | 25 | 7 |
| 3000074 | Klebsiella pneumoniae subsp. pneumoniae MGH 78578 | CP000647.1.gene3015.p01 | emrB | 6.0E-07 | 93.94 | 31 | 10 |
| 3000074 | Salmonella enterica subsp. enterica serovar Agona str. SL483 | CP001138.1.gene2906.p01 | emrB | 2.0E-10 | 100 | 30 | 10 |
| 3000089 | Aeromonas hydrophila | U14748.1.gene2.p01 | blaAER-1 | 7.0E-13 | 100 | 32 | 1 |
| 3000091 | Acinetobacter calcoaceticus subsp. anitratus | AF135373.1.gene1.p01 | blaCARB-5 | 2.0E-12 | 100 | 33 | 5 |
| 3000091 | Oligella urethralis | AY178993.1.gene1.p01 | blaCARB-8 | 7.0E-13 | 100 | 33 | 1 |
| 3000092 | Pseudomonas aeruginosa | Z22590.1.gene1.p01 | blaZ | 9.0E-14 | 100 | 33 | 2 |
| 3000116 | Enterococcus faecium DO | CP003583.1.gene1365.p01 | HMPREF0351_11334 | 1.0E-13 | 100 | 33 | 1 |
| 3000122 | Acinetobacter baumannii AB0057 | NC_011586.7045146.p01 | AB57_3104 | 3.0E-15 | 90.91 | 33 | 3 |
| 3000122 | Acinetobacter baumannii AB307-0294 | NC_011595.7057747.p01 | ABBFA_002299 | 7.0E-13 | 90.62 | 32 | 10 |
| 3000122 | Acinetobacter baumannii AYE | NC_010410.6000796.p01 | cat | 5.0E-12 | 90.62 | 26 | 14 |
| 3000122 | Escherichia coli 1520 | NC_010558.1.6276004.p01 | catA1 | 2.0E-14 | 96.97 | 33 | 2 |
| 3000122 | Klebsiella pneumoniae subsp. pneumoniae MGH 78578 | CP000647.1.gene2018.p01 | KPN_02018 | 5.0E-10 | 90.91 | 28 | 5 |
| 3000122 | Klebsiella pneumoniae | AF322577.2.gene4.p01 | catB4 | 1.0E-09 | 100 | 25 | 2 |
| 3000126 | Enterococcus faecalis | V01547.1.orf0.gene.p01 | V01547.1.orf0 | 3.0E-12 | 100 | 29 | 13 |
| 3000126 | Escherichia coli | V00359.1.orf1.gene.p01 | V00359.1.orf1 | 3.0E-15 | 100 | 33 | 1 |
| 3000165 | Escherichia coli 1520 | NC_010558.1.6275971.p01 | tetA(B) | 2.0E-12 | 96.97 | 33 | 8 |
| 3000165 | Acinetobacter baumannii AYE | NC_010410.6002597.p01 | tetA | 4.0E-12 | 100 | 33 | 2 |
| 3000165 | Pseudomonas aeruginosa | X75761.gene.p01 | tetA | 1.0E-12 | 100 | 33 | 1 |
| 3000165 | Shigella sonnei | AF534183.gene.p01 | tetA | 1.0E-11 | 100 | 33 | 4 |
| 3000167 | Escherichia coli 1520 | NC_010558.1.6275977.p01 | tetC | 1.0E-11 | 100 | 30 | 2 |
| 3000167 | uncultured bacterium | AY171578.gene.p01 | tetC* | 2.0E-13 | 100 | 33 | 1 |
| 3000174 | Pseudomonas sp. | AF133139.gene.p01 | tetG | 2.0E-11 | 93.94 | 33 | 2 |
| 3000174 | Pseudomonas sp. | AF133140.gene.p01 | tetG | 1.0E-07 | 96.3 | 27 | 1 |
| 3000186 | Clostridium difficile 630 | AM180355.1.gene636.p01 | tetM | 1.0E-12 | 90.91 | 33 | 1 |
| 3000186 | Enterococcus faecalis | M85225.gene.p01 | tetM | 1.0E-09 | 90.91 | 25 | 20 |
| 3000186 | Enterococcus faecalis | X04388.gene.p01 | tetM | 1.0E-11 | 93.94 | 30 | 11 |
| 3000186 | Staphylococcus aureus subsp. aureus Mu50 | NC_002758.1120355.p01 | tetM | 4.0E-08 | 96.97 | 26 | 4 |
| 3000190 | Megasphaera elsdenii | AY485126.gene.p01 | tetOW | 8.0E-08 | 90.32 | 26 | 45 |
| 3000191 | Bacteroides fragilis | Z21523.gene.p01 | tetQ | 1.0E-06 | 93.33 | 25 | 31 |
| 3000192 | Listeria monocytogenes | L09756.gene.p01 | tetS* | 8.0E-12 | 100 | 31 | 4 |
| 3000194 | Bifidobacterium longum subsp. longum F8 | DQ294299.gene.p01 | tetW_2 | 2.0E-08 | 93.94 | 27 | 69 |
| 3000194 | Butyrivibrio fibrisolvens | AJ222769.gene.p01 | tetW* | 5.0E-07 | 90.91 | 25 | 225 |
| 3000195 | Clostridium perfringens | L20800.gene.p01 | tetBP | 1.0E-11 | 100 | 32 | 5 |
| 3000196 | Clostridiaceae bacterium K10 | AJ295238.gene.p01 | tet32 | 3.0E-07 | 90.62 | 25 | 120 |
| 3000205 | Bacteroides fragilis | M37699.gene1.p01 | tetX | 2.0E-07 | 100 | 25 | 3 |
| 3000207 | Enterobacter cloacae subsp. cloacae ATCC 13047 | CP001918.1.gene1258.p01 | acrA | 1.0E-11 | 100 | 33 | 1 |
| 3000207 | Escherichia coli O157:H7 str. Sakai | NC_002695.1.912781.p01 | ECs1863 | 9.0E-11 | 90.91 | 33 | 4 |
| 3000210 | Acinetobacter baumannii ATCC 17978 | NC_009085.4918494.p01 | rpoB | 2.0E-06 | 90.62 | 25 | 286 |
| 3000210 | Acinetobacter baumannii AYE | NC_010410.6003841.p01 | rpoB | 6.0E-11 | 90.91 | 33 | 3 |
| 3000210 | Acinetobacter baumannii SDF | NC_010400.5987325.p01 | rpoB | 9.0E-07 | 90.91 | 26 | 40 |
| 3000210 | Azoarcus sp. BH72 | NC_008702.1.4609796.p01 | rpoB | 6.0E-06 | 90 | 25 | 275 |
| 3000210 | Bordetella pertussis CS | CP002695.1.gene18.p01 | rpoB | 3.0E-06 | 90 | 25 | 410 |
| 3000210 | Clostridium botulinum A2 str. Kyoto | CP001581.1.gene3846.p01 | rpoB | 2.0E-06 | 90 | 25 | 138 |
| 3000210 | Clostridium difficile 630 | AM180355.1.gene120.p01 | rpoB | 4.0E-06 | 90 | 25 | 154 |
| 3000210 | Enterobacter cloacae subsp. cloacae ATCC 13047 | CP001918.1.gene250.p01 | rpoB | 3.0E-11 | 96.97 | 33 | 8 |
| 3000210 | Enterococcus faecalis V583 | AE016830.1.gene3155.p01 | rpoB | 5.0E-07 | 90.62 | 25 | 173 |
| 3000210 | Enterococcus faecium DO | CP003583.1.gene2745.p01 | rpoB | 7.0E-07 | 90.32 | 25 | 37 |
| 3000210 | Escherichia coli O157:H7 str. Sakai | NC_002695.1.914942.p01 | rpoB | 8.0E-08 | 90.32 | 25 | 27 |
| 3000210 | Klebsiella pneumoniae subsp. pneumoniae MGH 78578 | CP000647.1.gene4402.p01 | rpoB | 1.0E-10 | 90.62 | 32 | 16 |
| 3000210 | Legionella pneumophila str. Corby | CP000675.2.gene392.p01 | rpoB | 3.0E-07 | 90.32 | 27 | 75 |
| 3000210 | Mycobacterium tuberculosis CDC1551 | AE000516.2.gene708.p01 | rpoB | 6.0E-06 | 90.32 | 25 | 73 |
| 3000210 | Neisseria gonorrhoeae NCCP11945 | NC_011035.1.6448762.p01 | rpoB | 4.0E-06 | 90 | 25 | 320 |
| 3000210 | Proteus mirabilis BB2000 | CP004022.1.gene2794.p01 | rpoB | 2.0E-08 | 93.33 | 30 | 18 |
| 3000210 | Pseudomonas aeruginosa PAO1 | NC_002516.2.881699.p01 | rpoB | 5.0E-07 | 90 | 25 | 94 |
| 3000210 | Salmonella enterica subsp. enterica serovar Agona str. SL483 | CP001138.1.gene4362.p01 | rpoB | 2.0E-06 | 90 | 25 | 95 |
| 3000210 | Staphylococcus aureus subsp. aureus COL | NC_002951.3236234.p01 | rpoB | 8.0E-10 | 93.94 | 33 | 2 |
| 3000210 | Staphylococcus aureus subsp. aureus JH9 | NC_009487.5169226.p01 | rpoB | 6.0E-09 | 90.91 | 32 | 3 |
| 3000210 | Staphylococcus aureus subsp. aureus MRSA252 | NC_002952.2860169.p01 | rpoB | 8.0E-12 | 90.91 | 33 | 2 |
| 3000210 | Staphylococcus aureus subsp. aureus Mu50 | NC_002758.1120515.p01 | rpoB | 8.0E-08 | 90.91 | 25 | 2 |
| 3000210 | Staphylococcus aureus subsp. aureus N315 | NC_002745.1123305.p01 | rpoB | 2.0E-06 | 90.32 | 25 | 123 |
| 3000210 | Streptococcus pneumoniae Taiwan19F-14 | NC_012469.1.7686402.p01 | rpoB | 1.0E-06 | 90.62 | 25 | 136 |
| 3000216 | Shigella dysenteriae Sd197 | CP000034.1.gene457.p01 | acrB | 1.0E-07 | 90 | 30 | 15 |
| 3000216 | Klebsiella pneumoniae subsp. pneumoniae MGH 78578 | CP000647.1.gene443.p01 | acrB | 2.0E-06 | 92.59 | 27 | 26 |
| 3000216 | Escherichia coli O157:H7 str. Sakai | NC_002695.1.914619.p01 | ECs0515 | 2.0E-07 | 90 | 26 | 46 |
| 3000216 | Enterobacter cloacae subsp. cloacae ATCC 13047 | CP001918.1.gene1257.p01 | acrB | 5.0E-09 | 90.91 | 29 | 13 |
| 3000216 | Escherichia coli O157:H7 str. Sakai | NC_002695.1.912777.p01 | ECs1864 | 3.0E-06 | 90.32 | 26 | 47 |
| 3000216 | Bordetella pertussis CS | CP002695.1.gene992.p01 | acrB | 8.0E-08 | 90 | 30 | 19 |
| 3000225 | Campylobacter fetus subsp. fetus | FN594949.1.gene25.p01 | ant(6)-Ib | 5.0E-13 | 90.62 | 32 | 5 |
| 3000226 | Acinetobacter baumannii AB0057 | NC_011586.7045179.p01 | folP | 5.0E-11 | 93.75 | 29 | 3 |
| 3000226 | Acinetobacter baumannii AB0057 | NC_011586.7045208.p01 | folP | 6.0E-14 | 100 | 33 | 1 |
| 3000226 | Acinetobacter baumannii AYE | NC_010410.6003232.p01 | folP | 2.0E-11 | 90.91 | 33 | 1 |
| 3000226 | Bordetella pertussis CS | CP002695.1.gene1085.p01 | folP | 3.0E-13 | 93.75 | 32 | 1 |
| 3000226 | Escherichia coli O157:H7 str. Sakai | NC_002695.1.916103.p01 | folP | 2.0E-09 | 92.31 | 26 | 10 |
| 3000226 | Klebsiella pneumoniae subsp. pneumoniae MGH 78578 | CP000647.1.gene3624.p01 | folP | 1.0E-10 | 96.97 | 25 | 15 |
| 3000226 | Shigella dysenteriae Sd197 | CP000034.1.gene3358.p01 | folP | 1.0E-12 | 93.94 | 33 | 1 |
| 3000232 | Acinetobacter baumannii SDF | NC_010400.5986843.p01 | ABSDF0145 | 1.0E-12 | 100 | 33 | 2 |
| 3000232 | Escherichia coli 1520 | NC_010558.1.6275994.p01 | aadA4 | 7.0E-08 | 96.43 | 25 | 40 |
| 3000232 | Klebsiella pneumoniae | AJ704863.gene12.p01 | aadA1 | 7.0E-13 | 100 | 33 | 2 |
| 3000232 | Plasmid NR79 | AF047479.2.orf1.gene.p01 | aadA3 | 7.0E-15 | 100 | 33 | 1 |
| 3000232 | Pseudomonas aeruginosa | AF294653.1.gene3.p01 | aadA2a | 4.0E-12 | 90.91 | 32 | 6 |
| 3000232 | Pseudomonas aeruginosa | AJ584652.2.gene7.p01 | aadA1 | 1.0E-15 | 100 | 33 | 1 |
| 3000232 | Pseudomonas aeruginosa | U37105.2.gene4.p01 | aadA10 | 4.0E-10 | 90.62 | 25 | 44 |
| 3000232 | Pseudomonas aeruginosa | Y18050.2.gene6.p01 | aadA1 | 2.0E-14 | 100 | 33 | 2 |
| 3000232 | Salmonella enterica subsp. enterica serovar Stanley | EU118119.1.orf1.gene.p01 | aadA2 | 1.0E-12 | 90.91 | 33 | 1 |
| 3000232 | Salmonella enterica subsp. enterica serovar Typhi | AY123251.gene3.p01 | aadA1 | 3.0E-11 | 90.32 | 31 | 12 |
| 3000232 | Salmonella enterica | AJ628353.gene.p01 | aadA1b | 4.0E-12 | 96.97 | 32 | 19 |
| 3000232 | uncultured bacterium | AY139598.1.gene2.p01 | aadA5 | 3.0E-14 | 100 | 33 | 1 |
| 3000237 | Acinetobacter baumannii AB307-0294 | NC_011595.7058144.p01 | ABBFA_003277 | 2.0E-08 | 90 | 30 | 4 |
| 3000237 | Escherichia coli O157:H7 str. Sakai | NC_002695.1.916248.p01 | tolC | 2.0E-11 | 96.97 | 32 | 7 |
| 3000237 | Klebsiella pneumoniae subsp. pneumoniae MGH 78578 | CP000647.1.gene3449.p01 | tolC | 1.0E-07 | 93.94 | 25 | 11 |
| 3000237 | Salmonella enterica subsp. enterica serovar Agona str. SL483 | CP001138.1.gene3341.p01 | SeAg_B3367 | 6.0E-13 | 100 | 31 | 1 |
| 3000237 | Shigella dysenteriae Sd197 | CP000034.1.gene3205.p01 | tolC | 4.0E-07 | 100 | 25 | 3 |
| 3000252 | Enterobacter aerogenes | AF335467.1.gene1.p01 | omp36 | 3.0E-12 | 90.91 | 33 | 2 |
| 3000252 | Enterobacter aerogenes | AF336095.1.gene1.p01 | omp36 | 1.0E-11 | 93.94 | 32 | 3 |
| 3000252 | Enterobacter aerogenes | AF336096.1.gene1.p01 | omp36 | 1.0E-09 | 90.62 | 32 | 4 |
| 3000252 | Enterobacter aerogenes | AF336098.1.gene1.p01 | omp36 | 4.0E-13 | 100 | 33 | 1 |
| 3000263 | Escherichia coli O157:H7 str. Sakai | NC_002695.1.917339.p01 | ECs2138 | 5.0E-10 | 93.33 | 25 | 3 |
| 3000263 | Klebsiella pneumoniae subsp. pneumoniae MGH 78578 | CP000647.1.gene1624.p01 | marA | 3.0E-14 | 100 | 32 | 2 |
| 3000263 | Shigella dysenteriae Sd197 | CP000034.1.gene1596.p01 | marA | 8.0E-13 | 96.43 | 28 | 1 |
| 3000264 | Azoarcus sp. BH72 | NC_008702.1.4608023.p01 | emrE | 7.0E-10 | 90 | 30 | 1 |
| 3000264 | Escherichia coli O157:H7 str. Sakai | NC_002695.1.913273.p01 | emrE | 2.0E-11 | 100 | 28 | 4 |
| 3000275 | Enterococcus faecium | AF110130.1.orf0.gene.p01 | linB | 2.0E-06 | 93.94 | 25 | 32 |
| 3000300 | Staphylococcus sciuri | AJ579365.gene.p01 | lsaB_orf3 | 7.0E-11 | 90.62 | 32 | 2 |
| 3000309 | Escherichia coli O157:H7 str. Sakai | NC_002695.1.915420.p01 | emrD | 2.0E-12 | 100 | 32 | 8 |
| 3000309 | Klebsiella pneumoniae subsp. pneumoniae MGH 78578 | CP000647.1.gene4115.p01 | emrD | 6.0E-07 | 90.62 | 26 | 10 |
| 3000316 | Escherichia coli | D16251.1.orf0.gene.p01 | mphA | 3.0E-13 | 100 | 33 | 3 |
| 3000318 | Staphylococcus aureus | AB013298.gene.p01 | mphBM | 1.0E-15 | 100 | 33 | 1 |
| 3000322 | Plasmid pWP14a | X13542.gene.p01 | aac(3)-III | 2.0E-11 | 100 | 30 | 1 |
| 3000361 | Escherichia coli | AY183453.gene.p01 | ereA3 | 1.0E-12 | 96.97 | 33 | 5 |
| 3000373 | Escherichia coli O157:H7 str. Sakai | NC_002695.1.915653.p01 | ECs3247 | 9.0E-12 | 93.94 | 33 | 10 |
| 3000373 | Shigella dysenteriae Sd197 | CP000034.1.gene2563.p01 | emrK | 4.0E-12 | 100 | 33 | 1 |
| 3000375 | Clostridium difficile 630 | AM180355.1.gene2254.p01 | ermB | 7.0E-08 | 96.88 | 26 | 30 |
| 3000375 | Streptococcus pneumoniae | AM410044.gene14.p01 | ermB | 3.0E-07 | 93.1 | 25 | 193 |
| 3000377 | Pseudomonas aeruginosa PAO1 | NC_002516.2.877855.p01 | mexA | 2.0E-09 | 90.62 | 32 | 1 |
| 3000378 | Acinetobacter baumannii AB307-0294 | NC_011595.7057907.p01 | ABBFA_000816 | 8.0E-07 | 90 | 26 | 16 |
| 3000378 | Pseudomonas aeruginosa PAO1 | NC_002516.2.877852.p01 | mexB | 3.0E-06 | 90.32 | 25 | 35 |
| 3000379 | Acinetobacter baumannii AB307-0294 | NC_011595.7058890.p01 | ABBFA_003020 | 2.0E-08 | 90.32 | 28 | 16 |
| 3000390 | Streptomyces coelicolor A3(2) | AL939114.1.orf1.gene.p01 | SCO2860 | 6.0E-08 | 90.62 | 25 | 3 |
| 3000410 | Citrobacter freundii | AY162283.2.gene7.p01 | sul1 | 1.0E-10 | 100 | 27 | 5 |
| 3000410 | Klebsiella pneumoniae | AF322577.2.gene6.p01 | sul1 | 1.0E-13 | 100 | 33 | 1 |
| 3000410 | Klebsiella pneumoniae | AJ704863.gene21.p01 | partial sul1 | 1.0E-13 | 100 | 33 | 3 |
| 3000410 | Klebsiella pneumoniae | DQ143913.1.gene6.p01 | sul1 | 2.0E-13 | 96.97 | 33 | 2 |
| 3000410 | Pseudomonas aeruginosa | AF191564.1.gene5.p01 | sul1 | 2.0E-13 | 100 | 33 | 1 |
| 3000410 | Pseudomonas aeruginosa | U37105.2.gene6.p01 | sul1 | 2.0E-09 | 96.97 | 28 | 11 |
| 3000412 | Escherichia coli | DQ464881.1.gene2.p01 | sul2 | 1.0E-07 | 96.3 | 27 | 15 |
| 3000445 | Rhodococcus equi | U56415.1.orf0.gene.p01 | iri | 2.0E-08 | 100 | 25 | 1 |
| 3000453 | Clostridium difficile 630 | AM180355.1.gene2499.p01 | sat | 3.0E-13 | 90.62 | 32 | 1 |
| 3000457 | Acinetobacter baumannii AYE | NC_010410.6000336.p01 | parE | 6.0E-08 | 90.91 | 25 | 6 |
| 3000457 | Acinetobacter baumannii SDF | NC_010400.5986295.p01 | parE | 1.0E-06 | 90.32 | 25 | 83 |
| 3000457 | Azoarcus sp. BH72 | NC_008702.1.4609137.p01 | parE | 2.0E-08 | 90.91 | 27 | 11 |
| 3000457 | Bordetella pertussis CS | CP002695.1.gene1273.p01 | parE | 4.0E-07 | 90 | 25 | 162 |
| 3000457 | Clostridium botulinum A2 str. Kyoto | CP001581.1.gene3049.p01 | CLM_3138 | 2.0E-11 | 90.32 | 31 | 1 |
| 3000457 | Enterococcus faecalis V583 | AE016830.1.gene1599.p01 | parE | 4.0E-08 | 90.62 | 27 | 35 |
| 3000457 | Enterococcus faecium DO | CP003583.1.gene1173.p01 | parE | 2.0E-07 | 90 | 25 | 25 |
| 3000457 | Klebsiella pneumoniae subsp. pneumoniae MGH 78578 | CP000647.1.gene3444.p01 | parE | 2.0E-11 | 96.97 | 29 | 13 |
| 3000457 | Legionella pneumophila str. Corby | CP000675.2.gene802.p01 | parE | 4.0E-09 | 90.32 | 31 | 1 |
| 3000457 | Proteus mirabilis BB2000 | CP004022.1.gene2481.p01 | parE | 8.0E-09 | 90 | 30 | 5 |
| 3000457 | Pseudomonas aeruginosa PAO1 | NC_002516.2.879897.p01 | parE | 4.0E-08 | 90.62 | 28 | 6 |
| 3000457 | Salmonella enterica subsp. enterica serovar Agona str. SL483 | CP001138.1.gene3336.p01 | parE | 4.0E-10 | 96.55 | 29 | 10 |
| 3000457 | Shigella dysenteriae Sd197 | CP000034.1.gene3210.p01 | parE | 5.0E-10 | 93.94 | 28 | 19 |
| 3000457 | Staphylococcus aureus RF122 | NC_007622.3794232.p01 | grlB | 8.0E-11 | 93.94 | 33 | 1 |
| 3000457 | Staphylococcus aureus subsp. aureus COL | NC_002951.3236245.p01 | parE | 5.0E-11 | 90.91 | 31 | 3 |
| 3000457 | Staphylococcus aureus subsp. aureus MRSA252 | NC_002952.2859942.p01 | grlB | 4.0E-09 | 90 | 30 | 4 |
| 3000457 | Staphylococcus aureus subsp. aureus N315 | NC_002745.1124025.p01 | parE | 1.0E-06 | 90.62 | 25 | 21 |
| 3000457 | Streptococcus pneumoniae Taiwan19F-14 | NC_012469.1.7686068.p01 | parE | 1.0E-06 | 90.62 | 25 | 37 |
| 3000461 | Clostridium botulinum A2 str. Kyoto | CP001581.1.gene3143.p01 | CLM_3236 | 2.0E-08 | 90.91 | 26 | 13 |
| 3000479 | Streptomyces caeruleus | AF205854.1.orf0.gene.p01 | gyrB-Rn | 3.0E-11 | 93.55 | 31 | 3 |
| 3000479 | Streptomyces roseochromogenes subsp. oscitans | AF329398.1.orf36.gene.p01 | gyrBR | 5.0E-09 | 90 | 30 | 4 |
| 3000480 | Streptomyces rishiriensis | AF205853.1.orf1.gene.p01 | parYR | 2.0E-09 | 90.91 | 30 | 5 |
| 3000480 | Streptomyces roseochromogenes subsp. oscitans | AY136281.1.orf0.gene.p01 | parYR | 1.0E-10 | 90.62 | 32 | 8 |
| 3000489 | Staphylococcus aureus subsp. aureus Mu50 | NC_002758.1121879.p01 | SAV1866 | 6.0E-11 | 93.75 | 32 | 1 |
| 3000491 | Escherichia coli O157:H7 str. Sakai | NC_002695.1.915267.p01 | ECs3332 | 3.0E-07 | 90.91 | 28 | 22 |
| 3000491 | Klebsiella pneumoniae subsp. pneumoniae MGH 78578 | CP000647.1.gene2803.p01 | acrD | 1.0E-07 | 90 | 27 | 27 |
| 3000491 | Shigella dysenteriae Sd197 | CP000034.1.gene2654.p01 | acrD | 2.0E-12 | 100 | 33 | 7 |
| 3000491 | Salmonella enterica subsp. enterica serovar Agona str. SL483 | CP001138.1.gene2601.p01 | acrD | 9.0E-09 | 90.91 | 32 | 15 |
| 3000493 | Escherichia coli | GQ465831.1.gene2.p01 | ompF | 1.0E-10 | 100 | 29 | 9 |
| 3000493 | Klebsiella pneumoniae subsp. pneumoniae MGH 78578 | CP000647.1.gene2517.p01 | asmA | 4.0E-11 | 93.94 | 33 | 10 |
| 3000493 | Klebsiella pneumoniae subsp. pneumoniae MGH 78578 | CP000647.1.gene956.p01 | ompF | 1.0E-06 | 96.77 | 31 | 5 |
| 3000493 | Shigella dysenteriae Sd197 | CP000034.1.gene2198.p01 | asmA | 7.0E-08 | 93.94 | 26 | 13 |
| 3000493 | Shigella dysenteriae Sd197 | CP000034.1.gene2328.p01 | ompF | 6.0E-12 | 100 | 30 | 1 |
| 3000493 | Shigella dysenteriae Sd197 | CP000034.1.gene3671.p01 | ompR | 9.0E-09 | 96.97 | 26 | 15 |
| 3000493 | Shigella dysenteriae Sd197 | CP000034.1.gene3672.p01 | envZ | 4.0E-09 | 90.62 | 26 | 32 |
| 3000498 | Bacteroides fragilis | M14730.gene.p01 | ermF* | 5.0E-10 | 90.62 | 27 | 9 |
| 3000499 | Escherichia coli O157:H7 str. Sakai | NC_002695.1.916015.p01 | ECs4137 | 4.0E-07 | 96 | 25 | 14 |
| 3000499 | Klebsiella pneumoniae subsp. pneumoniae MGH 78578 | CP000647.1.gene3709.p01 | acrE | 2.0E-07 | 90.32 | 25 | 6 |
| 3000502 | Klebsiella pneumoniae subsp. pneumoniae MGH 78578 | CP000647.1.gene3710.p01 | acrF | 4.0E-06 | 90 | 25 | 57 |
| 3000506 | Azoarcus sp. BH72 | NC_008702.1.4610136.p01 | mexR | 4.0E-09 | 100 | 25 | 1 |
| 3000508 | Escherichia coli O157:H7 str. Sakai | NC_002695.1.915747.p01 | ECs4396 | 1.0E-12 | 93.94 | 29 | 6 |
| 3000516 | Acinetobacter baumannii AB307-0294 | NC_011595.7058564.p01 | ABBFA_001502 | 1.0E-11 | 90.91 | 33 | 1 |
| 3000516 | Klebsiella pneumoniae subsp. pneumoniae MGH 78578 | CP000647.1.gene3013.p01 | emrR | 2.0E-14 | 100 | 33 | 3 |
| 3000516 | Shigella dysenteriae Sd197 | CP000034.1.gene2879.p01 | emrR | 5.0E-14 | 93.94 | 33 | 8 |
| 3000518 | Klebsiella pneumoniae subsp. pneumoniae MGH 78578 | CP000647.1.gene3780.p01 | crp | 5.0E-14 | 100 | 33 | 2 |
| 3000518 | Proteus mirabilis BB2000 | CP004022.1.gene2827.p01 | crp | 6.0E-12 | 90.91 | 29 | 4 |
| 3000518 | Shigella dysenteriae Sd197 | CP000034.1.gene3519.p01 | crp | 6.0E-08 | 96.97 | 26 | 13 |
| 3000522 | Lysinibacillus sphaericus | M15332.gene.p01 | erm(G)**_ermG* | 4.0E-12 | 90.91 | 33 | 4 |
| 3000533 | Acinetobacter baumannii AB307-0294 | NC_011595.7058613.p01 | ABBFA_003018 | 1.0E-07 | 90.32 | 25 | 23 |
| 3000533 | Azoarcus sp. BH72 | NC_008702.1.4609191.p01 | macA | 6.0E-13 | 90.32 | 31 | 1 |
| 3000533 | Enterobacter cloacae subsp. cloacae ATCC 13047 | CP001918.1.gene2796.p01 | ECL_02771 | 1.0E-12 | 100 | 33 | 2 |
| 3000533 | Escherichia coli O157:H7 str. Sakai | NC_002695.1.917702.p01 | ECs0964 | 2.0E-10 | 93.94 | 28 | 15 |
| 3000535 | Acinetobacter baumannii AB307-0294 | NC_011595.7060505.p01 | ABBFA_003019 | 9.0E-06 | 90 | 25 | 57 |
| 3000535 | Acinetobacter baumannii SDF | NC_010400.5985985.p01 | macB | 1.0E-08 | 92.86 | 28 | 3 |
| 3000535 | Azoarcus sp. BH72 | NC_008702.1.4606596.p01 | azo0832 | 3.0E-07 | 90 | 30 | 1 |
| 3000535 | Azoarcus sp. BH72 | NC_008702.1.4606597.p01 | azo0833 | 3.0E-09 | 93.94 | 33 | 1 |
| 3000535 | Azoarcus sp. BH72 | NC_008702.1.4606598.p01 | azo0834 | 1.0E-12 | 93.94 | 33 | 1 |
| 3000535 | Azoarcus sp. BH72 | NC_008702.1.4609454.p01 | macB | 9.0E-06 | 92.59 | 26 | 6 |
| 3000535 | Clostridium botulinum A2 str. Kyoto | CP001581.1.gene598.p01 | CLM_0622 | 2.0E-06 | 90.62 | 25 | 25 |
| 3000535 | Clostridium botulinum A2 str. Kyoto | CP001581.1.gene798.p01 | CLM_0827 | 3.0E-10 | 90.62 | 28 | 34 |
| 3000535 | Klebsiella pneumoniae subsp. pneumoniae MGH 78578 | CP000647.1.gene911.p01 | macA | 1.0E-07 | 100 | 26 | 5 |
| 3000535 | Klebsiella pneumoniae subsp. pneumoniae MGH 78578 | CP000647.1.gene912.p01 | macB | 9.0E-07 | 90 | 25 | 17 |
| 3000535 | Listeria monocytogenes | HE999704.1.gene196.p01 | BN418_0205 | 4.0E-08 | 90 | 27 | 2 |
| 3000535 | Proteus mirabilis BB2000 | CP004022.1.gene758.p01 | macB | 3.0E-09 | 93.1 | 29 | 2 |
| 3000535 | Salmonella enterica subsp. enterica serovar Agona str. SL483 | CP001138.1.gene935.p01 | SeAg_B0943 | 7.0E-11 | 90.91 | 31 | 3 |
| 3000535 | Streptococcus pneumoniae Taiwan19F-14 | NC_012469.1.7685735.p01 | SPT_1593 | 2.0E-10 | 90.32 | 28 | 14 |
| 3000535 | Streptococcus pneumoniae Taiwan19F-14 | NC_012469.1.7686878.p01 | SPT_1414 | 1.0E-11 | 90.62 | 32 | 4 |
| 3000549 | Acinetobacter baumannii AB307-0294 | NC_011595.7060199.p01 | ABBFA_001705 | 8.0E-15 | 100 | 33 | 1 |
| 3000549 | Acinetobacter baumannii ATCC 17978 | NC_009085.4919121.p01 | A1S_1754 | 7.0E-14 | 100 | 33 | 1 |
| 3000556 | Campylobacter fetus subsp. fetus | FN594949.1.gene24.p01 | tet44 | 6.0E-08 | 90.91 | 27 | 7 |
| 3000559 | Shigella dysenteriae Sd197 | CP000034.1.gene1340.p01 | btuR | 2.0E-12 | 90.91 | 32 | 4 |
| 3000566 | Acinetobacter sp. LUH5605 | AY743590.gene.p01 | tet39 | 2.0E-06 | 100 | 26 | 18 |
| 3000574 | Acinetobacter baumannii AB307-0294 | NC_011595.7059276.p01 | ABBFA_002430 | 1.0E-09 | 90.62 | 28 | 17 |
| 3000574 | Clostridium difficile 630 | AM180355.1.gene1830.p01 | vanR | 2.0E-10 | 100 | 26 | 2 |
| 3000574 | Enterococcus faecalis | DQ212986.1.gene4.p01 | vanRG | 2.0E-14 | 93.94 | 33 | 1 |
| 3000574 | Enterococcus gallinarum | AF162694.1.orf4.gene.p01 | vanRc | 8.0E-12 | 90 | 30 | 2 |
| 3000574 | Streptomyces toyocaensis | U82965.2.orf14.gene.p01 | U82965.2.orf14 | 8.0E-12 | 93.75 | 32 | 2 |
| 3000581 | Bordetella pertussis CS | CP002695.1.gene1737.p01 | cphA | 1.0E-06 | 92.31 | 26 | 2 |
| 3000581 | Bordetella pertussis CS | CP002695.1.gene1738.p01 | cphA | 3.0E-10 | 90.91 | 33 | 1 |
| 3000616 | Streptococcus pneumoniae Taiwan19F-14 | NC_012469.1.7685970.p01 | SPT_1925 | 1.0E-06 | 90.62 | 25 | 73 |
| 3000618 | Acinetobacter baumannii AB307-0294 | NC_011595.7058445.p01 | gyrA | 7.0E-06 | 90.62 | 25 | 129 |
| 3000618 | Acinetobacter baumannii AYE | NC_010410.6003186.p01 | gyrA | 8.0E-11 | 90.32 | 31 | 8 |
| 3000618 | Acinetobacter baumannii SDF | NC_010400.5986734.p01 | gyrA | 3.0E-09 | 90.32 | 30 | 12 |
| 3000618 | Azoarcus sp. BH72 | NC_008702.1.4606680.p01 | gyrA | 2.0E-06 | 90 | 25 | 159 |
| 3000618 | Bordetella pertussis CS | CP002695.1.gene952.p01 | gyrA | 4.0E-06 | 90 | 25 | 270 |
| 3000618 | Campylobacter jejuni subsp. doylei 269.97 | CP000768.1.gene728.p01 | gyrA | 2.0E-12 | 90.91 | 33 | 1 |
| 3000618 | Clostridium botulinum A2 str. Kyoto | CP001581.1.gene7.p01 | gyrA | 4.0E-08 | 90 | 25 | 18 |
| 3000618 | Clostridium difficile 630 | AM180355.1.gene6.p01 | gyrA | 3.0E-09 | 90.32 | 28 | 22 |
| 3000618 | Enterococcus faecalis V583 | AE016830.1.gene6.p01 | gyrA | 5.0E-06 | 90.62 | 25 | 41 |
| 3000618 | Escherichia coli O157:H7 str. Sakai | NC_002695.1.916822.p01 | ECs3114 | 4.0E-10 | 90 | 30 | 21 |
| 3000618 | Haemophilus influenzae 10810 | FQ312006.1.gene1417.p01 | HIB_14190 | 6.0E-10 | 96.55 | 29 | 1 |
| 3000618 | Helicobacter pylori Gambia94/24 | CP002332.1.gene704.p01 | HPGAM_03615 | 6.0E-08 | 90.91 | 25 | 5 |
| 3000618 | Klebsiella pneumoniae subsp. pneumoniae MGH 78578 | CP000647.1.gene2640.p01 | gyrA | 1.0E-08 | 93.94 | 29 | 21 |
| 3000618 | Legionella pneumophila str. Corby | CP000675.2.gene1514.p01 | gyrA | 4.0E-10 | 90 | 30 | 8 |
| 3000618 | Listeria monocytogenes | HE999704.1.gene7.p01 | BN418_0007 | 4.0E-09 | 90.32 | 31 | 4 |
| 3000618 | Mycobacterium tuberculosis CDC1551 | AE000516.2.gene6.p01 | gyrA | 1.0E-10 | 90.32 | 31 | 5 |
| 3000618 | Neisseria gonorrhoeae NCCP11945 | NC_011035.1.6447337.p01 | NGK_1285 | 4.0E-09 | 90 | 29 | 23 |
| 3000618 | Proteus mirabilis BB2000 | CP004022.1.gene1837.p01 | gyrA | 1.0E-09 | 90.91 | 33 | 3 |
| 3000618 | Pseudomonas aeruginosa PAO1 | NC_002516.2.882800.p01 | gyrA | 2.0E-07 | 90.91 | 26 | 17 |
| 3000618 | Salmonella enterica subsp. enterica serovar Agona str. SL483 | CP001138.1.gene2385.p01 | gyrA | 3.0E-06 | 92 | 25 | 8 |
| 3000618 | Staphylococcus aureus subsp. aureus COL | NC_002951.3236187.p01 | gyrA | 7.0E-12 | 90.91 | 33 | 2 |
| 3000618 | Staphylococcus aureus subsp. aureus MRSA252 | NC_002952.2859949.p01 | gyrA | 5.0E-12 | 90.91 | 33 | 1 |
| 3000618 | Staphylococcus aureus subsp. aureus Mu50 | NC_002758.1119966.p01 | gyrA | 7.0E-12 | 93.94 | 33 | 1 |
| 3000618 | Staphylococcus aureus subsp. aureus N315 | NC_002745.1122777.p01 | gyrA | 4.0E-08 | 90 | 30 | 3 |
| 3000618 | Staphylococcus aureus subsp. aureus str. Newman | NC_009641.5331984.p01 | gyrA | 4.0E-13 | 96.88 | 32 | 1 |
| 3000618 | Staphylococcus epidermidis ATCC 12228 | AE015929.1.gene5.p01 | SE_0005 | 3.0E-07 | 90.32 | 25 | 8 |
| 3000618 | Streptococcus pneumoniae Taiwan19F-14 | NC_012469.1.7686721.p01 | gyrA | 5.0E-09 | 90.62 | 28 | 42 |
| 3000618 | Vibrio cholerae MJ-1236 | CP001485.1.gene2164.p01 | VCD_003093 | 1.0E-08 | 90.91 | 30 | 9 |
| 3000618 | Yersinia pestis Antiqua | CP000308.1.gene965.p01 | YPA_0930 | 1.0E-08 | 90.32 | 26 | 9 |
| 3000619 | Acinetobacter baumannii ACICU | NC_010611.6237080.p01 | parC | 3.0E-11 | 90.91 | 33 | 2 |
| 3000619 | Acinetobacter baumannii AYE | NC_010410.6003198.p01 | parC | 3.0E-06 | 90.91 | 25 | 4 |
| 3000619 | Acinetobacter baumannii SDF | NC_010400.5984045.p01 | parC | 3.0E-06 | 90.91 | 25 | 57 |
| 3000619 | Bordetella pertussis CS | CP002695.1.gene1275.p01 | parC | 2.0E-09 | 90 | 29 | 18 |
| 3000619 | Enterococcus faecalis V583 | AE016830.1.gene1598.p01 | parC | 2.0E-08 | 90 | 27 | 11 |
| 3000619 | Enterococcus faecium DO | CP003583.1.gene1174.p01 | parC | 3.0E-09 | 90.91 | 28 | 5 |
| 3000619 | Escherichia coli str. K-12 substr. W3110 | M58408.gene.p01 | parC | 5.0E-06 | 90.91 | 25 | 18 |
| 3000619 | Klebsiella pneumoniae subsp. pneumoniae MGH 78578 | CP000647.1.gene3437.p01 | parC | 3.0E-08 | 96.97 | 25 | 11 |
| 3000619 | Legionella pneumophila str. Corby | CP000675.2.gene3231.p01 | parC | 2.0E-06 | 90.91 | 27 | 4 |
| 3000619 | Pseudomonas aeruginosa PAO1 | NC_002516.2.879741.p01 | parC | 6.0E-08 | 90 | 28 | 11 |
| 3000619 | Salmonella enterica subsp. enterica serovar Agona str. SL483 | CP001138.1.gene3329.p01 | parC | 4.0E-06 | 90.91 | 25 | 14 |
| 3000619 | Shigella dysenteriae Sd197 | CP000034.1.gene3218.p01 | parC | 6.0E-10 | 93.75 | 28 | 4 |
| 3000619 | Staphylococcus aureus subsp. aureus N315 | NC_002745.1124026.p01 | parC | 2.0E-08 | 92.59 | 27 | 3 |
| 3000619 | Streptococcus pneumoniae Taiwan19F-14 | NC_012469.1.7685406.p01 | parC | 8.0E-09 | 90.91 | 27 | 27 |
| 3000621 | Acinetobacter baumannii AB0057 | NC_011586.7045516.p01 | AB57_0437 | 9.0E-09 | 90 | 30 | 14 |
| 3000621 | Acinetobacter baumannii AB0057 | NC_011586.7045804.p01 | AB57_2380 | 1.0E-14 | 90.91 | 33 | 1 |
| 3000621 | Aeromonas sobria | U10251.1.gene1.p01 | blaZ | 2.0E-13 | 96.97 | 33 | 2 |
| 3000621 | Azoarcus sp. BH72 | NC_008702.1.4608898.p01 | azo0443 | 8.0E-09 | 90.62 | 26 | 9 |
| 3000621 | Klebsiella pneumoniae | AY034848.1.gene1.p01 | blaFOX-6 | 3.0E-12 | 96.88 | 32 | 1 |
| 3000621 | Klebsiella pneumoniae | JF896803.1.gene1.p01 | blaFOX-9 | 4.0E-12 | 90.91 | 33 | 2 |
| 3000621 | Staphylococcus aureus subsp. aureus USA300_TCH1516 | NC_010079.5775899.p01 | USA300HOU_1207 | 1.0E-11 | 93.94 | 33 | 1 |
| 3000621 | Staphylococcus aureus | NC_010419.6155842.p01 | pTZ2162_35 | 4.0E-16 | 100 | 33 | 1 |
| 3000656 | Escherichia coli O157:H7 str. Sakai | NC_002695.1.916016.p01 | ECs4136 | 4.0E-09 | 93.94 | 25 | 7 |
| 3000656 | Klebsiella pneumoniae subsp. pneumoniae MGH 78578 | CP000647.1.gene3708.p01 | envR | 3.0E-07 | 90.91 | 25 | 4 |
| 3000662 | Escherichia coli O157:H7 str. Sakai | NC_002695.1.912474.p01 | ECs1443 | 7.0E-08 | 96.3 | 27 | 8 |
| 3000676 | Acinetobacter baumannii AB0057 | NC_011586.7046013.p01 | AB57_0355 | 1.0E-11 | 90.91 | 28 | 5 |
| 3000676 | Escherichia coli O157:H7 str. Sakai | NC_002695.1.913113.p01 | ECs1739 | 2.0E-14 | 100 | 33 | 3 |
| 3000676 | Salmonella enterica subsp. enterica serovar Agona str. SL483 | CP001138.1.gene1383.p01 | hns | 5.0E-14 | 96.97 | 33 | 1 |
| 3000702 | Klebsiella pneumoniae subsp. pneumoniae MGH 78578 | CP000647.1.gene445.p01 | acrR | 8.0E-10 | 96.97 | 31 | 15 |
| 3000702 | Shigella dysenteriae Sd197 | CP000034.1.gene455.p01 | acrR | 2.0E-13 | 100 | 33 | 9 |
| 3000718 | Acinetobacter baumannii ATCC 19606 | NC_006877.3293011.p01 | marR | 5.0E-11 | 93.75 | 28 | 13 |
| 3000718 | Klebsiella pneumoniae subsp. pneumoniae MGH 78578 | CP000647.1.gene1252.p01 | KPN_01252 | 4.0E-12 | 90.91 | 33 | 2 |
| 3000718 | Klebsiella pneumoniae subsp. pneumoniae MGH 78578 | CP000647.1.gene1625.p01 | marR | 2.0E-09 | 96.97 | 25 | 6 |
| 3000718 | Klebsiella pneumoniae subsp. pneumoniae MGH 78578 | CP000647.1.gene1851.p01 | KPN_01851 | 5.0E-10 | 93.94 | 29 | 6 |
| 3000718 | Klebsiella pneumoniae subsp. pneumoniae MGH 78578 | CP000647.1.gene3283.p01 | KPN_03283 | 5.0E-13 | 93.94 | 30 | 3 |
| 3000718 | Klebsiella pneumoniae subsp. pneumoniae MGH 78578 | CP000647.1.gene4798.p01 | KPN_04761 | 1.0E-11 | 93.94 | 32 | 5 |
| 3000718 | Shigella dysenteriae Sd197 | CP000034.1.gene1597.p01 | marR | 7.0E-12 | 96.97 | 30 | 5 |
| 3000753 | Acinetobacter baumannii AB0057 | NC_011586.7045550.p01 | abeM | 2.0E-06 | 90.62 | 26 | 12 |
| 3000774 | Acinetobacter baumannii ATCC 17978 | NC_009085.4919119.p01 | A1S_1752 | 3.0E-14 | 93.94 | 33 | 1 |
| 3000774 | Acinetobacter baumannii SDF | NC_010400.5984386.p01 | ABSDF0738 | 1.0E-06 | 93.75 | 25 | 5 |
| 3000775 | Acinetobacter baumannii ATCC 17978 | NC_009085.4919117.p01 | A1S_1750 | 2.0E-09 | 90.91 | 32 | 3 |
| 3000776 | Acinetobacter baumannii SDF | NC_010400.5984384.p01 | ABSDF0736 | 1.0E-10 | 90.32 | 31 | 1 |
| 3000777 | Acinetobacter baumannii SDF | NC_010400.5984910.p01 | ABSDF1463 | 1.0E-11 | 96.97 | 33 | 2 |
| 3000778 | Acinetobacter baumannii SDF | NC_010400.5984909.p01 | ABSDF1462 | 1.0E-07 | 90.91 | 26 | 11 |
| 3000780 | Acinetobacter baumannii ATCC 17978 | NC_009085.4918693.p01 | A1S_2735 | 5.0E-06 | 90.32 | 25 | 7 |
| 3000781 | Acinetobacter baumannii AB0057 | NC_011586.7045444.p01 | adeJ | 4.0E-06 | 90 | 25 | 79 |
| 3000782 | Acinetobacter baumannii AB0057 | NC_011586.7045445.p01 | adeK | 6.0E-07 | 90.91 | 25 | 9 |
| 3000782 | Acinetobacter baumannii ATCC 17978 | NC_009085.4918695.p01 | A1S_2737 | 8.0E-07 | 90.91 | 25 | 9 |
| 3000792 | Enterobacter cloacae subsp. cloacae ATCC 13047 | CP001918.1.gene3439.p01 | ECL_03401 | 2.0E-10 | 90.62 | 32 | 5 |
| 3000792 | Escherichia coli O157:H7 str. Sakai | NC_002695.1.916584.p01 | ECs2882 | 2.0E-07 | 90.62 | 25 | 15 |
| 3000792 | Salmonella enterica subsp. enterica serovar Agona str. SL483 | CP001138.1.gene2234.p01 | SeAg_B2256 | 4.0E-12 | 100 | 33 | 1 |
| 3000793 | Enterobacter cloacae subsp. cloacae ATCC 13047 | CP001918.1.gene3440.p01 | ECL_03402 | 4.0E-10 | 90.91 | 30 | 11 |
| 3000793 | Escherichia coli O157:H7 str. Sakai | NC_002695.1.916585.p01 | ECs2883 | 9.0E-06 | 90.62 | 25 | 48 |
| 3000793 | Salmonella enterica subsp. enterica serovar Agona str. SL483 | CP001138.1.gene2235.p01 | SeAg_B2257 | 4.0E-07 | 90 | 26 | 28 |
| 3000794 | Enterobacter cloacae subsp. cloacae ATCC 13047 | CP001918.1.gene3441.p01 | ECL_03403 | 4.0E-08 | 90.62 | 31 | 17 |
| 3000794 | Escherichia coli O157:H7 str. Sakai | NC_002695.1.916586.p01 | ECs2884 | 1.0E-06 | 90.91 | 25 | 26 |
| 3000794 | Proteus mirabilis BB2000 | CP004022.1.gene1674.p01 | mdtC | 3.0E-07 | 90 | 30 | 14 |
| 3000794 | Salmonella enterica subsp. enterica serovar Agona str. SL483 | CP001138.1.gene2236.p01 | mdtC | 4.0E-07 | 90 | 26 | 18 |
| 3000795 | Escherichia coli O157:H7 str. Sakai | NC_002695.1.915750.p01 | ECs4393 | 1.0E-11 | 96.97 | 33 | 20 |
| 3000795 | Enterobacter cloacae subsp. cloacae ATCC 13047 | CP001918.1.gene3442.p01 | ECL_03404 | 9.0E-10 | 90.91 | 33 | 3 |
| 3000795 | Escherichia coli O157:H7 str. Sakai | NC_002695.1.916587.p01 | ECs2885 | 7.0E-07 | 96.15 | 25 | 14 |
| 3000800 | Pseudomonas aeruginosa PAO1 | NC_002516.2.881078.p01 | mexC | 3.0E-09 | 90.91 | 31 | 16 |
| 3000801 | Pseudomonas aeruginosa PAO1 | NC_002516.2.881071.p01 | mexD | 2.0E-06 | 90.91 | 28 | 5 |
| 3000801 | Pseudomonas aeruginosa | U57969.gene.p01 | mexD | 9.0E-09 | 90 | 30 | 6 |
| 3000804 | Pseudomonas aeruginosa PAO1 | NC_002516.2.882884.p01 | mexF | 9.0E-08 | 90.62 | 27 | 8 |
| 3000808 | Pseudomonas aeruginosa PAO1 | NC_002516.2.880346.p01 | mexI | 4.0E-06 | 90.91 | 26 | 4 |
| 3000814 | Acinetobacter baumannii AB307-0294 | NC_011595.7059912.p01 | ABBFA_002603 | 8.0E-08 | 90.91 | 26 | 62 |
| 3000822 | Enterococcus faecium DO | CP003583.1.gene1005.p01 | pmrA | 5.0E-10 | 100 | 31 | 1 |
| 3000826 | Escherichia coli O157:H7 str. Sakai | NC_002695.1.912965.p01 | ECs2654 | 4.0E-13 | 96.97 | 33 | 11 |
| 3000826 | Klebsiella pneumoniae subsp. pneumoniae MGH 78578 | CP000647.1.gene2414.p01 | sdiA | 3.0E-15 | 96.97 | 33 | 4 |
| 3000826 | Salmonella enterica subsp. enterica serovar Agona str. SL483 | CP001138.1.gene1162.p01 | SeAg_B1170 | 1.0E-12 | 93.55 | 31 | 1 |
| 3000828 | Acinetobacter baumannii AB307-0294 | NC_011595.7057856.p01 | ABBFA_000579 | 1.0E-09 | 90.91 | 26 | 21 |
| 3000828 | Acinetobacter baumannii SDF | NC_010400.5986590.p01 | baeR | 3.0E-10 | 90 | 25 | 14 |
| 3000828 | Enterobacter cloacae subsp. cloacae ATCC 13047 | CP001918.1.gene3444.p01 | ECL_03406 | 9.0E-12 | 100 | 29 | 1 |
| 3000828 | Escherichia coli O157:H7 str. Sakai | NC_002695.1.916589.p01 | ECs2887 | 2.0E-13 | 100 | 33 | 3 |
| 3000828 | Salmonella enterica subsp. enterica serovar Agona str. SL483 | CP001138.1.gene2239.p01 | baeR | 3.0E-08 | 96.15 | 26 | 3 |
| 3000829 | Klebsiella pneumoniae subsp. pneumoniae MGH 78578 | CP000647.1.gene2530.p01 | baeS | 5.0E-08 | 90.91 | 25 | 15 |
| 3000829 | Klebsiella pneumoniae subsp. pneumoniae MGH 78578 | CP000647.1.gene2531.p01 | baeR | 1.0E-13 | 96.97 | 33 | 3 |
| 3000829 | Acinetobacter baumannii AB307-0294 | NC_011595.7057524.p01 | ABBFA_000578 | 1.0E-08 | 90.32 | 26 | 18 |
| 3000829 | Acinetobacter baumannii SDF | NC_010400.5984250.p01 | baeS | 2.0E-07 | 90.62 | 25 | 4 |
| 3000829 | Enterobacter cloacae subsp. cloacae ATCC 13047 | CP001918.1.gene3443.p01 | ECL_03405 | 2.0E-12 | 93.94 | 33 | 1 |
| 3000829 | Escherichia coli O157:H7 str. Sakai | NC_002695.1.916588.p01 | ECs2886 | 8.0E-12 | 90.62 | 32 | 2 |
| 3000829 | Salmonella enterica subsp. enterica serovar Agona str. SL483 | CP001138.1.gene2238.p01 | SeAg_B2260 | 1.0E-12 | 96.97 | 33 | 1 |
| 3000830 | Escherichia coli O157:H7 str. Sakai | NC_002695.1.914983.p01 | cpxA | 1.0E-07 | 93.94 | 25 | 14 |
| 3000830 | Klebsiella pneumoniae subsp. pneumoniae MGH 78578 | CP000647.1.gene4256.p01 | cpxA | 1.0E-11 | 96.97 | 31 | 9 |
| 3000831 | Enterobacter cloacae subsp. cloacae ATCC 13047 | CP001918.1.gene5135.p01 | ECL_05064 | 1.0E-14 | 100 | 33 | 3 |
| 3000831 | Escherichia coli O157:H7 str. Sakai | NC_002695.1.915041.p01 | ECs4838 | 2.0E-10 | 100 | 27 | 6 |
| 3000831 | Klebsiella pneumoniae subsp. pneumoniae MGH 78578 | CP000647.1.gene4257.p01 | cpxR | 1.0E-11 | 100 | 30 | 2 |
| 3000831 | Proteus mirabilis BB2000 | CP004022.1.gene3215.p01 | cpxR | 2.0E-14 | 100 | 33 | 1 |
| 3000831 | Salmonella enterica subsp. enterica serovar Agona str. SL483 | CP001138.1.gene4273.p01 | cpxR | 1.0E-12 | 100 | 29 | 3 |
| 3000832 | Klebsiella pneumoniae subsp. pneumoniae MGH 78578 | CP000647.1.gene3517.p01 | evgA | 5.0E-11 | 90.91 | 28 | 5 |
| 3000833 | Shigella dysenteriae Sd197 | CP000034.1.gene4478.p01 | evgS | 2.0E-06 | 92.31 | 26 | 9 |
| 3000833 | Escherichia coli O157:H7 str. Sakai | NC_002695.1.915650.p01 | ECs3249 | 6.0E-06 | 93.94 | 27 | 21 |
| 3000833 | Escherichia coli O157:H7 str. Sakai | NC_002695.1.915651.p01 | ECs3248 | 3.0E-08 | 96.67 | 25 | 3 |
| 3000833 | Klebsiella pneumoniae subsp. pneumoniae MGH 78578 | CP000647.1.gene3518.p01 | evgS | 2.0E-08 | 90.32 | 26 | 18 |
| 3000834 | Enterobacter cloacae subsp. cloacae ATCC 13047 | CP001918.1.gene2526.p01 | ECL_02504 | 5.0E-15 | 100 | 33 | 1 |
| 3000834 | Klebsiella pneumoniae subsp. pneumoniae MGH 78578 | CP000647.1.gene2625.p01 | yejM | 4.0E-09 | 90.91 | 25 | 20 |
| 3000834 | Listeria monocytogenes | HE999704.1.gene2815.p01 | BN418_2960 | 8.0E-11 | 90 | 30 | 4 |
| 3000834 | Shigella dysenteriae Sd197 | CP000034.1.gene2022.p01 | phoP | 3.0E-11 | 100 | 28 | 3 |
| 3000834 | Streptococcus pneumoniae Taiwan19F-14 | NC_012469.1.7685629.p01 | SPT_1001 | 3.0E-07 | 92 | 25 | 1 |
| 3000835 | Klebsiella pneumoniae subsp. pneumoniae MGH 78578 | CP000647.1.gene1136.p01 | phoP | 1.0E-06 | 96.97 | 33 | 3 |
| 3000835 | Escherichia coli O157:H7 str. Sakai | NC_002695.1.913289.p01 | ECs1602 | 9.0E-07 | 100 | 33 | 2 |
| 3000835 | Enterobacter cloacae subsp. cloacae ATCC 13047 | CP001918.1.gene2527.p01 | ECL_02505 | 2.0E-08 | 96.3 | 27 | 1 |
| 3000835 | Escherichia coli O157:H7 str. Sakai | NC_002695.1.913290.p01 | ECs1601 | 3.0E-10 | 96.43 | 28 | 10 |
| 3000835 | Klebsiella pneumoniae subsp. pneumoniae MGH 78578 | CP000647.1.gene1135.p01 | phoQ | 2.0E-09 | 93.1 | 29 | 6 |
| 3000835 | Shigella dysenteriae Sd197 | CP000034.1.gene2023.p01 | phoQ | 2.0E-13 | 100 | 33 | 2 |
| 3000836 | Klebsiella pneumoniae subsp. pneumoniae MGH 78578 | CP000647.1.gene4500.p01 | soxR | 4.0E-11 | 100 | 28 | 3 |
| 3000836 | Escherichia coli O157:H7 str. Sakai | NC_002695.1.914292.p01 | ECs5045 | 5.0E-10 | 93.94 | 26 | 7 |
| 3000836 | Salmonella enterica subsp. enterica serovar Agona str. SL483 | CP001138.1.gene4489.p01 | soxR | 4.0E-12 | 90.91 | 33 | 4 |
| 3000837 | Enterobacter cloacae subsp. cloacae ATCC 13047 | CP001918.1.gene327.p01 | ECL_00320 | 9.0E-17 | 100 | 33 | 1 |
| 3000837 | Escherichia coli O157:H7 str. Sakai | NC_002695.1.914293.p01 | ECs5044 | 1.0E-12 | 90.91 | 33 | 2 |
| 3000837 | Escherichia coli O157:H7 str. Sakai | NC_002695.1.917670.p01 | ECs0930 | 1.0E-09 | 96.3 | 27 | 2 |
| 3000837 | Klebsiella pneumoniae subsp. pneumoniae MGH 78578 | CP000647.1.gene4499.p01 | soxS | 1.0E-09 | 96.15 | 25 | 2 |
| 3000838 | Listeria monocytogenes | HE999704.1.gene1528.p01 | BN418_1620 | 3.0E-08 | 90 | 25 | 5 |
| 3000873 | Escherichia coli | JF949916.1.gene1.p01 | blaTEM-1 | 4.0E-15 | 100 | 33 | 2 |
| 3000873 | Klebsiella pneumoniae | JF949915.1.gene1.p01 | blaTEM-1 | 8.0E-14 | 100 | 33 | 1 |
| 3000893 | Klebsiella pneumoniae | Y17583.1.gene1.p01 | blaTEM-22 | 1.0E-09 | 100 | 26 | 1 |
| 3000898 | Escherichia coli | U37195.1.gene1.p1 | blaTEM-28 | 1.0E-13 | 96.97 | 33 | 1 |
| 3000903 | Escherichia coli | GU371926.1.gene95.p01 | blaTEM-33 | 2.0E-12 | 93.94 | 33 | 6 |
| 3000916 | Klebsiella pneumoniae | Y10279.1.gene1.p01 | blaTEM-47 | 9.0E-15 | 100 | 33 | 1 |
| 3000917 | Klebsiella pneumoniae | Y10280.1.gene1.p01 | blaTEM-48 | 8.0E-16 | 100 | 33 | 2 |
| 3000941 | Klebsiella pneumoniae | AY130284.1.gene1.p1 | blaTEM-75 | 5.0E-14 | 100 | 33 | 1 |
| 3000980 | Escherichia coli | AY130282.1.gene1.p1 | blaTEM-117 | 2.0E-09 | 100 | 25 | 4 |
| 3001025 | Proteus mirabilis | EF136376.1.gene1.p01 | blaTEM-159 | 5.0E-15 | 100 | 33 | 1 |
| 3001065 | Klebsiella pneumoniae | Y11069.1.gene1.p01 | blaSHV-6 | 1.0E-15 | 100 | 33 | 1 |
| 3001095 | Klebsiella pneumoniae | AF317502.gene.p01 | blaSHV-37 | 3.0E-12 | 100 | 30 | 1 |
| 3001205 | Klebsiella pneumoniae subsp. pneumoniae MGH 78578 | CP000647.1.gene1649.p01 | KPN_01649 | 2.0E-14 | 90.91 | 33 | 2 |
| 3001205 | Klebsiella pneumoniae subsp. pneumoniae MGH 78578 | CP000647.1.gene1697.p01 | KPN_01697 | 1.0E-12 | 93.75 | 32 | 1 |
| 3001205 | Klebsiella pneumoniae subsp. pneumoniae MGH 78578 | CP000647.1.gene3367.p01 | KPN_03367 | 2.0E-12 | 100 | 29 | 1 |
| 3001211 | Escherichia coli | DQ464881.1.gene4.p01 | strA | 2.0E-09 | 100 | 25 | 10 |
| 3001211 | Pseudomonas aeruginosa | AF024602.1.gene5.p01 | strA | 1.0E-13 | 100 | 33 | 7 |
| 3001212 | Acinetobacter baumannii AYE | NC_010410.6003392.p01 | strB | 8.0E-14 | 100 | 33 | 2 |
| 3001212 | Escherichia coli | AJ313522.gene.p01 | strB | 2.0E-13 | 100 | 33 | 4 |
| 3001212 | Escherichia coli | DQ464881.1.gene5.p01 | strB | 2.0E-09 | 100 | 27 | 17 |
| 3001212 | Pseudomonas aeruginosa | AF024602.1.gene6.p01 | strB | 6.0E-14 | 100 | 33 | 2 |
| 3001213 | Acinetobacter baumannii AYE | NC_010410.6003949.p01 | ABAYE3640 | 2.0E-07 | 90.91 | 27 | 6 |
| 3001214 | Salmonella enterica subsp. enterica serovar Agona str. SL483 | CP001138.1.gene4809.p01 | SeAg_B4848 | 6.0E-10 | 90.91 | 33 | 4 |
| 3001214 | Salmonella enterica subsp. enterica serovar Agona str. SL483 | CP001138.1.gene894.p01 | SeAg_B0902 | 2.0E-08 | 90.91 | 28 | 11 |
| 3001215 | Escherichia coli O157:H7 str. Sakai | NC_002695.1.915390.p01 | ECs4647 | 1.0E-07 | 93.33 | 26 | 13 |
| 3001215 | Salmonella enterica subsp. enterica serovar Agona str. SL483 | CP001138.1.gene4042.p01 | SeAg_B4071 | 1.0E-10 | 90.91 | 33 | 6 |
| 3001216 | Enterobacter cloacae subsp. cloacae ATCC 13047 | CP001918.1.gene2596.p01 | ECL_02574 | 2.0E-11 | 93.94 | 30 | 3 |
| 3001216 | Salmonella enterica subsp. enterica serovar Agona str. SL483 | CP001138.1.gene2007.p01 | SeAg_B2022 | 2.0E-11 | 93.94 | 33 | 1 |

**Matched contigs of sample C1757 against CARD**

(Sorted by ARO-number)

| **ARO** | **Organism** | **Accession number** | **Gene** | **E value ≤** | **Identity (%) ≥** | **Hit length (aa) ≥** | **Number of contigs** |
| --- | --- | --- | --- | --- | --- | --- | --- |
| 3000017 | uncultured bacterium | AY139598.1.gene3.p01 | blaOXA-129 | 4.0E-129 | 97.8 | 182 | 1 |
| 3000017 | Pseudomonas aeruginosa | U37105.2.gene2.p01 | blaOXA-10 | 1.0E-103 | 92.59 | 162 | 1 |
| 3000017 | Salmonella enterica subsp. enterica serovar Bredeney | AM932669.1.gene2.p01 | blaOXA-129 | 1.0E-149 | 100 | 205 | 1 |
| 3000066 | Acinetobacter baumannii | GU207844.1.gene1.p01 | blaGES-14 | 3.0E-93 | 100 | 134 | 1 |
| 3000122 | Acinetobacter baumannii AYE | NC_010410.6000796.p01 | cat | 9.0E-42 | 92.65 | 68 | 1 |
| 3000122 | Klebsiella pneumoniae subsp. pneumoniae MGH 78578 | CP000647.1.gene2018.p01 | KPN_02018 | 6.0E-33 | 96.55 | 58 | 1 |
| 3000166 | Neisseria meningitidis | AB084246.gene.p01 | tetB | 8.0E-51 | 100 | 83 | 1 |
| 3000186 | Enterococcus faecalis | M85225.gene.p01 | tetM | 3.0E-12 | 100 | 32 | 1 |
| 3000186 | Enterococcus faecalis | X04388.gene.p01 | tetM | 7.0E-77 | 100 | 116 | 1 |
| 3000191 | Bacteroides fragilis | Z21523.gene.p01 | tetQ | < 1.0E-150 | 95.71 | 163 | 2 |
| 3000194 | Butyrivibrio fibrisolvens | AJ222769.gene.p01 | tetW* | < 1.0E-150 | 100 | 245 | 1 |
| 3000196 | Clostridiaceae bacterium K10 | AJ295238.gene.p01 | tet32 | 3.0E-76 | 98.32 | 119 | 1 |
| 3000210 | Acinetobacter baumannii ATCC 17978 | NC_009085.4918494.p01 | rpoB | 1.0E-52 | 94.29 | 105 | 3 |
| 3000210 | Acinetobacter baumannii SDF | NC_010400.5987325.p01 | rpoB | 3.0E-37 | 92.77 | 70 | 2 |
| 3000210 | Bordetella pertussis CS | CP002695.1.gene18.p01 | rpoB | 4.0E-55 | 92 | 100 | 1 |
| 3000210 | Clostridium botulinum A2 str. Kyoto | CP001581.1.gene3846.p01 | rpoB | 2.0E-38 | 92.31 | 91 | 1 |
| 3000210 | Clostridium difficile 630 | AM180355.1.gene120.p01 | rpoB | 6.0E-50 | 96.43 | 56 | 1 |
| 3000210 | Escherichia coli O157:H7 str. Sakai | NC_002695.1.914942.p01 | rpoB | 4.0E-64 | 100 | 105 | 1 |
| 3000210 | Klebsiella pneumoniae subsp. pneumoniae MGH 78578 | CP000647.1.gene4402.p01 | rpoB | 2.0E-68 | 100 | 115 | 1 |
| 3000210 | Neisseria gonorrhoeae NCCP11945 | NC_011035.1.6448762.p01 | rpoB | 3.0E-75 | 96 | 69 | 2 |
| 3000210 | Streptococcus pneumoniae Taiwan19F-14 | NC_012469.1.7686402.p01 | rpoB | 4.0E-09 | 92.59 | 27 | 4 |
| 3000226 | Escherichia coli O157:H7 str. Sakai | NC_002695.1.916103.p01 | folP | 5.0E-28 | 100 | 53 | 1 |
| 3000226 | Klebsiella pneumoniae subsp. pneumoniae MGH 78578 | CP000647.1.gene3624.p01 | folP | 2.0E-39 | 100 | 66 | 1 |
| 3000232 | Escherichia coli 1520 | NC_010558.1.6275994.p01 | aadA4 | < 1.0E-150 | 100 | 262 | 1 |
| 3000275 | Enterococcus faecium | AF110130.1.orf0.gene.p01 | linB | 3.0E-62 | 94.64 | 104 | 2 |
| 3000309 | Klebsiella pneumoniae subsp. pneumoniae MGH 78578 | CP000647.1.gene4115.p01 | emrD | 1.0E-15 | 98.29 | 73 | 2 |
| 3000373 | Escherichia coli O157:H7 str. Sakai | NC_002695.1.915653.p01 | ECs3247 | 3.0E-67 | 99.05 | 105 | 1 |
| 3000375 | Streptococcus pneumoniae | AM410044.gene14.p01 | ermB | 2.0E-160 | 99.18 | 245 | 1 |
| 3000410 | Pseudomonas aeruginosa | AF191564.1.gene5.p01 | sul1 | 1.0E-178 | 100 | 254 | 1 |
| 3000412 | Escherichia coli | DQ464881.1.gene2.p01 | sul2 | 2.0E-97 | 99.32 | 148 | 1 |
| 3000457 | Acinetobacter baumannii SDF | NC_010400.5986295.p01 | parE | 8.0E-31 | 92.06 | 63 | 2 |
| 3000457 | Salmonella enterica subsp. enterica serovar Agona str. SL483 | CP001138.1.gene3336.p01 | parE | 2.0E-54 | 97.87 | 94 | 1 |
| 3000461 | Clostridium botulinum A2 str. Kyoto | CP001581.1.gene3143.p01 | CLM_3236 | 2.0E-38 | 93.71 | 65 | 2 |
| 3000516 | Klebsiella pneumoniae subsp. pneumoniae MGH 78578 | CP000647.1.gene3013.p01 | emrR | 2.0E-70 | 99.03 | 103 | 1 |
| 3000518 | Klebsiella pneumoniae subsp. pneumoniae MGH 78578 | CP000647.1.gene3780.p01 | crp | 8.0E-71 | 100 | 84 | 1 |
| 3000518 | Shigella dysenteriae Sd197 | CP000034.1.gene3519.p01 | crp | 8.0E-109 | 100 | 151 | 1 |
| 3000533 | Escherichia coli O157:H7 str. Sakai | NC_002695.1.917702.p01 | ECs0964 | < 1.0E-150 | 99.11 | 224 | 1 |
| 3000535 | Acinetobacter baumannii AB307-0294 | NC_011595.7060505.p01 | ABBFA_003019 | < 1.0E-150 | 92.8 | 361 | 1 |
| 3000566 | Acinetobacter sp. LUH5605 | AY743590.gene.p01 | tet39 | 1.0E-17 | 97.67 | 43 | 1 |
| 3000616 | Streptococcus pneumoniae Taiwan19F-14 | NC_012469.1.7685970.p01 | SPT_1925 | 2.0E-74 | 90.4 | 122 | 2 |
| 3000618 | Acinetobacter baumannii AB307-0294 | NC_011595.7058445.p01 | gyrA | 1.0E-37 | 93.03 | 68 | 2 |
| 3000618 | Azoarcus sp. BH72 | NC_008702.1.4606680.p01 | gyrA | 1.0E-10 | 90.64 | 32 | 3 |
| 3000618 | Escherichia coli O157:H7 str. Sakai | NC_002695.1.916822.p01 | gyrA | 6.0E-61 | 99 | 100 | 1 |
| 3000618 | Klebsiella pneumoniae subsp. pneumoniae MGH 78578 | CP000647.1.gene2640.p01 | gyrA | 3.0E-36 | 100 | 68 | 2 |
| 3000618 | Streptococcus pneumoniae Taiwan19F-14 | NC_012469.1.7686721.p01 | gyrA | 4.0E-43 | 98.59 | 71 | 1 |
| 3000619 | Escherichia coli str. K-12 substr. W3110 | M58408.gene.p01 | parC | 2.0E-70 | 100 | 56 | 1 |
| 3000702 | Klebsiella pneumoniae subsp. pneumoniae MGH 78578 | CP000647.1.gene445.p01 | acrR | 8.0E-39 | 100 | 66 | 1 |
| 3000702 | Shigella dysenteriae Sd197 | CP000034.1.gene455.p01 | acrR | 7.0E-65 | 100 | 72 | 1 |
| 3000718 | Acinetobacter baumannii ATCC 19606 | NC_006877.3293011.p01 | marR | 2.0E-11 | 93.75 | 32 | 2 |
| 3000794 | Escherichia coli O157:H7 str. Sakai | NC_002695.1.916586.p01 | ECs2884 | 4.0E-41 | 96.08 | 102 | 1 |
| 3000795 | Escherichia coli O157:H7 str. Sakai | NC_002695.1.915750.p01 | ECs4393 | < 1.0E-150 | 99.53 | 215 | 1 |
| 3000814 | Acinetobacter baumannii AB307-0294 | NC_011595.7059912.p01 | ABBFA_002603 | 4.0E-82 | 90.15 | 132 | 2 |
| 3000828 | Acinetobacter baumannii AB307-0294 | NC_011595.7057856.p01 | ABBFA_000579 | 2.0E-147 | 91.74 | 218 | 1 |
| 3000829 | Klebsiella pneumoniae subsp. pneumoniae MGH 78578 | CP000647.1.gene2530.p01 | baeS | 1.0E-75 | 100 | 85 | 1 |
| 3000831 | Escherichia coli O157:H7 str. Sakai | NC_002695.1.915041.p01 | ECs4838 | 4.0E-39 | 100 | 64 | 1 |
| 3000834 | Klebsiella pneumoniae subsp. pneumoniae MGH 78578 | CP000647.1.gene2625.p01 | yejM | 3.0E-80 | 99.17 | 120 | 1 |
| 3000837 | Escherichia coli O157:H7 str. Sakai | NC_002695.1.917670.p01 | ECs0930 | 4.0E-20 | 97.5 | 40 | 1 |
| 3000936 | Escherichia coli | AF188199.1.gene1.p01 | blaTEM-70 | 1.0E-122 | 100 | 157 | 1 |
| 3001211 | Pseudomonas aeruginosa | AF024602.1.gene5.p01 | strA | 4.0E-42 | 98.57 | 70 | 2 |
| 3001212 | Escherichia coli | DQ464881.1.gene5.p01 | strB | 1.0E-107 | 100 | 153 | 1 |
