# Supplementary material for: Influence of a Non-Hospital Medical Care Facility on Antimicrobial Resistance in Wastewater
Source: PLoS One. 2015 Mar 30;10(3):e0122635. doi: 10.1371/journal.pone.0122635 (PMC4379178; doi:10.1371/journal.pone.0122635)
Supplement: S3 Table — Matched high-throughput sequencing reads/contigs of the samples C1754, C1755, C1756 and C1757 against the INTEGRALL database. (DOCX) [file pone.0122635.s003.docx]

**Table S3: Matched high-throughput sequencing reads/contigs of the samples C1754, C1755, C1756 and C1757 against the INTEGRALL database**

**Matched high-throughput sequencing reads of sample 1754 against the INTEGRALL database**

(Ranked alphabetically by Organism

| **Accession number** | **Organism** | **Integrase gene** | **E value**  **≤** | **Identity (%) ≥** | **Hit length (bp) ≥** | **Number of reads** |
| --- | --- | --- | --- | --- | --- | --- |
| CP001921 | Acinetobacter baumannii 1656-2 | *intI1* | 1.0E-25 | 98.55 | 69 | 1 |
| BX248359 | Corynebacterium diphtheriae | *intI1* | 3.0E-20 | 100 | 56 | 1 |
| AB188271 | Escherichia coli | *intI2* | 5.0E-45 | 100 | 101 | 2 |
| AJ639924 | IncP-1beta Plasmid pB3 | *intI1* | 1.0E-43 | 99.01 | 101 | 2 |
| FJ502341 | Neisseria sp. GSR70 | *intI1* | 4.0E-45 | 100 | 101 | 1 |
| AM055748 | Salmonella enterica | *intI1* | 2.0E-45 | 100 | 101 | 1 |
| AY339985 | Salmonella typhimurium DT104 | *groEL/intI1* | 7.0E-16 | 90.32 | 62 | 1 |
| BX664015 | Serratia marcescens | *intI1* | 5.0E-45 | 100 | 101 | 1 |
| AF406792 | Stenotrophomonas maltophilia | *intI1* | 7.0E-09 | 90.38 | 50 | 20 |
| EU531477 | uncultured bacterium | *intI1* | 7.0E-45 | 100 | 101 | 1 |
| FM866474 | uncultured bacterium | *intI* | 4.0E-41 | 100 | 94 | 1 |
| FJ377589 | uncultured microorganism | *intI* | 1.0E-32 | 91.92 | 99 | 1 |

**Matched high-throughput sequencing reads of sample 1755 against the INTEGRALL database**

(Ranked alphabetically by Organism)

| **Accession number** | **Organism** | **Integrase gene** | **E value**  **≤** | **Identity (%) ≥** | **Hit length (bp) ≥** | **Number of reads** |
| --- | --- | --- | --- | --- | --- | --- |
| CP001921 | Acinetobacter baumannii 1656-2 | *intI1* | 4.0E-27 | 98.99 | 69 | 6 |
| DQ372711 | Azoarcus communis | *intI1* | 2.0E-44 | 100 | 101 | 1 |
| AB027715 | Corynebacterium glutamicum | *intI1* | 6.0E-18 | 97.03 | 52 | 8 |
| CP000089 | Dechloromonas aromatica RCB | *intI* | 6.0E-18 | 90 | 70 | 4 |
| NC_011992 | Diaphorobacter sp. TPSY | *intI* | 3.0E-34 | 93 | 100 | 2 |
| AB061794 | Escherichia coli | *intI1* | 7.0E-43 | 99.01 | 100 | 4 |
| AB188258 | Escherichia coli | *intI1* | 2.0E-31 | 100 | 75 | 1 |
| AY970968 | Escherichia coli | *intI1* | 5.0E-13 | 92.16 | 51 | 1 |
| AB104852 | Pseudomonas aeruginosa | *intI1* | 1.0E-44 | 100 | 101 | 1 |
| AB281182 | Pseudomonas aeruginosa | *intI1* | 2.0E-42 | 99 | 100 | 2 |
| AY257539 | Pseudomonas aeruginosa | *intI1* | 1.0E-19 | 100 | 55 | 2 |
| PSEAADA | Pseudomonas aeruginosa | *intI1* | 4.0E-34 | 92.08 | 101 | 2 |
| DQ836009 | Salmonella enterica subsp. enterica serovar Stanley | *intI1* | 4.0E-35 | 96.04 | 83 | 4 |
| AY524415 | Salmonella enterica subsp. enterica serovar Typhimurium | *intI1* | 2.0E-18 | 95.08 | 54 | 4 |
| AY339985 | Salmonella typhimurium DT104 | *groEL/intI1* | 6.0E-11 | 90.2 | 51 | 1 |
| AB070224 | Serratia marcescens | *intI3* | 6.0E-25 | 94.67 | 75 | 8 |
| AF406792 | Stenotrophomonas maltophilia | *intI1* | 9.0E-08 | 90 | 50 | 48 |
| EU327987 | Thauera sp. E7 | *intI1* | 4.0E-40 | 97.03 | 101 | 2 |
| AY283624 | uncultured bacterium | *intI* | 6.0E-21 | 91.67 | 72 | 1 |
| DQ282282 | uncultured bacterium | *intI* | 1.0E-16 | 91.94 | 59 | 2 |
| DQ287861 | uncultured bacterium | *intI* | 4.0E-10 | 90 | 50 | 1 |
| EU531477 | uncultured bacterium | *intI1* | 2.0E-26 | 100 | 67 | 1 |
| FJ820120 | uncultured bacterium | *intI1* | 2.0E-11 | 90.57 | 53 | 2 |
| FJ820142 | uncultured bacterium | *intI1* | 2.0E-44 | 100 | 101 | 1 |
| FJ820144 | uncultured bacterium | *intI1* | 9.0E-10 | 90 | 50 | 3 |
| JX486125 | uncultured bacterium | *intI1* | 5.0E-31 | 98.73 | 79 | 1 |
| AF324484 | Xanthomonas sp. CIP 102397 | *intIA* | 8.0E-10 | 90 | 50 | 1 |

**Matched contigs of sample 1755 against the INTEGRALL database**

(Ranked alphabetically by bacterial host)

| **Accession number** | **Bacterial host** | **Integrase gene** | **E value**  **≤** | **Identity (%) ≥** | **Hit length (bp) ≥** | **Number of contigs** |
| --- | --- | --- | --- | --- | --- | --- |
| CP001921 | Acinetobacter baumannii 1656-2 | *intI1* | 2.0E-79 | 100 | 163 | 1 |
| CP000089 | Dechloromonas aromatica RCB | *intI* | 1.0E-20 | 91.43 | 70 | 1 |
| AF231133 | Pseudomonas aeruginosa | *intI1* | 2.0E-89 | 99.46 | 184 | 1 |
| PSEAAC3IB | Pseudomonas aeruginosa | *intI1* | < 1.0E-150 | 99.27 | 410 | 1 |
| AF406792 | Stenotrophomonas maltophilia | *intI1* | 2.0E-15 | 90.62 | 58 | 3 |
| FM866474 | uncultured bacterium | *intI* | 4.0E-91 | 100 | 184 | 1 |

**Matched high-throughput sequencing reads of sample 1756 against the INTEGRALL database**

(Ranked alphabetically by Organism)

| **Accession number** | **Organism** | **Integrase gene** | **E value**  **≤** | **Identity (%) ≥** | **Hit length (bp) ≥** | **Number of reads** |
| --- | --- | --- | --- | --- | --- | --- |
| CP001921 | Acinetobacter baumannii 1656-2 | *intI1* | 1.0E-16 | 92.31 | 51 | 62 |
| EU523064 | Acinetobacter seohaensis | *intI1* | 1.0E-44 | 100 | 101 | 1 |
| AF439785 | Campylobacter jejuni | *intI1* | 2.0E-44 | 100 | 101 | 3 |
| AF486817 | Citrobacter amalonaticus | *intI1* | 9.0E-14 | 98 | 50 | 1 |
| NC_011992 | Diaphorobacter sp. TPSY | *intI* | 7.0E-33 | 92.08 | 100 | 3 |
| AB188271 | Escherichia coli | *intI2* | 9.0E-39 | 96.04 | 97 | 5 |
| AM932677 | Escherichia coli | *intI2* | 3.0E-19 | 99.01 | 54 | 2 |
| FJ594767 | Escherichia coli | *intI1* | 1.0E-44 | 100 | 101 | 1 |
| X51546 | Escherichia coli | *intI2* | 7.0E-43 | 99.01 | 101 | 1 |
| AJ639924 | IncP-1beta Plasmid pB3 | *intI1* | 4.0E-40 | 97.03 | 97 | 9 |
| EF660563 | Klebsiella pneumoniae | *intI1* | 2.0E-36 | 100 | 86 | 1 |
| AJ938161 | Morganella morganii | *intI2* | 4.0E-44 | 100 | 101 | 2 |
| FJ502340 | Neisseria sp. GRW28 | *intI1* | 8.0E-25 | 100 | 65 | 1 |
| FJ502341 | Neisseria sp. GSR70 | *intI1* | 2.0E-44 | 100 | 101 | 4 |
| CP000450 | Nitrosomonas eutropha C91 | *intI* | 1.0E-30 | 95.4 | 87 | 1 |
| DQ533990 | Providencia stuartii | *intI2* | 1.0E-41 | 99 | 100 | 1 |
| AF231133 | Pseudomonas aeruginosa | *intI1* | 1.0E-25 | 100 | 67 | 1 |
| AJ511268 | Pseudomonas aeruginosa | *intI1* | 8.0E-43 | 99.01 | 101 | 1 |
| HM021184 | Pseudomonas aeruginosa | *intI1* | 9.0E-43 | 99.01 | 101 | 1 |
| PSEAADA | Pseudomonas aeruginosa | *intI1* | 7.0E-21 | 99.01 | 59 | 8 |
| AM261760 | Pseudomonas aeruginosa IncP-1alpha plasmid pBS228 | *intI2* | 7.0E-26 | 100 | 68 | 1 |
| AY038186 | Pseudomonas alcaligenes | *intIPac* | 3.0E-12 | 94.12 | 51 | 2 |
| AJ420864 | Pseudomonas putida | *intI1* | 1.0E-44 | 100 | 101 | 2 |
| FJ711658 | Riemerella anatipestifer | *intI1* | 1.0E-44 | 100 | 101 | 1 |
| AM055748 | Salmonella enterica | *intI1* | 1.0E-44 | 100 | 101 | 1 |
| DQ836009 | Salmonella enterica subsp. enterica serovar Stanley | *intI1* | 5.0E-17 | 100 | 51 | 4 |
| AY136758 | Salmonella enterica subsp. enterica serovar Typhimurium | *intI1* | 1.0E-44 | 100 | 101 | 1 |
| AY524415 | Salmonella enterica subsp. enterica serovar Typhimurium | *intI1* | 3.0E-13 | 93.33 | 50 | 9 |
| AY339985 | Salmonella typhimurium DT104 | *groEL/intI1* | 5.0E-14 | 90 | 60 | 1 |
| BX664015 | Serratia marcescens | *intI1* | 4.0E-42 | 100 | 97 | 3 |
| AF406792 | Stenotrophomonas maltophilia | *intI1* | 5.0E-07 | 90 | 50 | 52 |
| AB355131 | uncultured bacterium | *intI1* | 4.0E-44 | 100 | 101 | 2 |
| DQ282194 | uncultured bacterium | *intI2* | 4.0E-44 | 100 | 101 | 1 |
| DQ282196 | uncultured bacterium | *intI3* | 4.0E-44 | 100 | 101 | 1 |
| DQ282200 | uncultured bacterium | *intI3* | 2.0E-10 | 90.38 | 52 | 1 |
| EU531497 | uncultured bacterium | *intI1* | 3.0E-44 | 100 | 101 | 1 |
| FM866473 | uncultured bacterium | *intI* | 3.0E-44 | 100 | 101 | 1 |
| FM866474 | uncultured bacterium | *intI* | 7.0E-41 | 100 | 95 | 1 |
| JX486125 | uncultured bacterium | *intI1* | 1.0E-16 | 98.59 | 51 | 20 |
| FJ377589 | uncultured microorganism | *intI* | 5.0E-34 | 93.07 | 101 | 1 |
| FJ377597 | uncultured microorganism | *intI* | 6.0E-36 | 94.06 | 101 | 1 |

**Matched contigs of sample 1756 against the INTEGRALL database**

(Ranked alphabetically by Organism)

| **Accession number** | **Organism** | **Integrase gene** | **E value**  **≤** | **Identity (%) ≥** | **Hit length (bp) ≥** | **Number of contigs** |
| --- | --- | --- | --- | --- | --- | --- |
| CP001921 | Acinetobacter baumannii 1656-2 | *intI1* | 7.0E-78 | 100 | 162 | 1 |
| DQ533990 | Providencia stuartii | *intI2* | 8.0E-128 | 99.61 | 257 | 1 |
| PSEAADA | Pseudomonas aeruginosa | *intI1* | < 1.0E-150 | 99.92 | 1289 | 1 |
| AF406792 | Stenotrophomonas maltophilia | *intI1* | 4.0E-41 | 90.23 | 106 | 1 |

**Matched high-throughput sequencing reads of sample 1757 against the INTEGRALL database**

(Ranked alphabetically by Organism)

| **Accession number** | **Organism** | **Integrase gene** | **E value**  **≤** | **Identity (%) ≥** | **Hit length (bp) ≥** | **Number of reads** |
| --- | --- | --- | --- | --- | --- | --- |
| CP001921 | Acinetobacter baumannii 1656-2 | *intI1* | 1.0E-17 | 91.75 | 52 | 24 |
| EU523064 | Acinetobacter seohaensis | *intI1* | 6.0E-19 | 96.55 | 53 | 4 |
| BX248359 | Corynebacterium diphtheriae | *intI1* | 2.0E-27 | 97.33 | 75 | 1 |
| CP000089 | Dechloromonas aromatica RCB | *intI* | 4.0E-12 | 90.22 | 55 | 5 |
| EF467661 | Delftia acidovorans | *intI3* | 4.0E-31 | 98.02 | 77 | 6 |
| NC_011992 | Diaphorobacter sp. TPSY | *intI* | 9.0E-33 | 92.08 | 98 | 5 |
| AB188271 | Escherichia coli | *intI2* | 3.0E-14 | 96 | 50 | 3 |
| AF318070 | Escherichia coli | *intI2* | 5.0E-22 | 100 | 59 | 1 |
| AJ001816 | Escherichia coli | *intI2* | 2.0E-44 | 100 | 101 | 2 |
| AY781413 | Escherichia coli | *intI1* | 3.0E-19 | 100 | 54 | 1 |
| EF560797 | Escherichia coli | *intI1* | 6.0E-45 | 100 | 101 | 2 |
| AB733642 | Escherichia coli | *intI* | 2.0E-10 | 90.38 | 52 | 1 |
| AJ639924 | IncP-1beta Plasmid pB3 | *intI1* | 4.0E-42 | 98.02 | 101 | 5 |
| AY219651 | Klebsiella pneumoniae Plasmid p22K9 | *intI3* | 2.0E-29 | 100 | 74 | 2 |
| NC_013951 | Klebsiella pneumoniae plasmid pKF3-140 | *intI1* | 3.0E-44 | 100 | 101 | 1 |
| AB281182 | Pseudomonas aeruginosa | *intI1* | 2.0E-44 | 100 | 101 | 2 |
| AF043381 | Pseudomonas aeruginosa | *intI1* | 2.0E-43 | 99.01 | 101 | 1 |
| AJ511268 | Pseudomonas aeruginosa | *intI1* | 3.0E-24 | 100 | 63 | 1 |
| AY257539 | Pseudomonas aeruginosa | *intI1* | 5.0E-17 | 98.15 | 54 | 3 |
| AY775051 | Pseudomonas aeruginosa | *intI1* | 2.0E-35 | 98.85 | 87 | 3 |
| HM021184 | Pseudomonas aeruginosa | *intI1* | 8.0E-45 | 100 | 101 | 4 |
| PSEAADA | Pseudomonas aeruginosa | *intI1* | 3.0E-20 | 93.88 | 57 | 13 |
| AJ420864 | Pseudomonas putida | *intI1* | 9.0E-41 | 97.03 | 101 | 2 |
| FJ711658 | Riemerella anatipestifer | *intI1* | 2.0E-42 | 98.02 | 101 | 1 |
| EU664609 | Salmonella enterica subsp. enterica serovar Kentucky | *intI1delta* | 6.0E-45 | 100 | 101 | 5 |
| DQ836009 | Salmonella enterica subsp. enterica serovar Stanley | *intI1* | 8.0E-28 | 93.07 | 70 | 7 |
| AM261837 | Salmonella enterica subsp. enterica serovar Typhimurium | *intI1* | 1.0E-16 | 98.04 | 51 | 1 |
| AY524415 | Salmonella enterica subsp. enterica serovar Typhimurium | *intI1* | 3.0E-20 | 100 | 57 | 8 |
| EU924797 | Salmonella enterica subsp. enterica serovar Virchow | *intI1* | 1.0E-44 | 100 | 101 | 1 |
| AF071555 | Salmonella typhimurium DT104 | *groEL/intI1* | 2.0E-40 | 97.03 | 101 | 1 |
| AY339985 | Salmonella typhimurium DT104 | *groEL/intI1* | 5.0E-16 | 91.8 | 61 | 2 |
| AB070224 | Serratia marcescens | *intI3* | 1.0E-18 | 91.49 | 54 | 15 |
| BX664015 | Serratia marcescens | *intI1* | 1.0E-44 | 100 | 101 | 2 |
| AF406792 | Stenotrophomonas maltophilia | *intI1* | 2.0E-06 | 90 | 50 | 74 |
| EU327987 | Thauera sp. E7 | *intI1* | 2.0E-41 | 98.02 | 101 | 2 |
| AB355131 | uncultured bacterium | *intI1* | 1.0E-26 | 100 | 68 | 1 |
| DQ282194 | uncultured bacterium | *intI2* | 2.0E-44 | 100 | 101 | 1 |
| DQ282196 | uncultured bacterium | *intI3* | 4.0E-27 | 100 | 69 | 9 |
| DQ282200 | uncultured bacterium | *intI3* | 2.0E-44 | 100 | 101 | 3 |
| DQ282213 | uncultured bacterium | *intI* | 4.0E-14 | 90 | 60 | 1 |
| DQ282282 | uncultured bacterium | *intI* | 2.0E-17 | 94.92 | 59 | 1 |
| EU531477 | uncultured bacterium | *intI1* | 1.0E-27 | 100 | 70 | 3 |
| EU531490 | uncultured bacterium | *intI1* | 2.0E-44 | 100 | 101 | 1 |
| EU531497 | uncultured bacterium | *intI1* | 3.0E-20 | 100 | 56 | 1 |
| FJ820120 | uncultured bacterium | *intI1* | 4.0E-11 | 90.57 | 53 | 1 |
| FJ820144 | uncultured bacterium | *intI1* | 1.0E-11 | 90.74 | 54 | 1 |
| FJ820146 | uncultured bacterium | *intI1* | 9.0E-20 | 90.54 | 74 | 3 |
| FM866473 | uncultured bacterium | *intI* | 5.0E-17 | 100 | 50 | 1 |
| FM866499 | uncultured bacterium | *intI* | 6.0E-30 | 100 | 74 | 5 |
| JX486125 | uncultured bacterium | *intI1* | 6.0E-27 | 99 | 69 | 5 |
| FJ377589 | uncultured microorganism | *intI* | 2.0E-15 | 94.64 | 56 | 1 |
| FJ377605 | uncultured microorganism | *intI* | 2.0E-16 | 90.77 | 65 | 1 |
| FJ377611 | uncultured microorganism | *intI* | 4.0E-11 | 90.57 | 53 | 1 |
| AB114632 | Vibrio fluvialis | *intI1* | 3.0E-24 | 100 | 62 | 1 |

**Matched contigs of sample 1757 against the INTEGRALL database**

(Ranked alphabetically by Organism)

| **Accession number** | **Organism** | **Integrase gene** | **E value**  **≤** | **Identity (%) ≥** | **Hit length (bp) ≥** | **Number of contigs** |
| --- | --- | --- | --- | --- | --- | --- |
| CP001921 | Acinetobacter baumannii 1656-2 | *intI1* | 7.0E-79 | 100 | 163 | 1 |
| PSEAADA | Pseudomonas aeruginosa | *intI1* | < 1.0E-150 | 99.84 | 1288 | 1 |
| AY524415 | Salmonella enterica subsp. enterica serovar Typhimurium | *intI1* | 3.0E-64 | 100 | 136 | 1 |
| AB070224 | Serratia marcescens | *intI3* | < 1.0E-150 | 100 | 369 | 1 |
| AF406792 | Stenotrophomonas maltophilia | *intI1* | 7.0E-13 | 94.83 | 50 | 4 |
