# Supplementary material for: Influence of a Non-Hospital Medical Care Facility on Antimicrobial Resistance in Wastewater
Source: PLoS One. 2015 Mar 30;10(3):e0122635. doi: 10.1371/journal.pone.0122635 (PMC4379178; doi:10.1371/journal.pone.0122635)
Supplement: S4 Table — Assigned high-throughput sequencing reads/contigs of the samples C1754, C1755, C1756 and C1757 against the RefSeq plasmid. (DOCX) [file pone.0122635.s004.docx]

**Table S4: Assigned high-throughput sequencing reads/contigs of the samples C1754, C1755, C1756 and C1757 against the RefSeq plasmid database**

**Assigned high-throughput reads of sample C1754 against the RefSeq plasmid database**

(Sorted by Organism)

| **Accession number** | **Organism** | **Plasmid** | **E value ≤** | **Identity (%) ≥** | **Hit length (bp) ≥** | **Number of reads** |
| --- | --- | --- | --- | --- | --- | --- |
| NC_021976.1 | Acetobacter pasteurianus 386B | Apa386Bp1 | 2.0E-35 | 95.05 | 101 | 1 |
| NC_005793.2 | Achromobacter denitrificans | pEST4011 | 6.0E-36 | 95.05 | 100 | 34 |
| NC_014641.1 | Achromobacter xylosoxidans A8 | pA81 | 2.0E-38 | 97.03 | 101 | 2 |
| NC_006830.1 | Achromobacter xylosoxidans A8 | pA81 | 7.0E-43 | 100 | 101 | 1 |
| NC_022242.1 | Achromobacter xylosoxidans subsp. denitrificans pAX22 |  | 2.0E-36 | 95.05 | 99 | 7 |
| NC_015187.1 | Acidiphilium multivorum AIU301 | pACMV2 | 1.0E-38 | 97.03 | 101 | 1 |
| NC_015852.1 | Acidithiobacillus caldus SM-1 | pLAtc1 | 3.0E-42 | 99.01 | 101 | 1 |
| NC_010600.1 | Acidithiobacillus caldus strain MNG | pTcM1 | 5.0E-43 | 100 | 101 | 1 |
| NC_008765.1 | Acidovorax sp. JS42 | pAOVO01 | 1.0E-37 | 96.04 | 92 | 11 |
| NC_022565.1 | Acinetobacter baumannii 107m | p1ABIBUN | 2.0E-38 | 96.04 | 101 | 1 |
| NC_017163.1 | Acinetobacter baumannii 1656-2 | ABKp1 | 9.0E-34 | 96.04 | 91 | 22 |
| NC_017164.1 | Acinetobacter baumannii 1656-2 | ABKp2 | 5.0E-32 | 95.05 | 91 | 11 |
| NC_006877.1 | Acinetobacter baumannii 19606 | pMAC | 8.0E-37 | 95.05 | 94 | 15 |
| NC_010605.1 | Acinetobacter baumannii ACICU | pACICU1 | 4.0E-34 | 95 | 91 | 90 |
| NC_010606.1 | Acinetobacter baumannii ACICU | pACICU2 | 5.0E-43 | 100 | 101 | 3 |
| NC_009083.1 | Acinetobacter baumannii ATCC 17978 | pAB1 | 3.0E-37 | 95.96 | 99 | 3 |
| NC_009084.1 | Acinetobacter baumannii ATCC 17978 | pAB2 | 2.0E-38 | 96.04 | 101 | 8 |
| NC_010401.1 | Acinetobacter baumannii AYE | p1ABAYE | 3.0E-34 | 95.05 | 95 | 32 |
| NC_010402.1 | Acinetobacter baumannii AYE | p2ABAYE | 7.0E-44 | 100 | 101 | 5 |
| NC_010404.1 | Acinetobacter baumannii AYE | p3ABAYE | 2.0E-31 | 95 | 90 | 510 |
| NC_021727.1 | Acinetobacter baumannii BJAB07104 | p1BJAB07104 | 3.0E-39 | 100 | 94 | 32 |
| NC_021728.1 | Acinetobacter baumannii BJAB07104 | p2BJAB07104 | 2.0E-36 | 95.05 | 101 | 14 |
| NC_021734.1 | Acinetobacter baumannii BJAB0715 | pBJAB0715 | 3.0E-32 | 95 | 90 | 273 |
| NC_020525.1 | Acinetobacter baumannii D1279779 | pD1279779 | 4.0E-33 | 95 | 91 | 13 |
| NC_017848.1 | Acinetobacter baumannii MDR-TJ | pABTJ1 | 6.0E-36 | 95.05 | 95 | 33 |
| NC_020524.1 | Acinetobacter baumannii MDR-TJ | pABTJ2 | 3.0E-35 | 95 | 91 | 113 |
| NC_010481.1 | Acinetobacter baumannii | pABIR | 1.0E-34 | 95 | 90 | 82 |
| NC_021489.1 | Acinetobacter baumannii | pAB-NCGM253 DNA | 2.0E-32 | 95.05 | 90 | 19 |
| NC_012813.1 | Acinetobacter baumannii | pABVA01 | 8.0E-37 | 95.05 | 101 | 5 |
| NC_013277.1 | Acinetobacter baumannii | pMMA2 | 3.0E-36 | 95 | 100 | 19 |
| NC_013506.1 | Acinetobacter baumannii | pMMCU2 | 7.0E-32 | 95.05 | 91 | 31 |
| NC_019280.1 | Acinetobacter baumannii | pMMD | 9.0E-37 | 95.05 | 92 | 102 |
| NC_019345.1 | Acinetobacter baumannii | pRAY*-v2 | 3.0E-36 | 96.04 | 90 | 83 |
| NC_016977.1 | Acinetobacter baumannii | pTS236 | 2.0E-37 | 95.05 | 101 | 2 |
| NC_010395.1 | Acinetobacter baumannii SDF | p1ABSDF | 1.0E-39 | 97.03 | 101 | 1 |
| NC_010396.1 | Acinetobacter baumannii SDF | p2ABSDF | 2.0E-36 | 95.05 | 98 | 49 |
| NC_010398.1 | Acinetobacter baumannii SDF | p3ABSDF | 4.0E-33 | 95 | 90 | 54 |
| NC_020818.1 | Acinetobacter baumannii strain GF216 | pNDM-AB | 6.0E-34 | 96.04 | 91 | 69 |
| NC_019985.2 | Acinetobacter baumannii strain ZW85-1 | pAbNDM-1 | 9.0E-32 | 95.05 | 90 | 14 |
| NC_017166.1 | Acinetobacter baumannii TCDC-AB0715 | p2ABTCDC0715 | 2.0E-35 | 95.1 | 101 | 9 |
| NC_023031.1 | Acinetobacter baumannii ZW85-1 | ZW85p2 | 1.0E-33 | 95 | 92 | 96 |
| NC_023322.1 | Acinetobacter bereziniae strain CHI-40-1 | pNDM-BJ01 | 9.0E-38 | 96.04 | 101 | 17 |
| NC_013056.1 | Acinetobacter calcoaceticus strain Acal H12O-07 | pMMCU1 | 3.0E-36 | 95 | 100 | 5 |
| NC_019323.1 | Acinetobacter lwoffii | pABZ78 | 4.0E-37 | 95.05 | 96 | 3 |
| NC_019268.1 | Acinetobacter lwoffii | pNDM-BJ01 | 2.0E-41 | 99.01 | 101 | 9 |
| NC_023280.1 | Acinetobacter nosocomialis | pRAY*-v3 | 2.0E-36 | 95 | 99 | 14 |
| NC_019322.1 | Acinetobacter pittii | pABCA95 | 1.0E-36 | 98.02 | 90 | 62 |
| NC_000923.1 | Acinetobacter sp. SUN resistance | pRAY | 5.0E-37 | 95.05 | 101 | 1 |
| NC_010309.1 | Acinetobacter venetianus strain VE-C3 | pAV1 | 6.0E-33 | 95 | 91 | 72 |
| NC_010310.1 | Acinetobacter venetianus strain VE-C3 | pAV2 | 7.0E-34 | 95.05 | 90 | 200 |
| NC_007098.1 | Actinobacillus pleuropneumoniae pKMA2425 |  | 2.0E-44 | 100 | 101 | 2 |
| NC_006143.1 | Aeromonas caviae | pFBAOT6 | 2.0E-35 | 95 | 91 | 97 |
| NC_013780.1 | Aeromonas hydrophila | pAH3680 | 3.0E-44 | 100 | 101 | 1 |
| NC_021159.1 | Aeromonas hydrophila | pAhy2.5 | 4.0E-40 | 97.03 | 101 | 2 |
| NC_011207.1 | Aeromonas hydrophila | pBRST7.6 | 2.0E-38 | 96.04 | 101 | 1 |
| NC_003124.1 | Aeromonas salmonicida | pRAS3.2 | 2.0E-38 | 96.04 | 99 | 5 |
| NC_009349.1 | Aeromonas salmonicida subsp. salmonicida A449 | 4 | 6.0E-34 | 95.05 | 93 | 23 |
| NC_009350.1 | Aeromonas salmonicida subsp. salmonicida A449 | 5 | 1.0E-42 | 100 | 101 | 2 |
| NC_004339.1 | Aeromonas salmonicida subsp. salmonicida pAsal2 |  | 2.0E-36 | 95.1 | 99 | 5 |
| NC_003123.1 | Aeromonas salmonicida subsp. salmonicida | pRAS3.1 | 9.0E-44 | 100 | 101 | 1 |
| NC_014629.1 | Aggregatibacter actinomycetemcomitans D11S-1 | S57 | 4.0E-39 | 97.03 | 101 | 2 |
| NC_011987.1 | Agrobacterium radiobacter K84 | pAtK84c | 4.0E-34 | 95.05 | 101 | 1 |
| NC_015184.1 | Agrobacterium sp. H13-3 | pAspH13-3a | 4.0E-42 | 100 | 101 | 1 |
| NC_002147.1 | Agrobacterium tumefaciens MAFF301001 | pTi-SAKURA | 1.0E-42 | 100 | 101 | 1 |
| NC_019555.1 | Agrobacterium tumefaciens | pAoF64/95 | 7.0E-40 | 98.02 | 101 | 1 |
| NC_002377.1 | Agrobacterium tumefaciens | Ti | 6.0E-35 | 95.88 | 97 | 1 |
| NC_010929.1 | Agrobacterium tumefaciens Ti | pTiBo542 | 5.0E-37 | 96.04 | 101 | 2 |
| NC_011986.1 | Agrobacterium vitis S4 | pAtS4a | 7.0E-36 | 95.05 | 101 | 4 |
| NC_011984.1 | Agrobacterium vitis S4 | pAtS4c | 4.0E-37 | 96.04 | 101 | 2 |
| NC_011982.1 | Agrobacterium vitis S4 | pTiS4 | 2.0E-35 | 95.05 | 100 | 8 |
| NC_014908.1 | Alicycliphilus denitrificans BC | pALIDE01 | 2.0E-32 | 95.05 | 90 | 16 |
| NC_014911.1 | Alicycliphilus denitrificans BC | pALIDE02 | 1.0E-32 | 95.05 | 92 | 26 |
| NC_015423.1 | Alicycliphilus denitrificans K601 | pALIDE201 | 1.0E-37 | 96.04 | 101 | 10 |
| NC_021709.1 | Alteromonas macleodii str. 'English Channel 615' |  | 1.0E-42 | 100 | 101 | 6 |
| NC_013164.1 | Anaerococcus prevotii DSM 20548 | pAPRE01 | 2.0E-37 | 96.04 | 99 | 15 |
| NC_008712.1 | Arthrobacter aurescens TC1 | TC1 | 9.0E-35 | 95.05 | 90 | 92 |
| NC_006823.1 | Azoarcus sp. EbN1 | 1 | 2.0E-35 | 95.05 | 98 | 28 |
| NC_016594.1 | Azospirillum brasilense Sp245 | AZOBR_p1 | 9.0E-31 | 95.05 | 91 | 5 |
| NC_016618.1 | Azospirillum brasilense Sp245 | AZOBR_p2 | 4.0E-32 | 95.05 | 93 | 2 |
| NC_016596.1 | Azospirillum brasilense Sp245 | AZOBR_p4 | 6.0E-35 | 95.05 | 99 | 4 |
| NC_016585.1 | Azospirillum lipoferum 4B | AZO_p1 | 1.0E-33 | 95.05 | 94 | 5 |
| NC_016624.1 | Azospirillum lipoferum 4B | AZO_p5 | 9.0E-31 | 95 | 90 | 12 |
| NC_013855.1 | Azospirillum sp. B510 | pAB510a | 3.0E-30 | 95.05 | 90 | 35 |
| NC_013857.1 | Azospirillum sp. B510 | pAB510c | 2.0E-34 | 95 | 100 | 1 |
| NC_013858.1 | Azospirillum sp. B510 | pAB510d | 6.0E-34 | 95.05 | 96 | 2 |
| NC_013859.1 | Azospirillum sp. B510 | pAB510e | 4.0E-35 | 95.05 | 101 | 1 |
| NC_001496.1 | Bacillus anthracis virulence | pX01 | 6.0E-41 | 99.01 | 101 | 1 |
| NC_012473.1 | Bacillus cereus 03BB102 | p03BB102_179 | 3.0E-37 | 96.04 | 101 | 3 |
| NC_011657.1 | Bacillus cereus AH187 | pAH187_3 | 2.0E-44 | 100 | 101 | 1 |
| NC_007103.1 | Bacillus cereus E33L | pE33L466 | 4.0E-35 | 95.05 | 101 | 1 |
| NC_018492.1 | Bacillus cereus FRI-35 | p01 | 8.0E-40 | 99.01 | 101 | 2 |
| NC_010924.1 | Bacillus cereus strain AH187 | pCER270 | 1.0E-39 | 98.02 | 101 | 2 |
| NC_004604.2 | Bacillus megaterium QM B1551 | pBM400 | 2.0E-29 | 95 | 90 | 481 |
| NC_017139.1 | Bacillus megaterium WSH-002 | WSH-002_p1 | 2.0E-29 | 95.05 | 90 | 224 |
| NC_013963.1 | Bacillus sp. BS-01 | pBS-01 | 5.0E-42 | 99.01 | 101 | 17 |
| NC_014557.1 | Bacillus sp. BS-02 | pBS-02 | 5.0E-42 | 99.01 | 101 | 4 |
| NC_014172.1 | Bacillus thuringiensis BMB171 | pBMB171 | 5.0E-38 | 97.03 | 101 | 3 |
| NC_014937.1 | Bacillus thuringiensis CT43 | pBMB0558 | 3.0E-35 | 95.05 | 90 | 34 |
| NC_018689.1 | Bacillus thuringiensis MC28 | pMC429 | 3.0E-36 | 96.94 | 98 | 1 |
| NC_023074.1 | Bacillus thuringiensis serovar tenebrionis str. YBT-1765 | pBMB165 | 2.0E-37 | 96.04 | 101 | 1 |
| NC_019783.1 | Bacterium 36B | pTOR_02 | 2.0E-37 | 96.04 | 90 | 18 |
| NC_019798.1 | Bacterium 72B | pTOR_01 | 1.0E-38 | 97.03 | 92 | 5 |
| NC_005026.1 | Bacteroides fragilis IB143 | pBI143 | 2.0E-37 | 95.05 | 94 | 36 |
| NC_006873.1 | Bacteroides fragilis NCTC 9343 | pBF9343 | 1.0E-34 | 95.05 | 93 | 62 |
| NC_011073.1 | Bacteroides fragilis | pBFP35 | 2.0E-42 | 99.01 | 101 | 32 |
| NC_019534.1 | Bacteroides fragilis | pBFUK1 | 1.0E-36 | 95.05 | 91 | 78 |
| NC_006297.1 | Bacteroides fragilis YCH46 | pBFY46 | 2.0E-39 | 98.97 | 95 | 2 |
| NC_015166.1 | Bacteroides salanitronis DSM 18170 | pBACSA03 | 6.0E-37 | 97.85 | 93 | 3 |
| NC_004703.1 | Bacteroides thetaiotaomicron VPI-5482 | p5482 | 2.0E-37 | 96 | 92 | 45 |
| NC_007068.1 | Bifidobacterium catenulatum | pBC1 | 2.0E-44 | 100 | 101 | 1 |
| NC_021875.1 | Bifidobacterium kashiwanohense JCM 15439 | pBBKW-1 DNA | 2.0E-32 | 95.56 | 90 | 2 |
| NC_021876.1 | Bifidobacterium kashiwanohense JCM 15439 | pBBKW-2 DNA | 6.0E-39 | 96.97 | 99 | 2 |
| NC_004252.1 | Bifidobacterium longum DJO10A | pDOJH10L | 1.0E-34 | 97.8 | 91 | 29 |
| NC_004253.1 | Bifidobacterium longum DJO10A | pDOJH10S | 6.0E-40 | 97.03 | 101 | 6 |
| NC_002635.1 | Bifidobacterium longum KJ | pKJ36 | 2.0E-39 | 97.06 | 101 | 9 |
| NC_004978.1 | Bifidobacterium longum KJ | pKJ50 | 4.0E-44 | 100 | 101 | 4 |
| NC_010857.1 | Bifidobacterium longum | p6043A | 2.0E-42 | 99.01 | 101 | 6 |
| NC_010861.1 | Bifidobacterium longum | p6043B | 3.0E-44 | 100 | 101 | 1 |
| NC_011139.1 | Bifidobacterium longum | pFI2576 | 9.0E-42 | 99.01 | 100 | 11 |
| NC_006997.1 | Bifidobacterium longum | pMG1 | 1.0E-42 | 99.01 | 101 | 5 |
| NC_019200.1 | Bifidobacterium longum | pSP02 | 4.0E-44 | 100 | 101 | 1 |
| NC_006843.1 | Bifidobacterium longum | pTB6 | 8.0E-39 | 96.04 | 101 | 2 |
| NC_004769.1 | Bifidobacterium longum RW041 | pNAC2 | 3.0E-44 | 100 | 101 | 9 |
| NC_004768.1 | Bifidobacterium longum RW041 | pNAC3 | 1.0E-35 | 95.05 | 93 | 89 |
| NC_004770.1 | Bifidobacterium longum RW048 | pNAC1 | 3.0E-37 | 95.05 | 99 | 34 |
| NC_015053.1 | Bifidobacterium longum subsp. infantis 157F | p157F-NC1 | 1.0E-38 | 98.02 | 91 | 72 |
| NC_015066.1 | Bifidobacterium longum subsp. infantis 157F | p157F-NC2 | 1.0E-42 | 99.01 | 101 | 5 |
| NC_017220.1 | Bifidobacterium longum subsp. longum KACC 91563 | BLNIAS_P1 | 2.0E-41 | 98.99 | 99 | 2 |
| NC_017222.1 | Bifidobacterium longum subsp. longum KACC 91563 | BLNIAS_P2 | 1.0E-39 | 97.03 | 99 | 10 |
| NC_004443.1 | Bifidobacterium longum VMKB44 | pB44 | 3.0E-44 | 100 | 101 | 1 |
| NC_022590.1 | Brevibacterium sp. Ap13 | pAP13 | 7.0E-36 | 96.88 | 96 | 3 |
| NC_008385.1 | Burkholderia cepacia AMMD | 1 | 3.0E-37 | 98.02 | 90 | 81 |
| NC_019378.1 | Burkholderia cepacia | pIJB1 | 8.0E-36 | 95.05 | 92 | 17 |
| NC_019369.1 | Burkholderia cepacia | pYS1 | 7.0E-36 | 95.05 | 101 | 2 |
| NC_022995.1 | Burkholderia sp. M701 | pM7012 DNA | 1.0E-36 | 96.04 | 101 | 2 |
| NC_016591.1 | Burkholderia sp. YI23 | byi_2p | 2.0E-32 | 95.7 | 93 | 89 |
| NC_009230.1 | Burkholderia vietnamiensis G4 | pBVIE01 | 3.0E-35 | 95.05 | 99 | 2 |
| NC_009227.1 | Burkholderia vietnamiensis G4 | pBVIE02 | 2.0E-35 | 95.05 | 99 | 22 |
| NC_022355.1 | Campylobacter coli CVM N29710 | pN29710-1 | 1.0E-36 | 95.96 | 92 | 35 |
| NC_006134.1 | Campylobacter coli | pCC31 | 8.0E-32 | 95 | 90 | 511 |
| NC_022354.1 | Campylobacter jejuni subsp. jejuni 00-2544 |  | 6.0E-34 | 95 | 92 | 54 |
| NC_007141.1 | Campylobacter jejuni subsp. jejuni 81-176 | pTet | 3.0E-38 | 98.02 | 92 | 33 |
| NC_014801.1 | Campylobacter jejuni subsp. jejuni ICDCCJ07001 | pTet | 1.0E-35 | 95.05 | 92 | 152 |
| NC_013193.1 | Candidatus Accumulibacter phosphatis clade IIA str. UW-1 | pAph01 | 6.0E-40 | 98.02 | 101 | 4 |
| NC_013190.1 | Candidatus Accumulibacter phosphatis clade IIA str. UW-1 | pAph02 | 1.0E-41 | 99.01 | 101 | 4 |
| NC_013191.1 | Candidatus Accumulibacter phosphatis clade IIA str. UW-1 | pAph03 | 9.0E-37 | 95.96 | 99 | 2 |
| NC_022601.1 | Carnobacterium sp. WN1359 | pWNCR12 | 3.0E-38 | 96.97 | 94 | 6 |
| NC_022602.1 | Carnobacterium sp. WN1359 | pWNCR47 | 4.0E-36 | 95.05 | 99 | 15 |
| NC_022603.1 | Carnobacterium sp. WN1359 | pWNCR64 | 1.0E-38 | 96.15 | 101 | 6 |
| NC_010333.1 | Caulobacter sp. K31 | pCAUL02 | 2.0E-40 | 99 | 100 | 1 |
| NC_022552.1 | Citrobacter freundii NDMCF | pNDMCFuy | 2.0E-33 | 95.05 | 90 | 115 |
| NC_004464.2 | Citrobacter freundii | pCTX-M3 | 1.0E-38 | 97.03 | 101 | 1 |
| NC_019360.1 | Citrobacter freundii | pNDM-CIT | 9.0E-41 | 99.01 | 101 | 3 |
| NC_020122.1 | Citrobacter freundii strain CFSTE | pN-Cit | 8.0E-37 | 95.96 | 99 | 1 |
| NC_009793.1 | Citrobacter koseri ATCC BAA-895 | pCKO3 | 7.0E-44 | 100 | 101 | 7 |
| NC_013717.1 | Citrobacter rodentium ICC168 | pCROD1 | 1.0E-37 | 96.04 | 101 | 1 |
| NC_007772.1 | Clostridium perfringens CPE str. F4969 | pCPF4969 | 5.0E-43 | 100 | 101 | 1 |
| NC_010937.1 | Clostridium perfringens | pCW3 | 1.0E-37 | 96.04 | 101 | 8 |
| NC_019259.1 | Clostridium perfringens | pJIR3537 | 2.0E-41 | 99.01 | 101 | 3 |
| NC_003042.1 | Clostridium perfringens str. 13 | pCP13 | 2.0E-41 | 99.01 | 101 | 1 |
| NC_021652.1 | Clostridium thermocellum strain BL21 | pEBM107 | 4.0E-41 | 98.02 | 101 | 2 |
| NC_010332.1 | Collimonas fungivorans | pTer331 | 8.0E-32 | 95.56 | 90 | 2 |
| NC_021077.1 | Comamonas sp. 7D-2 | pBHB | 8.0E-37 | 97 | 95 | 38 |
| NC_010935.1 | Comamonas testosteroni CNB-1 | pCNB | 3.0E-35 | 95.96 | 90 | 96 |
| NC_016978.1 | Comamonas testosteroni | pI2 | 2.0E-37 | 96.04 | 99 | 8 |
| NC_002143.1 | Comamonas testosteroni PtL5 cryptic | pPT1 | 1.0E-36 | 95.05 | 99 | 10 |
| NC_020553.1 | Corynebacterium callunae DSM 20147 | pCC2 | 1.0E-38 | 97.03 | 101 | 2 |
| NC_005001.1 | Corynebacterium diphtheriae | pNG2 | 2.0E-40 | 99.01 | 95 | 6 |
| NC_001791.1 | Corynebacterium glutamicum strain 1014 | pXZ10145.1 | 2.0E-41 | 98.02 | 101 | 1 |
| NC_004945.1 | Corynebacterium glutamicum strain ATCC31830 | pCG4 | 1.0E-41 | 99.01 | 101 | 1 |
| NC_003490.1 | Corynebacterium jeikeium | pB85766 | 1.0E-43 | 100 | 101 | 2 |
| NC_021920.1 | Corynebacterium maris DSM 45190 | pCmaris1 | 7.0E-39 | 97.03 | 101 | 1 |
| NC_014167.1 | Corynebacterium resistens DSM 45100 | pJA144188 | 2.0E-37 | 97.03 | 90 | 23 |
| NC_009129.1 | Corynebacterium sp. L2-79-05 | pLEW279b | 1.0E-40 | 100 | 96 | 1 |
| NC_004939.1 | Corynebacterium striatum strain M82B R- | pTP10 | 4.0E-43 | 100 | 101 | 2 |
| NC_009779.1 | Cronobacter sakazakii ATCC BAA-894 | pESA2 | 3.0E-36 | 95.05 | 90 | 50 |
| NC_023025.1 | Cronobacter sakazakii CMCC 45402 | p2 | 2.0E-41 | 99.01 | 101 | 14 |
| NC_020261.1 | Cronobacter sakazakii Sp291 | pSP291-2 | 2.0E-40 | 98.02 | 101 | 2 |
| NC_021293.1 | Cronobacter sakazakii strain ATCC 29544 | pCSA2 | 4.0E-44 | 100 | 101 | 2 |
| NC_013285.1 | Cronobacter turicensis z3032 | pCTU3 | 4.0E-43 | 100 | 101 | 2 |
| NC_007974.2 | Cupriavidus metallidurans CH34 |  | 1.0E-30 | 95 | 90 | 63 |
| NC_006525.1 | Cupriavidus metallidurans CH34 | pMOL28 | 2.0E-33 | 95 | 94 | 5 |
| NC_006466.1 | Cupriavidus metallidurans CH34 | pMOL30 | 2.0E-42 | 100 | 101 | 1 |
| NC_015727.1 | Cupriavidus necator N-1 | BB1p | 1.0E-33 | 95.05 | 96 | 4 |
| NC_012527.1 | Deinococcus deserti VCD115 | 1 | 6.0E-31 | 95 | 90 | 47 |
| NC_008010.2 | Deinococcus geothermalis DSM 11300 | pDGEO01 | 2.0E-33 | 95.05 | 90 | 22 |
| NC_019789.1 | Deinococcus peraridilitoris DSM 19664 | pDEIPE01 | 1.0E-29 | 95 | 90 | 29 |
| NC_000958.1 | Deinococcus radiodurans R1 | MP1 | 1.0E-35 | 95.05 | 101 | 5 |
| NC_005088.1 | Delftia acidovorans B | pUO1 | 2.0E-34 | 95.05 | 95 | 17 |
| NC_019264.1 | Delftia acidovorans | pNB8c | 5.0E-43 | 100 | 101 | 1 |
| NC_019312.1 | Delftia sp. KV29 | pKV29 | 3.0E-32 | 95 | 90 | 324 |
| NC_009955.1 | Dinoroseobacter shibae DFL 12 | pDSHI01 | 9.0E-39 | 97.98 | 99 | 3 |
| NC_009957.1 | Dinoroseobacter shibae DFL 12 | pDSHI03 | 1.0E-35 | 95.05 | 101 | 13 |
| NC_009958.1 | Dinoroseobacter shibae DFL 12 | pDSHI04 | 2.0E-37 | 96.04 | 101 | 1 |
| NC_020280.1 | Edwardsiella ictaluri | pEI3 | 2.0E-40 | 99.01 | 94 | 5 |
| NC_014725.1 | Edwardsiella tarda strain CK41 | pCK41 | 5.0E-43 | 100 | 101 | 2 |
| NC_018742.1 | Emticicia oligotrophica DSM 17448 | pEMTOL01 | 7.0E-40 | 98.02 | 101 | 1 |
| NC_001735.4 | Enterobacter aerogenes | R751 | 4.0E-43 | 100 | 101 | 2 |
| NC_016515.1 | Enterobacter cloacae EcWSU1 | pEcWSU1_A | 1.0E-38 | 97.03 | 101 | 2 |
| NC_011404.1 | Enterobacter cloacae | pEC01 | 4.0E-44 | 100 | 101 | 1 |
| NC_012555.1 | Enterobacter cloacae | pEC-IMP | 2.0E-36 | 96 | 93 | 16 |
| NC_019242.1 | Enterobacter cloacae | pS51B | 2.0E-42 | 99.01 | 101 | 2 |
| NC_017097.1 | Enterobacter cloacae | pUL3AT | 3.0E-42 | 99.01 | 101 | 3 |
| NC_014107.1 | Enterobacter cloacae subsp. cloacae ATCC 13047 | pECL_A | 4.0E-37 | 96.04 | 101 | 3 |
| NC_021492.1 | Enterobacter sp. R4-368 | pENT01 | 2.0E-38 | 97.03 | 101 | 1 |
| NC_019240.1 | Enterococcus durans | pGL | 6.0E-44 | 100 | 101 | 1 |
| NC_017314.1 | Enterococcus faecalis 62 | EF62pA | 2.0E-41 | 98.02 | 101 | 3 |
| NC_017313.1 | Enterococcus faecalis 62 | EF62pB | 2.0E-41 | 99.01 | 101 | 1 |
| NC_017315.1 | Enterococcus faecalis 62 | EF62pC | 9.0E-39 | 98.02 | 93 | 6 |
| NC_018222.1 | Enterococcus faecalis D32 | EFD32pA | 5.0E-41 | 98.02 | 101 | 2 |
| NC_005013.1 | Enterococcus faecalis DS5 | pAMalpha1 | 7.0E-44 | 100 | 101 | 1 |
| NC_013514.1 | Enterococcus faecalis | pAMbeta1 | 1.0E-40 | 98.02 | 99 | 16 |
| NC_013533.1 | Enterococcus faecalis | pBEE99 | 5.0E-37 | 96 | 100 | 1 |
| NC_006827.2 | Enterococcus faecalis | pCF10 | 7.0E-35 | 95.05 | 94 | 41 |
| NC_014508.2 | Enterococcus faecalis | pEF-01 | 1.0E-41 | 99.01 | 101 | 10 |
| NC_011642.1 | Enterococcus faecalis | pMG2200 | 4.0E-34 | 95.05 | 92 | 9 |
| NC_014726.1 | Enterococcus faecalis | pTW9 | 2.0E-35 | 96.04 | 92 | 12 |
| NC_014475.1 | Enterococcus faecalis | pWZ1668 | 4.0E-43 | 100 | 101 | 1 |
| NC_019284.1 | Enterococcus faecalis | pWZ7140 | 4.0E-43 | 100 | 101 | 4 |
| NC_019213.1 | Enterococcus faecalis | pWZ909 | 6.0E-40 | 99.01 | 95 | 8 |
| NC_008445.1 | Enterococcus faecalis RE25 | pRE25 | 2.0E-40 | 98.02 | 97 | 16 |
| NC_004669.1 | Enterococcus faecalis V583 | pTEF1 | 3.0E-40 | 98.02 | 101 | 2 |
| NC_021987.1 | Enterococcus faecium Aus0085 | p1 | 1.0E-34 | 95 | 94 | 109 |
| NC_021995.1 | Enterococcus faecium Aus0085 | p2 | 3.0E-39 | 100 | 94 | 2 |
| NC_021988.1 | Enterococcus faecium Aus0085 | p3 | 9.0E-36 | 95 | 100 | 5 |
| NC_017961.1 | Enterococcus faecium DO | 1 | 3.0E-36 | 95.05 | 98 | 17 |
| NC_017962.1 | Enterococcus faecium DO | 2 | 5.0E-37 | 97.89 | 95 | 8 |
| NC_017963.1 | Enterococcus faecium DO | 3 | 2.0E-35 | 95.05 | 95 | 29 |
| NC_020208.1 | Enterococcus faecium NRRL B-2354 | pNB2354_1 | 2.0E-34 | 95.83 | 95 | 43 |
| NC_013317.1 | Enterococcus faecium | p5753cA | 2.0E-37 | 99.01 | 90 | 27 |
| NC_021170.1 | Enterococcus faecium | pF856 | 3.0E-36 | 95.05 | 98 | 5 |
| NC_011140.1 | Enterococcus faecium | pIP816 | 1.0E-41 | 99.01 | 101 | 2 |
| NC_016009.1 | Enterococcus faecium | pM7M2 | 1.0E-43 | 100 | 101 | 5 |
| NC_011364.1 | Enterococcus faecium | pMG1 | 5.0E-43 | 100 | 101 | 1 |
| NC_014959.1 | Enterococcus faecium | pS177 | 1.0E-40 | 98.02 | 101 | 31 |
| NC_008768.1 | Enterococcus faecium | pVEF1 | 7.0E-39 | 97.03 | 101 | 4 |
| NC_010980.1 | Enterococcus faecium | pVEF3 | 2.0E-35 | 95 | 100 | 21 |
| NC_017627.1 | Escherichia coli 042 | pAA | 2.0E-37 | 97.03 | 101 | 4 |
| NC_010719.1 | Escherichia coli 53638 | p53638_226 | 8.0E-40 | 98.99 | 99 | 9 |
| NC_011752.1 | Escherichia coli 55989 | 55989p | 3.0E-40 | 98.02 | 101 | 1 |
| NC_007675.1 | Escherichia coli A2363 | pAPEC-O2-ColV | 4.0E-37 | 96.04 | 92 | 10 |
| NC_006671.1 | Escherichia coli A2363 | pAPEC-O2-R | 3.0E-41 | 99.01 | 101 | 4 |
| NC_017629.1 | Escherichia coli ABU 83972 | pABU | 2.0E-42 | 99.02 | 101 | 9 |
| NC_023323.1 | Escherichia coli ACN001 | pACN001-A | 2.0E-40 | 98.02 | 101 | 1 |
| NC_023327.1 | Escherichia coli ACN001 | pACN001-B | 3.0E-38 | 97.03 | 101 | 20 |
| NC_009837.1 | Escherichia coli APEC O1 | pAPEC-O1-ColBM | 1.0E-42 | 100 | 101 | 3 |
| NC_009838.1 | Escherichia coli APEC O1 | pAPEC-O1-R | 5.0E-37 | 96.04 | 101 | 21 |
| NC_002142.1 | Escherichia coli B171 | pB171 | 4.0E-38 | 97 | 100 | 3 |
| NC_011980.1 | Escherichia coli chi7122 | pAPEC-1 | 7.0E-37 | 98.02 | 90 | 37 |
| NC_009787.1 | Escherichia coli E24377A | pETEC_35 | 3.0E-43 | 100 | 101 | 2 |
| NC_009788.1 | Escherichia coli E24377A | pETEC_73 | 2.0E-41 | 99.01 | 101 | 11 |
| NC_009790.1 | Escherichia coli E24377A | pETEC_74 | 5.0E-43 | 100 | 101 | 2 |
| NC_009786.1 | Escherichia coli E24377A | pETEC_80 | 6.0E-43 | 100 | 101 | 1 |
| NC_011754.1 | Escherichia coli ED1a | pECOED | 1.0E-35 | 95.05 | 99 | 6 |
| NC_007365.1 | Escherichia coli EH41 | pO113 | 1.0E-36 | 96.04 | 90 | 20 |
| NC_014232.1 | Escherichia coli ETEC 1392/75 | p1081 | 7.0E-43 | 100 | 101 | 1 |
| NC_014233.1 | Escherichia coli ETEC 1392/75 | p557 | 9.0E-39 | 97.03 | 101 | 15 |
| NC_014234.1 | Escherichia coli ETEC 1392/75 | p746 | 3.0E-39 | 99.01 | 94 | 4 |
| NC_014235.1 | Escherichia coli ETEC 1392/75 | p75 | 6.0E-44 | 100 | 101 | 1 |
| NC_017724.1 | Escherichia coli ETEC H10407 | p948 | 3.0E-41 | 99.01 | 101 | 2 |
| NC_022742.1 | Escherichia coli HUSEC2011 | pHUSEC2011-1 | 7.0E-43 | 100 | 101 | 1 |
| NC_015599.1 | Escherichia coli IncN | N3 | 2.0E-40 | 98.02 | 101 | 1 |
| NC_022661.1 | Escherichia coli JJ1886 | pJJ1886_1 | 1.0E-44 | 100 | 101 | 5 |
| NC_022649.1 | Escherichia coli JJ1886 | pJJ1886_2 | 4.0E-44 | 100 | 101 | 1 |
| NC_022650.1 | Escherichia coli JJ1886 | pJJ1886_4 | 5.0E-36 | 95.05 | 101 | 4 |
| NC_022651.1 | Escherichia coli JJ1886 | pJJ1886_5 | 2.0E-38 | 97.03 | 101 | 2 |
| NC_002483.1 | Escherichia coli K-12 | F DNA | 2.0E-38 | 97.03 | 101 | 11 |
| NC_002525.1 | Escherichia coli K-12 | R721 | 6.0E-36 | 95.92 | 92 | 22 |
| NC_016904.1 | Escherichia coli KO11FL | pEKO1101 | 8.0E-43 | 100 | 101 | 9 |
| NC_011917.1 | Escherichia coli LF82 | plLF82 | 2.0E-37 | 96.04 | 100 | 24 |
| NC_013354.1 | Escherichia coli O103:H2 str. 12009 | pO103 | 1.0E-39 | 98 | 100 | 4 |
| NC_018651.1 | Escherichia coli O104:H4 str. 2009EL-2050 | p09EL50 | 2.0E-38 | 97.03 | 99 | 12 |
| NC_013370.1 | Escherichia coli O111:H- str. 11128 | pO111_2 | 8.0E-36 | 95.05 | 99 | 15 |
| NC_013366.1 | Escherichia coli O111:H- str. 11128 | pO111_3 | 2.0E-41 | 99.01 | 101 | 2 |
| NC_011603.1 | Escherichia coli O127:H6 str. E2348/69 | pMAR2 | 3.0E-41 | 99.01 | 101 | 1 |
| NC_007414.1 | Escherichia coli O157:H7 EDL933 | pO157 | 3.0E-41 | 99.01 | 101 | 1 |
| NC_002128.1 | Escherichia coli O157:H7 str. Sakai | pO157 | 3.0E-41 | 99.01 | 101 | 1 |
| NC_019087.1 | Escherichia coli O25b:H4-ST131 str. EC958 | pKC396 | 3.0E-43 | 100 | 101 | 1 |
| NC_020271.1 | Escherichia coli O25b:H4-ST131 str. EC958 strain ST131 | pJIE186-2 | 1.0E-41 | 100 | 99 | 1 |
| NC_013362.1 | Escherichia coli O26:H11 str. 11368 | pO26_2 | 5.0E-43 | 100 | 101 | 3 |
| NC_013942.1 | Escherichia coli O55:H7 str. CB9615 | pO55 | 2.0E-41 | 99.01 | 101 | 6 |
| NC_017653.1 | Escherichia coli O55:H7 str. RM12579 | p12579_1 | 4.0E-40 | 98.02 | 101 | 6 |
| NC_017647.1 | Escherichia coli O7:K1 str. CE10 | pCE10A | 1.0E-37 | 96.04 | 101 | 6 |
| NC_014356.1 | Escherichia coli | IncQ-typepQ7 | 2.0E-39 | 97.03 | 101 | 1 |
| NC_010257.1 | Escherichia coli | MccC7-H22 | 2.0E-43 | 100 | 101 | 3 |
| NC_004998.1 | Escherichia coli | p1658/97 | 2.0E-38 | 97.03 | 101 | 2 |
| NC_015872.1 | Escherichia coli | p271A | 6.0E-39 | 97.03 | 93 | 5 |
| NC_019094.1 | Escherichia coli | p417H-90 | 6.0E-43 | 100 | 101 | 5 |
| NC_019062.1 | Escherichia coli | p838C-R1 | 1.0E-41 | 99.01 | 101 | 4 |
| NC_005324.1 | Escherichia coli | p9123 | 5.0E-44 | 100 | 101 | 2 |
| NC_019056.1 | Escherichia coli | pAm08CD9902 | 3.0E-42 | 99.01 | 101 | 1 |
| NC_011964.1 | Escherichia coli | pAPEC-O103-ColBM | 4.0E-41 | 99.01 | 99 | 3 |
| NC_019037.1 | Escherichia coli | pChi7122-2 | 1.0E-38 | 97.03 | 100 | 6 |
| NC_019039.1 | Escherichia coli | pChi7122-3 | 9.0E-39 | 98.02 | 96 | 19 |
| NC_019049.1 | Escherichia coli | pCM959 | 4.0E-37 | 95.05 | 93 | 22 |
| NC_006881.1 | Escherichia coli | pColK-K235 | 6.0E-44 | 100 | 101 | 1 |
| NC_007635.1 | Escherichia coli | pCoo | 7.0E-43 | 100 | 101 | 6 |
| NC_014477.1 | Escherichia coli | pCT | 7.0E-43 | 100 | 101 | 1 |
| NC_014382.1 | Escherichia coli | pEC_B24 | 2.0E-41 | 99.01 | 101 | 4 |
| NC_013175.1 | Escherichia coli | pEC14_114 | 8.0E-43 | 100 | 101 | 1 |
| NC_021997.1 | Escherichia coli | pEC386IL | 3.0E-43 | 100 | 101 | 1 |
| NC_021999.1 | Escherichia coli | pEC386IL | 2.0E-44 | 100 | 101 | 3 |
| NC_015472.1 | Escherichia coli | pECTm80 | 2.0E-43 | 100 | 101 | 1 |
| NC_012690.1 | Escherichia coli | peH4H | 5.0E-41 | 99.01 | 101 | 7 |
| NC_013120.1 | Escherichia coli | pEK204 | 7.0E-43 | 100 | 101 | 1 |
| NC_013122.1 | Escherichia coli | pEK499 | 4.0E-41 | 99.01 | 101 | 2 |
| NC_013121.1 | Escherichia coli | pEK516 | 3.0E-40 | 99.01 | 96 | 5 |
| NC_014615.1 | Escherichia coli | pETN48 | 1.0E-42 | 100 | 101 | 1 |
| NC_005923.1 | Escherichia coli | pFL129 | 9.0E-41 | 100 | 95 | 1 |
| NC_019089.1 | Escherichia coli | pGUE-NDM | 6.0E-43 | 100 | 101 | 2 |
| NC_016039.1 | Escherichia coli | pHK17a | 5.0E-43 | 100 | 101 | 1 |
| NC_020270.1 | Escherichia coli | pHN1122-1 | 2.0E-40 | 98.02 | 101 | 3 |
| NC_018995.1 | Escherichia coli | pHUSEC41-1 | 7.0E-43 | 100 | 101 | 1 |
| NC_019000.1 | Escherichia coli | pHUSEC41-2 | 2.0E-41 | 99.01 | 101 | 4 |
| NC_010885.1 | Escherichia coli | pIGWZ12 | 3.0E-44 | 100 | 101 | 2 |
| NC_014231.1 | Escherichia coli | pKC394 | 4.0E-43 | 100 | 101 | 1 |
| NC_019097.1 | Escherichia coli | plm | 7.0E-43 | 100 | 101 | 3 |
| NC_010064.1 | Escherichia coli | pLMO226 | 2.0E-44 | 100 | 101 | 4 |
| NC_019093.1 | Escherichia coli | pLST424C-61 | 1.0E-37 | 98.94 | 94 | 5 |
| NC_008486.1 | Escherichia coli | pMG828-1 | 8.0E-42 | 98.02 | 101 | 2 |
| NC_008489.1 | Escherichia coli | pMG828-4 | 6.0E-44 | 100 | 101 | 1 |
| NC_008490.1 | Escherichia coli | pMG828-5 | 5.0E-33 | 95.6 | 91 | 5 |
| NC_007682.3 | Escherichia coli | pMUR050 | 1.0E-37 | 96.04 | 101 | 1 |
| NC_019043.1 | Escherichia coli | pND11_107 | 2.0E-38 | 97.03 | 101 | 3 |
| NC_018994.1 | Escherichia coli | pNDM-1_Dok01 | 4.0E-37 | 96.04 | 99 | 3 |
| NC_019069.1 | Escherichia coli | pNDM10505 | 5.0E-35 | 95 | 100 | 13 |
| NC_022375.1 | Escherichia coli | pNDM-BTR | 4.0E-43 | 100 | 101 | 1 |
| NC_022992.1 | Escherichia coli | pO111-CRL-115 | 1.0E-34 | 96.04 | 91 | 30 |
| NC_022996.1 | Escherichia coli | pO26-CRL-125 | 6.0E-39 | 98.97 | 97 | 22 |
| NC_011812.1 | Escherichia coli | pO26-L | 4.0E-38 | 100 | 92 | 3 |
| NC_012487.1 | Escherichia coli | pO26-Vir | 5.0E-41 | 99.01 | 101 | 1 |
| NC_008460.1 | Escherichia coli | pO86A1 | 9.0E-43 | 100 | 101 | 2 |
| NC_010378.1 | Escherichia coli | pOLA52 | 7.0E-40 | 98.04 | 101 | 2 |
| NC_019061.1 | Escherichia coli | pPWD4_103 | 2.0E-38 | 97.03 | 101 | 9 |
| NC_012886.1 | Escherichia coli | pRAx | 4.0E-43 | 100 | 101 | 1 |
| NC_019088.1 | Escherichia coli | pRPEC180_47 | 1.0E-41 | 99.01 | 101 | 1 |
| NC_009602.1 | Escherichia coli | pSFO157 | 9.0E-43 | 100 | 101 | 2 |
| NC_019013.1 | Escherichia coli | pSYM1 | 4.0E-43 | 100 | 101 | 2 |
| NC_011514.1 | Escherichia coli | pTN38148 | 8.0E-44 | 100 | 101 | 2 |
| NC_010409.1 | Escherichia coli | pVM01 | 6.0E-40 | 98.02 | 101 | 8 |
| NC_022371.1 | Escherichia coli PMV-1 | pHUSEC411like | 4.0E-40 | 98.02 | 101 | 6 |
| NC_011747.1 | Escherichia coli S88 | pECOS88 | 5.0E-40 | 98.02 | 101 | 3 |
| NC_011419.1 | Escherichia coli SE11 | pSE11-1 | 7.0E-43 | 100 | 101 | 1 |
| NC_011413.1 | Escherichia coli SE11 | pSE11-2 | 7.0E-43 | 100 | 101 | 1 |
| NC_013655.1 | Escherichia coli SE15 | pECSF1 | 9.0E-43 | 100 | 101 | 5 |
| NC_010488.1 | Escherichia coli SMS-3-5 | pSMS35_130 | 3.0E-37 | 96.04 | 101 | 3 |
| NC_010486.1 | Escherichia coli SMS-3-5 | pSMS35_4 | 3.0E-44 | 100 | 101 | 1 |
| NC_023277.1 | Escherichia coli strain 63743 | pEQ2 | 1.0E-38 | 98.02 | 94 | 6 |
| NC_023329.1 | Escherichia coli strain B3804 | pIFM3804 | 3.0E-41 | 99.01 | 101 | 2 |
| NC_019990.1 | Escherichia coli strain BB1093 | pB1024 | 5.0E-44 | 100 | 101 | 3 |
| NC_023315.1 | Escherichia coli strain EQ011 | pEQ011 | 6.0E-43 | 100 | 101 | 2 |
| NC_022885.1 | Escherichia coli strain LK-NARMP | pKPC-LKEc | 8.0E-38 | 99.01 | 92 | 30 |
| NC_022377.1 | Escherichia coli strain SCEC2 | pSCEC2 | 1.0E-42 | 100 | 101 | 2 |
| NC_017630.1 | Escherichia coli UM146 | pUM146 | 2.0E-38 | 97.03 | 101 | 4 |
| NC_017640.1 | Escherichia coli UMNK88 | pUMNK88_Ent | 7.0E-36 | 95.05 | 97 | 3 |
| NC_017643.1 | Escherichia coli UMNK88 | pUMNK88_Hly | 1.0E-37 | 96.04 | 101 | 4 |
| NC_017639.1 | Escherichia coli UMNK88 | pUMNK88_K88 | 6.0E-43 | 100 | 101 | 2 |
| NC_012944.1 | Escherichia coli Vir68 | pVir68 | 5.0E-40 | 98.02 | 101 | 8 |
| NC_009716.1 | Escherichia sp. Sflu5 cryptic | pAK51 | 6.0E-37 | 95.05 | 101 | 4 |
| NC_012782.1 | Eubacterium eligens ATCC 27750 | unnamed | 2.0E-35 | 95 | 93 | 162 |
| NC_012780.1 | Eubacterium eligens ATCC 27750 | unnamed | 4.0E-30 | 95 | 90 | 6543 |
| NC_023287.1 | Exiguobacterium sp. S3-2 | pMC1 | 2.0E-35 | 95 | 99 | 26 |
| NC_010371.1 | Finegoldia magna ATCC 29328 | pFMC | 2.0E-35 | 95.05 | 100 | 3 |
| NC_009966.1 | Fluoribacter dumoffii Tex-KL | pLD-TEX-KL | 5.0E-43 | 100 | 101 | 5 |
| NC_019361.1 | Haemophilus parasuis | pFZ51 | 1.0E-43 | 100 | 101 | 1 |
| NC_012661.1 | Haemophilus parasuis | pHN61 | 2.0E-36 | 98.89 | 90 | 31 |
| NC_021186.1 | Haemophilus parasuis strain QY431 | pQY431 | 6.0E-44 | 100 | 101 | 1 |
| NC_005910.1 | Hafnia alvei | pAlvA | 4.0E-44 | 100 | 101 | 2 |
| NC_008154.1 | Human gut | pTRACA10 | 6.0E-37 | 95.05 | 101 | 1 |
| NC_008153.1 | Human gut | pTRACA17 | 1.0E-41 | 98.02 | 101 | 1 |
| NC_014633.1 | Ilyobacter polytropus DSM 2926 | pILYOP01 | 3.0E-33 | 95 | 91 | 35 |
| NC_014621.1 | Ketogulonicigenium vulgare Y25 | pYP1 | 5.0E-31 | 95 | 92 | 55 |
| NC_017386.1 | Ketogulonigenium vulgarum WSH-001 | 1 | 3.0E-39 | 98 | 100 | 1 |
| NC_021501.1 | Klebsiella oxytoca E718 | pKOX_NDM1 | 4.0E-40 | 98.02 | 101 | 6 |
| NC_018107.1 | Klebsiella oxytoca E718 | pKOX_R1 | 6.0E-38 | 98.96 | 96 | 4 |
| NC_019286.1 | Klebsiella oxytoca | pINCan01 | 1.0E-38 | 96.04 | 101 | 5 |
| NC_011282.1 | Klebsiella pneumoniae 342 | pKP187 | 3.0E-38 | 97.03 | 101 | 2 |
| NC_005249.1 | Klebsiella pneumoniae CG43 | pLVPK | 8.0E-40 | 98.02 | 101 | 7 |
| NC_022078.1 | Klebsiella pneumoniae JM45 | p1 | 1.0E-39 | 98.02 | 101 | 1 |
| NC_022083.1 | Klebsiella pneumoniae JM45 | p2 | 9.0E-38 | 100 | 90 | 1 |
| NC_017541.1 | Klebsiella pneumoniae KCTC 2242 | pKCTC2242 | 7.0E-34 | 96.04 | 90 | 36 |
| NC_006625.1 | Klebsiella pneumoniae NTUH-K2044 | pK2044 | 2.0E-35 | 95.05 | 101 | 3 |
| NC_011385.1 | Klebsiella pneumoniae | 12 | 3.0E-40 | 98.02 | 101 | 1 |
| NC_011382.1 | Klebsiella pneumoniae | 15S | 2.0E-43 | 100 | 101 | 2 |
| NC_011383.1 | Klebsiella pneumoniae | 9 | 5.0E-43 | 100 | 101 | 1 |
| NC_021666.1 | Klebsiella pneumoniae | ColE-LS6 | 1.0E-43 | 100 | 101 | 4 |
| NC_021667.1 | Klebsiella pneumoniae | IncA/C-LS6 | 9.0E-32 | 95 | 91 | 29 |
| NC_011511.1 | Klebsiella pneumoniae | p169 | 6.0E-40 | 97.03 | 100 | 10 |
| NC_011512.1 | Klebsiella pneumoniae | p9701 | 4.0E-44 | 100 | 101 | 2 |
| NC_022520.1 | Klebsiella pneumoniae | pBK15692 | 6.0E-43 | 100 | 101 | 8 |
| NC_015154.1 | Klebsiella pneumoniae | pc15-k | 2.0E-37 | 96.04 | 96 | 4 |
| NC_019166.1 | Klebsiella pneumoniae | pIMP-HZ1 | 1.0E-37 | 96.04 | 101 | 1 |
| NC_020087.1 | Klebsiella pneumoniae | pK1HV | 2.0E-33 | 95.05 | 91 | 42 |
| NC_010886.1 | Klebsiella pneumoniae | pK245 | 4.0E-40 | 98.02 | 101 | 8 |
| NC_010870.1 | Klebsiella pneumoniae | pK29 | 2.0E-35 | 95.05 | 101 | 12 |
| NC_019389.1 | Klebsiella pneumoniae | pKDO1 | 5.0E-40 | 98.02 | 101 | 1 |
| NC_013951.1 | Klebsiella pneumoniae | pKF3-140 | 3.0E-37 | 96.04 | 98 | 14 |
| NC_013542.1 | Klebsiella pneumoniae | pKF3-70 | 4.0E-38 | 98.02 | 92 | 12 |
| NC_002610.1 | Klebsiella pneumoniae | pKlebB-k17/80 | 1.0E-38 | 97.03 | 101 | 2 |
| NC_014312.1 | Klebsiella pneumoniae | pKP048 | 6.0E-40 | 98.02 | 101 | 6 |
| NC_021576.1 | Klebsiella pneumoniae | pKP1780 | 8.0E-39 | 97.03 | 96 | 3 |
| NC_021357.1 | Klebsiella pneumoniae | pKP53IL | 9.0E-44 | 100 | 101 | 2 |
| NC_021363.1 | Klebsiella pneumoniae | pKP53IL | 9.0E-42 | 100 | 97 | 1 |
| NC_021364.1 | Klebsiella pneumoniae | pKP53IL | 2.0E-42 | 99.01 | 101 | 1 |
| NC_020893.1 | Klebsiella pneumoniae | pKP53IL | 6.0E-43 | 100 | 101 | 3 |
| NC_019390.1 | Klebsiella pneumoniae | pKPN_CZ | 6.0E-35 | 95 | 91 | 105 |
| NC_019165.1 | Klebsiella pneumoniae | pKPN101-IT | 8.0E-43 | 100 | 101 | 2 |
| NC_021502.1 | Klebsiella pneumoniae | pKPoxa-48N2 | 6.0E-40 | 98.02 | 101 | 4 |
| NC_014016.1 | Klebsiella pneumoniae | pKpQIL | 2.0E-37 | 96.97 | 99 | 1 |
| NC_019155.1 | Klebsiella pneumoniae | pKpQIL-IT | 4.0E-41 | 99.01 | 101 | 4 |
| NC_021655.1 | Klebsiella pneumoniae | pKpQIL-LS6 | 3.0E-40 | 98.02 | 101 | 1 |
| NC_021656.1 | Klebsiella pneumoniae | pKpQIL-SC29 | 4.0E-43 | 100 | 101 | 1 |
| NC_023314.1 | Klebsiella pneumoniae | pKPS30 | 2.0E-40 | 98.02 | 101 | 1 |
| NC_010726.1 | Klebsiella pneumoniae | pMET-1 | 8.0E-38 | 96.04 | 101 | 7 |
| NC_019158.1 | Klebsiella pneumoniae | pNDM10469 | 3.0E-37 | 96.04 | 101 | 2 |
| NC_019153.1 | Klebsiella pneumoniae | pNDM-KN | 5.0E-41 | 99.01 | 101 | 1 |
| NC_016980.1 | Klebsiella pneumoniae | pNDM-MAR | 2.0E-42 | 100 | 101 | 4 |
| NC_019152.1 | Klebsiella pneumoniae | pSLMT | 8.0E-41 | 98.02 | 101 | 1 |
| NC_014478.1 | Klebsiella pneumoniae | unnamed | 7.0E-37 | 95.05 | 98 | 41 |
| NC_019988.1 | Klebsiella pneumoniae strain BB1088 | pB1019 | 2.0E-41 | 98.02 | 101 | 1 |
| NC_019888.1 | Klebsiella pneumoniae strain BK31551 | pBK31551 | 6.0E-37 | 100 | 90 | 5 |
| NC_020132.1 | Klebsiella pneumoniae strain BK32179 | pBK32179 | 1.0E-35 | 95.05 | 101 | 16 |
| NC_021078.1 | Klebsiella pneumoniae strain Kp002 | pJEG011 | 1.0E-31 | 95 | 90 | 57 |
| NC_023330.1 | Klebsiella pneumoniae strain KPS77 | pKPS77 | 1.0E-41 | 99.01 | 101 | 6 |
| NC_022740.1 | Klebsiella pneumoniae strain MGR-K194 | pNDM_MGR194 | 3.0E-43 | 100 | 101 | 1 |
| NC_022609.1 | Klebsiella pneumoniae strain N11-0042 | pKp11-42 | 3.0E-37 | 96.04 | 101 | 19 |
| NC_023334.1 | Klebsiella pneumoniae strain ST15 | pKP02022 | 1.0E-42 | 100 | 101 | 2 |
| NC_023333.1 | Klebsiella pneumoniae strain ST23 | pKP007 | 9.0E-34 | 95.05 | 90 | 30 |
| NC_023332.1 | Klebsiella pneumoniae strain ST48 | pKP09085 | 2.0E-35 | 95.05 | 90 | 32 |
| NC_016838.1 | Klebsiella pneumoniae subsp. pneumoniae HS11286 | pKPHS1 | 9.0E-43 | 100 | 101 | 3 |
| NC_016839.1 | Klebsiella pneumoniae subsp. pneumoniae HS11286 | pKPHS3 | 9.0E-36 | 95.05 | 101 | 3 |
| NC_016840.1 | Klebsiella pneumoniae subsp. pneumoniae HS11286 | pKPHS4 | 3.0E-44 | 100 | 101 | 1 |
| NC_021198.1 | Klebsiella pneumoniae subsp. pneumoniae KPX | pKPX-1 DNA | 9.0E-40 | 98.02 | 99 | 3 |
| NC_009649.1 | Klebsiella pneumoniae subsp. pneumoniae MGH 78578 | pKPN3 | 1.0E-35 | 95.05 | 90 | 27 |
| NC_009650.1 | Klebsiella pneumoniae subsp. pneumoniae MGH 78578 | pKPN4 | 8.0E-43 | 100 | 101 | 1 |
| NC_009651.1 | Klebsiella pneumoniae subsp. pneumoniae MGH 78578 | pKPN5 | 6.0E-43 | 100 | 101 | 2 |
| NC_003789.1 | Klebsiella sp. KCL-2 | pMGD2 | 3.0E-44 | 100 | 101 | 1 |
| NC_015213.1 | Lactobacillus acidophilus 30SC | pRKC30SC1 | 3.0E-41 | 98.02 | 101 | 12 |
| NC_015319.1 | Lactobacillus amylovorus GRL 1112 | 1 | 1.0E-41 | 99.01 | 101 | 2 |
| NC_017471.1 | Lactobacillus amylovorus GRL1118 | 1 | 2.0E-42 | 99.01 | 101 | 2 |
| NC_017472.1 | Lactobacillus amylovorus GRL1118 | 2 | 1.0E-33 | 95 | 92 | 157 |
| NC_008499.1 | Lactobacillus brevis ATCC 367 | 2 | 1.0E-41 | 99.01 | 101 | 1 |
| NC_020820.1 | Lactobacillus brevis KB290 | pKB290-1 DNA | 3.0E-43 | 100 | 101 | 2 |
| NC_020821.1 | Lactobacillus brevis KB290 | pKB290-2 DNA | 6.0E-39 | 97.03 | 101 | 4 |
| NC_020828.1 | Lactobacillus brevis KB290 | pKB290-8 DNA | 3.0E-42 | 99.01 | 101 | 1 |
| NC_018611.1 | Lactobacillus buchneri CD034 | pCD034-3 | 2.0E-40 | 98.02 | 101 | 2 |
| NC_015429.1 | Lactobacillus buchneri NRRL B-30929 | pLBUC02 | 3.0E-39 | 97.03 | 101 | 3 |
| NC_015421.1 | Lactobacillus buchneri NRRL B-30929 | pLBUC03 | 2.0E-38 | 100 | 91 | 3 |
| NC_008502.1 | Lactobacillus casei ATCC 334 | 1 | 2.0E-43 | 100 | 101 | 2 |
| NC_017476.1 | Lactobacillus casei BD-II | pBD-II | 2.0E-40 | 100 | 96 | 1 |
| NC_021722.1 | Lactobacillus casei LOCK919 | pLOCK919 | 3.0E-36 | 95.92 | 98 | 3 |
| NC_011352.1 | Lactobacillus casei str. Zhang | plca36 | 1.0E-35 | 95 | 100 | 6 |
| NC_020057.1 | Lactobacillus casei W56 | pW56 | 4.0E-43 | 100 | 101 | 2 |
| NC_003320.2 | Lactobacillus curvatus strain CRL705 | pRC18 | 5.0E-37 | 98.02 | 92 | 2 |
| NC_004947.1 | Lactobacillus fermentum | pKC5b | 1.0E-42 | 99.01 | 101 | 2 |
| NC_011839.1 | Lactobacillus gasseri | pLgLA39 | 6.0E-39 | 97.03 | 98 | 6 |
| NC_002102.1 | Lactobacillus helveticus DSM 20075 | pLH1 | 1.0E-43 | 100 | 101 | 1 |
| NC_014386.1 | Lactobacillus helveticus R0052 | pIR52-1 | 5.0E-44 | 100 | 101 | 1 |
| NC_013505.1 | Lactobacillus johnsonii FI9785 | p9785L | 4.0E-39 | 97.03 | 101 | 1 |
| NC_015598.1 | Lactobacillus kefiranofaciens ZW3 | pWW1 | 4.0E-37 | 96.04 | 101 | 5 |
| NC_015603.1 | Lactobacillus kefiranofaciens ZW3 | pWW2 | 1.0E-42 | 100 | 100 | 4 |
| NC_022114.1 | Lactobacillus paracasei subsp. paracasei 8700:2 | 1 | 8.0E-42 | 99.01 | 101 | 1 |
| NC_022123.1 | Lactobacillus paracasei subsp. paracasei 8700:2 | 2 | 5.0E-43 | 100 | 101 | 2 |
| NC_021515.1 | Lactobacillus plantarum 16 | Lp16A | 6.0E-44 | 100 | 101 | 1 |
| NC_021525.1 | Lactobacillus plantarum 16 | Lp16B | 7.0E-44 | 100 | 101 | 2 |
| NC_021526.1 | Lactobacillus plantarum 16 | Lp16D | 6.0E-39 | 99.01 | 93 | 2 |
| NC_021517.1 | Lactobacillus plantarum 16 | Lp16E | 3.0E-43 | 100 | 101 | 2 |
| NC_021518.1 | Lactobacillus plantarum 16 | Lp16F | 1.0E-37 | 96.04 | 101 | 2 |
| NC_021528.1 | Lactobacillus plantarum 16 | Lp16I | 1.0E-43 | 100 | 101 | 3 |
| NC_004944.1 | Lactobacillus plantarum 5057 | pMD5057 | 5.0E-40 | 97.98 | 99 | 3 |
| NC_006278.1 | Lactobacillus plantarum NC7 | p256 | 6.0E-44 | 100 | 101 | 3 |
| NC_012628.1 | Lactobacillus plantarum | pLFE1 | 1.0E-42 | 99.01 | 101 | 18 |
| NC_021233.1 | Lactobacillus plantarum subsp. plantarum P-8 | LBPp1 | 4.0E-36 | 95.05 | 97 | 7 |
| NC_021227.1 | Lactobacillus plantarum subsp. plantarum P-8 | LBPp5 | 1.0E-43 | 100 | 101 | 7 |
| NC_014558.2 | Lactobacillus plantarum subsp. plantarum ST-III | pST-III | 3.0E-38 | 97 | 100 | 7 |
| NC_006377.1 | Lactobacillus plantarum WCFS1 | pWCFS103 | 7.0E-38 | 96.04 | 101 | 1 |
| NC_021903.1 | Lactobacillus plantarum ZJ316 | pLP-ZJ101 | 1.0E-42 | 100 | 99 | 4 |
| NC_021912.1 | Lactobacillus plantarum ZJ316 | pLP-ZJ103 | 8.0E-38 | 96.04 | 101 | 7 |
| NC_021503.1 | Lactobacillus reuteri I5007 | pLRI01 | 2.0E-41 | 99.01 | 101 | 4 |
| NC_021495.1 | Lactobacillus reuteri I5007 | pLRI02 | 6.0E-41 | 98.02 | 101 | 2 |
| NC_021496.1 | Lactobacillus reuteri I5007 | pLRI03 | 1.0E-41 | 99.01 | 101 | 2 |
| NC_021504.1 | Lactobacillus reuteri I5007 | pLRI04 | 1.0E-36 | 95.05 | 90 | 46 |
| NC_021497.1 | Lactobacillus reuteri I5007 | pLRI05 | 3.0E-38 | 96.04 | 101 | 1 |
| NC_003528.1 | Lactobacillus reuteri | pTE44 | 3.0E-38 | 100 | 90 | 4 |
| NC_015701.1 | Lactobacillus reuteri SD2112 | pLR584 | 6.0E-42 | 99.01 | 101 | 2 |
| NC_010603.1 | Lactobacillus reuteri strain ATCC 55730 | pLR581 | 5.0E-35 | 96.04 | 90 | 90 |
| NC_011225.1 | Lactobacillus rhamnosus HN001 | pLR002 | 2.0E-43 | 100 | 101 | 1 |
| NC_013200.1 | Lactobacillus rhamnosus Lc 705 | pLC1 | 3.0E-40 | 98.02 | 101 | 4 |
| NC_017479.1 | Lactobacillus salivarius CECT 5713 | pHN1 | 3.0E-43 | 100 | 101 | 5 |
| NC_017480.1 | Lactobacillus salivarius CECT 5713 | pHN2 | 2.0E-43 | 100 | 101 | 1 |
| NC_017499.1 | Lactobacillus salivarius CECT 5713 | pHN3 | 4.0E-38 | 97.03 | 99 | 7 |
| NC_007930.1 | Lactobacillus salivarius UCC118 | pMP118 | 3.0E-39 | 100 | 95 | 1 |
| NC_006530.1 | Lactobacillus salivarius UCC118 | pSF118-44 | 2.0E-40 | 98.02 | 101 | 8 |
| NC_015979.1 | Lactobacillus sanfranciscensis TMW 1.1304 | pLS1 | 2.0E-41 | 99.01 | 101 | 6 |
| NC_015980.1 | Lactobacillus sanfranciscensis TMW 1.1304 | pLS2 | 1.0E-43 | 100 | 101 | 1 |
| NC_016970.1 | Lactococcus garvieae 21881 | pGL3 | 5.0E-41 | 98.02 | 101 | 8 |
| NC_010540.1 | Lactococcus garvieae | pKL0018 DNA | 2.0E-43 | 100 | 101 | 2 |
| NC_007191.1 | Lactococcus lactis cremoris 712 | pAG6 | 3.0E-42 | 99.01 | 101 | 5 |
| NC_004847.1 | Lactococcus lactis cremoris HP | pHP003 | 4.0E-42 | 99.01 | 101 | 2 |
| NC_002137.1 | Lactococcus lactis cremoris NIZO B40 | pNZ4000 | 3.0E-37 | 96.04 | 90 | 16 |
| NC_003101.1 | Lactococcus lactis CRL1127 | pCRL1127 | 3.0E-42 | 99.01 | 101 | 7 |
| NC_002798.1 | Lactococcus lactis DCH-4 | pSRQ700 | 3.0E-42 | 99.01 | 101 | 1 |
| NC_001949.1 | Lactococcus lactis DPC3147 | pMRC01 | 2.0E-41 | 99.01 | 101 | 10 |
| NC_000906.2 | Lactococcus lactis IL964 | pIL105 | 1.0E-39 | 97.03 | 101 | 2 |
| NC_004966.1 | Lactococcus lactis lactis bv. diacetylactis DPC220 | pAH82 | 6.0E-36 | 95.1 | 101 | 7 |
| NC_004955.1 | Lactococcus lactis lactis IPLA 972 | pBL1 | 4.0E-41 | 98.02 | 101 | 2 |
| NC_002502.1 | Lactococcus lactis lactis UC317 | pCI305 | 7.0E-44 | 100 | 101 | 1 |
| NC_004981.2 | Lactococcus lactis ML8 | pCL2.1 | 2.0E-44 | 100 | 101 | 1 |
| NC_009435.1 | Lactococcus lactis NCDO 1867 | pGdh442 | 6.0E-36 | 95.05 | 91 | 29 |
| NC_002150.1 | Lactococcus lactis | pAH33 | 3.0E-41 | 98.02 | 101 | 2 |
| NC_011610.1 | Lactococcus lactis | pKL001 | 5.0E-44 | 100 | 101 | 1 |
| NC_010901.1 | Lactococcus lactis | pNP40 | 2.0E-40 | 98.02 | 101 | 4 |
| NC_017500.1 | Lactococcus lactis | pSK11P | 6.0E-43 | 100 | 101 | 21 |
| NC_004980.1 | Lactococcus lactis | pWC1 | 1.0E-40 | 98.02 | 94 | 2 |
| NC_017497.1 | Lactococcus lactis subsp. cremoris A76 | pQA504 | 8.0E-39 | 96.04 | 100 | 3 |
| NC_017493.1 | Lactococcus lactis subsp. cremoris A76 | pQA549 | 2.0E-41 | 99.01 | 101 | 1 |
| NC_017496.1 | Lactococcus lactis subsp. cremoris A76 | pQA554 | 4.0E-37 | 96.04 | 90 | 29 |
| NC_019349.1 | Lactococcus lactis subsp. cremoris | pAF12 | 5.0E-41 | 100 | 96 | 5 |
| NC_019350.1 | Lactococcus lactis subsp. cremoris | pAF14 | 6.0E-41 | 98.02 | 101 | 10 |
| NC_017494.1 | Lactococcus lactis subsp. cremoris | pAW153 | 6.0E-44 | 100 | 101 | 1 |
| NC_019377.1 | Lactococcus lactis subsp. cremoris | pLP712 | 9.0E-39 | 98.02 | 93 | 10 |
| NC_008504.1 | Lactococcus lactis subsp. cremoris SK11 | 2 | 6.0E-39 | 97.94 | 97 | 6 |
| NC_008505.1 | Lactococcus lactis subsp. cremoris SK11 | 3 | 2.0E-35 | 95 | 90 | 21 |
| NC_008506.1 | Lactococcus lactis subsp. cremoris SK11 | 4 | 2.0E-40 | 98.02 | 101 | 24 |
| NC_008507.1 | Lactococcus lactis subsp. cremoris SK11 | 5 | 1.0E-43 | 100 | 101 | 1 |
| NC_019438.1 | Lactococcus lactis subsp. cremoris UC509.9 | pCIS1 | 1.0E-42 | 99.01 | 101 | 3 |
| NC_019434.1 | Lactococcus lactis subsp. cremoris UC509.9 | pCIS2 | 2.0E-36 | 95.96 | 99 | 4 |
| NC_019437.1 | Lactococcus lactis subsp. cremoris UC509.9 | pCIS4 | 3.0E-40 | 98.02 | 101 | 2 |
| NC_019432.1 | Lactococcus lactis subsp. cremoris UC509.9 | pCIS5 | 9.0E-44 | 100 | 101 | 2 |
| NC_019436.1 | Lactococcus lactis subsp. cremoris UC509.9 | pCIS6 | 3.0E-36 | 95.05 | 101 | 7 |
| NC_019431.1 | Lactococcus lactis subsp. cremoris UC509.9 | pCIS7 | 9.0E-39 | 97.03 | 101 | 8 |
| NC_019430.1 | Lactococcus lactis subsp. cremoris UC509.9 | pCIS8 | 8.0E-35 | 95.83 | 94 | 39 |
| NC_009137.1 | Lactococcus lactis subsp. lactis bv. diacetylactis | pDBORO | 1.0E-36 | 95.05 | 101 | 4 |
| NC_015900.1 | Lactococcus lactis subsp. lactis bv. diacetylactis | pVF18 | 4.0E-38 | 96.04 | 97 | 14 |
| NC_015912.1 | Lactococcus lactis subsp. lactis bv. diacetylactis | pVF21 | 2.0E-42 | 100 | 99 | 1 |
| NC_015901.1 | Lactococcus lactis subsp. lactis bv. diacetylactis | pVF22 | 1.0E-38 | 99.01 | 92 | 2 |
| NC_015902.1 | Lactococcus lactis subsp. lactis bv. diacetylactis | pVF50 | 2.0E-35 | 96.04 | 90 | 11 |
| NC_017483.1 | Lactococcus lactis subsp. lactis CV56 | pCV56A | 5.0E-35 | 97.8 | 91 | 9 |
| NC_017487.1 | Lactococcus lactis subsp. lactis CV56 | pCV56B | 7.0E-38 | 98.02 | 94 | 11 |
| NC_017484.1 | Lactococcus lactis subsp. lactis CV56 | pCV56C | 8.0E-37 | 96.04 | 92 | 8 |
| NC_009751.1 | Lactococcus lactis subsp. lactis K214 | pK214 | 1.0E-40 | 98.02 | 101 | 13 |
| NC_013657.1 | Lactococcus lactis subsp. lactis KF147 | pKF147A | 3.0E-36 | 95.05 | 101 | 9 |
| NC_015861.1 | Lactococcus lactis subsp. lactis | pIL3 | 8.0E-41 | 99.01 | 101 | 11 |
| NC_015862.1 | Lactococcus lactis subsp. lactis | pIL4 | 8.0E-39 | 97.03 | 101 | 15 |
| NC_015863.1 | Lactococcus lactis subsp. lactis | pIL5 | 3.0E-40 | 98.04 | 101 | 11 |
| NC_019308.1 | Lactococcus lactis subsp. lactis | pIL6 | 6.0E-38 | 96.04 | 101 | 8 |
| NC_015864.1 | Lactococcus lactis subsp. lactis | pIL7 | 1.0E-34 | 95.79 | 95 | 6 |
| NC_016042.1 | Lactococcus lactis subsp. lactis | pKP1 | 3.0E-39 | 97.03 | 101 | 9 |
| NC_008594.1 | Lactococcus lactis subsp. lactis | pL2 | 2.0E-42 | 99.01 | 101 | 2 |
| NC_002748.1 | Lactococcus lactis subsp. lactis strain MJC15 | pCD4 | 5.0E-37 | 95.05 | 101 | 4 |
| NC_004960.1 | Lactococcus lactis W-1 | pSRQ800 | 3.0E-41 | 98.99 | 99 | 2 |
| NC_004959.1 | Lactococcus lactis W-37 | pSRQ900 | 8.0E-44 | 100 | 101 | 1 |
| NC_010370.1 | Laribacter hongkongensis | pHLHK22 | 2.0E-40 | 98 | 100 | 2 |
| NC_006628.1 | Laribacter hongkongensis | pHLHK8 | 3.0E-41 | 98.02 | 101 | 2 |
| NC_010846.1 | Leptospira biflexa serovar Patoc strain 'Patoc 1 (Ames)' | p74 | 3.0E-39 | 97.98 | 99 | 1 |
| NC_018675.1 | Leuconostoc carnosum JB16 | pKLC3 | 2.0E-38 | 97 | 100 | 9 |
| NC_018699.1 | Leuconostoc carnosum JB16 | pKLC4 | 1.0E-34 | 97.78 | 90 | 3 |
| NC_010470.1 | Leuconostoc citreum KM20 | pLCK1 | 1.0E-35 | 95 | 100 | 10 |
| NC_010466.1 | Leuconostoc citreum KM20 | pLCK2 | 1.0E-40 | 98.02 | 101 | 6 |
| NC_010469.1 | Leuconostoc citreum KM20 | pLCK4 | 6.0E-40 | 97.98 | 99 | 4 |
| NC_004528.1 | Leuconostoc citreum pLC22R |  | 4.0E-41 | 99.01 | 101 | 1 |
| NC_014131.1 | Leuconostoc kimchii IMSNU 11154 | LkipL4701 | 4.0E-38 | 96.04 | 100 | 4 |
| NC_014132.1 | Leuconostoc kimchii IMSNU 11154 | LkipL4704 | 6.0E-37 | 96.91 | 97 | 2 |
| NC_014133.1 | Leuconostoc kimchii IMSNU 11154 | LkipL4719 | 2.0E-36 | 95.92 | 98 | 15 |
| NC_014134.1 | Leuconostoc kimchii IMSNU 11154 | LkipL4726 | 6.0E-38 | 96.04 | 100 | 6 |
| NC_008496.1 | Leuconostoc mesenteroides subsp. mesenteroides ATCC 8293 | pLEUM1 | 1.0E-41 | 99.01 | 101 | 2 |
| NC_016827.1 | Leuconostoc mesenteroides subsp. mesenteroides J18 | pKLE01 | 6.0E-33 | 95.65 | 92 | 2 |
| NC_016820.1 | Leuconostoc mesenteroides subsp. mesenteroides J18 | pKLE02 | 6.0E-39 | 97.03 | 101 | 2 |
| NC_016828.1 | Leuconostoc mesenteroides subsp. mesenteroides J18 | pKLE04 | 3.0E-39 | 97.03 | 101 | 1 |
| NC_003383.1 | Listeria innocua Clip11262 | pLI100 | 1.0E-38 | 97.03 | 101 | 2 |
| NC_014495.1 | Listeria monocytogenes SLCC2755 | pLM1-2bUG1 | 2.0E-40 | 98.02 | 101 | 6 |
| NC_022051.1 | Listeria monocytogenes strain J1926 |  | 4.0E-43 | 100 | 101 | 2 |
| NC_014255.1 | Listeria monocytogenes strain Lm1 | pLM33 | 2.0E-43 | 100 | 101 | 1 |
| NC_022045.1 | Listeria monocytogenes strain N1-011A |  | 5.0E-41 | 99.01 | 101 | 15 |
| NC_011996.1 | Macrococcus caseolyticus JCSC5402 | pMCCL2 | 1.0E-38 | 97.96 | 97 | 32 |
| NC_014213.1 | Meiothermus silvanus DSM 9946 | pMESIL01 | 7.0E-37 | 96.04 | 101 | 4 |
| NC_002679.1 | Mesorhizobium loti MAFF303099 | pMLa | 2.0E-38 | 97.98 | 99 | 1 |
| NC_002682.1 | Mesorhizobium loti MAFF303099 | pMLb | 2.0E-35 | 95.05 | 101 | 1 |
| NC_008242.1 | Mesorhizobium sp. BNC1 | 1 | 1.0E-33 | 95.79 | 95 | 30 |
| NC_008243.1 | Mesorhizobium sp. BNC1 | 2 | 3.0E-37 | 96.04 | 101 | 2 |
| NC_019972.1 | Methanomethylovorans hollandica DSM 15978 | pMETHO01 | 9.0E-41 | 99.01 | 101 | 12 |
| NC_008826.1 | Methylibium petroleiphilum PM1 | RPME01 | 5.0E-35 | 95.05 | 101 | 1 |
| NC_012811.1 | Methylobacterium extorquens AM1 mega |  | 1.0E-32 | 95.05 | 92 | 61 |
| NC_011758.1 | Methylobacterium extorquens CM4 | pMCHL01 | 3.0E-42 | 100 | 101 | 2 |
| NC_011892.1 | Methylobacterium nodulans ORS 2060 | pMNOD01 | 4.0E-35 | 95.05 | 101 | 1 |
| NC_010510.1 | Methylobacterium radiotolerans JCM 2831 | pMRAD01 | 9.0E-32 | 95 | 90 | 44 |
| NC_004954.1 | Micrococcus sp. 28 | pSD10 | 2.0E-34 | 95 | 95 | 39 |
| NC_022599.1 | Micrococcus sp. V7 | pLMV7 | 3.0E-34 | 95.05 | 95 | 23 |
| NC_019760.1 | Microcoleus sp. PCC 7113 | pMIC7113.02 | 1.0E-32 | 95 | 90 | 44 |
| NC_016036.1 | Morganella morganii strain M203 | R485 | 5.0E-43 | 100 | 101 | 1 |
| NC_017908.2 | Mycobacterium abscessus subsp. bolletii F1725 | BRA100 | 1.0E-37 | 98.02 | 91 | 88 |
| NC_018023.1 | Mycobacterium chubuense NBB4 | pMYCCH.02 | 5.0E-40 | 98.02 | 101 | 1 |
| NC_014811.1 | Mycobacterium gilvum Spyr1 | pMSPYR101 | 1.0E-37 | 97 | 100 | 2 |
| NC_008703.1 | Mycobacterium sp. KMS | pMKMS01 | 9.0E-41 | 99.01 | 101 | 1 |
| NC_010189.1 | Naegleria gruberi extrachromosomal rDNA | DNA | 1.0E-36 | 95.05 | 101 | 1 |
| NC_014105.1 | Neisseria gonorrhoeae | pEP5289 | 3.0E-43 | 100 | 101 | 1 |
| NC_017510.1 | Neisseria gonorrhoeae TCDC-NG08107 | pNGTCDC08107 | 1.0E-41 | 99.01 | 101 | 1 |
| NC_007959.1 | Nitrobacter hamburgensis X14 | 1 | 6.0E-37 | 96.04 | 101 | 2 |
| NC_008341.1 | Nitrosomonas eutropha C91 | 1 | 1.0E-38 | 97.03 | 101 | 9 |
| NC_008342.1 | Nitrosomonas eutropha C91 | 2 | 5.0E-36 | 95.05 | 101 | 1 |
| NC_015223.1 | Nitrosomonas sp. AL212 | pNAL21201 | 4.0E-39 | 98.02 | 101 | 2 |
| NC_015221.1 | Nitrosomonas sp. AL212 | pNAL21202 | 5.0E-43 | 100 | 101 | 2 |
| NC_006363.1 | Nocardia farcinica IFM 10152 | pNF2 | 4.0E-33 | 95.7 | 93 | 2 |
| NC_008697.1 | Nocardioides sp. JS614 | pNOCA01 | 3.0E-35 | 95.05 | 99 | 2 |
| NC_002033.1 | Novosphingobium aromaticivorans | pNL1 | 5.0E-35 | 95 | 100 | 3 |
| NC_015579.1 | Novosphingobium sp. PP1Y | Lpl | 4.0E-37 | 96.04 | 101 | 1 |
| NC_015583.1 | Novosphingobium sp. PP1Y | Mpl | 9.0E-35 | 95.05 | 101 | 2 |
| NC_009669.1 | Ochrobactrum anthropi ATCC 49188 | pOANT01 | 9.0E-32 | 95.05 | 91 | 114 |
| NC_009670.1 | Ochrobactrum anthropi ATCC 49188 | pOANT02 | 7.0E-43 | 100 | 101 | 10 |
| NC_009671.1 | Ochrobactrum anthropi ATCC 49188 | pOANT03 | 2.0E-38 | 97.03 | 101 | 6 |
| NC_009672.1 | Ochrobactrum anthropi ATCC 49188 | pOANT04 | 8.0E-40 | 98 | 100 | 1 |
| NC_020909.1 | Octadecabacter arcticus 238 | pOA238_118 | 2.0E-37 | 96.04 | 101 | 1 |
| NC_019553.1 | Oenococcus oeni | pOENI-1 | 7.0E-35 | 96.77 | 93 | 2 |
| NC_019554.1 | Oenococcus oeni | pOENI-1v2 | 7.0E-42 | 99.01 | 101 | 2 |
| NC_017539.1 | Oligotropha carboxidovorans OM4 | pOC167B | 8.0E-39 | 97.98 | 99 | 3 |
| NC_015689.1 | Oligotropha carboxidovorans OM5 | pHCG3 | 5.0E-40 | 98.02 | 101 | 2 |
| NC_011414.1 | Ornithobacterium rhinotracheale | pOR1 | 5.0E-35 | 95.05 | 92 | 4 |
| NC_022042.1 | Paracoccus aminophilus JCM 7686 | pAMI1 | 6.0E-32 | 95 | 91 | 88 |
| NC_022049.1 | Paracoccus aminophilus JCM 7686 | pAMI4 | 3.0E-36 | 96 | 100 | 1 |
| NC_022050.1 | Paracoccus aminophilus JCM 7686 | pAMI8 | 2.0E-35 | 95.05 | 94 | 23 |
| NC_010847.2 | Paracoccus aminophilus strain JCM 7686 | pAMI2 | 2.0E-36 | 95.05 | 100 | 2 |
| NC_013513.1 | Paracoccus aminophilus strain JCM 7686 | pAMI3 | 5.0E-37 | 95.05 | 101 | 2 |
| NC_014832.1 | Paracoccus aminophilus strain JCM 7686 | pAMI7 | 3.0E-39 | 97.03 | 99 | 11 |
| NC_008688.1 | Paracoccus denitrificans PD1222 | 1 | 2.0E-34 | 95 | 100 | 5 |
| NC_019289.1 | Paracoccus haeundaensis | pHAE1 | 2.0E-41 | 100 | 96 | 1 |
| NC_019367.1 | Paracoccus marcusii | pMARC5 | 2.0E-33 | 95.7 | 93 | 1 |
| NC_019356.1 | Paracoccus marcusii | pMOS6 | 1.0E-39 | 97.03 | 101 | 2 |
| NC_021241.1 | Paracoccus marcusii strain DSM 11574 | pMARC4 | 1.0E-36 | 95.05 | 101 | 1 |
| NC_009753.1 | Paracoccus methylutens strain DM12 | pMTH1 | 7.0E-38 | 96.97 | 99 | 30 |
| NC_019255.1 | Pasteurella aerogenes | pCCK343 | 4.0E-44 | 100 | 101 | 4 |
| NC_012216.1 | Pasteurella multocida | pB1006 | 2.0E-35 | 96.84 | 90 | 140 |
| NC_016973.1 | Pasteurella multocida | pCCK411 | 4.0E-44 | 100 | 101 | 5 |
| NC_004771.1 | Pasteurella multocida | pJR1 | 6.0E-37 | 95.05 | 101 | 2 |
| NC_010864.1 | Pediococcus acidilactici | pEOC01 | 7.0E-33 | 95.79 | 90 | 58 |
| NC_016636.1 | Pediococcus claussenii ATCC BAA-344 | pPECL-3 | 1.0E-43 | 100 | 101 | 3 |
| NC_017018.1 | Pediococcus claussenii ATCC BAA-344 | pPECL-7 | 6.0E-41 | 98.02 | 101 | 6 |
| NC_012031.1 | Pediococcus pentosaceus | pRS5 | 4.0E-41 | 98.02 | 101 | 1 |
| NC_008608.1 | Pelobacter propionicus DSM 2379 | pPRO2 | 6.0E-38 | 96.04 | 92 | 23 |
| NC_023148.1 | Phaeobacter gallaeciensis DSM 26640 | pGal_B134 | 9.0E-37 | 96 | 100 | 4 |
| NC_011143.1 | Phenylobacterium zucineum HLK1 | unnamed | 1.0E-39 | 98.02 | 100 | 14 |
| NC_016983.1 | Photobacterium damselae subsp. damselae | pAQU1 | 1.0E-42 | 100 | 101 | 5 |
| NC_008757.1 | Polaromonas naphthalenivorans CJ2 | pPNAP01 | 1.0E-33 | 95 | 100 | 5 |
| NC_008760.1 | Polaromonas naphthalenivorans CJ2 | pPNAP04 | 2.0E-38 | 97.03 | 101 | 2 |
| NC_007949.1 | Polaromonas sp. JS666 | 1 | 7.0E-37 | 96.04 | 101 | 1 |
| NC_007950.1 | Polaromonas sp. JS666 | 2 | 5.0E-32 | 95.65 | 92 | 2 |
| NC_022111.1 | Prevotella sp. oral taxon 299 str. F0039 |  | 3.0E-31 | 95 | 90 | 91 |
| NC_021086.1 | Propionibacterium acnes HL096PA1 |  | 1.0E-37 | 100 | 91 | 1 |
| NC_022197.1 | Proteus mirabilis strain BB1091 | pB1022 | 2.0E-44 | 100 | 101 | 1 |
| NC_010643.1 | Providencia rettgeri | R7K | 2.0E-40 | 98.02 | 101 | 6 |
| NC_022589.1 | Providencia rettgeri strain 09ACRGNY2001 | pPrY2001 | 8.0E-43 | 100 | 101 | 1 |
| NC_001621.1 | Pseudomonas aeruginosa | Birmingham IncP-alpha | 5.0E-43 | 100 | 101 | 1 |
| NC_008357.1 | Pseudomonas aeruginosa | pBS228 | 6.0E-43 | 100 | 101 | 1 |
| NC_009739.1 | Pseudomonas aeruginosa | pMATVIM-7 | 5.0E-38 | 96.04 | 101 | 15 |
| NC_022344.1 | Pseudomonas aeruginosa | pOZ176 | 6.0E-33 | 95.05 | 92 | 49 |
| NC_007100.1 | Pseudomonas aeruginosa | Rms149 | 6.0E-41 | 99 | 100 | 7 |
| NC_020452.1 | Pseudomonas aeruginosa strain COL-1 | pNOR-2000 | 1.0E-33 | 96.7 | 91 | 3 |
| NC_022346.1 | Pseudomonas aeruginosa strain ST308 | pCOL-1 | 3.0E-36 | 96 | 99 | 17 |
| NC_005909.1 | Pseudomonas alcaligenes | pRA2 | 1.0E-41 | 99.01 | 101 | 5 |
| NC_012674.1 | Pseudomonas fluorescens strain PC20 | pNAH20 | 8.0E-35 | 95.05 | 101 | 1 |
| NC_019906.1 | Pseudomonas putida HB3267 | pPC9 | 4.0E-32 | 95.6 | 91 | 3 |
| NC_004999.1 | Pseudomonas putida NCIB 9816-4 | pDTG1 | 2.0E-37 | 96.04 | 101 | 1 |
| NC_014124.1 | Pseudomonas putida | pDK1 | 1.0E-35 | 95.05 | 101 | 4 |
| NC_015855.1 | Pseudomonas putida | pGRT1 | 4.0E-35 | 95 | 100 | 8 |
| NC_013176.1 | Pseudomonas putida | pW2 | 6.0E-43 | 100 | 101 | 5 |
| NC_003350.1 | Pseudomonas putida | pWW0 | 3.0E-36 | 95.96 | 99 | 6 |
| NC_023274.1 | Pseudomonas putida strain LD209 | pLD209 | 3.0E-43 | 100 | 101 | 1 |
| NC_004956.1 | Pseudomonas sp. ADP atrazine catabolic | pADP-1 | 6.0E-32 | 95.05 | 91 | 31 |
| NC_010891.1 | Pseudomonas sp. CT14 | pCT14 | 6.0E-41 | 100 | 97 | 9 |
| NC_005244.2 | Pseudomonas sp. ND6 | pND6-1 | 3.0E-41 | 99.01 | 101 | 1 |
| NC_022739.1 | Pseudomonas sp. VLB120 | pSTY | 6.0E-37 | 96.04 | 101 | 17 |
| NC_007274.1 | Pseudomonas syringae pv. phaseolicola 1448A large |  | 3.0E-37 | 96.04 | 101 | 1 |
| NC_015314.1 | Pseudonocardia dioxanivorans CB1190 | pPSED01 | 3.0E-38 | 97.03 | 101 | 1 |
| NC_007968.1 | Psychrobacter cryohalolentis K5 | 1 | 3.0E-36 | 95.05 | 100 | 3 |
| NC_021158.1 | Psychrobacter maritimus | pKLH80 | 1.0E-36 | 95.05 | 91 | 49 |
| NC_021668.1 | Psychrobacter sp. G | psyG_26 | 2.0E-43 | 100 | 101 | 1 |
| NC_009516.1 | Psychrobacter sp. PRwf-1 | pRWF101 | 3.0E-38 | 96.04 | 101 | 3 |
| NC_017060.1 | Rahnella aquatilis HX2 | pRA1 | 3.0E-37 | 98.95 | 95 | 4 |
| NC_017807.1 | Rahnella aquatilis HX2 | pRA2 | 8.0E-39 | 100 | 93 | 3 |
| NC_019223.1 | Rahnella sp. WMR66 | pHW66 | 1.0E-38 | 96.04 | 101 | 1 |
| NC_005241.1 | Ralstonia eutropha H16 mega | pHG1 | 8.0E-37 | 96.04 | 101 | 1 |
| NC_012855.1 | Ralstonia pickettii 12D | pRp12D01 | 4.0E-34 | 95.83 | 96 | 1 |
| NC_012849.1 | Ralstonia pickettii 12D | pRp12D02 | 8.0E-35 | 95 | 92 | 21 |
| NC_019318.1 | Ralstonia pickettii | p712 | 5.0E-43 | 100 | 101 | 3 |
| NC_017589.1 | Ralstonia solanacearum CMR15 | CMR15_mp | 3.0E-30 | 95 | 90 | 24 |
| NC_021745.1 | Ralstonia solanacearum FQY_4 mega |  | 1.0E-30 | 95 | 90 | 106 |
| NC_003296.1 | Ralstonia solanacearum GMI1000 | pGMI1000MP | 1.0E-30 | 95 | 90 | 288 |
| NC_021909.1 | Rhizobium etli bv. mimosae str. Mim1 | pRetMIM1e | 4.0E-42 | 100 | 101 | 1 |
| NC_007762.1 | Rhizobium etli CFN 42 | p42a | 7.0E-40 | 98.02 | 101 | 1 |
| NC_007764.1 | Rhizobium etli CFN 42 | p42c | 2.0E-35 | 95.05 | 101 | 1 |
| NC_007765.1 | Rhizobium etli CFN 42 | p42e | 4.0E-35 | 95.05 | 101 | 1 |
| NC_007766.1 | Rhizobium etli CFN 42 | p42f | 5.0E-35 | 95.05 | 100 | 2 |
| NC_004041.2 | Rhizobium etli CFN 42 symbiotic | p42d | 3.0E-35 | 95.05 | 101 | 3 |
| NC_010996.1 | Rhizobium etli CIAT 652 | pB | 8.0E-37 | 96.04 | 101 | 1 |
| NC_011368.1 | Rhizobium leguminosarum bv. trifolii WSM2304 | pRLG201 | 2.0E-36 | 96.97 | 99 | 1 |
| NC_011370.1 | Rhizobium leguminosarum bv. trifolii WSM2304 | pRLG203 | 3.0E-35 | 95.05 | 101 | 2 |
| NC_011371.1 | Rhizobium leguminosarum bv. trifolii WSM2304 | pRLG204 | 5.0E-31 | 95.56 | 90 | 3 |
| NC_008381.1 | Rhizobium leguminosarum bv. viciae 3841 | pRL10 | 1.0E-34 | 95.05 | 95 | 3 |
| NC_008378.1 | Rhizobium leguminosarum bv. viciae 3841 | pRL12 | 2.0E-36 | 96.04 | 101 | 1 |
| NC_008382.1 | Rhizobium leguminosarum bv. viciae 3841 | pRL7 | 1.0E-35 | 95.92 | 98 | 3 |
| NC_022536.1 | Rhizobium sp. IRBG74 | IRBL74_p | 2.0E-40 | 99.01 | 101 | 4 |
| NC_020060.1 | Rhizobium tropici CIAT 899 | pRtrCIAT899a | 2.0E-35 | 95.05 | 100 | 3 |
| NC_020061.1 | Rhizobium tropici CIAT 899 | pRtrCIAT899b | 1.0E-36 | 96.04 | 101 | 1 |
| NC_009429.1 | Rhodobacter sphaeroides ATCC 17025 | pRSPA01 | 2.0E-30 | 95 | 90 | 43 |
| NC_009430.1 | Rhodobacter sphaeroides ATCC 17025 | pRSPA02 | 4.0E-32 | 95.05 | 90 | 14 |
| NC_005073.1 | Rhodococcus erythropolis linear | pBD2 | 3.0E-39 | 98 | 100 | 1 |
| NC_007491.1 | Rhodococcus erythropolis PR4 | pREL1 | 1.0E-39 | 98.02 | 101 | 2 |
| NC_008269.1 | Rhodococcus jostii RHA1 | pRHL1 | 3.0E-34 | 95 | 100 | 1 |
| NC_012520.1 | Rhodococcus opacus B4 | pROB01 | 8.0E-32 | 95.65 | 92 | 2 |
| NC_019261.1 | Riemerella anatipestifer | pRA0511 | 9.0E-44 | 100 | 101 | 1 |
| NC_015956.1 | Riemerella anatipestifer | pRA0726 | 9.0E-44 | 100 | 101 | 2 |
| NC_006569.1 | Ruegeria pomeroyi DSS-3 mega |  | 1.0E-34 | 95 | 100 | 1 |
| NC_008043.1 | Ruegeria sp. TM1040 mega |  | 2.0E-36 | 96.04 | 99 | 6 |
| NC_008042.1 | Ruegeria sp. TM1040 | unnamed | 6.0E-33 | 95 | 93 | 5 |
| NC_014825.1 | Ruminococcus albus 7 | pRUMAL02 | 5.0E-32 | 95.65 | 92 | 3 |
| NC_001398.1 | Saccharomyces cerevisiae A364A 2 micron circle |  | 3.0E-34 | 95.05 | 90 | 109 |
| NC_021871.1 | Salmonella bongori N268-08 | RM1 | 2.0E-37 | 96.04 | 101 | 1 |
| NC_021817.1 | Salmonella enterica subsp. enterica serovar Bareilly str. CFSAN000189 | unnamed | 2.0E-41 | 99.01 | 101 | 2 |
| NC_010499.1 | Salmonella enterica subsp. enterica serovar Brandenburg | pUO-SbR3 | 5.0E-44 | 100 | 101 | 7 |
| NC_010500.1 | Salmonella enterica subsp. enterica serovar Brandenburg | pUO-SbR5 | 2.0E-42 | 99.01 | 101 | 3 |
| NC_021819.1 | Salmonella enterica subsp. enterica Serovar Cubana str. CFSAN002050 |  | 9.0E-43 | 100 | 101 | 2 |
| NC_019134.1 | Salmonella enterica subsp. enterica serovar Derby | pSD4.0 | 3.0E-44 | 100 | 101 | 1 |
| NC_010421.1 | Salmonella enterica subsp. enterica serovar Dublin | pOU1114 | 1.0E-41 | 99.01 | 101 | 1 |
| NC_019105.1 | Salmonella enterica subsp. enterica serovar Dublin | pSD_88 | 3.0E-41 | 99.01 | 101 | 2 |
| NC_022267.1 | Salmonella enterica subsp. enterica serovar Enteritidis strain S1400/94 | pS1400_89 | 3.0E-41 | 99.01 | 101 | 1 |
| NC_019114.1 | Salmonella enterica subsp. enterica serovar Heidelberg | pSH111_227 | 7.0E-41 | 99.01 | 99 | 4 |
| NC_019123.1 | Salmonella enterica subsp. enterica serovar Heidelberg | pSH1148_107 | 9.0E-36 | 95.05 | 100 | 16 |
| NC_019115.1 | Salmonella enterica subsp. enterica serovar Heidelberg | pSH146_65 | 4.0E-38 | 97 | 99 | 14 |
| NC_019131.1 | Salmonella enterica subsp. enterica serovar Heidelberg | pSH146_87 | 6.0E-43 | 100 | 101 | 13 |
| NC_019117.1 | Salmonella enterica subsp. enterica serovar Heidelberg | pSH696_117 | 2.0E-38 | 97.03 | 101 | 5 |
| NC_021811.1 | Salmonella enterica subsp. enterica serovar Heidelberg str. 41578 | pSEEH1578_01 | 9.0E-43 | 100 | 101 | 4 |
| NC_021813.2 | Salmonella enterica subsp. enterica serovar Heidelberg str. CFSAN002069 | pCFSAN002069_01 | 8.0E-43 | 100 | 101 | 1 |
| NC_011082.1 | Salmonella enterica subsp. enterica serovar Heidelberg str. SL476 | pSL476_3 | 1.0E-42 | 99.01 | 101 | 2 |
| NC_011081.1 | Salmonella enterica subsp. enterica serovar Heidelberg str. SL476 | pSL476_91 | 3.0E-40 | 98.02 | 101 | 2 |
| NC_011077.1 | Salmonella enterica subsp. enterica serovar Kentucky str. CVM29188 | pCVM29188_101 | 7.0E-43 | 100 | 101 | 2 |
| NC_011076.1 | Salmonella enterica subsp. enterica serovar Kentucky str. CVM29188 | pCVM29188_146 | 1.0E-42 | 100 | 101 | 3 |
| NC_011078.1 | Salmonella enterica subsp. enterica serovar Kentucky str. CVM29188 | pCVM29188_46 | 8.0E-39 | 97.03 | 101 | 3 |
| NC_010259.1 | Salmonella enterica subsp. enterica serovar Newport | pA172 | 6.0E-44 | 100 | 101 | 4 |
| NC_010894.1 | Salmonella enterica subsp. enterica serovar Paratyphi A | pGY1 | 3.0E-44 | 100 | 101 | 1 |
| NC_003384.1 | Salmonella enterica subsp. enterica serovar Typhi str. CT18 | pHCM1 | 4.0E-37 | 96.04 | 101 | 23 |
| NC_003385.1 | Salmonella enterica subsp. enterica serovar Typhi str. CT18 | pHCM2 | 7.0E-37 | 96.94 | 98 | 1 |
| NC_016825.1 | Salmonella enterica subsp. enterica serovar Typhi str. P-stx-12 | unnamed | 1.0E-42 | 100 | 101 | 2 |
| NC_002090.1 | Salmonella enterica subsp. enterica serovar Typhimurium | NTP16 | 6.0E-44 | 100 | 101 | 3 |
| NC_019111.1 | Salmonella enterica subsp. enterica serovar Typhimurium | pSal8934a | 6.0E-37 | 96.04 | 90 | 3 |
| NC_002056.1 | Salmonella enterica subsp. enterica serovar Typhimurium | pSC101 | 7.0E-44 | 100 | 101 | 1 |
| NC_015570.1 | Salmonella enterica subsp. enterica serovar Typhimurium | pSe-Kan | 3.0E-41 | 98.02 | 101 | 1 |
| NC_009807.1 | Salmonella enterica subsp. enterica serovar Typhimurium | pTPqnrS-1a | 1.0E-35 | 95.05 | 101 | 2 |
| NC_006816.1 | Salmonella enterica subsp. enterica serovar Typhimurium | pU302L | 6.0E-43 | 100 | 101 | 7 |
| NC_003292.1 | Salmonella enterica subsp. enterica serovar Typhimurium | R46 | 2.0E-40 | 98.02 | 101 | 1 |
| NC_015965.1 | Salmonella enterica subsp. enterica serovar Typhimurium | R621a | 3.0E-41 | 99.01 | 101 | 7 |
| NC_017675.1 | Salmonella enterica subsp. enterica serovar Typhimurium str. ST4/74 | TY474p2 | 2.0E-42 | 100 | 100 | 9 |
| NC_023275.1 | Salmonella enterica subsp. enterica serovar Typhimurium strain 9134 | p9134 | 5.0E-40 | 98.02 | 101 | 21 |
| NC_021815.1 | Salmonella enterica subsp. enterica serovar Typhimurium var. 5- str. CFSAN001921 | unnamed | 2.0E-35 | 95.05 | 99 | 40 |
| NT_187135.1 | Salmonella enterica subsp. enterica serovar Weltevreden str. 2007-60-3289-1 | pSW82 | 7.0E-36 | 95.05 | 100 | 4 |
| NC_011604.1 | Salmonella enterica subsp. enterica serovar Westhampton | pWES-1 | 2.0E-38 | 96.04 | 101 | 1 |
| NC_003456.1 | Salmonella enteritidis | pK | 3.0E-44 | 100 | 101 | 1 |
| NC_019335.1 | Salmonella sp. 14 | p14-95A | 6.0E-37 | 96 | 100 | 1 |
| NC_002305.1 | Salmonella typhi | R27 | 1.0E-42 | 100 | 101 | 6 |
| NC_013518.1 | Sebaldella termitidis ATCC 33386 | pSTERM01 | 5.0E-36 | 95.05 | 99 | 13 |
| NC_021742.1 | Serratia liquefaciens ATCC 27592 |  | 8.0E-32 | 95 | 90 | 245 |
| NC_019267.1 | Serratia marcescens | pRIO-5 | 1.0E-43 | 100 | 101 | 1 |
| NC_019344.1 | Serratia marcescens | R830b | 2.0E-35 | 96.84 | 95 | 11 |
| NC_017580.1 | Shewanella baltica OS117 | pSBAL11702 | 2.0E-37 | 96.04 | 101 | 1 |
| NC_009661.1 | Shewanella baltica OS185 | pS18501 | 3.0E-40 | 98.02 | 101 | 1 |
| NC_008573.1 | Shewanella sp. ANA-3 | 1 | 3.0E-34 | 95.05 | 94 | 63 |
| NC_010660.1 | Shigella boydii CDC 3083-94 | pBS512_211 | 3.0E-38 | 100 | 93 | 1 |
| NC_007608.1 | Shigella boydii Sb227 | pSB4_227 | 2.0E-37 | 96.04 | 101 | 3 |
| NC_007607.1 | Shigella dysenteriae Sd197 | pSD1_197 | 7.0E-40 | 98.02 | 99 | 3 |
| NC_002134.1 | Shigella flexneri 2b | R100 DNA | 7.0E-43 | 100 | 101 | 1 |
| NC_002698.1 | Shigella flexneri 5a virulence | pWR501 | 2.0E-35 | 95.05 | 101 | 10 |
| NC_020991.1 | Shigella sonnei 10188 | pKHSB1 | 7.0E-43 | 100 | 101 | 1 |
| NC_022585.1 | Shigella sonnei eg211 | pDPT1 | 5.0E-44 | 100 | 101 | 1 |
| NC_002122.1 | Shigella sonnei | p9 DNA | 7.0E-43 | 100 | 101 | 1 |
| NC_013727.1 | Shigella sonnei | pEG356 | 5.0E-43 | 100 | 101 | 1 |
| NC_009346.1 | Shigella sonnei Ss046 | pSS046_spB | 1.0E-38 | 96.04 | 101 | 1 |
| NC_019256.1 | Shigella sp. LN126 | pLN126_33 | 2.0E-43 | 100 | 101 | 1 |
| NC_019254.1 | Shigella sp. MO17 | pMO17_54 | 2.0E-35 | 95.05 | 95 | 13 |
| NC_016813.1 | Sinorhizobium fredii HH103 | pSfHH103a | 5.0E-38 | 96.04 | 101 | 1 |
| NC_016814.1 | Sinorhizobium fredii HH103 | pSfHH103c | 8.0E-38 | 97 | 100 | 2 |
| NC_016815.1 | Sinorhizobium fredii HH103 | pSfHH103e | 6.0E-34 | 95 | 100 | 3 |
| NC_012586.1 | Sinorhizobium fredii NGR234 | pNGR234b | 2.0E-33 | 96.97 | 91 | 4 |
| NT_187151.1 | Sinorhizobium fredii USDA 257 | pUSDA257 fragment 2 | 1.0E-35 | 95.05 | 101 | 2 |
| NC_009620.1 | Sinorhizobium medicae WSM419 | pSMED01 | 1.0E-34 | 95.05 | 101 | 1 |
| NC_009622.1 | Sinorhizobium medicae WSM419 | pSMED03 | 8.0E-40 | 98.02 | 101 | 1 |
| NC_020527.1 | Sinorhizobium meliloti 2011 | pSymA | 2.0E-37 | 97.03 | 101 | 1 |
| NC_015592.1 | Sinorhizobium meliloti AK83 | pSINME02 | 9.0E-34 | 95.05 | 92 | 5 |
| NC_017323.1 | Sinorhizobium meliloti BL225C | pSINMEB02 | 2.0E-31 | 95.05 | 92 | 3 |
| NC_019846.1 | Sinorhizobium meliloti GR4 | pRmeGR4a | 3.0E-37 | 96.04 | 101 | 1 |
| NC_019848.1 | Sinorhizobium meliloti GR4 | pRmeGR4c | 7.0E-31 | 95.6 | 91 | 1 |
| NC_010865.1 | Sinorhizobium meliloti | pSmeSM11b | 3.0E-38 | 97.03 | 101 | 2 |
| NC_018682.1 | Sinorhizobium meliloti Rm41 | pRM41A | 2.0E-36 | 96 | 100 | 1 |
| NC_018701.1 | Sinorhizobium meliloti Rm41 | pSYMB | 1.0E-34 | 95.05 | 101 | 1 |
| NC_017327.1 | Sinorhizobium meliloti SM11 | pSmeSM11c | 3.0E-36 | 96.04 | 101 | 1 |
| NC_017326.1 | Sinorhizobium meliloti SM11 | pSmeSM11d | 2.0E-37 | 97.96 | 98 | 1 |
| NC_021209.1 | Sinorhizobium sp. M14 | pSinA | 9.0E-36 | 95.05 | 100 | 25 |
| NC_015595.1 | Sphingobium chlorophenolicum L-1 | pSPHCH01 | 9.0E-43 | 100 | 101 | 3 |
| NC_019376.1 | Sphingobium fuliginis ATCC 27551 | pPDL2 | 3.0E-43 | 100 | 101 | 1 |
| NC_014007.1 | Sphingobium japonicum UT26S | pCHQ1 | 2.0E-35 | 95.05 | 100 | 3 |
| NC_015974.1 | Sphingobium sp. SYK-6 | pSLPG | 2.0E-39 | 99.01 | 95 | 5 |
| NC_008246.1 | Sphingobium yanoikuyae | pYAN-1 DNA | 1.0E-38 | 98.94 | 94 | 1 |
| NC_022235.1 | Sphingomonas sp. ERG5 | pCADAB1 | 3.0E-36 | 95.96 | 99 | 2 |
| NC_008308.1 | Sphingomonas sp. KA1 | pCAR3 DNA | 6.0E-36 | 96.91 | 97 | 4 |
| NC_020542.1 | Sphingomonas sp. MM-1 | pISP0 | 5.0E-37 | 96.04 | 95 | 7 |
| NC_020562.1 | Sphingomonas sp. MM-1 | pISP1 | 3.0E-37 | 99.01 | 91 | 11 |
| NC_020543.1 | Sphingomonas sp. MM-1 | pISP2 | 1.0E-37 | 96.04 | 101 | 3 |
| NC_009507.1 | Sphingomonas wittichii RW1 | pSWIT01 | 9.0E-35 | 95 | 92 | 9 |
| NC_009508.1 | Sphingomonas wittichii RW1 | pSWIT02 | 6.0E-35 | 95 | 100 | 9 |
| NC_018974.1 | Staphylococcus aureus | p18811-P03 | 2.0E-43 | 100 | 101 | 1 |
| NC_002517.1 | Staphylococcus aureus | p21 | 2.0E-43 | 100 | 101 | 2 |
| NC_012547.1 | Staphylococcus aureus | pGO1 | 4.0E-43 | 100 | 101 | 1 |
| NC_010419.1 | Staphylococcus aureus | pTZ2162 | 3.0E-43 | 100 | 101 | 1 |
| NC_005127.1 | Staphylococcus aureus | pUB101 | 7.0E-42 | 99.01 | 101 | 2 |
| NC_001384.1 | Staphylococcus aureus | pUB110 | 4.0E-44 | 100 | 101 | 1 |
| NC_013328.1 | Staphylococcus aureus | pWBG753 | 2.0E-43 | 100 | 101 | 1 |
| NC_013320.1 | Staphylococcus aureus | SAP014A | 4.0E-43 | 100 | 101 | 1 |
| NC_013321.1 | Staphylococcus aureus | SAP017A | 2.0E-43 | 100 | 101 | 1 |
| NC_013324.1 | Staphylococcus aureus | SAP027A | 2.0E-43 | 100 | 101 | 1 |
| NC_013340.1 | Staphylococcus aureus | SAP076A | 6.0E-39 | 97.03 | 101 | 1 |
| NC_022126.1 | Staphylococcus aureus subsp. aureus 55/2053 |  | 7.0E-42 | 99.01 | 101 | 1 |
| NC_010066.1 | Staphylococcus aureus subsp. aureus USA300_TCH959 | pUSA300HOUMS | 2.0E-43 | 100 | 101 | 1 |
| NC_022598.1 | Staphylococcus aureus TY825 | pETB DNA | 5.0E-43 | 100 | 101 | 2 |
| NC_003969.1 | Staphylococcus epidermidis | pSepCH | 2.0E-44 | 100 | 101 | 3 |
| NC_013374.1 | Staphylococcus epidermidis | SAP045A | 1.0E-35 | 98.89 | 90 | 2 |
| NC_013381.1 | Staphylococcus epidermidis | SAP107A | 7.0E-39 | 97.03 | 101 | 4 |
| NC_013382.1 | Staphylococcus epidermidis | SAP107B | 4.0E-41 | 98.02 | 101 | 1 |
| NC_013383.1 | Staphylococcus epidermidis | SAP110A | 2.0E-36 | 95.05 | 101 | 4 |
| NC_016139.1 | Staphylococcus hyicus | p9811071-1 | 7.0E-40 | 97.96 | 98 | 1 |
| NC_020237.1 | Staphylococcus hyicus | pSTE1 c | 4.0E-42 | 99.01 | 101 | 3 |
| NC_007351.1 | Staphylococcus saprophyticus subsp. saprophyticus ATCC 15305 | pSSP1 | 3.0E-36 | 95.05 | 97 | 5 |
| NC_007352.1 | Staphylococcus saprophyticus subsp. saprophyticus ATCC 15305 | pSSP2 | 7.0E-42 | 99.01 | 101 | 1 |
| NC_015432.1 | Staphylococcus saprophyticus subsp. saprophyticus MS1146 | pSSAP1 | 1.0E-38 | 97.03 | 101 | 1 |
| NC_016643.1 | Staphylococcus saprophyticus subsp. saprophyticus | pSSAP2 | 1.0E-40 | 98.02 | 101 | 3 |
| NC_013944.1 | Staphylococcus simulans bv. staphylolyticus strain NRRL B-2628 | pACK1 | 4.0E-43 | 100 | 101 | 1 |
| NC_009130.1 | Staphylococcus sp. 693-2 | pLEW6932 | 4.0E-43 | 100 | 101 | 2 |
| NC_013371.1 | Staphylococcus sp. 693-2 | SAP008A | 6.0E-39 | 97 | 100 | 1 |
| NC_013388.1 | Staphylococcus sp. CDC25 | SAP018B | 3.0E-41 | 98.02 | 101 | 1 |
| NC_007165.1 | Staphylococcus warneri | pSW174 | 4.0E-44 | 100 | 101 | 1 |
| NC_020274.1 | Staphylococcus warneri SG1 | clonepvSw1 | 1.0E-36 | 95.05 | 101 | 3 |
| NC_020267.1 | Staphylococcus warneri SG1 | clonepvSw5 | 3.0E-38 | 96.97 | 99 | 2 |
| NC_020268.1 | Staphylococcus warneri SG1 | clonepvSw6 | 5.0E-44 | 100 | 101 | 1 |
| NC_001797.1 | Streptococcus agalactiae | pGB354 | 2.0E-42 | 99.01 | 101 | 2 |
| NC_002136.1 | Streptococcus agalactiae | pGB3634 | 2.0E-41 | 98.02 | 96 | 4 |
| NC_015219.1 | Streptococcus gallolyticus subsp. gallolyticus ATCC BAA-2069 | pSGG1 | 1.0E-32 | 95 | 91 | 199 |
| NC_016837.1 | Streptococcus infantarius subsp. infantarius CJ18 | pSICJ18-1 | 5.0E-37 | 96.97 | 99 | 5 |
| NC_019365.1 | Streptococcus infantis | pSI01 | 9.0E-44 | 100 | 101 | 2 |
| NC_016750.1 | Streptococcus macedonicus ACA-DC 198 | pSMA198 | 4.0E-42 | 99.01 | 101 | 2 |
| NC_010230.1 | Streptococcus pyogenes | pDN281 | 2.0E-40 | 97.98 | 99 | 1 |
| NC_006979.1 | Streptococcus pyogenes | pSM19035 | 2.0E-43 | 100 | 101 | 1 |
| NC_012923.1 | Streptococcus suis BM407 | pBM407 | 7.0E-36 | 95 | 99 | 3 |
| NC_000937.1 | Streptococcus thermophilus ST135 | pER35 | 3.0E-42 | 99.01 | 101 | 2 |
| NC_004747.1 | Streptococcus thermophilus ST2-1 | pND103 | 3.0E-44 | 100 | 101 | 2 |
| NC_001898.1 | Tatumella citrea | pUCD5000 | 4.0E-44 | 100 | 101 | 1 |
| NC_010938.1 | Tetragenococcus halophilus | pSKPB18 DNA | 3.0E-44 | 100 | 101 | 1 |
| NC_011667.1 | Thauera sp. MZ1T | pTha01 | 2.0E-37 | 96.04 | 101 | 1 |
| NC_011961.1 | Thermomicrobium roseum DSM 5159 | unnamed | 1.0E-31 | 95.65 | 92 | 12 |
| NC_019388.1 | Thermus oshimai JL-2 | pTHEOS02 | 4.0E-43 | 100 | 101 | 2 |
| NC_017273.1 | Thermus thermophilus SG0.5JP17-16 | pTHTHE1601 | 4.0E-35 | 95.05 | 101 | 2 |
| NC_014154.1 | Thiomonas intermedia K12 | pTINT01 | 6.0E-34 | 96.04 | 91 | 25 |
| NC_014155.1 | thiomonas intermedia K12 | pTINT02 | 3.0E-39 | 97.03 | 101 | 5 |
| NC_014144.1 | Thiomonas sp. str. 3As | pTHI | 4.0E-36 | 95.05 | 101 | 3 |
| NC_017966.1 | Tistrella mobilis KA081020-065 | pTM2 | 6.0E-35 | 95.05 | 101 | 2 |
| NC_017958.1 | Tistrella mobilis KA081020-065 | pTM3 | 2.0E-30 | 95 | 90 | 351 |
| NC_017959.1 | Tistrella mobilis KA081020-065 | pTM4 | 4.0E-32 | 95 | 90 | 43 |
| NC_005206.1 | Trueperella pyogenes | pAP2 | 7.0E-44 | 100 | 101 | 2 |
| NC_006385.1 | Uncultured bacterium activated sludge | pRSB101 | 3.0E-38 | 97 | 97 | 19 |
| NC_006352.1 | Uncultured bacterium activated sludge | pTB11 | 2.0E-41 | 99.01 | 101 | 12 |
| NC_019299.1 | Uncultured bacterium HH1107 | pHH1107 | 7.0E-34 | 95.05 | 91 | 24 |
| NC_019217.1 | Uncultured bacterium HHV216 | pHHV216 | 1.0E-31 | 95.05 | 90 | 36 |
| NC_019218.1 | Uncultured bacterium HHV35 | pHHV35 | 3.0E-33 | 95.05 | 90 | 271 |
| NC_007502.1 | Uncultured bacterium IncP-1beta multiresistance | pB8 | 2.0E-41 | 99.01 | 101 | 4 |
| NC_008055.1 | Uncultured bacterium IncP-1gamma | QKH54 | 2.0E-35 | 95 | 90 | 128 |
| NC_004840.1 | Uncultured bacterium | pB10 | 4.0E-38 | 97 | 100 | 2 |
| NC_019022.1 | Uncultured bacterium | pB11 | 5.0E-43 | 100 | 101 | 1 |
| NC_003430.1 | Uncultured bacterium | pB4 | 7.0E-36 | 95.05 | 101 | 13 |
| NC_019020.1 | Uncultured bacterium | pB5 | 5.0E-43 | 100 | 101 | 3 |
| NC_003213.2 | Uncultured bacterium | pIPO2T | 4.0E-36 | 95.05 | 101 | 1 |
| NC_008330.1 | Uncultured bacterium | pLB1 DNA | 1.0E-38 | 97.03 | 101 | 4 |
| NC_019216.1 | Uncultured bacterium | pRSB105 | 2.0E-35 | 95 | 98 | 25 |
| NC_003122.1 | Uncultured bacterium | pSB102. | 2.0E-35 | 95.05 | 97 | 5 |
| NC_019021.1 | Uncultured bacterium | pSP21 | 1.0E-37 | 96.04 | 101 | 17 |
| NC_007680.1 | Uncultured bacterium | pTP6 | 4.0E-43 | 100 | 101 | 4 |
| NC_013278.1 | Uncultured bacterium | pTRACA18 | 5.0E-37 | 95.05 | 92 | 13 |
| NC_013279.1 | Uncultured bacterium | pTRACA20 | 3.0E-38 | 96 | 100 | 12 |
| NC_013280.1 | Uncultured bacterium | pTRACA22 | 1.0E-32 | 95 | 90 | 569 |
| NC_013281.1 | Uncultured bacterium | pTRACA30 | 2.0E-40 | 99 | 100 | 12 |
| NC_019324.1 | UNVERIFIED: Clostridium sp. MT351 | unnamed | 2.0E-33 | 95.6 | 91 | 9 |
| NC_019352.1 | UNVERIFIED: Leuconostoc mesenteroides subsp. mesenteroides | pMBLT00 | 2.0E-36 | 95.05 | 98 | 6 |
| NC_019320.1 | Variovorax sp. DB1 | pDB1 | 1.0E-37 | 96.04 | 101 | 1 |
| NC_008771.1 | Verminephrobacter eiseniae EF01-2 | pVEIS01 | 9.0E-36 | 95 | 100 | 3 |
| NC_023291.1 | Vibrio cholerae strain BI144 | pVCR94deltaX | 4.0E-35 | 97.83 | 92 | 2 |
| NC_020451.1 | Vibrio coralliilyticus strain ATCC BAA-450 |  | 1.0E-33 | 95.7 | 93 | 5 |
| NC_004341.2 | Weissella cibaria | pKLCB | 1.0E-36 | 95.1 | 101 | 2 |
| NC_015756.1 | Weissella koreensis KACC 15510 | WKp2903 | 4.0E-38 | 96.04 | 101 | 4 |
| NC_009717.1 | Xanthobacter autotrophicus Py2 | pXAUT01 | 6.0E-37 | 96.04 | 101 | 39 |
| NC_016053.1 | Xanthomonas arboricola pv. pruni str. CFBP 5530 | pXap41 | 4.0E-36 | 95.05 | 101 | 1 |
| NC_007507.1 | Xanthomonas campestris pv. vesicatoria str. 85-10 | pXCV183 | 4.0E-37 | 96.04 | 101 | 1 |
| NC_020817.1 | Xanthomonas citri subsp. citri Aw12879 | pXcaw58 | 4.0E-37 | 96 | 100 | 1 |
| NC_022539.1 | Xanthomonas fuscans subsp. fuscans str. 4834-R | pla | 6.0E-34 | 95.74 | 94 | 1 |
| NC_005570.1 | Yersinia enterocolitica 29807 | p29807 | 9.0E-43 | 99.01 | 101 | 1 |
| NC_012209.1 | Yersinia enterocolitica | pYe4449-2 | 1.0E-43 | 100 | 101 | 2 |
| NC_019269.1 | Yersinia frederiksenii | pYF27601 | 4.0E-44 | 100 | 101 | 9 |
| NC_005814.1 | Yersinia pestis biovar Microtus str. 91001 | pCRY | 2.0E-36 | 95.05 | 101 | 5 |
| NC_009141.1 | Yersinia pestis biovar Orientalis str. IP275 | pIP1202 | 3.0E-32 | 96.67 | 90 | 3 |
| NC_003134.1 | Yersinia pestis CO92 | pMT1 | 2.0E-37 | 96.04 | 101 | 1 |
| NC_006323.1 | Yersinia pestis | pG8786 | 1.0E-42 | 100 | 101 | 1 |
| NC_009139.1 | Yersinia ruckeri YR71 | pYR1 | 5.0E-41 | 99.01 | 101 | 3 |

**Assigned contigs of sample C1754 against the RefSeq plasmid database**

(Sorted by Organism)

| **Accession number** | **Organism** | **Plasmid** | **E value ≤** | **Identity (%) ≥** | **Hit length (bp) ≥** | **Number of contigs** |
| --- | --- | --- | --- | --- | --- | --- |
| NC_017163.1 | Acinetobacter baumannii 1656-2 | ABKp1 | < 1.0E-150 | 99.41 | 1196 | 1 |
| NC_017164.1 | Acinetobacter baumannii 1656-2 | ABKp2 | 6.0E-42 | 98.99 | 99 | 1 |
| NC_010605.1 | Acinetobacter baumannii ACICU | pACICU1 | < 1.0E-150 | 97.52 | 483 | 1 |
| NC_010404.1 | Acinetobacter baumannii AYE | p3ABAYE | 3.0E-127 | 95.08 | 285 | 15 |
| NC_021734.1 | Acinetobacter baumannii BJAB0715 | pBJAB0715 | 5.0E-147 | 97.65 | 291 | 4 |
| NC_017848.1 | Acinetobacter baumannii MDR-TJ | pABTJ1 | < 1.0E-150 | 100 | 1180 | 1 |
| NC_020524.1 | Acinetobacter baumannii MDR-TJ | pABTJ2 | < 1.0E-150 | 97.56 | 862 | 1 |
| NC_010481.1 | Acinetobacter baumannii | pABIR | 4.0E-38 | 97.89 | 95 | 3 |
| NC_013506.1 | Acinetobacter baumannii | pMMCU2 | 1.0E-122 | 97.34 | 263 | 1 |
| NC_019280.1 | Acinetobacter baumannii | pMMD | < 1.0E-150 | 98.44 | 320 | 1 |
| NC_019345.1 | Acinetobacter baumannii | pRAY*-v2 | < 1.0E-150 | 99.14 | 317 | 3 |
| NC_010396.1 | Acinetobacter baumannii SDF | p2ABSDF | < 1.0E-150 | 95.58 | 566 | 1 |
| NC_020818.1 | Acinetobacter baumannii strain GF216 | pNDM-AB | < 1.0E-150 | 99.4 | 503 | 1 |
| NC_017166.1 | Acinetobacter baumannii TCDC-AB0715 | p2ABTCDC0715 | < 1.0E-150 | 99.36 | 1096 | 1 |
| NC_010310.1 | Acinetobacter venetianus strain VE-C3 | pAV2 | < 1.0E-150 | 96.99 | 730 | 2 |
| NC_006143.1 | Aeromonas caviae | pFBAOT6 | < 1.0E-150 | 95.52 | 509 | 2 |
| NC_009349.1 | Aeromonas salmonicida subsp. salmonicida A449 | 4 | < 1.0E-150 | 97.63 | 379 | 1 |
| NC_008712.1 | Arthrobacter aurescens TC1 | TC1 | < 1.0E-150 | 100 | 3168 | 1 |
| NC_014937.1 | Bacillus thuringiensis CT43 | pBMB0558 | < 1.0E-150 | 96.56 | 349 | 2 |
| NC_005026.1 | Bacteroides fragilis IB143 | pBI143 | < 1.0E-150 | 97.31 | 380 | 2 |
| NC_011073.1 | Bacteroides fragilis | pBFP35 | < 1.0E-150 | 100 | 347 | 1 |
| NC_019534.1 | Bacteroides fragilis | pBFUK1 | < 1.0E-150 | 98.64 | 353 | 3 |
| NC_011332.1 | Bifidobacterium bifidum | pB80 | < 1.0E-150 | 100 | 341 | 1 |
| NC_004252.1 | Bifidobacterium longum DJO10A | pDOJH10L | < 1.0E-150 | 100 | 608 | 1 |
| NC_010857.1 | Bifidobacterium longum | p6043A | 6.0E-122 | 98.03 | 243 | 4 |
| NC_004768.1 | Bifidobacterium longum RW041 | pNAC3 | < 1.0E-150 | 97.97 | 344 | 2 |
| NC_004770.1 | Bifidobacterium longum RW048 | pNAC1 | < 1.0E-150 | 97.93 | 376 | 2 |
| NC_015053.1 | Bifidobacterium longum subsp. infantis 157F | p157F-NC1 | < 1.0E-150 | 99.84 | 616 | 1 |
| NC_004443.1 | Bifidobacterium longum VMKB44 | pB44 | < 1.0E-150 | 98.42 | 317 | 2 |
| NC_019378.1 | Burkholderia cepacia | pIJB1 | < 1.0E-150 | 99.71 | 339 | 2 |
| NC_019369.1 | Burkholderia cepacia | pYS1 | < 1.0E-150 | 100 | 413 | 1 |
| NC_009227.1 | Burkholderia vietnamiensis G4 | pBVIE02 | < 1.0E-150 | 99.57 | 322 | 2 |
| NC_022355.1 | Campylobacter coli CVM N29710 | pN29710-1 | 3.0E-61 | 96.83 | 141 | 2 |
| NC_006134.1 | Campylobacter coli | pCC31 | 1.0E-83 | 95.58 | 182 | 2 |
| NC_008790.1 | Campylobacter jejuni subsp. jejuni 81-176 | pTet | 4.0E-78 | 96.07 | 167 | 3 |
| NC_014801.1 | Campylobacter jejuni subsp. jejuni ICDCCJ07001 | pTet | < 1.0E-150 | 97.79 | 633 | 2 |
| NC_019259.1 | Clostridium perfringens | pJIR3537 | < 1.0E-150 | 99.7 | 333 | 1 |
| NC_010935.1 | Comamonas testosteroni CNB-1 | pCNB | < 1.0E-150 | 99.44 | 411 | 2 |
| NC_002143.1 | Comamonas testosteroni PtL5 cryptic | pPT1 | < 1.0E-150 | 99.13 | 346 | 1 |
| NC_009779.1 | Cronobacter sakazakii ATCC BAA-894 | pESA2 | < 1.0E-150 | 98.3 | 763 | 1 |
| NC_019312.1 | Delftia sp. KV29 | pKV29 | < 1.0E-150 | 97.53 | 433 | 3 |
| NC_012555.1 | Enterobacter cloacae | pEC-IMP | 2.0E-72 | 96.39 | 158 | 3 |
| NC_013514.1 | Enterococcus faecalis | pAMbeta1 | < 1.0E-150 | 99.03 | 925 | 1 |
| NC_008445.1 | Enterococcus faecalis RE25 | pRE25 | < 1.0E-150 | 98.77 | 325 | 1 |
| NC_021987.1 | Enterococcus faecium Aus0085 | p1 | 1.0E-109 | 97.88 | 236 | 1 |
| NC_017963.1 | Enterococcus faecium DO | 3 | 5.0E-73 | 97.59 | 166 | 2 |
| NC_020208.1 | Enterococcus faecium NRRL B-2354 | pNB2354_1 | 8.0E-114 | 97.56 | 246 | 1 |
| NC_013317.1 | Enterococcus faecium | p5753cA | < 1.0E-150 | 99.01 | 404 | 1 |
| NC_014959.1 | Enterococcus faecium | pS177 | < 1.0E-150 | 99.74 | 391 | 1 |
| NC_007682.3 | Escherichia coli | pMUR050 | < 1.0E-150 | 100 | 436 | 2 |
| NC_018994.1 | Escherichia coli | pNDM-1_Dok01 | < 1.0E-150 | 98.07 | 519 | 1 |
| NC_009716.1 | Escherichia sp. Sflu5 cryptic | pAK51 | 5.0E-48 | 98.21 | 112 | 1 |
| NC_012782.1 | Eubacterium eligens ATCC 27750 | unnamed | 1.0E-54 | 98.48 | 328 | 1 |
| NC_012780.1 | Eubacterium eligens ATCC 27750 | unnamed | < 1.0E-150 | 95.06 | 131 | 50 |
| NC_012661.1 | Haemophilus parasuis | pHN61 | < 1.0E-150 | 99.54 | 305 | 3 |
| NC_018107.1 | Klebsiella oxytoca E718 | pKOX_R1 | 6.0E-147 | 97.09 | 378 | 1 |
| NC_019390.1 | Klebsiella pneumoniae | pKPN_CZ | 8.0E-142 | 95.27 | 325 | 4 |
| NC_014478.1 | Klebsiella pneumoniae | unnamed | < 1.0E-150 | 95.73 | 288 | 2 |
| NC_021078.1 | Klebsiella pneumoniae strain Kp002 | pJEG011 | < 1.0E-150 | 99.49 | 390 | 1 |
| NC_017472.1 | Lactobacillus amylovorus GRL1118 | 2 | 2.0E-121 | 96.95 | 459 | 2 |
| NC_021504.1 | Lactobacillus reuteri I5007 | pLRI04 | 5.0E-125 | 96.96 | 263 | 1 |
| NC_003101.1 | Lactococcus lactis CRL1127 | pCRL1127 | < 1.0E-150 | 99.21 | 254 | 1 |
| NC_019350.1 | Lactococcus lactis subsp. cremoris | pAF14 | 2.0E-62 | 98.94 | 567 | 1 |
| NC_017484.1 | Lactococcus lactis subsp. lactis CV56 | pCV56C | < 1.0E-150 | 98.57 | 140 | 1 |
| NC_003383.1 | Listeria innocua Clip11262 | pLI100 | < 1.0E-150 | 99.44 | 709 | 1 |
| NC_011996.1 | Macrococcus caseolyticus JCSC5402 | pMCCL2 | < 1.0E-150 | 99.75 | 399 | 2 |
| NC_012811.1 | Methylobacterium extorquens AM1 mega |  | 3.0E-124 | 99.29 | 424 | 1 |
| NC_004954.1 | Micrococcus sp. 28 | pSD10 | < 1.0E-150 | 96.03 | 277 | 1 |
| NC_009669.1 | Ochrobactrum anthropi ATCC 49188 | pOANT01 | < 1.0E-150 | 98.43 | 1082 | 1 |
| NC_009753.1 | Paracoccus methylutens strain DM12 | pMTH1 | 2.0E-41 | 99.48 | 385 | 1 |
| NC_012216.1 | Pasteurella multocida | pB1006 | < 1.0E-150 | 99 | 100 | 1 |
| NC_010864.1 | Pediococcus acidilactici | pEOC01 | < 1.0E-150 | 99.46 | 371 | 2 |
| NC_008608.1 | Pelobacter propionicus DSM 2379 | pPRO2 | < 1.0E-150 | 95.42 | 349 | 2 |
| NC_016974.1 | Providencia stuartii | pMR0211 | < 1.0E-150 | 97.81 | 320 | 1 |
| NC_022344.1 | Pseudomonas aeruginosa | pOZ176 | < 1.0E-150 | 96.83 | 442 | 1 |
| NC_003350.1 | Pseudomonas putida | pWW0 | < 1.0E-150 | 100 | 424 | 1 |
| NC_001398.1 | Saccharomyces cerevisiae A364A 2 |  | < 1.0E-150 | 96.78 | 363 | 3 |
| NC_003384.1 | Salmonella enterica subsp. enterica serovar Typhi str. CT18 | pHCM1 | < 1.0E-150 | 99.81 | 533 | 1 |
| NC_016825.1 | Salmonella enterica subsp. enterica serovar Typhi str. P-stx-12 | unnamed | 1.0E-145 | 99.3 | 711 | 1 |
| NC_008573.1 | Shewanella sp. ANA-3 | 1 | 9.0E-145 | 98.67 | 300 | 4 |
| NC_002698.1 | Shigella flexneri 5a virulence | pWR501 | < 1.0E-150 | 96.52 | 316 | 2 |
| NC_017958.1 | Tistrella mobilis KA081020-065 | pTM3 | 5.0E-104 | 95.19 | 346 | 7 |
| NC_019218.1 | Uncultured bacterium HHV35 | pHHV35 | < 1.0E-150 | 99.53 | 215 | 3 |
| NC_008055.1 | Uncultured bacterium IncP-1gamma | QKH54 | 1.0E-130 | 97.53 | 403 | 2 |
| NC_013280.1 | Uncultured bacterium | pTRACA22 | 2.0E-36 | 96.28 | 266 | 7 |
| NC_019324.1 | UNVERIFIED: Clostridium sp. MT351 | unnamed | 6.0E-92 | 95.79 | 95 | 1 |
| NC_009717.1 | Xanthobacter autotrophicus Py2 | pXAUT01 | < 1.0E-150 | 100 | 191 | 2 |

**Assigned high-throughput reads of sample C1755 against the RefSeq plasmid database**

(Sorted by Organism)

| **Accession number** | **Organism** | **Plasmid** | **E value ≤** | **Identity (%) ≥** | **Hit length (bp) ≥** | **Number of reads** |
| --- | --- | --- | --- | --- | --- | --- |
| NC_021976.1 | Acetobacter pasteurianus 386B | Apa386Bp1 | 4.0E-37 | 96.04 | 101 | 2 |
| NC_021992.1 | Acetobacter pasteurianus 386B | Apa386Bp3 | 5.0E-36 | 95 | 100 | 2 |
| NC_005793.2 | Achromobacter denitrificans | pEST4011 | 1.0E-32 | 95.05 | 90 | 429 |
| NC_014641.1 | Achromobacter xylosoxidans A8 | pA81 | 9.0E-36 | 95.05 | 96 | 29 |
| NC_006830.1 | Achromobacter xylosoxidans A8 | pA81 | 9.0E-36 | 95.05 | 101 | 3 |
| NC_022242.1 | Achromobacter xylosoxidans subsp. denitrificans | pAX22 | 2.0E-32 | 95 | 91 | 55 |
| NC_009467.1 | Acidiphilium cryptum JF-5 | pACRY01 | 2.0E-35 | 95.05 | 101 | 1 |
| NC_015852.1 | Acidithiobacillus caldus SM-1 | pLAtc1 | 4.0E-35 | 95.05 | 91 | 27 |
| NC_015854.1 | Acidithiobacillus caldus SM-1 | pLAtc3 | 3.0E-36 | 95.05 | 99 | 11 |
| NC_010600.1 | Acidithiobacillus caldus strain MNG | pTcM1 | 2.0E-35 | 95 | 90 | 17 |
| NC_005023.1 | Acidithiobacillus ferrooxidans | pTF5 | 1.0E-37 | 96 | 100 | 1 |
| NC_008765.1 | Acidovorax sp. JS42 | pAOVO01 | 6.0E-36 | 95.05 | 90 | 15 |
| NC_008766.1 | Acidovorax sp. JS42 | pAOVO02 | 2.0E-35 | 95 | 92 | 9 |
| NC_022565.1 | Acinetobacter baumannii 107m | p1ABIBUN | 7.0E-38 | 96 | 100 | 6 |
| NC_017163.1 | Acinetobacter baumannii 1656-2 | ABKp1 | 2.0E-35 | 95 | 90 | 91 |
| NC_017164.1 | Acinetobacter baumannii 1656-2 | ABKp2 | 5.0E-33 | 95 | 90 | 22 |
| NC_006877.1 | Acinetobacter baumannii 19606 | pMAC | 4.0E-35 | 95.79 | 95 | 12 |
| NC_010605.1 | Acinetobacter baumannii ACICU | pACICU1 | 6.0E-32 | 95 | 90 | 178 |
| NC_009083.1 | Acinetobacter baumannii ATCC 17978 | pAB1 | 1.0E-36 | 95.05 | 99 | 18 |
| NC_009084.1 | Acinetobacter baumannii ATCC 17978 | pAB2 | 2.0E-34 | 95.05 | 92 | 29 |
| NC_010401.1 | Acinetobacter baumannii AYE | p1ABAYE | 5.0E-37 | 95.05 | 90 | 78 |
| NC_010402.1 | Acinetobacter baumannii AYE | p2ABAYE | 8.0E-44 | 100 | 101 | 7 |
| NC_010404.1 | Acinetobacter baumannii AYE | p3ABAYE | 2.0E-30 | 95 | 90 | 586 |
| NC_021727.1 | Acinetobacter baumannii BJAB07104 | p1BJAB07104 | 6.0E-36 | 95.05 | 90 | 64 |
| NC_021728.1 | Acinetobacter baumannii BJAB07104 | p2BJAB07104 | 1.0E-38 | 98.99 | 92 | 64 |
| NC_021734.1 | Acinetobacter baumannii BJAB0715 | pBJAB0715 | 2.0E-33 | 95 | 90 | 538 |
| NC_021730.1 | Acinetobacter baumannii BJAB0868 | p1BJAB0868 | 1.0E-33 | 96.67 | 90 | 1 |
| NC_020525.1 | Acinetobacter baumannii D1279779 | pD1279779 | 7.0E-37 | 95.05 | 99 | 10 |
| NC_017848.1 | Acinetobacter baumannii MDR-TJ | pABTJ1 | 2.0E-35 | 95.05 | 90 | 97 |
| NC_020524.1 | Acinetobacter baumannii MDR-TJ | pABTJ2 | 1.0E-35 | 95.05 | 91 | 63 |
| NC_017172.1 | Acinetobacter baumannii MDR-ZJ06 | pMDR-ZJ06 | 8.0E-41 | 98.02 | 101 | 2 |
| NC_010481.1 | Acinetobacter baumannii | pABIR | 3.0E-35 | 95 | 90 | 173 |
| NC_021489.1 | Acinetobacter baumannii | pAB-NCGM253 | 1.0E-35 | 95.05 | 95 | 10 |
| NC_012813.1 | Acinetobacter baumannii | pABVA01 | 8.0E-37 | 95.05 | 101 | 4 |
| NC_013277.1 | Acinetobacter baumannii | pMMA2 | 1.0E-36 | 95.05 | 97 | 27 |
| NC_013506.1 | Acinetobacter baumannii | pMMCU2 | 1.0E-34 | 95 | 92 | 34 |
| NC_019199.1 | Acinetobacter baumannii | pMMCU3 | 2.0E-38 | 96.04 | 101 | 8 |
| NC_019280.1 | Acinetobacter baumannii | pMMD | 4.0E-35 | 95.05 | 90 | 94 |
| NC_019345.1 | Acinetobacter baumannii | pRAY*-v2 | 2.0E-32 | 95.05 | 90 | 102 |
| NC_016977.1 | Acinetobacter baumannii | pTS236 | 7.0E-37 | 95 | 100 | 3 |
| NC_010396.1 | Acinetobacter baumannii SDF | p2ABSDF | 1.0E-34 | 95 | 94 | 68 |
| NC_010398.1 | Acinetobacter baumannii SDF | p3ABSDF | 5.0E-32 | 95 | 90 | 83 |
| NC_020818.1 | Acinetobacter baumannii strain GF216 | pNDM-AB | 2.0E-33 | 96.04 | 91 | 111 |
| NC_019985.2 | Acinetobacter baumannii strain ZW85-1 | pAbNDM-1 | 2.0E-40 | 98.02 | 101 | 12 |
| NC_017166.1 | Acinetobacter baumannii TCDC-AB0715 | p2ABTCDC0715 | 2.0E-35 | 98.89 | 90 | 8 |
| NC_023031.1 | Acinetobacter baumannii ZW85-1 | ZW85p2 | 1.0E-33 | 95 | 92 | 144 |
| NC_023322.1 | Acinetobacter bereziniae strain CHI-40-1 | pNDM-BJ01 | 1.0E-35 | 95.05 | 90 | 39 |
| NC_013056.1 | Acinetobacter calcoaceticus strain Acal H12O-07 | pMMCU1 | 2.0E-38 | 96.04 | 101 | 2 |
| NC_019323.1 | Acinetobacter lwoffii | pABZ78 | 4.0E-37 | 95.05 | 101 | 2 |
| NC_019268.1 | Acinetobacter lwoffii | pNDM-BJ01 | 4.0E-43 | 100 | 101 | 2 |
| NC_023280.1 | Acinetobacter nosocomialis | pRAY*-v3 | 6.0E-37 | 95.05 | 96 | 9 |
| NC_019322.1 | Acinetobacter pittii | pABCA95 | 9.0E-33 | 96.04 | 90 | 33 |
| NC_010309.1 | Acinetobacter venetianus strain VE-C3 | pAV1 | 2.0E-32 | 95 | 92 | 101 |
| NC_010310.1 | Acinetobacter venetianus strain VE-C3 | pAV2 | 2.0E-34 | 95 | 90 | 567 |
| NC_007099.1 | Actinobacillus pleuropneumoniae | pPSAS1522 | 3.0E-44 | 100 | 101 | 3 |
| NC_007097.1 | Actinobacillus porcitonsillarum | pKMA757 | 2.0E-41 | 98.02 | 101 | 1 |
| NC_009476.1 | Aeromonas bestiarum 5S9 | pAb5S9 | 2.0E-43 | 100 | 101 | 1 |
| NC_006143.1 | Aeromonas caviae | pFBAOT6 | 5.0E-32 | 95 | 90 | 973 |
| NC_019262.1 | Aeromonas hydrophila | pAHH04 | 2.0E-42 | 99.01 | 101 | 2 |
| NC_019380.1 | Aeromonas hydrophila | pR148 | 7.0E-33 | 96.04 | 91 | 3 |
| NC_003124.1 | Aeromonas salmonicida | pRAS3.2 | 4.0E-36 | 95 | 99 | 33 |
| NC_004923.1 | Aeromonas salmonicida salmonicida A449 | pAsa1 | 2.0E-42 | 99.01 | 101 | 1 |
| NC_004925.1 | Aeromonas salmonicida salmonicida A449 | pAsa2 | 5.0E-37 | 95.05 | 101 | 1 |
| NC_004924.1 | Aeromonas salmonicida salmonicida A449 | pAsa3 | 2.0E-42 | 99.01 | 101 | 1 |
| NC_009349.1 | Aeromonas salmonicida subsp. salmonicida A449 | 4 | 7.0E-33 | 95.05 | 93 | 22 |
| NC_009350.1 | Aeromonas salmonicida subsp. salmonicida A449 | 5 | 5.0E-35 | 95 | 94 | 20 |
| NC_004338.1 | Aeromonas salmonicida subsp. salmonicida | pAsal1 | 1.0E-39 | 97.03 | 101 | 1 |
| NC_004339.1 | Aeromonas salmonicida subsp. Salmonicida | pAsal2 | 1.0E-38 | 96.04 | 101 | 2 |
| NC_004340.1 | Aeromonas salmonicida subsp. salmonicida | pAsal3 | 3.0E-39 | 97 | 100 | 1 |
| NC_009352.2 | Aeromonas salmonicida subsp. salmonicida | pAsa6 | 3.0E-39 | 97.03 | 101 | 3 |
| NC_003123.1 | Aeromonas salmonicida subsp. salmonicida | pRAS3.1 | 2.0E-34 | 95.05 | 90 | 67 |
| NC_002579.1 | Aggregatibacter actinomycetemcomitans | pVT745 | 1.0E-33 | 95.7 | 93 | 2 |
| NC_003064.2 | Agrobacterium fabrum str. C58 | At | 9.0E-38 | 97.03 | 101 | 1 |
| NC_011994.1 | Agrobacterium radiobacter K84 | pAgK84 | 1.0E-36 | 96.04 | 97 | 2 |
| NC_011990.1 | Agrobacterium radiobacter K84 | pAtK84b | 5.0E-35 | 95 | 100 | 2 |
| NC_002575.1 | Agrobacterium rhizogenes | pRi1724 DNA | 2.0E-35 | 95.05 | 101 | 1 |
| NC_010841.1 | Agrobacterium rhizogenes | pRi2659 | 4.0E-37 | 96.04 | 101 | 1 |
| NC_015184.1 | Agrobacterium sp. H13-3 | pAspH13-3a | 5.0E-35 | 95.05 | 100 | 5 |
| NC_006277.2 | Agrobacterium tumefaciens K84 | pAgK84 | 4.0E-36 | 95.05 | 101 | 1 |
| NC_002147.1 | Agrobacterium tumefaciens MAFF301001 | pTi-SAKURA | 2.0E-35 | 95.05 | 101 | 5 |
| NC_019555.1 | Agrobacterium tumefaciens | pAoF64/95 | 5.0E-35 | 95 | 98 | 3 |
| NC_002377.1 | Agrobacterium tumefaciens | Ti | 3.0E-38 | 97.03 | 101 | 1 |
| NC_010929.1 | Agrobacterium tumefaciens Ti | pTiBo542 | 2.0E-35 | 95.05 | 101 | 3 |
| NC_011986.1 | Agrobacterium vitis S4 | pAtS4a | 2.0E-31 | 95.05 | 90 | 21 |
| NC_011991.1 | Agrobacterium vitis S4 | pAtS4b | 6.0E-33 | 95.05 | 90 | 6 |
| NC_011984.1 | Agrobacterium vitis S4 | pAtS4c | 8.0E-34 | 95.05 | 93 | 7 |
| NC_011982.1 | Agrobacterium vitis S4 | pTiS4 | 8.0E-35 | 95.05 | 97 | 25 |
| NC_014908.1 | Alicycliphilus denitrificans BC | pALIDE01 | 2.0E-31 | 95 | 90 | 91 |
| NC_014911.1 | Alicycliphilus denitrificans BC | pALIDE02 | 8.0E-35 | 95 | 90 | 171 |
| NC_015423.1 | Alicycliphilus denitrificans K601 | pALIDE201 | 1.0E-31 | 95 | 90 | 28 |
| NC_021709.1 | Alteromonas macleodii str. 'English Channel 615' |  | 6.0E-41 | 99.01 | 101 | 14 |
| NC_013164.1 | Anaerococcus prevotii DSM 20548 | pAPRE01 | 1.0E-35 | 95.05 | 100 | 7 |
| NC_008712.1 | Arthrobacter aurescens TC1 | TC1 | 3.0E-35 | 95.05 | 101 | 1 |
| NC_006823.1 | Azoarcus sp. EbN1 | 1 | 1.0E-31 | 95.05 | 90 | 139 |
| NC_006824.1 | Azoarcus sp. EbN1 | 2 | 2.0E-35 | 95.05 | 100 | 9 |
| NC_020548.1 | Azoarcus sp. KH32C | pAZKH | 6.0E-35 | 95.05 | 101 | 4 |
| NC_016594.1 | Azospirillum brasilense Sp245 | AZOBR_p1 | 3.0E-30 | 95.05 | 90 | 2 |
| NC_016596.1 | Azospirillum brasilense Sp245 | AZOBR_p4 | 6.0E-35 | 95.05 | 96 | 9 |
| NC_016585.1 | Azospirillum lipoferum 4B | AZO_p1 | 2.0E-36 | 96.04 | 101 | 5 |
| NC_016623.1 | Azospirillum lipoferum 4B | AZO_p3 | 3.0E-31 | 95.6 | 91 | 2 |
| NC_016624.1 | Azospirillum lipoferum 4B | AZO_p5 | 7.0E-32 | 95.05 | 92 | 32 |
| NC_013855.1 | Azospirillum sp. B510 | pAB510a | 3.0E-30 | 95.56 | 90 | 1 |
| NC_013856.1 | Azospirillum sp. B510 | pAB510b | 2.0E-34 | 95 | 100 | 3 |
| NC_013857.1 | Azospirillum sp. B510 | pAB510c | 6.0E-35 | 95.05 | 101 | 1 |
| NC_013859.1 | Azospirillum sp. B510 | pAB510e | 5.0E-35 | 95.05 | 97 | 12 |
| NC_011771.1 | Bacillus cereus AH820 | pAH820_10 | 9.0E-44 | 100 | 101 | 2 |
| NC_018492.1 | Bacillus cereus FRI-35 | p01 | 7.0E-35 | 96.04 | 101 | 6 |
| NC_016792.1 | Bacillus cereus NC7401 | pNCcld | 5.0E-37 | 96.97 | 99 | 2 |
| NC_011973.1 | Bacillus cereus Q1 | pBc239 | 2.0E-42 | 100 | 101 | 1 |
| NC_004604.2 | Bacillus megaterium QM B1551 | pBM400 | 5.0E-30 | 95 | 90 | 778 |
| NC_017139.1 | Bacillus megaterium WSH-002 | WSH-002_p1 | 1.0E-31 | 95 | 90 | 89 |
| NC_013792.1 | Bacillus pseudofirmus OF4 | pBpOF4-01 | 2.0E-35 | 95.05 | 101 | 3 |
| NC_013963.1 | Bacillus sp. BS-01 | pBS-01 | 4.0E-37 | 98.02 | 90 | 599 |
| NC_014557.1 | Bacillus sp. BS-02 | pBS-02 | 6.0E-42 | 99.01 | 101 | 19 |
| NC_014937.1 | Bacillus thuringiensis CT43 | pBMB0558 | 4.0E-34 | 95.05 | 90 | 23 |
| NC_023074.1 | Bacillus thuringiensis serovar tenebrionis str. YBT-1765 | pBMB165 | 3.0E-41 | 99.01 | 101 | 1 |
| NC_019783.1 | Bacterium 36B | pTOR_02 | 3.0E-35 | 95.05 | 91 | 46 |
| NC_019798.1 | Bacterium 72B | pTOR_01 | 7.0E-42 | 99.01 | 101 | 8 |
| NC_006873.1 | Bacteroides fragilis NCTC 9343 | pBF9343 | 3.0E-36 | 95.05 | 98 | 31 |
| NC_011073.1 | Bacteroides fragilis | pBFP35 | 6.0E-43 | 100 | 99 | 3 |
| NC_019534.1 | Bacteroides fragilis | pBFUK1 | 1.0E-36 | 95.05 | 92 | 27 |
| NC_015166.1 | Bacteroides salanitronis DSM 18170 | pBACSA03 | 3.0E-41 | 98.02 | 101 | 1 |
| NC_004703.1 | Bacteroides thetaiotaomicron VPI-5482 | p5482 | 8.0E-37 | 95.96 | 99 | 5 |
| NC_007068.1 | Bifidobacterium catenulatum | pBC1 | 2.0E-44 | 100 | 101 | 2 |
| NC_021875.1 | Bifidobacterium kashiwanohense JCM 15439 | pBBKW-1 DNA | 3.0E-42 | 99.01 | 101 | 4 |
| NC_021876.1 | Bifidobacterium kashiwanohense JCM 15439 | pBBKW-2 DNA | 8.0E-38 | 95.96 | 99 | 2 |
| NC_004253.1 | Bifidobacterium longum DJO10A | pDOJH10S | 3.0E-44 | 100 | 101 | 1 |
| NC_002635.1 | Bifidobacterium longum KJ | pKJ36 | 4.0E-42 | 100 | 97 | 1 |
| NC_004978.1 | Bifidobacterium longum KJ | pKJ50 | 2.0E-42 | 99.01 | 101 | 6 |
| NC_010857.1 | Bifidobacterium longum | p6043A | 4.0E-44 | 100 | 101 | 6 |
| NC_011139.1 | Bifidobacterium longum | pFI2576 | 5.0E-39 | 96.04 | 101 | 12 |
| NC_006997.1 | Bifidobacterium longum | pMG1 | 1.0E-42 | 99.01 | 101 | 4 |
| NC_019200.1 | Bifidobacterium longum | pSP02 | 2.0E-41 | 98.02 | 101 | 4 |
| NC_006843.1 | Bifidobacterium longum | pTB6 | 3.0E-44 | 100 | 101 | 1 |
| NC_004768.1 | Bifidobacterium longum RW041 | pNAC3 | 9.0E-37 | 95.05 | 100 | 23 |
| NC_004770.1 | Bifidobacterium longum RW048 | pNAC1 | 6.0E-40 | 98.02 | 93 | 11 |
| NC_015053.1 | Bifidobacterium longum subsp. infantis 157F | p157F-NC1 | 9.0E-40 | 97.03 | 101 | 46 |
| NC_015066.1 | Bifidobacterium longum subsp. infantis 157F | p157F-NC2 | 2.0E-41 | 98.02 | 101 | 2 |
| NC_004443.1 | Bifidobacterium longum VMKB44 | pB44 | 3.0E-44 | 100 | 101 | 1 |
| NC_008459.1 | Bordetella pertussis | pBP136 DNA | 7.0E-39 | 97.03 | 95 | 9 |
| NC_009475.1 | Bradyrhizobium sp. BTAi1 | pBBta01 | 7.0E-35 | 95.05 | 95 | 2 |
| NC_022590.1 | Brevibacterium sp. Ap13 | pAP13 | 3.0E-41 | 99.01 | 101 | 2 |
| NC_008545.1 | Burkholderia cenocepacia HI2424 | 1 | 3.0E-37 | 96.04 | 101 | 1 |
| NC_008385.1 | Burkholderia cepacia AMMD | 1 | 4.0E-36 | 95.05 | 90 | 993 |
| NC_019378.1 | Burkholderia cepacia | pIJB1 | 1.0E-33 | 95.05 | 91 | 140 |
| NC_019369.1 | Burkholderia cepacia | pYS1 | 7.0E-36 | 95.05 | 91 | 84 |
| NC_022995.1 | Burkholderia sp. M701 | pM7012 DNA | 3.0E-32 | 95 | 90 | 35 |
| NC_016626.1 | Burkholderia sp. YI23 | byi_1p | 2.0E-34 | 95.05 | 101 | 2 |
| NC_016591.1 | Burkholderia sp. YI23 | byi_2p | 4.0E-33 | 95.74 | 94 | 16 |
| NC_009227.1 | Burkholderia vietnamiensis G4 | pBVIE02 | 2.0E-35 | 95.05 | 92 | 55 |
| NC_009229.1 | Burkholderia vietnamiensis G4 | pBVIE03 | 2.0E-35 | 95.05 | 99 | 11 |
| NC_022355.1 | Campylobacter coli CVM N29710 | pN29710-1 | 5.0E-36 | 95.05 | 93 | 15 |
| NC_006134.1 | Campylobacter coli | pCC31 | 9.0E-38 | 96.04 | 95 | 19 |
| NC_022354.1 | Campylobacter jejuni subsp. jejuni 00-2544 |  | 8.0E-39 | 97.03 | 101 | 9 |
| NC_006135.1 | Campylobacter jejuni subsp. jejuni 81-176 | pTet | 2.0E-40 | 98.02 | 99 | 6 |
| NC_007141.1 | Campylobacter jejuni subsp. jejuni 81-176 | pTet | 9.0E-38 | 96.04 | 99 | 8 |
| NC_014801.1 | Campylobacter jejuni subsp. jejuni ICDCCJ07001 | pTet | 1.0E-36 | 95.96 | 99 | 11 |
| NC_017282.1 | Campylobacter jejuni subsp. jejuni S3 | pTet | 3.0E-43 | 100 | 101 | 14 |
| NC_013193.1 | Candidatus Accumulibacter phosphatis clade IIA str. UW-1 | pAph01 | 1.0E-35 | 95.05 | 99 | 6 |
| NC_013190.1 | Candidatus Accumulibacter phosphatis clade IIA str. UW-1 | pAph02 | 1.0E-41 | 99.01 | 101 | 28 |
| NC_013191.1 | Candidatus Accumulibacter phosphatis clade IIA str. UW-1 | pAph03 | 1.0E-34 | 96.04 | 90 | 55 |
| NC_015390.1 | Carnobacterium sp. 17-4 | pCAR50 | 5.0E-36 | 95.05 | 99 | 11 |
| NC_022601.1 | Carnobacterium sp. WN1359 | pWNCR12 | 1.0E-36 | 95.96 | 93 | 22 |
| NC_022607.1 | Carnobacterium sp. WN1359 | pWNCR15 | 2.0E-35 | 95.83 | 96 | 2 |
| NC_022602.1 | Carnobacterium sp. WN1359 | pWNCR47 | 2.0E-33 | 95 | 91 | 302 |
| NC_022603.1 | Carnobacterium sp. WN1359 | pWNCR64 | 2.0E-35 | 95 | 99 | 38 |
| NC_022608.1 | Carnobacterium sp. WN1359 | pWNCR9 | 3.0E-42 | 99.01 | 101 | 3 |
| NC_010333.1 | Caulobacter sp. K31 | pCAUL02 | 4.0E-37 | 96.04 | 101 | 5 |
| NC_019360.1 | Citrobacter freundii | pNDM-CIT | 6.0E-37 | 96.04 | 101 | 2 |
| NC_020122.1 | Citrobacter freundii strain CFSTE | pN-Cit | 1.0E-40 | 98.02 | 101 | 1 |
| NC_020123.1 | Citrobacter freundii strain CFSTE | pT-OXA-181 | 6.0E-43 | 100 | 101 | 6 |
| NC_019983.1 | Citrobacter freundii strain Iona 2 | pCFI-1 | 2.0E-37 | 96 | 100 | 5 |
| NC_019991.1 | Citrobacter freundii strain Iona 4 | pCFI-2 | 2.0E-43 | 100 | 101 | 1 |
| NC_019984.1 | Citrobacter freundii strain Iona 6 | pCFI-3 | 5.0E-38 | 96.04 | 101 | 2 |
| NC_013717.1 | Citrobacter rodentium ICC168 | pCROD1 | 2.0E-34 | 95.79 | 95 | 2 |
| NC_013719.1 | Citrobacter rodentium ICC168 | pCROD3 | 3.0E-44 | 100 | 101 | 1 |
| NC_003114.1 | Citrobacter rodentium strain DBS100 | pCRP3 | 3.0E-44 | 100 | 101 | 1 |
| NC_010937.1 | Clostridium perfringens | pCW3 | 1.0E-35 | 95 | 100 | 3 |
| NC_021652.1 | Clostridium thermocellum strain BL21 | pEBM107 | 3.0E-42 | 99.01 | 101 | 2 |
| NC_010332.1 | Collimonas fungivorans | pTer331 | 2.0E-32 | 95.05 | 91 | 11 |
| NC_021077.1 | Comamonas sp. 7D-2 | pBHB | 7.0E-32 | 95 | 90 | 91 |
| NC_010935.1 | Comamonas testosteroni CNB-1 | pCNB | 5.0E-32 | 95 | 90 | 649 |
| NC_016978.1 | Comamonas testosteroni | pI2 | 3.0E-34 | 95 | 90 | 22 |
| NC_002143.1 | Comamonas testosteroni PtL5 cryptic | pPT1 | 7.0E-34 | 95 | 92 | 86 |
| NC_003227.1 | Corynebacterium glutamicum | pTET3 | 3.0E-35 | 97.8 | 91 | 9 |
| NC_001791.1 | Corynebacterium glutamicum strain 1014 | pXZ10145.1 | 9.0E-40 | 97.03 | 101 | 1 |
| NC_004945.1 | Corynebacterium glutamicum strain ATCC31830 R- | pCG4 | 2.0E-43 | 100 | 101 | 4 |
| NC_003490.1 | Corynebacterium jeikeium | pB85766 | 1.0E-43 | 100 | 101 | 1 |
| NC_014167.1 | Corynebacterium resistens DSM 45100 | pJA144188 | 3.0E-35 | 95.05 | 95 | 22 |
| NC_004939.1 | Corynebacterium striatum strain M82B R- | pTP10 | 4.0E-43 | 100 | 101 | 1 |
| NC_009779.1 | Cronobacter sakazakii ATCC BAA-894 | pESA2 | 5.0E-33 | 95.05 | 90 | 303 |
| NC_023025.1 | Cronobacter sakazakii CMCC 45402 | p2 | 5.0E-36 | 95.05 | 101 | 4 |
| NC_020261.1 | Cronobacter sakazakii Sp291 | pSP291-2 | 9.0E-39 | 97.03 | 101 | 3 |
| NC_021293.1 | Cronobacter sakazakii strain ATCC 29544 | pCSA2 | 1.0E-38 | 96.04 | 101 | 2 |
| NC_007974.2 | Cupriavidus metallidurans CH34 |  | 1.0E-30 | 95 | 90 | 281 |
| NC_006525.1 | Cupriavidus metallidurans CH34 | pMOL28 | 2.0E-34 | 95.05 | 96 | 29 |
| NC_015727.1 | Cupriavidus necator N-1 | BB1p | 8.0E-31 | 95 | 91 | 40 |
| NC_012527.1 | Deinococcus deserti VCD115 | 1 | 6.0E-31 | 95.05 | 90 | 26 |
| NC_008010.2 | Deinococcus geothermalis DSM 11300 | pDGEO01 | 1.0E-30 | 95.56 | 90 | 16 |
| NC_019789.1 | Deinococcus peraridilitoris DSM 19664 | pDEIPE01 | 1.0E-30 | 95 | 90 | 64 |
| NC_015162.1 | Deinococcus proteolyticus MRP | pDEIPR02 | 1.0E-42 | 100 | 101 | 2 |
| NC_015163.1 | Deinococcus proteolyticus MRP | pDEIPR04 | 3.0E-41 | 99.01 | 101 | 3 |
| NC_000959.1 | Deinococcus radiodurans R1 | CP1 | 8.0E-39 | 97.03 | 101 | 1 |
| NC_000958.1 | Deinococcus radiodurans R1 | MP1 | 5.0E-35 | 95 | 100 | 2 |
| NC_005088.1 | Delftia acidovorans B | pUO1 | 4.0E-32 | 95 | 91 | 185 |
| NC_019283.1 | Delftia acidovorans | pC1-1 | 1.0E-38 | 97.03 | 100 | 4 |
| NC_019263.1 | Delftia acidovorans | pLME1 | 6.0E-43 | 100 | 101 | 1 |
| NC_019264.1 | Delftia acidovorans | pNB8c | 5.0E-36 | 95.05 | 90 | 30 |
| NC_019312.1 | Delftia sp. KV29 | pKV29 | 1.0E-31 | 95 | 90 | 2973 |
| NC_009955.1 | Dinoroseobacter shibae DFL 12 | pDSHI01 | 2.0E-35 | 95.05 | 101 | 6 |
| NC_009957.1 | Dinoroseobacter shibae DFL 12 | pDSHI03 | 1.0E-35 | 95.05 | 95 | 42 |
| NC_009958.1 | Dinoroseobacter shibae DFL 12 | pDSHI04 | 7.0E-43 | 100 | 101 | 4 |
| NC_020280.1 | Edwardsiella ictaluri | pEI3 | 6.0E-36 | 95.05 | 91 | 22 |
| NC_014725.1 | Edwardsiella tarda strain CK41 | pCK41 | 2.0E-35 | 95.05 | 95 | 13 |
| NC_001735.4 | Enterobacter aerogenes | R751 | 2.0E-41 | 99.01 | 101 | 15 |
| NC_012555.1 | Enterobacter cloacae | pEC-IMP | 6.0E-37 | 96.04 | 93 | 6 |
| NC_012556.1 | Enterobacter cloacae | pEC-IMPQ | 8.0E-36 | 96.97 | 92 | 50 |
| NC_019368.1 | Enterobacter cloacae | pEl1573 | 3.0E-40 | 98.02 | 101 | 5 |
| NC_019346.1 | Enterobacter cloacae | pNE1280 | 6.0E-36 | 95.05 | 99 | 3 |
| NC_015175.1 | Enterobacter cloacae | pS51A | 2.0E-41 | 98.02 | 101 | 1 |
| NC_019242.1 | Enterobacter cloacae | pS51B | 4.0E-44 | 100 | 101 | 1 |
| NC_017097.1 | Enterobacter cloacae | pUL3AT | 8.0E-37 | 95.05 | 92 | 19 |
| NC_021087.1 | Enterobacter cloacae strain M15 |  | 4.0E-33 | 95.05 | 90 | 12 |
| NC_014107.1 | Enterobacter cloacae subsp. cloacae ATCC 13047 | pECL_A | 6.0E-35 | 95 | 99 | 38 |
| NC_021492.1 | Enterobacter sp. R4-368 | pENT01 | 2.0E-38 | 97.03 | 101 | 2 |
| NC_015515.1 | Enterobacter sp. W001 | pR23 | 8.0E-44 | 100 | 101 | 1 |
| NC_017314.1 | Enterococcus faecalis 62 | EF62pA | 4.0E-44 | 100 | 101 | 2 |
| NC_017315.1 | Enterococcus faecalis 62 | EF62pC | 4.0E-43 | 100 | 101 | 1 |
| NC_018222.1 | Enterococcus faecalis D32 | EFD32pA | 1.0E-43 | 100 | 101 | 7 |
| NC_013514.1 | Enterococcus faecalis | pAMbeta1 | 3.0E-36 | 95.05 | 97 | 38 |
| NC_006827.2 | Enterococcus faecalis | pCF10 | 3.0E-39 | 97.09 | 101 | 32 |
| NC_014508.2 | Enterococcus faecalis | pEF-01 | 5.0E-33 | 96.04 | 90 | 66 |
| NC_014726.1 | Enterococcus faecalis | pTW9 | 2.0E-36 | 96.04 | 92 | 26 |
| NC_014475.1 | Enterococcus faecalis | pWZ1668 | 4.0E-36 | 95.05 | 92 | 81 |
| NC_019213.1 | Enterococcus faecalis | pWZ909 | 4.0E-36 | 95.05 | 91 | 12 |
| NC_008445.1 | Enterococcus faecalis RE25 | pRE25 | 4.0E-37 | 96 | 100 | 16 |
| NC_004669.1 | Enterococcus faecalis V583 | pTEF1 | 5.0E-37 | 99.01 | 90 | 19 |
| NC_004671.1 | Enterococcus faecalis V583 | pTEF2 | 2.0E-41 | 99.01 | 101 | 1 |
| NC_004670.1 | Enterococcus faecalis V583 | pTEF3 | 3.0E-39 | 97.03 | 101 | 7 |
| NC_017032.1 | Enterococcus faecium Aus0004 | AUS0004_p1 | 5.0E-36 | 95.05 | 99 | 2 |
| NC_021987.1 | Enterococcus faecium Aus0085 | p1 | 1.0E-35 | 95.05 | 97 | 32 |
| NC_021995.1 | Enterococcus faecium Aus0085 | p2 | 1.0E-37 | 96.04 | 101 | 4 |
| NC_021988.1 | Enterococcus faecium Aus0085 | p3 | 1.0E-33 | 95 | 95 | 53 |
| NC_017961.1 | Enterococcus faecium DO | 1 | 3.0E-37 | 96.04 | 90 | 52 |
| NC_017962.1 | Enterococcus faecium DO | 2 | 1.0E-31 | 95.79 | 90 | 5 |
| NC_017963.1 | Enterococcus faecium DO | 3 | 2.0E-35 | 95.05 | 94 | 38 |
| NC_020208.1 | Enterococcus faecium NRRL B-2354 | pNB2354_1 | 1.0E-32 | 95 | 90 | 214 |
| NC_013317.1 | Enterococcus faecium | p5753cA | 8.0E-35 | 95 | 99 | 40 |
| NC_021170.1 | Enterococcus faecium | pF856 | 2.0E-37 | 96 | 96 | 26 |
| NC_011140.1 | Enterococcus faecium | pIP816 | 7.0E-38 | 97.03 | 96 | 3 |
| NC_016009.1 | Enterococcus faecium | pM7M2 | 7.0E-42 | 99.01 | 101 | 6 |
| NC_014959.1 | Enterococcus faecium | pS177 | 1.0E-34 | 95 | 91 | 176 |
| NC_008768.1 | Enterococcus faecium | pVEF1 | 7.0E-39 | 97.03 | 97 | 5 |
| NC_008821.1 | Enterococcus faecium | pVEF2 | 7.0E-39 | 97.03 | 101 | 3 |
| NC_010980.1 | Enterococcus faecium | pVEF3 | 2.0E-34 | 95.79 | 95 | 23 |
| NC_004446.1 | Erwinia amylovora IL-5 | pEA2.8 | 1.0E-42 | 99.01 | 101 | 2 |
| NC_010719.1 | Escherichia coli 53638 | p53638_226 | 4.0E-38 | 97.03 | 101 | 5 |
| NC_010720.1 | Escherichia coli 53638 | p53638_75 | 3.0E-40 | 98.02 | 101 | 2 |
| NC_011752.1 | Escherichia coli 55989 | 55989p | 2.0E-37 | 96.04 | 101 | 1 |
| NC_007675.1 | Escherichia coli A2363 | pAPEC-O2-ColV | 2.0E-33 | 95.74 | 94 | 4 |
| NC_006671.1 | Escherichia coli A2363 | pAPEC-O2-R | 3.0E-35 | 98.89 | 90 | 10 |
| NC_023327.1 | Escherichia coli ACN001 | pACN001-B | 3.0E-37 | 96.04 | 97 | 8 |
| NC_023326.1 | Escherichia coli ACN001 | pACN001-F | 3.0E-34 | 95.79 | 95 | 1 |
| NC_009838.1 | Escherichia coli APEC O1 | pAPEC-O1-R | 3.0E-40 | 99.01 | 101 | 5 |
| NC_002142.1 | Escherichia coli B171 | pB171 | 2.0E-41 | 99.01 | 101 | 13 |
| NC_011980.1 | Escherichia coli chi7122 | pAPEC-1 | 2.0E-38 | 97.03 | 101 | 13 |
| NC_009787.1 | Escherichia coli E24377A | pETEC_35 | 7.0E-38 | 96.04 | 101 | 14 |
| NC_009788.1 | Escherichia coli E24377A | pETEC_73 | 2.0E-35 | 95.1 | 101 | 3 |
| NC_009790.1 | Escherichia coli E24377A | pETEC_74 | 2.0E-41 | 99.01 | 101 | 2 |
| NC_009786.1 | Escherichia coli E24377A | pETEC_80 | 9.0E-41 | 99 | 100 | 3 |
| NC_011754.1 | Escherichia coli ED1a | pECOED | 9.0E-43 | 100 | 101 | 1 |
| NC_007365.1 | Escherichia coli EH41 | pO113 | 1.0E-35 | 96 | 91 | 2 |
| NC_014234.1 | Escherichia coli ETEC 1392/75 | p746 | 3.0E-40 | 98.02 | 101 | 6 |
| NC_014235.1 | Escherichia coli ETEC 1392/75 | p75 | 2.0E-36 | 95.1 | 102 | 1 |
| NC_017724.1 | Escherichia coli ETEC H10407 | p948 | 7.0E-43 | 100 | 101 | 2 |
| NC_013507.1 | Escherichia coli ETEC H10407 | pEntH10407 | 5.0E-43 | 100 | 101 | 1 |
| NC_018998.1 | Escherichia coli F18+ | pTC1 | 2.0E-37 | 96.04 | 101 | 3 |
| NC_022742.1 | Escherichia coli HUSEC2011 | pHUSEC2011-1 | 2.0E-38 | 98.02 | 93 | 5 |
| NC_022743.1 | Escherichia coli HUSEC2011 | pHUSEC2011-2 | 6.0E-43 | 100 | 101 | 4 |
| NC_022649.1 | Escherichia coli JJ1886 | pJJ1886_2 | 9.0E-40 | 97.03 | 101 | 4 |
| NC_022662.1 | Escherichia coli JJ1886 | pJJ1886_3 | 5.0E-44 | 100 | 101 | 10 |
| NC_022650.1 | Escherichia coli JJ1886 | pJJ1886_4 | 5.0E-36 | 95.05 | 101 | 5 |
| NC_002483.1 | Escherichia coli K-12 | F DNA | 8.0E-43 | 100 | 101 | 5 |
| NC_002525.1 | Escherichia coli K-12 | R721 | 1.0E-38 | 97.03 | 101 | 13 |
| NC_016904.1 | Escherichia coli KO11FL | pEKO1101 | 2.0E-38 | 97.03 | 101 | 3 |
| NC_011917.1 | Escherichia coli LF82 | plLF82 | 2.0E-37 | 96.04 | 101 | 4 |
| NC_018651.1 | Escherichia coli O104:H4 str. 2009EL-2050 | p09EL50 | 8.0E-43 | 100 | 101 | 1 |
| NC_018654.1 | Escherichia coli O104:H4 str. 2009EL-2050 | pAA-09EL50 | 2.0E-37 | 97.03 | 101 | 10 |
| NC_018662.1 | Escherichia coli O104:H4 str. 2009EL-2071 | pAA-09EL71 | 2.0E-41 | 99.01 | 101 | 2 |
| NC_013370.1 | Escherichia coli O111:H- str. 11128 | pO111_2 | 3.0E-35 | 95.05 | 95 | 14 |
| NC_013366.1 | Escherichia coli O111:H- str. 11128 | pO111_3 | 2.0E-37 | 96.04 | 101 | 1 |
| NC_013368.1 | Escherichia coli O111:H- str. 11128 | pO111_5 | 1.0E-38 | 96.04 | 101 | 1 |
| NC_011603.1 | Escherichia coli O127:H6 str. E2348/69 | pMAR2 | 7.0E-43 | 100 | 101 | 1 |
| NC_019087.1 | Escherichia coli O25b:H4-ST131 str. EC958 | pKC396 | 3.0E-43 | 100 | 101 | 3 |
| NC_020271.1 | Escherichia coli O25b:H4-ST131 str. EC958 strain ST131 | pJIE186-2 | 4.0E-41 | 99.01 | 101 | 1 |
| NC_013369.1 | Escherichia coli O26:H11 str. 11368 | pO26_1 | 5.0E-38 | 97.98 | 99 | 2 |
| NC_013362.1 | Escherichia coli O26:H11 str. 11368 | pO26_2 | 5.0E-43 | 100 | 101 | 1 |
| NC_014543.1 | Escherichia coli O26:H11 str. 11368 | pO26_4 | 1.0E-42 | 99.01 | 101 | 1 |
| NC_013942.1 | Escherichia coli O55:H7 str. CB9615 | pO55 | 1.0E-37 | 96.04 | 101 | 1 |
| NC_017653.1 | Escherichia coli O55:H7 str. RM12579 | p12579_1 | 8.0E-36 | 95.05 | 97 | 11 |
| NC_017657.1 | Escherichia coli O55:H7 str. RM12579 | p12579_2 | 1.0E-37 | 96.04 | 101 | 4 |
| NC_017647.1 | Escherichia coli O7:K1 str. CE10 | pCE10A | 4.0E-43 | 100 | 101 | 1 |
| NC_017659.1 | Escherichia coli O83:H1 str. NRG 857C | pO83_CORR | 1.0E-42 | 100 | 101 | 3 |
| NC_001371.1 | Escherichia coli | ColE1 | 5.0E-44 | 100 | 101 | 2 |
| NC_014356.1 | Escherichia coli | IncQ-type pQ7 | 1.0E-40 | 99.01 | 95 | 7 |
| NC_010257.1 | Escherichia coli | MccC7-H22 | 2.0E-43 | 100 | 101 | 4 |
| NC_004998.1 | Escherichia coli | p1658/97 | 3.0E-37 | 96.04 | 97 | 4 |
| NC_015872.1 | Escherichia coli | p271A | 3.0E-43 | 100 | 101 | 1 |
| NC_014843.1 | Escherichia coli | p3521 | 2.0E-31 | 95.96 | 90 | 10 |
| NC_019094.1 | Escherichia coli | p417H-90 | 1.0E-38 | 98.96 | 96 | 5 |
| NC_019062.1 | Escherichia coli | p838C-R1 | 6.0E-38 | 96.04 | 99 | 30 |
| NC_005324.1 | Escherichia coli | p9123 | 5.0E-44 | 100 | 101 | 1 |
| NC_019050.1 | Escherichia coli | pAm05WL3325 | 3.0E-44 | 100 | 101 | 1 |
| NC_019051.1 | Escherichia coli | pAm05WL6211 | 1.0E-39 | 97.03 | 101 | 1 |
| NC_019056.1 | Escherichia coli | pAm08CD9902 | 7.0E-38 | 96.04 | 90 | 29 |
| NC_019053.1 | Escherichia coli | pAm08WL3069 | 2.0E-44 | 100 | 101 | 1 |
| NC_019091.1 | Escherichia coli | pASL01a | 9.0E-36 | 95 | 90 | 14 |
| NC_019070.1 | Escherichia coli | pCERC1 | 4.0E-39 | 100 | 92 | 3 |
| NC_019037.1 | Escherichia coli | pChi7122-2 | 6.0E-37 | 96 | 100 | 10 |
| NC_019039.1 | Escherichia coli | pChi7122-3 | 2.0E-40 | 98.02 | 101 | 15 |
| NC_019049.1 | Escherichia coli | pCM959 | 7.0E-40 | 97.03 | 95 | 22 |
| NC_007635.1 | Escherichia coli | pCoo | 9.0E-36 | 95.05 | 101 | 4 |
| NC_022333.1 | Escherichia coli | pCss165Kan DNA | 5.0E-35 | 95 | 99 | 23 |
| NC_014477.1 | Escherichia coli | pCT | 7.0E-43 | 100 | 101 | 1 |
| NC_019067.1 | Escherichia coli | pE001 | 3.0E-43 | 100 | 101 | 1 |
| NC_014382.1 | Escherichia coli | pEC_B24 | 6.0E-43 | 100 | 101 | 2 |
| NC_019083.1 | Escherichia coli | pEC14_35 | 1.0E-41 | 99.01 | 101 | 1 |
| NC_021997.1 | Escherichia coli | pEC386IL | 2.0E-40 | 98.02 | 101 | 2 |
| NC_021999.1 | Escherichia coli | pEC386IL | 2.0E-44 | 100 | 101 | 1 |
| NC_012690.1 | Escherichia coli | peH4H | 6.0E-40 | 98.02 | 101 | 24 |
| NC_013120.1 | Escherichia coli | pEK204 | 7.0E-43 | 100 | 101 | 2 |
| NC_013121.1 | Escherichia coli | pEK516 | 1.0E-37 | 96.04 | 92 | 10 |
| NC_014615.1 | Escherichia coli | pETN48 | 6.0E-34 | 95.05 | 101 | 28 |
| NC_019424.1 | Escherichia coli | pFOS-HK151325 | 5.0E-43 | 100 | 101 | 1 |
| NC_019089.1 | Escherichia coli | pGUE-NDM | 7.0E-43 | 100 | 101 | 1 |
| NC_019081.1 | Escherichia coli | pHCG11 | 3.0E-41 | 100 | 96 | 6 |
| NC_019072.1 | Escherichia coli | pHK08 | 5.0E-37 | 96.08 | 101 | 7 |
| NC_020270.1 | Escherichia coli | pHN1122-1 | 9.0E-40 | 98.98 | 98 | 5 |
| NC_019074.1 | Escherichia coli | pHNDD81-1 | 6.0E-40 | 100 | 94 | 21 |
| NC_019000.1 | Escherichia coli | pHUSEC41-2 | 6.0E-36 | 95.05 | 101 | 5 |
| NC_018997.1 | Escherichia coli | pHUSEC41-3 | 3.0E-41 | 98.02 | 101 | 2 |
| NC_005248.1 | Escherichia coli | pIGAL1 | 7.0E-44 | 100 | 101 | 2 |
| NC_009781.1 | Escherichia coli | pIGJC156 | 2.0E-42 | 99.01 | 101 | 1 |
| NC_010885.1 | Escherichia coli | pIGWZ12 | 3.0E-44 | 100 | 101 | 1 |
| NC_014231.1 | Escherichia coli | pKC394 | 2.0E-40 | 98.02 | 101 | 6 |
| NC_019097.1 | Escherichia coli | Plm | 9.0E-36 | 95.05 | 101 | 1 |
| NC_010064.1 | Escherichia coli | pLMO226 | 1.0E-39 | 100 | 92 | 7 |
| NC_019093.1 | Escherichia coli | pLST424C-61 | 8.0E-34 | 95.05 | 94 | 15 |
| NC_008487.1 | Escherichia coli | pMG828-2 | 7.0E-40 | 97.03 | 101 | 3 |
| NC_008490.1 | Escherichia coli | pMG828-5 | 8.0E-37 | 95.05 | 100 | 5 |
| NC_007682.3 | Escherichia coli | pMUR050 | 4.0E-43 | 100 | 101 | 1 |
| NC_019043.1 | Escherichia coli | pND11_107 | 8.0E-37 | 96 | 100 | 2 |
| NC_018994.1 | Escherichia coli | pNDM-1_Dok01 | 5.0E-36 | 95.96 | 92 | 13 |
| NC_019069.1 | Escherichia coli | pNDM10505 | 3.0E-31 | 95.05 | 90 | 44 |
| NC_022375.1 | Escherichia coli | pNDM-BTR | 2.0E-40 | 98.02 | 101 | 1 |
| NC_019063.1 | Escherichia coli | pNDM-HK | 7.0E-43 | 100 | 101 | 1 |
| NC_019046.1 | Escherichia coli | pNMEC31_31 | 2.0E-43 | 100 | 101 | 2 |
| NC_022992.1 | Escherichia coli | pO111-CRL-115 | 9.0E-43 | 100 | 101 | 1 |
| NC_022996.1 | Escherichia coli | pO26-CRL-125 | 9.0E-43 | 100 | 101 | 1 |
| NC_011227.1 | Escherichia coli | pO26-S3 | 3.0E-44 | 100 | 101 | 1 |
| NC_008460.1 | Escherichia coli | pO86A1 | 3.0E-36 | 98.91 | 92 | 1 |
| NC_010378.1 | Escherichia coli | pOLA52 | 5.0E-36 | 95.05 | 101 | 5 |
| NC_019065.1 | Escherichia coli | pPG010208 | 1.0E-42 | 100 | 101 | 1 |
| NC_019061.1 | Escherichia coli | pPWD4_103 | 3.0E-41 | 99.01 | 101 | 1 |
| NC_012886.1 | Escherichia coli | pRAx | 5.0E-36 | 95.05 | 101 | 14 |
| NC_009602.1 | Escherichia coli | pSFO157 | 1.0E-35 | 95.05 | 101 | 5 |
| NC_019080.1 | Escherichia coli | pT108 | 2.0E-38 | 96.04 | 101 | 3 |
| NC_008597.1 | Escherichia coli | pVI678 | 5.0E-44 | 100 | 101 | 1 |
| NC_010409.1 | Escherichia coli | pVM01 | 1.0E-42 | 100 | 101 | 3 |
| NC_011747.1 | Escherichia coli S88 | pECOS88 | 5.0E-40 | 98.02 | 101 | 3 |
| NC_011416.1 | Escherichia coli SE11 | pSE11-3 | 2.0E-40 | 98.02 | 101 | 1 |
| NC_010488.1 | Escherichia coli SMS-3-5 | pSMS35_130 | 5.0E-40 | 98.02 | 101 | 4 |
| NC_010487.1 | Escherichia coli SMS-3-5 | pSMS35_3 | 1.0E-41 | 98.02 | 101 | 1 |
| NC_020278.2 | Escherichia coli strain 3A11 | pHN3A11 | 1.0E-39 | 98 | 100 | 5 |
| NC_019990.1 | Escherichia coli strain BB1093 | pB1024 | 5.0E-44 | 100 | 101 | 1 |
| NC_023315.1 | Escherichia coli strain EQ011 | pEQ011 | 7.0E-36 | 95.05 | 99 | 6 |
| NC_022885.1 | Escherichia coli strain LK-NARMP | pKPC-LKEc | 2.0E-33 | 96.74 | 92 | 1 |
| NC_017630.1 | Escherichia coli UM146 | pUM146 | 4.0E-41 | 99.01 | 99 | 5 |
| NC_017645.1 | Escherichia coli UMNK88 | pUMNK88 | 2.0E-33 | 95.05 | 101 | 1 |
| NC_017642.1 | Escherichia coli UMNK88 | pUMNK88_91 | 7.0E-43 | 100 | 101 | 1 |
| NC_017640.1 | Escherichia coli UMNK88 | pUMNK88_Ent | 9.0E-35 | 96.81 | 94 | 1 |
| NC_017643.1 | Escherichia coli UMNK88 | pUMNK88_Hly | 1.0E-38 | 97.03 | 101 | 6 |
| NC_017639.1 | Escherichia coli UMNK88 | pUMNK88_K88 | 5.0E-38 | 97.06 | 102 | 1 |
| NC_007941.1 | Escherichia coli UTI89 | pUTI89 | 9.0E-43 | 100 | 101 | 1 |
| NC_011743.1 | Escherichia fergusonii ATCC 35469 | pEFER | 9.0E-39 | 97.03 | 101 | 5 |
| NC_009716.1 | Escherichia sp. Sflu5 cryptic | pAK51 | 3.0E-41 | 98.02 | 101 | 4 |
| NC_012782.1 | Eubacterium eligens ATCC 27750 | unnamed | 5.0E-36 | 95.05 | 95 | 41 |
| NC_012780.1 | Eubacterium eligens ATCC 27750 | unnamed | 1.0E-30 | 95 | 90 | 1413 |
| NC_010608.1 | Exiguobacterium arabatum | pEspB | 7.0E-39 | 97.03 | 101 | 1 |
| NC_023287.1 | Exiguobacterium sp. S3-2 | pMC1 | 6.0E-36 | 95.05 | 93 | 47 |
| NC_010371.1 | Finegoldia magna ATCC 29328 | pFMC | 2.0E-34 | 95.05 | 94 | 3 |
| NC_002109.1 | Francisella tularensis | pOM1 | 4.0E-44 | 100 | 101 | 1 |
| NC_018583.1 | Gordonia sp. KTR9 | pGKT3 | 4.0E-37 | 96.04 | 101 | 1 |
| NC_005307.1 | Gordonia westfalica strain DSM44215T | pKB1 | 7.0E-37 | 96.94 | 98 | 1 |
| NC_011409.1 | Haemophilus influenzae | ICEhin1056 | 5.0E-43 | 100 | 101 | 3 |
| NC_020228.1 | Haemophilus parainfluenzae strain 72322 |  | 4.0E-44 | 100 | 101 | 1 |
| NC_012661.1 | Haemophilus parasuis | pHN61 | 5.0E-44 | 100 | 101 | 3 |
| NC_014633.1 | Ilyobacter polytropus DSM 2926 | pILYOP01 | 2.0E-30 | 95 | 90 | 33 |
| NC_014621.1 | Ketogulonicigenium vulgare Y25 | pYP1 | 2.0E-30 | 95 | 91 | 108 |
| NC_017386.1 | Ketogulonigenium vulgarum WSH-001 | 1 | 8.0E-41 | 99.01 | 101 | 2 |
| NC_021501.1 | Klebsiella oxytoca E718 | pKOX_NDM1 | 4.0E-34 | 95.96 | 91 | 31 |
| NC_018107.1 | Klebsiella oxytoca E718 | pKOX_R1 | 1.0E-39 | 99.01 | 101 | 10 |
| NC_019286.1 | Klebsiella oxytoca | pINCan01 | 2.0E-36 | 95 | 90 | 35 |
| NC_005249.1 | Klebsiella pneumoniae CG43 | pLVPK | 7.0E-35 | 96.04 | 90 | 68 |
| NC_022078.1 | Klebsiella pneumoniae JM45 | p1 | 1.0E-40 | 99.01 | 101 | 6 |
| NC_017541.1 | Klebsiella pneumoniae KCTC 2242 | pKCTC2242 | 3.0E-33 | 95 | 91 | 277 |
| NC_006625.1 | Klebsiella pneumoniae NTUH-K2044 | pK2044 | 2.0E-42 | 100 | 101 | 1 |
| NC_011383.1 | Klebsiella pneumoniae | 9 | 1.0E-38 | 97.03 | 101 | 3 |
| NC_021667.1 | Klebsiella pneumoniae | IncA/C-LS6 | 3.0E-32 | 95 | 90 | 135 |
| NC_011511.1 | Klebsiella pneumoniae | p169 | 3.0E-37 | 95.05 | 101 | 40 |
| NC_011512.1 | Klebsiella pneumoniae | p9701 | 2.0E-41 | 98.02 | 101 | 3 |
| NC_022520.1 | Klebsiella pneumoniae | pBK15692 | 6.0E-37 | 97.03 | 90 | 24 |
| NC_011406.1 | Klebsiella pneumoniae | pIGMS31 | 8.0E-37 | 98.02 | 92 | 2 |
| NC_011405.1 | Klebsiella pneumoniae | pIGRK | 2.0E-44 | 100 | 101 | 2 |
| NC_019157.1 | Klebsiella pneumoniae | pIncX-SHV | 1.0E-41 | 99.01 | 101 | 1 |
| NC_020087.1 | Klebsiella pneumoniae | pK1HV | 4.0E-41 | 99.01 | 101 | 20 |
| NC_010886.1 | Klebsiella pneumoniae | pK245 | 7.0E-43 | 100 | 101 | 1 |
| NC_021622.1 | Klebsiella pneumoniae | pK45-67VIM | 4.0E-43 | 100 | 101 | 1 |
| NC_013951.1 | Klebsiella pneumoniae | pKF3-140 | 2.0E-34 | 95.05 | 96 | 13 |
| NC_013542.1 | Klebsiella pneumoniae | pKF3-70 | 2.0E-41 | 99.01 | 101 | 4 |
| NC_013950.1 | Klebsiella pneumoniae | pKF3-94 | 7.0E-43 | 100 | 101 | 2 |
| NC_002610.1 | Klebsiella pneumoniae | pKlebB-k17/80 | 4.0E-44 | 100 | 101 | 1 |
| NC_021654.1 | Klebsiella pneumoniae | pKN-LS6 | 6.0E-36 | 97.92 | 96 | 3 |
| NC_014312.1 | Klebsiella pneumoniae | pKP048 | 1.0E-42 | 100 | 101 | 1 |
| NC_021576.1 | Klebsiella pneumoniae | pKP1780 | 1.0E-37 | 98.02 | 91 | 12 |
| NC_019160.1 | Klebsiella pneumoniae | pKP3-A | 3.0E-42 | 99.01 | 101 | 4 |
| NC_021356.1 | Klebsiella pneumoniae | pKP53IL | 7.0E-38 | 99.01 | 91 | 91 |
| NC_021357.1 | Klebsiella pneumoniae | pKP53IL | 7.0E-39 | 97 | 100 | 5 |
| NC_021364.1 | Klebsiella pneumoniae | pKP53IL | 4.0E-44 | 100 | 101 | 1 |
| NC_020893.1 | Klebsiella pneumoniae | pKPC-LK30 | 3.0E-41 | 99.01 | 101 | 1 |
| NC_019161.1 | Klebsiella pneumoniae | pKPC-NY79 | 3.0E-43 | 100 | 101 | 1 |
| NC_019390.1 | Klebsiella pneumoniae | pKPN_CZ | 3.0E-32 | 95 | 91 | 229 |
| NC_019165.1 | Klebsiella pneumoniae | pKPN101-IT | 4.0E-34 | 95 | 92 | 15 |
| NC_021502.1 | Klebsiella pneumoniae | pKPoxa-48N2 | 3.0E-37 | 96.04 | 101 | 53 |
| NC_021655.1 | Klebsiella pneumoniae | pKpQIL-LS6 | 6.0E-43 | 100 | 101 | 1 |
| NC_023314.1 | Klebsiella pneumoniae | pKPS30 | 1.0E-37 | 96.04 | 101 | 2 |
| NC_019158.1 | Klebsiella pneumoniae | pNDM10469 | 3.0E-36 | 96.91 | 97 | 1 |
| NC_021180.1 | Klebsiella pneumoniae | pNDM-1saitama01 DNA | 5.0E-35 | 96.81 | 93 | 10 |
| NC_014368.1 | Klebsiella pneumoniae | pNL194 | 9.0E-41 | 99.02 | 102 | 1 |
| NC_019154.1 | Klebsiella pneumoniae | pOXA-48 | 5.0E-43 | 100 | 101 | 1 |
| NC_019152.1 | Klebsiella pneumoniae | pSLMT | 2.0E-43 | 100 | 101 | 1 |
| NC_019163.1 | Klebsiella pneumoniae | pTR4 | 3.0E-43 | 100 | 101 | 7 |
| NC_016979.1 | Klebsiella pneumoniae | pUUH239.1 | 2.0E-42 | 99.01 | 101 | 2 |
| NC_014478.1 | Klebsiella pneumoniae | unnamed | 2.0E-32 | 95 | 90 | 41 |
| NC_019889.1 | Klebsiella pneumoniae strain 601 | pNDM-OM | 1.0E-40 | 100 | 97 | 1 |
| NC_019987.1 | Klebsiella pneumoniae strain BB1089 | pB1020 | 3.0E-44 | 100 | 101 | 1 |
| NC_019989.1 | Klebsiella pneumoniae strain BB1090 | pB1021 | 1.0E-42 | 99.01 | 101 | 1 |
| NC_019888.1 | Klebsiella pneumoniae strain BK31551 | pBK31551 | 2.0E-37 | 96.04 | 91 | 4 |
| NC_019899.1 | Klebsiella pneumoniae strain BK31567 | pBK31567 | 2.0E-40 | 98.02 | 101 | 3 |
| NC_020132.1 | Klebsiella pneumoniae strain BK32179 | pBK32179 | 6.0E-40 | 98.02 | 100 | 8 |
| NC_022652.1 | Klebsiella pneumoniae strain CRE114 | pIMP-PH114 | 3.0E-38 | 97.03 | 101 | 4 |
| NC_023027.1 | Klebsiella pneumoniae strain E71T |  | 5.0E-43 | 100 | 101 | 1 |
| NC_023331.1 | Klebsiella pneumoniae strain HS062105 | pHS062105-3 | 1.0E-41 | 99.01 | 101 | 2 |
| NC_021078.1 | Klebsiella pneumoniae strain Kp002 | pJEG011 | 1.0E-32 | 95 | 93 | 119 |
| NC_021238.1 | Klebsiella pneumoniae strain Kpn-1433 | pKP1433 | 2.0E-40 | 98.02 | 97 | 4 |
| NC_023330.1 | Klebsiella pneumoniae strain KPS77 | pKPS77 | 4.0E-36 | 95.05 | 101 | 2 |
| NC_022740.1 | Klebsiella pneumoniae strain MGR-K194 | pNDM_MGR194 | 4.0E-43 | 100 | 101 | 7 |
| NC_022609.1 | Klebsiella pneumoniae strain N11-0042 | pKp11-42 | 4.0E-35 | 95.05 | 95 | 15 |
| NC_023334.1 | Klebsiella pneumoniae strain ST15 | pKP02022 | 3.0E-38 | 97.03 | 101 | 1 |
| NC_023333.1 | Klebsiella pneumoniae strain ST23 | pKP007 | 2.0E-35 | 95.05 | 100 | 7 |
| NC_023332.1 | Klebsiella pneumoniae strain ST48 | pKP09085 | 2.0E-35 | 95.05 | 97 | 24 |
| NC_016838.1 | Klebsiella pneumoniae subsp. pneumoniae HS11286 | pKPHS1 | 1.0E-35 | 95.05 | 99 | 7 |
| NC_016846.1 | Klebsiella pneumoniae subsp. pneumoniae HS11286 | pKPHS2 | 4.0E-40 | 98.02 | 101 | 2 |
| NC_016839.1 | Klebsiella pneumoniae subsp. pneumoniae HS11286 | pKPHS3 | 2.0E-37 | 96.04 | 98 | 3 |
| NC_016840.1 | Klebsiella pneumoniae subsp. pneumoniae HS11286 | pKPHS4 | 3.0E-44 | 100 | 101 | 2 |
| NC_016847.1 | Klebsiella pneumoniae subsp. pneumoniae HS11286 | pKPHS5 | 5.0E-41 | 98 | 100 | 1 |
| NC_016841.1 | Klebsiella pneumoniae subsp. pneumoniae HS11286 | pKPHS6 | 4.0E-37 | 95 | 100 | 1 |
| NC_021198.1 | Klebsiella pneumoniae subsp. pneumoniae KPX | pKPX-1 DNA | 5.0E-37 | 96.97 | 99 | 7 |
| NC_021199.1 | Klebsiella pneumoniae subsp. pneumoniae KPX | pKPX-2 DNA | 4.0E-36 | 98.91 | 92 | 2 |
| NC_009649.1 | Klebsiella pneumoniae subsp. pneumoniae MGH 78578 | pKPN3 | 1.0E-37 | 97.03 | 97 | 13 |
| NC_009650.1 | Klebsiella pneumoniae subsp. pneumoniae MGH 78578 | pKPN4 | 2.0E-38 | 97.03 | 101 | 3 |
| NC_009651.1 | Klebsiella pneumoniae subsp. pneumoniae MGH 78578 | pKPN5 | 8.0E-36 | 95.05 | 93 | 35 |
| NC_003789.1 | Klebsiella sp. KCL-2 | pMGD2 | 8.0E-39 | 98.02 | 94 | 14 |
| NC_015213.1 | Lactobacillus acidophilus 30SC | pRKC30SC1 | 5.0E-38 | 99.01 | 90 | 87 |
| NC_015319.1 | Lactobacillus amylovorus GRL 1112 | plasmid1 | 7.0E-38 | 97.92 | 96 | 2 |
| NC_017471.1 | Lactobacillus amylovorus GRL1118 | plasmid1 | 4.0E-37 | 95.05 | 97 | 17 |
| NC_017472.1 | Lactobacillus amylovorus GRL1118 | plasmid2 | 1.0E-32 | 95.05 | 90 | 100 |
| NC_020820.1 | Lactobacillus brevis KB290 | pKB290-1 DNA | 4.0E-36 | 95.05 | 101 | 1 |
| NC_020822.1 | Lactobacillus brevis KB290 | pKB290-4 DNA | 2.0E-43 | 100 | 101 | 1 |
| NC_020823.1 | Lactobacillus brevis KB290 | pKB290-5 DNA | 1.0E-43 | 100 | 101 | 2 |
| NC_020828.1 | Lactobacillus brevis KB290 | pKB290-8 DNA | 7.0E-44 | 100 | 101 | 1 |
| NC_005952.1 | Lactobacillus brevis | pRH45II | 2.0E-43 | 100 | 101 | 1 |
| NC_018611.1 | Lactobacillus buchneri CD034 | pCD034-3 | 1.0E-38 | 97.03 | 101 | 1 |
| NC_015429.1 | Lactobacillus buchneri NRRL B-30929 | pLBUC02 | 9.0E-40 | 98.02 | 101 | 7 |
| NC_015421.1 | Lactobacillus buchneri NRRL B-30929 | pLBUC03 | 9.0E-44 | 100 | 101 | 4 |
| NC_008502.1 | Lactobacillus casei ATCC 334 | 1 | 1.0E-41 | 99.01 | 101 | 3 |
| NC_017476.1 | Lactobacillus casei BD-II | pBD-II | 8.0E-40 | 98 | 100 | 1 |
| NC_021722.1 | Lactobacillus casei LOCK919 | pLOCK919 | 3.0E-36 | 95.05 | 101 | 3 |
| NC_011352.1 | Lactobacillus casei str. Zhang | plca36 | 6.0E-39 | 97.03 | 101 | 6 |
| NC_016975.1 | Lactobacillus casei strain TISTR1341 | pRCEID7.6 | 3.0E-42 | 99.01 | 101 | 1 |
| NC_020057.1 | Lactobacillus casei W56 | pW56 | 3.0E-38 | 98.02 | 92 | 3 |
| NC_003320.2 | Lactobacillus curvatus strain CRL705 | pRC18 | 9.0E-40 | 98.02 | 101 | 1 |
| NC_004947.1 | Lactobacillus fermentum | pKC5b | 1.0E-37 | 96.04 | 101 | 8 |
| NC_011839.1 | Lactobacillus gasseri | pLgLA39 | 1.0E-40 | 98.02 | 101 | 5 |
| NC_017468.1 | Lactobacillus helveticus H10 | pH10 | 2.0E-36 | 95.05 | 99 | 3 |
| NC_013505.1 | Lactobacillus johnsonii FI9785 | p9785L | 4.0E-39 | 97.03 | 101 | 2 |
| NC_015598.1 | Lactobacillus kefiranofaciens ZW3 | pWW1 | 2.0E-35 | 95.05 | 101 | 6 |
| NC_015603.1 | Lactobacillus kefiranofaciens ZW3 | pWW2 | 1.0E-37 | 96.97 | 99 | 3 |
| NC_022114.1 | Lactobacillus paracasei subsp. paracasei 8700:2 | 1 | 2.0E-36 | 95.05 | 101 | 3 |
| NC_022123.1 | Lactobacillus paracasei subsp. paracasei 8700:2 | 2 | 3.0E-33 | 95 | 91 | 5 |
| NC_013544.1 | Lactobacillus paracasei subsp. paracasei | pCD02 | 3.0E-42 | 99.01 | 101 | 1 |
| NC_012222.2 | Lactobacillus paracasei subsp. paracasei | pSJ2-8 | 1.0E-43 | 100 | 101 | 2 |
| NC_021515.1 | Lactobacillus plantarum 16 | Lp16A | 2.0E-38 | 96.04 | 101 | 1 |
| NC_021525.1 | Lactobacillus plantarum 16 | Lp16B | 7.0E-44 | 100 | 101 | 1 |
| NC_021516.1 | Lactobacillus plantarum 16 | Lp16C | 9.0E-42 | 99.01 | 101 | 2 |
| NC_021526.1 | Lactobacillus plantarum 16 | Lp16D | 2.0E-40 | 98.02 | 100 | 2 |
| NC_021517.1 | Lactobacillus plantarum 16 | Lp16E | 2.0E-40 | 98.02 | 101 | 2 |
| NC_004944.1 | Lactobacillus plantarum 5057 | pMD5057 | 2.0E-39 | 97.03 | 101 | 11 |
| NC_019371.1 | Lactobacillus plantarum | pG6303 | 8.0E-38 | 96 | 100 | 1 |
| NC_012628.1 | Lactobacillus plantarum | pLFE1 | 9.0E-39 | 98 | 91 | 105 |
| NC_011101.1 | Lactobacillus plantarum | pLTK13 | 3.0E-43 | 100 | 101 | 1 |
| NC_019321.1 | Lactobacillus plantarum | pR18 | 4.0E-35 | 95.83 | 96 | 1 |
| NC_021233.1 | Lactobacillus plantarum subsp. plantarum P-8 | LBPp1 | 2.0E-40 | 98.02 | 101 | 5 |
| NC_021225.1 | Lactobacillus plantarum subsp. plantarum P-8 | LBPp2 | 2.0E-41 | 99.01 | 101 | 1 |
| NC_021234.1 | Lactobacillus plantarum subsp. plantarum P-8 | LBPp4 | 1.0E-41 | 99.01 | 101 | 2 |
| NC_021227.1 | Lactobacillus plantarum subsp. plantarum P-8 | LBPp5 | 1.0E-43 | 100 | 101 | 1 |
| NC_021228.1 | Lactobacillus plantarum subsp. plantarum P-8 | LBPp6 | 7.0E-44 | 100 | 101 | 1 |
| NC_006377.1 | Lactobacillus plantarum WCFS1 | pWCFS103 | 6.0E-39 | 97.03 | 101 | 5 |
| NC_021903.1 | Lactobacillus plantarum ZJ316 | pLP-ZJ101 | 5.0E-42 | 99.01 | 101 | 1 |
| NC_021912.1 | Lactobacillus plantarum ZJ316 | pLP-ZJ103 | 9.0E-38 | 96.04 | 101 | 2 |
| NC_004532.1 | Lactobacillus reuteri endogenous |  | 2.0E-41 | 98.02 | 99 | 7 |
| NC_021503.1 | Lactobacillus reuteri I5007 | pLRI01 | 2.0E-41 | 99.01 | 101 | 5 |
| NC_021495.1 | Lactobacillus reuteri I5007 | pLRI02 | 4.0E-37 | 98.02 | 92 | 7 |
| NC_021504.1 | Lactobacillus reuteri I5007 | pLRI04 | 1.0E-36 | 95.05 | 95 | 35 |
| NC_021497.1 | Lactobacillus reuteri I5007 | pLRI05 | 1.0E-36 | 95.05 | 101 | 3 |
| NC_015700.1 | Lactobacillus reuteri SD2112 | pLR581 | 2.0E-33 | 95.65 | 92 | 15 |
| NC_015701.1 | Lactobacillus reuteri SD2112 | pLR584 | 4.0E-38 | 96.04 | 99 | 9 |
| NC_010603.1 | Lactobacillus reuteri strain ATCC 55730 | pLR581 | 1.0E-36 | 95.05 | 101 | 11 |
| NC_011225.1 | Lactobacillus rhamnosus HN001 | pLR002 | 7.0E-38 | 96.04 | 101 | 6 |
| NC_013200.1 | Lactobacillus rhamnosus Lc 705 | pLC1 | 1.0E-37 | 96.04 | 101 | 6 |
| NC_004942.1 | Lactobacillus sakei | pRV500 | 1.0E-43 | 100 | 101 | 2 |
| NC_017479.1 | Lactobacillus salivarius CECT 5713 | pHN1 | 4.0E-43 | 100 | 101 | 2 |
| NC_017499.1 | Lactobacillus salivarius CECT 5713 | pHN3 | 3.0E-34 | 95.05 | 99 | 6 |
| NC_007930.1 | Lactobacillus salivarius UCC118 | pMP118 | 8.0E-41 | 99.01 | 101 | 2 |
| NC_006530.1 | Lactobacillus salivarius UCC118 | pSF118-44 | 3.0E-37 | 96 | 92 | 10 |
| NC_015979.1 | Lactobacillus sanfranciscensis TMW 1.1304 | pLS1 | 5.0E-43 | 100 | 101 | 1 |
| NC_016970.1 | Lactococcus garvieae 21881 | pGL3 | 4.0E-42 | 99.01 | 101 | 2 |
| NC_016971.1 | Lactococcus garvieae 21881 | pGL4 | 4.0E-36 | 97.87 | 94 | 4 |
| NC_010540.1 | Lactococcus garvieae | pKL0018 DNA | 4.0E-38 | 96.04 | 101 | 7 |
| NC_007191.1 | Lactococcus lactis cremoris 712 | pAG6 | 2.0E-39 | 97.03 | 99 | 5 |
| NC_004847.1 | Lactococcus lactis cremoris HP | pHP003 | 5.0E-42 | 99.01 | 101 | 4 |
| NC_002137.1 | Lactococcus lactis cremoris NIZO B40 | pNZ4000 | 7.0E-33 | 95.05 | 90 | 11 |
| NC_003101.1 | Lactococcus lactis CRL1127 | pCRL1127 | 1.0E-33 | 95.65 | 92 | 5 |
| NC_002798.1 | Lactococcus lactis DCH-4 | pSRQ700 | 1.0E-33 | 95.65 | 92 | 4 |
| NC_001949.1 | Lactococcus lactis DPC3147 | pMRC01 | 2.0E-40 | 98.02 | 101 | 5 |
| NC_000906.2 | Lactococcus lactis IL964 | pIL105 | 6.0E-38 | 97 | 100 | 2 |
| NC_004966.1 | Lactococcus lactis lactis bv. diacetylactis DPC220 | pAH82 | 8.0E-41 | 98.02 | 101 | 5 |
| NC_004955.1 | Lactococcus lactis lactis IPLA 972 | pBL1 | 9.0E-44 | 100 | 101 | 2 |
| NC_002502.1 | Lactococcus lactis lactis UC317 | pCI305 | 8.0E-37 | 95.05 | 101 | 1 |
| NC_009435.1 | Lactococcus lactis NCDO 1867 | pGdh442 | 2.0E-41 | 99.01 | 101 | 10 |
| NC_002799.1 | Lactococcus lactis | pCRL291.1 | 4.0E-38 | 100 | 90 | 5 |
| NC_011610.1 | Lactococcus lactis | pKL001 | 6.0E-37 | 95.05 | 101 | 2 |
| NC_010901.1 | Lactococcus lactis | pNP40 | 3.0E-40 | 98.02 | 101 | 3 |
| NC_017498.1 | Lactococcus lactis | pSK11A | 8.0E-44 | 100 | 101 | 3 |
| NC_013551.1 | Lactococcus lactis | pSK11B | 6.0E-41 | 98.02 | 101 | 2 |
| NC_017478.1 | Lactococcus lactis | pSK11L | 4.0E-43 | 100 | 101 | 1 |
| NC_017500.1 | Lactococcus lactis | pSK11P | 1.0E-33 | 95 | 95 | 24 |
| NC_004980.1 | Lactococcus lactis | pWC1 | 1.0E-42 | 99.01 | 101 | 1 |
| NC_017495.1 | Lactococcus lactis subsp. cremoris A76 | pQA518 | 1.0E-43 | 100 | 101 | 1 |
| NC_017493.1 | Lactococcus lactis subsp. cremoris A76 | pQA549 | 6.0E-34 | 95.83 | 96 | 9 |
| NC_017496.1 | Lactococcus lactis subsp. cremoris A76 | pQA554 | 5.0E-36 | 95.05 | 91 | 8 |
| NC_019347.1 | Lactococcus lactis subsp. cremoris | pAF04 | 8.0E-39 | 96.04 | 101 | 7 |
| NC_019349.1 | Lactococcus lactis subsp. cremoris | pAF12 | 1.0E-43 | 100 | 101 | 3 |
| NC_019350.1 | Lactococcus lactis subsp. cremoris | pAF14 | 1.0E-36 | 95.96 | 91 | 23 |
| NC_019351.1 | Lactococcus lactis subsp. cremoris | pAF22 | 5.0E-38 | 96.04 | 101 | 6 |
| NC_019377.1 | Lactococcus lactis subsp. cremoris | pLP712 | 4.0E-43 | 100 | 101 | 5 |
| NC_008503.1 | Lactococcus lactis subsp. cremoris SK11 | 1 | 5.0E-42 | 99.01 | 101 | 2 |
| NC_008504.1 | Lactococcus lactis subsp. cremoris SK11 | 2 | 8.0E-44 | 100 | 101 | 1 |
| NC_008505.1 | Lactococcus lactis subsp. cremoris SK11 | 3 | 1.0E-33 | 95.83 | 93 | 16 |
| NC_008506.1 | Lactococcus lactis subsp. cremoris SK11 | 4 | 1.0E-36 | 95.15 | 99 | 12 |
| NC_008507.1 | Lactococcus lactis subsp. cremoris SK11 | 5 | 5.0E-42 | 99.01 | 101 | 3 |
| NC_019434.1 | Lactococcus lactis subsp. cremoris UC509.9 | pCIS2 | 2.0E-42 | 99.01 | 101 | 4 |
| NC_019437.1 | Lactococcus lactis subsp. cremoris UC509.9 | pCIS4 | 6.0E-37 | 95.05 | 101 | 2 |
| NC_019432.1 | Lactococcus lactis subsp. cremoris UC509.9 | pCIS5 | 5.0E-41 | 99.01 | 101 | 1 |
| NC_019436.1 | Lactococcus lactis subsp. cremoris UC509.9 | pCIS6 | 7.0E-39 | 97.03 | 100 | 8 |
| NC_019431.1 | Lactococcus lactis subsp. cremoris UC509.9 | pCIS7 | 6.0E-35 | 95.05 | 96 | 8 |
| NC_004163.1 | Lactococcus lactis subsp. lactis bv. diacetylactis cryptic | pDR1-1B | 1.0E-39 | 97.03 | 101 | 1 |
| NC_009137.1 | Lactococcus lactis subsp. lactis bv. diacetylactis | pDBORO | 6.0E-42 | 99.01 | 101 | 2 |
| NC_004652.1 | Lactococcus lactis subsp. lactis bv. diacetylactis | pS7a | 2.0E-36 | 95 | 96 | 3 |
| NC_004653.1 | Lactococcus lactis subsp. lactis bv. diacetylactis | pS7b | 3.0E-41 | 98.06 | 103 | 1 |
| NC_015900.1 | Lactococcus lactis subsp. lactis bv. diacetylactis | pVF18 | 4.0E-38 | 96.04 | 101 | 4 |
| NC_015912.1 | Lactococcus lactis subsp. lactis bv. diacetylactis | pVF21 | 5.0E-38 | 96.04 | 101 | 4 |
| NC_015901.1 | Lactococcus lactis subsp. lactis bv. diacetylactis | pVF22 | 3.0E-41 | 99.01 | 97 | 11 |
| NC_015902.1 | Lactococcus lactis subsp. lactis bv. diacetylactis | pVF50 | 5.0E-36 | 95.05 | 101 | 11 |
| NC_017483.1 | Lactococcus lactis subsp. lactis CV56 | pCV56A | 1.0E-29 | 95.56 | 90 | 6 |
| NC_017487.1 | Lactococcus lactis subsp. lactis CV56 | pCV56B | 2.0E-32 | 95.6 | 91 | 12 |
| NC_017484.1 | Lactococcus lactis subsp. lactis CV56 | pCV56C | 5.0E-39 | 98.02 | 96 | 30 |
| NC_017485.1 | Lactococcus lactis subsp. lactis CV56 | pCV56D | 1.0E-38 | 96.04 | 100 | 2 |
| NC_009751.1 | Lactococcus lactis subsp. lactis K214 | pK214 | 5.0E-39 | 97.03 | 101 | 10 |
| NC_013657.1 | Lactococcus lactis subsp. lactis KF147 | pKF147A | 2.0E-40 | 98.02 | 101 | 1 |
| NC_015860.1 | Lactococcus lactis subsp. lactis | pIL1 | 5.0E-38 | 98.92 | 93 | 5 |
| NC_017489.1 | Lactococcus lactis subsp. lactis | pIL2 | 4.0E-34 | 95.7 | 93 | 9 |
| NC_015862.1 | Lactococcus lactis subsp. lactis | pIL4 | 2.0E-41 | 99.01 | 101 | 7 |
| NC_015863.1 | Lactococcus lactis subsp. lactis | pIL5 | 1.0E-40 | 98.02 | 100 | 13 |
| NC_019308.1 | Lactococcus lactis subsp. lactis | pIL6 | 6.0E-38 | 96.04 | 101 | 2 |
| NC_015864.1 | Lactococcus lactis subsp. lactis | pIL7 | 7.0E-31 | 95.56 | 90 | 9 |
| NC_016042.1 | Lactococcus lactis subsp. lactis | pKP1 | 1.0E-36 | 95.05 | 100 | 27 |
| NC_008594.1 | Lactococcus lactis subsp. lactis | pL2 | 3.0E-39 | 97 | 98 | 7 |
| NC_002748.1 | Lactococcus lactis subsp. lactis strain MJC15 | pCD4 | 2.0E-42 | 99.01 | 101 | 1 |
| NC_004960.1 | Lactococcus lactis W-1 | pSRQ800 | 3.0E-35 | 95.79 | 95 | 13 |
| NC_010370.1 | Laribacter hongkongensis | pHLHK22 | 1.0E-36 | 95.05 | 96 | 5 |
| NC_006628.1 | Laribacter hongkongensis | pHLHK8 | 5.0E-39 | 97 | 100 | 1 |
| NC_023146.1 | Leisingera methylohalidivorans DSM 14336 strain MB2 DSM 14336 |  | 3.0E-32 | 95.05 | 92 | 2 |
| NC_018674.1 | Leuconostoc carnosum JB16 | pKLC1 | 7.0E-42 | 99.01 | 101 | 2 |
| NC_018675.1 | Leuconostoc carnosum JB16 | pKLC3 | 3.0E-43 | 100 | 101 | 1 |
| NC_018699.1 | Leuconostoc carnosum JB16 | pKLC4 | 1.0E-41 | 99.01 | 101 | 1 |
| NC_010470.1 | Leuconostoc citreum KM20 | pLCK1 | 3.0E-36 | 95.92 | 98 | 4 |
| NC_010469.1 | Leuconostoc citreum KM20 | pLCK4 | 5.0E-41 | 98.02 | 101 | 3 |
| NC_014131.1 | Leuconostoc kimchii IMSNU 11154 | LkipL4701 | 3.0E-41 | 99 | 100 | 1 |
| NC_014133.1 | Leuconostoc kimchii IMSNU 11154 | LkipL4719 | 2.0E-36 | 95.05 | 99 | 5 |
| NC_008496.1 | Leuconostoc mesenteroides subsp. mesenteroides ATCC 8293 | pLEUM1 | 8.0E-38 | 96.04 | 101 | 6 |
| NC_016827.1 | Leuconostoc mesenteroides subsp. mesenteroides J18 | pKLE01 | 5.0E-40 | 98 | 100 | 2 |
| NC_016821.1 | Leuconostoc mesenteroides subsp. mesenteroides J18 | pKLE03 | 8.0E-42 | 99.01 | 101 | 1 |
| NC_003383.1 | Listeria innocua Clip11262 | pLI100 | 2.0E-36 | 98.91 | 92 | 5 |
| NC_021513.1 | Listeria innocua strain TTS-2011 | pDB2011 | 3.0E-41 | 98.99 | 99 | 1 |
| NC_014495.1 | Listeria monocytogenes SLCC2755 | pLM1-2bUG1 | 8.0E-40 | 98.98 | 98 | 14 |
| NC_022051.1 | Listeria monocytogenes strain J1926 |  | 4.0E-37 | 96 | 100 | 5 |
| NC_022045.1 | Listeria monocytogenes strain N1-011A |  | 1.0E-42 | 100 | 101 | 1 |
| NC_021828.1 | Listeria monocytogenes strain R2-502 |  | 2.0E-40 | 98.02 | 101 | 1 |
| NC_011996.1 | Macrococcus caseolyticus JCSC5402 | pMCCL2 | 6.0E-43 | 100 | 101 | 2 |
| NC_008739.1 | Marinobacter aquaeolei VT8 | pMAQU02 | 4.0E-37 | 96.04 | 99 | 4 |
| NC_014213.1 | Meiothermus silvanus DSM 9946 | pMESIL01 | 6.0E-38 | 97.03 | 100 | 5 |
| NC_002679.1 | Mesorhizobium loti MAFF303099 | pMLa | 1.0E-34 | 95 | 100 | 4 |
| NC_002682.1 | Mesorhizobium loti MAFF303099 | pMLb | 2.0E-34 | 96.88 | 96 | 1 |
| NC_008242.1 | Mesorhizobium sp. BNC1 | 1 | 4.0E-33 | 95.05 | 94 | 61 |
| NC_008243.1 | Mesorhizobium sp. BNC1 | 2 | 1.0E-34 | 95 | 96 | 39 |
| NC_019972.1 | Methanomethylovorans hollandica DSM 15978 | pMETHO01 | 2.0E-42 | 100 | 101 | 3 |
| NC_008826.1 | Methylibium petroleiphilum PM1 | RPME01 | 2.0E-34 | 95 | 99 | 2 |
| NC_012811.1 | Methylobacterium extorquens AM1 |  | 4.0E-33 | 95.05 | 90 | 180 |
| NC_012987.1 | Methylobacterium extorquens DM4 | p1METDI | 4.0E-35 | 95 | 100 | 1 |
| NC_010510.1 | Methylobacterium radiotolerans JCM 2831 | pMRAD01 | 1.0E-30 | 95 | 90 | 64 |
| NC_004954.1 | Micrococcus sp. 28 | pSD10 | 5.0E-35 | 95.05 | 93 | 19 |
| NC_022599.1 | Micrococcus sp. V7 | pLMV7 | 8.0E-36 | 95.05 | 100 | 11 |
| NC_019760.1 | Microcoleus sp. PCC 7113 | pMIC7113.02 | 2.0E-31 | 95 | 90 | 17 |
| NC_016036.1 | Morganella morganii strain M203 | R485 | 1.0E-38 | 97.03 | 101 | 5 |
| NC_021278.1 | Mycobacterium abscessus subsp. bolletii 50594 | 1 | 3.0E-37 | 96.04 | 101 | 2 |
| NC_021279.1 | Mycobacterium abscessus subsp. bolletii 50594 | 2 | 7.0E-43 | 100 | 101 | 1 |
| NC_017908.2 | Mycobacterium abscessus subsp. bolletii F1725 | BRA100 | 5.0E-36 | 95.05 | 90 | 662 |
| NC_020994.1 | Mycobacterium abscessus subsp. bolletii INCQS 00594 | pMAB01 | 6.0E-35 | 95.05 | 90 | 1393 |
| NC_018022.1 | Mycobacterium chubuense NBB4 | pMYCCH.01 | 1.0E-36 | 96.04 | 99 | 3 |
| NC_014811.1 | Mycobacterium gilvum Spyr1 | pMSPYR101 | 2.0E-42 | 100 | 101 | 1 |
| NC_008147.1 | Mycobacterium sp. MCS | plasmid1 | 2.0E-42 | 100 | 101 | 1 |
| NC_010189.1 | Naegleria gruberi | extrachromosomal rDNA | 3.0E-32 | 95.56 | 90 | 1 |
| NC_014105.1 | Neisseria gonorrhoeae | pEP5289 | 9.0E-38 | 96.04 | 101 | 5 |
| NC_007960.1 | Nitrobacter hamburgensis X14 | 2 | 3.0E-39 | 98 | 100 | 2 |
| NC_007961.1 | Nitrobacter hamburgensis X14 | 3 | 2.0E-33 | 96.74 | 92 | 1 |
| NC_008341.1 | Nitrosomonas eutropha C91 | plasmid1 | 6.0E-36 | 95.05 | 91 | 12 |
| NC_008342.1 | Nitrosomonas eutropha C91 | plasmid2 | 5.0E-36 | 95.05 | 101 | 1 |
| NC_015223.1 | Nitrosomonas sp. AL212 | pNAL21201 | 6.0E-38 | 97 | 100 | 3 |
| NC_015221.1 | Nitrosomonas sp. AL212 | pNAL21202 | 6.0E-36 | 95.05 | 97 | 11 |
| NC_006363.1 | Nocardia farcinica IFM 10152 | pNF2 | 5.0E-38 | 97 | 100 | 1 |
| NC_008697.1 | Nocardioides sp. JS614 | pNOCA01 | 9.0E-35 | 95.05 | 97 | 6 |
| NC_009426.1 | Novosphingobium aromaticivorans DSM 12444 | pNL1 | 7.0E-40 | 98.02 | 101 | 1 |
| NC_015583.1 | Novosphingobium sp. PP1Y | Mpl | 3.0E-35 | 95.96 | 99 | 2 |
| NC_009669.1 | Ochrobactrum anthropi ATCC 49188 | pOANT01 | 3.0E-32 | 95.05 | 90 | 147 |
| NC_009670.1 | Ochrobactrum anthropi ATCC 49188 | pOANT02 | 9.0E-36 | 95.05 | 101 | 14 |
| NC_009671.1 | Ochrobactrum anthropi ATCC 49188 | pOANT03 | 3.0E-35 | 95 | 100 | 9 |
| NC_009672.1 | Ochrobactrum anthropi ATCC 49188 | pOANT04 | 1.0E-37 | 96.04 | 101 | 2 |
| NC_020909.1 | Octadecabacter arcticus 238 | pOA238_118 | 1.0E-35 | 95.05 | 101 | 1 |
| NC_020910.1 | Octadecabacter arcticus 238 | pOA238_160 | 1.0E-35 | 95.05 | 101 | 1 |
| NC_019553.1 | Oenococcus oeni | pOENI-1 | 1.0E-43 | 100 | 101 | 1 |
| NC_019554.1 | Oenococcus oeni | pOENI-1v2 | 4.0E-32 | 95.56 | 90 | 5 |
| NC_017539.1 | Oligotropha carboxidovorans OM4 | pOC167B | 2.0E-40 | 99.01 | 101 | 2 |
| NC_011414.1 | Ornithobacterium rhinotracheale | pOR1 | 5.0E-36 | 95 | 100 | 2 |
| NC_014563.1 | Pantoea vagans C9-1 | pPag2 | 2.0E-34 | 95.83 | 96 | 1 |
| NC_014258.1 | Pantoea vagans C9-1 | pPag3 | 5.0E-35 | 95.05 | 99 | 5 |
| NC_022042.1 | Paracoccus aminophilus JCM 7686 | pAMI1 | 2.0E-32 | 95 | 90 | 162 |
| NC_022050.1 | Paracoccus aminophilus JCM 7686 | pAMI8 | 3.0E-32 | 95 | 90 | 108 |
| NC_010847.2 | Paracoccus aminophilus strain JCM 7686 | pAMI2 | 2.0E-36 | 95.05 | 98 | 5 |
| NC_013513.1 | Paracoccus aminophilus strain JCM 7686 | pAMI3 | 1.0E-38 | 96.04 | 101 | 1 |
| NC_014832.1 | Paracoccus aminophilus strain JCM 7686 | pAMI7 | 4.0E-38 | 96.04 | 91 | 50 |
| NC_008688.1 | Paracoccus denitrificans PD1222 | 1 | 2.0E-34 | 95 | 98 | 11 |
| NC_019289.1 | Paracoccus haeundaensis | pHAE1 | 5.0E-37 | 95.05 | 101 | 2 |
| NC_019356.1 | Paracoccus marcusii | pMOS6 | 4.0E-34 | 96.04 | 91 | 2 |
| NC_009753.1 | Paracoccus methylutens strain DM12 | pMTH1 | 2.0E-37 | 97.03 | 90 | 81 |
| NC_012216.1 | Pasteurella multocida | pB1006 | 9.0E-41 | 98 | 99 | 33 |
| NC_016973.1 | Pasteurella multocida | pCCK411 | 2.0E-42 | 99.01 | 101 | 1 |
| NC_004771.1 | Pasteurella multocida | pJR1 | 6.0E-37 | 95.05 | 101 | 2 |
| NC_004772.1 | Pasteurella multocida | pJR2 | 4.0E-44 | 100 | 101 | 2 |
| NC_010864.1 | Pediococcus acidilactici | pEOC01 | 2.0E-39 | 97.03 | 101 | 13 |
| NC_016608.1 | Pediococcus claussenii ATCC BAA-344 | pPECL-5 | 3.0E-36 | 95.05 | 99 | 7 |
| NC_017017.1 | Pediococcus claussenii ATCC BAA-344 | pPECL-6 | 8.0E-41 | 98.02 | 101 | 2 |
| NC_017019.1 | Pediococcus claussenii ATCC BAA-344 | pPECL-8 | 1.0E-34 | 95.96 | 93 | 5 |
| NC_012031.1 | Pediococcus pentosaceus | pRS5 | 9.0E-37 | 95.05 | 101 | 1 |
| NC_008608.1 | Pelobacter propionicus DSM 2379 | pPRO2 | 1.0E-35 | 95 | 90 | 232 |
| NC_018421.1 | Phaeobacter gallaeciensis 2.10 | pPGA2_239 | 9.0E-34 | 95 | 90 | 2 |
| NC_023148.1 | Phaeobacter gallaeciensis DSM 26640 | pGal_B134 | 4.0E-35 | 95.05 | 97 | 31 |
| NC_011143.1 | Phenylobacterium zucineum HLK1 | unnamed | 1.0E-39 | 98.02 | 101 | 10 |
| NC_016983.1 | Photobacterium damselae subsp. damselae | pAQU1 | 6.0E-35 | 95 | 96 | 22 |
| NC_008757.1 | Polaromonas naphthalenivorans CJ2 | pPNAP01 | 7.0E-31 | 95.05 | 90 | 153 |
| NC_008758.1 | Polaromonas naphthalenivorans CJ2 | pPNAP02 | 3.0E-32 | 95.65 | 90 | 15 |
| NC_008759.1 | Polaromonas naphthalenivorans CJ2 | pPNAP03 | 3.0E-38 | 97.03 | 101 | 1 |
| NC_008760.1 | Polaromonas naphthalenivorans CJ2 | pPNAP04 | 2.0E-34 | 95 | 96 | 15 |
| NC_008761.1 | Polaromonas naphthalenivorans CJ2 | pPNAP05 | 2.0E-35 | 95 | 99 | 15 |
| NC_007949.1 | Polaromonas sp. JS666 | 1 | 4.0E-34 | 95 | 95 | 27 |
| NC_007950.1 | Polaromonas sp. JS666 | 2 | 4.0E-34 | 95.05 | 95 | 6 |
| NC_015258.1 | Polymorphum gilvum SL003B-26A1 | pSL003B | 3.0E-33 | 95.05 | 91 | 4 |
| NC_022111.1 | Prevotella sp. oral taxon 299 str. F0039 |  | 1.0E-28 | 95 | 90 | 69 |
| NC_011513.1 | Proteus mirabilis | pRPCMY | 2.0E-39 | 97 | 93 | 25 |
| NC_010643.1 | Providencia rettgeri | R7K | 3.0E-43 | 100 | 101 | 4 |
| NC_016974.1 | Providencia stuartii | pMR0211 | 2.0E-35 | 95.05 | 101 | 1 |
| NC_019375.1 | Providencia stuartii | pTC2 | 1.0E-42 | 100 | 101 | 1 |
| NC_010675.1 | Pseudoalteromonas sp. 643A | pKW1 | 4.0E-37 | 95.05 | 101 | 1 |
| NC_008357.1 | Pseudomonas aeruginosa | pBS228 | 8.0E-36 | 95.05 | 101 | 24 |
| NC_009739.1 | Pseudomonas aeruginosa | pMATVIM-7 | 8.0E-36 | 95 | 90 | 72 |
| NC_022344.1 | Pseudomonas aeruginosa | pOZ176 | 9.0E-31 | 95 | 90 | 303 |
| NC_007100.1 | Pseudomonas aeruginosa | Rms149 | 5.0E-36 | 95.05 | 97 | 68 |
| NC_020452.1 | Pseudomonas aeruginosa strain COL-1 | pNOR-2000 | 7.0E-36 | 95 | 92 | 39 |
| NC_022345.1 | Pseudomonas aeruginosa strain ST1006 | pPA-2 | 3.0E-41 | 99.01 | 96 | 2 |
| NC_022346.1 | Pseudomonas aeruginosa strain ST308 | pCOL-1 | 1.0E-34 | 95 | 92 | 202 |
| NC_005909.1 | Pseudomonas alcaligenes | pRA2 | 7.0E-38 | 96.04 | 96 | 47 |
| NC_009444.1 | Pseudomonas fluorescens SBW25 | pQBR103 | 3.0E-42 | 100 | 101 | 1 |
| NC_012674.1 | Pseudomonas fluorescens strain PC20 | pNAH20 | 1.0E-38 | 97.03 | 101 | 2 |
| NC_021250.1 | Pseudomonas migulae strain D2RT | pD2RT | 1.0E-35 | 95.05 | 101 | 19 |
| NC_019906.1 | Pseudomonas putida HB3267 | pPC9 | 7.0E-36 | 95.05 | 101 | 4 |
| NC_004999.1 | Pseudomonas putida NCIB 9816-4 | pDTG1 | 6.0E-43 | 100 | 101 | 2 |
| NC_014124.1 | Pseudomonas putida | pDK1 | 4.0E-35 | 95.88 | 97 | 9 |
| NC_015855.1 | Pseudomonas putida | pGRT1 | 4.0E-35 | 95 | 100 | 6 |
| NC_013176.1 | Pseudomonas putida | pW2 | 2.0E-35 | 95 | 95 | 44 |
| NC_003350.1 | Pseudomonas putida | pWW0 | 2.0E-33 | 95 | 92 | 27 |
| NC_023274.1 | Pseudomonas putida strain LD209 | pLD209 | 3.0E-36 | 95.05 | 101 | 16 |
| NC_021506.1 | Pseudomonas resinovorans NBRC 106553 | pCAR1.3 DNA | 1.0E-31 | 95.05 | 90 | 44 |
| NC_004444.1 | Pseudomonas resinovorans | pCAR1 | 3.0E-33 | 96.74 | 92 | 3 |
| NC_004956.1 | Pseudomonas sp. ADP atrazine catabolic | pADP-1 | 2.0E-32 | 95 | 90 | 242 |
| NC_010891.1 | Pseudomonas sp. CT14 | pCT14 | 5.0E-36 | 95.05 | 92 | 204 |
| NC_005244.2 | Pseudomonas sp. ND6 | pND6-1 | 9.0E-36 | 95.05 | 98 | 6 |
| NC_022739.1 | Pseudomonas sp. VLB120 | pSTY | 3.0E-35 | 95.05 | 98 | 11 |
| NC_019334.1 | Pseudomonas syringae | pPT14-32 | 1.0E-40 | 98.02 | 101 | 2 |
| NC_007968.1 | Psychrobacter cryohalolentis K5 | 1 | 4.0E-36 | 95.05 | 96 | 46 |
| NC_021158.1 | Psychrobacter maritimus | pKLH80 | 4.0E-37 | 96 | 91 | 73 |
| NC_019306.1 | Psychrobacter sp. DAB_AL43B | pP43BP3 | 5.0E-37 | 95.05 | 101 | 1 |
| NC_019305.1 | Psychrobacter sp. DAB_AL43B | pP43BP4 | 6.0E-37 | 95.05 | 101 | 3 |
| NC_019274.1 | Psychrobacter sp. DAB_AL62B | pP62BP1 | 3.0E-36 | 95.05 | 101 | 4 |
| NC_021668.1 | Psychrobacter sp. G | PsyG_26 | 2.0E-36 | 95.05 | 100 | 11 |
| NC_009516.1 | Psychrobacter sp. PRwf-1 | pRWF101 | 2.0E-35 | 95.83 | 94 | 5 |
| NC_016819.1 | Rahnella aquatilis CIP 78.65 = ATCC 33071 | pRahaq202 | 5.0E-33 | 96.7 | 91 | 1 |
| NC_017060.1 | Rahnella aquatilis HX2 | PRA1 | 1.0E-36 | 96.04 | 101 | 2 |
| NC_019223.1 | Rahnella sp. WMR66 | pHW66 | 1.0E-39 | 97.03 | 101 | 1 |
| NC_005241.1 | Ralstonia eutropha H16 mega | pHG1 | 6.0E-33 | 95 | 92 | 5 |
| NC_007337.1 | Ralstonia eutropha JMP134 | 1 | 8.0E-36 | 97.85 | 93 | 2 |
| NC_012855.1 | Ralstonia pickettii 12D | pRp12D01 | 3.0E-35 | 95.92 | 98 | 2 |
| NC_012849.1 | Ralstonia pickettii 12D | pRp12D02 | 3.0E-33 | 95 | 90 | 79 |
| NC_019318.1 | Ralstonia pickettii | p712 | 7.0E-35 | 95.05 | 90 | 105 |
| NC_014309.1 | Ralstonia solanacearum CFBP2957 | RCFBPv3_mp | 6.0E-34 | 95 | 99 | 6 |
| NC_017589.1 | Ralstonia solanacearum CMR15 | CMR15_mp | 3.0E-30 | 95 | 90 | 205 |
| NC_021745.1 | Ralstonia solanacearum FQY_4 |  | 4.0E-30 | 95 | 90 | 488 |
| NC_003296.1 | Ralstonia solanacearum GMI1000 | pGMI1000MP | 5.0E-29 | 95 | 90 | 1222 |
| NC_017575.1 | Ralstonia solanacearum Po82 |  | 3.0E-31 | 95 | 90 | 9 |
| NC_014310.1 | Ralstonia solanacearum PSI07 mega | mpPSI07 | 2.0E-33 | 95 | 94 | 2 |
| NC_021906.1 | Rhizobium etli bv. mimosae str. Mim1 | pRetMIM1a | 2.0E-35 | 95.92 | 98 | 3 |
| NC_021909.1 | Rhizobium etli bv. mimosae str. Mim1 | pRetMIM1e | 5.0E-35 | 95.05 | 100 | 2 |
| NC_021911.1 | Rhizobium etli bv. mimosae str. Mim1 | pRetMIM1f | 3.0E-34 | 95 | 100 | 4 |
| NC_007762.1 | Rhizobium etli CFN 42 | p42a | 9.0E-33 | 95.05 | 93 | 5 |
| NC_007763.1 | Rhizobium etli CFN 42 | p42b | 3.0E-32 | 95.05 | 92 | 2 |
| NC_007764.1 | Rhizobium etli CFN 42 | p42c | 4.0E-38 | 97.03 | 101 | 1 |
| NC_007765.1 | Rhizobium etli CFN 42 | p42e | 1.0E-34 | 95 | 100 | 6 |
| NC_004041.2 | Rhizobium etli CFN 42 symbiotic | p42d | 3.0E-35 | 95.05 | 101 | 4 |
| NC_010998.1 | Rhizobium etli CIAT 652 | pA | 2.0E-32 | 95 | 100 | 5 |
| NC_010996.1 | Rhizobium etli CIAT 652 | pB | 4.0E-34 | 95.05 | 101 | 3 |
| NC_010997.1 | Rhizobium etli CIAT 652 | pC | 9.0E-35 | 95.05 | 101 | 1 |
| NC_012848.1 | Rhizobium leguminosarum bv. trifolii WSM1325 | pR132501 | 2.0E-34 | 96.84 | 95 | 1 |
| NC_012858.1 | Rhizobium leguminosarum bv. trifolii WSM1325 | pR132502 | 5.0E-36 | 96 | 100 | 1 |
| NC_012853.1 | Rhizobium leguminosarum bv. trifolii WSM1325 | pR132503 | 1.0E-35 | 95.96 | 99 | 1 |
| NC_012852.1 | Rhizobium leguminosarum bv. trifolii WSM1325 | pR132504 | 3.0E-35 | 95.92 | 98 | 1 |
| NC_012854.1 | Rhizobium leguminosarum bv. trifolii WSM1325 | pR132505 | 4.0E-33 | 95.74 | 94 | 2 |
| NC_011368.1 | Rhizobium leguminosarum bv. trifolii WSM2304 | pRLG201 | 1.0E-34 | 95.05 | 98 | 5 |
| NC_011366.1 | Rhizobium leguminosarum bv. trifolii WSM2304 | pRLG202 | 4.0E-35 | 95.05 | 101 | 3 |
| NC_011370.1 | Rhizobium leguminosarum bv. trifolii WSM2304 | pRLG203 | 6.0E-37 | 96.04 | 101 | 1 |
| NC_011371.1 | Rhizobium leguminosarum bv. trifolii WSM2304 | pRLG204 | 3.0E-34 | 95.05 | 96 | 3 |
| NC_008384.1 | Rhizobium leguminosarum bv. viciae 3841 | pRL11 | 1.0E-36 | 96.04 | 101 | 1 |
| NC_008378.1 | Rhizobium leguminosarum bv. viciae 3841 | pRL12 | 7.0E-35 | 95.05 | 101 | 2 |
| NC_008382.1 | Rhizobium leguminosarum bv. viciae 3841 | pRL7 | 1.0E-35 | 95.05 | 98 | 5 |
| NC_008383.1 | Rhizobium leguminosarum bv. viciae 3841 | pRL8 | 3.0E-31 | 95.56 | 90 | 3 |
| NC_022536.1 | Rhizobium sp. IRBG74 | IRBL74_p | 5.0E-35 | 95.05 | 101 | 4 |
| NC_020060.1 | Rhizobium tropici CIAT 899 | pRtrCIAT899a | 2.0E-35 | 95.05 | 101 | 3 |
| NC_020061.1 | Rhizobium tropici CIAT 899 | pRtrCIAT899b | 2.0E-34 | 95.05 | 90 | 6 |
| NC_020062.1 | Rhizobium tropici CIAT 899 | pRtrCIAT899c | 6.0E-34 | 95 | 99 | 6 |
| NC_014035.1 | Rhodobacter capsulatus SB 1003 | pRCB133 | 3.0E-37 | 96.04 | 101 | 2 |
| NC_007490.2 | Rhodobacter sphaeroides 2.4.1 | D | 2.0E-38 | 97.03 | 101 | 1 |
| NC_009429.1 | Rhodobacter sphaeroides ATCC 17025 | pRSPA01 | 1.0E-32 | 95 | 92 | 13 |
| NC_009430.1 | Rhodobacter sphaeroides ATCC 17025 | pRSPA02 | 4.0E-32 | 95 | 90 | 110 |
| NC_022125.1 | Rhodococcus erythropolis CCM2595 | pRECF1 | 2.0E-37 | 96.04 | 101 | 4 |
| NC_005073.1 | Rhodococcus erythropolis linear | pBD2 | 4.0E-37 | 96.04 | 100 | 5 |
| NC_007486.1 | Rhodococcus erythropolis PR4 | pREC1 | 8.0E-43 | 100 | 101 | 1 |
| NC_007491.1 | Rhodococcus erythropolis PR4 | pREL1 | 3.0E-34 | 95.05 | 94 | 6 |
| NC_023144.1 | Rhodococcus pyridinivorans SB3094 |  | 6.0E-38 | 97.03 | 101 | 1 |
| NC_015950.1 | Riemerella anatipestifer | pRA0846 | 4.0E-35 | 95.88 | 97 | 1 |
| NC_008042.1 | Ruegeria sp. TM1040 | unnamed | 1.0E-34 | 95.05 | 91 | 28 |
| NC_001398.1 | Saccharomyces cerevisiae A364A 2 |  | 6.0E-37 | 95.05 | 99 | 10 |
| NC_003079.1 | Salmonella choleraesuis 79500 | pSFD10 | 1.0E-42 | 99.01 | 101 | 2 |
| NC_005862.1 | Salmonella enterica enterica sv Choleraesuis | cryptic | 1.0E-39 | 97.03 | 101 | 7 |
| NC_002638.1 | Salmonella enterica enterica sv Choleraesuis RF-1 | pKDSC50 | 4.0E-36 | 97.85 | 93 | 1 |
| NC_021817.1 | Salmonella enterica subsp. enterica serovar Bareilly str. CFSAN000189 | unnamed | 7.0E-36 | 95.05 | 100 | 3 |
| NC_010499.1 | Salmonella enterica subsp. enterica serovar Brandenburg | pUO-SbR3 | 1.0E-38 | 96.04 | 94 | 23 |
| NC_010500.1 | Salmonella enterica subsp. enterica serovar Brandenburg | pUO-SbR5 | 1.0E-33 | 96.04 | 90 | 19 |
| NC_010119.1 | Salmonella enterica subsp. enterica serovar Choleraesuis | pOU7519 | 1.0E-35 | 95.05 | 101 | 1 |
| NC_021819.1 | Salmonella enterica subsp. enterica Serovar Cubana str. CFSAN002050 |  | 5.0E-40 | 98.02 | 100 | 5 |
| NC_021845.1 | Salmonella enterica subsp. enterica Serovar Cubana str. CFSAN002050 |  | 1.0E-42 | 100 | 101 | 1 |
| NC_019134.1 | Salmonella enterica subsp. enterica serovar Derby | pSD4.0 | 1.0E-42 | 99.01 | 101 | 3 |
| NC_019135.1 | Salmonella enterica subsp. enterica serovar Derby | pSD4.6 | 8.0E-40 | 97.03 | 101 | 4 |
| NC_010716.1 | Salmonella enterica subsp. enterica serovar Dublin | IncW pIE321 | 1.0E-41 | 99.01 | 101 | 9 |
| NC_009980.1 | Salmonella enterica subsp. enterica serovar Dublin | pMAK2 | 2.0E-34 | 95.79 | 95 | 34 |
| NC_019106.1 | Salmonella enterica subsp. enterica serovar Dublin | pSD_77 | 6.0E-43 | 100 | 101 | 1 |
| NC_019105.1 | Salmonella enterica subsp. enterica serovar Dublin | pSD_88 | 8.0E-36 | 95.05 | 99 | 9 |
| NC_019121.1 | Salmonella enterica subsp. enterica serovar Heidelberg | pSH111_166 | 6.0E-40 | 98.02 | 101 | 2 |
| NC_019114.1 | Salmonella enterica subsp. enterica serovar Heidelberg | pSH111_227 | 7.0E-41 | 99.01 | 101 | 6 |
| NC_019123.1 | Salmonella enterica subsp. enterica serovar Heidelberg | pSH1148_107 | 5.0E-33 | 95.05 | 90 | 56 |
| NC_019115.1 | Salmonella enterica subsp. enterica serovar Heidelberg | pSH146_65 | 1.0E-37 | 99.01 | 91 | 6 |
| NC_019116.1 | Salmonella enterica subsp. enterica serovar Heidelberg | pSH163_135 | 5.0E-40 | 98.02 | 101 | 1 |
| NC_019117.1 | Salmonella enterica subsp. enterica serovar Heidelberg | pSH696_117 | 9.0E-43 | 100 | 101 | 3 |
| NC_019128.1 | Salmonella enterica subsp. enterica serovar Heidelberg | pSH696_34 | 3.0E-43 | 100 | 101 | 3 |
| NC_021841.1 | Salmonella enterica subsp. enterica serovar Heidelberg str. 41578 | pSEEH1578_02 | 3.0E-43 | 100 | 101 | 1 |
| NC_017624.1 | Salmonella enterica subsp. enterica serovar Heidelberg str. B182 | pB182_37 | 2.0E-38 | 97.94 | 97 | 1 |
| NC_021813.2 | Salmonella enterica subsp. enterica serovar Heidelberg str. CFSAN002069 | pCFSAN002069_01 | 1.0E-34 | 95.83 | 96 | 24 |
| NC_011082.1 | Salmonella enterica subsp. enterica serovar Heidelberg str. SL476 | pSL476_3 | 3.0E-44 | 100 | 101 | 2 |
| NC_011081.1 | Salmonella enterica subsp. enterica serovar Heidelberg str. SL476 | pSL476_91 | 7.0E-43 | 100 | 101 | 12 |
| NC_019104.1 | Salmonella enterica subsp. enterica serovar Kentucky | pCS0010A_95 | 7.0E-43 | 100 | 101 | 5 |
| NC_011076.1 | Salmonella enterica subsp. enterica serovar Kentucky str. CVM29188 | pCVM29188_146 | 4.0E-35 | 97.87 | 94 | 1 |
| NC_022522.1 | Salmonella enterica subsp. enterica serovar Kentucky strain 1643/10 | p1643_10 | 1.0E-42 | 100 | 101 | 1 |
| NC_010259.1 | Salmonella enterica subsp. enterica serovar Newport | pA172 | 8.0E-37 | 95.05 | 93 | 76 |
| NC_014003.1 | Salmonella enterica subsp. enterica serovar Newport | pSN11/00Kan | 5.0E-44 | 100 | 101 | 1 |
| NC_010894.1 | Salmonella enterica subsp. enterica serovar Paratyphi A | pGY1 | 3.0E-44 | 100 | 101 | 1 |
| NC_011092.1 | Salmonella enterica subsp. enterica serovar Schwarzengrund str. CVM19633 | pCVM19633_110 | 5.0E-33 | 95.7 | 93 | 12 |
| NC_003384.1 | Salmonella enterica subsp. enterica serovar Typhi str. CT18 | pHCM1 | 4.0E-38 | 97.03 | 101 | 12 |
| NC_003385.1 | Salmonella enterica subsp. enterica serovar Typhi str. CT18 | pHCM2 | 9.0E-36 | 95.05 | 101 | 1 |
| NC_016825.1 | Salmonella enterica subsp. enterica serovar Typhi str. P-stx-12 | unnamed | 4.0E-37 | 96.04 | 99 | 2 |
| NC_002090.1 | Salmonella enterica subsp. enterica serovar Typhimurium | NTP16 | 3.0E-42 | 99.01 | 100 | 7 |
| NC_002056.1 | Salmonella enterica subsp. enterica serovar Typhimurium | pSC101 | 3.0E-43 | 100 | 100 | 3 |
| NC_006816.1 | Salmonella enterica subsp. enterica serovar Typhimurium | pU302L | 7.0E-36 | 95.05 | 101 | 3 |
| NC_022372.1 | Salmonella enterica subsp. enterica serovar Typhimurium | pYT3 DNA | 1.0E-35 | 95.05 | 97 | 3 |
| NC_003292.1 | Salmonella enterica subsp. enterica serovar Typhimurium | R46 | 2.0E-35 | 95 | 91 | 8 |
| NC_015965.1 | Salmonella enterica subsp. enterica serovar Typhimurium | R621a | 7.0E-43 | 100 | 101 | 4 |
| NC_005014.1 | Salmonella enterica subsp. enterica serovar Typhimurium | R64 | 9.0E-43 | 100 | 101 | 1 |
| NC_017675.1 | Salmonella enterica subsp. enterica serovar Typhimurium str. ST4/74 | TY474p2 | 2.0E-37 | 96.04 | 101 | 9 |
| NC_023275.1 | Salmonella enterica subsp. enterica serovar Typhimurium strain 9134 | p9134 | 1.0E-42 | 100 | 101 | 1 |
| NC_021815.1 | Salmonella enterica subsp. enterica serovar Typhimurium var. 5- str. CFSAN001921 | unnamed | 2.0E-35 | 95.05 | 94 | 24 |
| NC_019124.1 | Salmonella enterica subsp. enterica serovar Virchow | pVQS1 | 3.0E-43 | 100 | 101 | 1 |
| NC_011214.1 | Salmonella enterica subsp. enterica serovar Virchow str. SL491 | pSL491_5 | 6.0E-37 | 95.05 | 101 | 1 |
| NC_011604.1 | Salmonella enterica subsp. enterica serovar Westhampton | pWES-1 | 2.0E-33 | 95.92 | 91 | 6 |
| NC_019125.1 | Salmonella enterica subsp. salamae | pSGSC3045-121 | 1.0E-35 | 95.05 | 101 | 2 |
| NC_019342.1 | Salmonella sp. 14 | p14-120 | 5.0E-40 | 98.02 | 101 | 2 |
| NC_002305.1 | Salmonella typhi | R27 | 7.0E-40 | 98.02 | 101 | 8 |
| NC_013518.1 | Sebaldella termitidis ATCC 33386 | pSTERM01 | 9.0E-39 | 97.03 | 101 | 5 |
| NC_002523.4 | Serratia entomophila | pADAP | 3.0E-37 | 96.04 | 101 | 2 |
| NC_021742.1 | Serratia liquefaciens ATCC 27592 |  | 3.0E-31 | 95 | 90 | 231 |
| NC_019267.1 | Serratia marcescens | pRIO-5 | 1.0E-37 | 98.92 | 93 | 5 |
| NC_005211.1 | Serratia marcescens | R478 | 2.0E-42 | 100 | 101 | 1 |
| NC_019344.1 | Serratia marcescens | R830b | 1.0E-38 | 97.03 | 93 | 36 |
| NC_011664.1 | Shewanella baltica OS223 | pS22301 | 8.0E-36 | 95.05 | 101 | 1 |
| NC_008573.1 | Shewanella sp. ANA-3 | 1 | 2.0E-35 | 95.05 | 90 | 218 |
| NC_010660.1 | Shigella boydii CDC 3083-94 | pBS512_211 | 7.0E-41 | 99.01 | 101 | 1 |
| NC_007608.1 | Shigella boydii Sb227 | pSB4_227 | 5.0E-40 | 98.02 | 101 | 3 |
| NC_007607.1 | Shigella dysenteriae Sd197 | pSD1_197 | 2.0E-34 | 97.03 | 91 | 2 |
| NC_002698.1 | Shigella flexneri 5a virulence | pWR501 | 3.0E-32 | 96.04 | 90 | 6 |
| NC_019249.1 | Shigella flexneri | pSF301-2 | 2.0E-40 | 97.98 | 99 | 1 |
| NC_019197.1 | Shigella flexneri | pSF5 | 1.0E-36 | 98.92 | 93 | 2 |
| NC_019250.1 | Shigella flexneri strain 2a 301 | pSF301-3 | 7.0E-40 | 97.03 | 101 | 1 |
| NC_020991.1 | Shigella sonnei 10188 | pKHSB1 | 3.0E-41 | 99.01 | 101 | 1 |
| NC_016833.1 | Shigella sonnei 53G | A | 4.0E-38 | 97.03 | 101 | 1 |
| NC_002122.1 | Shigella sonnei | P9 DNA | 7.0E-43 | 100 | 101 | 2 |
| NC_007385.1 | Shigella sonnei Ss046 | pSS_046 | 1.0E-37 | 97 | 100 | 1 |
| NC_009345.1 | Shigella sonnei Ss046 | pSS046_spA | 4.0E-41 | 100 | 96 | 7 |
| NC_009346.1 | Shigella sonnei Ss046 | pSS046_spB | 2.0E-42 | 99.01 | 101 | 1 |
| NC_019256.1 | Shigella sp. LN126 | pLN126_33 | 1.0E-41 | 99.01 | 101 | 4 |
| NC_019254.1 | Shigella sp. MO17 | pMO17_54 | 2.0E-40 | 98.02 | 101 | 4 |
| NC_015742.1 | Sinorhizobium fredii GR64 | p64a | 2.0E-35 | 95.05 | 101 | 2 |
| NC_016813.1 | Sinorhizobium fredii HH103 | pSfHH103a | 1.0E-32 | 95.05 | 91 | 4 |
| NC_016836.1 | Sinorhizobium fredii HH103 | pSfHH103b | 5.0E-36 | 95.05 | 99 | 6 |
| NC_016814.1 | Sinorhizobium fredii HH103 | pSfHH103c | 2.0E-38 | 97.03 | 101 | 3 |
| NC_016815.1 | Sinorhizobium fredii HH103 | pSfHH103e | 7.0E-33 | 95 | 95 | 5 |
| NC_000914.2 | Sinorhizobium fredii NGR234 | pNGR234a | 1.0E-36 | 96.04 | 101 | 4 |
| NC_012586.1 | Sinorhizobium fredii NGR234 | pNGR234b | 4.0E-30 | 95.05 | 90 | 10 |
| NT_187169.1 | Sinorhizobium fredii USDA 257 | pUSDA257 fragment 19 | 4.0E-42 | 99 | 100 | 1 |
| NT_187151.1 | Sinorhizobium fredii USDA 257 | pUSDA257 fragment 2 | 6.0E-40 | 98.02 | 101 | 2 |
| NC_009620.1 | Sinorhizobium medicae WSM419 | pSMED01 | 1.0E-34 | 95.05 | 98 | 5 |
| NC_009621.1 | Sinorhizobium medicae WSM419 | pSMED02 | 4.0E-34 | 95 | 99 | 12 |
| NC_009622.1 | Sinorhizobium medicae WSM419 | pSMED03 | 2.0E-35 | 95.05 | 101 | 11 |
| NC_020527.1 | Sinorhizobium meliloti 2011 | pSymA | 2.0E-37 | 97.03 | 101 | 2 |
| NC_020560.1 | Sinorhizobium meliloti 2011 | pSymB | 3.0E-36 | 96.04 | 101 | 3 |
| NC_015597.1 | Sinorhizobium meliloti AK83 | pSINME01 | 1.0E-37 | 97 | 100 | 1 |
| NC_015592.1 | Sinorhizobium meliloti AK83 | pSINME02 | 1.0E-37 | 97.03 | 100 | 3 |
| NC_017324.1 | Sinorhizobium meliloti BL225C | pSINMEB01 | 1.0E-34 | 95.05 | 101 | 4 |
| NC_017323.1 | Sinorhizobium meliloti BL225C | pSINMEB02 | 1.0E-34 | 95.05 | 101 | 4 |
| NC_019846.1 | Sinorhizobium meliloti GR4 | pRmeGR4a | 5.0E-35 | 95 | 100 | 2 |
| NC_019847.1 | Sinorhizobium meliloti GR4 | pRmeGR4b | 2.0E-34 | 95.05 | 96 | 8 |
| NC_019848.1 | Sinorhizobium meliloti GR4 | pRmeGR4c | 4.0E-34 | 95 | 100 | 1 |
| NC_019313.1 | Sinorhizobium meliloti | pHRC017 | 2.0E-36 | 96 | 100 | 3 |
| NC_010865.1 | Sinorhizobium meliloti | pSmeSM11b | 5.0E-35 | 95 | 99 | 17 |
| NC_018682.1 | Sinorhizobium meliloti Rm41 | pRM41A | 2.0E-35 | 95.05 | 101 | 1 |
| NC_018683.1 | Sinorhizobium meliloti Rm41 | pSYMA | 1.0E-34 | 95.05 | 101 | 3 |
| NC_018701.1 | Sinorhizobium meliloti Rm41 | pSYMB | 2.0E-31 | 96.67 | 90 | 1 |
| NC_017327.1 | Sinorhizobium meliloti SM11 | pSmeSM11c | 1.0E-34 | 95.05 | 99 | 4 |
| NC_017326.1 | Sinorhizobium meliloti SM11 | pSmeSM11d | 2.0E-33 | 95.05 | 94 | 3 |
| NC_021209.1 | Sinorhizobium sp. M14 | pSinA | 5.0E-33 | 95 | 90 | 233 |
| NC_015595.1 | Sphingobium chlorophenolicum L-1 | pSPHCH01 | 4.0E-41 | 99.01 | 101 | 3 |
| NC_016000.1 | Sphingobium chungbukense strain DJ77 | pSY2 | 1.0E-43 | 100 | 101 | 1 |
| NC_019376.1 | Sphingobium fuliginis ATCC 27551 | pPDL2 | 3.0E-43 | 100 | 101 | 1 |
| NC_014007.1 | Sphingobium japonicum UT26S | pCHQ1 | 2.0E-35 | 96 | 93 | 4 |
| NC_014005.1 | Sphingobium japonicum UT26S | pUT1 | 2.0E-43 | 100 | 101 | 1 |
| NC_015974.1 | Sphingobium sp. SYK-6 | pSLPG | 1.0E-42 | 100 | 101 | 2 |
| NC_008246.1 | Sphingobium yanoikuyae | pYAN-1 DNA | 6.0E-36 | 97.8 | 91 | 1 |
| NC_007353.2 | Sphingomonas sp. A1 | pA1 DNA | 8.0E-39 | 97.03 | 101 | 21 |
| NC_008308.1 | Sphingomonas sp. KA1 | pCAR3 DNA | 1.0E-39 | 98.02 | 101 | 2 |
| NC_020542.1 | Sphingomonas sp. MM-1 | pISP0 | 7.0E-36 | 95.96 | 99 | 2 |
| NC_020562.1 | Sphingomonas sp. MM-1 | pISP1 | 1.0E-35 | 95.05 | 101 | 15 |
| NC_020543.1 | Sphingomonas sp. MM-1 | pISP2 | 1.0E-37 | 96.04 | 101 | 3 |
| NC_020544.1 | Sphingomonas sp. MM-1 | pISP3 | 7.0E-39 | 97.03 | 101 | 1 |
| NC_009507.1 | Sphingomonas wittichii RW1 | pSWIT01 | 5.0E-32 | 95.65 | 92 | 5 |
| NC_009508.1 | Sphingomonas wittichii RW1 | pSWIT02 | 7.0E-35 | 95.05 | 95 | 4 |
| NC_021657.1 | Staphylococcus aureus Bmb9393 | pBmb9393 | 1.0E-42 | 99.01 | 101 | 1 |
| NC_018974.1 | Staphylococcus aureus | p18811-P03 | 2.0E-43 | 100 | 101 | 1 |
| NC_002013.1 | Staphylococcus aureus | pC194 | 1.0E-42 | 99.01 | 101 | 1 |
| NC_013319.1 | Staphylococcus aureus | pI258 | 2.0E-37 | 99.01 | 90 | 25 |
| NC_007931.1 | Staphylococcus aureus | pSA1379 | 2.0E-43 | 100 | 101 | 1 |
| NC_013348.1 | Staphylococcus aureus | pSK156 | 4.0E-43 | 100 | 101 | 2 |
| NC_013322.1 | Staphylococcus aureus | SAP019A | 8.0E-43 | 100 | 100 | 1 |
| NC_013332.1 | Staphylococcus aureus | SAP052A | 1.0E-41 | 99.01 | 101 | 1 |
| NC_021230.1 | Staphylococcus aureus strain 1 | pSA8589 | 6.0E-44 | 100 | 101 | 1 |
| NC_022126.1 | Staphylococcus aureus subsp. aureus 55/2053 |  | 7.0E-42 | 99.01 | 101 | 3 |
| NC_002774.1 | Staphylococcus aureus subsp. aureus Mu50 | VRSAp | 2.0E-43 | 100 | 101 | 5 |
| NC_003140.1 | Staphylococcus aureus subsp. aureus N315 | pN315 | 2.0E-37 | 96 | 100 | 1 |
| NC_022598.1 | Staphylococcus aureus TY825 | pETB DNA | 5.0E-43 | 100 | 101 | 1 |
| NC_005005.1 | Staphylococcus epidermidis ATCC 12228 | pSE-12228-04 | 1.0E-43 | 100 | 101 | 1 |
| NC_013393.1 | Staphylococcus epidermidis | pSK105 | 3.0E-41 | 99.01 | 101 | 10 |
| NC_022618.1 | Staphylococcus epidermidis | pSWS47 | 2.0E-43 | 100 | 101 | 1 |
| NC_013374.1 | Staphylococcus epidermidis | SAP045A | 3.0E-43 | 100 | 101 | 1 |
| NC_006663.1 | Staphylococcus epidermidis RP62A | pSERP | 2.0E-43 | 100 | 101 | 2 |
| NC_007169.1 | Staphylococcus haemolyticus JCSC1435 | pSHaeA | 2.0E-37 | 95.05 | 101 | 1 |
| NC_020237.1 | Staphylococcus hyicus | pSTE1 | 4.0E-42 | 99.01 | 101 | 3 |
| NC_007352.1 | Staphylococcus saprophyticus subsp. saprophyticus ATCC 15305 | pSSP2 | 2.0E-43 | 100 | 101 | 1 |
| NC_015432.1 | Staphylococcus saprophyticus subsp. saprophyticus MS1146 | pSSAP1 | 6.0E-36 | 95.05 | 101 | 3 |
| NC_005076.1 | Staphylococcus sciuri pSCFS1 |  | 3.0E-39 | 97.03 | 101 | 1 |
| NC_020274.1 | Staphylococcus warneri SG1 | clone pvSw1 | 3.0E-38 | 96.97 | 99 | 1 |
| NC_020264.1 | Staphylococcus warneri SG1 | clone pvSw2 | 1.0E-39 | 97.03 | 101 | 1 |
| NC_020265.1 | Staphylococcus warneri SG1 | clone pvSw3 | 6.0E-40 | 97.03 | 101 | 1 |
| NC_001797.1 | Streptococcus agalactiae | pGB354 | 3.0E-41 | 98.02 | 99 | 11 |
| NC_002136.1 | Streptococcus agalactiae | pGB3634 | 7.0E-36 | 98.9 | 90 | 4 |
| NC_015219.1 | Streptococcus gallolyticus subsp. gallolyticus ATCC BAA-2069 | pSGG1 | 2.0E-37 | 96.04 | 90 | 50 |
| NC_016837.1 | Streptococcus infantarius subsp. infantarius CJ18 | pSICJ18-1 | 2.0E-35 | 95.05 | 96 | 9 |
| NC_019365.1 | Streptococcus infantis | pSI01 | 4.0E-42 | 99.01 | 101 | 7 |
| NC_016750.1 | Streptococcus macedonicus ACA-DC 198 | pSMA198 | 1.0E-36 | 95.05 | 93 | 27 |
| NC_006979.1 | Streptococcus pyogenes | pSM19035 | 8.0E-37 | 95.96 | 97 | 11 |
| NC_012923.1 | Streptococcus suis BM407 | pBM407 | 4.0E-39 | 97.03 | 101 | 8 |
| NC_005322.1 | Streptococcus thermophilus | pSMQ308 | 3.0E-41 | 98.02 | 101 | 1 |
| NC_005323.1 | Streptococcus thermophilus SMQ-173 | pSMQ173b | 2.0E-42 | 99.01 | 101 | 2 |
| NC_000937.1 | Streptococcus thermophilus ST135 | pER35 | 2.0E-39 | 97.03 | 101 | 6 |
| NC_015257.1 | Tetragenococcus halophilus | pHDC-I DNA | 2.0E-37 | 96.94 | 98 | 1 |
| NC_019354.1 | Tetragenococcus muriaticus | pHDC-I-1 | 2.0E-43 | 100 | 101 | 1 |
| NC_011667.1 | Thauera sp. MZ1T | pTha01 | 1.0E-32 | 95.05 | 90 | 51 |
| NC_011961.1 | Thermomicrobium roseum DSM 5159 | unnamed | 8.0E-35 | 95.05 | 97 | 3 |
| NC_014154.1 | Thiomonas intermedia K12 | pTINT01 | 3.0E-32 | 95 | 90 | 540 |
| NC_014155.1 | thiomonas intermedia K12 | pTINT02 | 3.0E-39 | 97.03 | 97 | 93 |
| NC_017966.1 | Tistrella mobilis KA081020-065 | pTM2 | 6.0E-35 | 95.05 | 100 | 6 |
| NC_017958.1 | Tistrella mobilis KA081020-065 | pTM3 | 2.0E-30 | 95 | 90 | 1785 |
| NC_017959.1 | Tistrella mobilis KA081020-065 | pTM4 | 2.0E-31 | 95 | 90 | 65 |
| NC_005206.1 | Trueperella pyogenes | pAP2 | 7.0E-44 | 100 | 101 | 2 |
| NC_006385.1 | Uncultured bacterium activated sludge | pRSB101 | 1.0E-35 | 95 | 99 | 55 |
| NC_006352.1 | Uncultured bacterium activated sludge | pTB11 | 5.0E-37 | 97.03 | 90 | 36 |
| NC_019299.1 | Uncultured bacterium HH1107 | pHH1107 | 2.0E-35 | 95.05 | 93 | 185 |
| NC_019217.1 | Uncultured bacterium HHV216 | pHHV216 | 8.0E-34 | 95 | 90 | 263 |
| NC_019218.1 | Uncultured bacterium HHV35 | pHHV35 | 1.0E-31 | 95 | 90 | 324 |
| NC_008272.1 | Uncultured bacterium IncP-1 | pKJK5 | 5.0E-36 | 95.05 | 101 | 5 |
| NC_007502.1 | Uncultured bacterium IncP-1beta multiresistance | pB8 | 4.0E-37 | 96 | 95 | 36 |
| NC_008055.1 | Uncultured bacterium IncP-1gamma | QKH54 | 4.0E-32 | 95 | 90 | 697 |
| NC_004840.1 | Uncultured bacterium | pB10 | 1.0E-37 | 96.04 | 91 | 78 |
| NC_019022.1 | Uncultured bacterium | PB11 | 2.0E-36 | 97.87 | 93 | 82 |
| NC_006388.1 | Uncultured bacterium | pB3 | 2.0E-41 | 99.01 | 101 | 13 |
| NC_003430.1 | Uncultured bacterium | pB4 | 9.0E-35 | 95.05 | 90 | 303 |
| NC_019020.1 | Uncultured bacterium | PB5 | 6.0E-36 | 95.05 | 99 | 84 |
| NC_013773.1 | Uncultured bacterium | pGNB2 | 7.0E-44 | 100 | 101 | 2 |
| NC_003213.2 | Uncultured bacterium | pIPO2T | 3.0E-37 | 96.04 | 95 | 6 |
| NC_008330.1 | Uncultured bacterium | pLB1 DNA | 1.0E-37 | 96.04 | 94 | 5 |
| NC_019216.1 | Uncultured bacterium | pRSB105 | 6.0E-35 | 95 | 94 | 73 |
| NC_003122.1 | Uncultured bacterium | pSB102 | 2.0E-35 | 95 | 98 | 37 |
| NC_019021.1 | Uncultured bacterium | PSP21 | 3.0E-33 | 96 | 90 | 8 |
| NC_007680.1 | Uncultured bacterium | pTP6 | 2.0E-34 | 95.05 | 95 | 37 |
| NC_013278.1 | Uncultured bacterium | pTRACA18 | 5.0E-37 | 95.05 | 98 | 3 |
| NC_013279.1 | Uncultured bacterium | pTRACA20 | 7.0E-40 | 97.03 | 101 | 6 |
| NC_013280.1 | Uncultured bacterium | pTRACA22 | 1.0E-33 | 95.05 | 90 | 98 |
| NC_019324.1 | UNVERIFIED: Clostridium sp. MT351 | unnamed | 2.0E-34 | 95.7 | 93 | 5 |
| NC_019352.1 | UNVERIFIED: Leuconostoc mesenteroides subsp. mesenteroides | pMBLT00 | 7.0E-42 | 99.01 | 101 | 2 |
| NC_019320.1 | Variovorax sp. DB1 | pDB1 | 1.0E-31 | 95 | 90 | 25 |
| NC_008771.1 | Verminephrobacter eiseniae EF01-2 | pVEIS01 | 3.0E-36 | 95.05 | 94 | 41 |
| NC_023291.1 | Vibrio cholerae strain BI144 | pVCR94deltaX | 2.0E-37 | 96.04 | 99 | 5 |
| NC_020451.1 | Vibrio coralliilyticus strain ATCC BAA-450 |  | 5.0E-32 | 95 | 90 | 26 |
| NC_009717.1 | Xanthobacter autotrophicus Py2 | pXAUT01 | 6.0E-31 | 95.05 | 90 | 49 |
| NC_003921.3 | Xanthomonas axonopodis pv. citri str. 306 | pXAC33 | 3.0E-36 | 95.05 | 99 | 11 |
| NC_020801.1 | Xanthomonas axonopodis Xac29-1 | pXAC33 | 5.0E-39 | 97.03 | 101 | 8 |
| NC_002490.1 | Xylella fastidiosa 9a5c | pXF51 | 8.0E-33 | 95.65 | 92 | 1 |
| NC_012208.1 | Yersinia enterocolitica | pYe4449-1 | 4.0E-44 | 100 | 101 | 2 |
| NC_010377.1 | Yersinia enterocolitica | pYE854 | 3.0E-41 | 99.01 | 101 | 1 |
| NC_019269.1 | Yersinia frederiksenii | pYF27601 | 5.0E-44 | 100 | 101 | 2 |
| NC_009139.1 | Yersinia ruckeri YR71 | pYR1 | 6.0E-40 | 98.02 | 101 | 1 |
| NC_022903.1 | Zymomonas mobilis subsp. mobilis str. CP4 = NRRL B-14023 |  | 3.0E-36 | 95.05 | 101 | 2 |
| NC_015716.1 | Zymomonas mobilis subsp. pomaceae ATCC 29192 | pZYMOP02 | 1.0E-35 | 95.88 | 97 | 2 |

**Assigned contigs of sample C1755 against the RefSeq plasmid database**

(Sorted by Organism)

| **Accession number** | **Organism** | **Plasmid** | **E value ≤** | **Identity (%) ≥** | **Hit length (bp) ≥** | **Number of reads** |
| --- | --- | --- | --- | --- | --- | --- |
| NC_005793.2 | Achromobacter denitrificans | pEST4011 | 4.0E-67 | 99.32 | 147 | 3 |
| NC_022242.1 | Achromobacter xylosoxidans subsp. denitrificans | pAX22 | 8.0E-135 | 97.88 | 283 | 3 |
| NC_008766.1 | Acidovorax sp. JS42 | pAOVO02 | < 1.0E-150 | 100 | 3168 | 1 |
| NC_010605.1 | Acinetobacter baumannii ACICU | pACICU1 | 1.0E-74 | 97.06 | 170 | 2 |
| NC_009084.1 | Acinetobacter baumannii ATCC 17978 | pAB2 | < 1.0E-150 | 96.81 | 533 | 1 |
| NC_010404.1 | Acinetobacter baumannii AYE | p3ABAYE | 1.0E-43 | 95.36 | 109 | 11 |
| NC_021734.1 | Acinetobacter baumannii BJAB0715 | pBJAB0715 | 1.0E-72 | 97.46 | 154 | 5 |
| NC_017848.1 | Acinetobacter baumannii MDR-TJ | pABTJ1 | < 1.0E-150 | 99.66 | 1194 | 1 |
| NC_010481.1 | Acinetobacter baumannii | pABIR | 2.0E-35 | 96.77 | 93 | 5 |
| NC_013277.1 | Acinetobacter baumannii | pMMA2 | < 1.0E-150 | 97.08 | 616 | 1 |
| NC_013506.1 | Acinetobacter baumannii | pMMCU2 | < 1.0E-150 | 98.12 | 426 | 1 |
| NC_019280.1 | Acinetobacter baumannii | pMMD | < 1.0E-150 | 95.3 | 681 | 1 |
| NC_019345.1 | Acinetobacter baumannii | pRAY*-v2 | < 1.0E-150 | 99.67 | 612 | 1 |
| NC_010398.1 | Acinetobacter baumannii SDF | p3ABSDF | 4.0E-62 | 96.65 | 139 | 2 |
| NC_020818.1 | Acinetobacter baumannii strain GF216 | pNDM-AB | < 1.0E-150 | 97.08 | 471 | 2 |
| NC_017166.1 | Acinetobacter baumannii TCDC-AB0715 | p2ABTCDC0715 | < 1.0E-150 | 99.36 | 1092 | 1 |
| NC_010309.1 | Acinetobacter venetianus strain VE-C3 | pAV1 | 2.0E-73 | 95.59 | 154 | 4 |
| NC_010310.1 | Acinetobacter venetianus strain VE-C3 | pAV2 | 5.0E-115 | 96.25 | 254 | 5 |
| NC_005312.1 | Actinobacillus pleuropneumoniae | pMS260 | 7.0E-135 | 99.26 | 272 | 1 |
| NC_006143.1 | Aeromonas caviae | pFBAOT6 | 1.0E-54 | 95.44 | 121 | 24 |
| NC_019380.1 | Aeromonas hydrophila | pR148 | < 1.0E-150 | 98.31 | 414 | 1 |
| NC_012885.1 | Aeromonas hydrophila | pRA1 | < 1.0E-150 | 96.42 | 419 | 1 |
| NC_003124.1 | Aeromonas salmonicida | pRAS3.2 | 2.0E-61 | 96.13 | 132 | 6 |
| NC_009349.1 | Aeromonas salmonicida subsp. salmonicida A449 | 4 | 7.0E-87 | 95.31 | 184 | 2 |
| NC_011982.1 | Agrobacterium vitis S4 | pTiS4 | 2.0E-53 | 96.24 | 133 | 2 |
| NC_014908.1 | Alicycliphilus denitrificans BC | pALIDE01 | 5.0E-132 | 98.26 | 269 | 3 |
| NC_014911.1 | Alicycliphilus denitrificans BC | pALIDE02 | 9.0E-120 | 98.41 | 251 | 6 |
| NC_008712.1 | Arthrobacter aurescens TC1 | TC1 | < 1.0E-150 | 99.89 | 449 | 2 |
| NC_013963.1 | Bacillus sp. BS-01 | pBS-01 | < 1.0E-150 | 99.96 | 5267 | 1 |
| NC_014557.1 | Bacillus sp. BS-02 | pBS-02 | 4.0E-104 | 99.26 | 211 | 2 |
| NC_011332.1 | Bifidobacterium bifidum | pB80 | 2.0E-114 | 100 | 229 | 1 |
| NC_004252.1 | Bifidobacterium longum DJO10A | pDOJH10L | 3.0E-65 | 100 | 139 | 2 |
| NC_002635.1 | Bifidobacterium longum KJ | pKJ36 | 1.0E-71 | 98.71 | 155 | 1 |
| NC_010857.1 | Bifidobacterium longum | p6043A | < 1.0E-150 | 99.57 | 693 | 1 |
| NC_004770.1 | Bifidobacterium longum RW048 | pNAC1 | 2.0E-105 | 99.53 | 215 | 1 |
| NC_019378.1 | Burkholderia cepacia | pIJB1 | < 1.0E-150 | 100 | 341 | 1 |
| NC_016591.1 | Burkholderia sp. YI23 | byi_2p | 2.0E-129 | 99.6 | 260 | 3 |
| NC_009227.1 | Burkholderia vietnamiensis G4 | pBVIE02 | < 1.0E-150 | 98.68 | 1135 | 1 |
| NC_022355.1 | Campylobacter coli CVM N29710 | pN29710-1 | 3.0E-54 | 95.98 | 130 | 2 |
| NC_022354.1 | Campylobacter jejuni subsp. jejuni 00-2544 |  | < 1.0E-150 | 99.49 | 785 | 1 |
| NC_014801.1 | Campylobacter jejuni subsp. jejuni ICDCCJ07001 | pTet | < 1.0E-150 | 99.61 | 514 | 1 |
| NC_022601.1 | Carnobacterium sp. WN1359 | pWNCR12 | 8.0E-48 | 96.58 | 117 | 3 |
| NC_022602.1 | Carnobacterium sp. WN1359 | pWNCR47 | 2.0E-80 | 95.96 | 183 | 3 |
| NC_022603.1 | Carnobacterium sp. WN1359 | pWNCR64 | 1.0E-86 | 97.89 | 190 | 2 |
| NC_020123.1 | Citrobacter freundii strain CFSTE | pT-OXA-181 | 1.0E-137 | 100 | 274 | 1 |
| NC_021077.1 | Comamonas sp. 7D-2 | pBHB | < 1.0E-150 | 96.67 | 439 | 2 |
| NC_010935.1 | Comamonas testosteroni CNB-1 | pCNB | < 1.0E-150 | 99.81 | 1076 | 1 |
| NC_014167.1 | Corynebacterium resistens DSM 45100 | pJA144188 | < 1.0E-150 | 98.32 | 833 | 1 |
| NC_006525.1 | Cupriavidus metallidurans CH34 | pMOL28 | < 1.0E-150 | 97.96 | 1326 | 1 |
| NC_019312.1 | Delftia sp. KV29 | pKV29 | 2.0E-127 | 98.61 | 258 | 6 |
| NC_009957.1 | Dinoroseobacter shibae DFL 12 | pDSHI03 | < 1.0E-150 | 99.67 | 920 | 1 |
| NC_001735.4 | Enterobacter aerogenes | R751 | < 1.0E-150 | 100 | 348 | 1 |
| NC_017097.1 | Enterobacter cloacae | pUL3AT | < 1.0E-150 | 97.37 | 419 | 1 |
| NC_021087.1 | Enterobacter cloacae strain M15 |  | < 1.0E-150 | 100 | 521 | 1 |
| NC_014107.1 | Enterobacter cloacae subsp. cloacae ATCC 13047 | pECL_A | < 1.0E-150 | 97.58 | 1732 | 1 |
| NC_013514.1 | Enterococcus faecalis | pAMbeta1 | 3.0E-83 | 96.81 | 188 | 1 |
| NC_014475.1 | Enterococcus faecalis | pWZ1668 | 4.0E-109 | 95.29 | 226 | 2 |
| NC_008445.1 | Enterococcus faecalis RE25 | pRE25 | < 1.0E-150 | 97.93 | 360 | 2 |
| NC_021987.1 | Enterococcus faecium Aus0085 | p1 | 1.0E-139 | 99.64 | 281 | 1 |
| NC_021988.1 | Enterococcus faecium Aus0085 | p3 | < 1.0E-150 | 96.77 | 334 | 2 |
| NC_017962.1 | Enterococcus faecium DO | 2 | < 1.0E-150 | 98.89 | 359 | 1 |
| NC_017963.1 | Enterococcus faecium DO | 3 | < 1.0E-150 | 97.52 | 363 | 1 |
| NC_020208.1 | Enterococcus faecium NRRL B-2354 | pNB2354_1 | 1.0E-111 | 97.14 | 245 | 3 |
| NC_013317.1 | Enterococcus faecium | p5753cA | < 1.0E-150 | 99.16 | 384 | 2 |
| NC_021170.1 | Enterococcus faecium | pF856 | < 1.0E-150 | 96.17 | 444 | 1 |
| NC_014959.1 | Enterococcus faecium | pS177 | < 1.0E-150 | 96.4 | 639 | 2 |
| NC_010558.1 | Escherichia coli 1520 | pIP1206 | < 1.0E-150 | 100 | 836 | 1 |
| NC_019066.1 | Escherichia coli | pAPEC1990_61 | < 1.0E-150 | 99.55 | 1323 | 1 |
| NC_005327.1 | Escherichia coli | pC15-1a | 2.0E-136 | 100 | 272 | 1 |
| NC_019049.1 | Escherichia coli | pCM959 | 8.0E-74 | 97.41 | 159 | 2 |
| NC_014615.1 | Escherichia coli | pETN48 | < 1.0E-150 | 95.35 | 387 | 1 |
| NC_014231.1 | Escherichia coli | pKC394 | < 1.0E-150 | 99.8 | 507 | 1 |
| NC_019061.1 | Escherichia coli | pPWD4_103 | < 1.0E-150 | 100 | 395 | 1 |
| NC_012780.1 | Eubacterium eligens ATCC 27750 | unnamed | 3.0E-41 | 96 | 108 | 14 |
| NC_023287.1 | Exiguobacterium sp. S3-2 | pMC1 | < 1.0E-150 | 98.7 | 540 | 1 |
| NC_018107.1 | Klebsiella oxytoca E718 | pKOX_R1 | < 1.0E-150 | 99.68 | 315 | 1 |
| NC_017541.1 | Klebsiella pneumoniae KCTC 2242 | pKCTC2242 | < 1.0E-150 | 99.35 | 2609 | 1 |
| NC_021667.1 | Klebsiella pneumoniae | IncA/C-LS6 | < 1.0E-150 | 99.35 | 611 | 1 |
| NC_020087.1 | Klebsiella pneumoniae | pK1HV | < 1.0E-150 | 100 | 305 | 1 |
| NC_019389.1 | Klebsiella pneumoniae | pKDO1 | < 1.0E-150 | 99.82 | 562 | 1 |
| NC_019390.1 | Klebsiella pneumoniae | pKPN_CZ | 2.0E-62 | 95.04 | 149 | 6 |
| NC_014478.1 | Klebsiella pneumoniae | unnamed | < 1.0E-150 | 97.25 | 2367 | 1 |
| NC_019888.1 | Klebsiella pneumoniae strain BK31551 | pBK31551 | < 1.0E-150 | 99.76 | 415 | 1 |
| NC_022609.1 | Klebsiella pneumoniae strain N11-0042 | pKp11-42 | < 1.0E-150 | 96.48 | 511 | 1 |
| NC_017472.1 | Lactobacillus amylovorus GRL1118 | 2 | 2.0E-46 | 95.93 | 106 | 4 |
| NC_004947.1 | Lactobacillus fermentum | pKC5b | 1.0E-128 | 96.14 | 285 | 1 |
| NC_015598.1 | Lactobacillus kefiranofaciens ZW3 | pWW1 | 8.0E-43 | 98.1 | 105 | 1 |
| NC_002799.1 | Lactococcus lactis | pCRL291.1 | 4.0E-79 | 98.82 | 169 | 1 |
| NC_008507.1 | Lactococcus lactis subsp. cremoris SK11 | 5 | < 1.0E-150 | 99.4 | 497 | 1 |
| NC_019430.1 | Lactococcus lactis subsp. cremoris UC509.9 | pCIS8 | < 1.0E-150 | 96.47 | 624 | 1 |
| NC_015901.1 | Lactococcus lactis subsp. lactis bv. diacetylactis | pVF22 | < 1.0E-150 | 98.62 | 723 | 1 |
| NC_017483.1 | Lactococcus lactis subsp. lactis CV56 | pCV56A | 1.0E-122 | 99.5 | 246 | 2 |
| NC_015863.1 | Lactococcus lactis subsp. lactis | pIL5 | < 1.0E-150 | 96.49 | 338 | 3 |
| NC_015864.1 | Lactococcus lactis subsp. lactis | pIL7 | 9.0E-52 | 96.85 | 127 | 1 |
| NC_003383.1 | Listeria innocua Clip11262 | pLI100 | 1.0E-117 | 98.38 | 247 | 1 |
| NC_008242.1 | Mesorhizobium sp. BNC1 | 1 | < 1.0E-150 | 97.09 | 447 | 2 |
| NC_012811.1 | Methylobacterium extorquens AM1 |  | 8.0E-40 | 98.02 | 101 | 4 |
| NC_009669.1 | Ochrobactrum anthropi ATCC 49188 | pOANT01 | 4.0E-52 | 96.15 | 130 | 2 |
| NC_022042.1 | Paracoccus aminophilus JCM 7686 | pAMI1 | 2.0E-68 | 95.65 | 147 | 5 |
| NC_022050.1 | Paracoccus aminophilus JCM 7686 | pAMI8 | 3.0E-86 | 95.64 | 195 | 2 |
| NC_009753.1 | Paracoccus methylutens strain DM12 | pMTH1 | < 1.0E-150 | 99.79 | 450 | 3 |
| NC_017019.1 | Pediococcus claussenii ATCC BAA-344 | pPECL-8 | 4.0E-57 | 95.71 | 140 | 1 |
| NC_008608.1 | Pelobacter propionicus DSM 2379 | pPRO2 | 3.0E-102 | 99.4 | 208 | 3 |
| NC_009739.1 | Pseudomonas aeruginosa | pMATVIM-7 | < 1.0E-150 | 95.49 | 710 | 1 |
| NC_022344.1 | Pseudomonas aeruginosa | pOZ176 | 8.0E-162 | 98.79 | 327 | 4 |
| NC_007100.1 | Pseudomonas aeruginosa | Rms149 | < 1.0E-150 | 100 | 485 | 1 |
| NC_020452.1 | Pseudomonas aeruginosa strain COL-1 | pNOR-2000 | < 1.0E-150 | 95.47 | 772 | 1 |
| NC_022346.1 | Pseudomonas aeruginosa strain ST308 | pCOL-1 | < 1.0E-150 | 95.86 | 349 | 2 |
| NC_021250.1 | Pseudomonas migulae strain D2RT | pD2RT | 1.0E-76 | 98.82 | 169 | 2 |
| NC_011838.1 | Pseudomonas putida | pCAR1.2 | 3.0E-87 | 98.87 | 182 | 4 |
| NC_005244.2 | Pseudomonas sp. ND6 | pND6-1 | 6.0E-143 | 99.31 | 289 | 1 |
| NC_007968.1 | Psychrobacter cryohalolentis K5 | 1 | < 1.0E-150 | 97.78 | 320 | 2 |
| NC_021158.1 | Psychrobacter maritimus | pKLH80 | < 1.0E-150 | 97.8 | 318 | 1 |
| NC_012849.1 | Ralstonia pickettii 12D | pRp12D02 | 9.0E-76 | 97.13 | 174 | 1 |
| NC_019318.1 | Ralstonia pickettii | p712 | 7.0E-114 | 97.73 | 233 | 2 |
| NC_019102.1 | Salmonella enterica subsp. enterica serovar Pullorum | pSPI12 | < 1.0E-150 | 97.81 | 320 | 1 |
| NC_011092.1 | Salmonella enterica subsp. enterica serovar Schwarzengrund str. CVM19633 | pCVM19633_110 | 1.0E-88 | 100 | 184 | 1 |
| NC_021742.1 | Serratia liquefaciens ATCC 27592 |  | 4.0E-64 | 99.29 | 141 | 1 |
| NC_008573.1 | Shewanella sp. ANA-3 | 1 | 4.0E-61 | 96.73 | 137 | 13 |
| NC_021209.1 | Sinorhizobium sp. M14 | pSinA | 1.0E-150 | 95.18 | 338 | 5 |
| NC_014154.1 | Thiomonas intermedia K12 | pTINT01 | 2.0E-42 | 95.47 | 98 | 21 |
| NC_017958.1 | Tistrella mobilis KA081020-065 | pTM3 | 1.0E-49 | 95.11 | 119 | 29 |
| NC_006385.1 | Uncultured bacterium activated sludge | pRSB101 | 1.0E-127 | 99.23 | 260 | 1 |
| NC_019299.1 | Uncultured bacterium HH1107 | pHH1107 | 3.0E-130 | 96.18 | 288 | 1 |
| NC_007502.1 | Uncultured bacterium IncP-1beta multiresistance | pB8 | < 1.0E-150 | 99.28 | 414 | 1 |
| NC_008055.1 | Uncultured bacterium IncP-1gamma | QKH54 | 2.0E-139 | 99.64 | 280 | 1 |
| NC_019022.1 | Uncultured bacterium | PB11 | < 1.0E-150 | 99.28 | 557 | 2 |
| NC_003430.1 | Uncultured bacterium | pB4 | 8.0E-89 | 95.22 | 203 | 7 |
| NC_019020.1 | Uncultured bacterium | PB5 | < 1.0E-150 | 96.33 | 327 | 5 |
| NC_019021.1 | Uncultured bacterium | PSP21 | 3.0E-119 | 99.81 | 240 | 2 |
| NC_013280.1 | Uncultured bacterium | pTRACA22 | < 1.0E-150 | 97.07 | 311 | 5 |
| NC_019324.1 | UNVERIFIED: Clostridium sp. MT351 | unnamed | 6.0E-36 | 95.74 | 94 | 1 |
| NC_020451.1 | Vibrio coralliilyticus strain ATCC BAA-450 |  | 7.0E-40 | 96.12 | 103 | 1 |
| NC_009717.1 | Xanthobacter autotrophicus Py2 | pXAUT01 | < 1.0E-150 | 99.58 | 476 | 2 |

**Assigned high-throughput reads of sample C1756 against the RefSeq plasmid database**

(Sorted by Organism)

| **Accession number** | **Organism** | **Plasmid** | **E value ≤** | **Identity (%) ≥** | **Hit length (bp) ≥** | **Number of reads** |
| --- | --- | --- | --- | --- | --- | --- |
| NC_021992.1 | Acetobacter pasteurianus 386B | Apa386Bp3 | 2.00E-31 | 95.05 | 90 | 6 |
| NC_005793.2 | Achromobacter denitrificans | pEST4011 | 6.00E-38 | 97 | 99 | 104 |
| NC_006830.1 | Achromobacter xylosoxidans A8 | pA81 | 4.00E-42 | 100 | 101 | 2 |
| NC_022242.1 | Achromobacter xylosoxidans subsp. denitrificans | pAX22 | 1.00E-35 | 95.05 | 101 | 18 |
| NC_009468.1 | Acidiphilium cryptum JF-5 | pACRY02 | 3.00E-34 | 95 | 100 | 4 |
| NC_015852.1 | Acidithiobacillus caldus SM-1 | pLAtc1 | 1.00E-37 | 96.04 | 101 | 6 |
| NC_010600.1 | Acidithiobacillus caldus strain MNG | pTcM1 | 4.00E-34 | 95.05 | 96 | 12 |
| NC_008765.1 | Acidovorax sp. JS42 | pAOVO01 | 1.00E-34 | 96.04 | 91 | 34 |
| NC_008766.1 | Acidovorax sp. JS42 | pAOVO02 | 2.00E-41 | 99.01 | 100 | 20 |
| NC_010481.1 | Acinetobacter baumannii | pABIR | 5.00E-35 | 95 | 90 | 185 |
| NC_012813.1 | Acinetobacter baumannii | pABVA01 | 1.00E-37 | 96.04 | 96 | 15 |
| NC_013277.1 | Acinetobacter baumannii | pMMA2 | 2.00E-35 | 95 | 92 | 29 |
| NC_013506.1 | Acinetobacter baumannii | pMMCU2 | 5.00E-36 | 95.05 | 93 | 36 |
| NC_016977.1 | Acinetobacter baumannii | pTS236 | 3.00E-38 | 96.04 | 101 | 2 |
| NC_019280.1 | Acinetobacter baumannii | pMMD | 1.00E-31 | 95.05 | 90 | 123 |
| NC_019345.1 | Acinetobacter baumannii | pRAY*-v2 | 4.00E-36 | 96.04 | 91 | 102 |
| NC_022565.1 | Acinetobacter baumannii 107m | p1ABIBUN | 2.00E-34 | 97.78 | 90 | 6 |
| NC_017164.1 | Acinetobacter baumannii 1656-2 | ABKp2 | 4.00E-36 | 95.05 | 91 | 14 |
| NC_017163.1 | Acinetobacter baumannii 1656-2 | ABKp1 | 1.00E-29 | 95.05 | 90 | 341 |
| NC_006877.1 | Acinetobacter baumannii 19606 | pMAC | 7.00E-34 | 95.05 | 92 | 22 |
| NC_010605.1 | Acinetobacter baumannii ACICU | pACICU1 | 9.00E-32 | 95.05 | 90 | 114 |
| NC_009083.1 | Acinetobacter baumannii ATCC 17978 | pAB1 | 2.00E-36 | 95.96 | 99 | 8 |
| NC_009084.1 | Acinetobacter baumannii ATCC 17978 | pAB2 | 1.00E-37 | 96.04 | 100 | 12 |
| NC_010401.1 | Acinetobacter baumannii AYE | p1ABAYE | 3.00E-36 | 95.05 | 99 | 42 |
| NC_010402.1 | Acinetobacter baumannii AYE | p2ABAYE | 4.00E-43 | 100 | 101 | 4 |
| NC_010404.1 | Acinetobacter baumannii AYE | p3ABAYE | 1.00E-29 | 95 | 90 | 628 |
| NC_021727.1 | Acinetobacter baumannii BJAB07104 | p1BJAB07104 | 3.00E-35 | 95.05 | 91 | 8 |
| NC_021734.1 | Acinetobacter baumannii BJAB0715 | pBJAB0715 | 2.00E-31 | 95.05 | 93 | 375 |
| NC_020525.1 | Acinetobacter baumannii D1279779 | pD1279779 | 9.00E-38 | 96.04 | 94 | 14 |
| NC_017848.1 | Acinetobacter baumannii MDR-TJ | pABTJ1 | 3.00E-30 | 95.05 | 90 | 57 |
| NC_020524.1 | Acinetobacter baumannii MDR-TJ | pABTJ2 | 2.00E-34 | 95.05 | 92 | 79 |
| NC_017172.1 | Acinetobacter baumannii MDR-ZJ06 | pMDR-ZJ06 | 8.00E-37 | 96 | 90 | 99 |
| NC_010395.1 | Acinetobacter baumannii SDF | p1ABSDF | 3.00E-36 | 95.05 | 101 | 4 |
| NC_010396.1 | Acinetobacter baumannii SDF | p2ABSDF | 4.00E-35 | 95 | 94 | 65 |
| NC_010398.1 | Acinetobacter baumannii SDF | p3ABSDF | 2.00E-34 | 95.05 | 91 | 36 |
| NC_020818.1 | Acinetobacter baumannii strain GF216 | pNDM-AB | 1.00E-32 | 95.05 | 90 | 153 |
| NC_019985.2 | Acinetobacter baumannii strain ZW85-1 | pAbNDM-1 | 3.00E-34 | 95.1 | 90 | 122 |
| NC_017166.1 | Acinetobacter baumannii TCDC-AB0715 | p2ABTCDC0715 | 3.00E-42 | 100 | 101 | 8 |
| NC_023031.1 | Acinetobacter baumannii ZW85-1 | ZW85p2 | 2.00E-33 | 95 | 90 | 114 |
| NC_023322.1 | Acinetobacter bereziniae strain CHI-40-1 | pNDM-BJ01 | 4.00E-38 | 97.03 | 101 | 1 |
| NC_013056.1 | Acinetobacter calcoaceticus strain Acal H12O-07 | pMMCU1 | 2.00E-34 | 95.15 | 95 | 3 |
| NC_019268.1 | Acinetobacter lwoffii | pNDM-BJ01 | 9.00E-41 | 99.01 | 101 | 6 |
| NC_019281.1 | Acinetobacter lwoffii | pNDM-BJ02 | 4.00E-38 | 97.03 | 99 | 3 |
| NC_019323.1 | Acinetobacter lwoffii | pABZ78 | 1.00E-33 | 96.04 | 91 | 28 |
| NC_023280.1 | Acinetobacter nosocomialis | pRAY*-v3 | 7.00E-32 | 95.65 | 92 | 20 |
| NC_019322.1 | Acinetobacter pittii | pABCA95 | 6.00E-38 | 96.04 | 100 | 132 |
| NC_000923.1 | Acinetobacter sp. SUN resistance | pRAY | 7.00E-38 | 96.04 | 101 | 4 |
| NC_010309.1 | Acinetobacter venetianus strain VE-C3 | pAV1 | 2.00E-35 | 95.05 | 94 | 89 |
| NC_010310.1 | Acinetobacter venetianus strain VE-C3 | pAV2 | 3.00E-34 | 95.05 | 94 | 247 |
| NC_003125.1 | Actinobacillus pleuropneumoniae | pTYM1 | 8.00E-38 | 99.01 | 101 | 12 |
| NC_010795.1 | Actinobacillus pleuropneumoniae | pHB0503 | 7.00E-43 | 100 | 101 | 14 |
| NC_005312.1 | Actinobacillus pleuropneumoniae | pMS260 | 4.00E-43 | 100 | 101 | 2 |
| NC_007099.1 | Actinobacillus pleuropneumoniae | pPSAS1522 | 7.00E-44 | 100 | 101 | 2 |
| NC_007098.1 | Actinobacillus pleuropneumoniae | pKMA2425 | 1.00E-43 | 100 | 101 | 10 |
| NC_007097.1 | Actinobacillus porcitonsillarum | pKMA757 | 8.00E-44 | 100 | 101 | 1 |
| NC_009476.1 | Aeromonas bestiarum 5S9 | pAb5S9 | 1.00E-42 | 100 | 101 | 2 |
| NC_006143.1 | Aeromonas caviae | pFBAOT6 | 2.00E-32 | 95.05 | 90 | 371 |
| NC_010919.1 | Aeromonas hydrophila | pRA3 | 4.00E-38 | 97.96 | 91 | 4 |
| NC_011207.1 | Aeromonas hydrophila | pBRST7.6 | 2.00E-39 | 99.01 | 94 | 4 |
| NC_012885.1 | Aeromonas hydrophila | pRA1 | 3.00E-40 | 99.01 | 101 | 6 |
| NC_013780.1 | Aeromonas hydrophila | pAH3680 | 2.00E-35 | 96.81 | 94 | 10 |
| NC_016852.1 | Aeromonas hydrophila | pAHH01 | 5.00E-42 | 100 | 99 | 2 |
| NC_021159.1 | Aeromonas hydrophila | pAhy2.5 | 6.00E-41 | 98.02 | 101 | 6 |
| NC_003124.1 | Aeromonas salmonicida | pRAS3.2 | 1.00E-37 | 96.04 | 101 | 21 |
| NC_003123.1 | Aeromonas salmonicida subsp. salmonicida | pRAS3.1 | 1.00E-38 | 97.03 | 101 | 2 |
| NC_004340.1 | Aeromonas salmonicida subsp. Salmonicida | pAsal3 | 1.00E-40 | 98.02 | 101 | 2 |
| NC_009352.2 | Aeromonas salmonicida subsp. salmonicida | pAsa6 | 2.00E-38 | 97.03 | 101 | 8 |
| NC_009349.1 | Aeromonas salmonicida subsp. salmonicida A449 | 4 | 8.00E-35 | 95.05 | 90 | 116 |
| NC_009350.1 | Aeromonas salmonicida subsp. salmonicida A449 | 5 | 3.00E-38 | 98.02 | 100 | 12 |
| NC_016611.1 | Aeromonas sobria | pAQ2-1 | 2.00E-40 | 98.02 | 101 | 2 |
| NC_002579.1 | Aggregatibacter actinomycetemcomitans | pVT745 | 1.00E-41 | 100 | 99 | 2 |
| NC_003064.2 | Agrobacterium fabrum str. C58 | At | 3.00E-34 | 95.05 | 101 | 4 |
| NC_002575.1 | Agrobacterium rhizogenes | pRi1724 | 1.00E-34 | 95.05 | 101 | 10 |
| NC_015184.1 | Agrobacterium sp. H13-3 | pAspH13-3a | 1.00E-33 | 95 | 92 | 11 |
| NC_002377.1 | Agrobacterium tumefaciens | Ti | 2.00E-36 | 96.04 | 101 | 4 |
| NC_019555.1 | Agrobacterium tumefaciens | pAoF64/95 | 3.00E-40 | 99.01 | 101 | 2 |
| NC_006277.2 | Agrobacterium tumefaciens K84 | pAgK84 | 2.00E-35 | 95.05 | 101 | 2 |
| NC_002147.1 | Agrobacterium tumefaciens MAFF301001 | pTi-SAKURA | 1.00E-34 | 95.05 | 101 | 13 |
| NC_010929.1 | Agrobacterium tumefaciens Ti | pTiBo542 | 4.00E-34 | 95 | 100 | 2 |
| NC_011982.1 | Agrobacterium vitis S4 | pTiS4 | 4.00E-34 | 95 | 99 | 26 |
| NC_011984.1 | Agrobacterium vitis S4 | pAtS4c | 4.00E-33 | 95 | 92 | 5 |
| NC_011986.1 | Agrobacterium vitis S4 | pAtS4a | 2.00E-39 | 98.99 | 99 | 4 |
| NC_011991.1 | Agrobacterium vitis S4 | pAtS4b | 1.00E-37 | 97.03 | 99 | 6 |
| NC_014908.1 | Alicycliphilus denitrificans BC | pALIDE01 | 1.00E-30 | 95.56 | 90 | 30 |
| NC_014911.1 | Alicycliphilus denitrificans BC | pALIDE02 | 4.00E-35 | 95.05 | 97 | 38 |
| NC_015423.1 | Alicycliphilus denitrificans K601 | pALIDE201 | 3.00E-36 | 96.94 | 98 | 18 |
| NC_013164.1 | Anaerococcus prevotii DSM 20548 | pAPRE01 | 3.00E-32 | 95 | 93 | 109 |
| NC_008712.1 | Arthrobacter aurescens TC1 | TC1 | 4.00E-36 | 96.04 | 91 | 2 |
| NC_011881.1 | Arthrobacter chlorophenolicus A6 | pACHL02 | 2.00E-36 | 96.04 | 99 | 2 |
| NC_015146.1 | Arthrobacter phenanthrenivorans Sphe3 | pASPHE301 | 2.00E-36 | 96.04 | 101 | 2 |
| NC_006823.1 | Azoarcus sp. EbN1 | 1 | 1.00E-34 | 95.05 | 97 | 52 |
| NC_006824.1 | Azoarcus sp. EbN1 | 2 | 1.00E-34 | 95.05 | 101 | 4 |
| NC_016594.1 | Azospirillum brasilense Sp245 | AZOBR_p1 | 2.00E-29 | 95 | 90 | 16 |
| NC_016596.1 | Azospirillum brasilense Sp245 | AZOBR_p4 | 1.00E-33 | 95 | 98 | 12 |
| NC_016618.1 | Azospirillum brasilense Sp245 | AZOBR_p2 | 2.00E-32 | 95.79 | 93 | 8 |
| NC_016623.1 | Azospirillum lipoferum 4B | AZO_p3 | 6.00E-31 | 96.67 | 90 | 2 |
| NC_016624.1 | Azospirillum lipoferum 4B | AZO_p5 | 5.00E-30 | 95 | 90 | 44 |
| NC_016585.1 | Azospirillum lipoferum 4B | AZO_p1 | 7.00E-32 | 95.05 | 90 | 40 |
| NC_013855.1 | Azospirillum sp. B510 | pAB510a | 1.00E-30 | 95.65 | 92 | 8 |
| NC_013856.1 | Azospirillum sp. B510 | pAB510b | 1.00E-32 | 95.05 | 95 | 2 |
| NC_013857.1 | Azospirillum sp. B510 | pAB510c | 2.00E-32 | 95.05 | 94 | 10 |
| NC_013858.1 | Azospirillum sp. B510 | pAB510d | 2.00E-31 | 95.7 | 93 | 2 |
| NC_013859.1 | Azospirillum sp. B510 | pAB510e | 3.00E-30 | 95 | 90 | 28 |
| NC_005707.1 | Bacillus cereus ATCC 10987 | pBc10987 | 9.00E-42 | 100 | 101 | 3 |
| NC_018492.1 | Bacillus cereus FRI-35 | p01 | 8.00E-41 | 99.01 | 101 | 3 |
| NC_016773.1 | Bacillus cereus NC7401 | pNC2 | 9.00E-43 | 100 | 100 | 4 |
| NC_011973.1 | Bacillus cereus Q1 | pBc239 | 5.00E-39 | 98.02 | 101 | 9 |
| NC_010924.1 | Bacillus cereus strain AH187 | pCER270 | 1.00E-34 | 95.92 | 98 | 14 |
| NC_010933.1 | Bacillus cereus strain G9241 | pBC210 | 2.00E-39 | 98.02 | 101 | 1 |
| NC_004604.2 | Bacillus megaterium QM B1551 | pBM400 | 9.00E-29 | 95 | 90 | 4674 |
| NC_017139.1 | Bacillus megaterium WSH-002 | WSH-002_p1 | 8.00E-31 | 95.05 | 91 | 64 |
| NC_013792.1 | Bacillus pseudofirmus OF4 | pBpOF4-01 | 3.00E-36 | 96.04 | 101 | 6 |
| NC_013963.1 | Bacillus sp. BS-01 | pBS-01 | 1.00E-39 | 98.02 | 98 | 315 |
| NC_014557.1 | Bacillus sp. BS-02 | pBS-02 | 2.00E-37 | 98.94 | 94 | 53 |
| NC_014172.1 | Bacillus thuringiensis BMB171 | pBMB171 | 1.00E-41 | 100 | 101 | 2 |
| NC_014937.1 | Bacillus thuringiensis CT43 | pBMB0558 | 1.00E-29 | 95 | 90 | 174 |
| NC_018486.1 | Bacillus thuringiensis HD-771 | p01 | 7.00E-42 | 100 | 101 | 1 |
| NC_017201.1 | Bacillus thuringiensis serovar finitimus YBT-020 | pBMB26 | 4.00E-39 | 98.02 | 101 | 1 |
| NC_023074.1 | Bacillus thuringiensis serovar tenebrionis str. YBT-1765 | pBMB165 | 2.00E-39 | 98.02 | 100 | 14 |
| NC_020385.1 | Bacillus thuringiensis serovar thuringiensis str. IS5056 | pIS56-328 | 1.00E-41 | 100 | 101 | 2 |
| NC_019783.1 | Bacterium 36B | pTOR_02 | 5.00E-41 | 99.01 | 101 | 41 |
| NC_019798.1 | Bacterium 72B | pTOR_01 | 9.00E-43 | 100 | 101 | 34 |
| NC_011073.1 | Bacteroides fragilis | pBFP35 | 6.00E-39 | 99.01 | 93 | 74 |
| NC_019534.1 | Bacteroides fragilis | pBFUK1 | 3.00E-34 | 95.05 | 95 | 193 |
| NC_005026.1 | Bacteroides fragilis IB143 | pBI143 | 5.00E-36 | 95.05 | 93 | 67 |
| NC_006873.1 | Bacteroides fragilis NCTC 9343 | pBF9343 | 6.00E-35 | 95 | 96 | 135 |
| NC_006297.1 | Bacteroides fragilis YCH46 | pBFY46 | 2.00E-35 | 96.04 | 91 | 12 |
| NC_015166.1 | Bacteroides salanitronis DSM 18170 | pBACSA03 | 3.00E-36 | 95.05 | 101 | 12 |
| NC_004703.1 | Bacteroides thetaiotaomicron VPI-5482 | p5482 | 4.00E-37 | 96.04 | 98 | 116 |
| NC_011332.1 | Bifidobacterium bifidum | pB80 | 1.00E-40 | 98.02 | 96 | 170 |
| NC_007068.1 | Bifidobacterium catenulatum | pBC1 | 1.00E-36 | 95.05 | 92 | 63 |
| NC_021875.1 | Bifidobacterium kashiwanohense JCM 15439 | pBBKW-1 | 1.00E-35 | 95 | 98 | 40 |
| NC_021876.1 | Bifidobacterium kashiwanohense JCM 15439 | pBBKW-2 | 5.00E-36 | 95 | 98 | 42 |
| NC_010861.1 | Bifidobacterium longum | p6043B | 2.00E-36 | 95.05 | 99 | 24 |
| NC_010857.1 | Bifidobacterium longum | p6043A | 6.00E-38 | 98.95 | 91 | 1109 |
| NC_011139.1 | Bifidobacterium longum | pFI2576 | 5.00E-35 | 95.05 | 90 | 294 |
| NC_019200.1 | Bifidobacterium longum | pSP02 | 2.00E-43 | 100 | 101 | 8 |
| NC_006843.1 | Bifidobacterium longum | pTB6 | 2.00E-36 | 95.05 | 101 | 46 |
| NC_006997.1 | Bifidobacterium longum | pMG1 | 8.00E-35 | 95.05 | 90 | 181 |
| NC_004252.1 | Bifidobacterium longum DJO10A | pDOJH10L | 8.00E-34 | 95 | 91 | 322 |
| NC_004253.1 | Bifidobacterium longum DJO10A | pDOJH10S | 2.00E-36 | 95.05 | 99 | 95 |
| NC_002635.1 | Bifidobacterium longum KJ | pKJ36 | 2.00E-35 | 95.05 | 90 | 415 |
| NC_004978.1 | Bifidobacterium longum KJ | pKJ50 | 2.00E-37 | 96.94 | 90 | 857 |
| NC_004943.1 | Bifidobacterium longum NCC2705 | pBLO1 | 6.00E-33 | 95.05 | 91 | 14 |
| NC_004768.1 | Bifidobacterium longum RW041 | pNAC3 | 2.00E-34 | 95 | 90 | 1752 |
| NC_004769.1 | Bifidobacterium longum RW041 | PNAC2 | 2.00E-35 | 95.92 | 90 | 396 |
| NC_004770.1 | Bifidobacterium longum RW048 | pNAC1 | 1.00E-32 | 95.05 | 90 | 464 |
| NC_015053.1 | Bifidobacterium longum subsp. infantis 157F | p157F-NC1 | 5.00E-41 | 98.02 | 101 | 3 |
| NC_015066.1 | Bifidobacterium longum subsp. infantis 157F | p157F-NC2 | 3.00E-33 | 96.67 | 90 | 57 |
| NC_017220.1 | Bifidobacterium longum subsp. longum KACC 91563 | BLNIAS_P1 | 8.00E-36 | 96.88 | 90 | 31 |
| NC_017222.1 | Bifidobacterium longum subsp. longum KACC 91563 | BLNIAS_P2 | 9.00E-31 | 95.05 | 90 | 36 |
| NC_004443.1 | Bifidobacterium longum VMKB44 | pB44 | 8.00E-35 | 95.05 | 90 | 219 |
| NC_010164.1 | Bifidobacterium sp. A24 | pBIFA24 | 8.00E-44 | 100 | 101 | 1 |
| NC_008459.1 | Bordetella pertussis | pBP136 | 8.00E-41 | 99.01 | 101 | 11 |
| NC_009475.1 | Bradyrhizobium sp. BTAi1 | pBBta01 | 1.00E-34 | 95.05 | 100 | 4 |
| NC_022590.1 | Brevibacterium sp. Ap13 | pAP13 | 2.00E-39 | 98.02 | 101 | 6 |
| NC_019369.1 | Burkholderia cepacia | pYS1 | 1.00E-36 | 96.04 | 92 | 6 |
| NC_019378.1 | Burkholderia cepacia | pIJB1 | 3.00E-37 | 97 | 99 | 17 |
| NC_008385.1 | Burkholderia cepacia AMMD | 1 | 5.00E-36 | 95.05 | 91 | 201 |
| NC_022995.1 | Burkholderia sp. M701 | pM7012 | 1.00E-33 | 95 | 91 | 4 |
| NC_016626.1 | Burkholderia sp. YI23 | byi_1p | 2.00E-30 | 95.65 | 92 | 6 |
| NC_009227.1 | Burkholderia vietnamiensis G4 | pBVIE02 | 3.00E-36 | 96.04 | 94 | 23 |
| NC_009229.1 | Burkholderia vietnamiensis G4 | pBVIE03 | 2.00E-30 | 95.56 | 90 | 5 |
| NC_006134.1 | Campylobacter coli | pCC31 | 1.00E-32 | 95 | 90 | 1098 |
| NC_022355.1 | Campylobacter coli CVM N29710 | pN29710-1 | 1.00E-33 | 95.05 | 90 | 180 |
| NC_007141.1 | Campylobacter jejuni subsp. jejuni 81-176 | pTet | 2.00E-35 | 98.02 | 91 | 27 |
| NC_014801.1 | Campylobacter jejuni subsp. jejuni ICDCCJ07001 | pTet | 9.00E-34 | 95 | 90 | 639 |
| NC_017282.1 | Campylobacter jejuni subsp. jejuni S3 | pTet | 5.00E-36 | 98.9 | 101 | 1 |
| NC_013190.1 | Candidatus Accumulibacter phosphatis clade IIA str. UW-1 | pAph02 | 2.00E-35 | 95.05 | 100 | 12 |
| NC_013191.1 | Candidatus Accumulibacter phosphatis clade IIA str. UW-1 | pAph03 | 5.00E-36 | 95.96 | 91 | 10 |
| NC_015390.1 | Carnobacterium sp. 17-4 | pCAR50 | 3.00E-35 | 95.05 | 101 | 8 |
| NC_022601.1 | Carnobacterium sp. WN1359 | pWNCR12 | 2.00E-37 | 96.04 | 91 | 121 |
| NC_022602.1 | Carnobacterium sp. WN1359 | pWNCR47 | 3.00E-34 | 95 | 91 | 137 |
| NC_022603.1 | Carnobacterium sp. WN1359 | pWNCR64 | 4.00E-34 | 95.05 | 91 | 83 |
| NC_022607.1 | Carnobacterium sp. WN1359 | pWNCR15 | 8.00E-36 | 95.05 | 100 | 6 |
| NC_022608.1 | Carnobacterium sp. WN1359 | pWNCR9 | 5.00E-36 | 95.05 | 101 | 2 |
| NC_010333.1 | Caulobacter sp. K31 | pCAUL02 | 9.00E-35 | 95.05 | 101 | 4 |
| NC_008244.1 | Chelativorans sp. BNC1 | 3 | 2.00E-42 | 100 | 101 | 2 |
| NC_019360.1 | Citrobacter freundii | pNDM-CIT | 1.00E-34 | 95.05 | 93 | 40 |
| NC_020123.1 | Citrobacter freundii strain CFSTE | pT-OXA-181 | 2.00E-38 | 97.98 | 96 | 48 |
| NC_019983.1 | Citrobacter freundii strain Iona 2 | pCFI-1 | 1.00E-42 | 100 | 101 | 10 |
| NC_009793.1 | Citrobacter koseri ATCC BAA-895 | pCKO3 | 6.00E-35 | 97.8 | 91 | 16 |
| NC_013719.1 | Citrobacter rodentium ICC168 | pCROD3 | 2.00E-43 | 100 | 101 | 1 |
| NC_013717.1 | Citrobacter rodentium ICC168 | pCROD1 | 5.00E-38 | 97.03 | 99 | 9 |
| NC_003114.1 | Citrobacter rodentium strain DBS100 | pCRP3 | 1.00E-38 | 98.95 | 95 | 19 |
| NC_010937.1 | Clostridium perfringens | pCW3 | 2.00E-35 | 95.05 | 92 | 23 |
| NC_011412.1 | Clostridium perfringens | pCP8533etx | 1.00E-39 | 98.02 | 101 | 4 |
| NC_015712.1 | Clostridium perfringens | pCPPB-1 | 6.00E-38 | 97.03 | 101 | 2 |
| NC_019259.1 | Clostridium perfringens | pJIR3537 | 2.00E-42 | 100 | 101 | 2 |
| NC_019687.1 | Clostridium perfringens | pCpb2-CP1 | 1.00E-34 | 95 | 100 | 2 |
| NC_006872.1 | Clostridium perfringens F5603 | pBCNF5603 | 4.00E-38 | 99.01 | 93 | 12 |
| NC_003042.1 | Clostridium perfringens str. 13 | pCP13 | 1.00E-40 | 99.01 | 101 | 4 |
| NC_010332.1 | Collimonas fungivorans | pTer331 | 5.00E-37 | 96.04 | 101 | 4 |
| NC_021077.1 | Comamonas sp. 7D-2 | pBHB | 1.00E-31 | 95 | 90 | 57 |
| NC_016968.1 | Comamonas testosteroni | pTB30 | 3.00E-42 | 100 | 101 | 16 |
| NC_016978.1 | Comamonas testosteroni | pI2 | 4.00E-35 | 95.05 | 96 | 32 |
| NC_010935.1 | Comamonas testosteroni CNB-1 | pCNB | 5.00E-34 | 95.05 | 90 | 355 |
| NC_002143.1 | Comamonas testosteroni PtL5 cryptic | pPT1 | 2.00E-38 | 97.03 | 99 | 24 |
| NC_003239.1 | Corynebacterium diphtheriae | pNGA2 | 2.00E-36 | 95.05 | 100 | 14 |
| NC_005001.1 | Corynebacterium diphtheriae | pNG2 | 1.00E-32 | 96.67 | 90 | 10 |
| NC_003227.1 | Corynebacterium glutamicum | pTET3 | 1.00E-42 | 100 | 101 | 2 |
| NC_001791.1 | Corynebacterium glutamicum strain 1014 | pXZ10145.1 | 9.00E-36 | 95.96 | 99 | 7 |
| NC_001415.1 | Corynebacterium glutamicum strain 22243 | pAG1 | 4.00E-41 | 99.01 | 101 | 4 |
| NC_004945.1 | Corynebacterium glutamicum strain ATCC31830 | pCG4 | 1.00E-42 | 100 | 101 | 6 |
| NC_003490.1 | Corynebacterium jeikeium | pB85766 | 1.00E-38 | 97.03 | 94 | 14 |
| NC_004774.1 | Corynebacterium jeikeium | pA501 | 1.00E-35 | 95.05 | 101 | 12 |
| NC_021920.1 | Corynebacterium maris DSM 45190 | pCmaris1 | 8.00E-35 | 95 | 100 | 14 |
| NC_014167.1 | Corynebacterium resistens DSM 45100 | pJA144188 | 5.00E-35 | 95 | 90 | 200 |
| NC_009128.1 | Corynebacterium sp. L2-79-05 | pLEW279a | 6.00E-41 | 99.01 | 101 | 4 |
| NC_009129.1 | Corynebacterium sp. L2-79-05 | pLEW279b | 7.00E-40 | 98.02 | 100 | 10 |
| NC_004939.1 | Corynebacterium striatum strain M82B | pTP10 | 2.00E-42 | 100 | 101 | 6 |
| NC_009779.1 | Cronobacter sakazakii ATCC BAA-894 | pESA2 | 2.00E-35 | 95.05 | 90 | 225 |
| NC_023025.1 | Cronobacter sakazakii CMCC 45402 | p2 | 3.00E-37 | 96.04 | 97 | 155 |
| NC_020261.1 | Cronobacter sakazakii Sp291 | pSP291-2 | 1.00E-40 | 99.01 | 99 | 28 |
| NC_021293.1 | Cronobacter sakazakii strain ATCC 29544 | pCSA2 | 2.00E-43 | 100 | 101 | 2 |
| NC_013285.1 | Cronobacter turicensis z3032 | pCTU3 | 6.00E-37 | 96.04 | 101 | 5 |
| NC_007974.2 | Cupriavidus metallidurans CH34 |  | 3.00E-29 | 95 | 90 | 115 |
| NC_006525.1 | Cupriavidus metallidurans CH34 | pMOL28 | 8.00E-35 | 95.05 | 91 | 8 |
| NC_015727.1 | Cupriavidus necator N-1 | BB1p | 3.00E-32 | 95 | 95 | 18 |
| NC_012527.1 | Deinococcus deserti VCD115 | 1 | 4.00E-29 | 95 | 90 | 443 |
| NC_008010.2 | Deinococcus geothermalis DSM 11300 | pDGEO01 | 2.00E-30 | 95.05 | 90 | 24 |
| NC_009939.1 | Deinococcus geothermalis DSM 11300 | pDGEO02 | 9.00E-42 | 100 | 101 | 2 |
| NC_019789.1 | Deinococcus peraridilitoris DSM 19664 | pDEIPE01 | 6.00E-30 | 95 | 90 | 23 |
| NC_015163.1 | Deinococcus proteolyticus MRP | pDEIPR04 | 4.00E-42 | 100 | 101 | 2 |
| NC_000958.1 | Deinococcus radiodurans R1 | MP1 | 2.00E-36 | 100 | 91 | 6 |
| NC_019264.1 | Delftia acidovorans | pNB8c | 7.00E-37 | 99.01 | 91 | 3 |
| NC_005088.1 | Delftia acidovorans B | pUO1 | 1.00E-34 | 95 | 93 | 77 |
| NC_019312.1 | Delftia sp. KV29 | pKV29 | 8.00E-30 | 95 | 90 | 1325 |
| NC_012797.1 | Desulfovibrio magneticus RS-1 | pDMC1 | 6.00E-38 | 97.03 | 101 | 6 |
| NC_008741.1 | Desulfovibrio vulgaris DP4 | pDVUL01 | 1.00E-33 | 95.92 | 94 | 4 |
| NC_009955.1 | Dinoroseobacter shibae DFL 12 | pDSHI01 | 8.00E-36 | 96 | 100 | 6 |
| NC_009956.1 | Dinoroseobacter shibae DFL 12 | pDSHI02 | 2.00E-35 | 95.96 | 99 | 2 |
| NC_009957.1 | Dinoroseobacter shibae DFL 12 | pDSHI03 | 6.00E-35 | 95.92 | 91 | 60 |
| NC_009958.1 | Dinoroseobacter shibae DFL 12 | pDSHI04 | 1.00E-34 | 96.04 | 95 | 12 |
| NC_020280.1 | Edwardsiella ictaluri | pEI3 | 3.00E-41 | 99.01 | 97 | 67 |
| NC_014725.1 | Edwardsiella tarda strain CK41 | pCK41 | 7.00E-38 | 97.03 | 96 | 38 |
| NC_013090.1 | Endophytic bacterium LOB-07 | pLK39 | 5.00E-37 | 95.05 | 100 | 9 |
| NC_001735.4 | Enterobacter aerogenes | R751 | 2.00E-42 | 100 | 101 | 8 |
| NC_015963.1 | Enterobacter asburiae LF7a | pENTAS01 | 2.00E-36 | 97.03 | 96 | 6 |
| NC_015969.1 | Enterobacter asburiae LF7a | pENTAS02 | 6.00E-35 | 95 | 97 | 14 |
| NC_012006.1 | Enterobacter cloacae | pCHE-A | 1.00E-41 | 99.01 | 101 | 6 |
| NC_012555.1 | Enterobacter cloacae | pEC-IMP | 9.00E-31 | 95.6 | 91 | 28 |
| NC_015175.1 | Enterobacter cloacae | pS51A | 7.00E-32 | 96.67 | 90 | 9 |
| NC_017097.1 | Enterobacter cloacae | pUL3AT | 9.00E-39 | 97.03 | 101 | 16 |
| NC_019346.1 | Enterobacter cloacae | pNE1280 | 3.00E-35 | 95.05 | 101 | 15 |
| NC_019242.1 | Enterobacter cloacae | pS51B | 1.00E-41 | 99.01 | 101 | 4 |
| NC_019368.1 | Enterobacter cloacae | pEl1573 | 2.00E-38 | 97.03 | 101 | 1 |
| NC_016515.1 | Enterobacter cloacae EcWSU1 | pEcWSU1_A | 6.00E-38 | 97.96 | 98 | 2 |
| NC_019986.1 | Enterobacter cloacae strain BB1092 | pB1023 | 7.00E-42 | 99.01 | 101 | 23 |
| NC_021087.1 | Enterobacter cloacae strain M15 |  | 1.00E-35 | 95.05 | 101 | 11 |
| NC_014107.1 | Enterobacter cloacae subsp. cloacae ATCC 13047 | pECL_A | 1.00E-34 | 95.05 | 94 | 45 |
| NC_009425.1 | Enterobacter sp. 638 | pENTE01 | 8.00E-35 | 95.05 | 101 | 2 |
| NC_021492.1 | Enterobacter sp. R4-368 | pENT01 | 2.00E-35 | 95.96 | 99 | 6 |
| NC_015515.1 | Enterobacter sp. W001 | pR23 | 5.00E-43 | 100 | 101 | 2 |
| NC_019240.1 | Enterococcus durans | pGL | 2.00E-41 | 99.01 | 98 | 26 |
| NC_006827.2 | Enterococcus faecalis | pCF10 | 3.00E-35 | 95.05 | 96 | 257 |
| NC_002630.1 | Enterococcus faecalis | pAM373 | 4.00E-37 | 96.97 | 99 | 10 |
| NC_011642.1 | Enterococcus faecalis | pMG2200 | 1.00E-30 | 95.05 | 90 | 54 |
| NC_013514.1 | Enterococcus faecalis | pAMbeta1 | 1.00E-35 | 95.05 | 91 | 162 |
| NC_013533.1 | Enterococcus faecalis | pBEE99 | 4.00E-35 | 95.05 | 93 | 43 |
| NC_014726.1 | Enterococcus faecalis | pTW9 | 5.00E-34 | 95.05 | 91 | 107 |
| NC_014508.2 | Enterococcus faecalis | pEF-01 | 3.00E-32 | 95.05 | 91 | 109 |
| NC_019213.1 | Enterococcus faecalis | pWZ909 | 5.00E-37 | 96.97 | 91 | 114 |
| NC_019385.1 | Enterococcus faecalis | PML21 | 7.00E-38 | 96.04 | 100 | 4 |
| NC_017313.1 | Enterococcus faecalis 62 | EF62pB | 4.00E-38 | 97 | 100 | 4 |
| NC_017314.1 | Enterococcus faecalis 62 | EF62pA | 2.00E-43 | 100 | 101 | 24 |
| NC_017315.1 | Enterococcus faecalis 62 | EF62pC | 5.00E-38 | 97.03 | 100 | 11 |
| NC_018222.1 | Enterococcus faecalis D32 | EFD32pA | 3.00E-33 | 95.05 | 90 | 37 |
| NC_008445.1 | Enterococcus faecalis RE25 | pRE25 | 5.00E-38 | 97.03 | 101 | 54 |
| NC_004669.1 | Enterococcus faecalis V583 | pTEF1 | 6.00E-38 | 99.01 | 93 | 17 |
| NC_004670.1 | Enterococcus faecalis V583 | pTEF3 | 4.00E-40 | 98.02 | 101 | 12 |
| NC_004671.1 | Enterococcus faecalis V583 | pTEF2 | 1.00E-40 | 99.01 | 101 | 10 |
| NC_008259.1 | Enterococcus faecium | pCIZ2 | 3.00E-43 | 100 | 101 | 2 |
| NC_008768.1 | Enterococcus faecium | pVEF1 | 4.00E-32 | 95.05 | 91 | 49 |
| NC_010290.1 | Enterococcus faecium | pJS33 | 3.00E-39 | 97.03 | 99 | 4 |
| NC_010291.1 | Enterococcus faecium | pJS42 | 2.00E-43 | 100 | 101 | 2 |
| NC_010330.1 | Enterococcus faecium | pRI1 | 3.00E-43 | 100 | 101 | 6 |
| NC_010880.1 | Enterococcus faecium | pEF1 | 1.00E-39 | 98.97 | 95 | 3 |
| NC_010980.1 | Enterococcus faecium | pVEF3 | 5.00E-33 | 95 | 90 | 105 |
| NC_011140.1 | Enterococcus faecium | pIP816 | 6.00E-41 | 99.01 | 101 | 8 |
| NC_011364.1 | Enterococcus faecium | pMG1 | 3.00E-42 | 100 | 101 | 2 |
| NC_013317.1 | Enterococcus faecium | p5753cA | 9.00E-31 | 95.05 | 91 | 216 |
| NC_014959.1 | Enterococcus faecium | pS177 | 6.00E-36 | 98.02 | 92 | 186 |
| NC_015849.1 | Enterococcus faecium | pNJAKD | 7.00E-42 | 99.01 | 99 | 4 |
| NC_016009.1 | Enterococcus faecium | pM7M2 | 1.00E-35 | 95.05 | 90 | 66 |
| NC_016967.1 | Enterococcus faecium | pZB18 | 5.00E-39 | 98.02 | 92 | 4 |
| NC_019253.1 | Enterococcus faecium | pHY | 3.00E-43 | 100 | 101 | 2 |
| NC_021170.1 | Enterococcus faecium | pF856 | 2.00E-35 | 96 | 99 | 8 |
| NC_006427.1 | Enterococcus faecium | pJB01 | 2.00E-39 | 99.01 | 93 | 4 |
| NC_007594.1 | Enterococcus faecium | pHT beta | 1.00E-40 | 99.01 | 101 | 2 |
| NC_017032.1 | Enterococcus faecium Aus0004 | AUS0004_p1 | 7.00E-37 | 96.04 | 99 | 24 |
| NC_021987.1 | Enterococcus faecium Aus0085 | p1 | 1.00E-31 | 95 | 91 | 769 |
| NC_021988.1 | Enterococcus faecium Aus0085 | p3 | 2.00E-35 | 95.05 | 94 | 50 |
| NC_021995.1 | Enterococcus faecium Aus0085 | p2 | 8.00E-37 | 96.04 | 96 | 35 |
| NC_021996.1 | Enterococcus faecium Aus0085 | p5 | 2.00E-43 | 100 | 100 | 2 |
| NC_017963.1 | Enterococcus faecium DO | 3 | 2.00E-32 | 95.05 | 90 | 257 |
| NC_017962.1 | Enterococcus faecium DO | 2 | 3.00E-35 | 95.05 | 91 | 44 |
| NC_017961.1 | Enterococcus faecium DO | 1 | 6.00E-35 | 95 | 90 | 330 |
| NC_020208.1 | Enterococcus faecium NRRL B-2354 | pNB2354_1 | 2.00E-30 | 95.05 | 90 | 364 |
| NC_005000.1 | Enterococcus faecium U37 | pRUM | 6.00E-40 | 98.02 | 94 | 14 |
| NC_022879.1 | Enterococcus mundtii QU 25 | pQY182 | 9.00E-35 | 95.05 | 96 | 8 |
| NC_018977.1 | Erwinia amylovora ATCC BAA-2158 | pEAR4.3 | 3.00E-37 | 95.96 | 101 | 1 |
| NC_018985.1 | Erwinia amylovora ATCC BAA-2158 | pEAR5.2 | 5.00E-39 | 97.03 | 101 | 2 |
| NC_020920.1 | Erwinia amylovora CFBP 2585 | pEA3 | 6.00E-41 | 99.01 | 101 | 2 |
| NC_004446.1 | Erwinia amylovora IL-5 | pEA2.8 | 3.00E-37 | 95.05 | 92 | 22 |
| NC_005246.1 | Erwinia amylovora LebB66 | pEL60 | 2.00E-41 | 99.01 | 101 | 1 |
| NC_002487.1 | Escherichia coli | pCol-let | 2.00E-37 | 99.01 | 90 | 11 |
| NC_008460.1 | Escherichia coli | pO86A1 | 6.00E-35 | 95.05 | 93 | 24 |
| NC_008486.1 | Escherichia coli | pMG828-1 | 4.00E-40 | 97.03 | 101 | 1 |
| NC_008487.1 | Escherichia coli | pMG828-2 | 2.00E-43 | 100 | 101 | 2 |
| NC_008489.1 | Escherichia coli | pMG828-4 | 1.00E-35 | 95 | 90 | 37 |
| NC_008490.1 | Escherichia coli | pMG828-5 | 2.00E-35 | 95 | 90 | 28 |
| NC_008597.1 | Escherichia coli | pVI678 | 3.00E-43 | 100 | 101 | 1 |
| NC_009602.1 | Escherichia coli | pSFO157 | 2.00E-34 | 96.04 | 90 | 16 |
| NC_009781.1 | Escherichia coli | pIGJC156 | 1.00E-41 | 99.01 | 101 | 2 |
| NC_010064.1 | Escherichia coli | pLMO226 | 7.00E-39 | 99.01 | 90 | 135 |
| NC_010257.1 | Escherichia coli | MccC7-H22 | 1.00E-37 | 98.02 | 92 | 51 |
| NC_010378.1 | Escherichia coli | pOLA52 | 6.00E-37 | 98.02 | 94 | 71 |
| NC_010409.1 | Escherichia coli | pVM01 | 2.00E-35 | 96.04 | 90 | 61 |
| NC_010883.1 | Escherichia coli | pIGMS5 | 7.00E-39 | 97.03 | 101 | 2 |
| NC_010885.1 | Escherichia coli | pIGWZ12 | 8.00E-42 | 99.01 | 101 | 14 |
| NC_010898.1 | Escherichia coli | pIGRW12 | 1.00E-40 | 98.02 | 101 | 10 |
| NC_010904.1 | Escherichia coli | pColG | 9.00E-38 | 99.01 | 101 | 7 |
| NC_011418.1 | Escherichia coli | pEC278 | 6.00E-38 | 96.04 | 94 | 16 |
| NC_011514.1 | Escherichia coli | pTN38148 | 3.00E-40 | 98.02 | 100 | 12 |
| NC_011799.1 | Escherichia coli | p5217 | 2.00E-43 | 100 | 101 | 2 |
| NC_011812.1 | Escherichia coli | pO26-L | 7.00E-38 | 97.03 | 101 | 8 |
| NC_011964.1 | Escherichia coli | pAPEC-O103-ColBM | 2.00E-35 | 96.04 | 92 | 51 |
| NC_011977.1 | Escherichia coli | ColE9-J | 5.00E-35 | 96.81 | 94 | 2 |
| NC_012487.1 | Escherichia coli | pO26-Vir | 7.00E-42 | 100 | 101 | 2 |
| NC_012690.1 | Escherichia coli | peH4H | 2.00E-34 | 98.89 | 90 | 10 |
| NC_012886.1 | Escherichia coli | pRAx | 9.00E-35 | 97 | 90 | 233 |
| NC_004429.1 | Escherichia coli | pIS2 | 6.00E-39 | 98.96 | 96 | 36 |
| NC_013120.1 | Escherichia coli | pEK204 | 2.00E-40 | 99.01 | 100 | 31 |
| NC_013121.1 | Escherichia coli | pEK516 | 6.00E-38 | 97.03 | 101 | 62 |
| NC_013122.1 | Escherichia coli | pEK499 | 5.00E-42 | 100 | 101 | 16 |
| NC_013175.1 | Escherichia coli | pEC14_114 | 5.00E-42 | 100 | 101 | 3 |
| NC_013503.1 | Escherichia coli | pMAS2027 | 3.00E-34 | 97.8 | 90 | 30 |
| NC_013652.1 | Escherichia coli | pPM18 | 5.00E-42 | 99.01 | 101 | 12 |
| NC_013782.1 | Escherichia coli | pEC14-9 | 3.00E-33 | 96.74 | 92 | 3 |
| NC_014231.1 | Escherichia coli | pKC394 | 2.00E-42 | 100 | 101 | 35 |
| NC_014356.1 | Escherichia coli | IncQ-type pQ7 | 1.00E-37 | 97.92 | 94 | 8 |
| NC_014382.1 | Escherichia coli | pEC_B24 | 9.00E-37 | 96.04 | 96 | 30 |
| NC_014384.1 | Escherichia coli | pEC_L8 | 5.00E-42 | 100 | 101 | 1 |
| NC_014477.1 | Escherichia coli | pCT | 4.00E-42 | 100 | 101 | 1 |
| NC_014615.1 | Escherichia coli | pETN48 | 3.00E-34 | 95.96 | 99 | 9 |
| NC_014843.1 | Escherichia coli | p3521 | 8.00E-33 | 96.74 | 92 | 2 |
| NC_004998.1 | Escherichia coli | p1658/97 | 1.00E-36 | 96.04 | 90 | 93 |
| NC_015472.1 | Escherichia coli | pECTm80 | 5.00E-40 | 98.02 | 101 | 10 |
| NC_015872.1 | Escherichia coli | p271A | 2.00E-35 | 95.05 | 101 | 49 |
| NC_016039.1 | Escherichia coli | pHK17a | 7.00E-38 | 97.03 | 95 | 8 |
| NC_005248.1 | Escherichia coli | pIGAL1 | 3.00E-37 | 100 | 90 | 2 |
| NC_018994.1 | Escherichia coli | pNDM-1_Dok01 | 4.00E-39 | 98.02 | 101 | 2 |
| NC_018995.1 | Escherichia coli | pHUSEC41-1 | 9.00E-37 | 98.92 | 100 | 4 |
| NC_018997.1 | Escherichia coli | pHUSEC41-3 | 4.00E-43 | 100 | 101 | 3 |
| NC_019000.1 | Escherichia coli | pHUSEC41-2 | 3.00E-42 | 100 | 101 | 3 |
| NC_019038.1 | Escherichia coli | pChi7122-4 | 8.00E-42 | 99.01 | 100 | 2 |
| NC_019039.1 | Escherichia coli | pChi7122-3 | 2.00E-31 | 95.05 | 91 | 131 |
| NC_019037.1 | Escherichia coli | pChi7122-2 | 1.00E-36 | 96.04 | 101 | 26 |
| NC_019033.1 | Escherichia coli | pQNR2078 | 4.00E-40 | 98.02 | 101 | 1 |
| NC_019044.1 | Escherichia coli | pND12_96 | 9.00E-40 | 98.02 | 101 | 4 |
| NC_019046.1 | Escherichia coli | pNMEC31_31 | 1.00E-42 | 100 | 101 | 4 |
| NC_005324.1 | Escherichia coli | p9123 | 2.00E-38 | 99.01 | 92 | 36 |
| NC_005327.1 | Escherichia coli | pC15-1a | 9.00E-38 | 100 | 93 | 1 |
| NC_019061.1 | Escherichia coli | pPWD4_103 | 2.00E-39 | 98.02 | 101 | 19 |
| NC_019067.1 | Escherichia coli | pE001 | 9.00E-40 | 98.02 | 100 | 7 |
| NC_019073.1 | Escherichia coli | pHN7A8 | 2.00E-39 | 98.02 | 101 | 9 |
| NC_019083.1 | Escherichia coli | pEC14_35 | 8.00E-40 | 98.02 | 101 | 8 |
| NC_019088.1 | Escherichia coli | pRPEC180_47 | 2.00E-42 | 100 | 101 | 6 |
| NC_019097.1 | Escherichia coli | Plm | 2.00E-40 | 99.01 | 101 | 18 |
| NC_019013.1 | Escherichia coli | pSYM1 | 9.00E-41 | 99.01 | 101 | 11 |
| NC_019049.1 | Escherichia coli | pCM959 | 5.00E-32 | 95.05 | 90 | 156 |
| NC_019059.1 | Escherichia coli | pJD8 | 7.00E-44 | 100 | 101 | 2 |
| NC_019062.1 | Escherichia coli | p838C-R1 | 1.00E-35 | 98.9 | 91 | 131 |
| NC_019068.1 | Escherichia coli | pKST21 | 3.00E-42 | 99.01 | 101 | 2 |
| NC_019071.1 | Escherichia coli | pHK09 | 3.00E-42 | 100 | 101 | 2 |
| NC_019074.1 | Escherichia coli | pHNDD81-1 | 2.00E-36 | 98.02 | 92 | 15 |
| NC_019075.1 | Escherichia coli | pKST23 | 4.00E-39 | 97.12 | 95 | 14 |
| NC_019077.1 | Escherichia coli | pEC34A | 2.00E-43 | 100 | 101 | 2 |
| NC_019079.1 | Escherichia coli | pEC886 | 4.00E-43 | 100 | 101 | 2 |
| NC_019081.1 | Escherichia coli | pHCG11 | 4.00E-43 | 100 | 101 | 9 |
| NC_019085.1 | Escherichia coli | pPAB19-3 | 6.00E-42 | 99.01 | 96 | 2 |
| NC_019089.1 | Escherichia coli | pGUE-NDM | 5.00E-37 | 98.02 | 100 | 29 |
| NC_019090.1 | Escherichia coli | pHK23a | 3.00E-42 | 100 | 96 | 2 |
| NC_019093.1 | Escherichia coli | pLST424C-61 | 7.00E-36 | 95.05 | 100 | 5 |
| NC_019094.1 | Escherichia coli | p417H-90 | 8.00E-38 | 97.03 | 101 | 22 |
| NC_019098.1 | Escherichia coli | pHHA45 | 6.00E-43 | 100 | 101 | 6 |
| NC_019053.1 | Escherichia coli | pAm08WL3069 | 5.00E-43 | 100 | 99 | 6 |
| NC_019054.1 | Escherichia coli | pAm08CD7339 | 1.00E-41 | 99.01 | 101 | 17 |
| NC_019056.1 | Escherichia coli | pAm08CD9902 | 1.00E-38 | 97.03 | 101 | 20 |
| NC_019091.1 | Escherichia coli | pASL01a | 3.00E-38 | 97.03 | 95 | 88 |
| NC_019424.1 | Escherichia coli | pFOS-HK151325 | 3.00E-42 | 100 | 101 | 2 |
| NC_020086.1 | Escherichia coli | pE66An | 9.00E-37 | 96.04 | 97 | 36 |
| NC_005923.1 | Escherichia coli | pFL129 | 3.00E-43 | 100 | 101 | 23 |
| NC_021981.1 | Escherichia coli | pEC386IL | 2.00E-43 | 100 | 101 | 2 |
| NC_021983.1 | Escherichia coli | pEC386IL | 6.00E-44 | 100 | 101 | 35 |
| NC_021997.1 | Escherichia coli | pEC386IL | 9.00E-40 | 98.02 | 101 | 6 |
| NC_021999.1 | Escherichia coli | pEC386IL | 1.00E-43 | 100 | 101 | 8 |
| NC_022333.1 | Escherichia coli | pCss165Kan | 8.00E-35 | 98.9 | 91 | 6 |
| NC_022374.1 | Escherichia coli | pHKU1 | 5.00E-38 | 97.03 | 96 | 8 |
| NC_022992.1 | Escherichia coli | pO111-CRL-115 | 3.00E-35 | 95.92 | 90 | 231 |
| NC_022996.1 | Escherichia coli | pO26-CRL-125 | 3.00E-39 | 100 | 96 | 13 |
| NC_006881.1 | Escherichia coli | pColK-K235 | 1.00E-37 | 96.04 | 91 | 30 |
| NC_007635.1 | Escherichia coli | pCoo | 2.00E-38 | 97.03 | 101 | 7 |
| NC_001740.1 | Escherichia coli | RSF1010 | 5.00E-42 | 100 | 99 | 58 |
| NC_017627.1 | Escherichia coli 042 | pAA | 1.00E-37 | 97.03 | 101 | 9 |
| NC_010558.1 | Escherichia coli 1520 | pIP1206 | 2.00E-36 | 96.04 | 101 | 11 |
| NC_010719.1 | Escherichia coli 53638 | p53638_226 | 6.00E-37 | 96.97 | 97 | 14 |
| NC_010720.1 | Escherichia coli 53638 | p53638_75 | 2.00E-39 | 98.02 | 100 | 91 |
| NC_011752.1 | Escherichia coli 55989 | 55989p | 4.00E-35 | 95.05 | 101 | 50 |
| NC_006671.1 | Escherichia coli A2363 | pAPEC-O2-R | 1.00E-36 | 99.01 | 91 | 37 |
| NC_007675.1 | Escherichia coli A2363 | pAPEC-O2-ColV | 2.00E-36 | 97.03 | 91 | 432 |
| NC_017629.1 | Escherichia coli ABU 83972 | pABU | 2.00E-38 | 99.02 | 91 | 7 |
| NC_023323.1 | Escherichia coli ACN001 | pACN001-A | 3.00E-35 | 95.05 | 93 | 45 |
| NC_023325.1 | Escherichia coli ACN001 | pACN001-D | 6.00E-40 | 98 | 100 | 11 |
| NC_023326.1 | Escherichia coli ACN001 | pACN001-F | 2.00E-39 | 99.01 | 96 | 20 |
| NC_023327.1 | Escherichia coli ACN001 | pACN001-B | 4.00E-32 | 95 | 90 | 66 |
| NC_023328.1 | Escherichia coli ACN001 | pACN001-E | 1.00E-41 | 99.01 | 100 | 14 |
| NC_009838.1 | Escherichia coli APEC O1 | pAPEC-O1-R | 2.00E-37 | 97.03 | 97 | 94 |
| NC_009837.1 | Escherichia coli APEC O1 | pAPEC-O1-ColBM | 5.00E-38 | 97.98 | 97 | 22 |
| NC_002142.1 | Escherichia coli B171 | pB171 | 3.00E-36 | 96 | 93 | 28 |
| NC_011980.1 | Escherichia coli chi7122 | pAPEC-1 | 1.00E-29 | 95 | 90 | 390 |
| NC_009786.1 | Escherichia coli E24377A | pETEC_80 | 9.00E-37 | 96.04 | 101 | 25 |
| NC_009787.1 | Escherichia coli E24377A | pETEC_35 | 2.00E-34 | 95.05 | 96 | 20 |
| NC_009788.1 | Escherichia coli E24377A | pETEC_73 | 1.00E-33 | 97.78 | 90 | 126 |
| NC_009789.1 | Escherichia coli E24377A | pETEC_6 | 2.00E-42 | 99.01 | 97 | 3 |
| NC_009790.1 | Escherichia coli E24377A | pETEC_74 | 4.00E-35 | 95.05 | 100 | 27 |
| NC_009791.1 | Escherichia coli E24377A | pETEC_5 | 1.00E-41 | 99.01 | 99 | 2 |
| NC_011754.1 | Escherichia coli ED1a | pECOED | 2.00E-34 | 95 | 90 | 23 |
| NC_007365.1 | Escherichia coli EH41 | pO113 | 2.00E-35 | 96.94 | 92 | 58 |
| NC_014235.1 | Escherichia coli ETEC 1392/75 | p75 | 3.00E-37 | 100 | 90 | 11 |
| NC_014233.1 | Escherichia coli ETEC 1392/75 | p557 | 7.00E-37 | 97.98 | 91 | 96 |
| NC_014232.1 | Escherichia coli ETEC 1392/75 | p1081 | 4.00E-36 | 96 | 100 | 4 |
| NC_014234.1 | Escherichia coli ETEC 1392/75 | p746 | 1.00E-40 | 99.01 | 99 | 63 |
| NC_013507.1 | Escherichia coli ETEC H10407 | pEntH10407 | 8.00E-37 | 96.04 | 96 | 18 |
| NC_017722.1 | Escherichia coli ETEC H10407 | p666 | 3.00E-37 | 96.04 | 101 | 3 |
| NC_017724.1 | Escherichia coli ETEC H10407 | p948 | 1.00E-36 | 97.92 | 96 | 18 |
| NC_017723.1 | Escherichia coli ETEC H10407 | p58 | 1.00E-41 | 99.01 | 101 | 2 |
| NC_018998.1 | Escherichia coli F18+ | pTC1 | 2.00E-34 | 95 | 99 | 9 |
| NC_022742.1 | Escherichia coli HUSEC2011 | pHUSEC2011-1 | 4.00E-35 | 95.05 | 100 | 238 |
| NC_022743.1 | Escherichia coli HUSEC2011 | pHUSEC2011-2 | 4.00E-37 | 96.04 | 97 | 66 |
| NC_022741.1 | Escherichia coli HUSEC2011 | pHUSEC2011-3 | 4.00E-41 | 98.02 | 101 | 6 |
| NC_022649.1 | Escherichia coli JJ1886 | pJJ1886_2 | 1.00E-34 | 97.03 | 90 | 34 |
| NC_022650.1 | Escherichia coli JJ1886 | pJJ1886_4 | 7.00E-37 | 96.04 | 93 | 14 |
| NC_022651.1 | Escherichia coli JJ1886 | pJJ1886_5 | 5.00E-35 | 95.05 | 91 | 102 |
| NC_022661.1 | Escherichia coli JJ1886 | pJJ1886_1 | 7.00E-44 | 100 | 101 | 14 |
| NC_022662.1 | Escherichia coli JJ1886 | pJJ1886_3 | 4.00E-41 | 99.01 | 101 | 27 |
| NC_002525.1 | Escherichia coli K-12 | R721 | 9.00E-36 | 95.05 | 101 | 92 |
| NC_002483.1 | Escherichia coli K-12 | F DNA | 9.00E-32 | 95.65 | 91 | 179 |
| NC_002145.1 | Escherichia coli KL4 | pKL1 | 1.00E-40 | 100 | 95 | 2 |
| NC_016904.1 | Escherichia coli KO11FL | pEKO1101 | 3.00E-39 | 98 | 96 | 15 |
| NC_011917.1 | Escherichia coli LF82 | plLF82 | 5.00E-35 | 95.05 | 90 | 145 |
| NC_013354.1 | Escherichia coli O103:H2 str. 12009 | pO103 | 7.00E-40 | 98.02 | 98 | 45 |
| NC_018651.1 | Escherichia coli O104:H4 str. 2009EL-2050 | p09EL50 | 1.00E-36 | 96.04 | 99 | 41 |
| NC_018659.1 | Escherichia coli O104:H4 str. 2011C-3493 | pESBL-EA11 | 1.00E-42 | 100 | 101 | 1 |
| NC_018666.1 | Escherichia coli O104:H4 str. 2011C-3493 | pAA-EA11 | 2.00E-37 | 98.94 | 94 | 1 |
| NC_013366.1 | Escherichia coli O111:H- str. 11128 | pO111_3 | 4.00E-35 | 95.05 | 101 | 58 |
| NC_013367.1 | Escherichia coli O111:H- str. 11128 | pO111_4 | 2.00E-40 | 98.02 | 101 | 4 |
| NC_013368.1 | Escherichia coli O111:H- str. 11128 | pO111_5 | 3.00E-43 | 100 | 101 | 2 |
| NC_013365.1 | Escherichia coli O111:H- str. 11128 | pO111_1 | 4.00E-40 | 99.01 | 101 | 19 |
| NC_013370.1 | Escherichia coli O111:H- str. 11128 | pO111_2 | 2.00E-34 | 95 | 94 | 247 |
| NC_011603.1 | Escherichia coli O127:H6 str. E2348/69 | pMAR2 | 1.00E-36 | 96.04 | 101 | 34 |
| NC_007414.1 | Escherichia coli O157:H7 EDL933 | pO157 | 1.00E-35 | 95.05 | 91 | 46 |
| NC_011350.1 | Escherichia coli O157:H7 str. EC4115 | pO157 | 4.00E-42 | 100 | 101 | 4 |
| NC_011351.1 | Escherichia coli O157:H7 str. EC4115 | pEC4115 | 7.00E-41 | 99.01 | 101 | 2 |
| NC_019087.1 | Escherichia coli O25b:H4-ST131 str. EC958 | pKC396 | 7.00E-43 | 100 | 101 | 5 |
| NC_020271.1 | Escherichia coli O25b:H4-ST131 str. EC958 strain ST131 | pJIE186-2 | 2.00E-34 | 96.04 | 90 | 28 |
| NC_013728.1 | Escherichia coli O26:H- | pO26-CRL | 2.00E-40 | 99.01 | 101 | 2 |
| NC_013363.1 | Escherichia coli O26:H11 str. 11368 | pO26_3 | 3.00E-36 | 95.05 | 101 | 2 |
| NC_013362.1 | Escherichia coli O26:H11 str. 11368 | pO26_2 | 1.00E-34 | 95.05 | 101 | 44 |
| NC_013369.1 | Escherichia coli O26:H11 str. 11368 | pO26_1 | 4.00E-35 | 95.05 | 98 | 36 |
| NC_013942.1 | Escherichia coli O55:H7 str. CB9615 | pO55 | 3.00E-35 | 95.05 | 100 | 32 |
| NC_017653.1 | Escherichia coli O55:H7 str. RM12579 | p12579_1 | 2.00E-34 | 95.05 | 90 | 129 |
| NC_017654.1 | Escherichia coli O55:H7 str. RM12579 | p12579_3 | 5.00E-43 | 100 | 101 | 4 |
| NC_017648.1 | Escherichia coli O7:K1 str. CE10 | pCE10B | 1.00E-41 | 99.01 | 101 | 2 |
| NC_017647.1 | Escherichia coli O7:K1 str. CE10 | pCE10A | 1.00E-39 | 98.02 | 100 | 69 |
| NC_017659.1 | Escherichia coli O83:H1 str. NRG 857C | pO83_CORR | 2.00E-42 | 100 | 101 | 1 |
| NC_022371.1 | Escherichia coli PMV-1 pHUSEC411like | pHUSEC411like | 1.00E-35 | 95.05 | 92 | 68 |
| NC_011747.1 | Escherichia coli S88 | pECOS88 | 1.00E-30 | 95 | 90 | 67 |
| NC_011413.1 | Escherichia coli SE11 | pSE11-2 | 2.00E-34 | 95 | 100 | 7 |
| NC_011416.1 | Escherichia coli SE11 | pSE11-3 | 7.00E-37 | 96.04 | 96 | 18 |
| NC_011419.1 | Escherichia coli SE11 | pSE11-1 | 4.00E-42 | 100 | 101 | 11 |
| NC_013655.1 | Escherichia coli SE15 | pECSF1 | 2.00E-40 | 99.01 | 101 | 12 |
| NC_010486.1 | Escherichia coli SMS-3-5 | pSMS35_4 | 2.00E-43 | 100 | 101 | 2 |
| NC_010487.1 | Escherichia coli SMS-3-5 | pSMS35_3 | 2.00E-36 | 95.05 | 96 | 7 |
| NC_010488.1 | Escherichia coli SMS-3-5 | pSMS35_130 | 2.00E-36 | 96 | 98 | 12 |
| NC_020278.2 | Escherichia coli strain 3A11 | pHN3A11 | 1.00E-40 | 99.01 | 100 | 10 |
| NC_023277.1 | Escherichia coli strain 63743 | pEQ2 | 1.00E-41 | 100 | 101 | 10 |
| NC_023329.1 | Escherichia coli strain B3804 | pIFM3804 | 2.00E-34 | 96.84 | 95 | 47 |
| NC_019990.1 | Escherichia coli strain BB1093 | pB1024 | 1.00E-40 | 99.01 | 96 | 42 |
| NC_023315.1 | Escherichia coli strain EQ011 | pEQ011 | 1.00E-36 | 96.04 | 99 | 117 |
| NC_022885.1 | Escherichia coli strain LK-NARMP | pKPC-LKEc | 6.00E-42 | 100 | 101 | 40 |
| NC_023289.1 | Escherichia coli strain T23 | pEQ1 | 4.00E-42 | 100 | 101 | 8 |
| NC_017630.1 | Escherichia coli UM146 | pUM146 | 5.00E-37 | 96.04 | 93 | 221 |
| NC_011739.1 | Escherichia coli UMN026 | p2ESCUM | 1.00E-42 | 100 | 101 | 4 |
| NC_011749.1 | Escherichia coli UMN026 | p1ESCUM | 5.00E-42 | 100 | 101 | 2 |
| NC_017642.1 | Escherichia coli UMNK88 | pUMNK88_91 | 2.00E-39 | 98.02 | 95 | 53 |
| NC_017639.1 | Escherichia coli UMNK88 | pUMNK88_K88 | 3.00E-31 | 95.6 | 91 | 52 |
| NC_017640.1 | Escherichia coli UMNK88 | pUMNK88_Ent | 2.00E-39 | 98.02 | 101 | 21 |
| NC_017643.1 | Escherichia coli UMNK88 | pUMNK88_Hly | 3.00E-36 | 96.04 | 96 | 50 |
| NC_007941.1 | Escherichia coli UTI89 | pUTI89 | 1.00E-36 | 96.04 | 93 | 27 |
| NC_012944.1 | Escherichia coli Vir68 | pVir68 | 5.00E-36 | 96 | 90 | 28 |
| NC_011743.1 | Escherichia fergusonii ATCC 35469 | pEFER | 3.00E-35 | 95.05 | 96 | 32 |
| NC_009716.1 | Escherichia sp. Sflu5 cryptic | pAK51 | 2.00E-33 | 95.05 | 90 | 59 |
| NC_012782.1 | Eubacterium eligens ATCC 27750 | unnamed | 2.00E-32 | 95 | 90 | 1325 |
| NC_012780.1 | Eubacterium eligens ATCC 27750 | unnamed | 8.00E-29 | 95 | 90 | 35420 |
| NC_010607.1 | Exiguobacterium arabatum | pEspA | 2.00E-36 | 95.05 | 101 | 4 |
| NC_010608.1 | Exiguobacterium arabatum | pEspB | 9.00E-40 | 98.02 | 101 | 4 |
| NC_023058.1 | Exiguobacterium sp. S3-2 | pMC3 | 2.00E-36 | 95.05 | 98 | 4 |
| NC_023287.1 | Exiguobacterium sp. S3-2 | pMC1 | 5.00E-33 | 95 | 91 | 130 |
| NC_023288.1 | Exiguobacterium sp. S3-2 | pMC2 | 2.00E-37 | 96.04 | 101 | 4 |
| NC_010371.1 | Finegoldia magna ATCC 29328 | pFMC | 2.00E-30 | 95 | 90 | 42 |
| NC_010848.1 | Flavobacterium sp. KI723T1 | pOAD2 | 8.00E-35 | 95 | 91 | 10 |
| NC_009966.1 | Fluoribacter dumoffii Tex-KL | pLD-TEX-KL | 3.00E-42 | 100 | 101 | 2 |
| NC_002109.1 | Francisella tularensis | pOM1 | 2.00E-43 | 100 | 101 | 2 |
| NC_016021.1 | Gluconacetobacter xylinus NBRC 3288 | pGXY020 | 2.00E-33 | 95.79 | 95 | 4 |
| NC_016028.1 | Gluconacetobacter xylinus NBRC 3288 | pGXY030 | 1.00E-42 | 100 | 101 | 4 |
| NC_019397.1 | Gluconobacter oxydans H24 | unnamed | 1.00E-33 | 96.81 | 94 | 4 |
| NC_013442.1 | Gordonia bronchialis DSM 43247 | pGBRO01 | 9.00E-37 | 96.04 | 101 | 2 |
| NC_016907.1 | Gordonia polyisoprenivorans VH2 | p174 | 2.00E-31 | 95.05 | 92 | 12 |
| NC_018580.1 | Gordonia sp. KTR9 | pGKT2 | 9.00E-35 | 95.05 | 101 | 8 |
| NC_018582.1 | Gordonia sp. KTR9 | pGKT1 | 2.00E-39 | 98.02 | 101 | 2 |
| NC_018583.1 | Gordonia sp. KTR9 | pGKT3 | 4.00E-39 | 98.02 | 101 | 6 |
| NC_005307.1 | Gordonia westfalica strain DSM44215T | pKB1 | 3.00E-37 | 97 | 100 | 4 |
| NC_011409.1 | Haemophilus influenzae | ICEhin1056 | 6.00E-38 | 97.03 | 93 | 10 |
| NC_019181.1 | Haemophilus influenzae | pPN223 | 4.00E-39 | 100 | 93 | 1 |
| NC_019182.1 | Haemophilus influenzae | pLFH64 | 2.00E-43 | 100 | 101 | 7 |
| NC_019183.1 | Haemophilus influenzae | pLFS5 | 7.00E-39 | 100 | 101 | 9 |
| NC_012661.1 | Haemophilus parasuis | pHN61 | 1.00E-34 | 97.78 | 90 | 80 |
| NC_021186.1 | Haemophilus parasuis strain QY431 | pQY431 | 4.00E-43 | 100 | 101 | 32 |
| NC_008153.1 | Human gut | pTRACA17 | 7.00E-41 | 98.02 | 101 | 2 |
| NC_008154.1 | Human gut | pTRACA10 | 2.00E-32 | 95 | 91 | 56 |
| NC_014633.1 | Ilyobacter polytropus DSM 2926 | pILYOP01 | 1.00E-29 | 95 | 90 | 104 |
| NC_014621.1 | Ketogulonicigenium vulgare Y25 | pYP1 | 8.00E-31 | 95 | 90 | 242 |
| NC_014626.1 | Ketogulonicigenium vulgare Y25 | pYP12 | 3.00E-36 | 96.97 | 99 | 2 |
| NC_017386.1 | Ketogulonigenium vulgarum WSH-001 | 1 | 4.00E-34 | 95 | 92 | 13 |
| NC_019286.1 | Klebsiella oxytoca | pINCan01 | 3.00E-36 | 95.05 | 100 | 21 |
| NC_018107.1 | Klebsiella oxytoca E718 | pKOX_R1 | 8.00E-39 | 98.02 | 101 | 18 |
| NC_021501.1 | Klebsiella oxytoca E718 | pKOX_NDM1 | 7.00E-34 | 95 | 91 | 34 |
| NC_002610.1 | Klebsiella pneumoniae | pKlebB-k17/80 | 3.00E-36 | 95.05 | 93 | 16 |
| NC_010261.1 | Klebsiella pneumoniae | pH205 | 5.00E-32 | 95.56 | 101 | 5 |
| NC_010726.1 | Klebsiella pneumoniae | pMET-1 | 6.00E-36 | 95.96 | 99 | 28 |
| NC_010870.1 | Klebsiella pneumoniae | pK29 | 3.00E-37 | 97.03 | 97 | 27 |
| NC_010886.1 | Klebsiella pneumoniae | pK245 | 9.00E-38 | 98.02 | 93 | 64 |
| NC_003486.1 | Klebsiella pneumoniae | pJHCMW1 | 3.00E-40 | 98.02 | 97 | 10 |
| NC_011383.1 | Klebsiella pneumoniae | 9 | 3.00E-42 | 100 | 101 | 2 |
| NC_011385.1 | Klebsiella pneumoniae | 12 | 1.00E-34 | 95 | 92 | 6 |
| NC_011406.1 | Klebsiella pneumoniae | pIGMS31 | 5.00E-36 | 95 | 100 | 5 |
| NC_011511.1 | Klebsiella pneumoniae | p169 | 2.00E-36 | 95.05 | 92 | 184 |
| NC_011512.1 | Klebsiella pneumoniae | p9701 | 1.00E-40 | 98.99 | 97 | 86 |
| NC_011641.1 | Klebsiella pneumoniae | pCTXM360 | 8.00E-37 | 96.04 | 101 | 5 |
| NC_011640.1 | Klebsiella pneumoniae | pKpn114 | 3.00E-35 | 95.05 | 101 | 29 |
| NC_013542.1 | Klebsiella pneumoniae | pKF3-70 | 6.00E-32 | 96.04 | 91 | 113 |
| NC_013950.1 | Klebsiella pneumoniae | pKF3-94 | 2.00E-33 | 95.05 | 90 | 257 |
| NC_013951.1 | Klebsiella pneumoniae | pKF3-140 | 2.00E-36 | 96.04 | 97 | 67 |
| NC_014016.1 | Klebsiella pneumoniae | pKpQIL | 1.00E-36 | 98.02 | 91 | 47 |
| NC_014312.1 | Klebsiella pneumoniae | pKP048 | 2.00E-36 | 96.04 | 99 | 75 |
| NC_014368.1 | Klebsiella pneumoniae | pNL194 | 3.00E-42 | 100 | 101 | 16 |
| NC_014478.1 | Klebsiella pneumoniae | unnamed | 5.00E-35 | 95.05 | 92 | 114 |
| NC_015154.1 | Klebsiella pneumoniae | pc15-k | 4.00E-36 | 96.04 | 90 | 198 |
| NC_016966.1 | Klebsiella pneumoniae | pUUH239.2 | 2.00E-36 | 96.04 | 101 | 1 |
| NC_016979.1 | Klebsiella pneumoniae | pUUH239.1 | 3.00E-36 | 95.05 | 94 | 58 |
| NC_016976.1 | Klebsiella pneumoniae | pR55 | 1.00E-33 | 96.81 | 91 | 8 |
| NC_016980.1 | Klebsiella pneumoniae | pNDM-MAR | 2.00E-37 | 97.03 | 101 | 28 |
| NC_019158.1 | Klebsiella pneumoniae | pNDM10469 | 2.00E-34 | 95.1 | 101 | 2 |
| NC_019152.1 | Klebsiella pneumoniae | pSLMT | 1.00E-35 | 95.05 | 101 | 12 |
| NC_019153.1 | Klebsiella pneumoniae | pNDM-KN | 2.00E-36 | 96.04 | 101 | 2 |
| NC_019154.1 | Klebsiella pneumoniae | pOXA-48 | 2.00E-41 | 99.01 | 100 | 1 |
| NC_019155.1 | Klebsiella pneumoniae | pKpQIL-IT | 5.00E-36 | 97.03 | 90 | 285 |
| NC_019156.1 | Klebsiella pneumoniae | pAAC154 | 2.00E-37 | 98.94 | 90 | 2 |
| NC_019157.1 | Klebsiella pneumoniae | pIncX-SHV | 8.00E-41 | 99.01 | 101 | 9 |
| NC_019160.1 | Klebsiella pneumoniae | pKP3-A | 1.00E-42 | 100 | 100 | 12 |
| NC_019165.1 | Klebsiella pneumoniae | pKPN101-IT | 1.00E-36 | 98.99 | 91 | 20 |
| NC_019166.1 | Klebsiella pneumoniae | pIMP-HZ1 | 8.00E-43 | 100 | 101 | 1 |
| NC_019389.1 | Klebsiella pneumoniae | pKDO1 | 6.00E-35 | 95.05 | 101 | 88 |
| NC_019390.1 | Klebsiella pneumoniae | pKPN_CZ | 2.00E-31 | 95 | 91 | 603 |
| NC_020893.1 | Klebsiella pneumoniae | pKPC-LK30 | 6.00E-33 | 96.04 | 90 | 147 |
| NC_020087.1 | Klebsiella pneumoniae | pK1HV | 8.00E-40 | 99.01 | 97 | 10 |
| NC_020088.1 | Klebsiella pneumoniae | pK18An | 2.00E-37 | 100 | 92 | 34 |
| NC_021180.1 | Klebsiella pneumoniae | pNDM-1saitama01 | 8.00E-32 | 95 | 91 | 57 |
| NC_021356.1 | Klebsiella pneumoniae | pKP53IL | 4.00E-37 | 96.04 | 94 | 54 |
| NC_021357.1 | Klebsiella pneumoniae | pKP53IL | 2.00E-41 | 99.01 | 101 | 30 |
| NC_021363.1 | Klebsiella pneumoniae | pKP53IL | 1.00E-41 | 99.01 | 101 | 49 |
| NC_021364.1 | Klebsiella pneumoniae | pKP53IL | 1.00E-40 | 98.02 | 101 | 18 |
| NC_021365.1 | Klebsiella pneumoniae | pKP53IL | 7.00E-44 | 100 | 101 | 4 |
| NC_021488.1 | Klebsiella pneumoniae | pKPoxa-48N1 | 2.00E-38 | 100 | 94 | 2 |
| NC_021502.1 | Klebsiella pneumoniae | pKPoxa-48N2 | 1.00E-30 | 95.56 | 90 | 95 |
| NC_021576.1 | Klebsiella pneumoniae | pKP1780 | 9.00E-41 | 99.01 | 101 | 14 |
| NC_021654.1 | Klebsiella pneumoniae | pKN-LS6 | 1.00E-34 | 95.05 | 101 | 10 |
| NC_021655.1 | Klebsiella pneumoniae | pKpQIL-LS6 | 9.00E-39 | 99.01 | 94 | 96 |
| NC_021656.1 | Klebsiella pneumoniae | pKpQIL-SC29 | 5.00E-38 | 97.03 | 93 | 116 |
| NC_021622.1 | Klebsiella pneumoniae | pK45-67VIM | 1.00E-40 | 99.01 | 101 | 7 |
| NC_022520.1 | Klebsiella pneumoniae | pBK15692 | 1.00E-40 | 99.01 | 101 | 39 |
| NC_023314.1 | Klebsiella pneumoniae | pKPS30 | 2.00E-37 | 97 | 97 | 19 |
| NC_005018.1 | Klebsiella pneumoniae 2kI | pKPN2 | 8.00E-42 | 99.01 | 101 | 6 |
| NC_011281.1 | Klebsiella pneumoniae 342 | pKP91 | 4.00E-35 | 96 | 90 | 117 |
| NC_011282.1 | Klebsiella pneumoniae 342 | pKP187 | 3.00E-34 | 95.05 | 90 | 88 |
| NC_005015.1 | Klebsiella pneumoniae BM4493 | pIP843 | 2.00E-39 | 99.01 | 94 | 28 |
| NC_005249.1 | Klebsiella pneumoniae CG43 | pLVPK | 1.00E-34 | 95.05 | 90 | 131 |
| NC_021660.2 | Klebsiella pneumoniae FCF3SP | pKPC_FCF/3SP | 2.00E-42 | 100 | 101 | 4 |
| NC_022078.1 | Klebsiella pneumoniae JM45 | p1 | 1.00E-41 | 100 | 101 | 4 |
| NC_022083.1 | Klebsiella pneumoniae JM45 | p2 | 2.00E-41 | 99.01 | 101 | 44 |
| NC_017541.1 | Klebsiella pneumoniae KCTC 2242 | pKCTC2242 | 6.00E-31 | 95.05 | 91 | 58 |
| NC_006625.1 | Klebsiella pneumoniae NTUH-K2044 | pK2044 | 9.00E-42 | 100 | 101 | 2 |
| NC_019988.1 | Klebsiella pneumoniae strain BB1088 | pB1019 | 4.00E-34 | 95.74 | 94 | 8 |
| NC_019987.1 | Klebsiella pneumoniae strain BB1089 | pB1020 | 1.00E-43 | 100 | 101 | 4 |
| NC_019989.1 | Klebsiella pneumoniae strain BB1090 | pB1021 | 2.00E-35 | 95.83 | 96 | 6 |
| NC_019888.1 | Klebsiella pneumoniae strain BK31551 | pBK31551 | 4.00E-35 | 95.05 | 95 | 12 |
| NC_019899.1 | Klebsiella pneumoniae strain BK31567 | pBK31567 | 2.00E-36 | 97.03 | 93 | 4 |
| NC_020132.1 | Klebsiella pneumoniae strain BK32179 | pBK32179 | 2.00E-36 | 96.04 | 92 | 43 |
| NC_021078.1 | Klebsiella pneumoniae strain Kp002 | pJEG011 | 5.00E-33 | 95 | 90 | 131 |
| NC_021238.1 | Klebsiella pneumoniae strain Kpn-1433 | pKP1433 | 1.00E-39 | 98.02 | 96 | 32 |
| NC_023330.1 | Klebsiella pneumoniae strain KPS77 | pKPS77 | 5.00E-31 | 95 | 90 | 159 |
| NC_022740.1 | Klebsiella pneumoniae strain MGR-K194 | pNDM_MGR194 | 5.00E-37 | 99.01 | 91 | 59 |
| NC_022609.1 | Klebsiella pneumoniae strain N11-0042 | pKp11-42 | 7.00E-35 | 95.05 | 94 | 181 |
| NC_023334.1 | Klebsiella pneumoniae strain ST15 | pKP02022 | 2.00E-35 | 95.05 | 90 | 34 |
| NC_023333.1 | Klebsiella pneumoniae strain ST23 | pKP007 | 1.00E-33 | 95.05 | 92 | 145 |
| NC_023332.1 | Klebsiella pneumoniae strain ST48 | pKP09085 | 1.00E-33 | 95 | 90 | 165 |
| NC_016838.1 | Klebsiella pneumoniae subsp. pneumoniae HS11286 | pKPHS1 | 2.00E-32 | 95.05 | 90 | 174 |
| NC_016839.1 | Klebsiella pneumoniae subsp. pneumoniae HS11286 | pKPHS3 | 3.00E-32 | 95.05 | 91 | 15 |
| NC_016846.1 | Klebsiella pneumoniae subsp. pneumoniae HS11286 | pKPHS2 | 4.00E-36 | 97.03 | 101 | 34 |
| NC_016840.1 | Klebsiella pneumoniae subsp. pneumoniae HS11286 | pKPHS4 | 4.00E-39 | 97.03 | 101 | 17 |
| NC_016847.1 | Klebsiella pneumoniae subsp. pneumoniae HS11286 | pKPHS5 | 2.00E-36 | 95.05 | 101 | 13 |
| NC_021198.1 | Klebsiella pneumoniae subsp. pneumoniae KPX | pKPX-1 | 5.00E-33 | 96.77 | 93 | 6 |
| NC_021199.1 | Klebsiella pneumoniae subsp. pneumoniae KPX | pKPX-2 | 6.00E-36 | 96.04 | 90 | 91 |
| NC_009649.1 | Klebsiella pneumoniae subsp. pneumoniae MGH 78578 | pKPN3 | 4.00E-33 | 95.05 | 90 | 615 |
| NC_009650.1 | Klebsiella pneumoniae subsp. pneumoniae MGH 78578 | pKPN4 | 5.00E-35 | 95.05 | 101 | 45 |
| NC_009651.1 | Klebsiella pneumoniae subsp. pneumoniae MGH 78578 | pKPN5 | 1.00E-36 | 97.98 | 91 | 84 |
| NC_009652.1 | Klebsiella pneumoniae subsp. pneumoniae MGH 78578 | pKPN6 | 2.00E-36 | 95.05 | 98 | 37 |
| NC_009653.1 | Klebsiella pneumoniae subsp. pneumoniae MGH 78578 | pKPN7 | 3.00E-33 | 95.05 | 90 | 14 |
| NC_003789.1 | Klebsiella sp. KCL-2 | pMGD2 | 2.00E-36 | 95.05 | 93 | 80 |
| NC_015213.1 | Lactobacillus acidophilus 30SC | pRKC30SC1 | 1.00E-41 | 99.01 | 100 | 22 |
| NC_015322.1 | Lactobacillus amylovorus GRL 1112 | 2 | 1.00E-42 | 100 | 101 | 2 |
| NC_015319.1 | Lactobacillus amylovorus GRL 1112 | 1 | 7.00E-34 | 95.88 | 92 | 83 |
| NC_017471.1 | Lactobacillus amylovorus GRL1118 | 1 | 2.00E-36 | 95.05 | 91 | 66 |
| NC_017472.1 | Lactobacillus amylovorus GRL1118 | 2 | 3.00E-30 | 95 | 90 | 1338 |
| NC_012550.1 | Lactobacillus brevis | pLB925A03 | 2.00E-41 | 99.01 | 101 | 2 |
| NC_005952.1 | Lactobacillus brevis | pRH45II | 3.00E-37 | 96.04 | 100 | 34 |
| NC_008498.1 | Lactobacillus brevis ATCC 367 | 1 | 7.00E-36 | 95.92 | 98 | 8 |
| NC_008499.1 | Lactobacillus brevis ATCC 367 | 2 | 8.00E-40 | 98.02 | 101 | 4 |
| NC_020820.1 | Lactobacillus brevis KB290 | pKB290-1 | 2.00E-35 | 96.04 | 95 | 9 |
| NC_020821.1 | Lactobacillus brevis KB290 | pKB290-2 | 1.00E-36 | 96.04 | 98 | 21 |
| NC_020822.1 | Lactobacillus brevis KB290 | pKB290-4 | 3.00E-37 | 96.04 | 96 | 37 |
| NC_020823.1 | Lactobacillus brevis KB290 | pKB290-5 | 2.00E-37 | 97.92 | 96 | 7 |
| NC_020824.1 | Lactobacillus brevis KB290 | pKB290-7 | 2.00E-40 | 98.02 | 101 | 2 |
| NC_020826.1 | Lactobacillus brevis KB290 | pKB290-3 | 8.00E-40 | 98.02 | 99 | 9 |
| NC_020828.1 | Lactobacillus brevis KB290 | pKB290-8 | 2.00E-41 | 99.01 | 101 | 4 |
| NC_018611.1 | Lactobacillus buchneri CD034 | pCD034-3 | 7.00E-36 | 95.05 | 97 | 22 |
| NC_015421.1 | Lactobacillus buchneri NRRL B-30929 | pLBUC03 | 9.00E-40 | 99.01 | 95 | 20 |
| NC_015429.1 | Lactobacillus buchneri NRRL B-30929 | pLBUC02 | 9.00E-36 | 95.05 | 95 | 24 |
| NC_015420.1 | Lactobacillus buchneri NRRL B-30929 | pLBUC01 | 5.00E-38 | 97.03 | 101 | 8 |
| NC_008502.1 | Lactobacillus casei ATCC 334 | 1 | 8.00E-39 | 97.98 | 98 | 7 |
| NC_017476.1 | Lactobacillus casei BD-II | pBD-II | 1.00E-34 | 95.05 | 91 | 6 |
| NC_017475.1 | Lactobacillus casei LC2W | pLC2W | 2.00E-35 | 95.05 | 90 | 81 |
| NC_021722.1 | Lactobacillus casei LOCK919 | pLOCK919 | 3.00E-37 | 96.04 | 101 | 12 |
| NC_011352.1 | Lactobacillus casei str. Zhang | plca36 | 3.00E-33 | 95.05 | 90 | 225 |
| NC_016975.1 | Lactobacillus casei strain TISTR1341 | pRCEID7.6 | 4.00E-36 | 98.9 | 91 | 10 |
| NC_020057.1 | Lactobacillus casei W56 | pW56 | 4.00E-39 | 98 | 100 | 2 |
| NC_003320.2 | Lactobacillus curvatus strain CRL705 | pRC18 | 1.00E-35 | 95.05 | 101 | 24 |
| NC_004849.1 | Lactobacillus delbrueckii subsp. lactis | pJBL2 | 2.00E-41 | 99.01 | 101 | 2 |
| NC_011798.1 | Lactobacillus farciminis KCTC 3681 | pLF24 | 1.00E-43 | 100 | 93 | 2 |
| NC_004566.1 | Lactobacillus fermentum | pLME300 | 4.00E-40 | 98.02 | 99 | 4 |
| NC_004947.1 | Lactobacillus fermentum | pKC5b | 1.00E-40 | 98.02 | 99 | 21 |
| NC_011839.1 | Lactobacillus gasseri | pLgLA39 | 2.00E-33 | 95.05 | 94 | 71 |
| NC_002102.1 | Lactobacillus helveticus DSM 20075 | pLH1 | 4.00E-34 | 97.78 | 90 | 6 |
| NC_017468.1 | Lactobacillus helveticus H10 | pH10 | 1.00E-35 | 95.05 | 92 | 71 |
| NC_014386.1 | Lactobacillus helveticus R0052 | pIR52-1 | 4.00E-41 | 99.01 | 97 | 4 |
| NC_013505.1 | Lactobacillus johnsonii FI9785 | p9785L | 1.00E-32 | 95.1 | 91 | 20 |
| NC_015598.1 | Lactobacillus kefiranofaciens ZW3 | pWW1 | 2.00E-30 | 95 | 90 | 113 |
| NC_015603.1 | Lactobacillus kefiranofaciens ZW3 | pWW2 | 2.00E-35 | 95.05 | 91 | 197 |
| NC_010913.1 | Lactobacillus paracasei | pMA3 | 2.00E-38 | 100 | 92 | 2 |
| NC_013543.1 | Lactobacillus paracasei subsp. paracasei | pCD01 | 2.00E-33 | 95.05 | 92 | 25 |
| NC_013544.1 | Lactobacillus paracasei subsp. paracasei | pCD02 | 4.00E-37 | 96 | 90 | 40 |
| NC_012222.2 | Lactobacillus paracasei subsp. paracasei | pSJ2-8 | 3.00E-41 | 99.01 | 101 | 20 |
| NC_022114.1 | Lactobacillus paracasei subsp. paracasei 8700:2 | 1 | 7.00E-39 | 97.98 | 99 | 7 |
| NC_022123.1 | Lactobacillus paracasei subsp. paracasei 8700:2 | 2 | 2.00E-32 | 95.05 | 90 | 15 |
| NC_011101.1 | Lactobacillus plantarum | pLTK13 | 7.00E-34 | 96.74 | 99 | 49 |
| NC_003894.1 | Lactobacillus plantarum | pLP9000 | 2.00E-34 | 95.88 | 97 | 8 |
| NC_012628.1 | Lactobacillus plantarum | pLFE1 | 5.00E-38 | 98.02 | 91 | 146 |
| NC_013789.1 | Lactobacillus plantarum | pXY3 | 4.00E-37 | 96.04 | 101 | 1 |
| NC_019371.1 | Lactobacillus plantarum | pG6303 | 1.00E-38 | 97.03 | 101 | 4 |
| NC_019379.1 | Lactobacillus plantarum | pG6302 | 1.00E-37 | 96.04 | 99 | 6 |
| NC_021515.1 | Lactobacillus plantarum 16 | Lp16A | 9.00E-38 | 96.04 | 95 | 5 |
| NC_021516.1 | Lactobacillus plantarum 16 | Lp16C | 2.00E-34 | 95.05 | 91 | 43 |
| NC_021517.1 | Lactobacillus plantarum 16 | Lp16E | 1.00E-38 | 97.98 | 99 | 13 |
| NC_021518.1 | Lactobacillus plantarum 16 | Lp16F | 1.00E-39 | 98.02 | 101 | 13 |
| NC_021519.1 | Lactobacillus plantarum 16 | Lp16H | 7.00E-38 | 97.03 | 101 | 4 |
| NC_021520.1 | Lactobacillus plantarum 16 | Lp16L | 5.00E-40 | 98 | 100 | 2 |
| NC_021525.1 | Lactobacillus plantarum 16 | Lp16B | 3.00E-42 | 99.01 | 101 | 1 |
| NC_021526.1 | Lactobacillus plantarum 16 | Lp16D | 3.00E-33 | 95.96 | 92 | 20 |
| NC_021527.1 | Lactobacillus plantarum 16 | Lp16G | 1.00E-40 | 99.01 | 101 | 4 |
| NC_021528.1 | Lactobacillus plantarum 16 | Lp16I | 3.00E-41 | 99.01 | 101 | 10 |
| NC_004944.1 | Lactobacillus plantarum 5057 | pMD5057 | 6.00E-36 | 95.05 | 99 | 109 |
| NC_006278.1 | Lactobacillus plantarum NC7 | p256 | 7.00E-39 | 97.96 | 98 | 16 |
| NC_021226.1 | Lactobacillus plantarum subsp. plantarum P-8 | LBPp3 | 9.00E-40 | 98.02 | 100 | 6 |
| NC_021225.1 | Lactobacillus plantarum subsp. plantarum P-8 | LBPp2 | 1.00E-32 | 95.92 | 90 | 35 |
| NC_021227.1 | Lactobacillus plantarum subsp. plantarum P-8 | LBPp5 | 3.00E-41 | 99.01 | 101 | 16 |
| NC_021228.1 | Lactobacillus plantarum subsp. plantarum P-8 | LBPp6 | 8.00E-41 | 98.02 | 100 | 22 |
| NC_021233.1 | Lactobacillus plantarum subsp. plantarum P-8 | LBPp1 | 3.00E-35 | 95.05 | 95 | 47 |
| NC_021234.1 | Lactobacillus plantarum subsp. plantarum P-8 | LBPp4 | 3.00E-38 | 97.03 | 100 | 23 |
| NC_014558.2 | Lactobacillus plantarum subsp. plantarum ST-III | pST-III | 3.00E-34 | 95.05 | 92 | 67 |
| NC_006377.1 | Lactobacillus plantarum WCFS1 | pWCFS103 | 4.00E-31 | 95.56 | 90 | 8 |
| NC_021903.1 | Lactobacillus plantarum ZJ316 | pLP-ZJ101 | 7.00E-43 | 100 | 101 | 2 |
| NC_021904.1 | Lactobacillus plantarum ZJ316 | pLP-ZJ102 | 4.00E-34 | 95.79 | 95 | 8 |
| NC_021912.1 | Lactobacillus plantarum ZJ316 | pLP-ZJ103 | 2.00E-35 | 95.05 | 94 | 52 |
| NC_003528.1 | Lactobacillus reuteri | pTE44 | 2.00E-38 | 98.02 | 90 | 82 |
| NC_004532.1 | Lactobacillus reuteri endogenous |  | 2.00E-43 | 100 | 101 | 1 |
| NC_021495.1 | Lactobacillus reuteri I5007 | pLRI02 | 8.00E-36 | 95.05 | 92 | 54 |
| NC_021496.1 | Lactobacillus reuteri I5007 | pLRI03 | 2.00E-34 | 97.03 | 91 | 10 |
| NC_021497.1 | Lactobacillus reuteri I5007 | pLRI05 | 7.00E-36 | 95.05 | 94 | 65 |
| NC_021503.1 | Lactobacillus reuteri I5007 | pLRI01 | 1.00E-32 | 95 | 90 | 152 |
| NC_021504.1 | Lactobacillus reuteri I5007 | pLRI04 | 2.00E-34 | 95.05 | 90 | 179 |
| NC_015701.1 | Lactobacillus reuteri SD2112 | pLR584 | 1.00E-33 | 95.74 | 91 | 210 |
| NC_010603.1 | Lactobacillus reuteri strain ATCC 55730 | pLR581 | 1.00E-31 | 95.05 | 90 | 1922 |
| NC_010621.1 | Lactobacillus reuteri strain ATCC 55730 | pLR585 | 8.00E-35 | 96.77 | 92 | 166 |
| NC_011223.1 | Lactobacillus rhamnosus HN001 | pLR001 | 2.00E-41 | 99.01 | 101 | 10 |
| NC_011225.1 | Lactobacillus rhamnosus HN001 | pLR002 | 2.00E-35 | 95.05 | 98 | 42 |
| NC_013200.1 | Lactobacillus rhamnosus Lc 705 | pLC1 | 3.00E-35 | 95.05 | 92 | 69 |
| NC_010936.1 | Lactobacillus sakei | pYSI8 | 2.00E-43 | 100 | 96 | 4 |
| NC_004942.1 | Lactobacillus sakei | pRV500 | 3.00E-40 | 98.02 | 100 | 6 |
| NC_011652.1 | Lactobacillus sakei strain BM5 | pYC2 | 2.00E-39 | 97.03 | 101 | 2 |
| NC_017479.1 | Lactobacillus salivarius CECT 5713 | pHN1 | 9.00E-34 | 95 | 90 | 591 |
| NC_017499.1 | Lactobacillus salivarius CECT 5713 | pHN3 | 5.00E-33 | 95 | 92 | 444 |
| NC_017480.1 | Lactobacillus salivarius CECT 5713 | pHN2 | 2.00E-36 | 95.05 | 93 | 85 |
| NC_006529.1 | Lactobacillus salivarius UCC118 | pSF118-20 | 2.00E-37 | 98.02 | 91 | 15 |
| NC_006530.1 | Lactobacillus salivarius UCC118 | pSF118-44 | 2.00E-35 | 95.05 | 92 | 155 |
| NC_007930.1 | Lactobacillus salivarius UCC118 | pMP118 | 1.00E-33 | 95.05 | 96 | 159 |
| NC_015980.1 | Lactobacillus sanfranciscensis TMW 1.1304 | pLS2 | 1.00E-35 | 96 | 100 | 11 |
| NC_015979.1 | Lactobacillus sanfranciscensis TMW 1.1304 | pLS1 | 4.00E-34 | 95.05 | 90 | 36 |
| NC_004957.1 | Lactobacillus sp. PC121B | p121BS | 2.00E-43 | 100 | 101 | 2 |
| NC_010540.1 | Lactococcus garvieae | pKL0018 | 2.00E-37 | 96.97 | 92 | 91 |
| NC_016970.1 | Lactococcus garvieae 21881 | pGL3 | 5.00E-37 | 96 | 90 | 43 |
| NC_016971.1 | Lactococcus garvieae 21881 | pGL4 | 1.00E-38 | 97.03 | 99 | 84 |
| NC_016982.1 | Lactococcus garvieae 21881 | pGL5 | 2.00E-39 | 98.02 | 99 | 12 |
| NC_002150.1 | Lactococcus lactis | pAH33 | 3.00E-36 | 95.05 | 92 | 57 |
| NC_008436.1 | Lactococcus lactis | pND324 | 7.00E-42 | 99.01 | 101 | 6 |
| NC_002799.1 | Lactococcus lactis | pCRL291.1 | 7.00E-37 | 96.04 | 93 | 18 |
| NC_010901.1 | Lactococcus lactis | pNP40 | 7.00E-37 | 96.04 | 94 | 88 |
| NC_011610.1 | Lactococcus lactis | pKL001 | 4.00E-35 | 95.83 | 96 | 9 |
| NC_013551.1 | Lactococcus lactis | pSK11B | 2.00E-37 | 98.04 | 94 | 8 |
| NC_004980.1 | Lactococcus lactis | pWC1 | 7.00E-41 | 98.02 | 101 | 12 |
| NC_017500.1 | Lactococcus lactis | pSK11P | 1.00E-40 | 99.01 | 101 | 2 |
| NC_002192.1 | Lactococcus lactis | pWV01 | 5.00E-41 | 98.02 | 101 | 2 |
| NC_007191.1 | Lactococcus lactis cremoris 712 | pAG6 | 4.00E-37 | 97.03 | 90 | 118 |
| NC_004847.1 | Lactococcus lactis cremoris HP | pHP003 | 2.00E-37 | 96.04 | 93 | 62 |
| NC_002137.1 | Lactococcus lactis cremoris NIZO B40 | pNZ4000 | 2.00E-33 | 95 | 90 | 222 |
| NC_003101.1 | Lactococcus lactis CRL1127 | pCRL1127 | 3.00E-38 | 97 | 97 | 78 |
| NC_002798.1 | Lactococcus lactis DCH-4 | pSRQ700 | 2.00E-40 | 98.02 | 96 | 10 |
| NC_001949.1 | Lactococcus lactis DPC3147 | pMRC01 | 4.00E-33 | 95.05 | 90 | 136 |
| NC_000906.2 | Lactococcus lactis IL964 | pIL105 | 2.00E-41 | 99.01 | 100 | 36 |
| NC_004966.1 | Lactococcus lactis lactis bv. diacetylactis DPC220 | pAH82 | 4.00E-35 | 95 | 94 | 126 |
| NC_004164.2 | Lactococcus lactis lactis bv. diacetylactis DRC1 cryptic | pDR1-1 | 1.00E-41 | 99.01 | 100 | 10 |
| NC_004955.1 | Lactococcus lactis lactis IPLA 972 | pBL1 | 2.00E-39 | 97.03 | 97 | 39 |
| NC_002502.1 | Lactococcus lactis lactis UC317 | pCI305 | 4.00E-43 | 100 | 101 | 3 |
| NC_009435.1 | Lactococcus lactis NCDO 1867 | pGdh442 | 4.00E-34 | 95.05 | 90 | 302 |
| NC_019347.1 | Lactococcus lactis subsp. cremoris | pAF04 | 9.00E-41 | 98.02 | 101 | 10 |
| NC_019348.1 | Lactococcus lactis subsp. cremoris | pAF07 | 3.00E-43 | 100 | 101 | 2 |
| NC_019349.1 | Lactococcus lactis subsp. cremoris | pAF12 | 6.00E-36 | 95.05 | 91 | 41 |
| NC_019350.1 | Lactococcus lactis subsp. cremoris | pAF14 | 7.00E-36 | 95.05 | 93 | 166 |
| NC_019351.1 | Lactococcus lactis subsp. cremoris | pAF22 | 2.00E-38 | 97.03 | 101 | 6 |
| NC_019377.1 | Lactococcus lactis subsp. cremoris | pLP712 | 1.00E-34 | 95.15 | 90 | 67 |
| NC_017494.1 | Lactococcus lactis subsp. cremoris | pAW153 | 7.00E-39 | 97.03 | 101 | 11 |
| NC_017495.1 | Lactococcus lactis subsp. cremoris A76 | pQA518 | 7.00E-37 | 96 | 99 | 23 |
| NC_017496.1 | Lactococcus lactis subsp. cremoris A76 | pQA554 | 1.00E-33 | 95.05 | 91 | 446 |
| NC_017497.1 | Lactococcus lactis subsp. cremoris A76 | pQA504 | 2.00E-43 | 100 | 101 | 10 |
| NC_017493.1 | Lactococcus lactis subsp. cremoris A76 | pQA549 | 1.00E-33 | 96.77 | 92 | 17 |
| NC_008503.1 | Lactococcus lactis subsp. cremoris SK11 | 1 | 7.00E-36 | 95.05 | 101 | 28 |
| NC_008504.1 | Lactococcus lactis subsp. cremoris SK11 | 2 | 2.00E-40 | 98.02 | 99 | 44 |
| NC_008505.1 | Lactococcus lactis subsp. cremoris SK11 | 3 | 4.00E-31 | 95 | 90 | 232 |
| NC_008506.1 | Lactococcus lactis subsp. cremoris SK11 | 4 | 3.00E-34 | 95.05 | 90 | 471 |
| NC_008507.1 | Lactococcus lactis subsp. cremoris SK11 | 5 | 9.00E-35 | 96.81 | 91 | 6 |
| NC_019430.1 | Lactococcus lactis subsp. cremoris UC509.9 | pCIS8 | 5.00E-34 | 95.83 | 91 | 51 |
| NC_019431.1 | Lactococcus lactis subsp. cremoris UC509.9 | pCIS7 | 3.00E-35 | 95.05 | 92 | 84 |
| NC_019432.1 | Lactococcus lactis subsp. cremoris UC509.9 | pCIS5 | 1.00E-38 | 97.03 | 100 | 43 |
| NC_019438.1 | Lactococcus lactis subsp. cremoris UC509.9 | pCIS1 | 7.00E-39 | 99.01 | 101 | 3 |
| NC_019436.1 | Lactococcus lactis subsp. cremoris UC509.9 | pCIS6 | 2.00E-35 | 95.05 | 98 | 55 |
| NC_019434.1 | Lactococcus lactis subsp. cremoris UC509.9 | pCIS2 | 5.00E-39 | 97.03 | 99 | 26 |
| NC_019437.1 | Lactococcus lactis subsp. cremoris UC509.9 | pCIS4 | 4.00E-35 | 96.04 | 94 | 66 |
| NC_008594.1 | Lactococcus lactis subsp. lactis | pL2 | 1.00E-40 | 98.02 | 99 | 13 |
| NC_013783.1 | Lactococcus lactis subsp. lactis | pAR141 | 7.00E-44 | 100 | 101 | 4 |
| NC_015861.1 | Lactococcus lactis subsp. lactis | pIL3 | 3.00E-35 | 95.96 | 92 | 88 |
| NC_015862.1 | Lactococcus lactis subsp. lactis | pIL4 | 4.00E-33 | 95.05 | 91 | 207 |
| NC_015863.1 | Lactococcus lactis subsp. lactis | pIL5 | 4.00E-35 | 95.1 | 91 | 171 |
| NC_015864.1 | Lactococcus lactis subsp. lactis | pIL7 | 1.00E-36 | 96.04 | 90 | 129 |
| NC_015860.1 | Lactococcus lactis subsp. lactis | pIL1 | 8.00E-38 | 96.04 | 92 | 30 |
| NC_016042.1 | Lactococcus lactis subsp. lactis | pKP1 | 1.00E-33 | 95.05 | 90 | 100 |
| NC_019308.1 | Lactococcus lactis subsp. lactis | pIL6 | 3.00E-32 | 95 | 90 | 90 |
| NC_009137.1 | Lactococcus lactis subsp. lactis bv. diacetylactis | pDBORO | 4.00E-33 | 95.05 | 99 | 79 |
| NC_004652.1 | Lactococcus lactis subsp. lactis bv. diacetylactis | pS7a | 5.00E-35 | 97.03 | 91 | 20 |
| NC_015912.1 | Lactococcus lactis subsp. lactis bv. diacetylactis | pVF21 | 3.00E-37 | 96.04 | 99 | 54 |
| NC_015902.1 | Lactococcus lactis subsp. lactis bv. diacetylactis | pVF50 | 3.00E-35 | 95.05 | 93 | 254 |
| NC_015900.1 | Lactococcus lactis subsp. lactis bv. diacetylactis | pVF18 | 1.00E-35 | 95.05 | 91 | 229 |
| NC_015901.1 | Lactococcus lactis subsp. lactis bv. diacetylactis | pVF22 | 1.00E-35 | 95.05 | 97 | 106 |
| NC_004163.1 | Lactococcus lactis subsp. lactis bv. diacetylactis cryptic | pDR1-1B | 5.00E-35 | 97.8 | 91 | 7 |
| NC_017484.1 | Lactococcus lactis subsp. lactis CV56 | pCV56C | 3.00E-32 | 95.15 | 92 | 184 |
| NC_017483.1 | Lactococcus lactis subsp. lactis CV56 | pCV56A | 5.00E-37 | 96.04 | 93 | 43 |
| NC_017487.1 | Lactococcus lactis subsp. lactis CV56 | pCV56B | 3.00E-32 | 95.05 | 90 | 101 |
| NC_009751.1 | Lactococcus lactis subsp. lactis K214 | pK214 | 5.00E-35 | 95.05 | 90 | 137 |
| NC_013657.1 | Lactococcus lactis subsp. lactis KF147 | pKF147A | 3.00E-32 | 95.05 | 90 | 216 |
| NC_002748.1 | Lactococcus lactis subsp. lactis strain MJC15 | pCD4 | 6.00E-33 | 95.96 | 92 | 13 |
| NC_004960.1 | Lactococcus lactis W-1 | pSRQ800 | 4.00E-43 | 100 | 101 | 12 |
| NC_004959.1 | Lactococcus lactis W-37 | pSRQ900 | 1.00E-37 | 96.04 | 101 | 24 |
| NC_010370.1 | Laribacter hongkongensis | pHLHK22 | 7.00E-43 | 100 | 96 | 8 |
| NC_006628.1 | Laribacter hongkongensis | pHLHK8 | 8.00E-39 | 97.03 | 101 | 4 |
| NC_006365.1 | Legionella pneumophila str. Paris | pLPP | 1.00E-36 | 96.04 | 101 | 2 |
| NC_018141.1 | Legionella pneumophila subsp. pneumophila str. Lorraine | pLELO | 6.00E-42 | 100 | 101 | 2 |
| NC_020522.1 | Legionella pneumophila subsp. pneumophila str. Philadelphia 1 |  | 1.00E-40 | 99.01 | 101 | 4 |
| NC_023136.1 | Leisingera methylohalidivorans DSM 14336 strain MB2, DSM 14336 | unnamed | 1.00E-34 | 95.92 | 98 | 2 |
| NC_018674.1 | Leuconostoc carnosum JB16 | pKLC1 | 5.00E-40 | 98.02 | 100 | 28 |
| NC_018675.1 | Leuconostoc carnosum JB16 | pKLC3 | 3.00E-33 | 95.05 | 99 | 31 |
| NC_018699.1 | Leuconostoc carnosum JB16 | pKLC4 | 2.00E-35 | 95.05 | 101 | 14 |
| NC_010312.2 | Leuconostoc citreum | pCB42 | 8.00E-42 | 99.01 | 101 | 12 |
| NC_004528.1 | Leuconostoc citreum | pLC22R | 5.00E-36 | 95.05 | 98 | 6 |
| NC_010466.1 | Leuconostoc citreum KM20 | pLCK2 | 1.00E-33 | 95.74 | 93 | 40 |
| NC_010467.1 | Leuconostoc citreum KM20 | pLCK3 | 2.00E-38 | 97.03 | 100 | 14 |
| NC_010469.1 | Leuconostoc citreum KM20 | pLCK4 | 2.00E-35 | 95 | 92 | 160 |
| NC_010470.1 | Leuconostoc citreum KM20 | pLCK1 | 4.00E-31 | 95.05 | 90 | 108 |
| NC_014132.1 | Leuconostoc kimchii IMSNU 11154 | LkipL4704 | 1.00E-35 | 95.05 | 97 | 19 |
| NC_014133.1 | Leuconostoc kimchii IMSNU 11154 | LkipL4719 | 1.00E-35 | 95.05 | 99 | 35 |
| NC_014134.1 | Leuconostoc kimchii IMSNU 11154 | LkipL4726 | 1.00E-35 | 95.05 | 97 | 54 |
| NC_004992.1 | Leuconostoc lactis | pCI411 | 5.00E-36 | 96.84 | 95 | 2 |
| NC_008496.1 | Leuconostoc mesenteroides subsp. mesenteroides ATCC 8293 | pLEUM1 | 1.00E-37 | 95.24 | 98 | 25 |
| NC_016820.1 | Leuconostoc mesenteroides subsp. mesenteroides J18 | pKLE02 | 8.00E-40 | 98.02 | 94 | 16 |
| NC_016827.1 | Leuconostoc mesenteroides subsp. mesenteroides J18 | pKLE01 | 4.00E-37 | 96.04 | 94 | 24 |
| NC_016828.1 | Leuconostoc mesenteroides subsp. mesenteroides J18 | pKLE04 | 2.00E-37 | 96.04 | 100 | 10 |
| NC_016821.1 | Leuconostoc mesenteroides subsp. mesenteroides J18 | pKLE03 | 2.00E-38 | 97.03 | 98 | 14 |
| NC_014496.1 | Listeria grayi | pLGUG1 | 3.00E-42 | 100 | 101 | 8 |
| NC_003383.1 | Listeria innocua Clip11262 | pLI100 | 8.00E-38 | 97.03 | 101 | 18 |
| NC_018888.1 | Listeria monocytogenes serotype 7 str. SLCC2482 | pLM7UG1 | 7.00E-36 | 98.91 | 92 | 33 |
| NC_022051.1 | Listeria monocytogenes strain J1926 |  | 7.00E-37 | 96.04 | 90 | 37 |
| NC_014255.1 | Listeria monocytogenes strain Lm1 | pLM33 | 7.00E-40 | 98.99 | 99 | 39 |
| NC_022045.1 | Listeria monocytogenes strain N1-011A |  | 7.00E-35 | 95.05 | 94 | 4 |
| NC_021828.1 | Listeria monocytogenes strain R2-502 |  | 2.00E-36 | 96.97 | 99 | 23 |
| NC_011995.1 | Macrococcus caseolyticus JCSC5402 | pMCCL1 | 3.00E-41 | 99.01 | 101 | 2 |
| NC_012003.1 | Macrococcus caseolyticus JCSC5402 | pMCCL8 | 4.00E-38 | 96.04 | 101 | 2 |
| NC_002637.1 | Mannheimia haemolytica R122 | pMHSCS1 | 2.00E-37 | 100 | 90 | 2 |
| NC_014213.1 | Meiothermus silvanus DSM 9946 | pMESIL01 | 2.00E-39 | 99 | 100 | 4 |
| NC_002679.1 | Mesorhizobium loti MAFF303099 | pMLa | 2.00E-34 | 95.05 | 94 | 4 |
| NC_008242.1 | Mesorhizobium sp. BNC1 | 1 | 2.00E-34 | 95.05 | 94 | 144 |
| NC_008243.1 | Mesorhizobium sp. BNC1 | 2 | 2.00E-34 | 95 | 100 | 28 |
| NC_019972.1 | Methanomethylovorans hollandica DSM 15978 | pMETHO01 | 6.00E-39 | 98.02 | 101 | 16 |
| NC_008826.1 | Methylibium petroleiphilum PM1 | RPME01 | 3.00E-34 | 95.05 | 101 | 4 |
| NC_012807.1 | Methylobacterium extorquens AM1 | p1META1 | 2.00E-42 | 100 | 101 | 4 |
| NC_012811.1 | Methylobacterium extorquens AM1 |  | 1.00E-32 | 95 | 91 | 301 |
| NC_011758.1 | Methylobacterium extorquens CM4 | pMCHL01 | 2.00E-34 | 95.05 | 101 | 14 |
| NC_012987.1 | Methylobacterium extorquens DM4 | p1METDI | 4.00E-32 | 95.7 | 93 | 8 |
| NC_012989.1 | Methylobacterium extorquens DM4 | p2METDI | 2.00E-42 | 100 | 101 | 2 |
| NC_011887.1 | Methylobacterium nodulans ORS 2060 | pMNOD02 | 4.00E-31 | 95.65 | 92 | 2 |
| NC_011892.1 | Methylobacterium nodulans ORS 2060 | pMNOD01 | 1.00E-30 | 95.05 | 91 | 10 |
| NC_010510.1 | Methylobacterium radiotolerans JCM 2831 | pMRAD01 | 1.00E-31 | 95 | 93 | 135 |
| NC_010517.1 | Methylobacterium radiotolerans JCM 2831 | pMRAD04 | 4.00E-38 | 97.03 | 101 | 2 |
| NC_004954.1 | Micrococcus sp. 28 | pSD10 | 9.00E-35 | 95 | 90 | 170 |
| NC_022599.1 | Micrococcus sp. V7 | pLMV7 | 2.00E-34 | 95 | 91 | 90 |
| NC_019760.1 | Microcoleus sp. PCC 7113 | pMIC7113.02 | 1.00E-30 | 95 | 90 | 194 |
| NC_016036.1 | Morganella morganii strain M203 | R485 | 7.00E-37 | 98.02 | 91 | 18 |
| NC_021279.1 | Mycobacterium abscessus subsp. bolletii 50594 | 2 | 4.00E-42 | 100 | 101 | 4 |
| NC_021278.1 | Mycobacterium abscessus subsp. bolletii 50594 | 1 | 8.00E-35 | 96.04 | 91 | 16 |
| NC_020994.1 | Mycobacterium abscessus subsp. bolletii INCQS 00594 | pMAB01 | 7.00E-36 | 95.05 | 91 | 440 |
| NC_018022.1 | Mycobacterium chubuense NBB4 | pMYCCH.01 | 3.00E-34 | 95.05 | 95 | 34 |
| NC_018023.1 | Mycobacterium chubuense NBB4 | pMYCCH.02 | 7.00E-35 | 95.05 | 101 | 4 |
| NC_009339.1 | Mycobacterium gilvum PYR-GCK | pMFLV01 | 2.00E-34 | 95.05 | 98 | 14 |
| NC_009341.1 | Mycobacterium gilvum PYR-GCK | pMFLV03 | 7.00E-37 | 100 | 90 | 4 |
| NC_014811.1 | Mycobacterium gilvum Spyr1 | pMSPYR101 | 4.00E-34 | 95 | 100 | 24 |
| NC_010604.1 | Mycobacterium marinum M | pMM23 | 3.00E-37 | 96.04 | 101 | 8 |
| NC_019958.1 | Mycobacterium smegmatis JS623 | pMYCSM02 | 1.00E-34 | 95.05 | 101 | 2 |
| NC_008703.1 | Mycobacterium sp. KMS | pMKMS01 | 1.00E-34 | 95.05 | 100 | 42 |
| NC_008147.1 | Mycobacterium sp. MCS | 1 | 2.00E-32 | 95 | 92 | 30 |
| NC_020275.1 | Mycobacterium yongonense 05-1390 | pMyong1 | 5.00E-36 | 96 | 90 | 2 |
| NC_020276.1 | Mycobacterium yongonense 05-1390 | pMyong2 | 2.00E-37 | 97.92 | 96 | 4 |
| NC_010881.1 | Neisseria gonorrhoeae | pSJ5.2 | 2.00E-38 | 100 | 92 | 12 |
| NC_014105.1 | Neisseria gonorrhoeae | pEP5289 | 4.00E-38 | 97.03 | 101 | 22 |
| NC_019211.1 | Neisseria gonorrhoeae | pEM1 | 2.00E-43 | 100 | 101 | 9 |
| NC_007959.1 | Nitrobacter hamburgensis X14 | 1 | 1.00E-34 | 95.05 | 100 | 8 |
| NC_007960.1 | Nitrobacter hamburgensis X14 | 2 | 2.00E-35 | 95.05 | 95 | 15 |
| NC_007961.1 | Nitrobacter hamburgensis X14 | 3 | 1.00E-37 | 97.03 | 101 | 6 |
| NC_008341.1 | Nitrosomonas eutropha C91 | 1 | 3.00E-35 | 95.05 | 100 | 45 |
| NC_008342.1 | Nitrosomonas eutropha C91 | 2 | 7.00E-37 | 96.04 | 99 | 2 |
| NC_015221.1 | Nitrosomonas sp. AL212 | pNAL21202 | 3.00E-35 | 95.05 | 96 | 6 |
| NC_007617.1 | Nitrosospira multiformis ATCC 25196 | 3 | 6.00E-43 | 100 | 101 | 2 |
| NC_006362.1 | Nocardia farcinica IFM 10152 | pNF1 | 8.00E-42 | 100 | 101 | 2 |
| NC_006363.1 | Nocardia farcinica IFM 10152 | pNF2 | 4.00E-35 | 95.05 | 97 | 10 |
| NC_008697.1 | Nocardioides sp. JS614 | pNOCA01 | 5.00E-34 | 95.05 | 92 | 15 |
| NC_015583.1 | Novosphingobium sp. PP1Y | Mpl | 4.00E-35 | 96 | 100 | 2 |
| NC_009669.1 | Ochrobactrum anthropi ATCC 49188 | pOANT01 | 4.00E-32 | 95.05 | 90 | 438 |
| NC_009670.1 | Ochrobactrum anthropi ATCC 49188 | pOANT02 | 5.00E-35 | 95.05 | 92 | 36 |
| NC_009671.1 | Ochrobactrum anthropi ATCC 49188 | pOANT03 | 5.00E-35 | 95.05 | 101 | 24 |
| NC_009672.1 | Ochrobactrum anthropi ATCC 49188 | pOANT04 | 5.00E-39 | 98.02 | 95 | 10 |
| NC_019553.1 | Oenococcus oeni | pOENI-1 | 4.00E-40 | 98.02 | 101 | 19 |
| NC_019554.1 | Oenococcus oeni | pOENI-1v2 | 2.00E-33 | 95.74 | 91 | 28 |
| NC_017536.1 | Oligotropha carboxidovorans OM4 | pHCG3B | 2.00E-40 | 99.01 | 101 | 2 |
| NC_017539.1 | Oligotropha carboxidovorans OM4 | pOC167B | 8.00E-35 | 95.05 | 101 | 11 |
| NC_019730.1 | Oscillatoria nigro-viridis PCC 7112 | pOSC7112.02 | 3.00E-36 | 96.04 | 101 | 2 |
| NC_016817.1 | Pantoea ananatis LMG 5342 | pPANA10 | 1.00E-34 | 95.05 | 101 | 10 |
| NC_014842.1 | Pantoea sp. At-9b | pPAT9B05 | 1.00E-37 | 97.03 | 101 | 2 |
| NC_019316.1 | Paracoccus aestuarii | pAES4 | 5.00E-40 | 98 | 98 | 2 |
| NC_019366.1 | Paracoccus aestuarii | pAES7 | 7.00E-36 | 95.05 | 101 | 2 |
| NC_022042.1 | Paracoccus aminophilus JCM 7686 | pAMI1 | 5.00E-32 | 95 | 90 | 587 |
| NC_022043.1 | Paracoccus aminophilus JCM 7686 | pAMI5 | 3.00E-31 | 95.65 | 92 | 4 |
| NC_022049.1 | Paracoccus aminophilus JCM 7686 | pAMI4 | 4.00E-31 | 95.05 | 90 | 6 |
| NC_022050.1 | Paracoccus aminophilus JCM 7686 | pAMI8 | 2.00E-31 | 95 | 90 | 121 |
| NC_010847.2 | Paracoccus aminophilus strain JCM 7686 | pAMI2 | 2.00E-37 | 96.04 | 99 | 8 |
| NC_013513.1 | Paracoccus aminophilus strain JCM 7686 | pAMI3 | 1.00E-35 | 95.05 | 97 | 51 |
| NC_014832.1 | Paracoccus aminophilus strain JCM 7686 | pAMI7 | 7.00E-38 | 97.03 | 92 | 76 |
| NC_008688.1 | Paracoccus denitrificans PD1222 | 1 | 7.00E-30 | 95 | 90 | 56 |
| NC_019289.1 | Paracoccus haeundaensis | pHAE1 | 2.00E-37 | 96 | 100 | 2 |
| NC_019356.1 | Paracoccus marcusii | pMOS6 | 1.00E-36 | 95.96 | 99 | 6 |
| NC_009753.1 | Paracoccus methylutens strain DM12 | pMTH1 | 6.00E-35 | 96.97 | 90 | 110 |
| NC_004160.1 | Paracoccus pantotrophus | pWKS1 | 3.00E-39 | 97.03 | 101 | 2 |
| NC_019255.1 | Pasteurella aerogenes | pCCK343 | 7.00E-38 | 96.04 | 95 | 12 |
| NC_012216.1 | Pasteurella multocida | pB1006 | 4.00E-35 | 95.05 | 90 | 1855 |
| NC_004771.1 | Pasteurella multocida | pJR1 | 6.00E-40 | 98.04 | 101 | 10 |
| NC_004772.1 | Pasteurella multocida | pJR2 | 1.00E-41 | 99.01 | 101 | 10 |
| NC_016973.1 | Pasteurella multocida | pCCK411 | 2.00E-43 | 100 | 101 | 44 |
| NC_006994.1 | Pasteurella multocida 381 | pCCK381 | 5.00E-43 | 100 | 101 | 2 |
| NC_001774.1 | Pasteurella multocida Pm1096 | pIG1 | 2.00E-42 | 99.01 | 101 | 15 |
| NC_010864.1 | Pediococcus acidilactici | pEOC01 | 9.00E-34 | 95.05 | 90 | 396 |
| NC_004832.1 | Pediococcus acidilactici H | pSMB74 | 2.00E-41 | 99.01 | 101 | 6 |
| NC_016608.1 | Pediococcus claussenii ATCC BAA-344 | pPECL-5 | 6.00E-35 | 95.88 | 93 | 19 |
| NC_016636.1 | Pediococcus claussenii ATCC BAA-344 | pPECL-3 | 3.00E-41 | 99.01 | 101 | 14 |
| NC_016606.1 | Pediococcus claussenii ATCC BAA-344 | pPECL-2 | 1.00E-43 | 100 | 101 | 2 |
| NC_016607.1 | Pediococcus claussenii ATCC BAA-344 | pPECL-4 | 1.00E-35 | 95.05 | 100 | 7 |
| NC_017017.1 | Pediococcus claussenii ATCC BAA-344 | pPECL-6 | 4.00E-34 | 96.77 | 93 | 29 |
| NC_017019.1 | Pediococcus claussenii ATCC BAA-344 | pPECL-8 | 4.00E-37 | 96.97 | 99 | 28 |
| NC_017018.1 | Pediococcus claussenii ATCC BAA-344 | pPECL-7 | 3.00E-41 | 99.01 | 101 | 28 |
| NC_002126.1 | Pediococcus pentosaceus | pMD136 | 9.00E-43 | 100 | 101 | 2 |
| NC_012031.1 | Pediococcus pentosaceus | pRS5 | 5.00E-36 | 95.05 | 101 | 10 |
| NC_008608.1 | Pelobacter propionicus DSM 2379 | pPRO2 | 5.00E-35 | 95.88 | 91 | 161 |
| NC_018421.1 | Phaeobacter gallaeciensis 2.10 | pPGA2_239 | 1.00E-33 | 95.05 | 96 | 4 |
| NC_023148.1 | Phaeobacter gallaeciensis DSM 26640 | pGal_B134 | 2.00E-36 | 96.04 | 100 | 52 |
| NC_011143.1 | Phenylobacterium zucineum HLK1 | unnamed | 3.00E-37 | 97.03 | 99 | 20 |
| NC_016983.1 | Photobacterium damselae subsp. damselae | pAQU1 | 2.00E-38 | 99.01 | 95 | 24 |
| NC_008757.1 | Polaromonas naphthalenivorans CJ2 | pPNAP01 | 2.00E-33 | 95 | 98 | 19 |
| NC_008758.1 | Polaromonas naphthalenivorans CJ2 | pPNAP02 | 8.00E-36 | 96.94 | 98 | 2 |
| NC_008760.1 | Polaromonas naphthalenivorans CJ2 | pPNAP04 | 1.00E-37 | 97.03 | 96 | 2 |
| NC_008761.1 | Polaromonas naphthalenivorans CJ2 | pPNAP05 | 4.00E-34 | 95.83 | 96 | 2 |
| NC_007949.1 | Polaromonas sp. JS666 | 1 | 3.00E-37 | 97.03 | 100 | 6 |
| NC_007950.1 | Polaromonas sp. JS666 | 2 | 6.00E-34 | 95 | 100 | 4 |
| NC_015258.1 | Polymorphum gilvum SL003B-26A1 | pSL003B | 2.00E-38 | 98.97 | 97 | 8 |
| NC_022111.1 | Prevotella sp. oral taxon 299 str. F0039 |  | 5.00E-30 | 95 | 90 | 195 |
| NC_021086.1 | Propionibacterium acnes HL096PA1 |  | 1.00E-40 | 99.01 | 101 | 6 |
| NC_011513.1 | Proteus mirabilis | pRPCMY | 7.00E-42 | 99.01 | 101 | 16 |
| NC_022197.1 | Proteus mirabilis strain BB1091 | pB1022 | 6.00E-41 | 98.02 | 101 | 21 |
| NC_003905.1 | Proteus vulgaris | Rts1 DNA | 2.00E-31 | 96.74 | 92 | 5 |
| NC_010643.1 | Providencia rettgeri | R7K | 2.00E-42 | 100 | 101 | 2 |
| NC_008357.1 | Pseudomonas aeruginosa | pBS228 | 4.00E-35 | 95.05 | 100 | 22 |
| NC_009739.1 | Pseudomonas aeruginosa | pMATVIM-7 | 1.00E-35 | 95.92 | 90 | 96 |
| NC_019202.1 | Pseudomonas aeruginosa | pKLC102 | 2.00E-40 | 99.01 | 101 | 10 |
| NC_022344.1 | Pseudomonas aeruginosa | pOZ176 | 8.00E-34 | 95 | 90 | 86 |
| NC_007100.1 | Pseudomonas aeruginosa | Rms149 | 3.00E-35 | 95.05 | 100 | 26 |
| NC_001621.1 | Pseudomonas aeruginosa | Birmingham IncP-alpha | 1.00E-40 | 99.01 | 101 | 4 |
| NC_020452.1 | Pseudomonas aeruginosa strain COL-1 | pNOR-2000 | 4.00E-35 | 95.05 | 90 | 40 |
| NC_022346.1 | Pseudomonas aeruginosa strain ST308 | pCOL-1 | 2.00E-35 | 95.05 | 90 | 148 |
| NC_005909.1 | Pseudomonas alcaligenes | pRA2 | 1.00E-37 | 98.02 | 92 | 26 |
| NC_004951.1 | Pseudomonas fulva | pNI10 | 2.00E-43 | 100 | 101 | 8 |
| NC_003350.1 | Pseudomonas putida | pWW0 | 2.00E-34 | 95 | 98 | 34 |
| NC_013176.1 | Pseudomonas putida | pW2 | 1.00E-40 | 99.01 | 101 | 16 |
| NC_015855.1 | Pseudomonas putida | pGRT1 | 6.00E-42 | 100 | 101 | 4 |
| NC_019906.1 | Pseudomonas putida HB3267 | pPC9 | 1.00E-41 | 100 | 99 | 4 |
| NC_008275.1 | Pseudomonas putida MT53 | pWW53 | 1.00E-37 | 97.03 | 99 | 2 |
| NC_004999.1 | Pseudomonas putida NCIB 9816-4 | pDTG1 | 4.00E-42 | 100 | 101 | 2 |
| NC_018746.1 | Pseudomonas putida ND6 | pND6-2 | 2.00E-34 | 95 | 100 | 6 |
| NC_023274.1 | Pseudomonas putida strain LD209 | pLD209 | 9.00E-40 | 98.02 | 97 | 26 |
| NC_004444.1 | Pseudomonas resinovorans | pCAR1 | 8.00E-42 | 100 | 101 | 2 |
| NC_004956.1 | Pseudomonas sp. ADP atrazine catabolic | pADP-1 | 2.00E-34 | 95 | 91 | 95 |
| NC_010891.1 | Pseudomonas sp. CT14 | pCT14 | 3.00E-35 | 95.05 | 90 | 94 |
| NC_005244.2 | Pseudomonas sp. ND6 | pND6-1 | 2.00E-38 | 97.03 | 100 | 23 |
| NC_022739.1 | Pseudomonas sp. VLB120 | pSTY | 6.00E-40 | 99.01 | 101 | 10 |
| NC_015314.1 | Pseudonocardia dioxanivorans CB1190 | pPSED01 | 6.00E-31 | 95.05 | 91 | 20 |
| NC_007968.1 | Psychrobacter cryohalolentis K5 | 1 | 5.00E-37 | 98.94 | 94 | 4 |
| NC_021158.1 | Psychrobacter maritimus | pKLH80 | 3.00E-35 | 95.05 | 90 | 114 |
| NC_019275.1 | Psychrobacter sp. DAB_AL32B | pP32BP1 | 5.00E-39 | 97.03 | 101 | 4 |
| NC_019276.1 | Psychrobacter sp. DAB_AL43B | pP43BP1 | 1.00E-40 | 98.02 | 101 | 2 |
| NC_021668.1 | Psychrobacter sp. G | PsyG_26 | 6.00E-40 | 98.02 | 101 | 2 |
| NC_016819.1 | Rahnella aquatilis CIP 78.65 = ATCC 33071 | pRahaq202 | 6.00E-35 | 95.05 | 101 | 4 |
| NC_017060.1 | Rahnella aquatilis HX2 | PRA1 | 1.00E-39 | 99.01 | 101 | 4 |
| NC_017807.1 | Rahnella aquatilis HX2 | PRA2 | 5.00E-38 | 97.03 | 101 | 2 |
| NC_017773.1 | Rahnella aquatilis HX2 | PRA2 | 7.00E-38 | 97.03 | 99 | 2 |
| NC_019203.1 | Rahnella sp. WMR42 | pHW42 | 2.00E-43 | 100 | 101 | 1 |
| NC_015062.1 | Rahnella sp. Y9602 | pRAHAQ01 | 1.00E-38 | 98.02 | 101 | 6 |
| NC_015063.1 | Rahnella sp. Y9602 | pRAHAQ02 | 2.00E-40 | 99.01 | 101 | 4 |
| NC_005241.1 | Ralstonia eutropha H16 | pHG1 | 7.00E-34 | 95.05 | 95 | 4 |
| NC_019318.1 | Ralstonia pickettii | p712 | 1.00E-39 | 98.02 | 95 | 22 |
| NC_012849.1 | Ralstonia pickettii 12D | pRp12D02 | 2.00E-30 | 95.05 | 90 | 77 |
| NC_014309.1 | Ralstonia solanacearum CFBP2957 | RCFBPv3_mp | 1.00E-33 | 95.05 | 98 | 4 |
| NC_017589.1 | Ralstonia solanacearum CMR15 | CMR15_mp | 2.00E-31 | 95 | 90 | 118 |
| NC_021745.1 | Ralstonia solanacearum FQY_4 |  | 2.00E-29 | 95 | 90 | 552 |
| NC_003296.1 | Ralstonia solanacearum GMI1000 | pGMI1000MP | 4.00E-32 | 97.03 | 90 | 11 |
| NC_017575.1 | Ralstonia solanacearum Po82 |  | 9.00E-34 | 95.05 | 99 | 6 |
| NC_014310.1 | Ralstonia solanacearum PSI07 | mpPSI07 | 9.00E-34 | 95.05 | 101 | 4 |
| NC_021911.1 | Rhizobium etli bv. mimosae str. Mim1 | pRetMIM1f | 4.00E-35 | 96 | 100 | 2 |
| NC_007762.1 | Rhizobium etli CFN 42 | p42a | 1.00E-33 | 95 | 94 | 18 |
| NC_007763.1 | Rhizobium etli CFN 42 | p42b | 9.00E-35 | 95.05 | 101 | 4 |
| NC_007764.1 | Rhizobium etli CFN 42 | p42c | 4.00E-34 | 95 | 100 | 2 |
| NC_007765.1 | Rhizobium etli CFN 42 | p42e | 2.00E-34 | 95.05 | 99 | 6 |
| NC_007766.1 | Rhizobium etli CFN 42 | p42f | 3.00E-34 | 95.05 | 101 | 2 |
| NC_004041.2 | Rhizobium etli CFN 42 symbiotic | p42d | 7.00E-33 | 95.05 | 92 | 5 |
| NC_010996.1 | Rhizobium etli CIAT 652 | pB | 4.00E-30 | 95.65 | 92 | 4 |
| NC_010998.1 | Rhizobium etli CIAT 652 | pA | 2.00E-34 | 95.05 | 100 | 2 |
| NC_012848.1 | Rhizobium leguminosarum bv. trifolii WSM1325 | pR132501 | 4.00E-34 | 95.05 | 90 | 5 |
| NC_012852.1 | Rhizobium leguminosarum bv. trifolii WSM1325 | pR132504 | 2.00E-34 | 95.05 | 99 | 4 |
| NC_012854.1 | Rhizobium leguminosarum bv. trifolii WSM1325 | pR132505 | 1.00E-34 | 95.05 | 101 | 2 |
| NC_012858.1 | Rhizobium leguminosarum bv. trifolii WSM1325 | pR132502 | 7.00E-36 | 96.04 | 101 | 2 |
| NC_011366.1 | Rhizobium leguminosarum bv. trifolii WSM2304 | pRLG202 | 2.00E-34 | 95.05 | 101 | 2 |
| NC_011368.1 | Rhizobium leguminosarum bv. trifolii WSM2304 | pRLG201 | 6.00E-34 | 95.05 | 91 | 4 |
| NC_011370.1 | Rhizobium leguminosarum bv. trifolii WSM2304 | pRLG203 | 8.00E-32 | 96.7 | 91 | 2 |
| NC_011371.1 | Rhizobium leguminosarum bv. trifolii WSM2304 | pRLG204 | 2.00E-33 | 95.83 | 96 | 2 |
| NC_008378.1 | Rhizobium leguminosarum bv. viciae 3841 | pRL12 | 2.00E-38 | 98.02 | 101 | 1 |
| NC_008379.1 | Rhizobium leguminosarum bv. viciae 3841 | pRL9 | 2.00E-34 | 95.05 | 101 | 4 |
| NC_008381.1 | Rhizobium leguminosarum bv. viciae 3841 | pRL10 | 5.00E-30 | 95.05 | 90 | 4 |
| NC_008382.1 | Rhizobium leguminosarum bv. viciae 3841 | pRL7 | 7.00E-32 | 95.65 | 101 | 5 |
| NC_008383.1 | Rhizobium leguminosarum bv. viciae 3841 | pRL8 | 3.00E-39 | 98.02 | 99 | 2 |
| NC_008384.1 | Rhizobium leguminosarum bv. viciae 3841 | pRL11 | 1.00E-33 | 95 | 100 | 6 |
| NC_022536.1 | Rhizobium sp. IRBG74 | IRBL74_p | 1.00E-33 | 95 | 99 | 12 |
| NC_020061.1 | Rhizobium tropici CIAT 899 | pRtrCIAT899b | 3.00E-34 | 95 | 99 | 7 |
| NC_020062.1 | Rhizobium tropici CIAT 899 | pRtrCIAT899c | 2.00E-29 | 95.05 | 90 | 20 |
| NC_020060.1 | Rhizobium tropici CIAT 899 | pRtrCIAT899a | 1.00E-34 | 95.05 | 99 | 8 |
| NC_004527.1 | Rhodobacter blasticus | pMG160 | 3.00E-34 | 95.05 | 101 | 1 |
| NC_014035.1 | Rhodobacter capsulatus SB 1003 | pRCB133 | 3.00E-33 | 95 | 93 | 8 |
| NC_007488.2 | Rhodobacter sphaeroides 2.4.1 | B | 1.00E-36 | 96.04 | 99 | 4 |
| NC_007490.2 | Rhodobacter sphaeroides 2.4.1 | D | 5.00E-35 | 95.05 | 101 | 2 |
| NC_009429.1 | Rhodobacter sphaeroides ATCC 17025 | pRSPA01 | 4.00E-31 | 95 | 92 | 44 |
| NC_009430.1 | Rhodobacter sphaeroides ATCC 17025 | pRSPA02 | 3.00E-30 | 95 | 90 | 209 |
| NC_009431.1 | Rhodobacter sphaeroides ATCC 17025 | pRSPA03 | 6.00E-35 | 95.05 | 101 | 2 |
| NC_011962.1 | Rhodobacter sphaeroides KD131 | pRSKD131A | 8.00E-35 | 95.05 | 101 | 2 |
| NC_006258.1 | Rhodococcus erythropolis | pRE8424 | 1.00E-40 | 98.02 | 101 | 2 |
| NC_022125.1 | Rhodococcus erythropolis CCM2595 | pRECF1 | 2.00E-34 | 95 | 100 | 20 |
| NC_005073.1 | Rhodococcus erythropolis linear | pBD2 | 4.00E-40 | 99.01 | 101 | 18 |
| NC_007486.1 | Rhodococcus erythropolis PR4 | pREC1 | 4.00E-42 | 100 | 101 | 12 |
| NC_007491.1 | Rhodococcus erythropolis PR4 | pREL1 | 1.00E-34 | 95.05 | 90 | 71 |
| NC_008271.1 | Rhodococcus jostii RHA1 | pRHL3 | 5.00E-34 | 95 | 100 | 2 |
| NC_012520.1 | Rhodococcus opacus B4 | pROB01 | 5.00E-31 | 95.65 | 92 | 2 |
| NC_023144.1 | Rhodococcus pyridinivorans SB3094 |  | 6.00E-40 | 99.01 | 98 | 6 |
| NC_013548.1 | Rhodopseudomonas palustris | pRPSZY | 2.00E-39 | 97.03 | 101 | 12 |
| NC_019261.1 | Riemerella anatipestifer | pRA0511 | 5.00E-43 | 100 | 101 | 2 |
| NC_015728.1 | Roseobacter litoralis Och 149 | pRLO149_83 | 2.00E-39 | 98.02 | 101 | 2 |
| NC_006569.1 | Ruegeria pomeroyi DSS-3 megaplasmid |  | 8.00E-34 | 95 | 100 | 4 |
| NC_004574.1 | Ruegeria sp. PR1b | pSD25 | 7.00E-35 | 95.05 | 100 | 6 |
| NC_008042.1 | Ruegeria sp. TM1040 | unnamed | 2.00E-34 | 95 | 94 | 90 |
| NC_008043.1 | Ruegeria sp. TM1040 |  | 1.00E-33 | 95.88 | 97 | 2 |
| NC_014824.1 | Ruminococcus albus 7 | pRUMAL01 | 7.00E-40 | 99.01 | 100 | 4 |
| NC_001398.1 | Saccharomyces cerevisiae A364A 2 |  | 3.00E-43 | 100 | 101 | 14 |
| NC_021871.1 | Salmonella bongori N268-08 | RM1 | 4.00E-36 | 96 | 95 | 8 |
| NC_003079.1 | Salmonella choleraesuis 79500 | pSFD10 | 2.00E-43 | 100 | 101 | 1 |
| NC_005862.1 | Salmonella enterica enterica sv Choleraesuis | cryptic | 3.00E-43 | 100 | 101 | 16 |
| NC_002638.1 | Salmonella enterica enterica sv Choleraesuis RF-1 | pKDSC50 | 3.00E-31 | 95.56 | 90 | 5 |
| NC_007208.1 | Salmonella enterica OU7025 | pOU1113 | 1.00E-40 | 99.01 | 101 | 1 |
| NC_021817.1 | Salmonella enterica subsp. enterica serovar Bareilly str. CFSAN000189 | unnamed | 2.00E-33 | 95.79 | 95 | 2 |
| NC_010499.1 | Salmonella enterica subsp. enterica serovar Brandenburg | pUO-SbR3 | 1.00E-41 | 99.01 | 101 | 65 |
| NC_010500.1 | Salmonella enterica subsp. enterica serovar Brandenburg | pUO-SbR5 | 5.00E-40 | 98.02 | 101 | 78 |
| NC_009981.1 | Salmonella enterica subsp. enterica serovar Choleraesuis | pMAK1 | 5.00E-39 | 100 | 96 | 2 |
| NC_010119.1 | Salmonella enterica subsp. enterica serovar Choleraesuis | pOU7519 | 6.00E-29 | 96.7 | 91 | 1 |
| NC_021819.1 | Salmonella enterica subsp. enterica Serovar Cubana str. CFSAN002050 |  | 1.00E-37 | 97.03 | 99 | 36 |
| NC_021845.1 | Salmonella enterica subsp. enterica Serovar Cubana str. CFSAN002050 |  | 4.00E-39 | 98.02 | 101 | 16 |
| NC_019134.1 | Salmonella enterica subsp. enterica serovar Derby | pSD4.0 | 2.00E-43 | 100 | 101 | 4 |
| NC_019135.1 | Salmonella enterica subsp. enterica serovar Derby | pSD4.6 | 6.00E-38 | 96.04 | 101 | 6 |
| NC_022376.1 | Salmonella enterica subsp. enterica serovar Derby str. T12 | pST12 | 2.00E-41 | 99.01 | 101 | 8 |
| NC_009980.1 | Salmonella enterica subsp. enterica serovar Dublin | pMAK2 | 2.00E-37 | 100 | 92 | 4 |
| NC_010421.1 | Salmonella enterica subsp. enterica serovar Dublin | pOU1114 | 2.00E-42 | 100 | 101 | 11 |
| NC_010422.1 | Salmonella enterica subsp. enterica serovar Dublin | pOU1115 | 3.00E-42 | 100 | 101 | 2 |
| NC_010716.1 | Salmonella enterica subsp. enterica serovar Dublin | IncW pIE321 | 1.00E-41 | 99.01 | 101 | 1 |
| NC_019105.1 | Salmonella enterica subsp. enterica serovar Dublin | pSD_88 | 8.00E-38 | 97.03 | 100 | 116 |
| NC_010860.1 | Salmonella enterica subsp. enterica serovar Enteritidis | pSE34 | 1.00E-42 | 100 | 101 | 2 |
| NC_022267.1 | Salmonella enterica subsp. enterica serovar Enteritidis strain S1400/94 | pS1400_89 | 4.00E-35 | 95.05 | 100 | 13 |
| NC_019114.1 | Salmonella enterica subsp. enterica serovar Heidelberg | pSH111_227 | 7.00E-39 | 98 | 100 | 3 |
| NC_019115.1 | Salmonella enterica subsp. enterica serovar Heidelberg | pSH146_65 | 3.00E-35 | 95.05 | 100 | 37 |
| NC_019117.1 | Salmonella enterica subsp. enterica serovar Heidelberg | pSH696_117 | 4.00E-41 | 99.01 | 101 | 6 |
| NC_019121.1 | Salmonella enterica subsp. enterica serovar Heidelberg | pSH111_166 | 4.00E-38 | 98.02 | 101 | 1 |
| NC_019128.1 | Salmonella enterica subsp. enterica serovar Heidelberg | pSH696_34 | 1.00E-42 | 100 | 101 | 3 |
| NC_019129.1 | Salmonella enterica subsp. enterica serovar Heidelberg | pSH146_32 | 5.00E-43 | 100 | 101 | 2 |
| NC_019131.1 | Salmonella enterica subsp. enterica serovar Heidelberg | pSH146_87 | 3.00E-41 | 99.01 | 101 | 10 |
| NC_019132.1 | Salmonella enterica subsp. enterica serovar Heidelberg | pSH1148_4.8 | 8.00E-36 | 95.1 | 94 | 34 |
| NC_021841.1 | Salmonella enterica subsp. enterica serovar Heidelberg str. 41578 | pSEEH1578_02 | 3.00E-38 | 97.03 | 99 | 5 |
| NC_021869.1 | Salmonella enterica subsp. enterica serovar Heidelberg str. 41578 | pSEEH1578_03 | 1.00E-40 | 98.02 | 101 | 18 |
| NC_021813.2 | Salmonella enterica subsp. enterica serovar Heidelberg str. CFSAN002069 | pCFSAN002069_01 | 5.00E-35 | 95.05 | 91 | 81 |
| NC_021842.2 | Salmonella enterica subsp. enterica serovar Heidelberg str. CFSAN002069 | pCFSAN002069_02 | 7.00E-41 | 99.01 | 101 | 6 |
| NC_011081.1 | Salmonella enterica subsp. enterica serovar Heidelberg str. SL476 | pSL476_91 | 8.00E-40 | 98.02 | 101 | 21 |
| NC_011082.1 | Salmonella enterica subsp. enterica serovar Heidelberg str. SL476 | pSL476_3 | 1.00E-39 | 99.01 | 94 | 16 |
| NC_019104.1 | Salmonella enterica subsp. enterica serovar Kentucky | pCS0010A_95 | 4.00E-42 | 100 | 101 | 2 |
| NC_011076.1 | Salmonella enterica subsp. enterica serovar Kentucky str. CVM29188 | pCVM29188_146 | 8.00E-37 | 96.04 | 101 | 7 |
| NC_011077.1 | Salmonella enterica subsp. enterica serovar Kentucky str. CVM29188 | pCVM29188_101 | 4.00E-42 | 100 | 101 | 1 |
| NC_011078.1 | Salmonella enterica subsp. enterica serovar Kentucky str. CVM29188 | pCVM29188_46 | 2.00E-35 | 95.05 | 101 | 13 |
| NC_022522.1 | Salmonella enterica subsp. enterica serovar Kentucky strain 1643/10 | p1643_10 | 2.00E-36 | 96.04 | 93 | 4 |
| NC_014003.1 | Salmonella enterica subsp. enterica serovar Newport | pSN11/00Kan | 3.00E-43 | 100 | 101 | 2 |
| NC_011079.1 | Salmonella enterica subsp. enterica serovar Newport str. SL254 | pSL254_3 | 4.00E-38 | 96.04 | 101 | 2 |
| NC_010894.1 | Salmonella enterica subsp. enterica serovar Paratyphi A | pGY1 | 9.00E-41 | 98.02 | 101 | 6 |
| NC_019112.1 | Salmonella enterica subsp. enterica serovar Pullorum | pSPUV | 3.00E-41 | 99.01 | 101 | 1 |
| NC_019102.1 | Salmonella enterica subsp. enterica serovar Pullorum | pSPI12 | 6.00E-37 | 96.04 | 101 | 1 |
| NC_011092.1 | Salmonella enterica subsp. enterica serovar Schwarzengrund str. CVM19633 | pCVM19633_110 | 3.00E-38 | 97.98 | 99 | 4 |
| NC_011422.1 | Salmonella enterica subsp. enterica serovar Typhi str. 404ty | pBSSB1 | 1.00E-35 | 95.05 | 99 | 12 |
| NC_003384.1 | Salmonella enterica subsp. enterica serovar Typhi str. CT18 | pHCM1 | 2.00E-37 | 97.03 | 101 | 4 |
| NC_003385.1 | Salmonella enterica subsp. enterica serovar Typhi str. CT18 | pHCM2 | 1.00E-37 | 97.03 | 94 | 8 |
| NC_016825.1 | Salmonella enterica subsp. enterica serovar Typhi str. P-stx-12 | unnamed | 3.00E-40 | 99.01 | 101 | 12 |
| NC_002056.1 | Salmonella enterica subsp. enterica serovar Typhimurium | pSC101 | 4.00E-43 | 100 | 101 | 13 |
| NC_009807.1 | Salmonella enterica subsp. enterica serovar Typhimurium | pTPqnrS-1a | 9.00E-33 | 95.65 | 92 | 8 |
| NC_003292.1 | Salmonella enterica subsp. enterica serovar Typhimurium | R46 | 9.00E-41 | 99.01 | 101 | 37 |
| NC_005014.1 | Salmonella enterica subsp. enterica serovar Typhimurium | R64 | 8.00E-40 | 99 | 100 | 2 |
| NC_015570.1 | Salmonella enterica subsp. enterica serovar Typhimurium | pSe-Kan | 1.00E-41 | 99.01 | 101 | 33 |
| NC_019111.1 | Salmonella enterica subsp. enterica serovar Typhimurium | pSal8934a | 1.00E-42 | 100 | 101 | 16 |
| NC_022372.1 | Salmonella enterica subsp. enterica serovar Typhimurium | pYT3 | 2.00E-40 | 99.01 | 101 | 4 |
| NC_006816.1 | Salmonella enterica subsp. enterica serovar Typhimurium | pU302L | 8.00E-38 | 100 | 93 | 12 |
| NC_006815.1 | Salmonella enterica subsp. enterica serovar Typhimurium G8430 | pU302S | 1.00E-43 | 100 | 101 | 4 |
| NC_017675.1 | Salmonella enterica subsp. enterica serovar Typhimurium str. ST4/74 | TY474p2 | 3.00E-41 | 99.01 | 101 | 13 |
| NC_016862.1 | Salmonella enterica subsp. enterica serovar Typhimurium str. T000240 | pSTMDT12_S | 4.00E-43 | 100 | 101 | 2 |
| NC_021157.1 | Salmonella enterica subsp. enterica serovar Typhimurium str. U288 | pSTU288-3 | 2.00E-43 | 100 | 101 | 2 |
| NC_023275.1 | Salmonella enterica subsp. enterica serovar Typhimurium strain 9134 | p9134 | 1.00E-31 | 96.67 | 90 | 11 |
| NC_023276.1 | Salmonella enterica subsp. enterica serovar Typhimurium strain 9134 | p9134dAT | 2.00E-38 | 97.03 | 93 | 2 |
| NC_021815.1 | Salmonella enterica subsp. enterica serovar Typhimurium var. 5- str. CFSAN001921 | unnamed | 5.00E-33 | 95 | 90 | 766 |
| NC_021816.1 | Salmonella enterica subsp. enterica serovar Typhimurium var. 5- str. CFSAN001921 | unnamed | 6.00E-44 | 100 | 101 | 1 |
| NC_021843.1 | Salmonella enterica subsp. enterica serovar Typhimurium var. 5- str. CFSAN001921 | unnamed | 3.00E-35 | 95.05 | 99 | 13 |
| NC_011214.1 | Salmonella enterica subsp. enterica serovar Virchow str. SL491 | pSL491_5 | 3.00E-43 | 100 | 101 | 4 |
| NT_187135.1 | Salmonella enterica subsp. enterica serovar Weltevreden str. 2007-60-3289-1 | pSW82 | 4.00E-35 | 95.05 | 99 | 28 |
| NC_011604.1 | Salmonella enterica subsp. enterica serovar Westhampton | pWES-1 | 6.00E-36 | 95.05 | 101 | 6 |
| NC_019125.1 | Salmonella enterica subsp. salamae | pSGSC3045-121 | 2.00E-39 | 98.02 | 101 | 4 |
| NC_003455.1 | Salmonella enteritidis | pP | 8.00E-42 | 99.01 | 101 | 2 |
| NC_003456.1 | Salmonella enteritidis | pK | 5.00E-38 | 96.04 | 99 | 5 |
| NC_003457.1 | Salmonella enteritidis | pC | 2.00E-43 | 100 | 101 | 2 |
| NC_019342.1 | Salmonella sp. 14 | p14-120 | 6.00E-35 | 95.05 | 101 | 4 |
| NC_019343.1 | Salmonella sp. 96A-29192 | p96A29192-65 | 5.00E-33 | 95.74 | 94 | 2 |
| NC_002305.1 | Salmonella typhi | R27 | 9.00E-35 | 95.05 | 94 | 73 |
| NC_013518.1 | Sebaldella termitidis ATCC 33386 | pSTERM01 | 9.00E-35 | 95 | 99 | 30 |
| NC_013519.1 | Sebaldella termitidis ATCC 33386 | pSTERM02 | 2.00E-36 | 95.96 | 99 | 2 |
| NC_021742.1 | Serratia liquefaciens ATCC 27592 |  | 5.00E-31 | 95 | 90 | 1481 |
| NC_010796.1 | Serratia marcescens | pRK10 | 5.00E-32 | 95.05 | 90 | 14 |
| NC_005211.1 | Serratia marcescens | R478 | 5.00E-40 | 99.01 | 101 | 2 |
| NC_019267.1 | Serratia marcescens | pRIO-5 | 2.00E-37 | 97.03 | 91 | 70 |
| NC_019344.1 | Serratia marcescens | R830b | 4.00E-35 | 95.05 | 98 | 56 |
| NC_020212.1 | Serratia marcescens WW4 | pSmWW4 | 1.00E-38 | 97.06 | 102 | 1 |
| NC_008573.1 | Shewanella sp. ANA-3 | 1 | 1.00E-34 | 95.05 | 90 | 178 |
| NC_010657.1 | Shigella boydii CDC 3083-94 | pBS512_33 | 5.00E-42 | 100 | 100 | 4 |
| NC_010660.1 | Shigella boydii CDC 3083-94 | pBS512_211 | 3.00E-41 | 100 | 100 | 6 |
| NC_007608.1 | Shigella boydii Sb227 | pSB4_227 | 6.00E-35 | 95.05 | 95 | 13 |
| NC_007607.1 | Shigella dysenteriae Sd197 | pSD1_197 | 7.00E-36 | 96 | 100 | 24 |
| NC_019197.1 | Shigella flexneri | pSF5 | 2.00E-35 | 95.05 | 95 | 34 |
| NC_017320.1 | Shigella flexneri 2002017 | pSFxv_2 | 3.00E-43 | 100 | 100 | 2 |
| NC_017321.1 | Shigella flexneri 2002017 | pSFxv_4 | 1.00E-39 | 98.02 | 101 | 1 |
| NC_017319.1 | Shigella flexneri 2002017 | pSFxv_1 | 9.00E-42 | 100 | 101 | 3 |
| NC_002773.1 | Shigella flexneri 2a | p2457TS2 | 1.00E-37 | 96.08 | 102 | 1 |
| NC_004851.1 | Shigella flexneri 2a str. 301 | pCP301 | 4.00E-34 | 98.89 | 90 | 8 |
| NC_002134.1 | Shigella flexneri 2b | R100 | 1.00E-36 | 96.04 | 98 | 23 |
| NC_019250.1 | Shigella flexneri strain 2a 301 | pSF301-3 | 6.00E-44 | 100 | 101 | 1 |
| NC_021922.1 | Shigella flexneri strain HN006 | pSFyv | 2.00E-40 | 98.02 | 101 | 2 |
| NC_008439.1 | Shigella sonnei | pKKTET7 | 1.00E-43 | 100 | 101 | 1 |
| NC_002809.1 | Shigella sonnei | ColJs | 2.00E-43 | 100 | 101 | 1 |
| NC_013727.1 | Shigella sonnei | pEG356 | 3.00E-36 | 99.01 | 90 | 21 |
| NC_020412.1 | Shigella sonnei | pDPT3 | 1.00E-37 | 96.04 | 101 | 2 |
| NC_020413.1 | Shigella sonnei | pDT4 | 2.00E-43 | 100 | 100 | 14 |
| NC_001378.1 | Shigella sonnei | pKYM | 5.00E-41 | 98.02 | 101 | 6 |
| NC_002122.1 | Shigella sonnei | P9 | 1.00E-41 | 100 | 100 | 22 |
| NC_016833.1 | Shigella sonnei 53G | A | 8.00E-41 | 99.01 | 101 | 4 |
| NC_016834.1 | Shigella sonnei 53G | E | 4.00E-43 | 100 | 100 | 12 |
| NC_022585.1 | Shigella sonnei eg211 | pDPT1 | 4.00E-35 | 95.83 | 94 | 2 |
| NC_019256.1 | Shigella sp. LN126 | pLN126_33 | 9.00E-39 | 99.01 | 94 | 144 |
| NC_019254.1 | Shigella sp. MO17 | pMO17_54 | 3.00E-35 | 95.05 | 91 | 106 |
| NC_016815.1 | Sinorhizobium fredii HH103 | pSfHH103e | 5.00E-31 | 95.05 | 90 | 27 |
| NC_016814.1 | Sinorhizobium fredii HH103 | pSfHH103c | 2.00E-36 | 96.04 | 101 | 7 |
| NC_016813.1 | Sinorhizobium fredii HH103 | pSfHH103a | 1.00E-35 | 95.05 | 99 | 20 |
| NC_016836.1 | Sinorhizobium fredii HH103 | pSfHH103b | 1.00E-34 | 95.88 | 97 | 8 |
| NT_187146.1 | Sinorhizobium fredii HH103 | pSfHH103d fragment 1 | 7.00E-39 | 97.03 | 101 | 2 |
| NT_187149.1 | Sinorhizobium fredii HH103 | pSfHH103d pfragment 4 | 5.00E-38 | 97.03 | 101 | 2 |
| NT_187150.1 | Sinorhizobium fredii HH103 | pSfHH103d fragment 5 | 5.00E-36 | 95.05 | 101 | 2 |
| NC_012586.1 | Sinorhizobium fredii NGR234 | pNGR234b | 2.00E-30 | 95 | 90 | 18 |
| NC_000914.2 | Sinorhizobium fredii NGR234 | pNGR234a | 1.00E-38 | 98.02 | 101 | 4 |
| NT_187151.1 | Sinorhizobium fredii USDA 257 | pUSDA257 fragment 2 | 6.00E-36 | 96.08 | 101 | 5 |
| NT_187169.1 | Sinorhizobium fredii USDA 257 | pUSDA257 fragment 19 | 6.00E-42 | 99.01 | 101 | 2 |
| NT_187164.1 | Sinorhizobium fredii USDA 257 | pUSDA257 fragment 4 | 2.00E-35 | 95.05 | 101 | 2 |
| NC_009620.1 | Sinorhizobium medicae WSM419 | pSMED01 | 7.00E-34 | 95.05 | 101 | 2 |
| NC_009621.1 | Sinorhizobium medicae WSM419 | pSMED02 | 2.00E-32 | 95.05 | 90 | 8 |
| NC_009622.1 | Sinorhizobium medicae WSM419 | pSMED03 | 4.00E-34 | 95 | 99 | 8 |
| NC_010865.1 | Sinorhizobium meliloti | pSmeSM11b | 3.00E-34 | 95 | 99 | 27 |
| NC_013545.1 | Sinorhizobium meliloti | pSmeSM11a | 7.00E-35 | 95.05 | 101 | 10 |
| NC_019313.1 | Sinorhizobium meliloti | pHRC017 | 1.00E-34 | 95.05 | 101 | 3 |
| NC_020527.1 | Sinorhizobium meliloti 2011 | pSymA | 6.00E-34 | 95.05 | 96 | 11 |
| NC_020560.1 | Sinorhizobium meliloti 2011 | pSymB | 8.00E-34 | 95.05 | 101 | 4 |
| NC_015592.1 | Sinorhizobium meliloti AK83 | pSINME02 | 1.00E-34 | 96.04 | 98 | 2 |
| NC_015597.1 | Sinorhizobium meliloti AK83 | pSINME01 | 3.00E-35 | 95.05 | 101 | 1 |
| NC_017324.1 | Sinorhizobium meliloti BL225C | pSINMEB01 | 7.00E-34 | 95.05 | 90 | 4 |
| NC_017323.1 | Sinorhizobium meliloti BL225C | pSINMEB02 | 3.00E-33 | 95 | 98 | 8 |
| NC_019849.1 | Sinorhizobium meliloti GR4 | pRmeGR4d | 2.00E-35 | 96.04 | 101 | 2 |
| NC_019846.1 | Sinorhizobium meliloti GR4 | pRmeGR4a | 8.00E-35 | 95.05 | 101 | 4 |
| NC_019847.1 | Sinorhizobium meliloti GR4 | pRmeGR4b | 1.00E-34 | 95.05 | 99 | 10 |
| NC_019848.1 | Sinorhizobium meliloti GR4 | pRmeGR4c | 5.00E-35 | 100 | 90 | 1 |
| NC_018682.1 | Sinorhizobium meliloti Rm41 | pRM41A | 1.00E-35 | 96 | 100 | 4 |
| NC_018683.1 | Sinorhizobium meliloti Rm41 | pSYMA | 2.00E-33 | 95 | 98 | 3 |
| NC_018701.1 | Sinorhizobium meliloti Rm41 | pSYMB | 5.00E-30 | 95.6 | 91 | 2 |
| NC_017326.1 | Sinorhizobium meliloti SM11 | pSmeSM11d | 7.00E-34 | 95.05 | 98 | 2 |
| NC_017327.1 | Sinorhizobium meliloti SM11 | pSmeSM11c | 1.00E-37 | 98 | 100 | 4 |
| NC_021209.1 | Sinorhizobium sp. M14 | pSinA | 1.00E-31 | 95 | 91 | 163 |
| NC_015595.1 | Sphingobium chlorophenolicum L-1 | pSPHCH01 | 5.00E-36 | 99.01 | 90 | 6 |
| NC_016000.1 | Sphingobium chungbukense strain DJ77 | pSY2 | 1.00E-35 | 95.05 | 101 | 2 |
| NC_019376.1 | Sphingobium fuliginis ATCC 27551 | pPDL2 | 4.00E-37 | 98.94 | 94 | 2 |
| NC_014007.1 | Sphingobium japonicum UT26S | pCHQ1 | 3.00E-34 | 95 | 97 | 14 |
| NC_015974.1 | Sphingobium sp. SYK-6 | pSLPG | 3.00E-39 | 98.02 | 90 | 14 |
| NC_008246.1 | Sphingobium yanoikuyae | pYAN-1 | 1.00E-40 | 98.02 | 101 | 2 |
| NC_008247.1 | Sphingobium yanoikuyae | pYAN-2 | 2.00E-38 | 97 | 100 | 4 |
| NC_007353.2 | Sphingomonas sp. A1 | pA1 | 2.00E-35 | 95.05 | 101 | 9 |
| NC_022235.1 | Sphingomonas sp. ERG5 | pCADAB1 | 2.00E-36 | 96.04 | 100 | 6 |
| NC_008308.1 | Sphingomonas sp. KA1 | pCAR3 | 2.00E-37 | 97.03 | 101 | 6 |
| NC_020542.1 | Sphingomonas sp. MM-1 | pISP0 | 5.00E-34 | 95 | 100 | 16 |
| NC_020544.1 | Sphingomonas sp. MM-1 | pISP3 | 2.00E-35 | 95.05 | 101 | 4 |
| NC_020562.1 | Sphingomonas sp. MM-1 | pISP1 | 4.00E-32 | 95.7 | 93 | 22 |
| NC_009507.1 | Sphingomonas wittichii RW1 | pSWIT01 | 1.00E-41 | 100 | 97 | 10 |
| NC_009508.1 | Sphingomonas wittichii RW1 | pSWIT02 | 6.00E-32 | 95.7 | 91 | 18 |
| NC_001763.1 | Staphylococcus aureus | J3358 | 1.00E-41 | 99.01 | 101 | 2 |
| NC_001767.1 | Staphylococcus aureus | pKH6 | 2.00E-43 | 100 | 101 | 2 |
| NC_002517.1 | Staphylococcus aureus | p21 | 9.00E-43 | 100 | 101 | 6 |
| NC_010077.1 | Staphylococcus aureus | EDINA | 1.00E-41 | 99.01 | 101 | 1 |
| NC_013319.1 | Staphylococcus aureus | pI258 | 1.00E-37 | 100 | 92 | 22 |
| NC_013321.1 | Staphylococcus aureus | SAP017A | 2.00E-33 | 96.81 | 94 | 1 |
| NC_013322.1 | Staphylococcus aureus | SAP019A | 6.00E-40 | 98.02 | 98 | 6 |
| NC_013324.1 | Staphylococcus aureus | SAP027A | 1.00E-42 | 100 | 101 | 2 |
| NC_013326.1 | Staphylococcus aureus | pWBG746 | 6.00E-41 | 99.01 | 101 | 1 |
| NC_013332.1 | Staphylococcus aureus | SAP052A | 7.00E-40 | 98.02 | 101 | 2 |
| NC_013341.1 | Staphylococcus aureus | SAP077B | 3.00E-40 | 98.02 | 101 | 2 |
| NC_013344.1 | Staphylococcus aureus | SAP082A | 3.00E-38 | 98.95 | 92 | 1 |
| NC_013347.1 | Staphylococcus aureus | pSK62 | 1.00E-34 | 96.77 | 101 | 1 |
| NC_013348.1 | Staphylococcus aureus | pSK156 | 5.00E-37 | 96.04 | 100 | 4 |
| NC_013349.1 | Staphylococcus aureus | SAP099B | 4.00E-40 | 98.02 | 101 | 2 |
| NC_013350.1 | Staphylococcus aureus | pWBG754 | 1.00E-43 | 100 | 96 | 2 |
| NC_013550.1 | Staphylococcus aureus | pBORa53 | 8.00E-43 | 100 | 101 | 3 |
| NC_005011.1 | Staphylococcus aureus | pMW2 | 9.00E-43 | 100 | 101 | 2 |
| NC_005565.1 | Staphylococcus aureus | pSN2 | 2.00E-38 | 96.04 | 101 | 2 |
| NC_001384.1 | Staphylococcus aureus | pUB110 | 2.00E-43 | 100 | 101 | 5 |
| NC_001391.1 | Staphylococcus aureus | pNS1 | 7.00E-44 | 100 | 101 | 1 |
| NC_001393.1 | Staphylococcus aureus | pT181 | 2.00E-43 | 100 | 101 | 1 |
| NC_021230.1 | Staphylococcus aureus strain 1 | pSA8589 | 3.00E-43 | 100 | 101 | 2 |
| NC_023278.1 | Staphylococcus aureus strain SA268 | pSA268 | 5.00E-40 | 98.02 | 101 | 3 |
| NC_017350.1 | Staphylococcus aureus subsp. aureus 11819-97 | p11819-97 | 4.00E-35 | 95.88 | 96 | 2 |
| NC_022126.1 | Staphylococcus aureus subsp. aureus 55/2053 |  | 4.00E-34 | 96.77 | 93 | 5 |
| NC_017346.1 | Staphylococcus aureus subsp. aureus ECT-R 2 | pLUH01 | 5.00E-40 | 97.03 | 101 | 1 |
| NC_002774.1 | Staphylococcus aureus subsp. aureus Mu50 | VRSAp | 1.00E-42 | 100 | 101 | 8 |
| NC_020565.1 | Staphylococcus aureus subsp. aureus ST228 | pI3T3 | 2.00E-32 | 95.65 | 101 | 1 |
| NC_020183.2 | Staphylococcus aureus subsp. aureus ST398 | pUR3912 | 1.00E-41 | 99.01 | 101 | 2 |
| NC_022598.1 | Staphylococcus aureus TY825 | pETB | 3.00E-42 | 100 | 101 | 4 |
| NC_008352.1 | Staphylococcus chromogenes | pLNU8 | 5.00E-41 | 98.02 | 100 | 2 |
| NC_008356.1 | Staphylococcus epidermidis | pLNU6 | 1.00E-36 | 95.92 | 98 | 4 |
| NC_003969.1 | Staphylococcus epidermidis | pSepCH | 1.00E-43 | 100 | 101 | 2 |
| NC_013378.1 | Staphylococcus epidermidis | SAP105B | 1.00E-40 | 99 | 100 | 4 |
| NC_013380.1 | Staphylococcus epidermidis | SAP106B | 2.00E-39 | 97.98 | 99 | 1 |
| NC_013381.1 | Staphylococcus epidermidis | SAP107A | 2.00E-35 | 95.05 | 101 | 6 |
| NC_013382.1 | Staphylococcus epidermidis | SAP107B | 5.00E-43 | 100 | 101 | 4 |
| NC_013383.1 | Staphylococcus epidermidis | SAP110A | 1.00E-42 | 100 | 101 | 10 |
| NC_013389.1 | Staphylococcus epidermidis | SAP108A | 5.00E-41 | 99.01 | 101 | 4 |
| NC_013390.1 | Staphylococcus epidermidis | SAP108B | 7.00E-40 | 97.98 | 101 | 1 |
| NC_013393.1 | Staphylococcus epidermidis | pSK105 | 1.00E-42 | 100 | 101 | 2 |
| NC_013395.1 | Staphylococcus epidermidis | pSK108 | 4.00E-36 | 95.96 | 99 | 5 |
| NC_013392.2 | Staphylococcus epidermidis | SAP108D | 6.00E-41 | 98.02 | 101 | 4 |
| NC_019303.1 | Staphylococcus epidermidis | pUR3036 | 1.00E-35 | 95.88 | 97 | 2 |
| NC_005003.1 | Staphylococcus epidermidis ATCC 12228 | pSE-12228-06 | 3.00E-43 | 100 | 101 | 2 |
| NC_005004.1 | Staphylococcus epidermidis ATCC 12228 | pSE-12228-05 | 1.00E-42 | 100 | 101 | 2 |
| NC_005005.1 | Staphylococcus epidermidis ATCC 12228 | pSE-12228-04 | 2.00E-37 | 96.04 | 99 | 6 |
| NC_005566.1 | Staphylococcus epidermidis SK398 | pSK639 | 2.00E-40 | 98.02 | 101 | 2 |
| NC_016139.1 | Staphylococcus hyicus | p9811071-1 | 7.00E-44 | 100 | 101 | 3 |
| NC_020237.1 | Staphylococcus hyicus | pSTE1 | 5.00E-43 | 100 | 101 | 6 |
| NC_016643.1 | Staphylococcus saprophyticus subsp. saprophyticus | pSSAP2 | 2.00E-36 | 96 | 97 | 19 |
| NC_007351.1 | Staphylococcus saprophyticus subsp. saprophyticus ATCC 15305 | pSSP1 | 5.00E-37 | 96.04 | 101 | 17 |
| NC_007352.1 | Staphylococcus saprophyticus subsp. saprophyticus ATCC 15305 | pSSP2 | 4.00E-35 | 95.1 | 101 | 15 |
| NC_015432.1 | Staphylococcus saprophyticus subsp. saprophyticus MS1146 | pSSAP1 | 6.00E-38 | 97.03 | 101 | 12 |
| NC_015173.2 | Staphylococcus simulans bv. staphylolyticus strain NRRL B-2628 | pACK2 | 1.00E-38 | 98.02 | 101 | 2 |
| NC_009130.1 | Staphylococcus sp. 693-2 | pLEW6932 | 6.00E-37 | 96.04 | 101 | 14 |
| NC_013371.1 | Staphylococcus sp. 693-2 | SAP008A | 1.00E-37 | 96.04 | 101 | 4 |
| NC_013387.1 | Staphylococcus sp. CDC25 | SAP018A | 1.00E-42 | 100 | 101 | 4 |
| NC_005207.3 | Staphylococcus warneri | pPI-1 | 1.00E-42 | 100 | 101 | 6 |
| NC_005208.1 | Staphylococcus warneri | pPI-2 | 1.00E-43 | 100 | 101 | 2 |
| NC_007166.1 | Staphylococcus warneri | pSW49 | 1.00E-42 | 99.01 | 101 | 1 |
| NC_020264.1 | Staphylococcus warneri SG1 | pvSw2 | 2.00E-41 | 99.01 | 101 | 2 |
| NC_020274.1 | Staphylococcus warneri SG1 | pvSw1 | 6.00E-37 | 96 | 100 | 64 |
| NC_001797.1 | Streptococcus agalactiae | pGB354 | 1.00E-36 | 96.91 | 94 | 58 |
| NC_002136.1 | Streptococcus agalactiae | pGB3634 | 2.00E-37 | 96.04 | 90 | 114 |
| NC_001380.1 | Streptococcus agalactiae | pLS1 | 2.00E-43 | 100 | 101 | 2 |
| NC_015973.1 | Streptococcus agalactiae strain GB2001 | pGB2001 | 3.00E-36 | 95.05 | 101 | 4 |
| NC_015971.1 | Streptococcus agalactiae strain GB2002 | pGB2002 | 3.00E-43 | 100 | 101 | 2 |
| NC_015219.1 | Streptococcus gallolyticus subsp. gallolyticus ATCC BAA-2069 | pSGG1 | 4.00E-34 | 95.05 | 90 | 1238 |
| NC_016837.1 | Streptococcus infantarius subsp. infantarius CJ18 | pSICJ18-1 | 2.00E-31 | 95.05 | 90 | 117 |
| NC_019365.1 | Streptococcus infantis | pSI01 | 6.00E-36 | 95.05 | 91 | 28 |
| NC_016750.1 | Streptococcus macedonicus ACA-DC 198 | pSMA198 | 6.00E-36 | 95.05 | 92 | 140 |
| NC_012642.1 | Streptococcus parasanguinis | pFW213 | 4.00E-36 | 95.05 | 100 | 26 |
| NC_015876.1 | Streptococcus pseudopneumoniae IS7493 | pDRPIS7493 | 2.00E-43 | 100 | 101 | 2 |
| NC_010230.1 | Streptococcus pyogenes | pDN281 | 2.00E-37 | 95.96 | 101 | 2 |
| NC_006979.1 | Streptococcus pyogenes | pSM19035 | 1.00E-36 | 96 | 100 | 44 |
| NC_010423.2 | Streptococcus pyogenes isolate 9116-03 | pRW35 | 1.00E-41 | 99.01 | 101 | 2 |
| NC_002140.1 | Streptococcus suis | pSSU1 | 1.00E-40 | 98.02 | 101 | 2 |
| NC_012923.1 | Streptococcus suis BM407 | pBM407 | 1.00E-35 | 95.05 | 101 | 30 |
| NC_002776.1 | Streptococcus thermophilus | pER13 | 1.00E-40 | 98.02 | 100 | 4 |
| NC_005322.1 | Streptococcus thermophilus | pSMQ308 | 2.00E-33 | 97.78 | 90 | 1 |
| NC_019231.1 | Streptococcus thermophilus | pK1002C2 | 2.00E-43 | 100 | 101 | 2 |
| NC_019232.1 | Streptococcus thermophilus | pK2007C6 | 7.00E-41 | 98.02 | 101 | 5 |
| NC_008501.1 | Streptococcus thermophilus LMD-9 | 2 | 3.00E-41 | 98.02 | 101 | 3 |
| NC_005323.1 | Streptococcus thermophilus SMQ-173 | pSMQ173b | 2.00E-43 | 100 | 101 | 4 |
| NC_000937.1 | Streptococcus thermophilus ST135 | pER35 | 5.00E-36 | 95.05 | 94 | 114 |
| NC_000938.1 | Streptococcus thermophilus ST136 | pER36 | 2.00E-43 | 100 | 99 | 2 |
| NC_004747.1 | Streptococcus thermophilus ST2-1 | pND103 | 1.00E-39 | 96.19 | 105 | 1 |
| NC_017585.1 | Streptomyces cattleya NRRL 8057 = DSM 46488 | pSCATT | 2.00E-29 | 95.05 | 90 | 10 |
| NC_023283.1 | Streptomyces sp. FR1 | pFRL3 | 4.00E-37 | 97.03 | 101 | 2 |
| NC_019329.1 | Sulfitobacter sp. DFL14 | pDFL14-10 | 2.00E-36 | 95.96 | 99 | 14 |
| NC_001898.1 | Tatumella citrea | pUCD5000 | 2.00E-43 | 100 | 101 | 1 |
| NC_015261.1 | Tetragenococcus halophilus | pHDC-RI | 9.00E-43 | 100 | 101 | 4 |
| NC_015255.1 | Tetragenococcus halophilus HO | pHDC-HO | 2.00E-42 | 100 | 101 | 2 |
| NC_019354.1 | Tetragenococcus muriaticus | pHDC-I-1 | 5.00E-40 | 98.02 | 100 | 11 |
| NC_011667.1 | Thauera sp. MZ1T | pTha01 | 4.00E-35 | 95.05 | 101 | 24 |
| NC_011961.1 | Thermomicrobium roseum DSM 5159 | unnamed | 3.00E-30 | 95 | 90 | 161 |
| NC_014975.1 | Thermus scotoductus SA-01 | pTSC8 | 4.00E-43 | 100 | 101 | 2 |
| NC_014154.1 | Thiomonas intermedia K12 | pTINT01 | 2.00E-35 | 95.05 | 99 | 80 |
| NC_014155.1 | thiomonas intermedia K12 | pTINT02 | 7.00E-38 | 98.95 | 93 | 40 |
| NC_017958.1 | Tistrella mobilis KA081020-065 | pTM3 | 1.00E-28 | 95 | 90 | 1442 |
| NC_017959.1 | Tistrella mobilis KA081020-065 | pTM4 | 9.00E-31 | 95 | 90 | 247 |
| NC_017966.1 | Tistrella mobilis KA081020-065 | pTM2 | 7.00E-30 | 95.05 | 90 | 16 |
| NC_005206.1 | Trueperella pyogenes | pAP2 | 1.00E-37 | 100 | 91 | 12 |
| NC_014159.1 | Tsukamurella paurometabola DSM 20162 | pTpau01 | 1.00E-36 | 96.04 | 101 | 2 |
| NC_008330.1 | Uncultured bacterium | pLB1 | 8.00E-37 | 96.04 | 91 | 10 |
| NC_003122.1 | Uncultured bacterium | pSB102 | 4.00E-40 | 99 | 99 | 14 |
| NC_003430.1 | Uncultured bacterium | pB4 | 1.00E-34 | 95 | 90 | 40 |
| NC_013278.1 | Uncultured bacterium | pTRACA18 | 4.00E-34 | 95.05 | 93 | 114 |
| NC_013279.1 | Uncultured bacterium | pTRACA20 | 2.00E-36 | 95.05 | 99 | 14 |
| NC_013280.1 | Uncultured bacterium | pTRACA22 | 1.00E-29 | 95 | 90 | 2498 |
| NC_013281.1 | Uncultured bacterium | pTRACA30 | 1.00E-32 | 95.92 | 90 | 30 |
| NC_004840.1 | Uncultured bacterium | pB10 | 5.00E-33 | 95.05 | 92 | 16 |
| NC_019020.1 | Uncultured bacterium | PB5 | 2.00E-37 | 99.01 | 91 | 18 |
| NC_019021.1 | Uncultured bacterium | PSP21 | 3.00E-36 | 96.97 | 90 | 120 |
| NC_019022.1 | Uncultured bacterium | PB11 | 3.00E-35 | 95.05 | 93 | 10 |
| NC_019216.1 | Uncultured bacterium | pRSB105 | 4.00E-33 | 95.74 | 92 | 60 |
| NC_006388.1 | Uncultured bacterium | pB3 | 3.00E-40 | 98.99 | 101 | 14 |
| NC_007680.1 | Uncultured bacterium | pTP6 | 9.00E-35 | 95 | 100 | 14 |
| NC_006352.1 | Uncultured bacterium activated sludge | pTB11 | 3.00E-35 | 96.88 | 96 | 58 |
| NC_006385.1 | Uncultured bacterium activated sludge | pRSB101 | 2.00E-35 | 95.05 | 90 | 90 |
| NC_019299.1 | Uncultured bacterium HH1107 | pHH1107 | 3.00E-42 | 100 | 101 | 2 |
| NC_019217.1 | Uncultured bacterium HHV216 | pHHV216 | 4.00E-33 | 95.05 | 90 | 182 |
| NC_019218.1 | Uncultured bacterium HHV35 | pHHV35 | 2.00E-32 | 95.05 | 91 | 79 |
| NC_007502.1 | Uncultured bacterium IncP-1beta multiresistance | pB8 | 7.00E-37 | 96.04 | 94 | 32 |
| NC_008055.1 | Uncultured bacterium IncP-1gamma | QKH54 | 3.00E-35 | 95.05 | 96 | 29 |
| NC_019324.1 | UNVERIFIED: Clostridium sp. MT351 | unnamed | 4.00E-32 | 95 | 90 | 44 |
| NC_019352.1 | UNVERIFIED: Leuconostoc mesenteroides subsp. mesenteroides | pMBLT00 | 1.00E-35 | 95.05 | 101 | 28 |
| NC_019320.1 | Variovorax sp. DB1 | pDB1 | 2.00E-38 | 100 | 94 | 36 |
| NC_008771.1 | Verminephrobacter eiseniae EF01-2 | pVEIS01 | 2.00E-35 | 95.05 | 91 | 12 |
| NC_023291.1 | Vibrio cholerae strain BI144 | pVCR94deltaX | 3.00E-39 | 98.02 | 101 | 8 |
| NC_020451.1 | Vibrio coralliilyticus strain ATCC BAA-450 |  | 3.00E-31 | 95 | 90 | 42 |
| NC_015756.1 | Weissella koreensis KACC 15510 | WKp2903 | 1.00E-34 | 95.05 | 93 | 22 |
| NC_009717.1 | Xanthobacter autotrophicus Py2 | pXAUT01 | 5.00E-34 | 95 | 90 | 288 |
| NC_016053.1 | Xanthomonas arboricola pv. pruni str. CFBP 5530 | pXap41 | 2.00E-35 | 95.05 | 99 | 5 |
| NC_003922.1 | Xanthomonas axonopodis pv. citri str. 306 | pXAC64 | 3.00E-35 | 95.05 | 101 | 2 |
| NC_020801.1 | Xanthomonas axonopodis Xac29-1 | pXAC33 | 7.00E-40 | 98.02 | 101 | 8 |
| NC_022539.1 | Xanthomonas fuscans subsp. fuscans str. 4834-R | pla | 2.00E-35 | 95.05 | 101 | 2 |
| NC_010377.1 | Yersinia enterocolitica | pYE854 | 1.00E-36 | 96.04 | 100 | 16 |
| NC_012208.1 | Yersinia enterocolitica | pYe4449-1 | 2.00E-36 | 95.05 | 100 | 8 |
| NC_012209.1 | Yersinia enterocolitica | pYe4449-2 | 1.00E-38 | 97.03 | 101 | 13 |
| NC_005570.1 | Yersinia enterocolitica 29807 | p29807 | 5.00E-42 | 99.01 | 101 | 2 |
| NC_019269.1 | Yersinia frederiksenii | pYF27601 | 2.00E-37 | 98.02 | 90 | 105 |
| NC_006323.1 | Yersinia pestis | pG8786 | 2.00E-41 | 100 | 100 | 2 |
| NC_017170.1 | Yersinia pestis A1122 | unnamed | 5.00E-32 | 95.56 | 101 | 4 |
| NC_005814.1 | Yersinia pestis biovar Microtus str. 91001 | pCRY | 9.00E-37 | 96 | 100 | 42 |
| NC_005815.1 | Yersinia pestis biovar Microtus str. 91001 | pMT1 | 8.00E-39 | 98 | 97 | 2 |
| NC_004835.1 | Yersinia pestis strain KIM5 | pMT1 | 2.00E-39 | 98.02 | 101 | 3 |
| NC_004837.1 | Yersinia pestis strain KIM5 | pPCP1 | 1.00E-31 | 95.05 | 90 | 14 |
| NC_011759.1 | Yersinia pseudotuberculosis | pGDT4 | 1.00E-36 | 96.97 | 99 | 2 |
| NC_009139.1 | Yersinia ruckeri YR71 | pYR1 | 3.00E-39 | 98.02 | 95 | 2 |
| NC_013356.1 | Zymomonas mobilis subsp. mobilis NCIB 11163 | pZA1001 | 5.00E-38 | 97.03 | 101 | 79 |
| NC_022903.1 | Zymomonas mobilis subsp. mobilis str. CP4 = NRRL B-14023 |  | 2.00E-35 | 95.05 | 96 | 10 |
| NC_015716.1 | Zymomonas mobilis subsp. pomaceae ATCC 29192 | pZYMOP02 | 2.00E-35 | 95.05 | 99 | 12 |

**Assigned contigs of sample C1756 against the RefSeq plasmid database**

(Sorted by Organism)

| **Accession number** | **Organism** | **Plasmid** | **E value ≤** | **Identity (%) ≥** | **Hit length (bp) ≥** | **Number of reads** |
| --- | --- | --- | --- | --- | --- | --- |
| NC_022242.1 | Achromobacter xylosoxidans subsp. denitrificans | pAX22 | < 1.0E-150 | 98.98 | 393 | 1 |
| NC_010605.1 | Acinetobacter baumannii ACICU | pACICU1 | 1.0E-149 | 96.33 | 327 | 1 |
| NC_009084.1 | Acinetobacter baumannii ATCC 17978 | pAB2 | < 1.0E-150 | 98.1 | 316 | 1 |
| NC_010404.1 | Acinetobacter baumannii AYE | p3ABAYE | 2.0E-31 | 95.56 | 90 | 16 |
| NC_021734.1 | Acinetobacter baumannii BJAB0715 | pBJAB0715 | 3.0E-102 | 95.12 | 245 | 6 |
| NC_017848.1 | Acinetobacter baumannii MDR-TJ | pABTJ1 | < 1.0E-150 | 100 | 1002 | 1 |
| NC_020524.1 | Acinetobacter baumannii MDR-TJ | pABTJ2 | < 1.0E-150 | 97.73 | 352 | 1 |
| NC_010481.1 | Acinetobacter baumannii | pABIR | 1.0E-35 | 96.15 | 92 | 9 |
| NC_013277.1 | Acinetobacter baumannii | pMMA2 | < 1.0E-150 | 96.68 | 662 | 1 |
| NC_013506.1 | Acinetobacter baumannii | pMMCU2 | < 1.0E-150 | 98.32 | 416 | 1 |
| NC_019280.1 | Acinetobacter baumannii | pMMD | < 1.0E-150 | 98.49 | 1193 | 1 |
| NC_019311.1 | Acinetobacter baumannii | pRAY*-v1 | < 1.0E-150 | 96.56 | 349 | 1 |
| NC_019345.1 | Acinetobacter baumannii | pRAY*-v2 | 1.0E-42 | 96.33 | 109 | 4 |
| NC_020818.1 | Acinetobacter baumannii strain GF216 | pNDM-AB | 2.0E-137 | 99.64 | 278 | 3 |
| NC_017166.1 | Acinetobacter baumannii TCDC-AB0715 | p2ABTCDC0715 | < 1.0E-150 | 99.45 | 1089 | 1 |
| NC_023031.1 | Acinetobacter baumannii ZW85-1 | ZW85p2 | 1.0E-41 | 100 | 99 | 1 |
| NC_010309.1 | Acinetobacter venetianus strain VE-C3 | pAV1 | < 1.0E-150 | 97.41 | 580 | 2 |
| NC_010310.1 | Acinetobacter venetianus strain VE-C3 | pAV2 | < 1.0E-150 | 99.15 | 1291 | 1 |
| NC_006143.1 | Aeromonas caviae | pFBAOT6 | 5.0E-96 | 95.73 | 202 | 25 |
| NC_003124.1 | Aeromonas salmonicida | pRAS3.2 | < 1.0E-150 | 96.84 | 368 | 2 |
| NC_009349.1 | Aeromonas salmonicida subsp. salmonicida A449 | 4 | 2.0E-41 | 98.25 | 102 | 4 |
| NC_014908.1 | Alicycliphilus denitrificans BC | pALIDE01 | 5.0E-104 | 98.65 | 222 | 1 |
| NC_013164.1 | Anaerococcus prevotii DSM 20548 | pAPRE01 | < 1.0E-150 | 98.86 | 351 | 1 |
| NC_008712.1 | Arthrobacter aurescens TC1 | TC1 | < 1.0E-150 | 99.74 | 761 | 2 |
| NC_017139.1 | Bacillus megaterium WSH-002 | WSH-002_p1 | 2.0E-36 | 96 | 100 | 1 |
| NC_014557.1 | Bacillus sp. BS-02 | pBS-02 | < 1.0E-150 | 99.84 | 1227 | 1 |
| NC_014937.1 | Bacillus thuringiensis CT43 | pBMB0558 | 3.0E-120 | 95.34 | 265 | 9 |
| NC_005026.1 | Bacteroides fragilis IB143 | pBI143 | 1.0E-149 | 95.35 | 312 | 4 |
| NC_006873.1 | Bacteroides fragilis NCTC 9343 | pBF9343 | 2.0E-134 | 100 | 269 | 1 |
| NC_011073.1 | Bacteroides fragilis | pBFP35 | 7.0E-106 | 99.33 | 215 | 7 |
| NC_019534.1 | Bacteroides fragilis | pBFUK1 | 2.0E-65 | 98.66 | 144 | 4 |
| NC_007068.1 | Bifidobacterium catenulatum | pBC1 | 5.0E-66 | 98.67 | 150 | 3 |
| NC_021875.1 | Bifidobacterium kashiwanohense JCM 15439 | pBBKW-1 DNA | < 1.0E-150 | 100 | 344 | 1 |
| NC_021876.1 | Bifidobacterium kashiwanohense JCM 15439 | pBBKW-2 DNA | 2.0E-64 | 95.89 | 146 | 3 |
| NC_004252.1 | Bifidobacterium longum DJO10A | pDOJH10L | < 1.0E-150 | 98.66 | 2021 | 1 |
| NC_004253.1 | Bifidobacterium longum DJO10A | pDOJH10S | < 1.0E-150 | 97.83 | 414 | 3 |
| NC_002635.1 | Bifidobacterium longum KJ | pKJ36 | 8.0E-103 | 99.07 | 216 | 3 |
| NC_010861.1 | Bifidobacterium longum | p6043B | 7.0E-72 | 98.95 | 155 | 4 |
| NC_011139.1 | Bifidobacterium longum | pFI2576 | < 1.0E-150 | 99.76 | 420 | 3 |
| NC_006997.1 | Bifidobacterium longum | pMG1 | < 1.0E-150 | 100 | 530 | 1 |
| NC_004769.1 | Bifidobacterium longum RW041 | PNAC2 | < 1.0E-150 | 99.09 | 329 | 1 |
| NC_004768.1 | Bifidobacterium longum RW041 | pNAC3 | 8.0E-45 | 96.01 | 103 | 8 |
| NC_004770.1 | Bifidobacterium longum RW048 | pNAC1 | 3.0E-101 | 96.52 | 230 | 6 |
| NC_015053.1 | Bifidobacterium longum subsp. infantis 157F | p157F-NC1 | < 1.0E-150 | 99.89 | 4419 | 1 |
| NC_015066.1 | Bifidobacterium longum subsp. infantis 157F | p157F-NC2 | < 1.0E-150 | 99.78 | 445 | 1 |
| NC_017222.1 | Bifidobacterium longum subsp. longum KACC 91563 | BLNIAS_P2 | < 1.0E-150 | 96.63 | 1099 | 1 |
| NC_004443.1 | Bifidobacterium longum VMKB44 | pB44 | 4.0E-126 | 99.77 | 252 | 2 |
| NC_019378.1 | Burkholderia cepacia | pIJB1 | < 1.0E-150 | 99.82 | 1117 | 1 |
| NC_009227.1 | Burkholderia vietnamiensis G4 | pBVIE02 | < 1.0E-150 | 100 | 608 | 1 |
| NC_022355.1 | Campylobacter coli CVM N29710 | pN29710-1 | 4.0E-83 | 98.36 | 183 | 3 |
| NC_006134.1 | Campylobacter coli | pCC31 | 4.0E-62 | 96.4 | 144 | 2 |
| NC_008790.1 | Campylobacter jejuni subsp. jejuni 81-176 | pTet | 5.0E-87 | 95.74 | 182 | 6 |
| NC_014801.1 | Campylobacter jejuni subsp. jejuni ICDCCJ07001 | pTet | < 1.0E-150 | 97.63 | 377 | 3 |
| NC_022601.1 | Carnobacterium sp. WN1359 | pWNCR12 | 7.0E-47 | 96.58 | 117 | 2 |
| NC_022602.1 | Carnobacterium sp. WN1359 | pWNCR47 | < 1.0E-150 | 95.46 | 463 | 1 |
| NC_022603.1 | Carnobacterium sp. WN1359 | pWNCR64 | 1.0E-90 | 98.97 | 194 | 1 |
| NC_020123.1 | Citrobacter freundii strain CFSTE | pT-OXA-181 | < 1.0E-150 | 100 | 624 | 1 |
| NC_010935.1 | Comamonas testosteroni CNB-1 | pCNB | < 1.0E-150 | 99.91 | 1079 | 1 |
| NC_002143.1 | Comamonas testosteroni PtL5 cryptic | pPT1 | < 1.0E-150 | 99.03 | 412 | 1 |
| NC_004833.1 | Corynebacterium renale | pCR1 | 2.0E-99 | 99.51 | 205 | 1 |
| NC_014167.1 | Corynebacterium resistens DSM 45100 | pJA144188 | 1.0E-111 | 96.03 | 252 | 3 |
| NC_009779.1 | Cronobacter sakazakii ATCC BAA-894 | pESA2 | 1.0E-53 | 97.59 | 120 | 5 |
| NC_023025.1 | Cronobacter sakazakii CMCC 45402 | p2 | < 1.0E-150 | 98.49 | 522 | 3 |
| NC_007974.2 | Cupriavidus metallidurans CH34 |  | 6.0E-81 | 97.67 | 177 | 2 |
| NC_007972.2 | Cupriavidus metallidurans CH34 | pMOL28 | < 1.0E-150 | 99.75 | 407 | 1 |
| NC_005088.1 | Delftia acidovorans B | pUO1 | < 1.0E-150 | 99.81 | 2679 | 1 |
| NC_019312.1 | Delftia sp. KV29 | pKV29 | 7.0E-80 | 95.86 | 172 | 11 |
| NC_009957.1 | Dinoroseobacter shibae DFL 12 | pDSHI03 | < 1.0E-150 | 98.74 | 449 | 2 |
| NC_001735.4 | Enterobacter aerogenes | R751 | 4.0E-139 | 95.08 | 278 | 3 |
| NC_012555.1 | Enterobacter cloacae | pEC-IMP | < 1.0E-150 | 99.82 | 1673 | 1 |
| NC_015175.1 | Enterobacter cloacae | pS51A | 2.0E-100 | 95.65 | 230 | 2 |
| NC_014107.1 | Enterobacter cloacae subsp. cloacae ATCC 13047 | pECL_A | < 1.0E-150 | 95.6 | 595 | 2 |
| NC_013514.1 | Enterococcus faecalis | pAMbeta1 | 2.0E-63 | 98.6 | 143 | 2 |
| NC_013533.1 | Enterococcus faecalis | pBEE99 | < 1.0E-150 | 98.14 | 321 | 2 |
| NC_006827.2 | Enterococcus faecalis | pCF10 | 3.0E-123 | 96.1 | 249 | 18 |
| NC_014508.2 | Enterococcus faecalis | pEF-01 | 8.0E-51 | 99.15 | 118 | 3 |
| NC_014726.1 | Enterococcus faecalis | pTW9 | 9.0E-55 | 100 | 123 | 1 |
| NC_014475.1 | Enterococcus faecalis | pWZ1668 | < 1.0E-150 | 97.71 | 409 | 6 |
| NC_008445.1 | Enterococcus faecalis RE25 | pRE25 | < 1.0E-150 | 95.05 | 316 | 8 |
| NC_004669.1 | Enterococcus faecalis V583 | pTEF1 | < 1.0E-150 | 98.79 | 331 | 1 |
| NC_021987.1 | Enterococcus faecium Aus0085 | p1 | 3.0E-145 | 98.99 | 298 | 2 |
| NC_017961.1 | Enterococcus faecium DO | 1 | 2.0E-123 | 100 | 249 | 1 |
| NC_017962.1 | Enterococcus faecium DO | 2 | 2.0E-31 | 96.67 | 90 | 2 |
| NC_017963.1 | Enterococcus faecium DO | 3 | 4.0E-46 | 95.16 | 124 | 9 |
| NC_020208.1 | Enterococcus faecium NRRL B-2354 | pNB2354_1 | 3.0E-47 | 95.93 | 123 | 14 |
| NC_013317.1 | Enterococcus faecium | p5753cA | < 1.0E-150 | 99.89 | 952 | 1 |
| NC_021170.1 | Enterococcus faecium | pF856 | < 1.0E-150 | 96.97 | 2246 | 1 |
| NC_006427.1 | Enterococcus faecium | pJB01 | 5.0E-46 | 98.17 | 109 | 1 |
| NC_011364.1 | Enterococcus faecium | pMG1 | < 1.0E-150 | 99.82 | 559 | 1 |
| NC_014959.1 | Enterococcus faecium | pS177 | < 1.0E-150 | 99.87 | 1571 | 1 |
| NC_008768.1 | Enterococcus faecium | pVEF1 | 1.0E-145 | 97.42 | 310 | 3 |
| NC_010980.1 | Enterococcus faecium | pVEF3 | < 1.0E-150 | 99.44 | 356 | 2 |
| NC_017627.1 | Escherichia coli 042 | pAA | < 1.0E-150 | 95.75 | 706 | 1 |
| NC_010558.1 | Escherichia coli 1520 | pIP1206 | 1.0E-57 | 98.3 | 136 | 7 |
| NC_010719.1 | Escherichia coli 53638 | p53638_226 | 5.0E-139 | 98.96 | 289 | 1 |
| NC_011752.1 | Escherichia coli 55989 | 55989p | < 1.0E-150 | 97.24 | 408 | 3 |
| NC_007675.1 | Escherichia coli A2363 | pAPEC-O2-ColV | 6.0E-80 | 95.79 | 190 | 4 |
| NC_006671.1 | Escherichia coli A2363 | pAPEC-O2-R | < 1.0E-150 | 97.84 | 339 | 2 |
| NC_023323.1 | Escherichia coli ACN001 | pACN001-A | < 1.0E-150 | 97.39 | 410 | 2 |
| NC_023327.1 | Escherichia coli ACN001 | pACN001-B | 6.0E-80 | 95.16 | 190 | 3 |
| NC_009837.1 | Escherichia coli APEC O1 | pAPEC-O1-ColBM | 1.0E-69 | 95.11 | 161 | 17 |
| NC_009838.1 | Escherichia coli APEC O1 | pAPEC-O1-R | 5.0E-140 | 99.3 | 286 | 4 |
| NC_002142.1 | Escherichia coli B171 | pB171 | < 1.0E-150 | 96.13 | 336 | 3 |
| NC_011980.1 | Escherichia coli chi7122 | pAPEC-1 | 3.0E-100 | 96.06 | 207 | 9 |
| NC_009788.1 | Escherichia coli E24377A | pETEC_73 | < 1.0E-150 | 99.61 | 313 | 2 |
| NC_009786.1 | Escherichia coli E24377A | pETEC_80 | 6.0E-44 | 98.15 | 108 | 4 |
| NC_013507.1 | Escherichia coli ETEC H10407 | pEntH10407 | < 1.0E-150 | 98.5 | 601 | 1 |
| NC_022649.1 | Escherichia coli JJ1886 | pJJ1886_2 | 0.0E+00 | 97.68 | 689 | 1 |
| NC_022662.1 | Escherichia coli JJ1886 | pJJ1886_3 | < 1.0E-150 | 100 | 378 | 1 |
| NC_002483.1 | Escherichia coli K-12 | F DNA | < 1.0E-150 | 96.82 | 363 | 3 |
| NC_002525.1 | Escherichia coli K-12 | R721 | < 1.0E-150 | 95.77 | 356 | 3 |
| NC_016904.1 | Escherichia coli KO11FL | pEKO1101 | < 1.0E-150 | 99.41 | 340 | 1 |
| NC_018651.1 | Escherichia coli O104:H4 str. 2009EL-2050 | p09EL50 | < 1.0E-150 | 96.13 | 362 | 1 |
| NC_013365.1 | Escherichia coli O111:H- str. 11128 | pO111_1 | < 1.0E-150 | 97.15 | 1178 | 2 |
| NC_013370.1 | Escherichia coli O111:H- str. 11128 | pO111_2 | < 1.0E-150 | 98.96 | 385 | 1 |
| NC_013366.1 | Escherichia coli O111:H- str. 11128 | pO111_3 | < 1.0E-150 | 99.7 | 331 | 1 |
| NC_002128.1 | Escherichia coli O157:H7 str. Sakai | pO157 | < 1.0E-150 | 100 | 316 | 1 |
| NC_020271.1 | Escherichia coli O25b:H4-ST131 str. EC958 strain ST131 | pJIE186-2 | 1.0E-143 | 98.65 | 297 | 1 |
| NC_013362.1 | Escherichia coli O26:H11 str. 11368 | pO26_2 | < 1.0E-150 | 99.65 | 573 | 1 |
| NC_017653.1 | Escherichia coli O55:H7 str. RM12579 | p12579_1 | < 1.0E-150 | 97.86 | 312 | 5 |
| NC_017648.1 | Escherichia coli O7:K1 str. CE10 | pCE10B | < 1.0E-150 | 98.04 | 459 | 1 |
| NC_009133.1 | Escherichia coli | NR1 | < 1.0E-150 | 99.78 | 379 | 3 |
| NC_015872.1 | Escherichia coli | p271A | < 1.0E-150 | 100 | 584 | 1 |
| NC_019062.1 | Escherichia coli | p838C-R1 | < 1.0E-150 | 99.38 | 483 | 1 |
| NC_008444.1 | Escherichia coli | p9705 | < 1.0E-150 | 99.77 | 427 | 1 |
| NC_011964.1 | Escherichia coli | pAPEC-O103-ColBM | 5.0E-143 | 98.65 | 296 | 3 |
| NC_012692.1 | Escherichia coli | pAR060302 | < 1.0E-150 | 96.9 | 387 | 1 |
| NC_019091.1 | Escherichia coli | pASL01a | < 1.0E-150 | 100 | 1047 | 1 |
| NC_019039.1 | Escherichia coli | pChi7122-3 | < 1.0E-150 | 96.73 | 1253 | 1 |
| NC_019049.1 | Escherichia coli | pCM959 | < 1.0E-150 | 98.01 | 429 | 4 |
| NC_022333.1 | Escherichia coli | pCss165Kan DNA | < 1.0E-150 | 99.83 | 587 | 1 |
| NC_014477.1 | Escherichia coli | pCT | < 1.0E-150 | 100 | 332 | 1 |
| NC_014383.1 | Escherichia coli | pEC_Bactec | < 1.0E-150 | 98.49 | 332 | 1 |
| NC_013120.1 | Escherichia coli | pEK204 | 1.0E-148 | 99.34 | 301 | 2 |
| NC_013122.1 | Escherichia coli | pEK499 | 9.0E-95 | 96.29 | 212 | 11 |
| NC_013121.1 | Escherichia coli | pEK516 | < 1.0E-150 | 100 | 349 | 1 |
| NC_019089.1 | Escherichia coli | pGUE-NDM | 1.0E-148 | 99.34 | 301 | 1 |
| NC_019075.1 | Escherichia coli | pKST23 | < 1.0E-150 | 95.38 | 692 | 1 |
| NC_009131.1 | Escherichia coli | pLEW517 | < 1.0E-150 | 97.49 | 319 | 4 |
| NC_010862.1 | Escherichia coli | pMAR7 | < 1.0E-150 | 99.44 | 355 | 2 |
| NC_013503.1 | Escherichia coli | pMAS2027 | < 1.0E-150 | 99.58 | 471 | 1 |
| NC_008489.1 | Escherichia coli | pMG828-4 | < 1.0E-150 | 97.99 | 398 | 2 |
| NC_007682.3 | Escherichia coli | pMUR050 | < 1.0E-150 | 100 | 694 | 1 |
| NC_018994.1 | Escherichia coli | pNDM-1_Dok01 | < 1.0E-150 | 97.13 | 1081 | 1 |
| NC_019063.1 | Escherichia coli | pNDM-HK | < 1.0E-150 | 99.48 | 388 | 1 |
| NC_022992.1 | Escherichia coli | pO111-CRL-115 | < 1.0E-150 | 99.22 | 337 | 2 |
| NC_012487.1 | Escherichia coli | pO26-Vir | 2.0E-73 | 97.71 | 161 | 4 |
| NC_008460.1 | Escherichia coli | pO86A1 | 4.0E-87 | 95.42 | 196 | 4 |
| NC_010378.1 | Escherichia coli | pOLA52 | 7.0E-48 | 97.39 | 113 | 7 |
| NC_019061.1 | Escherichia coli | pPWD4_103 | < 1.0E-150 | 99.89 | 891 | 1 |
| NC_009602.1 | Escherichia coli | pSFO157 | 4.0E-49 | 98.99 | 113 | 8 |
| NC_010409.1 | Escherichia coli | pVM01 | < 1.0E-150 | 97.24 | 332 | 4 |
| NC_011419.1 | Escherichia coli SE11 | pSE11-1 | 5.0E-40 | 97.12 | 104 | 3 |
| NC_011413.1 | Escherichia coli SE11 | pSE11-2 | < 1.0E-150 | 96.9 | 355 | 1 |
| NC_010488.1 | Escherichia coli SMS-3-5 | pSMS35_130 | 0.0E+00 | 99.32 | 438 | 1 |
| NC_023315.1 | Escherichia coli strain EQ011 | pEQ011 | 2.0E-83 | 95.36 | 196 | 4 |
| NC_011749.1 | Escherichia coli UMN026 | p1ESCUM | < 1.0E-150 | 97.54 | 325 | 1 |
| NC_017639.1 | Escherichia coli UMNK88 | pUMNK88_K88 | < 1.0E-150 | 96.66 | 329 | 1 |
| NC_012944.1 | Escherichia coli Vir68 | pVir68 | 7.0E-78 | 95.34 | 193 | 4 |
| NC_009716.1 | Escherichia sp. Sflu5 cryptic | pAK51 | < 1.0E-150 | 95.77 | 331 | 1 |
| NC_012782.1 | Eubacterium eligens ATCC 27750 | unnamed | 5.0E-120 | 95.45 | 248 | 53 |
| NC_012780.1 | Eubacterium eligens ATCC 27750 | unnamed | 3.0E-33 | 95.27 | 90 | 193 |
| NC_023287.1 | Exiguobacterium sp. S3-2 | pMC1 | 4.0E-33 | 95.06 | 93 | 6 |
| NC_012661.1 | Haemophilus parasuis | pHN61 | < 1.0E-150 | 99.6 | 1734 | 1 |
| NC_008153.1 | Human gut | pTRACA17 | 8.0E-51 | 97.54 | 122 | 1 |
| NC_014621.1 | Ketogulonicigenium vulgare Y25 | pYP1 | 4.0E-34 | 95.35 | 94 | 3 |
| NC_018107.1 | Klebsiella oxytoca E718 | pKOX_R1 | < 1.0E-150 | 99.55 | 396 | 3 |
| NC_011282.1 | Klebsiella pneumoniae 342 | pKP187 | 1.0E-146 | 97.15 | 316 | 1 |
| NC_011281.1 | Klebsiella pneumoniae 342 | pKP91 | 2.0E-76 | 97.69 | 168 | 3 |
| NC_005015.1 | Klebsiella pneumoniae BM4493 | pIP843 | 3.0E-55 | 95.74 | 141 | 1 |
| NC_017541.1 | Klebsiella pneumoniae KCTC 2242 | pKCTC2242 | 1.0E-39 | 96.15 | 106 | 4 |
| NC_011385.1 | Klebsiella pneumoniae | 12 | 4.0E-141 | 97.98 | 297 | 3 |
| NC_015154.1 | Klebsiella pneumoniae | pc15-k | 3.0E-68 | 95.78 | 166 | 5 |
| NC_003486.1 | Klebsiella pneumoniae | pJHCMW1 | < 1.0E-150 | 97.78 | 315 | 1 |
| NC_020087.1 | Klebsiella pneumoniae strain K1HV | pK1HV | 5.0E-111 | 99.57 | 230 | 1 |
| NC_010886.1 | Klebsiella pneumoniae | pK245 | 4.0E-85 | 98.22 | 184 | 9 |
| NC_010870.1 | Klebsiella pneumoniae | pK29 | 1.0E-135 | 96.59 | 276 | 2 |
| NC_019389.1 | Klebsiella pneumoniae | pKDO1 | < 1.0E-150 | 99.51 | 511 | 2 |
| NC_013542.1 | Klebsiella pneumoniae | pKF3-70 | < 1.0E-150 | 99.44 | 321 | 2 |
| NC_013950.1 | Klebsiella pneumoniae | pKF3-94 | < 1.0E-150 | 96.79 | 314 | 8 |
| NC_021654.1 | Klebsiella pneumoniae | pKN-LS6 | < 1.0E-150 | 98.24 | 398 | 5 |
| NC_014312.1 | Klebsiella pneumoniae | pKP048 | 5.0E-143 | 95.02 | 286 | 9 |
| NC_019160.1 | Klebsiella pneumoniae | pKP3-A | < 1.0E-150 | 99.57 | 461 | 1 |
| NC_020893.1 | Klebsiella pneumoniae | pKPC-LK30 | < 1.0E-150 | 95.3 | 660 | 1 |
| NC_019390.1 | Klebsiella pneumoniae | pKPN_CZ | 4.0E-51 | 96.86 | 120 | 18 |
| NC_021502.1 | Klebsiella pneumoniae | pKPoxa-48N2 | < 1.0E-150 | 99.23 | 1955 | 1 |
| NC_014016.1 | Klebsiella pneumoniae | pKpQIL | < 1.0E-150 | 100 | 465 | 1 |
| NC_019155.1 | Klebsiella pneumoniae | pKpQIL-IT | 8.0E-127 | 96.91 | 256 | 12 |
| NC_019162.1 | Klebsiella pneumoniae | pNDM-HN380 | < 1.0E-150 | 99.64 | 554 | 1 |
| NC_014368.1 | Klebsiella pneumoniae | pNL194 | < 1.0E-150 | 100 | 336 | 1 |
| NC_014478.1 | Klebsiella pneumoniae | unnamed | 2.0E-69 | 95.29 | 170 | 2 |
| NC_019888.1 | Klebsiella pneumoniae strain BK31551 | pBK31551 | 5.0E-134 | 99.63 | 272 | 1 |
| NC_020132.1 | Klebsiella pneumoniae strain BK32179 | pBK32179 | 3.0E-51 | 95.45 | 132 | 5 |
| NC_021238.1 | Klebsiella pneumoniae strain Kpn-1433 | pKP1433 | < 1.0E-150 | 99.63 | 691 | 2 |
| NC_022609.1 | Klebsiella pneumoniae strain N11-0042 | pKp11-42 | < 1.0E-150 | 96.86 | 514 | 2 |
| NC_023333.1 | Klebsiella pneumoniae strain ST23 | pKP007 | 1.0E-65 | 98.96 | 147 | 2 |
| NC_016840.1 | Klebsiella pneumoniae subsp. pneumoniae HS11286 | pKPHS4 | < 1.0E-150 | 98.53 | 337 | 2 |
| NC_021198.1 | Klebsiella pneumoniae subsp. pneumoniae KPX | pKPX-1 DNA | < 1.0E-150 | 96.4 | 389 | 2 |
| NC_021199.1 | Klebsiella pneumoniae subsp. pneumoniae KPX | pKPX-2 DNA | 2.0E-58 | 97.87 | 133 | 3 |
| NC_009649.1 | Klebsiella pneumoniae subsp. pneumoniae MGH 78578 | pKPN3 | 5.0E-37 | 95.21 | 91 | 55 |
| NC_009650.1 | Klebsiella pneumoniae subsp. pneumoniae MGH 78578 | pKPN4 | < 1.0E-150 | 99.29 | 471 | 2 |
| NC_009651.1 | Klebsiella pneumoniae subsp. pneumoniae MGH 78578 | pKPN5 | 2.0E-121 | 100 | 246 | 4 |
| NC_009652.1 | Klebsiella pneumoniae subsp. pneumoniae MGH 78578 | pKPN6 | < 1.0E-150 | 98.12 | 320 | 1 |
| NC_015319.1 | Lactobacillus amylovorus GRL 1112 | 1 | < 1.0E-150 | 99.02 | 306 | 1 |
| NC_017471.1 | Lactobacillus amylovorus GRL1118 | 1 | 7.0E-124 | 97.01 | 268 | 1 |
| NC_017472.1 | Lactobacillus amylovorus GRL1118 | 2 | 9.0E-42 | 95.35 | 102 | 14 |
| NC_008499.1 | Lactobacillus brevis ATCC 367 | 2 | < 1.0E-150 | 100 | 427 | 1 |
| NC_005952.1 | Lactobacillus brevis | pRH45II | < 1.0E-150 | 99.71 | 696 | 1 |
| NC_018611.1 | Lactobacillus buchneri CD034 | pCD034-3 | < 1.0E-150 | 96.15 | 1325 | 1 |
| NC_015420.1 | Lactobacillus buchneri NRRL B-30929 | pLBUC01 | < 1.0E-150 | 99.48 | 762 | 1 |
| NC_015429.1 | Lactobacillus buchneri NRRL B-30929 | pLBUC02 | 6.0E-94 | 95.85 | 217 | 1 |
| NC_008502.1 | Lactobacillus casei ATCC 334 | 1 | 8.0E-139 | 99.64 | 280 | 2 |
| NC_011352.1 | Lactobacillus casei str. Zhang | plca36 | 3.0E-95 | 95.11 | 225 | 10 |
| NC_004947.1 | Lactobacillus fermentum | pKC5b | 4.0E-121 | 97.67 | 258 | 2 |
| NC_011839.1 | Lactobacillus gasseri | pLgLA39 | < 1.0E-150 | 97.2 | 357 | 2 |
| NC_017468.1 | Lactobacillus helveticus H10 | pH10 | 2.0E-121 | 95.18 | 265 | 3 |
| NC_015598.1 | Lactobacillus kefiranofaciens ZW3 | pWW1 | 2.0E-85 | 97.54 | 192 | 2 |
| NC_015603.1 | Lactobacillus kefiranofaciens ZW3 | pWW2 | 5.0E-81 | 96.77 | 186 | 3 |
| NC_022123.1 | Lactobacillus paracasei subsp. paracasei 8700:2 | 2 | < 1.0E-150 | 96.67 | 480 | 1 |
| NC_021526.1 | Lactobacillus plantarum 16 | Lp16D | 2.0E-39 | 95.41 | 109 | 2 |
| NC_021225.1 | Lactobacillus plantarum subsp. plantarum P-8 | LBPp2 | 6.0E-42 | 98.08 | 104 | 1 |
| NC_021226.1 | Lactobacillus plantarum subsp. plantarum P-8 | LBPp3 | < 1.0E-150 | 100 | 338 | 1 |
| NC_014558.2 | Lactobacillus plantarum subsp. plantarum ST-III | pST-III | 2.0E-111 | 98.13 | 230 | 2 |
| NC_021503.1 | Lactobacillus reuteri I5007 | pLRI01 | 5.0E-132 | 99.26 | 270 | 2 |
| NC_021495.1 | Lactobacillus reuteri I5007 | pLRI02 | 3.0E-77 | 98.25 | 171 | 3 |
| NC_021504.1 | Lactobacillus reuteri I5007 | pLRI04 | 8.0E-41 | 96.55 | 96 | 5 |
| NC_021497.1 | Lactobacillus reuteri I5007 | pLRI05 | < 1.0E-150 | 96.7 | 455 | 3 |
| NC_015701.1 | Lactobacillus reuteri SD2112 | pLR584 | < 1.0E-150 | 99.72 | 1818 | 1 |
| NC_010603.1 | Lactobacillus reuteri strain ATCC 55730 | pLR581 | < 1.0E-150 | 97.65 | 852 | 2 |
| NC_010621.1 | Lactobacillus reuteri strain ATCC 55730 | pLR585 | < 1.0E-150 | 99.91 | 1089 | 2 |
| NC_013200.1 | Lactobacillus rhamnosus Lc 705 | pLC1 | 8.0E-143 | 97.3 | 293 | 7 |
| NC_017479.1 | Lactobacillus salivarius CECT 5713 | pHN1 | 2.0E-123 | 95.94 | 277 | 19 |
| NC_017480.1 | Lactobacillus salivarius CECT 5713 | pHN2 | 2.0E-138 | 98.84 | 281 | 2 |
| NC_017499.1 | Lactobacillus salivarius CECT 5713 | pHN3 | < 1.0E-150 | 98.52 | 337 | 3 |
| NC_007930.1 | Lactobacillus salivarius UCC118 | pMP118 | < 1.0E-150 | 95.04 | 383 | 2 |
| NC_006529.1 | Lactobacillus salivarius UCC118 | pSF118-20 | 1.0E-128 | 98.87 | 266 | 1 |
| NC_006530.1 | Lactobacillus salivarius UCC118 | pSF118-44 | 4.0E-113 | 95.56 | 245 | 11 |
| NC_015979.1 | Lactobacillus sanfranciscensis TMW 1.1304 | pLS1 | 3.0E-52 | 98 | 121 | 4 |
| NC_016971.1 | Lactococcus garvieae 21881 | pGL4 | 7.0E-130 | 95.53 | 291 | 2 |
| NC_010540.1 | Lactococcus garvieae | pKL0018 DNA | 1.0E-127 | 100 | 256 | 1 |
| NC_007191.1 | Lactococcus lactis cremoris 712 | pAG6 | < 1.0E-150 | 99.93 | 1366 | 1 |
| NC_004847.1 | Lactococcus lactis cremoris HP | pHP003 | < 1.0E-150 | 98.28 | 522 | 1 |
| NC_002137.1 | Lactococcus lactis cremoris NIZO B40 | pNZ4000 | < 1.0E-150 | 97.42 | 339 | 7 |
| NC_001949.1 | Lactococcus lactis DPC3147 | pMRC01 | < 1.0E-150 | 96.5 | 314 | 6 |
| NC_004966.1 | Lactococcus lactis lactis bv. diacetylactis DPC220 | pAH82 | < 1.0E-150 | 97.25 | 910 | 2 |
| NC_009435.1 | Lactococcus lactis NCDO 1867 | pGdh442 | 6.0E-145 | 95.48 | 289 | 21 |
| NC_010901.1 | Lactococcus lactis | pNP40 | < 1.0E-150 | 98.71 | 541 | 1 |
| NC_017493.1 | Lactococcus lactis subsp. cremoris A76 | pQA549 | < 1.0E-150 | 97.07 | 341 | 1 |
| NC_017496.1 | Lactococcus lactis subsp. cremoris A76 | pQA554 | 2.0E-41 | 97.17 | 106 | 8 |
| NC_019377.1 | Lactococcus lactis subsp. cremoris | pLP712 | < 1.0E-150 | 99.29 | 469 | 2 |
| NC_008504.1 | Lactococcus lactis subsp. cremoris SK11 | 2 | < 1.0E-150 | 95.57 | 451 | 1 |
| NC_008505.1 | Lactococcus lactis subsp. cremoris SK11 | 3 | 1.0E-65 | 96.2 | 158 | 9 |
| NC_008506.1 | Lactococcus lactis subsp. cremoris SK11 | 4 | < 1.0E-150 | 97.47 | 321 | 9 |
| NC_019436.1 | Lactococcus lactis subsp. cremoris UC509.9 | pCIS6 | 2.0E-71 | 96.36 | 158 | 4 |
| NC_019430.1 | Lactococcus lactis subsp. cremoris UC509.9 | pCIS8 | < 1.0E-150 | 97.27 | 952 | 1 |
| NC_009137.1 | Lactococcus lactis subsp. lactis bv. diacetylactis | pDBORO | < 1.0E-150 | 98.49 | 332 | 1 |
| NC_015900.1 | Lactococcus lactis subsp. lactis bv. diacetylactis | pVF18 | 6.0E-145 | 96.58 | 306 | 7 |
| NC_015901.1 | Lactococcus lactis subsp. lactis bv. diacetylactis | pVF22 | 8.0E-119 | 97.67 | 257 | 5 |
| NC_015902.1 | Lactococcus lactis subsp. lactis bv. diacetylactis | pVF50 | 3.0E-148 | 98.66 | 295 | 6 |
| NC_017483.1 | Lactococcus lactis subsp. lactis CV56 | pCV56A | < 1.0E-150 | 95.58 | 339 | 1 |
| NC_017487.1 | Lactococcus lactis subsp. lactis CV56 | pCV56B | < 1.0E-150 | 95.9 | 374 | 4 |
| NC_017484.1 | Lactococcus lactis subsp. lactis CV56 | pCV56C | 6.0E-84 | 96.92 | 195 | 6 |
| NC_017488.1 | Lactococcus lactis subsp. lactis CV56 | pCV56E | 3.0E-48 | 95.87 | 121 | 1 |
| NC_009751.1 | Lactococcus lactis subsp. lactis K214 | pK214 | < 1.0E-150 | 97.85 | 327 | 3 |
| NC_013657.1 | Lactococcus lactis subsp. lactis KF147 | pKF147A | 2.0E-40 | 95.41 | 109 | 4 |
| NC_015860.1 | Lactococcus lactis subsp. lactis | pIL1 | < 1.0E-150 | 97.36 | 455 | 1 |
| NC_015862.1 | Lactococcus lactis subsp. lactis | pIL4 | < 1.0E-150 | 95 | 331 | 8 |
| NC_015863.1 | Lactococcus lactis subsp. lactis | pIL5 | < 1.0E-150 | 97.52 | 315 | 4 |
| NC_019308.1 | Lactococcus lactis subsp. lactis | pIL6 | < 1.0E-150 | 96.31 | 515 | 1 |
| NC_015864.1 | Lactococcus lactis subsp. lactis | pIL7 | < 1.0E-150 | 99.75 | 405 | 1 |
| NC_016042.1 | Lactococcus lactis subsp. lactis | pKP1 | 6.0E-138 | 96.77 | 295 | 3 |
| NC_010470.1 | Leuconostoc citreum KM20 | pLCK1 | < 1.0E-150 | 95.14 | 453 | 1 |
| NC_010466.1 | Leuconostoc citreum KM20 | pLCK2 | < 1.0E-150 | 97.84 | 417 | 1 |
| NC_010467.1 | Leuconostoc citreum KM20 | pLCK3 | < 1.0E-150 | 98.44 | 385 | 1 |
| NC_014134.1 | Leuconostoc kimchii IMSNU 11154 | LkipL4726 | 1.0E-136 | 98.99 | 273 | 3 |
| NC_008496.1 | Leuconostoc mesenteroides subsp. mesenteroides ATCC 8293 | pLEUM1 | 6.0E-34 | 95.74 | 94 | 3 |
| NC_013767.1 | Listeria monocytogenes 08-5578 | pLM5578 | 6.0E-57 | 98.88 | 130 | 2 |
| NC_011996.1 | Macrococcus caseolyticus JCSC5402 | pMCCL2 | < 1.0E-150 | 99.89 | 902 | 1 |
| NC_008242.1 | Mesorhizobium sp. BNC1 | 1 | 1.0E-156 | 95.97 | 347 | 2 |
| NC_012811.1 | Methylobacterium extorquens AM1 |  | 1.0E-149 | 95.82 | 300 | 7 |
| NC_004954.1 | Micrococcus sp. 28 | pSD10 | < 1.0E-150 | 99.86 | 1397 | 1 |
| NC_022599.1 | Micrococcus sp. V7 | pLMV7 | 8.0E-132 | 99.63 | 268 | 1 |
| NC_009669.1 | Ochrobactrum anthropi ATCC 49188 | pOANT01 | 2.0E-118 | 97.51 | 249 | 6 |
| NC_019554.1 | Oenococcus oeni | pOENI-1v2 | < 1.0E-150 | 99.12 | 454 | 1 |
| NC_014842.1 | Pantoea sp. At-9b | pPAT9B05 | 3.0E-50 | 95.38 | 130 | 1 |
| NC_022042.1 | Paracoccus aminophilus JCM 7686 | pAMI1 | 1.0E-131 | 99.63 | 268 | 1 |
| NC_022050.1 | Paracoccus aminophilus JCM 7686 | pAMI8 | 6.0E-74 | 96.05 | 177 | 2 |
| NC_013513.1 | Paracoccus aminophilus strain JCM 7686 | pAMI3 | < 1.0E-150 | 97.29 | 553 | 1 |
| NC_014832.1 | Paracoccus aminophilus strain JCM 7686 | pAMI7 | < 1.0E-150 | 97.64 | 459 | 4 |
| NC_009753.1 | Paracoccus methylutens strain DM12 | pMTH1 | < 1.0E-150 | 99.94 | 1614 | 1 |
| NC_010864.1 | Pediococcus acidilactici | pEOC01 | 9.0E-39 | 95.31 | 109 | 7 |
| NC_017019.1 | Pediococcus claussenii ATCC BAA-344 | pPECL-8 | 3.0E-113 | 97.33 | 242 | 2 |
| NC_008608.1 | Pelobacter propionicus DSM 2379 | pPRO2 | < 1.0E-150 | 98.75 | 469 | 4 |
| NC_023148.1 | Phaeobacter gallaeciensis DSM 26640 | pGal_B134 | < 1.0E-150 | 98.58 | 564 | 1 |
| NC_008613.1 | Photobacterium damselae subsp. piscicida | pP91278 | < 1.0E-150 | 100 | 355 | 3 |
| NC_009739.1 | Pseudomonas aeruginosa | pMATVIM-7 | 1.0E-116 | 95.65 | 251 | 3 |
| NC_022344.1 | Pseudomonas aeruginosa | pOZ176 | < 1.0E-150 | 95.96 | 317 | 2 |
| NC_020452.1 | Pseudomonas aeruginosa strain COL-1 | pNOR-2000 | 1.0E-104 | 98.21 | 224 | 1 |
| NC_003350.1 | Pseudomonas putida | pWW0 | < 1.0E-150 | 99.02 | 317 | 2 |
| NC_021158.1 | Psychrobacter maritimus strain MR29-12 | pKLH80 | < 1.0E-150 | 98.26 | 459 | 1 |
| NC_008382.1 | Rhizobium leguminosarum bv. viciae 3841 | pRL7 | 1.0E-113 | 96.83 | 252 | 1 |
| NC_022536.1 | Rhizobium sp. IRBG74 | IRBL74_p | 3.0E-122 | 95.7 | 279 | 1 |
|  |  |  | < 1.0E-150 |  |  |  |
| NC_009981.1 | Salmonella enterica subsp. enterica serovar Choleraesuis | pMAK1 | < 1.0E-150 | 99.57 | 465 | 4 |
| NC_010119.1 | Salmonella enterica subsp. enterica serovar Choleraesuis | pOU7519 | < 1.0E-150 | 99.48 | 384 | 1 |
| NC_021819.1 | Salmonella enterica subsp. enterica Serovar Cubana str. CFSAN002050 |  | 9.0E-142 | 99.65 | 851 | 1 |
| NC_009980.1 | Salmonella enterica subsp. enterica serovar Dublin | pMAK2 | 6.0E-133 | 99.65 | 286 | 1 |
| NC_010422.1 | Salmonella enterica subsp. enterica serovar Dublin | pOU1115 | < 1.0E-150 | 99.63 | 270 | 1 |
| NC_019131.1 | Salmonella enterica subsp. enterica serovar Heidelberg | pSH146_87 | < 1.0E-150 | 98.61 | 575 | 1 |
| NC_019117.1 | Salmonella enterica subsp. enterica serovar Heidelberg | pSH696_117 | < 1.0E-150 | 99.69 | 652 | 1 |
| NC_021869.1 | Salmonella enterica subsp. enterica serovar Heidelberg str. 41578 | pSEEH1578_03 | 2.0E-146 | 95.24 | 1702 | 1 |
| NC_011081.1 | Salmonella enterica subsp. enterica serovar Heidelberg str. SL476 | pSL476_91 | 1.0E-149 | 97.19 | 292 | 5 |
| NC_011077.1 | Salmonella enterica subsp. enterica serovar Kentucky str. CVM29188 | pCVM29188_101 | 4.0E-50 | 100 | 298 | 3 |
| NC_011076.1 | Salmonella enterica subsp. enterica serovar Kentucky str. CVM29188 | pCVM29188_146 | 1.0E-37 | 98.33 | 115 | 5 |
| NC_022522.1 | Salmonella enterica subsp. enterica serovar Kentucky strain 1643/10 | p1643_10 | 5.0E-144 | 95.24 | 105 | 1 |
| NC_011079.1 | Salmonella enterica subsp. enterica serovar Newport str. SL254 | pSL254_3 | 2.0E-42 | 95.38 | 325 | 1 |
| NC_009140.1 | Salmonella enterica subsp. enterica serovar Newport str. SL254 | pSN254 | < 1.0E-150 | 100 | 101 | 3 |
| NC_003384.1 | Salmonella enterica subsp. enterica serovar Typhi str. CT18 | pHCM1 | 2.0E-138 | 100 | 1121 | 1 |
| NC_015570.1 | Salmonella enterica subsp. enterica serovar Typhimurium | pSe-Kan | < 1.0E-150 | 100 | 275 | 1 |
| NC_006816.1 | Salmonella enterica subsp. enterica serovar Typhimurium | pU302L | < 1.0E-150 | 99.88 | 2509 | 1 |
| NC_003292.1 | Salmonella enterica subsp. enterica serovar Typhimurium | R46 | < 1.0E-150 | 100 | 307 | 2 |
| NC_017675.1 | Salmonella enterica subsp. enterica serovar Typhimurium str. ST4/74 | TY474p2 | 2.0E-144 | 97.5 | 400 | 2 |
| NC_021815.1 | Salmonella enterica subsp. enterica serovar Typhimurium var. 5- str. CFSAN001921 | unnamed | 5.0E-132 | 95.68 | 309 | 34 |
| NC_002305.1 | Salmonella typhi | R27 | 2.0E-84 | 97.44 | 269 | 4 |
| NC_021742.1 | Serratia liquefaciens ATCC 27592 |  | 2.0E-38 | 95.19 | 208 | 2 |
| NC_008573.1 | Shewanella sp. ANA-3 | 1 | < 1.0E-150 | 97.04 | 94 | 16 |
| NC_010660.1 | Shigella boydii CDC 3083-94 | pBS512_211 | 2.0E-41 | 96.17 | 339 | 1 |
| NC_007607.1 | Shigella dysenteriae Sd197 | pSD1_197 | 1.0E-44 | 99.02 | 102 | 1 |
| NC_004851.1 | Shigella flexneri 2a str. 301 | pCP301 | < 1.0E-150 | 100 | 105 | 1 |
| NC_002698.1 | Shigella flexneri 5a virulence | pWR501 | 9.0E-60 | 99.56 | 396 | 2 |
| NC_016833.1 | Shigella sonnei 53G | A | < 1.0E-150 | 96.58 | 146 | 1 |
| NC_019256.1 | Shigella sp. LN126 | pLN126_33 | 1.0E-132 | 95.58 | 1267 | 1 |
| NC_019254.1 | Shigella sp. MO17 | pMO17_54 | 2.0E-100 | 96.01 | 284 | 7 |
| NC_016836.1 | Sinorhizobium fredii HH103 | pSfHH103b | < 1.0E-150 | 98.6 | 215 | 1 |
| NT_187147.1 | Sinorhizobium fredii HH103 | pSfHH103d fragment 2 | < 1.0E-150 | 95.06 | 385 | 1 |
| NC_021209.1 | Sinorhizobium sp. M14 | pSinA | < 1.0E-150 | 95.02 | 326 | 3 |
| NC_013319.1 | Staphylococcus aureus | pI258 | < 1.0E-150 | 99.88 | 4004 | 1 |
| NC_002774.1 | Staphylococcus aureus subsp. aureus Mu50 isolate C3912 | VRSAp | < 1.0E-150 | 100 | 315 | 2 |
| NC_020183.2 | Staphylococcus aureus subsp. aureus ST398 | pUR3912 | 5.0E-57 | 97.62 | 336 | 1 |
| NC_002136.1 | Streptococcus agalactiae | pGB3634 | < 1.0E-150 | 95.17 | 145 | 2 |
| NC_016750.1 | Streptococcus macedonicus ACA-DC 198 | pSMA198 | 5.0E-79 | 98.12 | 319 | 2 |
| NC_000937.1 | Streptococcus thermophilus ST135 | pER35 | < 1.0E-150 | 98.28 | 174 | 3 |
| NC_014154.1 | Thiomonas intermedia K12 | pTINT01 | 2.0E-120 | 96.12 | 332 | 6 |
| NC_014155.1 | thiomonas intermedia K12 | pTINT02 | 2.0E-126 | 95.02 | 281 | 1 |
| NC_017958.1 | Tistrella mobilis KA081020-065 | pTM3 | < 1.0E-150 | 95.15 | 260 | 32 |
| NC_006385.1 | Uncultured bacterium activated sludge | pRSB101 | 2.0E-118 | 96.84 | 696 | 1 |
| NC_019217.1 | Uncultured bacterium HHV216 | pHHV216 | 6.0E-87 | 96.58 | 263 | 1 |
| NC_019218.1 | Uncultured bacterium HHV35 | pHHV35 | < 1.0E-150 | 100 | 182 | 2 |
| NC_008272.1 | Uncultured bacterium IncP-1 | pKJK5 | < 1.0E-150 | 97.59 | 623 | 1 |
| NC_008055.1 | Uncultured bacterium IncP-1gamma | QKH54 | < 1.0E-150 | 100 | 309 | 3 |
| NC_004840.1 | Uncultured bacterium | pB10 | < 1.0E-150 | 99.07 | 970 | 1 |
| NC_019022.1 | Uncultured bacterium | PB11 | 3.0E-136 | 100 | 441 | 1 |
| NC_019021.1 | Uncultured bacterium | PSP21 | < 1.0E-150 | 95.45 | 276 | 8 |
| NC_013278.1 | Uncultured bacterium | pTRACA18 | < 1.0E-150 | 97.43 | 583 | 1 |
| NC_013279.1 | Uncultured bacterium | pTRACA20 | 9.0E-61 | 97.89 | 380 | 1 |
| NC_013280.1 | Uncultured bacterium | pTRACA22 | 1.0E-144 | 95.09 | 145 | 15 |
| NC_013281.1 | Uncultured bacterium | pTRACA30 | < 1.0E-150 | 99.32 | 292 | 1 |
| NC_002524.1 | Uncultured eubacterium pIE1115 | pIE1115 | 1.0E-36 | 100 | 580 | 1 |
| NC_019324.1 | UNVERIFIED: Clostridium sp. MT351 | unnamed | < 1.0E-150 | 95.88 | 97 | 1 |
| NC_019320.1 | Variovorax sp. DB1 | pDB1 | < 1.0E-150 | 99.75 | 400 | 1 |
| NC_023291.1 | Vibrio cholerae strain BI144 | pVCR94deltaX | 1.0E-57 | 96.59 | 440 | 2 |
| NC_015756.1 | Weissella koreensis KACC 15510 | WKp2903 | < 1.0E-150 | 98.05 | 132 | 3 |
| NC_003922.1 | Xanthomonas axonopodis pv. citri str. 306 | pXAC64 | < 1.0E-150 | 98.68 | 379 | 1 |
| NC_010377.1 | Yersinia enterocolitica | pYE854 | < 1.0E-150 | 99.77 | 429 | 1 |
| NC_008120.1 | Yersinia pestis Antiqua | pMT | < 1.0E-150 | 96.91 | 940 | 1 |
| NC_009141.1 | Yersinia pestis biovar Orientalis str. IP275 | pIP1202 | < 1.0E-150 | 99.43 | 353 | 2 |
| NC_009139.1 | Yersinia ruckeri YR71 | pYR1 | < 1.0E-150 | 96.75 | 431 | 1 |

**Assigned high-throughput reads of sample C1757 against the RefSeq plasmid database**

(Sorted by Organism)

| **Accession number** | **Organism** | **Plasmid** | **E value ≤** | **Identity (%) ≥** | **Hit length (bp) ≥** | **Number of reads** |
| --- | --- | --- | --- | --- | --- | --- |
| NC_021992.1 | Acetobacter pasteurianus 386B | Apa386Bp3 | 1.0E-35 | 95 | 100 | 6 |
| NC_017114.1 | Acetobacter pasteurianus IFO 3283-12 | pAPA12-030 | 4.0E-41 | 99.01 | 101 | 2 |
| NC_005793.2 | Achromobacter denitrificans | pEST4011 | 9.0E-32 | 95 | 90 | 850 |
| NC_014641.1 | Achromobacter xylosoxidans A8 | pA81 | 6.0E-35 | 96.04 | 90 | 29 |
| NC_006830.1 | Achromobacter xylosoxidans A8 | pA81 | 4.0E-31 | 96.67 | 90 | 4 |
| NC_014642.1 | Achromobacter xylosoxidans A8 | pA82 | 4.0E-35 | 95.05 | 96 | 4 |
| NC_022242.1 | Achromobacter xylosoxidans subsp. Denitrificans | pAX22 | 3.0E-33 | 95 | 90 | 118 |
| NC_015852.1 | Acidithiobacillus caldus SM-1 | pLAtc1 | 7.0E-36 | 95 | 96 | 24 |
| NC_015854.1 | Acidithiobacillus caldus SM-1 | pLAtc3 | 6.0E-36 | 95.05 | 101 | 15 |
| NC_010600.1 | Acidithiobacillus caldus strain MNG | pTcM1 | 2.0E-32 | 95.05 | 90 | 20 |
| NC_005023.1 | Acidithiobacillus ferrooxidans | pTF5 | 4.0E-36 | 95.05 | 99 | 2 |
| NC_008765.1 | Acidovorax sp. JS42 | pAOVO01 | 1.0E-30 | 95.05 | 90 | 82 |
| NC_008766.1 | Acidovorax sp. JS42 | pAOVO02 | 2.0E-33 | 95.74 | 91 | 144 |
| NC_022565.1 | Acinetobacter baumannii 107m | p1ABIBUN | 3.0E-34 | 95 | 94 | 43 |
| NC_017163.1 | Acinetobacter baumannii 1656-2 | ABKp1 | 3.0E-31 | 95.05 | 90 | 688 |
| NC_017164.1 | Acinetobacter baumannii 1656-2 | ABKp2 | 5.0E-36 | 95 | 92 | 59 |
| NC_006877.1 | Acinetobacter baumannii 19606 | pMAC | 2.0E-35 | 95.05 | 93 | 65 |
| NC_010605.1 | Acinetobacter baumannii ACICU | pACICU1 | 3.0E-32 | 95 | 90 | 391 |
| NC_010606.1 | Acinetobacter baumannii ACICU | pACICU2 | 7.0E-39 | 97.98 | 99 | 1 |
| NC_009083.1 | Acinetobacter baumannii ATCC 17978 | pAB1 | 3.0E-36 | 95.05 | 97 | 15 |
| NC_009084.1 | Acinetobacter baumannii ATCC 17978 | pAB2 | 3.0E-35 | 95.05 | 99 | 46 |
| NC_010401.1 | Acinetobacter baumannii AYE | p1ABAYE | 4.0E-36 | 95.05 | 91 | 263 |
| NC_010402.1 | Acinetobacter baumannii AYE | p2ABAYE | 2.0E-36 | 97.03 | 91 | 104 |
| NC_010404.1 | Acinetobacter baumannii AYE | p3ABAYE | 4.0E-31 | 95 | 90 | 1662 |
| NC_021727.1 | Acinetobacter baumannii BJAB07104 | p1BJAB07104 | 7.0E-39 | 100 | 94 | 5 |
| NC_021728.1 | Acinetobacter baumannii BJAB07104 | p2BJAB07104 | 3.0E-38 | 97 | 100 | 11 |
| NC_021734.1 | Acinetobacter baumannii BJAB0715 | pBJAB0715 | 2.0E-32 | 95 | 90 | 1276 |
| NC_020525.1 | Acinetobacter baumannii D1279779 | pD1279779 | 2.0E-34 | 95 | 94 | 48 |
| NC_017848.1 | Acinetobacter baumannii MDR-TJ | pABTJ1 | 3.0E-32 | 95.05 | 90 | 162 |
| NC_020524.1 | Acinetobacter baumannii MDR-TJ | pABTJ2 | 4.0E-32 | 95.05 | 90 | 357 |
| NC_017172.1 | Acinetobacter baumannii MDR-ZJ06 | pMDR-ZJ06 | 4.0E-36 | 95.05 | 90 | 229 |
| NC_019359.1 | Acinetobacter baumannii | pAB120 | 8.0E-42 | 99.01 | 101 | 1 |
| NC_010481.1 | Acinetobacter baumannii | pABIR | 4.0E-32 | 95 | 90 | 276 |
| NC_021489.1 | Acinetobacter baumannii strain: NCGM 253 | pAB-NCGM253 | 6.0E-36 | 95.1 | 99 | 3 |
| NC_012813.1 | Acinetobacter baumannii | pABVA01 | 2.0E-36 | 95.05 | 98 | 22 |
| NC_013277.1 | Acinetobacter baumannii | pMMA2 | 3.0E-35 | 95 | 96 | 109 |
| NC_013506.1 | Acinetobacter baumannii | pMMCU2 | 2.0E-35 | 95.05 | 91 | 68 |
| NC_019280.1 | Acinetobacter baumannii | pMMD | 1.0E-32 | 95 | 90 | 227 |
| NC_019345.1 | Acinetobacter baumannii | pRAY*-v2 | 8.0E-34 | 95.05 | 91 | 307 |
| NC_016977.1 | Acinetobacter baumannii | pTS236 | 9.0E-40 | 97.03 | 101 | 4 |
| NC_010395.1 | Acinetobacter baumannii SDF | p1ABSDF | 5.0E-35 | 95 | 100 | 1 |
| NC_010396.1 | Acinetobacter baumannii SDF | p2ABSDF | 4.0E-31 | 95.05 | 90 | 96 |
| NC_010398.1 | Acinetobacter baumannii SDF | p3ABSDF | 3.0E-32 | 95 | 91 | 153 |
| NC_020818.1 | Acinetobacter baumannii strain GF216 | pNDM-AB | 3.0E-35 | 95.05 | 90 | 342 |
| NC_019985.2 | Acinetobacter baumannii strain ZW85-1 | pAbNDM-1 | 1.0E-33 | 95.05 | 90 | 445 |
| NC_017166.1 | Acinetobacter baumannii TCDC-AB0715 | p2ABTCDC0715 | 1.0E-35 | 97.85 | 93 | 21 |
| NC_023031.1 | Acinetobacter baumannii ZW85-1 | ZW85p2 | 5.0E-31 | 95.05 | 90 | 314 |
| NC_023322.1 | Acinetobacter bereziniae strain CHI-40-1 | pNDM-BJ01 | 7.0E-37 | 100 | 90 | 4 |
| NC_013056.1 | Acinetobacter calcoaceticus strain Acal H12O-07 | pMMCU1 | 2.0E-36 | 95.05 | 101 | 1 |
| NC_019323.1 | Acinetobacter lwoffii | pABZ78 | 9.0E-37 | 95.05 | 97 | 6 |
| NC_019268.1 | Acinetobacter lwoffii | pNDM-BJ01 | 1.0E-40 | 99.01 | 97 | 14 |
| NC_023280.1 | Acinetobacter nosocomialis | pRAY*-v3 | 1.0E-35 | 96.04 | 91 | 18 |
| NC_019322.1 | Acinetobacter pittii | pABCA95 | 3.0E-36 | 96.04 | 91 | 196 |
| NC_002760.1 | Acinetobacter sp. EB104, | pAC450, | 2.0E-38 | 96.04 | 101 | 2 |
| NC_000923.1 | Acinetobacter sp. SUN resistance | pRAY, | 2.0E-39 | 97.03 | 101 | 3 |
| NC_010309.1 | Acinetobacter venetianus strain VE-C3 | pAV1 | 5.0E-32 | 95 | 90 | 236 |
| NC_010310.1 | Acinetobacter venetianus strain VE-C3 | pAV2 | 5.0E-33 | 95 | 90 | 901 |
| NC_007098.1 | Actinobacillus pleuropneumoniae | pKMA2425 | 5.0E-44 | 100 | 101 | 7 |
| NC_010795.1 | Actinobacillus pleuropneumoniae | pHB0503 | 3.0E-43 | 100 | 101 | 3 |
| NC_005312.1 | Actinobacillus pleuropneumoniae | pMS260 | 4.0E-38 | 96.04 | 101 | 4 |
| NC_007099.1 | Actinobacillus pleuropneumoniae | pPSAS1522 | 7.0E-44 | 100 | 101 | 2 |
| NC_003125.1 | Actinobacillus pleuropneumoniae | pTYM1 | 7.0E-44 | 100 | 101 | 5 |
| NC_019014.1 | Aeromonas aquariorum AAK1 | pAAk1 | 4.0E-41 | 98.02 | 101 | 1 |
| NC_009476.1 | Aeromonas bestiarum 5S9 | pAb5S9 | 2.0E-41 | 99.01 | 101 | 17 |
| NC_006143.1 | Aeromonas caviae | pFBAOT6 | 2.0E-34 | 95 | 90 | 1458 |
| NC_013780.1 | Aeromonas hydrophila | pAH3680 | 6.0E-44 | 100 | 101 | 1 |
| NC_016852.1 | Aeromonas hydrophila | pAHH01 | 2.0E-41 | 99 | 100 | 8 |
| NC_019262.1 | Aeromonas hydrophila | pAHH04 | 5.0E-42 | 99.01 | 100 | 2 |
| NC_021159.1 | Aeromonas hydrophila | pAhy2.5 | 1.0E-38 | 96.04 | 101 | 3 |
| NC_011207.1 | Aeromonas hydrophila | pBRST7.6 | 3.0E-39 | 97.03 | 101 | 9 |
| NC_019380.1 | Aeromonas hydrophila | pR148 | 3.0E-42 | 100 | 101 | 1 |
| NC_012885.1 | Aeromonas hydrophila | pRA1 | 1.0E-40 | 99.01 | 101 | 6 |
| NC_010919.1 | Aeromonas hydrophila | pRA3 | 2.0E-38 | 97.03 | 101 | 6 |
| NC_003124.1 | Aeromonas salmonicida | pRAS3.2 | 1.0E-33 | 95 | 91 | 122 |
| NC_004924.1 | Aeromonas salmonicida salmonicida A449 | pAsa3 | 2.0E-39 | 97.03 | 98 | 2 |
| NC_009349.1 | Aeromonas salmonicida subsp. salmonicida A449 | 4 | 1.0E-34 | 95 | 90 | 113 |
| NC_009350.1 | Aeromonas salmonicida subsp. salmonicida A449 | 5 | 2.0E-31 | 95.05 | 91 | 82 |
| NC_004338.1 | Aeromonas salmonicida subsp. salmonicida | pAsal1 | 4.0E-36 | 95 | 99 | 13 |
| NC_004339.1 | Aeromonas salmonicida subsp. Salmonicida | pAsal2 | 1.0E-35 | 95 | 96 | 9 |
| NC_004340.1 | Aeromonas salmonicida subsp. Salmonicida | pAsal3 | 1.0E-36 | 97.85 | 93 | 2 |
| NC_009352.2 | Aeromonas salmonicida subsp. salmonicida | pAsa6 | 4.0E-36 | 96 | 90 | 14 |
| NC_016611.1 | Aeromonas sobria | pAQ2-1 | 6.0E-35 | 95.05 | 92 | 16 |
| NC_002579.1 | Aggregatibacter actinomycetemcomitans | pVT745 | 9.0E-39 | 97.03 | 95 | 5 |
| NC_003064.2 | Agrobacterium fabrum str. C58 | At | 1.0E-34 | 95.05 | 99 | 4 |
| NC_011990.1 | Agrobacterium radiobacter K84 | pAtK84b | 3.0E-35 | 95.05 | 101 | 2 |
| NC_011987.1 | Agrobacterium radiobacter K84 | pAtK84c | 2.0E-34 | 95.96 | 92 | 3 |
| NC_002575.1 | Agrobacterium rhizogenes | pRi1724 DNA | 1.0E-35 | 95.96 | 99 | 1 |
| NC_010841.1 | Agrobacterium rhizogenes | pRi2659 | 3.0E-35 | 95.05 | 101 | 1 |
| NC_015184.1 | Agrobacterium sp. H13-3 | pAspH13-3a | 1.0E-34 | 95.05 | 95 | 8 |
| NC_006277.2 | Agrobacterium tumefaciens K84 | pAgK84 | 1.0E-34 | 95.05 | 101 | 4 |
| NC_002147.1 | Agrobacterium tumefaciens MAFF301001 | pTi-SAKURA | 4.0E-35 | 95.05 | 98 | 4 |
| NC_019555.1 | Agrobacterium tumefaciens | pAoF64/95 | 1.0E-34 | 95 | 98 | 18 |
| NC_002377.1 | Agrobacterium tumefaciens | Ti | 5.0E-33 | 95.05 | 101 | 1 |
| NC_011986.1 | Agrobacterium vitis S4 | pAtS4a | 1.0E-35 | 95.05 | 98 | 19 |
| NC_011991.1 | Agrobacterium vitis S4 | pAtS4b | 2.0E-35 | 95.05 | 101 | 10 |
| NC_011984.1 | Agrobacterium vitis S4 | pAtS4c | 1.0E-34 | 95 | 98 | 5 |
| NC_011981.1 | Agrobacterium vitis S4 | pAtS4e | 6.0E-32 | 96.04 | 91 | 3 |
| NC_011982.1 | Agrobacterium vitis S4 | pTiS4 | 2.0E-33 | 95.05 | 93 | 17 |
| NC_014908.1 | Alicycliphilus denitrificans BC | pALIDE01 | 5.0E-31 | 95 | 90 | 170 |
| NC_014911.1 | Alicycliphilus denitrificans BC | pALIDE02 | 9.0E-32 | 95.05 | 90 | 253 |
| NC_015423.1 | Alicycliphilus denitrificans K601 | pALIDE201 | 3.0E-32 | 95 | 90 | 76 |
| NC_021709.1 | Alteromonas macleodii str. 'English Channel 615' |  | 9.0E-37 | 100 | 91 | 6 |
| NC_007412.1 | Anabaena variabilis ATCC 29413 | C | 2.0E-39 | 98.02 | 101 | 4 |
| NC_013164.1 | Anaerococcus prevotii DSM 20548 | pAPRE01 | 2.0E-35 | 95.05 | 97 | 38 |
| NC_006823.1 | Azoarcus sp. EbN1 | 1 | 1.0E-34 | 95 | 90 | 146 |
| NC_006824.1 | Azoarcus sp. EbN1 | 2 | 2.0E-32 | 95.05 | 93 | 4 |
| NC_020548.1 | Azoarcus sp. KH32C | pAZKH DNA, | 2.0E-32 | 95 | 94 | 6 |
| NC_016594.1 | Azospirillum brasilense Sp245 | AZOBR_p1 | 3.0E-34 | 95.05 | 98 | 4 |
| NC_016618.1 | Azospirillum brasilense Sp245 | AZOBR_p2 | 2.0E-32 | 95.05 | 94 | 4 |
| NC_016596.1 | Azospirillum brasilense Sp245 | AZOBR_p4 | 1.0E-34 | 95.05 | 95 | 16 |
| NC_016585.1 | Azospirillum lipoferum 4B | AZO_p1 | 3.0E-31 | 95.05 | 91 | 9 |
| NC_016586.1 | Azospirillum lipoferum 4B | AZO_p2 | 6.0E-33 | 95 | 95 | 3 |
| NC_016623.1 | Azospirillum lipoferum 4B | AZO_p3 | 6.0E-32 | 95.05 | 93 | 8 |
| NC_016624.1 | Azospirillum lipoferum 4B | AZO_p5 | 2.0E-30 | 95.05 | 90 | 29 |
| NC_016588.1 | Azospirillum lipoferum 4B | AZO_p6 | 4.0E-36 | 96 | 100 | 1 |
| NC_013855.1 | Azospirillum sp. B510 | pAB510a | 3.0E-34 | 95.05 | 101 | 2 |
| NC_013856.1 | Azospirillum sp. B510 | pAB510b | 2.0E-31 | 95.05 | 90 | 18 |
| NC_013857.1 | Azospirillum sp. B510 | pAB510c | 3.0E-35 | 95.96 | 99 | 4 |
| NC_013858.1 | Azospirillum sp. B510 | pAB510d | 6.0E-32 | 95.7 | 93 | 3 |
| NC_013859.1 | Azospirillum sp. B510 | pAB510e | 4.0E-33 | 95 | 90 | 25 |
| NC_018492.1 | Bacillus cereus FRI-35 | p01 | 1.0E-40 | 99.01 | 101 | 3 |
| NC_011973.1 | Bacillus cereus Q1 | pBc239 | 2.0E-39 | 98.02 | 101 | 5 |
| NC_010924.1 | Bacillus cereus strain AH187 | pCER270 | 2.0E-34 | 95.05 | 101 | 4 |
| NC_010934.1 | Bacillus cereus strain G9241 | pBCXO1 | 3.0E-42 | 100 | 101 | 1 |
| NC_004604.2 | Bacillus megaterium QM B1551 | pBM400 | 3.0E-29 | 95 | 90 | 1857 |
| NC_017139.1 | Bacillus megaterium WSH-002 | WSH-002_p1 | 3.0E-31 | 95 | 90 | 40 |
| NC_013792.1 | Bacillus pseudofirmus OF4 | pBpOF4-01 | 4.0E-36 | 96 | 100 | 9 |
| NC_013963.1 | Bacillus sp. BS-01 | pBS-01 | 3.0E-37 | 98.02 | 90 | 876 |
| NC_014557.1 | Bacillus sp. BS-02 | pBS-02 | 3.0E-36 | 95.05 | 101 | 22 |
| NC_014937.1 | Bacillus thuringiensis CT43 | pBMB0558 | 2.0E-35 | 95.05 | 97 | 42 |
| NC_023074.1 | Bacillus thuringiensis serovar tenebrionis str. YBT-1765 | pBMB165 | 5.0E-41 | 99.01 | 101 | 8 |
| NC_019783.1 | Bacterium 36B | pTOR_02 | 1.0E-32 | 95.65 | 92 | 51 |
| NC_019798.1 | Bacterium 72B | pTOR_01 | 3.0E-37 | 96 | 90 | 18 |
| NC_005026.1 | Bacteroides fragilis IB143 | pBI143 | 2.0E-30 | 95.05 | 90 | 57 |
| NC_006873.1 | Bacteroides fragilis NCTC 9343 | pBF9343 | 2.0E-35 | 95.05 | 90 | 80 |
| NC_011073.1 | Bacteroides fragilis | pBFP35 | 3.0E-37 | 98.91 | 90 | 57 |
| NC_019534.1 | Bacteroides fragilis | pBFUK1 | 1.0E-33 | 95.05 | 93 | 86 |
| NC_006297.1 | Bacteroides fragilis YCH46 | pBFY46 | 1.0E-37 | 96.04 | 101 | 2 |
| NC_015166.1 | Bacteroides salanitronis DSM 18170 | pBACSA03 | 3.0E-38 | 96.04 | 101 | 4 |
| NC_004703.1 | Bacteroides thetaiotaomicron VPI-5482 | p5482 | 9.0E-34 | 95.92 | 92 | 39 |
| NC_011332.1 | Bifidobacterium bifidum | pB80 | 9.0E-44 | 100 | 101 | 5 |
| NC_007068.1 | Bifidobacterium catenulatum | pBC1 | 1.0E-38 | 96.04 | 101 | 1 |
| NC_021875.1 | Bifidobacterium kashiwanohense JCM 15439 | pBBKW-1 DNA | 1.0E-37 | 97.03 | 90 | 9 |
| NC_004253.1 | Bifidobacterium longum DJO10A | pDOJH10S | 2.0E-38 | 96.04 | 101 | 11 |
| NC_002635.1 | Bifidobacterium longum KJ | pKJ36 | 5.0E-39 | 99.01 | 92 | 10 |
| NC_004978.1 | Bifidobacterium longum KJ | pKJ50 | 2.0E-38 | 98.02 | 91 | 32 |
| NC_004943.1 | Bifidobacterium longum NCC2705 | pBLO1 | 1.0E-39 | 97.03 | 101 | 3 |
| NC_010857.1 | Bifidobacterium longum | p6043A | 6.0E-39 | 98.02 | 92 | 57 |
| NC_011139.1 | Bifidobacterium longum | pFI2576 | 3.0E-39 | 97.06 | 101 | 22 |
| NC_006997.1 | Bifidobacterium longum | pMG1 | 3.0E-42 | 99.01 | 101 | 3 |
| NC_019200.1 | Bifidobacterium longum | pSP02 | 8.0E-44 | 100 | 101 | 1 |
| NC_006843.1 | Bifidobacterium longum | pTB6 | 1.0E-39 | 97.03 | 101 | 1 |
| NC_004769.1 | Bifidobacterium longum RW041 | PNAC2 | 1.0E-39 | 97.03 | 101 | 2 |
| NC_004768.1 | Bifidobacterium longum RW041 | pNAC3 | 1.0E-33 | 95.05 | 90 | 114 |
| NC_004770.1 | Bifidobacterium longum RW048 | pNAC1 | 4.0E-34 | 95.7 | 93 | 20 |
| NC_017220.1 | Bifidobacterium longum subsp. longum KACC 91563 | BLNIAS_P1 | 4.0E-41 | 98.02 | 101 | 5 |
| NC_017222.1 | Bifidobacterium longum subsp. longum KACC 91563 | BLNIAS_P2 | 1.0E-36 | 95.05 | 101 | 4 |
| NC_004443.1 | Bifidobacterium longum VMKB44 | pB44 | 5.0E-39 | 97.03 | 97 | 4 |
| NC_008459.1 | Bordetella pertussis | pBP136 DNA | 1.0E-39 | 98.02 | 95 | 42 |
| NC_009475.1 | Bradyrhizobium sp. BTAi1 | pBBta01 | 4.0E-35 | 95.05 | 101 | 1 |
| NC_022590.1 | Brevibacterium sp. Ap13 | pAP13 | 1.0E-42 | 100 | 101 | 1 |
| NC_008385.1 | Burkholderia cepacia AMMD | 1 | 3.0E-35 | 95.05 | 90 | 1491 |
| NC_019378.1 | Burkholderia cepacia | pIJB1 | 3.0E-32 | 95.05 | 90 | 250 |
| NC_019369.1 | Burkholderia cepacia | pYS1 | 5.0E-35 | 95 | 92 | 13 |
| NC_010625.1 | Burkholderia phymatum STM815 | pBPHY01 | 3.0E-34 | 95.05 | 101 | 1 |
| NC_022995.1 | Burkholderia sp. M701 | pM7012 DNA | 6.0E-31 | 95 | 91 | 53 |
| NC_016626.1 | Burkholderia sp. YI23 | byi_1p | 3.0E-34 | 95.05 | 99 | 5 |
| NC_016591.1 | Burkholderia sp. YI23 | byi_2p | 1.0E-36 | 96.04 | 92 | 19 |
| NC_009227.1 | Burkholderia vietnamiensis G4 | pBVIE02 | 5.0E-35 | 95.96 | 91 | 136 |
| NC_009229.1 | Burkholderia vietnamiensis G4 | pBVIE03 | 1.0E-34 | 95 | 98 | 10 |
| NC_009228.1 | Burkholderia vietnamiensis G4 | pBVIE04 | 3.0E-33 | 95.74 | 94 | 1 |
| NC_022355.1 | Campylobacter coli CVM N29710 | pN29710-1 | 4.0E-34 | 95.05 | 90 | 44 |
| NC_006134.1 | Campylobacter coli | pCC31 | 2.0E-32 | 95.05 | 90 | 243 |
| NC_022354.1 | Campylobacter jejuni subsp. jejuni 00-2544 |  | 6.0E-32 | 95.05 | 91 | 9 |
| NC_008790.1 | Campylobacter jejuni subsp. jejuni 81-176 | pTet | 1.0E-40 | 99 | 100 | 3 |
| NC_007141.1 | Campylobacter jejuni subsp. jejuni 81-176 | pTet | 2.0E-37 | 100 | 91 | 2 |
| NC_014801.1 | Campylobacter jejuni subsp. jejuni ICDCCJ07001 | pTet | 1.0E-32 | 95.05 | 90 | 124 |
| NC_013193.1 | Candidatus Accumulibacter phosphatis clade IIA str. UW-1 | pAph01 | 9.0E-36 | 96.91 | 97 | 2 |
| NC_013190.1 | Candidatus Accumulibacter phosphatis clade IIA str. UW-1 | pAph02 | 2.0E-36 | 95.96 | 91 | 22 |
| NC_013191.1 | Candidatus Accumulibacter phosphatis clade IIA str. UW-1 | pAph03 | 9.0E-35 | 95 | 94 | 99 |
| NC_015390.1 | Carnobacterium sp. 17-4 | pCAR50 | 2.0E-32 | 95.92 | 90 | 13 |
| NC_022601.1 | Carnobacterium sp. WN1359 | pWNCR12 | 5.0E-32 | 96.04 | 90 | 70 |
| NC_022607.1 | Carnobacterium sp. WN1359 | pWNCR15 | 4.0E-41 | 99 | 100 | 2 |
| NC_022602.1 | Carnobacterium sp. WN1359 | pWNCR47 | 6.0E-32 | 95 | 90 | 484 |
| NC_022603.1 | Carnobacterium sp. WN1359 | pWNCR64 | 1.0E-35 | 95.05 | 99 | 67 |
| NC_022608.1 | Carnobacterium sp. WN1359 | pWNCR9 | 5.0E-37 | 97.03 | 94 | 9 |
| NC_010335.1 | Caulobacter sp. K31 | pCAUL01 | 4.0E-35 | 95.05 | 101 | 2 |
| NC_010333.1 | Caulobacter sp. K31 | pCAUL02 | 3.0E-42 | 100 | 101 | 2 |
| NC_008244.1 | Chelativorans sp. BNC1 | 3 | 4.0E-40 | 98.02 | 101 | 1 |
| NC_022552.1 | Citrobacter freundii NDMCF | pNDMCFuy | 2.0E-39 | 98.02 | 101 | 2 |
| NC_019360.1 | Citrobacter freundii | pNDM-CIT | 2.0E-39 | 98.02 | 100 | 42 |
| NC_020122.1 | Citrobacter freundii strain CFSTE | pN-Cit | 1.0E-38 | 97.03 | 101 | 4 |
| NC_020123.1 | Citrobacter freundii strain CFSTE | pT-OXA-181 | 3.0E-32 | 95.05 | 90 | 38 |
| NC_019983.1 | Citrobacter freundii strain Iona 2 | pCFI-1 | 4.0E-36 | 95.05 | 101 | 9 |
| NC_019991.1 | Citrobacter freundii strain Iona 4 | pCFI-2 | 2.0E-41 | 99.01 | 101 | 6 |
| NC_019984.1 | Citrobacter freundii strain Iona 6 | pCFI-3 | 4.0E-36 | 95.05 | 101 | 1 |
| NC_009794.1 | Citrobacter koseri ATCC BAA-895 | pCKO2 | 3.0E-43 | 100 | 100 | 2 |
| NC_009793.1 | Citrobacter koseri ATCC BAA-895 | pCKO3 | 2.0E-43 | 100 | 101 | 4 |
| NC_013717.1 | Citrobacter rodentium ICC168 | pCROD1 | 9.0E-43 | 100 | 101 | 1 |
| NC_013718.1 | Citrobacter rodentium ICC168 | pCROD2 | 5.0E-38 | 99.01 | 92 | 16 |
| NC_003114.1 | Citrobacter rodentium strain DBS100 | pCRP3 | 2.0E-42 | 99.01 | 101 | 2 |
| NC_007773.1 | Clostridium perfringens | pCPF5603 | 5.0E-41 | 99.01 | 101 | 10 |
| NC_010937.1 | Clostridium perfringens | pCW3 | 4.0E-40 | 98.02 | 101 | 25 |
| NC_001388.1 | Clostridium perfringens | pIP404 | 5.0E-38 | 96.04 | 101 | 1 |
| NC_019259.1 | Clostridium perfringens | pJIR3537 | 8.0E-43 | 100 | 101 | 2 |
| NC_019688.1 | Clostridium perfringens | pNetB-NE10 | 1.0E-42 | 100 | 101 | 3 |
| NC_003042.1 | Clostridium perfringens str. 13 | pCP13 | 2.0E-38 | 97.03 | 101 | 4 |
| NC_010332.1 | Collimonas fungivorans | pTer331 | 3.0E-35 | 95.05 | 92 | 9 |
| NC_021077.1 | Comamonas sp. 7D-2 | pBHB | 1.0E-31 | 95 | 90 | 115 |
| NC_010935.1 | Comamonas testosteroni CNB-1 | pCNB | 3.0E-32 | 95 | 90 | 1559 |
| NC_016978.1 | Comamonas testosteroni | pI2 | 8.0E-33 | 95.05 | 91 | 43 |
| NC_016968.1 | Comamonas testosteroni | pTB30 | 1.0E-42 | 100 | 101 | 39 |
| NC_002143.1 | Comamonas testosteroni PtL5 cryptic | pPT1 | 5.0E-33 | 95 | 90 | 207 |
| NC_010813.1 | Corynebacterium aurimucosum ATCC 700975 | pET44827 | 6.0E-36 | 95.05 | 100 | 4 |
| NC_003227.1 | Corynebacterium glutamicum | pTET3 | 5.0E-36 | 95.05 | 101 | 3 |
| NC_001791.1 | Corynebacterium glutamicum strain 1014 | pXZ10145.1 | 2.0E-39 | 97.03 | 101 | 1 |
| NC_004945.1 | Corynebacterium glutamicum strain ATCC31830 | pCG4 | 6.0E-36 | 95.05 | 91 | 5 |
| NC_004774.1 | Corynebacterium jeikeium | pA501 | 9.0E-38 | 96.04 | 101 | 1 |
| NC_021920.1 | Corynebacterium maris DSM 45190 | pCmaris1 | 3.0E-35 | 95 | 100 | 3 |
| NC_014167.1 | Corynebacterium resistens DSM 45100 | pJA144188 | 7.0E-35 | 95.05 | 91 | 84 |
| NC_009129.1 | Corynebacterium sp. L2-79-05 | pLEW279b | 2.0E-36 | 95.96 | 94 | 13 |
| NC_004939.1 | Corynebacterium striatum strain M82B | pTP10 | 4.0E-40 | 98.02 | 101 | 2 |
| NC_009779.1 | Cronobacter sakazakii ATCC BAA-894 | pESA2 | 1.0E-32 | 95.05 | 90 | 574 |
| NC_023025.1 | Cronobacter sakazakii CMCC 45402 | p2 | 1.0E-35 | 95.92 | 97 | 49 |
| NC_020261.1 | Cronobacter sakazakii Sp291 | pSP291-2 | 1.0E-35 | 96.04 | 93 | 37 |
| NC_020262.1 | Cronobacter sakazakii Sp291 | pSP291-3 | 8.0E-44 | 100 | 101 | 1 |
| NC_021293.1 | Cronobacter sakazakii strain ATCC 29544 | pCSA2 | 9.0E-44 | 100 | 101 | 7 |
| NC_013285.1 | Cronobacter turicensis z3032 | pCTU3 | 9.0E-43 | 100 | 101 | 1 |
| NC_007974.2 | Cupriavidus metallidurans CH34 |  | 3.0E-29 | 95 | 90 | 665 |
| NC_006525.1 | Cupriavidus metallidurans CH34 | pMOL28 | 5.0E-33 | 95 | 91 | 49 |
| NC_006466.1 | Cupriavidus metallidurans CH34 | pMOL30 | 4.0E-35 | 95.05 | 101 | 1 |
| NC_015727.1 | Cupriavidus necator N-1 | BB1p | 5.0E-31 | 95 | 91 | 74 |
| NC_012527.1 | Deinococcus deserti VCD115 | 1 | 2.0E-29 | 95 | 90 | 63 |
| NC_008010.2 | Deinococcus geothermalis DSM 11300 | pDGEO01 | 2.0E-30 | 95.56 | 90 | 10 |
| NC_019789.1 | Deinococcus peraridilitoris DSM 19664 | pDEIPE01 | 6.0E-31 | 95 | 90 | 60 |
| NC_015163.1 | Deinococcus proteolyticus MRP | pDEIPR04 | 2.0E-42 | 100 | 101 | 1 |
| NC_005088.1 | Delftia acidovorans B | pUO1 | 8.0E-32 | 95 | 90 | 407 |
| NC_019264.1 | Delftia acidovorans | pNB8c | 9.0E-37 | 96 | 90 | 24 |
| NC_019312.1 | Delftia sp. KV29 | pKV29 | 3.0E-30 | 95 | 90 | 6403 |
| NC_012797.1 | Desulfovibrio magneticus RS-1 | pDMC1 | 1.0E-42 | 100 | 101 | 1 |
| NC_009955.1 | Dinoroseobacter shibae DFL 12 | pDSHI01 | 1.0E-33 | 95 | 92 | 13 |
| NC_009956.1 | Dinoroseobacter shibae DFL 12 | pDSHI02 | 1.0E-32 | 95 | 93 | 4 |
| NC_009957.1 | Dinoroseobacter shibae DFL 12 | pDSHI03 | 2.0E-35 | 95.05 | 90 | 86 |
| NC_009958.1 | Dinoroseobacter shibae DFL 12 | pDSHI04 | 6.0E-35 | 95 | 99 | 10 |
| NC_020280.1 | Edwardsiella ictaluri | pEI3 | 3.0E-37 | 97.03 | 91 | 39 |
| NC_014725.1 | Edwardsiella tarda strain CK41 | pCK41 | 5.0E-35 | 95.88 | 97 | 43 |
| NC_018742.1 | Emticicia oligotrophica DSM 17448 | pEMTOL01 | 3.0E-35 | 95.05 | 101 | 1 |
| NC_001735.4 | Enterobacter aerogenes | R751 | 4.0E-35 | 95.05 | 90 | 43 |
| NC_015963.1 | Enterobacter asburiae LF7a | pENTAS01 | 7.0E-37 | 96.04 | 99 | 7 |
| NC_015969.1 | Enterobacter asburiae LF7a | pENTAS02 | 6.0E-36 | 95.05 | 99 | 7 |
| NC_016515.1 | Enterobacter cloacae EcWSU1 | pEcWSU1_A | 8.0E-32 | 95.6 | 91 | 18 |
| NC_012006.1 | Enterobacter cloacae | pCHE-A | 1.0E-37 | 99.01 | 90 | 16 |
| NC_011404.1 | Enterobacter cloacae | pEC01 | 8.0E-38 | 96 | 100 | 1 |
| NC_012555.1 | Enterobacter cloacae | pEC-IMP | 7.0E-34 | 95.05 | 92 | 29 |
| NC_019368.1 | Enterobacter cloacae | pEl1573 | 1.0E-42 | 100 | 101 | 1 |
| NC_019346.1 | Enterobacter cloacae | pNE1280 | 1.0E-35 | 95.05 | 92 | 25 |
| NC_015175.1 | Enterobacter cloacae | pS51A | 1.0E-43 | 100 | 101 | 1 |
| NC_019242.1 | Enterobacter cloacae | pS51B | 4.0E-42 | 99.01 | 101 | 2 |
| NC_017097.1 | Enterobacter cloacae | pUL3AT | 3.0E-34 | 96.04 | 92 | 23 |
| NC_019986.1 | Enterobacter cloacae strain BB1092 | pB1023 | 3.0E-42 | 99.01 | 101 | 18 |
| NC_021087.1 | Enterobacter cloacae strain M15 |  | 2.0E-35 | 95.1 | 94 | 26 |
| NC_014107.1 | Enterobacter cloacae subsp. cloacae ATCC 13047 | pECL_A | 1.0E-34 | 95 | 92 | 191 |
| NC_021492.1 | Enterobacter sp. R4-368 | pENT01 | 8.0E-41 | 99.01 | 101 | 11 |
| NC_015515.1 | Enterobacter sp. W001 | pR23 | 2.0E-36 | 95.05 | 100 | 8 |
| NC_017315.1 | Enterococcus faecalis 62 | EF62pC | 9.0E-43 | 100 | 101 | 1 |
| NC_018222.1 | Enterococcus faecalis D32 | EFD32pA | 5.0E-39 | 97.03 | 101 | 16 |
| NC_002630.1 | Enterococcus faecalis | pAM373 | 2.0E-37 | 97.03 | 101 | 3 |
| NC_013514.1 | Enterococcus faecalis | pAMbeta1 | 5.0E-36 | 96 | 91 | 74 |
| NC_013533.1 | Enterococcus faecalis | pBEE99 | 1.0E-35 | 95.05 | 101 | 15 |
| NC_006827.2 | Enterococcus faecalis | pCF10 | 1.0E-36 | 97.03 | 90 | 90 |
| NC_014508.2 | Enterococcus faecalis | pEF-01 | 7.0E-35 | 95.96 | 91 | 70 |
| NC_011642.1 | Enterococcus faecalis | pMG2200 | 7.0E-35 | 95.88 | 97 | 9 |
| NC_019385.1 | Enterococcus faecalis | PML21 | 1.0E-36 | 95.05 | 99 | 17 |
| NC_014726.1 | Enterococcus faecalis | pTW9 | 6.0E-35 | 95 | 95 | 51 |
| NC_014475.1 | Enterococcus faecalis | pWZ1668 | 9.0E-36 | 95.05 | 90 | 71 |
| NC_019213.1 | Enterococcus faecalis | pWZ909 | 2.0E-37 | 96.04 | 99 | 33 |
| NC_008445.1 | Enterococcus faecalis RE25 | pRE25 | 3.0E-35 | 95.92 | 90 | 50 |
| NC_004669.1 | Enterococcus faecalis V583 | pTEF1 | 5.0E-41 | 99.01 | 101 | 25 |
| NC_004670.1 | Enterococcus faecalis V583 | pTEF3 | 3.0E-43 | 100 | 101 | 11 |
| NC_017032.1 | Enterococcus faecium Aus0004 | AUS0004_p1 | 2.0E-37 | 96.04 | 101 | 3 |
| NC_021987.1 | Enterococcus faecium Aus0085 | p1 | 5.0E-31 | 95.05 | 90 | 104 |
| NC_021995.1 | Enterococcus faecium Aus0085 | p2 | 4.0E-35 | 95 | 91 | 8 |
| NC_021988.1 | Enterococcus faecium Aus0085 | p3 | 6.0E-36 | 95.05 | 96 | 108 |
| NC_017961.1 | Enterococcus faecium DO | 1 | 7.0E-36 | 95.92 | 91 | 86 |
| NC_017962.1 | Enterococcus faecium DO | 2 | 3.0E-31 | 95.05 | 90 | 21 |
| NC_017963.1 | Enterococcus faecium DO | 3 | 2.0E-33 | 95 | 93 | 113 |
| NC_020208.1 | Enterococcus faecium NRRL B-2354 | pNB2354_1 | 2.0E-31 | 95 | 90 | 426 |
| NC_013317.1 | Enterococcus faecium | p5753cA | 7.0E-33 | 95 | 92 | 88 |
| NC_010880.1 | Enterococcus faecium | pEF1 | 4.0E-36 | 95.05 | 101 | 2 |
| NC_021170.1 | Enterococcus faecium | pF856 | 7.0E-35 | 95.05 | 95 | 9 |
| NC_007594.1 | Enterococcus faecium | pHT beta | 1.0E-35 | 95.05 | 101 | 1 |
| NC_011140.1 | Enterococcus faecium | pIP816 | 2.0E-41 | 99.01 | 101 | 2 |
| NC_010290.1 | Enterococcus faecium | pJS33 | 1.0E-39 | 97.03 | 100 | 3 |
| NC_010291.1 | Enterococcus faecium | pJS42 | 7.0E-44 | 100 | 101 | 1 |
| NC_016009.1 | Enterococcus faecium | pM7M2 | 2.0E-34 | 95.79 | 95 | 19 |
| NC_014959.1 | Enterococcus faecium | pS177 | 4.0E-33 | 95 | 93 | 228 |
| NC_008768.1 | Enterococcus faecium | pVEF1 | 1.0E-33 | 95.05 | 92 | 38 |
| NC_010980.1 | Enterococcus faecium | pVEF3 | 4.0E-35 | 95 | 90 | 37 |
| NC_016967.1 | Enterococcus faecium | pZB18 | 5.0E-41 | 99.01 | 101 | 3 |
| NC_005000.1 | Enterococcus faecium U37 | pRUM | 5.0E-36 | 97.85 | 93 | 3 |
| NC_022884.1 | Enterococcus mundtii QU 25 | pQY024 | 6.0E-35 | 95.05 | 101 | 1 |
| NC_020920.1 | Erwinia amylovora CFBP 2585 | pEA3 | 3.0E-40 | 98.99 | 99 | 3 |
| NC_004446.1 | Erwinia amylovora IL-5 | pEA2.8 | 2.0E-42 | 99.01 | 101 | 6 |
| NC_014305.1 | Erwinia billingiae Eb661 | pEB170 | 3.0E-42 | 100 | 101 | 1 |
| NC_013265.1 | Erwinia pyrifoliae Ep1/96 | pEP05 | 1.0E-36 | 95.05 | 101 | 1 |
| NC_017446.1 | Erwinia sp. Ejp617 | pJE02 | 9.0E-44 | 100 | 101 | 1 |
| NC_017627.1 | Escherichia coli 042 | pAA | 4.0E-38 | 97.03 | 101 | 7 |
| NC_010558.1 | Escherichia coli 1520 | pIP1206 | 1.0E-34 | 95.88 | 92 | 15 |
| NC_010719.1 | Escherichia coli 53638 | p53638_226 | 1.0E-35 | 96.91 | 97 | 6 |
| NC_010720.1 | Escherichia coli 53638 | p53638_75 | 8.0E-39 | 97.98 | 99 | 16 |
| NC_011752.1 | Escherichia coli 55989 | 55989p | 6.0E-40 | 98.02 | 101 | 29 |
| NC_007675.1 | Escherichia coli A2363 | pAPEC-O2-ColV | 8.0E-37 | 96.04 | 100 | 6 |
| NC_006671.1 | Escherichia coli A2363 | pAPEC-O2-R | 2.0E-36 | 97.03 | 90 | 22 |
| NC_017629.1 | Escherichia coli ABU 83972 | pABU | 3.0E-37 | 95.05 | 101 | 1 |
| NC_023323.1 | Escherichia coli ACN001 | pACN001-A | 1.0E-35 | 95.05 | 93 | 31 |
| NC_023327.1 | Escherichia coli ACN001 | pACN001-B | 3.0E-35 | 95.05 | 99 | 41 |
| NC_023324.1 | Escherichia coli ACN001 | pACN001-C | 4.0E-42 | 99.01 | 101 | 3 |
| NC_023328.1 | Escherichia coli ACN001 | pACN001-E | 6.0E-41 | 98.02 | 101 | 3 |
| NC_023326.1 | Escherichia coli ACN001 | pACN001-F | 1.0E-42 | 100 | 101 | 17 |
| NC_009837.1 | Escherichia coli APEC O1 | pAPEC-O1-ColBM | 1.0E-40 | 99.01 | 101 | 4 |
| NC_009838.1 | Escherichia coli APEC O1 | pAPEC-O1-R | 1.0E-36 | 97.03 | 91 | 64 |
| NC_002142.1 | Escherichia coli B171 | pB171 | 4.0E-36 | 98.91 | 92 | 17 |
| NC_011980.1 | Escherichia coli chi7122 | pAPEC-1 | 7.0E-35 | 96.04 | 90 | 24 |
| NC_009787.1 | Escherichia coli E24377A | pETEC_35 | 1.0E-38 | 97.03 | 101 | 4 |
| NC_009791.1 | Escherichia coli E24377A | pETEC_5 | 4.0E-42 | 99.01 | 101 | 4 |
| NC_009788.1 | Escherichia coli E24377A | pETEC_73 | 6.0E-40 | 98.02 | 101 | 16 |
| NC_009790.1 | Escherichia coli E24377A | pETEC_74 | 6.0E-40 | 98.02 | 101 | 9 |
| NC_009786.1 | Escherichia coli E24377A | pETEC_80 | 6.0E-34 | 96.04 | 93 | 14 |
| NC_011754.1 | Escherichia coli ED1a | pECOED | 2.0E-42 | 100 | 101 | 4 |
| NC_007365.1 | Escherichia coli EH41 | pO113 | 7.0E-37 | 96.04 | 99 | 17 |
| NC_014233.1 | Escherichia coli ETEC 1392/75 | p557 | 3.0E-36 | 98.02 | 92 | 29 |
| NC_014234.1 | Escherichia coli ETEC 1392/75 | p746 | 6.0E-40 | 98.02 | 101 | 19 |
| NC_014235.1 | Escherichia coli ETEC 1392/75 | p75 | 5.0E-42 | 99.01 | 101 | 5 |
| NC_017724.1 | Escherichia coli ETEC H10407 | p948 | 1.0E-36 | 98.92 | 93 | 17 |
| NC_013507.1 | Escherichia coli ETEC H10407 | pEntH10407 | 1.0E-42 | 100 | 101 | 5 |
| NC_018998.1 | Escherichia coli F18+ | pTC1 | 6.0E-41 | 99.01 | 101 | 14 |
| NC_022742.1 | Escherichia coli HUSEC2011 | pHUSEC2011-1 | 4.0E-37 | 100 | 91 | 38 |
| NC_022743.1 | Escherichia coli HUSEC2011 | pHUSEC2011-2 | 5.0E-35 | 95 | 91 | 35 |
| NC_022741.1 | Escherichia coli HUSEC2011 | pHUSEC2011-3 | 1.0E-42 | 99.01 | 101 | 9 |
| NC_022661.1 | Escherichia coli JJ1886 | pJJ1886_1 | 6.0E-40 | 97.03 | 101 | 8 |
| NC_022649.1 | Escherichia coli JJ1886 | pJJ1886_2 | 3.0E-37 | 96.04 | 92 | 11 |
| NC_022662.1 | Escherichia coli JJ1886 | pJJ1886_3 | 4.0E-42 | 99.01 | 99 | 47 |
| NC_022650.1 | Escherichia coli JJ1886 | pJJ1886_4 | 1.0E-35 | 95.05 | 99 | 13 |
| NC_022651.1 | Escherichia coli JJ1886 | pJJ1886_5 | 2.0E-35 | 95.05 | 97 | 65 |
| NC_002483.1 | Escherichia coli K-12 | F DNA | 4.0E-37 | 96.04 | 101 | 8 |
| NC_002525.1 | Escherichia coli K-12 | R721 | 5.0E-35 | 95.05 | 90 | 18 |
| NC_002145.1 | Escherichia coli KL4 | pKL1 | 3.0E-44 | 100 | 101 | 2 |
| NC_016904.1 | Escherichia coli KO11FL | pEKO1101 | 7.0E-41 | 99.01 | 101 | 12 |
| NC_011917.1 | Escherichia coli LF82 | plLF82 | 2.0E-35 | 95.05 | 97 | 85 |
| NC_013354.1 | Escherichia coli O103:H2 str. 12009 | pO103 | 5.0E-41 | 99.01 | 101 | 10 |
| NC_018651.1 | Escherichia coli O104:H4 str. 2009EL-2050 | p09EL50 | 4.0E-32 | 95.65 | 92 | 24 |
| NC_018654.1 | Escherichia coli O104:H4 str. 2009EL-2050 | pAA-09EL50 | 3.0E-37 | 97.98 | 91 | 42 |
| NC_018666.1 | Escherichia coli O104:H4 str. 2011C-3493 | pAA-EA11 | 5.0E-41 | 99.01 | 101 | 1 |
| NC_013365.1 | Escherichia coli O111:H- str. 11128 | pO111_1 | 7.0E-38 | 100 | 93 | 3 |
| NC_013370.1 | Escherichia coli O111:H- str. 11128 | pO111_2 | 4.0E-31 | 95.05 | 90 | 66 |
| NC_013366.1 | Escherichia coli O111:H- str. 11128 | pO111_3 | 3.0E-37 | 96.04 | 92 | 12 |
| NC_013367.1 | Escherichia coli O111:H- str. 11128 | pO111_4 | 3.0E-39 | 97.03 | 101 | 1 |
| NC_011603.1 | Escherichia coli O127:H6 str. E2348/69 | pMAR2 | 6.0E-35 | 96.04 | 90 | 8 |
| NC_007414.1 | Escherichia coli O157:H7 EDL933 | pO157 | 1.0E-42 | 100 | 101 | 7 |
| NC_002128.1 | Escherichia coli O157:H7 str. Sakai | pO157 | 5.0E-42 | 100 | 100 | 5 |
| NC_002127.1 | Escherichia coli O157:H7 str. Sakai | pOSAK1 | 2.0E-42 | 99.01 | 101 | 2 |
| NC_019087.1 | Escherichia coli O25b:H4-ST131 str. EC958 | pKC396 | 8.0E-36 | 95.05 | 100 | 12 |
| NC_020271.1 | Escherichia coli O25b:H4-ST131 str. EC958 strain ST131 | pJIE186-2 | 9.0E-35 | 95.05 | 101 | 4 |
| NC_013728.1 | Escherichia coli O26:H- | pO26-CRL | 4.0E-38 | 97.03 | 101 | 2 |
| NC_013369.1 | Escherichia coli O26:H11 str. 11368 | pO26_1 | 6.0E-41 | 99.01 | 101 | 9 |
| NC_013362.1 | Escherichia coli O26:H11 str. 11368 | pO26_2 | 3.0E-37 | 96.04 | 101 | 15 |
| NC_013942.1 | Escherichia coli O55:H7 str. CB9615 | pO55 | 7.0E-39 | 99.01 | 94 | 11 |
| NC_017653.1 | Escherichia coli O55:H7 str. RM12579 | p12579_1 | 2.0E-35 | 95.05 | 91 | 38 |
| NC_017655.1 | Escherichia coli O55:H7 str. RM12579 | p12579_5 | 2.0E-39 | 97.03 | 101 | 2 |
| NC_017647.1 | Escherichia coli O7:K1 str. CE10 | pCE10A | 8.0E-37 | 96.04 | 90 | 69 |
| NC_017649.1 | Escherichia coli O7:K1 str. CE10 | pCE10C | 3.0E-42 | 99.01 | 101 | 3 |
| NC_017659.1 | Escherichia coli O83:H1 str. NRG 857C | pO83_CORR | 4.0E-40 | 100 | 97 | 14 |
| NC_001371.1 | Escherichia coli | ColE1 | 3.0E-38 | 98.02 | 91 | 4 |
| NC_014356.1 | Escherichia coli | IncQ-type pQ7 | 3.0E-39 | 98.96 | 96 | 31 |
| NC_010257.1 | Escherichia coli | MccC7-H22 | 1.0E-37 | 96.04 | 101 | 16 |
| NC_004998.1 | Escherichia coli | p1658/97 | 4.0E-32 | 95.05 | 92 | 32 |
| NC_015872.1 | Escherichia coli | p271A | 2.0E-37 | 99.01 | 91 | 31 |
| NC_014843.1 | Escherichia coli | p3521 | 9.0E-34 | 95 | 93 | 8 |
| NC_019094.1 | Escherichia coli | p417H-90 | 1.0E-35 | 97.03 | 91 | 5 |
| NC_011799.1 | Escherichia coli | p5217 | 4.0E-42 | 99.01 | 101 | 1 |
| NC_019062.1 | Escherichia coli | p838C-R1 | 2.0E-35 | 95 | 99 | 131 |
| NC_019051.1 | Escherichia coli | pAm05WL6211 | 1.0E-43 | 100 | 101 | 2 |
| NC_019054.1 | Escherichia coli | pAm08CD7339 | 1.0E-43 | 100 | 101 | 1 |
| NC_019056.1 | Escherichia coli | pAm08CD9902 | 5.0E-38 | 96.04 | 91 | 23 |
| NC_019053.1 | Escherichia coli | pAm08WL3069 | 5.0E-44 | 100 | 101 | 1 |
| NC_011964.1 | Escherichia coli | pAPEC-O103-ColBM | 1.0E-32 | 96.7 | 91 | 11 |
| NC_019091.1 | Escherichia coli | pASL01a | 3.0E-32 | 95.05 | 91 | 51 |
| NC_005327.1 | Escherichia coli | pC15-1a | 1.0E-42 | 100 | 101 | 18 |
| NC_019037.1 | Escherichia coli | pChi7122-2 | 2.0E-35 | 95.05 | 99 | 17 |
| NC_019039.1 | Escherichia coli | pChi7122-3 | 1.0E-35 | 95.05 | 99 | 13 |
| NC_019049.1 | Escherichia coli | pCM959 | 3.0E-36 | 95 | 95 | 41 |
| NC_013589.1 | Escherichia coli | pColE1-H22 | 1.0E-35 | 95.05 | 101 | 1 |
| NC_006881.1 | Escherichia coli | pColK-K235 | 3.0E-33 | 96.67 | 90 | 18 |
| NC_022333.1 | Escherichia coli | pCss165Kan | 1.0E-33 | 95.05 | 91 | 20 |
| NC_014477.1 | Escherichia coli | pCT | 8.0E-40 | 98.02 | 101 | 2 |
| NC_020086.1 | Escherichia coli | pE66An | 7.0E-40 | 98.02 | 101 | 10 |
| NC_014382.1 | Escherichia coli | pEC_B24 | 5.0E-41 | 99.01 | 101 | 3 |
| NC_013175.1 | Escherichia coli | pEC14_114 | 2.0E-42 | 100 | 101 | 1 |
| NC_019083.1 | Escherichia coli | pEC14_35 | 3.0E-40 | 98.02 | 101 | 4 |
| NC_013782.1 | Escherichia coli | pEC14-9 | 1.0E-39 | 97.03 | 101 | 4 |
| NC_011418.1 | Escherichia coli | pEC278 | 2.0E-39 | 99.01 | 93 | 14 |
| NC_019077.1 | Escherichia coli | pEC34A | 6.0E-38 | 96.08 | 102 | 1 |
| NC_021983.1 | Escherichia coli | pEC386IL | 1.0E-42 | 99.01 | 101 | 6 |
| NC_021997.1 | Escherichia coli | pEC386IL | 7.0E-43 | 100 | 101 | 9 |
| NC_021999.1 | Escherichia coli | pEC386IL | 2.0E-42 | 99.01 | 101 | 12 |
| NC_019079.1 | Escherichia coli | pEC886 | 2.0E-43 | 100 | 101 | 1 |
| NC_015472.1 | Escherichia coli | pECTm80 | 8.0E-39 | 97.03 | 101 | 11 |
| NC_019086.1 | Escherichia coli | pECY6-7 | 5.0E-37 | 95.05 | 101 | 1 |
| NC_012690.1 | Escherichia coli | peH4H | 2.0E-38 | 97.98 | 99 | 27 |
| NC_013122.1 | Escherichia coli | pEK499 | 1.0E-39 | 98.02 | 101 | 1 |
| NC_013121.1 | Escherichia coli | pEK516 | 5.0E-41 | 99.01 | 101 | 7 |
| NC_014615.1 | Escherichia coli | pETN48 | 1.0E-33 | 95 | 91 | 41 |
| NC_005923.1 | Escherichia coli | pFL129 | 1.0E-43 | 100 | 101 | 19 |
| NC_019424.1 | Escherichia coli | pFOS-HK151325 | 1.0E-42 | 100 | 101 | 1 |
| NC_019089.1 | Escherichia coli | pGUE-NDM | 9.0E-39 | 98.02 | 94 | 10 |
| NC_019081.1 | Escherichia coli | pHCG11 | 7.0E-41 | 98.99 | 99 | 39 |
| NC_019098.1 | Escherichia coli | pHHA45 | 6.0E-43 | 100 | 101 | 1 |
| NC_019071.1 | Escherichia coli | pHK09 | 6.0E-40 | 98.02 | 101 | 2 |
| NC_016039.1 | Escherichia coli | pHK17a | 5.0E-41 | 99.01 | 101 | 3 |
| NC_022374.1 | Escherichia coli | pHKU1 | 2.0E-37 | 96.04 | 101 | 6 |
| NC_019073.1 | Escherichia coli | pHN7A8 | 5.0E-35 | 97 | 92 | 3 |
| NC_019074.1 | Escherichia coli | pHNDD81-1 | 8.0E-36 | 95 | 94 | 34 |
| NC_018995.1 | Escherichia coli | pHUSEC41-1 | 6.0E-41 | 99.01 | 101 | 3 |
| NC_019000.1 | Escherichia coli | pHUSEC41-2 | 1.0E-36 | 97.94 | 90 | 40 |
| NC_010898.1 | Escherichia coli | pIGRW12 | 9.0E-44 | 100 | 101 | 1 |
| NC_010885.1 | Escherichia coli | pIGWZ12 | 2.0E-39 | 97.03 | 101 | 11 |
| NC_004429.1 | Escherichia coli | pIS2 | 2.0E-39 | 98.96 | 96 | 8 |
| NC_019059.1 | Escherichia coli | pJD8 | 3.0E-37 | 95.05 | 100 | 3 |
| NC_014231.1 | Escherichia coli | pKC394 | 2.0E-37 | 97.92 | 94 | 7 |
| NC_019068.1 | Escherichia coli | pKST21 | 2.0E-40 | 98.06 | 101 | 8 |
| NC_019097.1 | Escherichia coli | Plm | 2.0E-42 | 100 | 101 | 2 |
| NC_010064.1 | Escherichia coli | pLMO226 | 8.0E-40 | 98.02 | 93 | 22 |
| NC_019093.1 | Escherichia coli | pLST424C-61 | 1.0E-35 | 97.94 | 97 | 4 |
| NC_013503.1 | Escherichia coli | pMAS2027 | 7.0E-43 | 100 | 101 | 6 |
| NC_008486.1 | Escherichia coli | pMG828-1 | 3.0E-38 | 96.94 | 98 | 1 |
| NC_008487.1 | Escherichia coli | pMG828-2 | 7.0E-44 | 100 | 101 | 1 |
| NC_008488.1 | Escherichia coli | pMG828-3 | 9.0E-44 | 100 | 101 | 14 |
| NC_008489.1 | Escherichia coli | pMG828-4 | 1.0E-36 | 95.05 | 99 | 14 |
| NC_008490.1 | Escherichia coli | pMG828-5 | 2.0E-36 | 95.05 | 100 | 16 |
| NC_019043.1 | Escherichia coli | pND11_107 | 7.0E-35 | 95 | 100 | 1 |
| NC_018994.1 | Escherichia coli | pNDM-1_Dok01 | 6.0E-32 | 95.05 | 92 | 37 |
| NC_019069.1 | Escherichia coli | pNDM10505 | 2.0E-31 | 95.05 | 91 | 124 |
| NC_022375.1 | Escherichia coli | pNDM-BTR | 2.0E-38 | 97.03 | 101 | 1 |
| NC_019063.1 | Escherichia coli | pNDM-HK | 4.0E-31 | 96.67 | 90 | 3 |
| NC_019047.1 | Escherichia coli | pNGX2-QnrS1 | 6.0E-43 | 100 | 101 | 1 |
| NC_019046.1 | Escherichia coli | pNMEC31_31 | 5.0E-43 | 100 | 101 | 2 |
| NC_022992.1 | Escherichia coli | pO111-CRL-115 | 8.0E-41 | 99.01 | 101 | 10 |
| NC_011812.1 | Escherichia coli | pO26-L | 6.0E-40 | 98.02 | 101 | 4 |
| NC_012487.1 | Escherichia coli | pO26-Vir | 3.0E-35 | 98.9 | 91 | 1 |
| NC_008460.1 | Escherichia coli | pO86A1 | 8.0E-35 | 96.04 | 101 | 15 |
| NC_010378.1 | Escherichia coli | pOLA52 | 3.0E-35 | 96 | 90 | 28 |
| NC_019065.1 | Escherichia coli | pPG010208 | 2.0E-42 | 100 | 101 | 1 |
| NC_013652.1 | Escherichia coli | pPM18 | 1.0E-37 | 96.04 | 94 | 9 |
| NC_019033.1 | Escherichia coli | pQNR2078 | 3.0E-41 | 99.01 | 101 | 6 |
| NC_012886.1 | Escherichia coli | pRAx | 3.0E-35 | 95 | 91 | 44 |
| NC_019088.1 | Escherichia coli | pRPEC180_47 | 1.0E-39 | 98 | 100 | 1 |
| NC_009602.1 | Escherichia coli | pSFO157 | 2.0E-35 | 95.05 | 99 | 27 |
| NC_019013.1 | Escherichia coli | pSYM1 | 4.0E-41 | 99.01 | 101 | 4 |
| NC_011514.1 | Escherichia coli | pTN38148 | 2.0E-43 | 100 | 101 | 1 |
| NC_010409.1 | Escherichia coli | pVM01 | 1.0E-32 | 95.7 | 93 | 89 |
| NC_019082.1 | Escherichia coli | pZS50 | 6.0E-38 | 100 | 92 | 5 |
| NC_001740.1 | Escherichia coli | RSF1010 | 1.0E-43 | 100 | 101 | 40 |
| NC_022371.1 | Escherichia coli PMV-1 | pHUSEC411like | 2.0E-42 | 100 | 101 | 1 |
| NC_011747.1 | Escherichia coli S88 | pECOS88 | 2.0E-35 | 95.05 | 96 | 46 |
| NC_011419.1 | Escherichia coli SE11 | pSE11-1 | 8.0E-40 | 98.02 | 101 | 6 |
| NC_011413.1 | Escherichia coli SE11 | pSE11-2 | 2.0E-34 | 96.81 | 94 | 13 |
| NC_011416.1 | Escherichia coli SE11 | pSE11-3 | 6.0E-39 | 98.02 | 97 | 9 |
| NC_011407.1 | Escherichia coli SE11 | pSE11-4 | 1.0E-36 | 95.05 | 99 | 7 |
| NC_011411.1 | Escherichia coli SE11 | pSE11-6 | 7.0E-44 | 100 | 101 | 1 |
| NC_013655.1 | Escherichia coli SE15 | pECSF1 | 2.0E-42 | 100 | 101 | 3 |
| NC_010488.1 | Escherichia coli SMS-3-5 | pSMS35_130 | 2.0E-35 | 95.05 | 101 | 13 |
| NC_010487.1 | Escherichia coli SMS-3-5 | pSMS35_3 | 5.0E-39 | 97.03 | 101 | 5 |
| NC_010486.1 | Escherichia coli SMS-3-5 | pSMS35_4 | 7.0E-44 | 100 | 101 | 2 |
| NC_020278.2 | Escherichia coli strain 3A11 | pHN3A11 | 6.0E-34 | 95.79 | 90 | 12 |
| NC_023329.1 | Escherichia coli strain B3804 | pIFM3804 | 1.0E-38 | 97.98 | 99 | 2 |
| NC_019990.1 | Escherichia coli strain BB1093 | pB1024 | 1.0E-36 | 95.05 | 90 | 14 |
| NC_023315.1 | Escherichia coli strain EQ011 | pEQ011 | 2.0E-33 | 95.05 | 94 | 53 |
| NC_022885.1 | Escherichia coli strain LK-NARMP | pKPC-LKEc | 2.0E-42 | 100 | 101 | 3 |
| NC_022377.1 | Escherichia coli strain SCEC2 | pSCEC2 | 2.0E-35 | 97.85 | 93 | 1 |
| NC_017630.1 | Escherichia coli UM146 | pUM146 | 2.0E-36 | 96.08 | 101 | 60 |
| NC_011749.1 | Escherichia coli UMN026 | p1ESCUM | 2.0E-36 | 97.89 | 95 | 1 |
| NC_011739.1 | Escherichia coli UMN026 | p2ESCUM | 6.0E-43 | 100 | 101 | 1 |
| NC_017645.1 | Escherichia coli UMNK88 | pUMNK88 | 4.0E-33 | 95.05 | 101 | 2 |
| NC_017642.1 | Escherichia coli UMNK88 | pUMNK88_91 | 1.0E-42 | 100 | 101 | 15 |
| NC_017640.1 | Escherichia coli UMNK88 | pUMNK88_Ent | 4.0E-37 | 96.04 | 101 | 14 |
| NC_017643.1 | Escherichia coli UMNK88 | pUMNK88_Hly | 3.0E-37 | 96.97 | 99 | 11 |
| NC_017639.1 | Escherichia coli UMNK88 | pUMNK88_K88 | 6.0E-41 | 99.01 | 101 | 15 |
| NC_007941.1 | Escherichia coli UTI89 | pUTI89 | 3.0E-34 | 95.05 | 97 | 49 |
| NC_012944.1 | Escherichia coli Vir68 | pVir68 | 3.0E-35 | 95.05 | 101 | 11 |
| NC_017637.1 | Escherichia coli W | pRK1 | 4.0E-37 | 96.04 | 101 | 1 |
| NC_017903.1 | Escherichia coli Xuzhou21 | pO157_Sal | 6.0E-43 | 100 | 101 | 1 |
| NC_011743.1 | Escherichia fergusonii ATCC 35469 | pEFER | 5.0E-33 | 95.05 | 92 | 44 |
| NC_009716.1 | Escherichia sp. Sflu5 cryptic | pAK51 | 5.0E-35 | 95.79 | 92 | 22 |
| NC_012782.1 | Eubacterium eligens ATCC 27750 | unnamed | 6.0E-33 | 95.05 | 91 | 83 |
| NC_012780.1 | Eubacterium eligens ATCC 27750 | unnamed | 8.0E-30 | 95 | 90 | 6286 |
| NC_010607.1 | Exiguobacterium arabatum | pEspA | 3.0E-37 | 97.87 | 94 | 1 |
| NC_023287.1 | Exiguobacterium sp. S3-2 | pMC1 | 7.0E-33 | 95 | 92 | 113 |
| NC_010371.1 | Finegoldia magna ATCC 29328 | pFMC | 3.0E-36 | 97 | 100 | 2 |
| NC_002132.1 | Flavobacterium sp. KP1 | pFL1 | 2.0E-36 | 98.89 | 90 | 3 |
| NC_002109.1 | Francisella tularensis | pOM1 | 8.0E-44 | 100 | 101 | 1 |
| NC_016028.1 | Gluconacetobacter xylinus NBRC 3288 | pGXY030 | 5.0E-43 | 100 | 101 | 2 |
| NC_016907.1 | Gordonia polyisoprenivorans VH2 | p174 | 6.0E-38 | 97.03 | 101 | 3 |
| NC_011409.1 | Haemophilus influenzae | ICEhin1056 | 7.0E-38 | 99.01 | 92 | 11 |
| NC_019184.1 | Haemophilus influenzae | pLFH49 | 2.0E-38 | 100 | 91 | 1 |
| NC_019182.1 | Haemophilus influenzae | pLFH64 | 9.0E-44 | 100 | 101 | 1 |
| NC_019181.1 | Haemophilus influenzae | pPN223 | 7.0E-44 | 100 | 101 | 5 |
| NC_006828.1 | Haemophilus parasuis HS1543 | pHS-Tet | 9.0E-44 | 100 | 101 | 1 |
| NC_012661.1 | Haemophilus parasuis | pHN61 | 1.0E-37 | 98.02 | 90 | 29 |
| NC_022367.1 | Haemophilus parasuis | pTetHS016 | 2.0E-42 | 99.01 | 101 | 5 |
| NC_021186.1 | Haemophilus parasuis strain QY431 | pQY431 | 1.0E-43 | 100 | 101 | 2 |
| NC_008154.1 | Human gut | pTRACA10 | 3.0E-38 | 96.04 | 100 | 4 |
| NC_008153.1 | Human gut | pTRACA17 | 5.0E-44 | 100 | 101 | 2 |
| NC_014633.1 | Ilyobacter polytropus DSM 2926 | pILYOP01 | 5.0E-29 | 95 | 90 | 118 |
| NC_014621.1 | Ketogulonicigenium vulgare Y25 | pYP1 | 1.0E-30 | 95 | 90 | 159 |
| NC_014626.1 | Ketogulonicigenium vulgare Y25 | pYP12 | 4.0E-35 | 95.05 | 101 | 1 |
| NC_017386.1 | Ketogulonigenium vulgarum WSH-001 | 1 | 1.0E-36 | 96.04 | 101 | 1 |
| NC_021501.1 | Klebsiella oxytoca E718 | pKOX_NDM1 | 4.0E-32 | 95.05 | 90 | 90 |
| NC_018107.1 | Klebsiella oxytoca E718 | pKOX_R1 | 3.0E-32 | 96.04 | 91 | 54 |
| NC_019286.1 | Klebsiella oxytoca | pINCan01 | 6.0E-34 | 95.05 | 90 | 82 |
| NC_005018.1 | Klebsiella pneumoniae 2kI | pKPN2 | 4.0E-41 | 98.02 | 101 | 23 |
| NC_011282.1 | Klebsiella pneumoniae 342 | pKP187 | 3.0E-35 | 95.05 | 94 | 75 |
| NC_011281.1 | Klebsiella pneumoniae 342 | pKP91 | 5.0E-36 | 95.96 | 99 | 30 |
| NC_005015.1 | Klebsiella pneumoniae BM4493 | pIP843 | 5.0E-42 | 99.01 | 99 | 21 |
| NC_005249.1 | Klebsiella pneumoniae CG43 | pLVPK | 1.0E-34 | 95 | 90 | 417 |
| NC_021664.2 | Klebsiella pneumoniae FCF1305 | pKPC_FCF13/05 | 1.0E-41 | 100 | 99 | 1 |
| NC_021660.2 | Klebsiella pneumoniae FCF3SP | pKPC_FCF/3SP | 4.0E-41 | 99.01 | 101 | 2 |
| NC_022078.1 | Klebsiella pneumoniae JM45 | p1 | 6.0E-35 | 95.05 | 93 | 25 |
| NC_022083.1 | Klebsiella pneumoniae JM45 | p2 | 9.0E-42 | 99.01 | 101 | 12 |
| NC_017541.1 | Klebsiella pneumoniae KCTC 2242 | pKCTC2242 | 4.0E-35 | 95.05 | 91 | 52 |
| NC_011385.1 | Klebsiella pneumoniae | 12 | 1.0E-41 | 100 | 99 | 6 |
| NC_011383.1 | Klebsiella pneumoniae | 9 | 3.0E-37 | 96.04 | 101 | 6 |
| NC_021666.1 | Klebsiella pneumoniae | ColE-LS6 | 2.0E-43 | 100 | 101 | 3 |
| NC_021667.1 | Klebsiella pneumoniae | IncA/C-LS6 | 2.0E-31 | 95 | 90 | 190 |
| NC_011511.1 | Klebsiella pneumoniae | p169 | 9.0E-36 | 95.05 | 96 | 131 |
| NC_011512.1 | Klebsiella pneumoniae | p9701 | 5.0E-34 | 96.04 | 91 | 42 |
| NC_022520.1 | Klebsiella pneumoniae | pBK15692 | 5.0E-41 | 99.01 | 101 | 9 |
| NC_015154.1 | Klebsiella pneumoniae | pc15-k | 1.0E-36 | 98.92 | 93 | 79 |
| NC_010261.1 | Klebsiella pneumoniae | pH205 | 5.0E-37 | 96.04 | 97 | 5 |
| NC_011406.1 | Klebsiella pneumoniae | pIGMS31 | 1.0E-39 | 97.03 | 93 | 7 |
| NC_019157.1 | Klebsiella pneumoniae | pIncX-SHV | 7.0E-43 | 100 | 101 | 2 |
| NC_003486.1 | Klebsiella pneumoniae | pJHCMW1 | 2.0E-43 | 100 | 101 | 1 |
| NC_020088.1 | Klebsiella pneumoniae strain K18An | pK18An, | 8.0E-37 | 96.97 | 99 | 7 |
| NC_020087.1 | Klebsiella pneumoniae strain K1HV | pK1HV | 3.0E-34 | 95.05 | 91 | 63 |
| NC_010886.1 | Klebsiella pneumoniae | pK245 | 2.0E-35 | 95.05 | 99 | 21 |
| NC_010870.1 | Klebsiella pneumoniae | pK29 | 3.0E-31 | 95.6 | 91 | 24 |
| NC_021622.1 | Klebsiella pneumoniae | pK45-67VIM | 2.0E-38 | 97.03 | 101 | 1 |
| NC_019389.1 | Klebsiella pneumoniae | pKDO1 | 1.0E-39 | 98.02 | 101 | 17 |
| NC_013951.1 | Klebsiella pneumoniae | pKF3-140 | 3.0E-35 | 95.05 | 92 | 113 |
| NC_013542.1 | Klebsiella pneumoniae | pKF3-70 | 6.0E-34 | 96.77 | 93 | 32 |
| NC_013950.1 | Klebsiella pneumoniae | pKF3-94 | 6.0E-35 | 95 | 90 | 66 |
| NC_002610.1 | Klebsiella pneumoniae | pKlebB-k17/80 | 4.0E-35 | 95.05 | 90 | 25 |
| NC_021654.1 | Klebsiella pneumoniae | pKN-LS6 | 1.0E-36 | 96.04 | 96 | 13 |
| NC_014312.1 | Klebsiella pneumoniae | pKP048 | 3.0E-35 | 95.05 | 101 | 41 |
| NC_021576.1 | Klebsiella pneumoniae | pKP1780 | 8.0E-37 | 98.02 | 93 | 33 |
| NC_019160.1 | Klebsiella pneumoniae | pKP3-A | 1.0E-43 | 100 | 101 | 7 |
| NC_021356.1 | Klebsiella pneumoniae | pKP53IL | 6.0E-36 | 95.05 | 90 | 182 |
| NC_021357.1 | Klebsiella pneumoniae | pKP53IL | 6.0E-37 | 96.04 | 100 | 9 |
| NC_021358.1 | Klebsiella pneumoniae | pKP53IL | 1.0E-43 | 100 | 101 | 3 |
| NC_021360.1 | Klebsiella pneumoniae | pKP53IL | 4.0E-44 | 100 | 101 | 1 |
| NC_021363.1 | Klebsiella pneumoniae | pKP53IL | 5.0E-42 | 99.01 | 101 | 11 |
| NC_021364.1 | Klebsiella pneumoniae | pKP53IL | 3.0E-43 | 100 | 100 | 13 |
| NC_020893.1 | Klebsiella pneumoniae | pKPC-LK30 | 1.0E-37 | 97.03 | 92 | 38 |
| NC_019161.1 | Klebsiella pneumoniae | pKPC-NY79 | 1.0E-32 | 95.05 | 92 | 6 |
| NC_019390.1 | Klebsiella pneumoniae | pKPN_CZ | 8.0E-31 | 95 | 90 | 627 |
| NC_019165.1 | Klebsiella pneumoniae | pKPN101-IT | 2.0E-35 | 95.05 | 100 | 29 |
| NC_011640.1 | Klebsiella pneumoniae | pKpn114 | 2.0E-37 | 96.04 | 99 | 21 |
| NC_021502.1 | Klebsiella pneumoniae | pKPoxa-48N2 | 3.0E-35 | 95.05 | 91 | 167 |
| NC_014016.1 | Klebsiella pneumoniae | pKpQIL | 4.0E-38 | 97.03 | 99 | 14 |
| NC_019155.1 | Klebsiella pneumoniae | pKpQIL-IT | 8.0E-41 | 99.01 | 101 | 3 |
| NC_021655.1 | Klebsiella pneumoniae | pKpQIL-LS6 | 7.0E-40 | 98.02 | 96 | 23 |
| NC_021656.1 | Klebsiella pneumoniae | pKpQIL-SC29 | 2.0E-38 | 97.03 | 93 | 26 |
| NC_023314.1 | Klebsiella pneumoniae | pKPS30 | 1.0E-34 | 95.05 | 101 | 14 |
| NC_010726.1 | Klebsiella pneumoniae | pMET-1 | 4.0E-40 | 98.02 | 101 | 7 |
| NC_019158.1 | Klebsiella pneumoniae | pNDM10469 | 3.0E-34 | 95 | 100 | 9 |
| NC_021180.1 | Klebsiella pneumoniae | pNDM-1saitama01 | 2.0E-38 | 97.03 | 99 | 36 |
| NC_019153.1 | Klebsiella pneumoniae | pNDM-KN | 7.0E-37 | 96.04 | 101 | 2 |
| NC_016980.1 | Klebsiella pneumoniae | pNDM-MAR | 5.0E-35 | 96.04 | 90 | 37 |
| NC_014368.1 | Klebsiella pneumoniae | pNL194 | 6.0E-34 | 96.04 | 90 | 20 |
| NC_016976.1 | Klebsiella pneumoniae | pR55 | 7.0E-37 | 96.04 | 101 | 2 |
| NC_019152.1 | Klebsiella pneumoniae | pSLMT | 4.0E-43 | 100 | 101 | 9 |
| NC_016979.1 | Klebsiella pneumoniae | pUUH239.1 | 4.0E-42 | 99.01 | 101 | 2 |
| NC_014478.1 | Klebsiella pneumoniae | unnamed | 2.0E-35 | 95.05 | 91 | 166 |
| NC_019889.1 | Klebsiella pneumoniae strain 601 | pNDM-OM | 9.0E-39 | 100 | 94 | 1 |
| NC_019988.1 | Klebsiella pneumoniae strain BB1088 | pB1019 | 5.0E-34 | 96.7 | 91 | 21 |
| NC_019987.1 | Klebsiella pneumoniae strain BB1089 | pB1020 | 4.0E-39 | 97 | 100 | 2 |
| NC_019989.1 | Klebsiella pneumoniae strain BB1090 | pB1021 | 3.0E-41 | 98.02 | 101 | 6 |
| NC_019888.1 | Klebsiella pneumoniae strain BK31551 | pBK31551 | 2.0E-34 | 95.05 | 94 | 13 |
| NC_019899.1 | Klebsiella pneumoniae strain BK31567 | pBK31567 | 9.0E-36 | 95.05 | 99 | 18 |
| NC_020132.1 | Klebsiella pneumoniae strain BK32179 | pBK32179 | 1.0E-33 | 95.05 | 93 | 18 |
| NC_023331.1 | Klebsiella pneumoniae strain HS062105 | pHS062105-3 | 7.0E-43 | 100 | 101 | 1 |
| NC_021078.1 | Klebsiella pneumoniae strain Kp002 | pJEG011 | 2.0E-32 | 95 | 90 | 181 |
| NC_021238.1 | Klebsiella pneumoniae strain Kpn-1433 | pKP1433 | 2.0E-37 | 96.04 | 101 | 15 |
| NC_023330.1 | Klebsiella pneumoniae strain KPS77 | pKPS77 | 4.0E-33 | 95.05 | 91 | 47 |
| NC_022740.1 | Klebsiella pneumoniae strain MGR-K194 | pNDM_MGR194 | 2.0E-36 | 95.96 | 90 | 26 |
| NC_022609.1 | Klebsiella pneumoniae strain N11-0042 | pKp11-42 | 9.0E-35 | 95.88 | 90 | 124 |
| NC_023334.1 | Klebsiella pneumoniae strain ST15 | pKP02022 | 1.0E-40 | 99.01 | 101 | 14 |
| NC_023333.1 | Klebsiella pneumoniae strain ST23 | pKP007 | 2.0E-34 | 95.05 | 92 | 52 |
| NC_023332.1 | Klebsiella pneumoniae strain ST48 | pKP09085 | 6.0E-33 | 95.05 | 90 | 156 |
| NC_016838.1 | Klebsiella pneumoniae subsp. pneumoniae HS11286 | pKPHS1 | 1.0E-33 | 96.04 | 91 | 44 |
| NC_016846.1 | Klebsiella pneumoniae subsp. pneumoniae HS11286 | pKPHS2 | 2.0E-36 | 98.02 | 90 | 8 |
| NC_016839.1 | Klebsiella pneumoniae subsp. pneumoniae HS11286 | pKPHS3 | 2.0E-35 | 95.05 | 100 | 3 |
| NC_016840.1 | Klebsiella pneumoniae subsp. pneumoniae HS11286 | pKPHS4 | 1.0E-39 | 97.03 | 101 | 9 |
| NC_016847.1 | Klebsiella pneumoniae subsp. pneumoniae HS11286 | pKPHS5 | 7.0E-37 | 96.08 | 91 | 7 |
| NC_021198.1 | Klebsiella pneumoniae subsp. pneumoniae KPX | pKPX-1 | 4.0E-36 | 96.04 | 95 | 25 |
| NC_021199.1 | Klebsiella pneumoniae subsp. pneumoniae KPX | pKPX-2 | 3.0E-35 | 95.05 | 93 | 31 |
| NC_009649.1 | Klebsiella pneumoniae subsp. pneumoniae MGH 78578 | pKPN3 | 1.0E-33 | 95 | 93 | 110 |
| NC_009650.1 | Klebsiella pneumoniae subsp. pneumoniae MGH 78578 | pKPN4 | 4.0E-38 | 97.03 | 98 | 17 |
| NC_009651.1 | Klebsiella pneumoniae subsp. pneumoniae MGH 78578 | pKPN5 | 2.0E-35 | 95.05 | 90 | 57 |
| NC_009652.1 | Klebsiella pneumoniae subsp. pneumoniae MGH 78578 | pKPN6 | 4.0E-35 | 95.88 | 96 | 23 |
| NC_009653.1 | Klebsiella pneumoniae subsp. pneumoniae MGH 78578 | pKPN7 | 2.0E-38 | 98.02 | 91 | 8 |
| NC_003789.1 | Klebsiella sp. KCL-2 | pMGD2 | 6.0E-38 | 98.02 | 90 | 30 |
| NC_015213.1 | Lactobacillus acidophilus 30SC | pRKC30SC1 | 8.0E-40 | 98.02 | 94 | 107 |
| NC_003458.1 | Lactobacillus acidophilus | pLA103 | 2.0E-43 | 100 | 101 | 1 |
| NC_015319.1 | Lactobacillus amylovorus GRL 1112 | plasmid1 | 6.0E-43 | 100 | 101 | 3 |
| NC_017471.1 | Lactobacillus amylovorus GRL1118 | plasmid1 | 3.0E-42 | 99.01 | 101 | 5 |
| NC_017472.1 | Lactobacillus amylovorus GRL1118 | plasmid2 | 6.0E-34 | 95.05 | 91 | 100 |
| NC_008499.1 | Lactobacillus brevis ATCC 367 | 2 | 7.0E-36 | 95.05 | 101 | 4 |
| NC_020820.1 | Lactobacillus brevis KB290 | pKB290-1 | 2.0E-38 | 97.03 | 101 | 4 |
| NC_020821.1 | Lactobacillus brevis KB290 | pKB290-2 | 2.0E-35 | 95 | 97 | 5 |
| NC_020822.1 | Lactobacillus brevis KB290 | pKB290-4 | 9.0E-39 | 97.03 | 101 | 8 |
| NC_020823.1 | Lactobacillus brevis KB290 | pKB290-5 | 1.0E-41 | 99.01 | 101 | 2 |
| NC_020824.1 | Lactobacillus brevis KB290 | pKB290-7 | 8.0E-42 | 99.01 | 101 | 2 |
| NC_005952.1 | Lactobacillus brevis | pRH45II | 6.0E-41 | 99 | 99 | 2 |
| NC_018611.1 | Lactobacillus buchneri CD034 | pCD034-3 | 9.0E-43 | 100 | 101 | 2 |
| NC_015420.1 | Lactobacillus buchneri NRRL B-30929 | pLBUC01 | 9.0E-43 | 100 | 101 | 1 |
| NC_015429.1 | Lactobacillus buchneri NRRL B-30929 | pLBUC02 | 4.0E-36 | 95.05 | 101 | 11 |
| NC_015421.1 | Lactobacillus buchneri NRRL B-30929 | pLBUC03 | 9.0E-41 | 98.02 | 101 | 4 |
| NC_008502.1 | Lactobacillus casei ATCC 334 | 1 | 1.0E-37 | 99.01 | 91 | 7 |
| NC_017476.1 | Lactobacillus casei BD-II | pBD-II | 9.0E-37 | 96 | 100 | 3 |
| NC_017475.1 | Lactobacillus casei LC2W | pLC2W | 7.0E-36 | 95.05 | 90 | 29 |
| NC_021722.1 | Lactobacillus casei LOCK919 | pLOCK919 | 1.0E-37 | 96.04 | 96 | 5 |
| NC_011352.1 | Lactobacillus casei str. Zhang | plca36 | 7.0E-36 | 95.05 | 99 | 28 |
| NC_016975.1 | Lactobacillus casei strain TISTR1341 | pRCEID7.6 | 7.0E-41 | 98.02 | 101 | 3 |
| NC_020057.1 | Lactobacillus casei W56 | pW56 | 2.0E-38 | 97.03 | 100 | 22 |
| NC_003320.2 | Lactobacillus curvatus strain CRL705 | pRC18 | 7.0E-39 | 97.03 | 101 | 2 |
| NC_011798.1 | Lactobacillus farciminis KCTC 3681 | pLF24 | 3.0E-40 | 98.02 | 101 | 1 |
| NC_004947.1 | Lactobacillus fermentum | pKC5b | 9.0E-37 | 95.05 | 100 | 18 |
| NC_011839.1 | Lactobacillus gasseri | pLgLA39 | 1.0E-38 | 97.03 | 99 | 9 |
| NC_002102.1 | Lactobacillus helveticus DSM 20075 | pLH1 | 4.0E-36 | 95.05 | 101 | 3 |
| NC_017468.1 | Lactobacillus helveticus H10 | pH10 | 4.0E-43 | 100 | 101 | 1 |
| NC_014386.1 | Lactobacillus helveticus R0052 | pIR52-1 | 1.0E-36 | 95.05 | 101 | 2 |
| NC_013505.1 | Lactobacillus johnsonii FI9785 | p9785L | 5.0E-36 | 95.05 | 98 | 4 |
| NC_015598.1 | Lactobacillus kefiranofaciens ZW3 | pWW1 | 4.0E-35 | 95.05 | 91 | 7 |
| NC_015603.1 | Lactobacillus kefiranofaciens ZW3 | pWW2 | 4.0E-40 | 98.02 | 101 | 2 |
| NC_021573.1 | Lactobacillus paracasei | pLP5402 | 1.0E-43 | 100 | 101 | 1 |
| NC_010913.1 | Lactobacillus paracasei | pMA3 | 9.0E-44 | 100 | 101 | 1 |
| NC_022114.1 | Lactobacillus paracasei subsp. paracasei 8700:2 | 1 | 3.0E-39 | 97.98 | 99 | 4 |
| NC_022123.1 | Lactobacillus paracasei subsp. paracasei 8700:2 | 2 | 4.0E-35 | 95 | 100 | 7 |
| NC_013543.1 | Lactobacillus paracasei subsp. paracasei | pCD01 | 2.0E-40 | 98.02 | 101 | 6 |
| NC_013544.1 | Lactobacillus paracasei subsp. paracasei | pCD02 | 4.0E-38 | 96.04 | 101 | 10 |
| NC_012222.2 | Lactobacillus paracasei subsp. paracasei | pSJ2-8 | 1.0E-40 | 98.02 | 101 | 9 |
| NC_021516.1 | Lactobacillus plantarum 16 | Lp16C | 1.0E-36 | 95.96 | 99 | 2 |
| NC_021526.1 | Lactobacillus plantarum 16 | Lp16D | 7.0E-36 | 95.05 | 90 | 14 |
| NC_021517.1 | Lactobacillus plantarum 16 | Lp16E | 3.0E-41 | 99.01 | 101 | 2 |
| NC_021518.1 | Lactobacillus plantarum 16 | Lp16F | 2.0E-37 | 96.04 | 101 | 6 |
| NC_021527.1 | Lactobacillus plantarum 16 | Lp16G | 9.0E-43 | 100 | 101 | 2 |
| NC_021519.1 | Lactobacillus plantarum 16 | Lp16H | 3.0E-38 | 97.03 | 101 | 2 |
| NC_021528.1 | Lactobacillus plantarum 16 | Lp16I | 2.0E-38 | 97.06 | 93 | 4 |
| NC_004944.1 | Lactobacillus plantarum 5057 | pMD5057 | 5.0E-38 | 96.04 | 101 | 20 |
| NC_006278.1 | Lactobacillus plantarum NC7 | p256 | 1.0E-43 | 100 | 101 | 2 |
| NC_019379.1 | Lactobacillus plantarum | pG6302 | 1.0E-39 | 98.02 | 100 | 5 |
| NC_019371.1 | Lactobacillus plantarum | pG6303 | 9.0E-41 | 98.02 | 101 | 3 |
| NC_012628.1 | Lactobacillus plantarum | pLFE1 | 1.0E-35 | 96.81 | 94 | 115 |
| NC_011101.1 | Lactobacillus plantarum | pLTK13 | 2.0E-41 | 99.01 | 101 | 1 |
| NC_021233.1 | Lactobacillus plantarum subsp. plantarum P-8 | LBPp1 | 2.0E-37 | 96.04 | 101 | 14 |
| NC_021225.1 | Lactobacillus plantarum subsp. plantarum P-8 | LBPp2 | 2.0E-37 | 96.04 | 101 | 11 |
| NC_021226.1 | Lactobacillus plantarum subsp. plantarum P-8 | LBPp3 | 1.0E-38 | 97.03 | 95 | 4 |
| NC_021234.1 | Lactobacillus plantarum subsp. plantarum P-8 | LBPp4 | 3.0E-40 | 98.02 | 101 | 4 |
| NC_021227.1 | Lactobacillus plantarum subsp. plantarum P-8 | LBPp5 | 3.0E-43 | 100 | 101 | 1 |
| NC_021228.1 | Lactobacillus plantarum subsp. plantarum P-8 | LBPp6 | 1.0E-43 | 100 | 101 | 1 |
| NC_014558.2 | Lactobacillus plantarum subsp. plantarum ST-III | pST-III | 2.0E-37 | 96.04 | 100 | 11 |
| NC_006376.1 | Lactobacillus plantarum WCFS1 | pWCFS102 | 6.0E-36 | 95.05 | 91 | 3 |
| NC_006377.1 | Lactobacillus plantarum WCFS1 | pWCFS103 | 2.0E-37 | 97.92 | 96 | 10 |
| NC_021903.1 | Lactobacillus plantarum ZJ316 | pLP-ZJ101 | 3.0E-43 | 100 | 101 | 1 |
| NC_021904.1 | Lactobacillus plantarum ZJ316 | pLP-ZJ102 | 6.0E-43 | 100 | 101 | 2 |
| NC_021912.1 | Lactobacillus plantarum ZJ316 | pLP-ZJ103 | 4.0E-39 | 97.98 | 99 | 4 |
| NC_019233.1 | Lactobacillus rennini | pREN | 3.0E-42 | 99.01 | 101 | 3 |
| NC_021503.1 | Lactobacillus reuteri I5007 | pLRI01 | 1.0E-35 | 95.05 | 96 | 40 |
| NC_021495.1 | Lactobacillus reuteri I5007 | pLRI02 | 9.0E-37 | 97.03 | 94 | 11 |
| NC_021496.1 | Lactobacillus reuteri I5007 | pLRI03 | 7.0E-43 | 100 | 101 | 1 |
| NC_021504.1 | Lactobacillus reuteri I5007 | pLRI04 | 2.0E-33 | 95.05 | 91 | 145 |
| NC_021497.1 | Lactobacillus reuteri I5007 | pLRI05 | 2.0E-37 | 96.04 | 93 | 13 |
| NC_003528.1 | Lactobacillus reuteri | pTE44 | 2.0E-38 | 99.01 | 91 | 20 |
| NC_015701.1 | Lactobacillus reuteri SD2112 | pLR584 | 1.0E-36 | 97.03 | 92 | 36 |
| NC_010603.1 | Lactobacillus reuteri strain ATCC 55730 | pLR581 | 3.0E-34 | 95.05 | 90 | 194 |
| NC_010621.1 | Lactobacillus reuteri strain ATCC 55730 | pLR585 | 2.0E-43 | 100 | 101 | 1 |
| NC_011223.1 | Lactobacillus rhamnosus HN001 | pLR001 | 1.0E-37 | 100 | 90 | 2 |
| NC_011225.1 | Lactobacillus rhamnosus HN001 | pLR002 | 2.0E-41 | 99.01 | 101 | 11 |
| NC_013200.1 | Lactobacillus rhamnosus Lc 705 | pLC1 | 1.0E-34 | 95.05 | 91 | 35 |
| NC_004942.1 | Lactobacillus sakei | pRV500 | 9.0E-42 | 99.01 | 101 | 2 |
| NC_011652.1 | Lactobacillus sakei strain BM5 | pYC2 | 4.0E-37 | 95.05 | 99 | 3 |
| NC_017479.1 | Lactobacillus salivarius CECT 5713 | pHN1 | 2.0E-32 | 95.6 | 91 | 72 |
| NC_017480.1 | Lactobacillus salivarius CECT 5713 | pHN2 | 4.0E-36 | 95.05 | 101 | 9 |
| NC_017499.1 | Lactobacillus salivarius CECT 5713 | pHN3 | 4.0E-35 | 95.05 | 97 | 57 |
| NC_007930.1 | Lactobacillus salivarius UCC118 | pMP118 | 1.0E-36 | 97.03 | 91 | 16 |
| NC_006529.1 | Lactobacillus salivarius UCC118 | pSF118-20 | 8.0E-39 | 97.03 | 99 | 9 |
| NC_006530.1 | Lactobacillus salivarius UCC118 | pSF118-44 | 1.0E-32 | 95.65 | 92 | 26 |
| NC_015979.1 | Lactobacillus sanfranciscensis TMW 1.1304 | pLS1 | 1.0E-35 | 95.05 | 100 | 15 |
| NC_015980.1 | Lactobacillus sanfranciscensis TMW 1.1304 | pLS2 | 1.0E-41 | 99.01 | 101 | 1 |
| NC_004957.1 | Lactobacillus sp. PC121B | p121BS | 7.0E-44 | 100 | 101 | 1 |
| NC_016970.1 | Lactococcus garvieae 21881 | pGL3 | 3.0E-35 | 97.03 | 91 | 9 |
| NC_016971.1 | Lactococcus garvieae 21881 | pGL4 | 2.0E-38 | 99.01 | 92 | 16 |
| NC_016982.1 | Lactococcus garvieae 21881 | pGL5 | 5.0E-41 | 99.01 | 101 | 1 |
| NC_010540.1 | Lactococcus garvieae | pKL0018 | 9.0E-38 | 96.04 | 93 | 73 |
| NC_007191.1 | Lactococcus lactis cremoris 712 | pAG6 | 1.0E-38 | 98.99 | 92 | 30 |
| NC_004847.1 | Lactococcus lactis cremoris HP | pHP003 | 3.0E-36 | 95.05 | 98 | 9 |
| NC_002137.1 | Lactococcus lactis cremoris NIZO B40 | pNZ4000 | 8.0E-36 | 95.05 | 99 | 45 |
| NC_003101.1 | Lactococcus lactis CRL1127 | pCRL1127 | 2.0E-36 | 95.05 | 96 | 22 |
| NC_002798.1 | Lactococcus lactis DCH-4 | pSRQ700 | 2.0E-36 | 95.05 | 92 | 16 |
| NC_001949.1 | Lactococcus lactis DPC3147 | pMRC01 | 4.0E-35 | 98.02 | 90 | 18 |
| NC_000906.2 | Lactococcus lactis IL964 | pIL105 | 2.0E-36 | 96.94 | 93 | 14 |
| NC_004966.1 | Lactococcus lactis lactis bv. diacetylactis DPC220 | pAH82 | 4.0E-36 | 95.05 | 91 | 47 |
| NC_004164.2 | Lactococcus lactis lactis bv. diacetylactis DRC1 cryptic | pDR1-1 | 3.0E-33 | 95 | 90 | 7 |
| NC_004955.1 | Lactococcus lactis lactis IPLA 972 | pBL1 | 5.0E-38 | 96.04 | 99 | 15 |
| NC_002502.1 | Lactococcus lactis lactis UC317 | pCI305 | 6.0E-36 | 95 | 100 | 4 |
| NC_004981.2 | Lactococcus lactis ML8 | pCL2.1 | 4.0E-37 | 95.05 | 101 | 3 |
| NC_009435.1 | Lactococcus lactis NCDO 1867 | pGdh442 | 1.0E-35 | 95.05 | 90 | 39 |
| NC_002150.1 | Lactococcus lactis | pAH33 | 2.0E-40 | 98 | 100 | 19 |
| NC_002799.1 | Lactococcus lactis | pCRL291.1 | 2.0E-38 | 98.02 | 91 | 5 |
| NC_011610.1 | Lactococcus lactis | pKL001 | 5.0E-35 | 95.79 | 95 | 5 |
| NC_008436.1 | Lactococcus lactis | pND324 | 3.0E-41 | 98.02 | 101 | 2 |
| NC_010901.1 | Lactococcus lactis | pNP40 | 1.0E-35 | 95.05 | 100 | 15 |
| NC_013551.1 | Lactococcus lactis | pSK11B | 4.0E-40 | 97.12 | 97 | 5 |
| NC_004980.1 | Lactococcus lactis | pWC1 | 6.0E-37 | 95.05 | 101 | 2 |
| NC_017497.1 | Lactococcus lactis subsp. cremoris A76 | pQA504 | 2.0E-39 | 100 | 93 | 4 |
| NC_017495.1 | Lactococcus lactis subsp. cremoris A76 | pQA518 | 8.0E-38 | 96.04 | 97 | 10 |
| NC_017493.1 | Lactococcus lactis subsp. cremoris A76 | pQA549 | 4.0E-40 | 98.02 | 101 | 4 |
| NC_017496.1 | Lactococcus lactis subsp. cremoris A76 | pQA554 | 4.0E-34 | 95 | 90 | 162 |
| NC_019347.1 | Lactococcus lactis subsp. cremoris | pAF04 | 6.0E-38 | 96.04 | 93 | 12 |
| NC_019349.1 | Lactococcus lactis subsp. cremoris | pAF12 | 6.0E-38 | 96.04 | 99 | 16 |
| NC_019350.1 | Lactococcus lactis subsp. cremoris | pAF14 | 1.0E-35 | 95 | 90 | 62 |
| NC_019351.1 | Lactococcus lactis subsp. cremoris | pAF22 | 7.0E-40 | 98 | 100 | 2 |
| NC_017494.1 | Lactococcus lactis subsp. cremoris | pAW153 | 5.0E-42 | 99.01 | 101 | 1 |
| NC_019377.1 | Lactococcus lactis subsp. cremoris | pLP712 | 3.0E-36 | 97.87 | 94 | 7 |
| NC_008503.1 | Lactococcus lactis subsp. cremoris SK11 | 1 | 4.0E-40 | 98.02 | 98 | 18 |
| NC_008504.1 | Lactococcus lactis subsp. cremoris SK11 | 2 | 2.0E-43 | 100 | 101 | 5 |
| NC_008505.1 | Lactococcus lactis subsp. cremoris SK11 | 3 | 9.0E-32 | 95 | 93 | 90 |
| NC_008506.1 | Lactococcus lactis subsp. cremoris SK11 | 4 | 3.0E-35 | 96.04 | 90 | 80 |
| NC_008507.1 | Lactococcus lactis subsp. cremoris SK11 | 5 | 5.0E-39 | 97.03 | 101 | 9 |
| NC_019438.1 | Lactococcus lactis subsp. cremoris UC509.9 | pCIS1 | 5.0E-40 | 98.97 | 97 | 5 |
| NC_019434.1 | Lactococcus lactis subsp. cremoris UC509.9 | pCIS2 | 2.0E-39 | 98.02 | 93 | 8 |
| NC_019437.1 | Lactococcus lactis subsp. cremoris UC509.9 | pCIS4 | 1.0E-36 | 96.04 | 91 | 17 |
| NC_019432.1 | Lactococcus lactis subsp. cremoris UC509.9 | pCIS5 | 2.0E-36 | 95.92 | 98 | 13 |
| NC_019436.1 | Lactococcus lactis subsp. cremoris UC509.9 | pCIS6 | 9.0E-35 | 95 | 91 | 12 |
| NC_019431.1 | Lactococcus lactis subsp. cremoris UC509.9 | pCIS7 | 2.0E-38 | 97.03 | 97 | 18 |
| NC_019430.1 | Lactococcus lactis subsp. cremoris UC509.9 | pCIS8 | 3.0E-38 | 97.03 | 101 | 5 |
| NC_004163.1 | Lactococcus lactis subsp. lactis bv. diacetylactis cryptic | pDR1-1B | 2.0E-42 | 100 | 99 | 1 |
| NC_009137.1 | Lactococcus lactis subsp. lactis bv. diacetylactis | pDBORO | 7.0E-38 | 96.04 | 91 | 27 |
| NC_004652.1 | Lactococcus lactis subsp. lactis bv. diacetylactis | pS7a | 1.0E-36 | 95.05 | 101 | 15 |
| NC_015900.1 | Lactococcus lactis subsp. lactis bv. diacetylactis | pVF18 | 4.0E-36 | 95.05 | 90 | 48 |
| NC_015912.1 | Lactococcus lactis subsp. lactis bv. diacetylactis | pVF21 | 4.0E-36 | 95.05 | 101 | 28 |
| NC_015901.1 | Lactococcus lactis subsp. lactis bv. diacetylactis | pVF22 | 8.0E-39 | 97.03 | 101 | 30 |
| NC_015902.1 | Lactococcus lactis subsp. lactis bv. diacetylactis | pVF50 | 1.0E-35 | 95.05 | 96 | 47 |
| NC_017483.1 | Lactococcus lactis subsp. lactis CV56 | pCV56A | 4.0E-40 | 98.02 | 100 | 14 |
| NC_017487.1 | Lactococcus lactis subsp. lactis CV56 | pCV56B | 8.0E-35 | 95.05 | 96 | 36 |
| NC_017484.1 | Lactococcus lactis subsp. lactis CV56 | pCV56C | 2.0E-35 | 95.05 | 94 | 114 |
| NC_009751.1 | Lactococcus lactis subsp. lactis K214 | pK214 | 3.0E-33 | 95.7 | 93 | 36 |
| NC_013657.1 | Lactococcus lactis subsp. lactis KF147 | pKF147A | 1.0E-32 | 95.05 | 90 | 40 |
| NC_013783.1 | Lactococcus lactis subsp. lactis | pAR141 | 6.0E-40 | 97.03 | 101 | 2 |
| NC_015860.1 | Lactococcus lactis subsp. lactis | pIL1 | 1.0E-36 | 95.05 | 101 | 11 |
| NC_015861.1 | Lactococcus lactis subsp. lactis | pIL3 | 4.0E-36 | 95.05 | 96 | 28 |
| NC_015862.1 | Lactococcus lactis subsp. lactis | pIL4 | 2.0E-37 | 96.04 | 91 | 24 |
| NC_015863.1 | Lactococcus lactis subsp. lactis | pIL5 | 8.0E-33 | 95.65 | 92 | 55 |
| NC_019308.1 | Lactococcus lactis subsp. lactis | pIL6 | 5.0E-36 | 95.05 | 100 | 37 |
| NC_015864.1 | Lactococcus lactis subsp. lactis | pIL7 | 5.0E-37 | 96 | 92 | 21 |
| NC_016042.1 | Lactococcus lactis subsp. lactis | pKP1 | 3.0E-36 | 95.05 | 94 | 48 |
| NC_008594.1 | Lactococcus lactis subsp. lactis | pL2 | 2.0E-38 | 96.04 | 99 | 16 |
| NC_002748.1 | Lactococcus lactis subsp. lactis strain MJC15 | pCD4 | 1.0E-35 | 95.05 | 94 | 7 |
| NC_004960.1 | Lactococcus lactis W-1 | pSRQ800 | 1.0E-37 | 96.94 | 98 | 6 |
| NC_004959.1 | Lactococcus lactis W-37 | pSRQ900 | 1.0E-40 | 98.02 | 99 | 11 |
| NC_010370.1 | Laribacter hongkongensis | pHLHK22 | 2.0E-37 | 96 | 100 | 26 |
| NC_006628.1 | Laribacter hongkongensis | pHLHK8 | 6.0E-36 | 95 | 100 | 9 |
| NC_018674.1 | Leuconostoc carnosum JB16 | pKLC1 | 2.0E-40 | 98.02 | 101 | 4 |
| NC_018675.1 | Leuconostoc carnosum JB16 | pKLC3 | 9.0E-35 | 95.05 | 101 | 2 |
| NC_018699.1 | Leuconostoc carnosum JB16 | pKLC4 | 7.0E-36 | 95.05 | 101 | 2 |
| NC_010470.1 | Leuconostoc citreum KM20 | pLCK1 | 9.0E-35 | 95.05 | 91 | 22 |
| NC_010466.1 | Leuconostoc citreum KM20 | pLCK2 | 1.0E-31 | 95.56 | 90 | 5 |
| NC_010469.1 | Leuconostoc citreum KM20 | pLCK4 | 2.0E-36 | 95.05 | 100 | 32 |
| NC_014132.1 | Leuconostoc kimchii IMSNU 11154 | LkipL4704 | 1.0E-37 | 96.97 | 99 | 4 |
| NC_014133.1 | Leuconostoc kimchii IMSNU 11154 | LkipL4719 | 1.0E-35 | 95 | 100 | 7 |
| NC_014134.1 | Leuconostoc kimchii IMSNU 11154 | LkipL4726 | 7.0E-35 | 95.05 | 92 | 7 |
| NC_004992.1 | Leuconostoc lactis | pCI411 | 2.0E-37 | 98.91 | 92 | 2 |
| NC_008496.1 | Leuconostoc mesenteroides subsp. mesenteroides ATCC 8293 | pLEUM1 | 9.0E-35 | 95.05 | 96 | 33 |
| NC_016820.1 | Leuconostoc mesenteroides subsp. mesenteroides J18 | pKLE02 | 2.0E-42 | 100 | 100 | 4 |
| NC_016821.1 | Leuconostoc mesenteroides subsp. mesenteroides J18 | pKLE03 | 4.0E-43 | 100 | 101 | 1 |
| NC_003383.1 | Listeria innocua Clip11262 | pLI100 | 4.0E-37 | 96.04 | 99 | 11 |
| NC_018888.1 | Listeria monocytogenes serotype 7 str. SLCC2482 | pLM7UG1 | 4.0E-40 | 98.02 | 101 | 12 |
| NC_014495.1 | Listeria monocytogenes SLCC2755 | pLM1-2bUG1 | 3.0E-37 | 96.04 | 95 | 35 |
| NC_022051.1 | Listeria monocytogenes strain J1926 |  | 5.0E-40 | 98.02 | 101 | 11 |
| NC_014255.1 | Listeria monocytogenes strain Lm1 | pLM33 | 2.0E-41 | 99.01 | 101 | 3 |
| NC_022045.1 | Listeria monocytogenes strain N1-011A |  | 3.0E-35 | 95.05 | 101 | 1 |
| NC_021828.1 | Listeria monocytogenes strain R2-502 |  | 1.0E-35 | 95.05 | 101 | 10 |
| NC_002637.1 | Mannheimia haemolytica R122 | pMHSCS1 | 4.0E-41 | 100 | 96 | 1 |
| NC_008739.1 | Marinobacter aquaeolei VT8 | pMAQU02 | 8.0E-38 | 97.03 | 101 | 14 |
| NC_014213.1 | Meiothermus silvanus DSM 9946 | pMESIL01 | 2.0E-40 | 99.01 | 101 | 1 |
| NC_002679.1 | Mesorhizobium loti MAFF303099 | pMLa | 5.0E-36 | 96 | 100 | 3 |
| NC_008242.1 | Mesorhizobium sp. BNC1 | 1 | 2.0E-34 | 95 | 95 | 88 |
| NC_008243.1 | Mesorhizobium sp. BNC1 | 2 | 2.0E-31 | 95 | 91 | 36 |
| NC_019972.1 | Methanomethylovorans hollandica DSM 15978 | pMETHO01 | 5.0E-42 | 100 | 101 | 3 |
| NC_007349.1 | Methanosarcina barkeri str. fusaro | 1 | 2.0E-37 | 96.04 | 101 | 1 |
| NC_008826.1 | Methylibium petroleiphilum PM1 | RPME01 | 4.0E-34 | 95 | 100 | 3 |
| NC_012811.1 | Methylobacterium extorquens AM1 |  | 9.0E-33 | 95 | 90 | 250 |
| NC_012809.1 | Methylobacterium extorquens AM1 | p2META1 | 7.0E-36 | 95.05 | 101 | 1 |
| NC_011758.1 | Methylobacterium extorquens CM4 | pMCHL01 | 6.0E-42 | 100 | 101 | 3 |
| NC_011892.1 | Methylobacterium nodulans ORS 2060 | pMNOD01 | 9.0E-35 | 95.05 | 99 | 4 |
| NC_010721.1 | Methylobacterium populi BJ001 | pMPOP02 | 4.0E-43 | 100 | 101 | 1 |
| NC_010510.1 | Methylobacterium radiotolerans JCM 2831 | pMRAD01 | 8.0E-30 | 95 | 90 | 153 |
| NC_004954.1 | Micrococcus sp. 28 | pSD10 | 9.0E-36 | 95.05 | 91 | 34 |
| NC_022599.1 | Micrococcus sp. V7 | pLMV7 | 9.0E-33 | 95.7 | 93 | 10 |
| NC_019760.1 | Microcoleus sp. PCC 7113 | pMIC7113.02 | 1.0E-31 | 95 | 90 | 51 |
| NC_016036.1 | Morganella morganii strain M203 | R485 | 1.0E-35 | 95.05 | 99 | 18 |
| NC_021278.1 | Mycobacterium abscessus subsp. bolletii 50594 | 1 | 1.0E-39 | 98.02 | 101 | 1 |
| NC_021279.1 | Mycobacterium abscessus subsp. bolletii 50594 | 2 | 2.0E-42 | 100 | 101 | 1 |
| NC_017908.2 | Mycobacterium abscessus subsp. bolletii F1725 | BRA100 | 1.0E-35 | 95.05 | 91 | 61 |
| NC_020994.1 | Mycobacterium abscessus subsp. bolletii INCQS 00594 | pMAB01 | 9.0E-37 | 96.04 | 90 | 2944 |
| NC_018022.1 | Mycobacterium chubuense NBB4 | pMYCCH.01 | 2.0E-37 | 97.96 | 98 | 1 |
| NC_009339.1 | Mycobacterium gilvum PYR-GCK | pMFLV01 | 6.0E-35 | 95.05 | 101 | 1 |
| NC_014811.1 | Mycobacterium gilvum Spyr1 | pMSPYR101 | 3.0E-36 | 96.94 | 98 | 1 |
| NC_008703.1 | Mycobacterium sp. KMS | pMKMS01 | 1.0E-36 | 96.04 | 93 | 8 |
| NC_008704.1 | Mycobacterium sp. KMS | pMKMS02 | 1.0E-34 | 95 | 100 | 1 |
| NC_010189.1 | Naegleria gruberi | extrachromosomal rDNA | 3.0E-36 | 95.05 | 101 | 1 |
| NC_019211.1 | Neisseria gonorrhoeae | pEM1 | 8.0E-44 | 100 | 101 | 4 |
| NC_014105.1 | Neisseria gonorrhoeae | pEP5289 | 7.0E-43 | 100 | 101 | 1 |
| NC_007959.1 | Nitrobacter hamburgensis X14 | 1 | 1.0E-36 | 96.04 | 101 | 2 |
| NC_007960.1 | Nitrobacter hamburgensis X14 | 2 | 3.0E-36 | 96 | 99 | 5 |
| NC_007961.1 | Nitrobacter hamburgensis X14 | 3 | 8.0E-35 | 97.03 | 90 | 2 |
| NC_008341.1 | Nitrosomonas eutropha C91 | plasmid1 | 1.0E-34 | 95.05 | 96 | 28 |
| NC_008342.1 | Nitrosomonas eutropha C91 | plasmid2 | 1.0E-35 | 95.05 | 101 | 2 |
| NC_015223.1 | Nitrosomonas sp. AL212 | pNAL21201 | 2.0E-35 | 95.05 | 101 | 7 |
| NC_015221.1 | Nitrosomonas sp. AL212 | pNAL21202 | 2.0E-32 | 95.05 | 92 | 16 |
| NC_006363.1 | Nocardia farcinica IFM 10152 | pNF2 | 2.0E-35 | 95.92 | 98 | 1 |
| NC_008697.1 | Nocardioides sp. JS614 | pNOCA01 | 6.0E-35 | 95.05 | 101 | 1 |
| NC_002033.1 | Novosphingobium aromaticivorans | pNL1 | 6.0E-38 | 97.03 | 101 | 3 |
| NC_015583.1 | Novosphingobium sp. PP1Y | Mpl | 2.0E-34 | 95.05 | 101 | 6 |
| NC_009669.1 | Ochrobactrum anthropi ATCC 49188 | pOANT01 | 2.0E-32 | 95.05 | 90 | 226 |
| NC_009670.1 | Ochrobactrum anthropi ATCC 49188 | pOANT02 | 4.0E-37 | 96.04 | 91 | 24 |
| NC_009671.1 | Ochrobactrum anthropi ATCC 49188 | pOANT03 | 6.0E-35 | 95 | 100 | 14 |
| NC_019553.1 | Oenococcus oeni | pOENI-1 | 8.0E-38 | 97.03 | 96 | 8 |
| NC_019554.1 | Oenococcus oeni | pOENI-1v2 | 4.0E-43 | 100 | 101 | 2 |
| NC_017536.1 | Oligotropha carboxidovorans OM4 | pHCG3B | 6.0E-37 | 96.97 | 99 | 1 |
| NC_017539.1 | Oligotropha carboxidovorans OM4 | pOC167B | 3.0E-36 | 96 | 97 | 3 |
| NC_011414.1 | Ornithobacterium rhinotracheale | pOR1 | 6.0E-32 | 95.56 | 90 | 10 |
| NC_017533.1 | Pantoea ananatis AJ13355 | pEA320 | 6.0E-35 | 95.05 | 101 | 1 |
| NC_016817.1 | Pantoea ananatis LMG 5342 | pPANA10 | 7.0E-40 | 100 | 97 | 9 |
| NC_014842.1 | Pantoea sp. At-9b | pPAT9B05 | 8.0E-41 | 99.01 | 101 | 2 |
| NC_014561.1 | Pantoea vagans C9-1 | pPag1 | 1.0E-40 | 99.01 | 101 | 1 |
| NC_014258.1 | Pantoea vagans C9-1 | pPag3 | 2.0E-30 | 95.05 | 94 | 8 |
| NC_019316.1 | Paracoccus aestuarii | pAES4 | 3.0E-38 | 96.97 | 99 | 2 |
| NC_022042.1 | Paracoccus aminophilus JCM 7686 | pAMI1 | 6.0E-30 | 95 | 90 | 325 |
| NC_022049.1 | Paracoccus aminophilus JCM 7686 | pAMI4 | 3.0E-34 | 95 | 98 | 4 |
[truncated: 61,972 more chars]
